# Establishment and characterization of Hanwoo cumulus cell line for heat stress studies

**DOI:** 10.5713/ab.250896

**Published:** 2026-06-15

**Authors:** Ismail Shaleh, Sung Woo Kim, Tae Sub Park, Joonghoon Park

**Affiliations:** 1 Graduate School of Int’l Agriculture Technology, Green Bio Science & Technology, Seoul National University, Pyeongchang, Korea; 2 Department of Biology, Faculty of Mathematics and Natural Science, IPB University, Bogor, Indonesia; 3 Hanwoo Research Institute, National Institute of Animal Science, Rural Development Administration, Pyeongchang, Korea; 4 Department of Agricultural Biotechnology, Research Institute of Agriculture and Life Science, Seoul National University, Seoul, Korea

**Keywords:** Cattle Reproduction, Hanwoo Cattle, Heat Stress, Immortalized Cumulus Cells, Proteomics, Transcriptomics

## Abstract

**Objective:**

Heat stress (HS) negatively affects cattle reproduction, decreasing livestock productivity. HS response in cattle reproductive cumulus cells remain understudied due to limited cell lines and comprehensive omics data. This study aims to establish immortalized cumulus cells from Hanwoo cattle as a HS model and explores their HS response using molecular markers including transcriptomics and proteomics approaches.

**Methods:**

Immortalized cumulus cells were established from primary cumulus isolated from fresh Hanwoo cattle follicles by using *piggyBac* transposon-mediated SV40T expression system. HS condition was optimized using reactive oxygen species (ROS) detection, quantitative reverse-transcription polymerase chain reaction, and Western blotting on oxidative and endoplasmic reticulum stress markers under different HS and recovery periods (4 h, 12 h, and 24 h). Transcriptomic and proteomic data were generated to identify HS-related differentially expressed genes (DEG) and proteins (DEP) utilized for gene ontology (GO) and pathway enrichment analysis.

**Results:**

We established the first immortalized cumulus cell line with high specificity (99% CD44[+]) for *in vitro* HS study. ROS accumulation was observed at 4 h HS, leading to immediate upregulation of HSP70 and BiP proteins, with oxidative stress gene expression (*SOD1, CAT, GPX1*) peaked at 12 h HS. We identified 754 DEGs (fold change≥|2|) and 357 DEPs (fold change≥|1.5|) related to HS in the cumulus cell line. Enrichment analysis exhibited upregulation in GO and pathways related to protein unfolding and cellular response to HS, respectively, from both the DEG and DEP list. Downregulated DEG and DEP showed significant enrichment in GO related to the extracellular matrix, potentially affecting cumulus-oocyte complex communication for reproductive function.

**Conclusion:**

The cumulus cell line serves as a robust *in vitro* model for studying HS response in cattle reproductive system. The multi-omics data could help clarify the comprehensive HS response and identify possible molecular targets to mitigate negative HS effects in the future.

## INTRODUCTION

Amid global warming, heat stress (HS) has been well known hindering reproductive capacity in livestock animals including cattle. At cellular level, elevated temperature disturbs mitochondrial permeability and elevates intracellular reactive oxygen species (ROS) causing physiological imbalance and apoptosis in cattle reproductive cells [1,2]. Hyperthermal condition at thermal humidity index≥72 is also dramatically lowering cattle reproductive capacity by compromising oocyte maturation and early embryo development [3]. Moreover, this chronical effects can be prolonged post-HS period and even impairs cattle progenies leading to a progressive reduction in livestock performance across generations [4]. Unfortunately, recent mitigation strategies to improve cattle reproductive capacity under HS are still limited to technical supports such as cooling system and in-vitro fertilization [5] while targeted molecular approach to alleviate heat strain in cattle reproduction is still lacking.

Cumulus cells are specialized somatic cells derived from granulosa cells that surround oocyte in a unique cumulus-oocyte complex (COC) structure [6]. Tight connection of cumulus with oocyte in COC play pivotal roles in oocyte growth, maturation, ovulation, fertilization, and early embryonic development through their bi-directional communication and nutritional support via gap junction [6–9]. Cumulus cells protect and support oocyte competence by providing microenvironment of hyaluronan-rich extracellular matrix (ECM) during cumulus expansion where intricate intercellular signaling occurred such as upregulation of ERK1/2-dependent pathway for oocyte meiotic maturation, toll-like receptor for follicular innate immune response, and Ca^2+^-dependent signaling for acrosomal activity during fertilization [6,7,10]. Despite the crucial functions, currently there is no cell line of bovine cumulus cell line exists. Therefore, establishment of cumulus cell line is a major step to overcome limitations on the cumulus primary cell study and to increase experimental reproducibility and scalability.

Detrimental effects caused by HS promote adaptive response of bovine cumulus cells in supporting oocyte maturation and fertilization. Previous *in vitro* study found that HS at 41°C for 12 hours trigger ROS accumulation, reduce cellular proliferation, cause early follicle maturation, decrease estradiol and progesterone secretion, and hinder oocyte nuclear maturation in bovine COC [11,12]. To alleviate the damaging ROS effect, bovine cumulus cells under HS activate oxidative defense mechanisms through upregulation of antioxidant protein/genes such as HO-1, SOD2, NRF2, catalase (*CAT*), and *PRDX1* preventing cellular apoptosis [12,13]. Furthermore, hyperthermic condition also causes cytoplasmic protein misfolding and endoplasmic reticulum (ER) stress which induce upregulation of protein repair mechanisms from various chaperone molecules including HSP70, HSP90 and BiP/GRP78 [12–15]. In addition, high-throughput approach using transcriptomics also reveal alteration of differentially expressed genes (DEG) related to cell junctions or adhesion molecules in both *in vitro* and *in vivo* study of bovine cumulus cells under HS [16,17]. On the other hand, proteomics study of differentially expressed proteins (DEP) in bovine cumulus cells study under HS is recently very limited. Indeed, the integration of multi-omics approaches has recently gained increasing attention for elucidating the effects and responses of HS in bovine cumulus cells, yet comprehensive data remains unclear.

Consequently, this study aims to establish immortalized cumulus cell line from Hanwoo (a Korean local breed) for HS *in vitro* model and to explore the HS-related molecular response in the cumulus cells by integrating transcriptomics and proteomics approaches.

## MATERIALS AND METHODS

**Figure 1:**
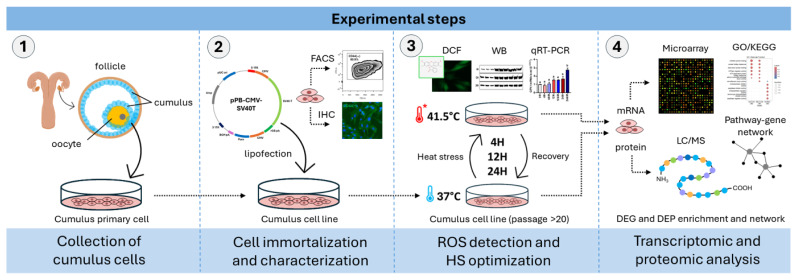
Schematic figure of experimental steps in this study. FACS, florescence-associated cell sorting; IHC, immunohistochemistry; DCF, dichlorofluorescein; WB, Western blotting; qRT-PCR, quantitative reverse-transcription polymerase chain reaction; ROS, reactive oxygen species; HS, heat stress; GO, gene ontology; KEGG, Kyoto Encyclopedia of Genes and Genomes; LC/MS, liquid chromatography–mass spectrometry; DEG, differentially expressed genes; DEP, differentially expressed proteins.

The schematic figure of experimental steps is provided in Figure 1.

### Primary cells, cell line generation and characterization

Ovaries from female Hanwoo cattle were collected at a local slaughterhouse and transported on ice. Cumulus–oocyte complexes were aspirated from large follicles and cumulus cells were isolated after 22 h *in vitro* maturation in TCM-199 (Gibco), treated with hyaluronidase (Sigma-Aldrich) and 0.1% trypsin-EDTA (Thermo Fisher Scientific), then cultured in TCM-199/Ham’s F-10 (Gibco) with 15% FBS (HyClone). Primary cumulus cells were immortalized by transfecting SV40 large T antigen (SV40T) expression vector containing *piggyBac* elements and transposase (VectorBuilder) using Lipofectamine 3000 (Invitrogen) per manufacturer’s instructions (Figure 1). Stable transgenic cells were selected with 4 μg/mL puromycin, and SV40T insertion was verified by polymerase chain reaction (PCR). Cells were maintained in DMEM/high glucose with 10% FBS and 1% antibiotic–antimycotic solution. The immortalized cells had doubling time about 21 hours (Supplement 1) and were passaged every 3 to 4 days when reaching 80% to 90% confluency after seeding at 0.5 to 1×10^3^ cells/cm^2^ density.

### Immunohistochemistry

In a 48-well plate, 10,000 cells/well were seeded one day before immunohistochemistry (IHC) treatment to allow cell attachment. After Dulbecco’s phosphate-buffered saline (DPBS) washing, the cells were fixed with 4% paraformaldehyde for 20 min at room temperature (RT), washed with Tris buffered saline with Tween 20 (TBST) 3×5 min, and applied with blocking solution (5% donkey serum in TBST) for 30 min at RT. Without prior washing, primary antibodies were applied for 5 h at 4°C. After TBST washing 3×5 min, secondary antibodies were applied for 60 min at RT in dark condition. Following TBST washing 3×5 min, the cells were applied with 4’,6-diamidino-2-phenylindole (DAPI, 1×) 10 min at RT in dark, washed with DPBS 2 to 3 times, and prepared for IHC imaging.

### Florescence-associated cell sorting

Cells were harvested using 0.5% trypsin-EDTA (TE), centrifuged 700×g for 3 min to get cell pellet and completely resuspended in 1 mL of 1% bovine serum albumin in DPBS. Primary antibody was applied for 30 min on ice with agitation every 5 mins. After DPBS wash, secondary fluorescein isothiocyanate conjugated antibody was applied for 30 mins on ice with agitation every 5 mins. After DPBS wash, the cells were prepared for florescence-associated cell sorting (FACS) sorting using BD FACSAria (BD Biosciences) following the manufacturer’s protocols.

### Heat stress condition and DNA/RNA/protein extraction

Cells were grown in 3×60 mm plate until 60% to 80% confluency for each treatment: control, HS, and recovery group. HS at 41.5°C followed by recovery at 37°C were applied for 4 h, 12 h, and 24 h in 5% CO_2_ incubator at ~95% relative humidity (RH). After cell harvesting, total genomic DNA, total RNA, and protein were extracted by using DNeasy Blood & Tissue Kit (Qiagen), RNeasy Plus Mini Kit (Qiagen), RIPA buffer (Thermo Fisher Scientific), respectively, following the manufacturer protocols. Protease inhibitor and phosphatase inhibitor were added prior extraction. DNA and RNA concentration and quality was evaluated by using nanodrop while protein lysates concentration was determined by using Pierce BCA Protein Assay Kits (Thermo Fisher Scientific) following manufacturer’s procedure.

### Total reactive oxygen species detection

Cells were seeded at ~10,000 cells/well density in 48-well plate assigned into control and HS group. Next day, the HS-group was treated at 41.5°C for 4 h in CO_2_ incubator. Total intracellular ROS level was measured using 10 μM chloromethyl-2′,7′-dichlorodihydrofluorescein diacetate (CM-H2-DCFDA) (Invitrogen) in DPBS followed by 37°C incubation for 5 min without washing prior fluorescence imaging.

### Quantitative reverse-transcription polymerase chain reaction

Complementary DNA (cDNA) was generated from 1 μg total RNA using the Superscript III First-Strand Synthesis System (Invitrogen) following the manufacturer’s protocol. qRT-PCR was performed in 25 μL volume reaction including 2 μL cDNA, 2.5 μL PCR buffer, 1 μL dNTP mixture (2.5 mM), 1 μL SYBR, 1 U Taq DNA polymerase, and 10 pmol forward and reverse primer (Supplement 2). The quantitative (q)RT-PCR were programmed at 95°C for 3 mins followed by 40 cycles of 95°C for 3 min, 60°C for 30 s, 72°C for 30 s by using CFX Duet Real-Time PCR System (Bio-Rad). Melting curve profiles were generated for amplicon specificity evaluation. Data were normalized with GAPDH expression level and calculated under the 2^−ΔΔCt^ method. Three antioxidant genes observed were superoxide dismutase 1 (*SOD1*), *CAT*, and glutathione peroxidase 1 (*GPX1*) genes. Primers lists are in Supplement 2.

### Western blot

Protein lysates were normalized with DPBS, mixed with 1× Laemmli buffer, and heated at 95°C for 5 min. After centrifugation (20,000 ×g, 1 min), samples were separated on 10% sodium dodecyl sulfate–polyacrylamide gel electrophoresis (SDS–PAGE) (50 V for 5 min, then 100 V for 90 min) and transferred to a 0.45 μm PVDF membrane using a Mini Trans-Blot Cell (Bio-Rad; 100 V, 3 h). Membranes were blocked and incubated overnight at 4°C with primary antibodies against HSP70 (PTGLab 10995-1-AP), BiP/GRP78 (PTGLab 11587-1-AP), and ACTB (Santa Cruz sc-47778). After washing (5×5 min, TBST), HRP-conjugated secondary antibody was applied for 1 h at RT, and signals were visualized with luminol–hydrogen peroxide substrate.

### Microarray

Total RNA quality control was conducted with Agilent Bioanalyzer with RNA integrity number (RIN) ranging at 8.4 to 9.3 and A260/280≥2.0 prepared for GeneChip Cow Gene 1.0 ST Array. The cDNA was synthesized using GeneChip WT Amplification kit as described by the manufacturer. Sense cDNA was fragmented and biotin-labeled with terminal deoxynucleotidyl transferase using the GeneChip WT Terminal labeling kit. Approximately 5.5 μg of labeled DNA target was hybridized to Affymetrix GeneChip Array at 45°C for 16 h. Hybridized arrays were washed and stained on a GeneChip Fluidics Station 450 and scanned on a GCS3000 Scanner (Affymetrix). The probe cell intensity data computation and CEL files generation were performed using Affymetrix GeneChip Command Console Software (AGCC). The results of normalized fold change genes are available in Supplement 3.

### Proteomic liquid chromatography–mass spectrometry

Proteins (100 μg) were digested with S-Trap mini columns (Protifi), labeled with TMT10 reagents (Thermo Fisher Scientific), and fractionated by reverse-phase liquid chromatography (LC) (Agilent). Peptides were analyzed on a Q Exactive HF-X mass spectrometer coupled to an Ultimate 3000 RSLCnano system. Mass spectrometry (MS) scans (m/z 400–2,000, 60,000 resolution) were acquired in top-10 DDA mode, with HCD fragmentation (NCE 32) and MS/MS at 45,000 resolutions. Data were processed in Proteome Discoverer 3.0 (Thermo Fisher Scientific) using CHIMERYS against UniProt with ≥2 unique peptides at 1% FDR. Normalized fold-change results are shown in Supplement 4.

### Data analysis

Quantification of protein band intensity was done using ImageJ. Quantitative data were analyzed and visualized using Prism and R. Statistical mean differences (p*<*0.05) were analyzed using One-way ANOVA with automatic compact letter display using “multicompView” R 4.5.2 package. Transcriptomics and proteomics gene ontology (GO) enrichment was done by using “clusterProfiler” R package with p<0.05 and q<0.2. Cluster pathway (Kyoto Encyclopedia of Genes and Genomes [KEGG] and Reactome) enrichment was performed by multiple batch analysis in Metascape (https://metascape.org/) [18] with p-value<0.01, minimum count of 3, and enrichment factor>1.5. Visualization was generated by using R “ComplexHeatmap” for heat map and “igraph” for pathway-gene network. Human database was used as reference for all enrichment analysis.

## RESULTS

### Establishment of Hanwoo cumulus cell line

**Figure 2:**
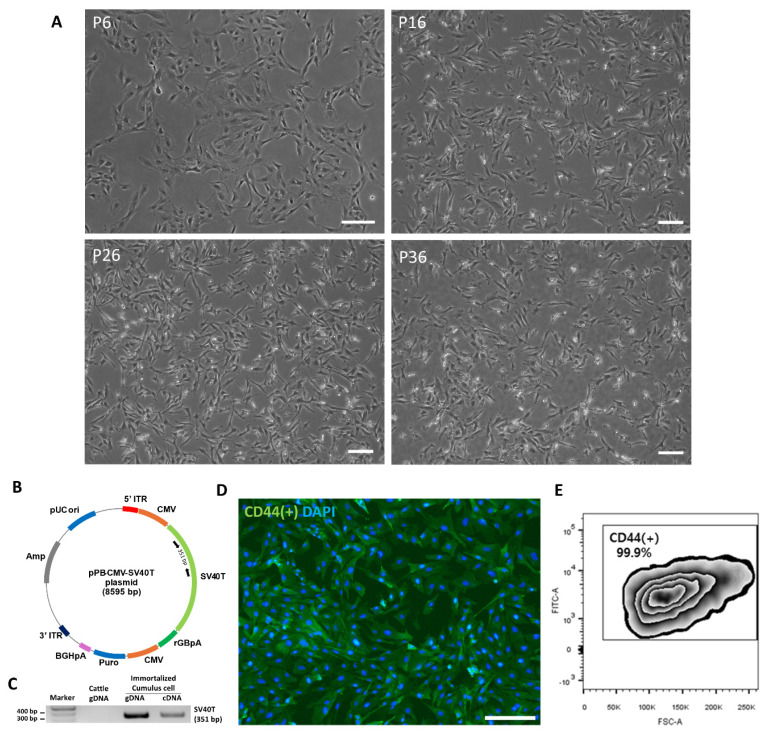
Cumulus cell line establishment for heat stress model. (A) Primary cumulus cells at passage 6 (P6) and immortalized cumulus cell line at different passages (P16, P26, and P36). (B) The vector map of piggy-bac SV40T plasmid for cumulus cell immortalization with flanking arrows showing primer pair location for SV40T PCR validation (351 bp amplicon). (C) PCR confirmation of SV40T integration in the cumulus genomic DNA (gDNA) and complementary DNA (cDNA) compared to intact Hanwoo cattle gDNA. (D) Immunohistochemistry of cumulus cells treated with anti-CD44 antibody (green) and DAPI (blue). (E) Contour plot of CD44(+) cumulus cells isolated by using FACS sorting. Scale bar = 200 μm. PCR, polymerase chain reaction; CD44, cluster of differentiation 44; FACS, florescence-associated cell sorting.

To evaluate HS response in Hanwoo cumulus cells, we established cumulus cell line immortalized by transfecting the SV40T antigen *piggyBac* expression vector (Figures 2A, 2B). The integration of the SV40T gene was successfully generated in which specific 351 bp PCR-amplicon segment of the SV40T genes was observed in the immortalized cumulus cell both in genomic DNA and mRNA expression (cDNA) sample compared to intact Hanwoo cattle genomic DNA (Figures 2B, 2C, uncropped electrophoresis gel images are in Supplement 5). Cluster of differentiation 44 (CD44) protein is a specific hyaluronic acid receptor in cumulus cells that interact with follicular glycosaminoglycan playing important roles in supporting oogenesis and embryogenesis in mammals [19]. The IHC images by using anti-CD44 specific antibody, presented major fluorescence signal (Figure 2D) in the immortalized cumulus cells with FACS sorting signal CD44-positivity score at 99.9% (Figure 2E). This prominent specific biomarker signal confirmed the homogeneity of established cumulus cell line isolated from Hanwoo cattle. As far as this experiment was conducted, the cumulus cell line was still actively proliferating for more than 50 passages.

### Oxidative stress validation

**Figure 3:**
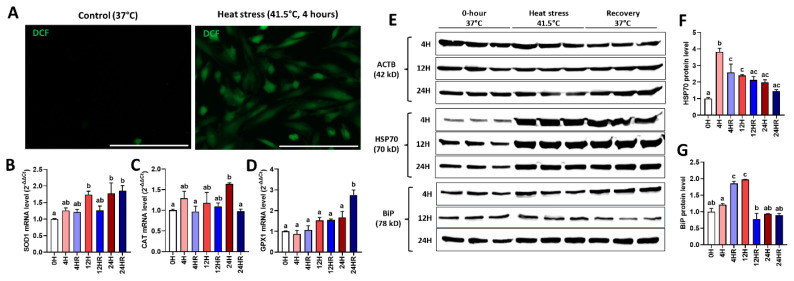
Cumulus cell optimization of heat stress condition. (A) ROS detection by DCF fluorescence after incubation at 41.5°C for 4 h. (B–D) 2^−ΔΔCt^ value of mRNA relative expression from three oxidative stress marker genes (SOD1, CAT, and GPX1) in three periods of heat stress and recovery time (4 h, 12 h, and 24 h) by using qRT-PCR. (E) Protein expression of heat stress molecular markers by using HRP-Ab anti-HSP70, anti-BiP, and anti-ACTB in 10% SDS-PAGE under three periods of heat stress and recovery time (4 h, 12 h, and 24 h). (F, G) Normalized relative quantification of the HSP70 and BiP protein expression based on the SDS-PAGE protein band figures. Labelling: −H = hours of heat stress period; −HR = hours of recovery period, DCF = dichlorodihydrofluorescein. Bar height represents mean value±SEM and the differences between means were analyzed using one-way ANOVA followed by Tukey’s Multiple Comparisons Test in biologically independent samples (n = 3). ^a–c^ Different compact letters represent significant difference with p≤0.05. ROS, reactive oxygen species; SOD1, superoxide dismutase 1; CAT, catalase; GPX1, glutathione peroxidase 1; qRT-PCR, quantitative reverse-transcription polymerase chain reaction; HSP, heat shock protein; SEM, standard error of the mean.

Elevated ROS level, represented by dichlorofluorescein (DCF) fluorescence signal, in cumulus cells under HS has been confirmed at 41.5°C for 4 h HS period compared to the control (Figure 3A). However, the mRNA expressions of the three oxidative stress response markers (*SOD1*, *CAT*, *GPX1*) did not exhibit significant increase (p<0.05) during 4 h HS and subsequent 4 h recovery period (Figures 3B–3D). Significant upregulation was observed at 12-h HS in *SOD1* expression at 1.74±0.08-fold increase relative to control (mean±standard error of the mean [SEM], p<0.05) followed by downregulation at 12 h recovery period (Figure 2B). On the other hand, no apparent increase was detected for *CAT* and *GPX1* mRNA level at 12 h HS and recovery (Figures 3C, 3D). Under 24 h HS treatment, *SOD1* and *CAT* presented significant relative increase at 1.77±0.25-fold and 1.64±0.02-fold, respectively, but not *GPX1*. The upregulation remains significant during 24 h recovery for *SOD1* (1.64±0.02) while *CAT* returned to base level relative to control group. Interestingly, *GPX1* mRNA expression was significantly elevated later during 24 h post-HS period by up to 2.75±0.19-fold increase (Figure 3D). In summary, this dynamic changes in mRNA expression of oxidative stress might represent complex and prolonged response of the immortalized cumulus cells to maintain their oxidative cellular balance under HS condition.

### Endoplasmic reticulum stress validation

Substantial increase of HSP70 protein expression was detected in cumulus cells under 4 h HS period reaching significant 3.83±0.17-fold increase (p<0.05) compared to the control level (Figures 3E, 3F, Supplement 6). This upregulation reduced during 4 h recovery but it was still significantly higher than control with 2.57±0.42-fold difference (Figure 3F). The HSP70 protein remains significantly expressed at 12 h HS condition with 2.39±0.05-fold increase while it declined to normal level at 12 h recovery period. Conversely, the ER-specific chaperone, BiP, showed no significant protein expression level alteration during 4 h HS, yet it significantly stimulated afterwards during 4 h recovery treatment approaching 1.86±0.04-fold relative increase (Figures 3E, 3G). Under 12 h HS, BiP level also peaked at 1.97±0.01-fold change of significant protein expression and returned to control base level during 12 h recovery (Figure 3G). Interestingly, no significant protein level changes were observed in both HSP70 and BiP during 24 h HS and recovery treatments. These ER stress protein upregulated expressions exhibited immediate response of the cumulus cells to misfolded proteins caused by hyperthermic conditions. Overall, together with the oxidative stress gene markers expression results described above, we determined 12 h as optimal HS and recovery treatment period for the cumulus cell line and applied this treatment to generate transcriptomics and proteomics data.

### Differentially expressed genes and proteins

**Figure 4:**
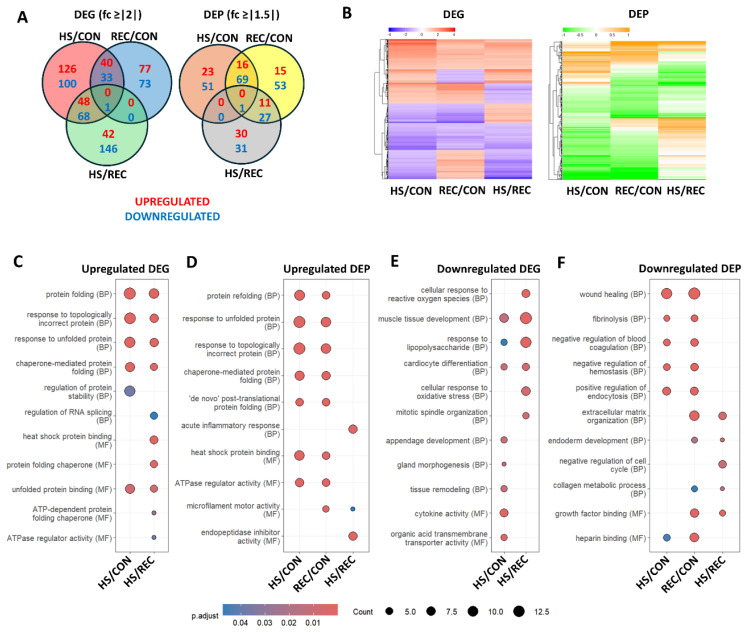
DEG-DEP Venn diagram, heat map expression, and functional enrichment analysis of cumulus cells under 12 h of heat stress and 12 h recovery period. (A) Venn diagram of the numbers of overlapping upregulated and downregulated DEG (fc≥|2|), DEP (fc≥|1.5|). (B) Heat map of DEG showing fold-change and DEP showing log2 value clustered by Euclidean distance method showing fold-change score representing relative genes expression among three ratio groups. For heatmap clarity, DEGs and DEPs with fold-change≥|4| and ≥|1| are colored by maximum color, respectively. (C–F) ClusterPlot of top GOs with significant enrichment (p≤0.05, q≤0.2) of biological process (BP) and molecular functions (MF) based on gene sets from upregulated DEG (C), upregulated DEP (D), downregulated DEG (E), and downregulated DEP (F). Non-significant enriched cluster labels are not shown in the diagram. DEG, differentially expressed genes; fc, normalized fold-change; DEP, differentially expressed proteins; HS/CON, heat stress vs control; REC/CON, recovery vs control; HS/REC, heat stress vs recovery; GO, Gene Ontology.

Under 12 h period of HS and recovery treatments, there were in total 754 DEGs (fold-change threshold log_2_≥|2|) with 333 genes (44.2%) were upregulated and 421 genes (55.8%) were downregulated (Figure 4A). Under HS condition, HS/CON-only group exhibited 226 DEGs (31.0%) in with 126 and 100 DEGs upregulated and downregulated, respectively. During recovery (REC/CON), there were in total 224 DEGs (29.7%) with 117 upregulated DEGs and 107 downregulated DEGs. From these, 73 DEGs (10.0 %) were altered in both HS and recovery group. In proteomics data, limited enrichment terms were observed at 2-fold change threshold (Supplement 7); therefore, lower threshold value at 1.5-fold-change was applied for more robust functional enrichment analysis. Overall, there were a smaller number of DEPs (fold-change threshold 1.5) compared to the DEGs (Figure 4A). Among the total 327 DEPs, 95 DEPs (29.1%) were upregulated while 232 DEPs (70.9%) were downregulated. From 160 DEPs in HS group (HS/CON), 74 DEPs (22.6%) altered in HS/CON-only group with 16 DEPs upregulated and 69 DEPs downregulated in both HS and recovery group. In recovery (REC/CON)-only group, there were 15 DEPs upregulated and 53 DEPs downregulated. Additionally, twelve genes were concordantly expressed in both DEG and DEP including heat shock proteins, epiregulin (EREG), and store-operated calcium entry-associated regulatory factor (SARAF) (Supplement 8).

Fold change heat maps of DEG and DEP showed alternative view of relative gene expression dynamics among ratio groups (Figure 4B). Gene clusters of HS/CON that stayed upregulated or downregulated during REC/CON (showing the same color in both ratio) represent pro-longed response of DEGs or DEPs during recovery. There were about two-thirds of DEGs and three-fourths of DEPs displayed pro-longed upregulation or downregulation in both HS and recovery. Furthermore, the HS/REC color column represents the gene behavior changes during recovery in which red color displayed higher expression at HS compared to recovery, and vice versa. About one-fourth of the DEGs were either increased or more decreased during recovery (genes with different color of HS/REC compared to HS/CON and REC/CON), and of those about a half portion displayed in DEPs heat map. The cluster portion of each ratio groups the heat map corresponds to numbers of overlapping DEG and DEP in Figure 4A.

### Gene Ontology enrichment on biological process and molecular function terms

To understand comprehensive biological meanings of the DEGs and DEPs lists of this study, we performed GO enrichment analysis on biological process (BP) and molecular function (MF) terms. Both upregulated DEGs and DEPs significant top GO enrichment terms (p-value≤0.05) were mostly related to protein folding and HS-response related terms (Figures 4C–4F). In upregulated DEGs enrichment, many of these terms were also significantly enriched in HS/REC group (Figure 4C) which might represent returning mechanism at mRNA expression level related to HS-response functions during recovery period. Interestingly, on the other hand, all MF terms related to HS-response in upregulated DEPs (HS/CON) were also exhibited in recovery groups (REC/CON; Figure 4D) which may indicate pro-longed HS-response at protein level in the cumulus cells during 12 h recovery period. In upregulated DEPs HS/REC group, other significantly enriched BP terms related to acute inflammation response and MF terms related to endopeptidase inhibitor activity were also detected (Figure 4D).

Downregulated DEGs and DEPs exhibited top significant enrichment to various GOs, particularly terms related to ROS response, tissue development, immune response, ECM structures, wound healing, and transmembrane transporter activity (Figures 4E, 4F). In line with oxidative stress validation described above (Figures 3B–3D), cellular response to ROS and oxidative stress terms were enriched in HS/REC group representing delayed response during recovery (Figure 4E). Immune related terms including response to lipopolysaccharide (BP term) and cytokine activity (MF term) were enriched in HS/CON group. Additionally, ECM-related terms were presented in recovery GOs (REC/CON; Figure 4F) showing potential delayed effect on cumulus ECM post-HS condition. Developmental BP terms i.e., muscle tissue development, cardiocyte differentiation, and gland morphogenesis, and MF terms of transmembrane transporter activity terms were enriched in HS/CON group of downregulated DEGs (Figure 4E). Complete list of GO enrichment results and corresponding gene list are available in Supplements 9–12.

### Pathway enrichment and pathway-gene network

**Figure 5:**
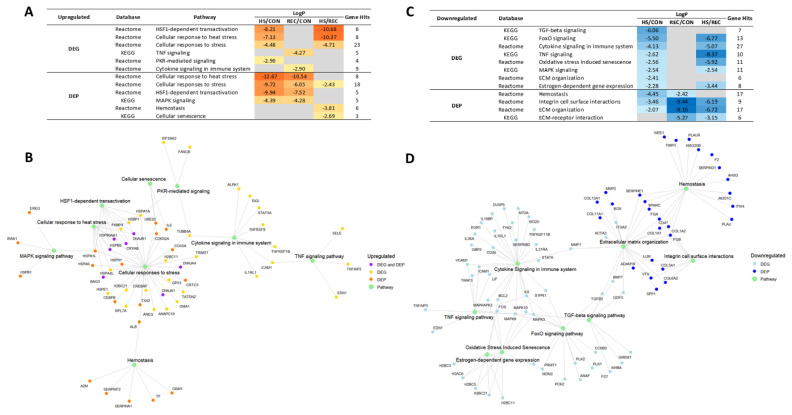
KEGG and Reactome pathway enrichment and pathway-gene network under HS. (A, B) Significantly enriched pathways of upregulated DEG and DEP with LogP enrichment scores from each group (A) and their pathway-genes networks (B). (C, D) Significantly enriched pathways of downregulated DEG and DEP with LogP enrichment score from each group (C) and their pathway-genes networks (D). All dots are labelled with the pathway or gene symbol. The KEGG-Reactome pathway enrichment are conducted with batch enrichment analysis by Metascape under p<0.01, minimum count = 3, and enrichment factor>1.5. HS/CON, heat stress vs control; REC/CON, recovery vs control; HS/REC, heat stress vs recovery; DEG, differentially expressed genes; DEP, differentially expressed proteins; KEGG, Kyoto Encyclopedia of Genes and Genomes.

To complement GO enrichment results, pathway enrichment analysis was performed according to KEGG and Reactome gene sets databases. Supporting GO results, upregulated DEG-DEP gene list highlighted significant pathway enrichments (logP<2, minimum count = 3, enrichment factor>1.5) to HS-related responses including HSF-1 dependent transactivation and cellular response to HS or stress in both upregulated DEGs and DEPs (Figure 5A). The HS-related pathway enrichment also exhibited the same recovery pattern as GO enrichment in upregulated DEGs at HS/CON-HS/REC cluster and pro-longed response of HS in upregulated DEPs during recovery (REC/CON; Figure 5A). Immune and inflammatory related pathways i.e., TNF signaling and cytokine signaling in immune system, from upregulated DEGs were presented during recovery (REC/CON). Other stress related pathways such as PKR signaling (upregulated DEGs) were enriched during HS and MAPK signaling (upregulated DEPs) was enriched in HS and recovery groups. Hemostasis and cellular senescence pathways were also enriched in HS/REC upregulated DEPs.

To remark prominent genes in cumulus HS response, pathway-genes network of the significantly enriched pathway was generated. Upregulated DEG-DEP pathway-genes network showed 55 DEGs and 45 DEPs interconnected in highlighted pathways related to cellular stress, HS, and immune response (Figure 5B). The upregulated DEGs involved in these pathways are centralized to chaperone or heat shock proteins (HSPs) and co-chaperones molecules such as BAG3 and DNAJ family (Figure 5B). Seven DEG-DEPs significantly enriched to HS response GOs were also observed in response to HS pathway i.e., *HSP90AA1, HSPB8, DNAJB1, DNAJA1, DNAJA4, CRYAB*, and *BAG3* (Supplement 8). Additionally, a major extracellular antioxidant gene, *glutathione peroxidase 3* (*GPX3*), was also highlighted in the cellular response to stress pathway (Figure 5B). EREG that was concordantly expressed at mRNA and protein level (Supplement 8) was found involved in MAPK signaling pathway. Complete list of pathway enrichment and corresponding gene list are available in Supplements 13–16.

Among enriched pathways of downregulated DEGs and DEPs, highlighted pathways were found to be related to ECM, immune system, stress or oxidative stress response, estrogen activity, and hemostasis (Figure 5C). ECM organization pathway enrichment exhibited in both downregulated DEGs and DEPs. At protein level, this downregulation of ECM organization pathway prolonged during recovery periods, including ECM interaction pathways such as integrin surface and ECM-receptor interaction (Figure 5C).

Many downregulated DEG-DEP expressions in the ECM organization were related to collagen, including COL1A1 that interconnected hemostasis and integrin cell surface interaction pathways, and other molecules inter-related to cellular signaling such as vitronectin (VTN) and *TGFB2* (part of TGF-β signaling pathway; Figure 5D). All genes in hemostasis pathway were downregulated proteins that enriched in both HS and recovery periods and many of them interconnected with ECM pathways. Furthermore, pathways and genes related to inflammatory response and cell survival were also identified during HS such as cytokine signaling (including Interleukin-6, *IL6*), FoxO signaling, TNF signaling, MAPK signaling, and oxidative stress induced senescence (Figures 5C, 5D).

## DISCUSSION

Cumulus cells are indispensable specialized cells that surround mammalian oocyte in the COC structure providing suitable microenvironment and nutritious supplements for the maturing oocyte [7,8]. To our knowledge, this study established the first specific Hanwoo cumulus cell line (99.9% of CD44[+] cells) that can be utilized to study HS response in cattle. While research on cumulus cells by using primary cells were reported [20,21], there are limitations of using primary cells such as batch variability, limited life span, and reproducibility. This study generates cumulus cell line model that can be potentially utilized for various long-term *in vitro* study related to cumulus cells biology.

Hyperthermal stress (starting from 40°C) has been well-known generating over accumulated ROS in the cells that disturb cellular oxidative balance leading to DNA damage and apoptosis [1,2,22,23]. Recent study found that 41.5°C hyperthermia for 4 h in the cumulus cell line has caused intracellular ROS accumulation as previously reported [13,24]. The cells respond to this physiological oxidative imbalance by upregulating mRNA expressions of antioxidant genes i.e., *SOD1, CAT, GPX1* starting at 12 h HS period with approximately 1.5 to 2.5-fold-change increase. In line with this result, Khan et al [24] also found higher *SOD1* mRNA and protein level at about 2-fold and 1.5 fold increase, respectively, compared to control under 40°C HS for 24 h in bovine granulosa cells. Additionally, unaltered or recovering mRNA level of *SOD1, CAT*, and *GPX1* at short period of HS in this study (41.5°C for 4 h)have also been found previously in *in vitro* research of primary bovine oocyte-cumulus-granulosa cell complexes under 40.5°C HS for 5 h [25]. The SOD1 or CuZn-SOD functions as a key cytoplasmic enzyme from SODs family that convert primary ROS such as superoxide anion (O_2_^•−^) into hydrogen peroxide (H_2_O_2_) [26,27]. Subsequently, CAT and GPX1 enzymes convert these intracellular H_2_O_2_ into water through direct and glutathione-coupled reaction, respectively, maintaining cellular redox homeostasis [28,29]. Alteration of the cumulus antioxidant response under HS is crucial because biochemical process in cumulus cells determine redox status of oocyte in COC [8]. Therefore, upregulation of antioxidant genes in cumulus cells under HS is indeed part of the cellular response to alleviate ROS level in preventing cellular damage with noting that individual genes might show unique dynamic of expression level during and post-HS condition [13,25].

Beside oxidative stress, HS causes protein misfolding and aggregation that disturb cellular physiological balance [30]. Cumulus cells response this by upregulating HSP mechanism to repair the unfolded proteins in cytoplasm and ER preventing cellular apoptosis [12,14,31]. Immortalized cumulus cells in this study respond to 41.5°C HS treatment by increasing protein expression of the HSP70 (~4-fold increase) and BiP protein (~2-fold increase) at 4 h and 12 h HS period, respectively. This is coherent with previous study that observed increased mRNA and protein level of both HSP70 and BiP in bovine cumulus under HS [12]. Previous study also show comparable results that bovine granulosa cells increase *HSP70* mRNA and protein level up to 4-fold change under 41°C HS for 2 h relative to control [22]. HSP70 is a multifunctional chaperone known for its immediate upregulation during HS that play roles in assisting protein folding and preventing misfolded protein aggregation [14]. *In vitro* supplementation of HSP70 to COC has also been known restoring developmental competence of oocyte and improve embryo quality [15]. Additionally, the high level of stress-inducible BiP protein suggests an activation of ER-stress mechanism [32]. When HS trigger excessive misfolded protein inside ER lumen, BiP protein expression is upregulated as part of unfolded protein response (UPR) preventing aggregation and promoting correct folding by utilizing ATP [32,33]. Consequently, BiP upregulation protects reproductive cells from toxic ROS effect, maintaining ER and cell functioning, and promoting support to preimplanted embryo [32]. Overall, these findings suggest that upregulation of major chaperone HSP70 and ER-stress marker BiP are protective response in cumulus cells to maintain protein homeostasis and supportive functions for oocyte and early embryo development as previously reported.

Supporting findings above, the cumulus GOs and pathway enrichment of upregulated DEG-DEP under HS of this study also displayed coherent functional terms to protein folding, UPR, and cellular response to HS. HSF1, the master pathway for HSP activation [14], is enriched in both upregulated DEG and DEP showing concordant HSP activation in cumulus cells under HS (Supplement 8). Consistent with our findings, other transcriptomic study of primary granulosa cells under HS exhibited enriched terms related to ER-protein processing and protein folding including upregulation of many HSPs proteins particularly HSP90AA1 [17,22]. These concordant upregulated HS-related GOs terms suggest optimal HS conditions are relatively well-established in this study. Additionally, prolonged *in vitro* HS responses during recovery observed in this study are expected results because *in vivo* and *in vitro* studies have detected carry-over detrimental effect in COC functionality, embryo, and cattle progeny post-HS [4,34]. Therefore, pro-longed HS-response may suggest extended protective HS responses of cumulus cells especially at protein level (Figure 5A) to sustain oocyte development. Further study is needed to elucidate this specific cellular recovery response after HS.

Heat-induced stress causes harmful impacts on COC viability compromising oocyte nuclear maturation and capacitation [11,12,22]. This study revealed that alleviated temperature trigger downregulation of DEG and DEP related to ECM, development, and signaling process in cumulus cells suggesting potential effects on COC functionality. In previous study, HS condition has found altering ECM related processes such as collagen formation, cell junction, and cell adhesion in cumulus, oocyte, and granulosa cells [16,17]. Downregulated ECM function, especially in the COC gap junctions is of concern because it plays major roles in transferring small nutritional and regulatory molecules for oocyte growth and development [9]. Impaired gap junctional COC communication under HS increase progesterone level leading to early oocyte meiotic maturation [35]. Furthermore, downregulation of integrin function and protein such as VTN (Figure 5D) might disturb cumulus cell adhesion, integrins (αvβ3) signaling, and sperm capacitation [36]. Interestingly, SARAF and EREG, two proteins that were concordantly upregulated under HS at DEG and DEP (Supplement 8), are key proteins that regulate Ca^2+^ influx in COC, an important process for oocyte maturation and acrosomal reaction during fertilization [10]. Moreover, ER-chaperones including BiP protein is also activated in calcium-dependent manner [37]. While upregulation of these proteins suggest compensational response to balance intracellular Ca^2+^-signaling under HS, this also suggest possible detrimental effect on follicular cycle such as accelerated or delayed oocyte maturation due to EREG or SARAF upregulation, respectively [7,10]. Extended upregulation of EREG during recovery may suggest possible persistent disturbance on maturing oocyte. Collectively, these results indicate that HS may affect cumulus ECM functions involved in supporting oocyte maturation and fertilization.

We are aware that careful interpretation should be conducted when using SV40T immortalized cumulus cells in this study. For example, HSP overexpression during HS often related to tumorigenesis and suppression of p53 functions [38]. However, since SV40 large T-antigen binds directly to p53 transcription factor inhibiting its function [39], the immortalized cumulus cell cannot be utilized to confirm this alteration. Despite this, our cumulus cell line shows common HS-related response aligned with previous findings. Accordingly, immortalized cumulus cells in this study can still be reasonably and optimally applied for further study regarding HS cellular mechanism.

In summary, this study presents prior Hanwoo cumulus cell line as a robust *in vitro* model to evaluate reproductive Hanwoo cell response under HS. As previously reported, HS increases ROS accumulation in cumulus cells and activates cellular HSP and ER-stress response as early as 4 h HS (HSP70 and BiP upregulation). This response peaked at 12 h HS period as followed by the increase of antioxidant genes expression (*SOD1, CAT, GPX1*). Furthermore, this study also highlights GO terms and pathways altered under HS, such as upregulation of protective response of protein folding repair mechanism and potential negative effects downregulated ECM functions, particularly on the gap junction and integrin impairment, which might interfere with cumulus expansion and communication compromising oocyte competence [9,35]. The immortalized cumulus cells also demonstrate prolonged HS response during recovery period as widely known that HS has delayed effect in cattle reproduction. Besides that, this study also provides valuable omics data, particularly on HS-related cumulus proteomics which are currently very limited. The findings described herein can enhance fundamental knowledge and provide data to elaborate molecular targets for future study to mitigate HS negative effect in cattle reproductive functions. Finally, the establishment of immortalized cumulus cells provides a promising *in vitro* model to investigate the effects of HS on bovine reproduction that offers higher reproducibility within a simplified system.

## CONCLUSION

Hyperthermic conditions compromise bovine reproduction by alleviating cellular ROS accumulation and impairing normal protein folding in cumulus cells which disturb its functionality in supporting oocyte maturation and fertilization. Cumulus cells response to the HS conditions by activating antioxidant genes and protein repair mechanism of both cytoplasmic and ER-specific HSPs to prevent cellular damage and maintain normal cumulus functions in COC. However, HS downregulates ECM functions in cumulus cells including gap junctions and integrin activity which might results in disturbing cumulus-oocyte communication and signaling, compromising oocyte development and capacitation. As a result, HS reduces overall cattle reproductive capacity and potentially causes substantial loss in cattle livestock production.

Utilization of immortalized cumulus cells in this study confirms and advances understanding of HS response in cattle cumulus cells. Not only for this research, transcriptomics and proteomics data from the HS-treated cumulus cells can also be adopted for future research such as mitigating the negative effect of HS in cumulus function by applying molecular targeting approach.

## Data Availability

Upon reasonable request, the datasets of this study can be available from the corresponding author.

## SUPPLEMENTARY MATERIAL

**Supplement 1**. Proliferation data of the immortalized cumulus cells at passage 25.

**Supplement 2**. List of primers used in this study.

**Supplement 3**. Microarray cumulus cells normalized raw data.

**Supplement 4**. Proteomics cumulus cells normalized raw data.

**Supplement 5**. Uncropped electrophoresis image of SV40-T insertion genes.

**Supplement 6**. Uncropped Western Blot imaging of HSP70 and BiP.

**Supplement 7**. STRING and enrichment analysis of DEP at 2-fold change.

**Supplement 8**. Venn diagram of concordant DEG-DEP and STRING analysis of upregulated DEG-DEP.

**Supplement 9**. Cumulus upregulated DEG - GO enrichment (BP, MF).

**Supplement 10**. Cumulus downregulated DEG - GO enrichment (BP, MF).

**Supplement 11**. Cumulus upregulated DEP - GO enrichment (BP, MF).

**Supplement 12**. Cumulus downregulated DEP - GO enrichment (BP, MF).

**Supplement 13**. Cumulus upregulated DEG pathway enrichment (KEGG and Reactome).

**Supplement 14**. Cumulus downregulated DEG pathway enrichment (KEGG and Reactome).

**Supplement 15**. Cumulus upregulated DEP pathway enrichment (KEGG and Reactome).

**Supplement 16**. Cumulus downregulated DEP pathway enrichment (KEGG and Reactome).
