# Supplementary figures and images for: Establishment and characterization of Hanwoo cumulus cell line for heat stress studies
Source: Anim Biosci. 2026 Jun 15;39(7):250896. doi: 10.5713/ab.250896 (PMC13353149; doi:10.5713/ab.250896)

**Supplement 1.** Proliferation data of the immortalized cumulus cells at passage 25.

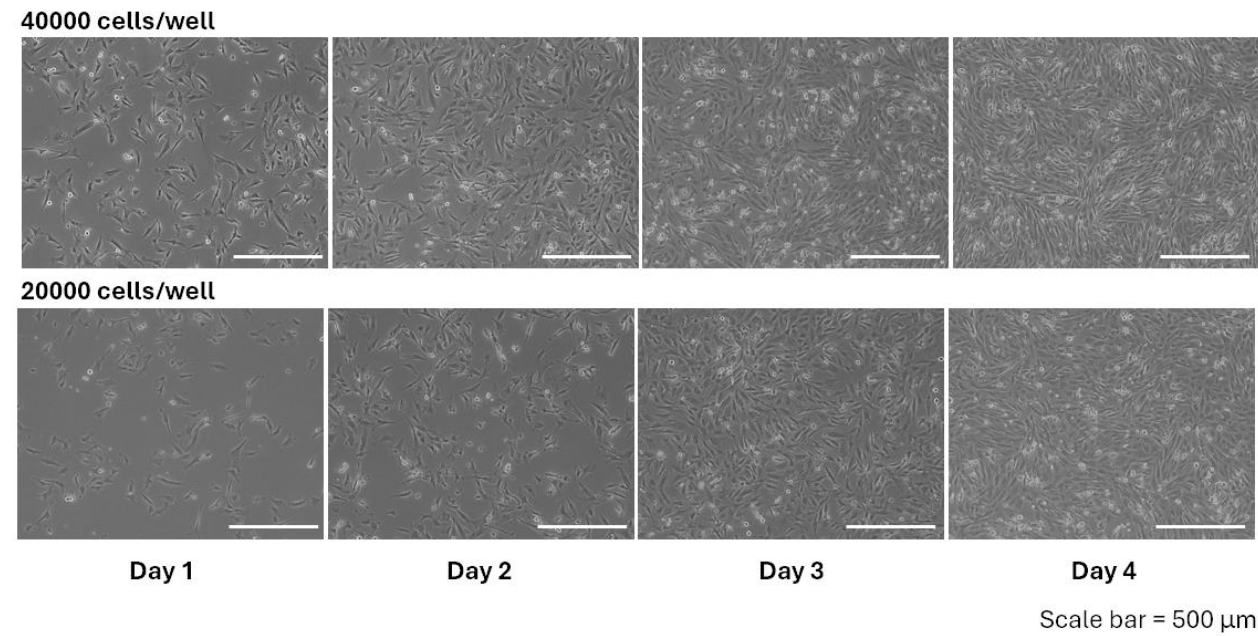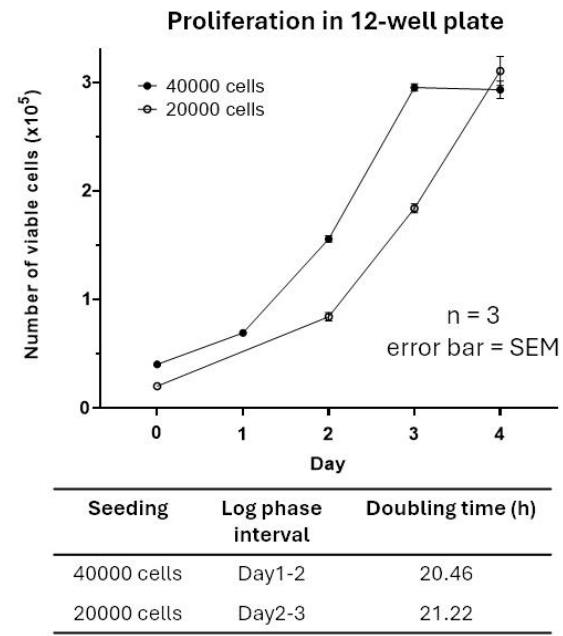

Supplement: Supplementary file 1 [file ab-250896-Supplementary-1.pdf]

Supplement 6. Uncropped Western Blot Imaging of HSP70 and BiP

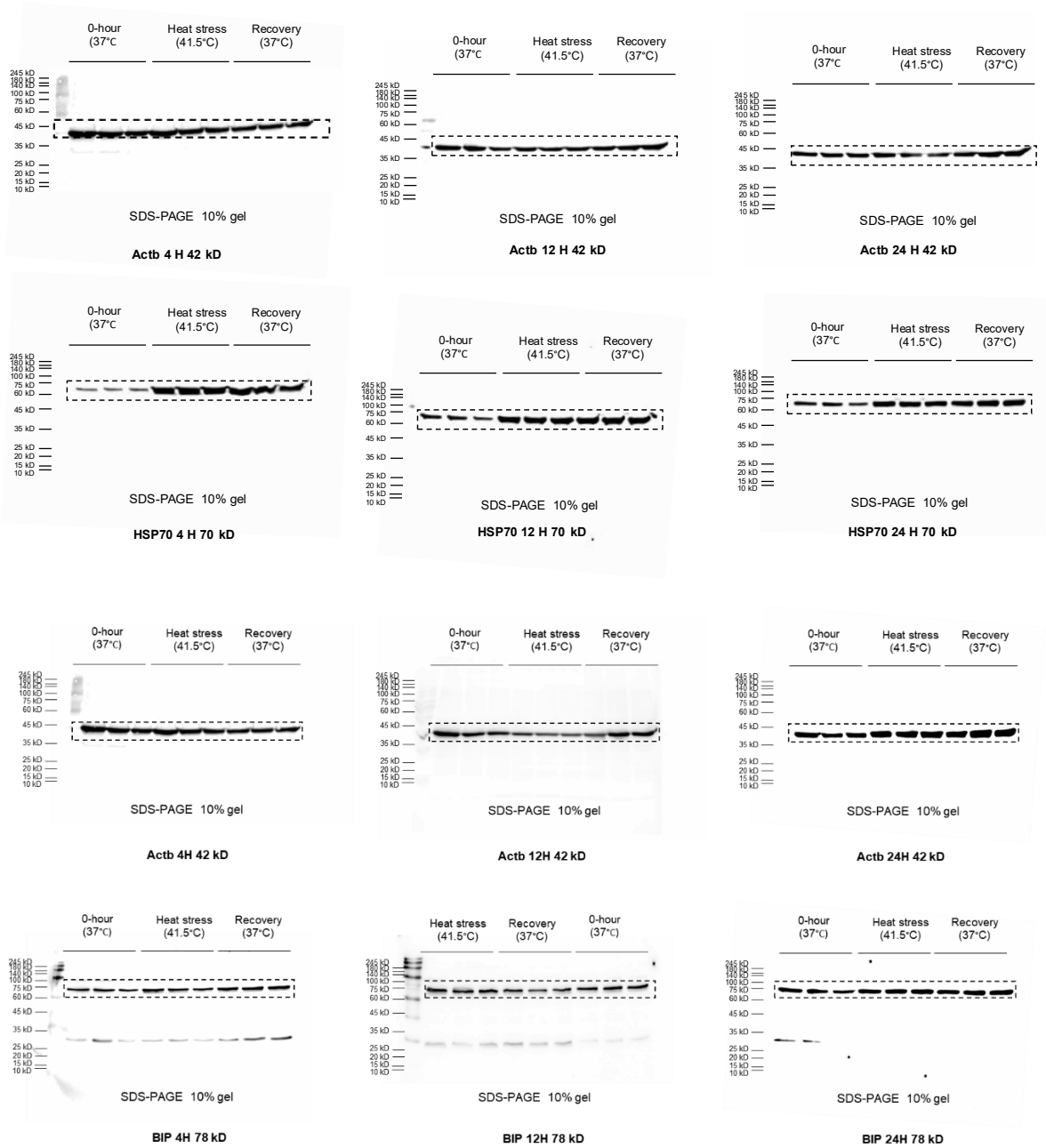

Supplement: Supplementary file 6 [file ab-250896-Supplementary-6.pdf]
