# Supplementary material for: Establishment and characterization of Hanwoo cumulus cell line for heat stress studies
Source: Anim Biosci. 2026 Jun 15;39(7):250896. doi: 10.5713/ab.250896 (PMC13353149; doi:10.5713/ab.250896)
Supplement: Supplementary file 2 [file ab-250896-Supplementary-2.pdf]

**Supplement 2. List of primers used in this study**

qRT-PCR Primers of antioxidant genes for cattle (Ref?)

| Gene symbol | Description | Primer sequence (5'-3') | Tm (°C) | Amplicon length (bp) |
|---|---|---|---|---|
| *GADPH* | Glyceraldehyde-3-phosphate dehydrogenase | F: GCCGATGCCCCCATGT | 58.4 | 80 |
| | | R: CAGGAGGCATTGCTGACAATC | 58.6 | |
| *SOD1* | Superoxide dismutase 1 | F: CACGATGGTGGTCCATGAAA | 57.2 | 80 |
| | | R: TTCCAGCGTTGCCAGTCTTT | 59.5 | |
| *CAT* | Catalase | F: TCCAAGGCGAAGGTGTTTG | 57.3 | 80 |
| | | R: CCCGATTCTCCAGCAACAGT | 59.1 | |
| *GPX1* | Glutathione Peroxidase 1 | F: CCCCTGCAACCAGTTTGG | 57.6 | 80 |
| | | R: CGCCTGGTCGGACGTACTT | 61 | |

SV40T vector primers

| Target location | Primer sequence (5'-3') | Tm (°C) | Amplicon length (bp) |
|---|---|---|---|
| Coding region of SV40-T gene | F: ATGGGAGCAGTGGTGGAATG | 59 | 351 |
| | R: GCAGACACTCTATGCCTGTGT | 59.1 | |
| Overlapping region of CMV and SV40-T sequence | F: GGCACCAAAATCAACGGGAC | 59.1 | 398 |
| | R: AGTTGCATCCCAGAAGCCTC | 59.1 | |
