# Supplementary material for: Establishment and characterization of Hanwoo cumulus cell line for heat stress studies
Source: Anim Biosci. 2026 Jun 15;39(7):250896. doi: 10.5713/ab.250896 (PMC13353149; doi:10.5713/ab.250896)
Supplement: Supplementary file 3 [file ab-250896-Supplementary-3.pdf]

Supplement 3. Microarray cumulus cells normalized raw data

ProbeID	Gene_Symbol	Gene_ID	mRNA Accession	CM12H/CM0H.fc	CM12R/CM0H.fc	CM12H/CM12R.fc
12730813	CRYAB	281719	NM_174290	11.20157739	6.239454918	1.795281405
12759325	LOC616720	616720	XM_001788477	5.00499976	2.527971152	1.979848448
12867245	SMS	615950	NM_001035471	4.862067761	3.094207611	1.571345033
12889007	CLU	280750	NM_173902	4.721057322	5.261375435	-1.114448539
12804546	HSPA1A	281825 // 282254	NM_174550	4.2594584	1.181655915	3.604652036
12726925	LYVE1	404179	NM_205815	4.171655432	2.973207186	1.403082655
12683494	SLC5A3	282362	NM_174607	4.040573285	1.649489964	2.449589493
12856372	CLEC2B	100297496	XM_010805551	4.027570939	7.985263418	-1.982649974
12840607	LRIF1	539067	XM_003581959	3.968269478	1.528111696	2.596845171
12790461	SERF2	527852	NM_001206348	3.944520677	2.355168042	1.674836193
12759819	LOC618289	618289	XM_010800228	3.759419736	2.47435071	1.519356056
12805397	ENPP5	512304	NM_001206724	3.728046875	3.07560451	1.212134676
12812811	HSPB1	516099	NM_001025569	3.632467777	1.602695111	2.266474611
12726906	TRIM21	359715	NM_182655	3.545354771	2.663863207	1.330907218
12802720	FLOT1	532573	NM_001076887	3.49276602	1.851327332	1.88662802
12717715	LOC100138864	100138864	XR_810477	3.371708935	2.26605048	1.487923135
12712971	MTUS2	616677	XM_005194136	3.340027544	1.755254224	1.902873953
12703510				3.287634688	1.400653403	2.347215007
12778599	LOC540098	540098	XR_805091	3.218235719	1.305660669	2.464833166
12751423	BANP	513446	NM_001075620	3.206612448	-1.177029109	3.774276193
12759673	ZNF585A	767967	XM_002694957	3.175205574	1.60840419	1.974134109
12868064	GDF11	540517	XM_002687421	3.161216843	2.163504318	1.461155782
12781386	SCRN3	511983	NM_001075547	3.158895036	1.250365606	2.526377101
12708565	GEN1	785690	NM_001205450	3.113311647	1.557551644	1.998849707
12733529	C15H11orf71	615529	NM_001078051	3.100798977	2.229437877	1.390843409
12903563	LOC508820	508820	NM_001075373	3.061545236	1.666475376	1.837137998
12886230	TNFAIP8	529336	NM_001083711	3.043159603	-1.094604733	3.331056905
12678907	ST3GAL6	444860	NM_001002883	2.994344057	1.436193362	2.08491707
12784117	CLK1	613808	NM_001102271	2.925209596	1.394250705	2.098051367
12847098	CCDC18	540863	XM_005193418	2.911757079	2.461879246	1.182737571
12785694	RAD1	518502	NM_001192490	2.901361376	1.229183239	2.360397769
12777737	SCN9A	533065	NM_001110787	2.855493301	1.378472035	2.071491643
12839892	ODF2L	533508	NM_001075993	2.835474142	1.695745895	1.67211028
12750919	ZNF529	510501	XM_002694950	2.83186011	1.272265705	2.225840168
12821078	BAG3	782633	NM_001082471	2.829897894	1.210187007	2.338397189
12682890	LOC100139345	100139345	XM_002685011	2.81573195	1.358062617	2.073344716
12805505	SNRNP48	513601	XM_002697597	2.809960791	1.453438472	1.933319397
12868450	RASSF9	615588	NM_001193083	2.80772181	1.777512863	1.579578898
12862891	THAP2	781850	XM_003582168	2.807643964	1.106512693	2.537380713
12728877	DEPDC7	532586	NM_001192730	2.777929527	1.879501413	1.478014067
12784836	RANBP3L	100296398	XM_002696376	2.777852507	2.168037935	1.281274863
12892282	MIRLET7F-1	791061	NR_030957	2.777756236	1.521558708	1.825599118
12873712	COMMD8	507292	XM_002688237	2.777332681	1.265958402	2.193857774
12906573	MIR105B	100312990	NR_031127	2.754327287	1.942278391	1.418090888
12718996	NSUN6	511775	NM_001083670	2.751445972	1.49634205	1.838781428
12779266	RSRP1	615263	NM_001103298	2.751331545	-1.153861448	3.1746554
12779974	MIR2351	100313159	NR_031077	2.730622727	1.403948485	1.944959346
12808650	ADCYAP1	615187	NM_001046555	2.703671739	3.304816115	-1.222343699
12902018			XM_002683967	2.672018506	2.061436469	1.296192508
12778639	CYTIP	540450	NM_001102243	2.671185191	1.456130846	1.834440358
12786691	LOC783613	783613	XM_010816713	2.658698163	1.39784074	1.902003631
12910548	LOC784704	784704	XR_816901	2.653506348	1.370944341	1.935531786
12900274	FUCA2	515729	NM_001205818	2.635104179	2.01359006	1.308659707
12886251	CLK4	529633	NM_001192988	2.633004524	1.166834542	2.256536321
12851611	TBX20	539419	NM_001192237	2.628937784	1.664512841	1.579403727
12726617	MDK	280852	NM_173935	2.626843042	1.345749603	1.951955279
12855975	BET1	616526	NM_001099157	2.618662258	1.296956417	2.019082695
12879520	PCDHGA8	521340	NM_001099705	2.613838491	1.067243962	2.449148072
12791662	HSP90AA1	281832	NM_001012670	2.603044544	1.127033188	2.309643204
12901028	SLC35A1	536838	NM_001034637	2.582162867	1.392521884	1.85430685
12772627	TMEM100	613987	NM_001046522	2.5519322	1.171088414	2.179111473
12807097	ATAT1	786491	NM_001276323	2.539861802	1.155574281	2.197921712
12713136	USPL1	788351	XM_001255437	2.538559368	1.802913274	1.408031881
12755419	ZNF729	768043	XM_005219628	2.519993478	1.573808494	1.601207191
12892464	MIR2469	100313237	NR_031008	2.519364735	1.996440395	1.261928351
12825573	UFSP2	617788	NM_001076477	2.516171049	1.134825127	2.217232408
12720706	ZNFX1	539807	NM_001205716	2.510683201	2.375415686	1.056944776
12906051	ERCC6L	782916	NM_001102530	2.501875502	1.471431111	1.700300805
12855928	RGS2	100848920 // 513055	NM_001075596	2.487764635	1.237381183	2.010507893

ProbeID	Gene_Symbol	Gene_ID	mRNA Accession	CM12H/CM0H.fc	CM12R/CM0H.fc	CM12H/CM12R.fc
12697010	LOC613512	613512	XR_809342	2.483853354	1.204195808	2.062665671
12710969	MIR15A	100170925	NR_030793	2.476032069	-1.21326946	3.004094091
12866943	FOXRED2	532871	NM_001206011	2.471248345	2.47433356	-1.001248444
12856114	LOC511712	511712	XR_803175	2.470392024	1.183180352	2.087925159
12812729	EPHB4	515756	NM_001206268	2.46874872	1.851417161	1.33343731
12753652	MED29	614626	NM_001080316	2.456697116	1.818350102	1.351058365
12908846	GABRE	524115	XM_002699672	2.449980047	2.226580852	1.100332847
12753128	CNTNAP4	536775	NM_001206511	2.439304733	2.081639149	1.171819205
12891820	C8H9orf85	614778	NM_001101235	2.399743151	1.463010375	1.640277604
12712702	RFXAP	540760	XM_002691750	2.370908523	1.199522318	1.976543902
12881344	TUBB4A	540236	NM_001034697	2.360495937	2.56558887	-1.086885526
12692579			ENSBTAT00000034439	2.356686736	1.286793006	1.831441983
12753154	ZNF175	537103	XM_001790619	2.356180395	1.484883762	1.58677767
12772102	ZFP3	538648	NM_001099089	2.354368263	1.504871186	1.564498201
12747823	HSPB8	539524	NM_001014955	2.348793694	1.011053963	2.323114077
12803821	ZSCAN12	768084	NM_001077138	2.343573432	1.810063111	1.294746806
12829760	CHORDC1	505144	NM_001045912	2.338445815	-1.161814709	2.716840744
12899712	CASP8AP2	505138	XM_010798926	2.3379596	1.134691413	2.060436497
12754336	ZNF566	767945	NM_001077017	2.324966615	1.458353028	1.594241291
12702031	SUPT7L	539152	NM_001076150	2.321987169	2.172174492	1.068968988
12805757	NKAPL	515716	NM_001102498	2.318882952	2.275036986	1.019272638
12725482	GGH	525303	NM_001105394	2.311693295	1.380566134	1.674453137
12839817	MCOLN2	532671	NM_001192734	2.304653723	-1.207622883	2.783152574
12708822	MIR2303	100313126	NR_031116	2.304254391	2.71896956	-1.179978031
12816236	ZNF500	510490	NM_001105353	2.303695443	1.121041744	2.054959556
12786853			ENSBTAT00000009630	2.300009787	1.218976235	1.886837266
12732358	MPZL3	521837	NM_001098051	2.299579381	1.126619228	2.041132729
12847867	RNPC3	768060	NM_001077117	2.295454762	1.25304654	1.831899046
12910712	SCML1	786670	XM_001254287	2.293641638	1.671403428	1.372284871
12753873	ZNF576	616814	NM_001076433	2.292004696	1.331959301	1.720776825
12895395	LOC616883	616883	XM_002689834	2.291305776	-1.021465162	2.340489025
12878480	ZNF354B	510078	XM_005193344	2.288829504	1.420619309	1.611149088
12808949	LOC100295873	100295873	XR_815221	2.28867086	2.561572989	-1.119240443
12905870	KANTR	617166	NR_132122	2.271964039	1.237535576	1.835877758
12790978	PAX9	540196	NM_001192369	2.271098061	1.946267485	1.166899247
12871206	PARM1	518368	NM_001075771	2.264135028	2.280926566	-1.007416315
12815040	PGP	538173	NM_001038172	2.261014117	1.708416812	1.323455788
12845158	BTBD8	520456	NM_001105200	2.260857401	1.571693607	1.438484823
12810708	C24H18orf32	767921	NM_001195062	2.254832083	1.693220663	1.331682356
12886623	LOC100851455	100851455	XM_005193529	2.24827732	-1.091165549	2.453242756
12777779	DNAJB2	533668	NM_001034592	2.241555158	1.529839178	1.465222743
12729666	SLC35C1	540413	NM_001101210	2.234884075	2.643189755	-1.182696581
12732550	LOC528515	528515	XM_002693239	2.229916981	1.463010375	1.524197654
12816222	AGFG2	510361	NM_001206722	2.226519119	2.77052618	-1.244330739
12737711	LOC784034	784034	XM_001252463	2.223280535	1.326891126	1.675556111
12754347	HSBP1	767979	NM_001113316	2.222340687	-1.028548625	2.285785458
12730651				2.222155845	1.487345691	1.49404127
12697892	LOC790312	790312	XM_002690745	2.217247777	1.789107406	1.239303895
12858232	KMT2D	506805	XM_010805028	2.216955789	1.895699117	1.169466066
12737308	MARC2	615506	NM_001076380	2.215634641	1.452602532	1.525286231
12841195			ENSBTAT00000033541	2.208627318	1.296498017	1.703533124
12735978	TNFRSF9	520341	NM_001035336	2.2081834	2.137597166	1.033021299
12851621	FAM71F1	539444	NM_001046457	2.201611636	1.670395812	1.318017933
12825157	SARAF	515461	NM_001076856	2.199948879	1.269939704	1.732325458
12905810	FANCB	616549	XM_010800601	2.19818072	-1.451626199	3.190936723
12904277			ENSBTAT00000020218	2.194420493	1.128565381	1.944433641
12680464	DTX3L	515051	NM_001192396	2.187328644	1.622333025	1.348261183
12795924	CAMK1	520498	NM_001076868	2.186070609	1.50857874	1.449092812
12808216	ANKRD12	534739	XM_002697743	2.183859436	2.03292924	1.07424272
12746863	LOC101906230	101906230	XR_812325	2.18290599	1.518629565	1.437418341
12740874	TRMT1L	540872	NM_001098121	2.181862218	1.24601376	1.751073936
12851673	UBN2	540792	NM_001193208	2.18004815	1.67581164	1.300890923
12741933	PPID	281420	NM_174153	2.177963837	1.098458222	1.982746175
12772695	CORO6	614661	NM_001102282	2.177888356	1.210044413	1.79984167
12851464	C4H7orf60	538658	XM_002686799	2.176756453	1.233895315	1.764133818
12707526	ZBTB26	100848248	XM_003582824	2.176620664	1.393661312	1.561800306
12736714	TMEM183A	534797	NM_001034616	2.176318941	-1.109438734	2.41449253
12767392	CALCOCO2	281942	NM_174400	2.171090704	1.288211968	1.685352068
12813124	ZNF394	522836	NM_001077955	2.160776718	1.693866295	1.275647744
12773248	HOXB4	768240	NM_001078114	2.159923177	1.382625079	1.562190075

ProbeID	Gene_Symbol	Gene_ID	mRNA Accession	CM12H/CM0H.fc	CM12R/CM0H.fc	CM12H/CM12R.fc
12815778	KIAA0430	506615	NM_001271994	2.155077871	1.344295218	1.603128422
12691680	ICE2	541255	NM_001076265	2.146490816	1.690019625	1.27009816
12841430	PPAP2B	617707	NM_001076473	2.146059387	1.992003248	1.077337293
12831467	PRCP	534927	NM_001038164	2.144096739	2.108080559	1.017084821
12805356	ZKSCAN8	511794	NM_001100344	2.142507122	1.345964165	1.591801014
12691075	SV2C	538430	NM_001192019	2.139464885	1.10292906	1.939802806
12895239	UHRF2	613759	NM_001102270	2.139301765	1.22959227	1.739846466
12877262	MFAP3	317721	NM_001166519	2.135020615	1.302271269	1.639459201
12785577	CREBRF	513587	XM_002696253	2.132594985	1.116412358	1.910221585
12826652	DDX50	534331	NM_001206053	2.126764072	2.454637528	-1.15416541
12699808	RPL7A	513128	NM_001040520	2.12657244	1.087986006	1.954595398
12750962	TXNL4B	510719	NM_001014897	2.121243149	1.595645314	1.329395154
12781532	XKR8	514365	XM_002685687	2.120728594	1.475731238	1.437069664
12810622	TIMM21	614633	NM_001035453	2.119949649	1.297262107	1.634172183
12849348	TMEM176A	404082	NM_205779	2.1154726	1.53453335	1.378577142
12855706	LSMEM1	613798	NM_001099718	2.115032746	1.1766865	1.797447957
12798858	TATDN2	510836	XM_002696881	2.114930126	1.44413939	1.464491683
12865041	FKBP4	508535	NM_001034322	2.113010593	-1.009408402	2.132890646
12697644				2.111883131	1.079437715	1.956465947
12843761	ADAMTSL4	507654	NM_001101061	2.110536822	2.231525047	-1.057325806
12857312	CBY1	282859	NM_174739	2.107393903	2.270263886	-1.077285022
12836937	OMA1	506223	NM_001035033	2.105335274	1.318831271	1.596364388
12842922			ENSBTAT00000043099	2.096975492	1.466970643	1.429459752
12846170	RABGGTB	533276	NM_001015646	2.09569679	1.494362337	1.40240204
12738517	PLOD1	281409	BT025353	2.094839916	1.908448162	1.097666658
12753239	NFAT5	538523	XM_002694839	2.094041451	1.5386339	1.360974466
12747503	SPPL3	535156	XM_002694525	2.093083694	2.140710937	-1.022754582
12887373	ARSI	540390	XM_002689292	2.089894333	1.0463315	1.99735393
12865903	SLC6A12	514339	NM_001101994	2.08861995	1.55980967	1.339022312
12881643	MRPL22	614639	NM_001035454	2.088576518	1.494880333	1.397152984
12880946	ARHGAP26	538219	NM_001205522	2.088127782	1.641540108	1.272054074
12894545	RNF38	532877	NM_001034581	2.086492884	1.458474336	1.430599656
12754730	LOC787397	787397	XM_010814918	2.080196767	1.509405044	1.378156762
12761520	ZNHIT3	504662	NM_001045895	2.079591264	1.654711839	1.256769436
12865887	DDX17	514323	NM_001101993	2.07957685	-1.279287035	2.660375703
12820493	SUFU	535067	NM_001098083	2.07957685	1.799006002	1.155958817
12745816	HSPA4L	506096	XM_002707745	2.079375056	-1.010990892	2.102229243
12753012	DEDD2	534251	NM_001076017	2.078236731	-1.031447221	2.143591499
12861727	WNT10B	539337	XM_002687290	2.077617398	1.171900432	1.772861704
12814558	YPEL3	787498	NM_001205409	2.077171016	1.881665258	1.103900392
12704139	EIF2AK2	347700	NM_178109	2.076379285	1.226519355	1.692903807
12891839	TMEM38B	615646	NM_001076387	2.06445361	1.405564832	1.46877153
12706234	DPM2	523737	NM_001046268	2.061679393	1.795978401	1.147942198
12758837	TOX3	539135	XM_002694777	2.060736438	1.691543174	1.218258256
12890878	DNAJA1	528862	NM_001015637	2.059808191	1.096951697	1.87775651
12776458	EYA3	514364	NM_001206731	2.059522661	1.520599266	1.354415135
12780621	PGAP1	504677	NM_001205550	2.059465559	1.182467064	1.741668433
12879363	CYFIP2	518833	NM_001205850	2.059023078	1.769252552	1.163781324
12844109	INTS3	511052	NM_001193087	2.056498472	1.429548929	1.438564592
12754885	PAPD5	789894	XM_010814610	2.054845608	1.267407098	1.621298801
12793887	MBIP	774027	NM_001077518	2.054560765	1.220049769	1.683997504
12872978	MIR2450B	100313224	NR_031191	2.053578362	1.440540279	1.425561224
12885399	ERAP1	514617	NM_001102003	2.050022864	1.708215512	1.200096152
12799813	RBM5	534216	NM_001046374	2.048133848	1.437926566	1.424366095
12852248	ARL4A	767906	NM_001076985	2.04786413	1.331645435	1.537844893
12835058	B4GAT1	618055	NM_001034808	2.046927494	1.158413243	1.767009749
12808661	RTTN	615378	NM_001206207	2.043680968	1.683810753	1.213723671
12838581	HENMT1	515853	XM_002686160	2.040821496	1.430946764	1.426203649
12789359	PRPF39	505547	NM_001109789	2.037994277	1.421821149	1.433368942
12854094	SAMD9	514205	NM_001205781	2.036892723	2.206928668	-1.083478105
12709831	PIBF1	524913	XM_002691887	2.036610369	1.220041312	1.669296236
12697609	GCNT4	782825	XM_005200869	2.03140796	1.637869032	1.240274968
12869555	EREG	100295476	XM_010797936	2.03040848	2.041330812	-1.005379376
12808744	PHLPP1	615982	XM_002707786	2.027384884	1.371476593	1.478249716
12838432	MCOLN3	514345	NM_001192367	2.027160053	1.366475885	1.483494934
12906463	TMEM257	101901895	XM_005228587	2.026232885	-1.451485339	2.941047327
12754295	ZNF181	767826	NM_001076549	2.025966052	-1.05338294	2.134118077
12713011	DCUN1D2	618507	XM_010811009	2.024899071	1.157169337	1.749872733
12724392	TRPA1	505317	XM_010812107	2.022710711	1.696792325	1.192079126
12696838	VCPKMT	540184	NM_001110083	2.019110686	1.509781737	1.337352702

ProbeID	Gene_Symbol	Gene_ID	mRNA Accession	CM12H/CM0H.fc	CM12R/CM0H.fc	CM12H/CM12R.fc
12722156	TATDN1	509365	NM_001075402	2.018830797	1.322859645	1.526111107
12759624	ADAT1	618521	NM_001081620	2.016928582	1.268646388	1.589827237
12795463	EIF1B	513481	NM_001037598	2.012027467	1.315644763	1.529309068
12741155	TATDN3	615965	NM_001080320	2.011344214	1.33491696	1.506718601
12760151	ZNF583	789836	NM_001083534	2.010493957	1.530390688	1.31371288
12753402	ZNF432	539365	NM_001192232	2.009337628	1.044157975	1.924361712
12774536	HSPE1	281833	NM_174346	2.008140206	1.145748185	1.75268897
12799774	MLH1	533652	NM_001075994	2.005580675	1.50962477	1.328529258
12685693	KLHL24	533510	NM_001206196	2.003260446	1.042379055	1.921815713
12686285	MYNN	539376	NM_001035424	2.002941104	-1.015169057	2.033323831
12773273	SMCR8	781661	XM_003583598	2.002788394	2.095987336	-1.046534593
12875155	PAQR3	534876	NM_001206068	2.002136033	1.517451072	1.319407307
12725871	PLAG1	539210	XM_002692655	2.001969507	-1.415292258	2.833371943
12833395	CTSF	509715	NM_001075416	2.00120644	2.221231869	-1.109946393
12748975	LOC100299712	100299712	XM_005219581	1.994282792	1.277718478	1.560815489
12895265	GKAP1	613960	NM_001040573	1.989547026	1.253567776	1.587107665
12754148	LOC104974934	104974934	XM_010815399	1.983845948	-1.411667186	2.800530227
12701712	TRMT61B	535837	XM_001790235	1.983804695	2.105700132	-1.061445281
12729991	RNF214	616238	NM_001206820	1.980205285	2.017473887	-1.018820575
12691345	ZC2HC1C	540480	XM_005193743	1.969869422	1.569451014	1.255132785
12721001	ECHDC3	617368	NM_001193156	1.968586355	2.025376335	-1.028848102
12771254	SLC25A35	527669	NM_001031763	1.966990514	1.46778433	1.340108675
12682311	LOC614129	614129	XR_083075	1.959927108	1.307698541	1.498760645
12880985	DNAJB1	538426	NM_001033763	1.957239088	-1.091770787	2.136856459
12691887	TMEM260	614796	NM_001099139	1.954907032	1.127814659	1.733358417
12908896	UXT	525680	NM_001037471	1.95429736	1.616552094	1.208929404
12772826			ENSBTAT00000006464	1.948089554	1.612858642	1.207848911
12818565	FOPNL	782821	NM_001101286	1.946442869	1.882108762	1.034181928
12681770	ZMAT3	538512	NM_001076108	1.9457684	1.224998515	1.588384293
12689708	VPS18	518781	NM_001100380	1.940946026	1.485408769	1.306674679
12854397	ORC5	519409	NM_001099704	1.935048867	1.523468848	1.270159787
12758399	ZNF416	530973	XM_002695492	1.92856798	-1.21535687	2.343898343
12779684	LOC785710	785710	XR_804983	1.927685906	1.316009588	1.464796247
12791746	NFKBIA	282291	NM_001045868	1.926270085	1.298629601	1.483309855
12745062	USP30	100140210	NM_001191361	1.924001602	2.456339543	-1.276682691
12778449	ID3	538690	NM_001014950	1.911811124	1.31244776	1.456675977
12701187	AHSA2	531017	NM_001102134	1.911691862	-1.264958452	2.418210779
12807542	NPC1	286772	NM_174758	1.909784694	1.950048492	-1.021082899
12790914	LINS	539267	NM_001205649	1.90421978	1.40559406	1.354743758
12774686	HMGCL	317658	NM_001075132	1.9040482	1.828955534	1.041057677
12754725	ZNF471	787287	XM_001254729	1.901884981	2.268737981	-1.192889162
12789116	CYP1A1	282870	XM_002696635	1.89692153	-1.074458678	2.038163799
12888026	GADD45B	618405	NM_001040604	1.896540263	2.340635037	-1.234160478
12872976	MIR2450C	100313223	NR_031190	1.895751678	-1.09066648	2.067632809
12805654	CAP2	515190	NM_001098979	1.89358476	-1.075203694	2.035989329
12847465	BTBD19	615438	NM_001101241	1.891079481	1.248529578	1.514645318
12694538	PARP6	511329	NM_001103241	1.883792421	1.603406247	1.174869079
12874660	HSD17B11	527592	NM_001046286	1.880087751	1.409281681	1.334075208
12902050			XM_003583467	1.871973484	-1.114178205	2.085712057
12833481	PCF11	510604	XM_010820857	1.86800718	1.295590684	1.441818934
12795124	TRANK1	509859	XM_002696940	1.864475707	2.136071591	-1.14566877
12896966	TSPYL4	508104	NM_001101064	1.859210343	1.569135566	1.184862789
12828142	P4HA1	518288	NM_001075770	1.850993719	-1.105822629	2.046870741
12806585	MYLIP	541070	NM_001102253	1.848032335	1.301323815	1.420117202
12748602	ZNF570	100138971	NM_001191353	1.843886687	1.382644247	1.333594445
12714001	ID1	497011	NM_001097568	1.841421632	1.222614854	1.506133863
12860539	SLC38A4	527476	NM_001205943	1.841153613	-1.14077953	2.100350354
12682021	GTF2E1	540525	NM_001103294	1.838475563	1.764023769	1.042205664
12837074	TXNIP	506790	NM_001101875	1.836679629	1.420333776	1.293132404
12746819	ZNF280B	517697	NM_001077935	1.836170464	1.150443394	1.596054593
12900168	ZBTB2	513775	NM_001205768	1.835470592	1.482806146	1.237835841
12895667	SVEP1	781282	XM_002705057	1.833232795	2.325337299	-1.268435359
12829082	H19	100126192	NR_003958	1.821339667	1.086923196	1.67568387
12688722	HAUS2	508698	NM_001076819	1.819169536	1.374245733	1.323758548
12844992	LOC516742	516742	XM_002686058	1.818148451	4.308094809	-2.36949563
12724433	NOV	505727	NM_001102382	1.816447917	1.794522485	1.012217976
12699996	CHCHD5	514560	NM_001037601	1.815000568	-1.128346369	2.0479493
12738537	SELE	281484	NM_174181	1.81429619	2.170127791	-1.196126521
12868298	HELB	614572	NM_001206182	1.807981606	1.329690056	1.359701532
12684497	HSPBAP1	513211	NM_001014911	1.800303325	-1.166333201	2.099753539

ProbeID	Gene_Symbol	Gene_ID	mRNA Accession	CM12H/CM0H.fc	CM12R/CM0H.fc	CM12H/CM12R.fc
12874999			ENSBTAT00000003877	1.800290846	-1.036578949	1.866143592
12834357	TMEM86A	532018	NM_001035388	1.798282902	2.397166304	-1.333030694
12853877	POT1	511292	XM_002686916	1.796924757	-1.054654167	1.895134182
12860511	CCDC77	527084	NM_001102106	1.783634487	-1.291287279	2.303184524
12776966	SYF2	522372	XM_002685769	1.77612116	1.17385971	1.513060841
12765241	CTNS	613527	NM_001102264	1.775702631	2.707910394	-1.52497966
12708934	LOC520016	520016	XM_005199553	1.772566804	-1.325080493	2.348793694
12726404	LOC100296550	100296550	XM_002693189	1.769203498	-1.004828995	1.777746973
12798341	CRELD1	504854	NM_001014851	1.765161272	1.465070409	1.204830335
12715842	TTPAL	526582	NM_001205931	1.760176377	2.268077598	-1.288551322
12851820	THAP5	614615	NM_001076339	1.758944546	-1.239475711	2.180169041
12829222	LOC100301320	100301320	XM_002699128	1.757530834	1.349514053	1.302343485
12707555	ZER1	540351	NM_001205808	1.747400126	2.119934955	-1.213193775
12724001	LOC100299242	100299242	XR_811002	1.735378066	-1.430877336	2.483113144
12906065	LOC104970028	104970028	XM_010799725	1.735354009	1.101347696	1.575664085
12901444	LRP11	617450	NM_001206831	1.735053321	1.162854022	1.492064601
12738690	TNFRSF1B	338033	NM_001040490	1.732937952	2.181348079	-1.258757174
12905449	KLHL15	540246	NM_001205799	1.730573241	1.277266878	1.354903404
12749545	BCAM	282862	NM_174741	1.725757794	2.281733027	-1.322162957
12767655	GABARAP	327715	NM_001034048	1.720085168	1.71259023	1.004376376
12755092	ZNF345	518207	ENSBTAT00000006317	1.711534057	-1.229481477	2.10429942
12710135	SPG20	534027	NM_001077996	1.710241429	1.225142871	1.395952643
12908237	PHF8	513959	XM_002700122	1.707197537	2.304446062	-1.349841487
12700376	ATAD2B	518785	NM_001206750	1.705884536	1.477901378	1.154261415
12711421	BRCA2	507069	XM_002684277	1.703108089	2.26677312	-1.33096257
12842687	HSD3B1	281824	NM_174343	1.701267498	1.975626191	-1.161267228
12785958	GFM2	527467	NM_001102109	1.69263394	1.044252068	1.620905519
12869323	LOC786295	786295	XR_806897	1.687994262	1.504391437	1.122044582
12882982	LOC104972838	104972838	XM_010807286	1.686041445	1.598301965	1.054895434
12859545	LETMD1	514595	NM_001083430	1.680627504	2.303344174	-1.370526287
12904467	SCML2	523477	NM_001192580	1.661343044	1.154549477	1.438953528
12907389	TCEANC	504389	NM_001024472	1.660617722	2.098080452	-1.263433736
12724157	CA2	280740	NM_178572	1.658294223	1.030468211	1.609262862
12682510			GENSCAN00000013473	1.656260958	-1.405564832	2.327982156
12838206	FUBP1	513562	NM_001076846	1.656077283	-1.394579326	2.309531142
12780807	GCA	507139	XM_002685376	1.653427744	1.156287376	1.429945339
12846879	PPCS	539294	NM_001102228	1.653198546	1.328280647	1.244615398
12791907	FOXA1	503579	NM_001206029	1.64698795	1.545121191	1.065928006
12890318	CTSL	515200	NM_001083686	1.641904254	1.309122407	1.254202239
12745498	P2RX7	286814	NM_001206516	1.639754688	2.254066377	-1.374636336
12815042				1.6377101	-1.402936781	2.297603736
12742074	TRPC3	286866	NM_001104960	1.636280405	-1.25364598	2.051316352
12874543	ALPK1	524375	XM_002688076	1.633889027	2.299164992	-1.407173286
12852612	TMEM196	100140139	NM_001206771	1.632496615	-1.551614324	2.533005132
12887990	NUDT12	617720	NM_001046608	1.630992333	1.174209635	1.389012902
12764961	KCTD11	539167	NM_001035421	1.630246362	2.246844061	-1.378223632
12701968	TMEM178A	538491	NM_001192028	1.630133366	2.022724732	-1.240833894
12867117	NABP2	533842	NM_001101151	1.630065572	1.663959132	-1.020792759
12793939	LOC783909	783909	XR_813909	1.625270667	3.549485696	-2.183935124
12802769	TBC1D7	532704	ENSBTAT00000022819	1.624538574	1.556720564	1.043564665
12809357	ZNF397	506448	NM_001075262	1.616126357	1.707765634	-1.05670304
12803451	MBOAT1	541284	NM_001192857	1.610010392	1.350534037	1.192128704
12885292	MYO9B	513493	NM_001193096	1.609642162	2.013673805	-1.25100712
12736829	RERE	535394	XM_001790418	1.605597193	2.075645404	-1.292755999
12771385	KRBA2	530088	XM_002695875	1.598512472	-1.057362451	1.690207065
12901165	HIVEP2	540396	XM_005199423	1.59811364	2.051359008	-1.283612728
12750702	TRAPPC2L	509955	NM_001098920	1.592717059	1.600718922	-1.005024032
12862258	RNF41	614134	NM_001046525	1.585821071	1.729709786	-1.090734521
12689068	GNPNAT1	512299	NM_001075559	1.581233034	-1.368494845	2.163909255
12775398	STRADB	506029	NM_001192081	1.580893302	1.228893592	1.286436281
12876894	GPX3	281210	NM_174077	1.579666491	3.043729183	-1.926817591
12799988	ATXN7	536833	XM_002696986	1.579633643	1.307517268	1.20811685
12718784	AHCY	508158	NM_001034315	1.579535104	1.165306933	1.355467009
12777446	INHBB	530430	NM_176852	1.577467191	1.177290211	1.339913622
12834765	SF1	541229	NM_001081614	1.575729616	1.248503616	1.262094555
12848182	LOC785516	785516	XR_805351	1.571486632	-1.41204885	2.219015892
12716981	C13H20orf196	615129	NM_001046552	1.570800541	1.813302978	-1.154381432
12906577	MIR224	100313023	NR_031149	1.566473105	1.367480253	1.145517898
12840461	C3H1orf43	538459	NM_001015666	1.565800056	-1.013397377	1.58677767
12825959	GHITM	404143	NM_001034052	1.563186594	1.317524692	1.186457152

ProbeID	Gene_Symbol	Gene_ID	mRNA Accession	CM12H/CM0H.fc	CM12R/CM0H.fc	CM12H/CM12R.fc
12872030	AREG	538751	NM_001099092	1.553551427	2.059836746	-1.325888998
12891980	PTPLAD2	618814	NM_001076522	1.552302795	1.338790297	1.15948166
12868668	CEP83	618145	NM_001206525	1.550227553	-1.058660494	1.641164667
12836111	PIK3R3	286865	NM_174796	1.544157595	1.470442123	1.050131502
12827415	B3GALNT2	100125310	NM_001103343	1.543141118	-1.289060522	1.989202294
12864136	ITFG2	282275	NM_174562	1.542242895	1.688193179	-1.094635082
12807296	PMAIP1	100296226	XM_002697779	1.540960627	1.307481017	1.178572084
12773566	LOC788196	788196	XM_002696017	1.537088252	-1.709009006	2.626897667
12684570	YEATS2	514192	NM_001105367	1.530677127	1.531080354	-1.000263431
12723396	GDAP1	613472	NM_001101222	1.530327042	1.17399804	1.303517544
12720367	RBM38	533516	NM_001206023	1.527836327	1.348906172	1.132648333
12899023	IRAK1BP1	782235	NM_001110093	1.520230411	1.14374069	1.32917402
12771193	ENDOV	526597	NM_001079777	1.519598297	1.345348558	1.129520144
12826200	TET1	513640	XM_010820679	1.51938765	1.004237149	1.512976941
12735104	DNM3	506315	NM_001243284	1.516757032	1.286177717	1.179274848
12831635	SPTY2D1	539967	NM_001205738	1.514047009	1.368286176	1.106528032
12900979	FOXO3	535530	NM_001206083	1.512075314	1.395314173	1.083680897
12771754	METRNL	534297	NM_001206050	1.508871556	2.026682368	-1.343177529
12882137	CYSTM1	777789	NM_001078164	1.506645496	1.147775114	1.312666111
12855087	TRA2A	534586	NM_001046380	1.505340653	1.409819045	1.067754516
12720825	SEC61A2	614128	NM_001046524	1.504965067	1.575041673	-1.046563609
12788410	CEP72	617312	XM_005199617	1.502421902	2.126159752	-1.415154923
12717330	PTER	782020	NM_001101282	1.499987003	1.290866673	1.161999945
12708470				1.493192327	-1.076493792	1.60741227
12707896	LOC614219	614219	XR_809762	1.492974992	2.049752898	-1.372931836
12762156	RNF157	507697	NM_001205618	1.491795727	2.03889856	-1.366741118
12744793	ANAPC10	783986	NM_001080357	1.491206445	-1.364970716	2.035453128
12891928	LOC616903	616903	XM_005199316	1.489625836	2.436381407	-1.635566025
12905351	STK26	539445	NM_001163786	1.487737504	1.235778347	1.203887014
12835724	DBT	280759	NM_173905	1.484811717	1.09485514	1.356171846
12823748	PROSC	509643	NM_001034357	1.483793165	1.409652929	1.052594674
12700959	HS1BP3	526092	NM_001046280	1.481347384	1.738869905	-1.173843437
12785900	SERF1A	526395	NM_001113301	1.4767852	1.386684902	1.064975322
12864864	ATF1	506967	NM_001075289	1.475086952	1.117116773	1.320441146
12760968	CHRNB1	282179	NM_174516	1.473370232	1.292173685	1.140226155
12779978	MIR2353	100313161	NR_031070	1.47023829	-1.41519416	2.080672642
12792573	ACSBG1	515577	NM_001024548	1.467702941	-1.385282294	2.033182897
12821704	ANKRD22	504991	NM_001081579	1.466909635	1.521474337	-1.037197044
12823401	LOC101908780	101908780	XR_232529	1.466055784	-1.103135492	1.617258169
12873212	SPP1	281499	NM_174187	1.464278526	1.274383948	1.149008922
12690161	JMY	528498	XM_002690458	1.46412629	1.157089131	1.265353075
12827055	ADIRF	613941	NM_001114513	1.462990093	-1.471502507	2.152793589
12681397	PLOD2	533642	NM_001101149	1.460254671	-1.005525731	1.468323646
12729020	LOC101905272	101905272	XR_237504	1.459738555	1.817279091	-1.244934639
12795739			ENSBTAT00000000611	1.459657612	1.382979719	1.055443975
12851791	GTPBP10	613957	NM_001080314	1.459060797	1.087262278	1.341958447
12862249	BHLHE41	613907	NM_001076297	1.457746643	1.805051501	-1.23824775
12748985	LOC104968473	104968473	XM_010815384	1.455908814	-1.501474528	2.186009999
12702649			ENSBTAT00000011846	1.45262267	2.053151377	-1.413409979
12864483	LTBR	504653	NM_001103228	1.452250171	1.962210748	-1.351152017
12861857	PIP4K2C	540005	NM_001076196	1.449343942	1.490472751	-1.028377535
12772311	KRT31	539597 // 618453	NM_001099099	1.448319604	-1.476754492	2.13881248
12739608	ATF3	515266	NM_001046193	1.447938172	1.141230334	1.268751915
12761115	STAT5A	282375	NM_001012673	1.447486607	2.120772694	-1.465141496
12770183	SYNGR2	513812	NM_001100358	1.438963502	1.245228067	1.155582291
12776899	SLC39A10	521004	NM_001205880	1.436671279	-1.055517136	1.516431153
12702331	AFTPH	540837	XM_001790625	1.434581568	1.521970083	-1.060915682
12697491	ATG12	767903	NM_001076982	1.434521907	1.081362326	1.32658765
12846468	LRIG2	535493	NM_001192860	1.434362822	1.072873514	1.336935625
12720838	MKKS	614288	NM_001206171	1.433865796	1.089321643	1.316292397
12791835	TJP1	407102	XM_005196201	1.433041113	1.517808732	-1.059152259
12815109	TPT1	326599	NM_001014388	1.432623984	1.0461502	1.369424758
12900065	OR4A15	511653	XM_002683697	1.432554474	1.078480432	1.328308268
12865876	TMEM106C	514271	NM_001034461	1.431343562	1.392319202	1.028028314
12717971	CST3	281102	NM_174029	1.430431091	1.463334917	-1.023002733
12752300	SEPW1	523694	NM_001163225	1.427726858	1.340536034	1.065041761
12848618	CHPF2	100616099	NM_001206923	1.427321169	1.565528747	-1.096830048
12696534	ZBTB25	538889	NM_001164023	1.427054072	-1.605196594	2.290702335
12784152	FAM171B	614438	NM_001206755	1.425590868	1.121033973	1.271674991
12871398	PLA2G12A	527214	NM_001098063	1.424751192	1.334528393	1.067606504

ProbeID	Gene_Symbol	Gene_ID	mRNA Accession	CM12H/CM0H.fc	CM12R/CM0H.fc	CM12H/CM12R.fc
12756068	CFAP20	445424	NM_001003905	1.423754103	1.02666108	1.386781023
12806978	OARD1	768056	NM_001077113	1.423527141	-1.433786288	2.041033695
12889316	DDX58	504760	XM_002689480	1.421693036	2.770238138	-1.948548715
12859375	A2M	513856	NM_001109795	1.418818454	2.882378908	-2.03153469
12845808	DNALI1	529053	NM_001191299	1.416234337	2.160701832	-1.525666887
12790479	DNAJA4	528615	NM_001102120	1.415694527	-1.47111497	2.082649412
12793470	ARRDC4	538517	NM_001192034	1.415674902	2.244462512	-1.585436395
12747034	ULK1	526310	NM_001205927	1.415017603	1.528863918	-1.080455759
12727205	ATG13	507340	NM_001076812	1.413939117	1.416715431	-1.001963532
12820687	STAMBPL1	538785	NM_001076129	1.411491069	1.205256324	1.171112767
12889017	LPL	280843	NM_001075120	1.411119337	1.385503159	1.018488718
12734488	FMOD	281168	NM_174058	1.409682242	2.068607597	-1.467428286
12864197			NM_174719	1.409477062	1.226774429	1.148929281
12824753	PLAT	281407	NM_174146	1.405730467	1.971221641	-1.402275677
12816240	ROGDI	510492	NM_001075452	1.401673176	2.315205092	-1.651743881
12732105	TIMM10B	514937	ENSBTAT00000004639	1.398112061	1.479807997	-1.058433038
12774409	MAP2	281294	NM_001205807	1.396097791	1.192434482	1.170796226
12691905	TMEM55B	615077	NM_001076359	1.396049407	1.298701615	1.074957782
12834190	TMEM135	523885	NM_001035348	1.395246474	1.152151154	1.210992559
12890108	ZCCHC7	511821	NM_001046100	1.394888688	1.294594249	1.077471717
12712605	SPRY2	539090	NM_001076147	1.393632332	1.284582903	1.084890924
12711245			GENSCAN00000015974	1.392811481	1.038693498	1.34092635
12687663	MAP2K5	100295732	NM_001206770	1.391238729	-1.18304914	1.645903782
12890642	TRIM32	521975	NM_001075824	1.388521966	-1.466970643	2.036920961
12753538	KCNJ14	540740	NM_001206021	1.386742574	-1.495263766	2.073545924
12786659	ACTBL2	780991	XM_010816767	1.385656825	2.181166647	-1.57410306
12890889	NR4A3	528877	XM_005199247	1.383842736	2.535517093	-1.832229217
12828783	RHOBTB1	540513	NM_001082453	1.383353627	1.063190412	1.301134407
12872507	TRMT10A	616544	NM_001035477	1.38120743	1.112588424	1.241436096
12711770	IFT88	514177	NM_001110786	1.381130842	1.149781721	1.20121134
12766656	LOC100140873	100140873	XM_001789469	1.381121268	2.611448042	-1.89081734
12792498	CPEB1	514174	XM_010817000	1.377726959	1.107955548	1.243485772
12830417	RBM14	511512	NM_001014901	1.377621917	1.091256313	1.262418279
12864750	LARGE	506466	NM_001205588	1.37545601	1.337009763	1.028755398
12843848	LOC508547	508547	XM_005197743	1.373341104	1.355307297	1.013306065
12800563	PPARG-TSEN2	724061	NR_003096	1.373236396	1.049098399	1.308968156
12687319			ENSBTAT00000065519	1.371552646	2.542380563	-1.853651459
12712601	POLR1D	539061 // 616972	NM_001076144	1.371077384	1.253767641	1.093565777
12797808	MIR2370	100313422	NR_030980	1.365955041	1.208141972	1.130624606
12764421	ADPRM	534038	NM_001103279	1.364895028	1.523722306	-1.116365929
12801900	CDKN1A	513497	NM_001098958	1.363968191	1.487881882	-1.090847933
12836092	PI4KB	286846	NM_174783	1.363268752	2.03939326	-1.495958341
12740137	RCOR3	532635	NM_001192733	1.362191939	1.609285172	-1.181393844
12721420			XM_005215689	1.36166329	-1.29615657	1.764928819
12900397	TMEM242	517251	NM_001046228	1.361587785	1.219449488	1.116559397
12897474	KIAA1244	522232	XM_002690274	1.36153116	1.053156618	1.292809765
12878068	P4HA2	507327	NM_001034293	1.360644332	-1.056183128	1.437089586
12809919	RIOK3	522917	NM_001075836	1.358665206	-1.008967707	1.370849317
12851902	RP9	100336120	XM_002704099	1.353861351	1.12039698	1.208376473
12784595	LOC783612	783612	XR_082746	1.351049001	-1.903388421	2.571571024
12857852	HCFC2	505341	NM_001192948	1.350871082	1.305072541	1.035092716
12858048	SMDT1	506149	NM_001045937	1.350730636	-1.066704076	1.440829875
12770107	CTC1	513500	XM_002695868	1.348223802	1.362021994	-1.010234348
12808553	SYT4	539867	NM_001098108	1.347196224	1.097446035	1.227574004
12864461	MGST1	493719	XM_005206974	1.345553729	1.497005996	-1.112557577
12848033	GSTM1	327709 // 783879	NM_001080354	1.34122381	1.634319444	-1.218528505
12762513	EIF1	509764	NM_001014884	1.340043654	1.178866214	1.136722419
12878199	CALR3	508555	NM_001038514	1.338456266	1.116799345	1.198475154
12720619	TRIB3	538465	NM_001076103	1.33557408	-1.066837173	1.424840076
12825680			NM_001206313	1.333890279	2.262519145	-1.696180849
12779554	NKAIN1	618218	XM_002685664	1.331036377	1.250053638	1.064783411
12774370	ECE1	281133	NM_181009	1.330455263	1.668683102	-1.254219626
12725874	E2F5	539427	XM_001789547	1.329127956	1.463324774	-1.100966064
12827605	LYST	281072	NM_174020	1.326330209	1.121710203	1.182417888
12869915	CXCL2	281214 // 281734	NM_174299	1.321136927	1.800465556	-1.362815253
12798602	GXYLT2	507299	XM_002696971	1.320514369	-1.196300643	1.579732189
12759305	B9D2	616609	NM_001040593	1.319325001	-1.574146704	2.076811101
12880668	CNN1	534583	NM_001046379	1.314268461	1.092739867	1.202727658
12708347	NOTCH1	767866	XM_010810569	1.312156682	1.529298468	-1.165484647
12710632	N4BP2L1	616069	NM_001046574	1.311865669	-1.209298165	1.586436746

ProbeID	Gene_Symbol	Gene_ID	mRNA Accession	CM12H/CM0H.fc	CM12R/CM0H.fc	CM12H/CM12R.fc
12693124	LOC100298610	100298610	XM_002690554	1.310702261	-1.316420137	1.725434849
12822938	MGEA5	538561	NM_001206448	1.306910187	1.144327498	1.142077062
12855532	FAM126A	540584	XM_002686713	1.306792428	1.057325806	1.235941107
12702463	MEIS1	613877	NM_001083507	1.30557922	1.374560112	-1.05283547
12905544	MTMR1	541155	NM_001206067	1.3021088	1.26323233	1.030775391
12702977	TMEM182	618298	NM_001083784	1.301594446	-1.599332605	2.081682436
12718839	RBM39	508722	NM_001206504	1.301125388	-1.226272833	1.595534717
12826575	ZNF25	101905074	XM_005193104	1.300277905	1.022924736	1.271137415
12852132	LOC101906257	101906257	XR_805947	1.298863659	1.151145344	1.128322906
12686567	TIPARP	540975	NM_001206048	1.298827648	1.107203186	1.173070728
12906346	ZNF75D	100125581 // 614260	XM_003585582	1.296219461	1.304954948	-1.006739203
12822991	RAB11FIP2	540709	NM_001192771	1.295500884	1.527328085	-1.178947929
12895145	SMARCA2	540904	NM_001099115	1.286026169	1.321759779	-1.027786067
12879171	FSTL3	515367	NM_001075710	1.285135072	2.053236767	-1.597681684
12861583	CALCOCO1	538675	NM_001046435	1.284743186	1.638754793	-1.275550485
12678807	SLCO2A1	282468	NM_174829	1.284378126	1.326578455	-1.032856624
12694122	DUT	507945	XM_005211801	1.283586036	-1.037534996	1.331765433
12725603	SPAG1	530104	XM_010812192	1.278719651	-1.115932682	1.42696505
12731183	SMPD1	505097	NM_001075187	1.2779842	1.12311838	1.137889133
12809844	METTL4	521222	NM_001205883	1.277762761	-1.627638149	2.079735416
12865340	TSPAN31	510619	NM_001037595	1.276337616	-1.004508659	1.282092186
12853718	SNX10	508836	NM_001075375	1.275798069	-1.211916246	1.546160407
12823470	NRG1	281361	NM_174128	1.273695132	1.287310434	-1.010689608
12869844	KIT	280832	NM_001166484	1.273368517	-1.001519145	1.275302948
12849921	NUB1	511298	NM_001080906	1.27176314	1.500090978	-1.179536449
12879860	RASGRF2	524957	XM_002689373	1.268707944	1.793564962	-1.413694121
12881001	MKNK2	538519	NM_001205275	1.265344304	1.126018086	1.123733553
12686922	GBE1	615745	NM_001122729	1.263153528	-1.226970021	1.549851511
12725842	OXR1	539092 // 782927	NM_001076148	1.257039514	1.156944774	1.086516437
12708063	NFU1	615964	NM_001046566	1.254654381	1.031954956	1.215803435
12811007				1.251388716	1.40898866	-1.125940039
12905959	HMGN5	767875	XM_002699936	1.250001651	1.390294004	-1.112233734
12901148	PLAGL1	539761	NM_001103289	1.249759072	1.313330485	-1.050866934
12708452	LOC781337	781337	XR_809708	1.249594492	1.282910032	-1.02666108
12888606	MIR23A	791041	NR_031347	1.249412614	2.283188542	-1.82740955
12806780	LOC616868	616868	XM_002702885	1.248702673	2.539351309	-2.033591633
12813341	TBC1D24	529002	NM_001046296	1.248131551	1.169968753	1.066807594
12679157	LRRC3	506054	ENSBTAT00000023769	1.246290165	-1.065063908	1.327378673
12746135	TMEM120B	508790	NM_001101908	1.245323014	1.455091625	-1.168445141
12757357	LOC515089	515089	XM_005192763	1.244848349	1.062019312	1.172152272
12899003	SDHAF4	768071	NM_001077128	1.243209988	1.4346114	-1.153957427
12802761	ZFAND3	532641	NM_001102145	1.241091945	1.003158798	1.237183931
12690032	GALNT16	526331	NM_001101127	1.240541501	1.060209961	1.170090404
12892785	SLC1A1	282353	NM_174599	1.239381209	1.371581167	-1.106666098
12813407	HS3ST2	532099	NM_001205994	1.237647095	-1.044150738	1.292290127
12849788	GPNMB	509600	NM_001038065	1.234845031	1.840847353	-1.490751719
12768215	UTP18	505846	NM_001098879	1.233262577	1.261753423	-1.023102011
12714880	KIAA1217	513129	XM_010811217	1.226400338	2.110215006	-1.720657554
12713397	LOC618696	618696	XM_010811733	1.222021781	1.165363476	1.04861857
12797985	PPARG	281993	NM_181024	1.219187486	1.138291454	1.071067943
12897211	AKAP12	513774	XM_010799018	1.2184356	1.149973009	1.059534085
12873825	BOD1L1	508527	NM_001192190	1.218021838	2.003371534	-1.644774725
12792817	TRAPPC6B	521470	NM_001038134	1.216958524	1.392888717	-1.144565479
12892865	FZD3	445419	NM_001192964	1.215121015	-1.032441473	1.25454133
12872713	LOC782855	782855	XR_800937	1.21369002	1.154389434	1.051369654
12703595	LOC100300896	100300896	XM_002691338	1.213412434	1.054793072	1.150379602
12750311	SPINT2	507484	NM_001045971	1.210119901	1.772456229	-1.464694719
12822057	MKI67	513220	XM_002698582	1.208736687	1.466055784	-1.212882673
12782756	GLB1L	532551	NM_001206002	1.204813633	1.163192603	1.035781718
12887334	PWWP2A	540114	XM_010798064	1.203336389	1.318027069	-1.095310572
12779057	NABP1	613474	NM_001098124	1.202936093	1.216756094	-1.011488558
12889602	FAM206A	507081	NM_001045956	1.199031865	1.34616943	-1.12271364
12697687	LOC785050	785050	XM_001253192	1.198657926	-1.424267369	1.707209371
12711493	FRY	507622	NM_001205616	1.198649618	1.377631466	-1.149319572
12836813	IQGAP3	505886	NM_001098880	1.196831457	2.192717571	-1.832102221
12902320	LOC783993	783993	XM_010802316	1.196159686	-1.1920626	1.425897225
12902073			XR_804038	1.195504864	-1.358119098	1.623637988
12866764	LOC782591	782591	XM_001790444	1.195306002	1.14556554	1.043420006
12910785	LOC788379	788379	XR_803524	1.195214868	-1.786294559	2.135005816
12739170	PDPN	509732	NM_001033120	1.193244759	1.753758383	-1.46973902

ProbeID	Gene_Symbol	Gene_ID	mRNA Accession	CM12H/CM0H.fc	CM12R/CM0H.fc	CM12H/CM12R.fc
12804650	BOLA-DQA2	282535	XM_005196597	1.192872625	2.365524331	-1.983048552
12851723	GSTK1	613498	NM_001079616	1.190576233	1.138394029	1.045838438
12766875	LOC100336815	100336815	XR_238798	1.187427969	1.254924004	-1.056842215
12780223	ACVR2A	281598	NM_174227	1.185413177	1.052959539	1.125791765
12904255	ARMCX3	516747	NM_001192453	1.183081942	1.001158226	1.181713251
12860348	NELL2	524622	NM_001102084	1.18266379	1.286980327	-1.088204727
12868324	DESI1	614655	XM_002687946	1.181557632	1.225083428	-1.036837641
12799597	SEMA3F	531141	XM_002697033	1.17972451	1.173021942	1.005713932
12718246	PFKFB3	407183	NM_001077837	1.179446517	1.078487907	1.093611258
12691926	CDKN3	615282	NM_001040582	1.178498563	2.100728909	-1.782546856
12844440	KDM4A	512622	NM_001206316	1.178327033	1.127126936	1.045425315
12805748	BOLA	507917 // 533049 // 505676 // 515712 // 751813	NM_001038518	1.177992211	1.33062127	-1.12956712
12849526	STEAP2	504616	NM_001077847	1.175390383	-1.14070046	1.340768351
12755342	LOC101907883	101907883	XM_010814968	1.175219305	1.118813836	1.05041542
12902223	TSC22D3	100125309	BC148966	1.175162284	-1.01969663	1.198309021
12894704	ABCA1	535379	NM_001024693	1.174266609	2.51460187	-2.141423293
12718222	LOC404103	404103	NM_205786	1.174063142	1.316630022	-1.121430334
12892568	OMD	280885	ENSBTAT00000015704	1.172908117	1.280990698	-1.092149232
12825460	THAP1	538615	NM_001034648	1.172883727	-1.292478247	1.515926704
12898939	LYRM2	615821	NM_001113310	1.172054779	1.428706923	-1.218976235
12903973	TCEAL4	513987	XM_002699844	1.166777928	1.368864836	-1.173200833
12794992	NPRL2	508487	NM_001015547	1.164459126	1.188119542	-1.020318804
12737971			GENSCAN00000023254	1.161951619	-1.071944342	1.245547465
12899908	IFNGR1	508619	NM_001035063	1.161806656	-1.026967126	1.193137242
12813668	CALN1	538163	NM_001206115	1.159304861	1.341177328	-1.156880621
12895040	LZTS1	539634	NM_001192287	1.159160228	1.161871082	-1.002338636
12762966	GLOD4	512334	NM_001101953	1.159152193	1.308714134	-1.12902701
12696574	TGFB3	538957	NM_001101183	1.156792417	1.043514032	1.108554731
12762696	IFI35	510697	NM_001075462	1.15653586	1.110854604	1.041122624
12906223	MXRA5	786844	XM_001254409	1.155558261	-1.049789447	1.213092868
12838632	LAMTOR5	516090	NM_001034517	1.155550252	1.3393472	-1.159055781
12827782	SLC25A16	282524	NM_174672	1.153709497	1.354274321	-1.173843437
12910441	LOC781489	781489	XM_002700317	1.153373676	-1.064842457	1.228161258
12727890	TAF10	514443	NM_001098968	1.153245769	1.1346049	1.016429392
12769776	SLC43A2	511955	NM_001075546	1.153221789	1.398780898	-1.212933117
12834028	LOC784113	784113	XM_001252523	1.153173828	1.284208987	-1.113630014
12864062	KITLG	281885	NM_174375	1.152750267	-1.122736986	1.294235361
12820005	ZRANB1	523338	NM_001101114	1.151560334	1.210287671	-1.050998055
12825048	RWDD4	509865	NM_001076824	1.148817794	-1.089842759	1.252030754
12766526	LOC100850190	100850190	XM_003585325	1.146280404	1.089804989	1.051821579
12683252	GYG1	280812	NM_001045867	1.146177118	-1.185709014	1.35903254
12889619	FAM160B2	507256	XM_002689765	1.145073337	1.189297791	-1.038621503
12899918	SMPD2	509018	NM_001075383	1.143859613	1.606632538	-1.404571435
12825915	VDAC2	282120	NM_174486	1.142227481	-1.182958941	1.351208211
12694383	ABHD4	509896	NM_001034368	1.140763716	1.699181543	-1.489512262
12852485			GENSCAN00000019568	1.138812315	1.05602208	1.078398205
12901720			GENSCAN00000014068	1.138039001	-1.176792535	1.339235801
12802401	HIST1H2BJ	522960	XM_002697500	1.137731399	8.451222221	-7.428134818
12855202	TAX1BP1	535589	NM_001046409	1.137502724	1.254576114	-1.102921415
12777335	DLX2	528490	NM_001192662	1.136919415	1.537375945	-1.352229475
12682652	TMSB4	781334	ENSBTAT00000007124	1.136793334	-1.406607681	1.599022235
12792596	CKB	516210	NM_001015613	1.136218265	-1.128393296	1.282101073
12846597	LRRC52	536513	NM_001206099	1.135171284	1.194063864	-1.051879906
12787220	DNAJC21	509302	NM_001192218	1.134046659	1.510148057	-1.331645435
12678716	SYNJ1	282087	NM_174468	1.133968056	1.621939493	-1.430322031
12849736	MPLKIP	509064	NM_001075389	1.132876033	-1.02511801	1.161331624
12712472	TGDS	534594	NM_001101159	1.129551461	1.010934832	1.117333606
12886461	UPF1	532933	XM_001789911	1.129418368	1.347485735	-1.193079352
12765844	TRAF4	781568 // 783305	NM_001101280	1.128635786	1.317241619	-1.167109562
12829973	SLC3A2	507107	NM_001024488	1.128393296	1.088001089	1.037125154
12769692	MED11	511672	NM_001038084	1.127947563	2.185085906	-1.937222951
12723793	SLC10A5	104968462	XM_010812265	1.127064437	1.650656582	-1.464562742
12780541	IGFBP5	404185	NM_001105327	1.126791042	1.329533381	-1.179928958
12825130	VPS37A	513985	NM_001046161	1.126736372	1.218672098	-1.08159471
12854729	LOC524050	524050	XM_602368	1.126119555	1.184008961	-1.051406093
12834738	ZBTB44	540532	XM_002699251	1.125284658	1.209449054	-1.074793871
12885658	PRDM6	519857	NM_001103255	1.124247753	-1.177437107	1.323731021
12779112	TAF12	614227	NM_001034754	1.123788078	1.061239294	1.058939378
12770228	INPP5K	514123	NM_001101986	1.123258516	1.067650906	1.052084076
12809364	MPPE1	506830	NM_001305022	1.122573572	-1.159762986	1.301919277

ProbeID	Gene_Symbol	Gene_ID	mRNA Accession	CM12H/CM0H.fc	CM12R/CM0H.fc	CM12H/CM12R.fc
12862594			ENSBTAT00000037671	1.120521243	1.145779952	-1.022541928
12896090	C9H6orf120	100125289	NM_001103331	1.120334854	1.266801078	-1.130734328
12897014	FRK	509227	XM_002690084	1.120296027	1.152223031	-1.028498721
12795056	PDZRN3	509083	XM_002696956	1.119729303	1.475403947	-1.317643418
12871228	AGPAT9	519739	NM_001192514	1.119411132	-1.080440781	1.209457438
12751361	APLP1	513154	NM_001038095	1.11829437	-1.057985606	1.183139347
12792605	STRN3	516375	NM_001098042	1.11747302	1.217532261	-1.089540632
12788177	BOD1	540063	NM_001076200	1.117039343	1.246782664	-1.116149285
12779277	PTMA	615626 // 786336	NM_001039953	1.11609513	-1.393091482	1.554822618
12719526	ATP9A	516579	XM_003583005	1.115777992	1.363618427	-1.22212343
12871178	NUDT9	517589	NM_001101096	1.115314049	1.120653287	-1.004787206
12891530	FAM122A	539712	NM_001101196	1.115267665	1.239999896	-1.111840623
12711664	INTS6	509695	XM_010799168	1.112318541	1.48546025	-1.335462995
12812425	SNRNP25	513526	NM_001166612	1.111024014	-1.047528866	1.163829726
12862676	TAS2R10	664636	NM_001046626	1.110115664	-1.197146739	1.328971347
12678842	ADAMTS5	286805	NM_001166515	1.109092736	1.401070935	-1.263258599
12910377	BEX2	767968 // 768028	NM_001077087	1.107556272	1.039269631	1.065706375
12738390	LZIC	767886	BC151733	1.107111096	-1.809398274	2.003204905
12708443	YPEL5	780856	NM_001079793	1.107103422	1.090726961	1.015014263
12775752	CHN1	508266	NM_001075349	1.106919265	1.760713286	-1.590642915
12768248	PRPSAP2	506111	NM_001081513	1.106627745	2.104182736	-1.901436817
12909762	CAPN6	539360	NM_001192231	1.104022826	-1.109269566	1.22465892
12754903	LOC789960	789960	XM_005219828	1.103808576	1.233330965	-1.11734135
12869909	GRO1	281212	NM_175700	1.100546422	1.509185349	-1.371305489
12868425	C5H12orf5	615392	NM_001076370	1.099463721	1.041050461	1.056109921
12843090	NIT1	504199	NM_001038023	1.098732358	1.485264631	-1.351798389
12692985			GENSCAN00000032237	1.097735136	-1.237303994	1.358232068
12690836	RORA	535597	NM_001192861	1.09727109	-1.040083965	1.141254066
12833412	AQP11	510038	NM_001110069	1.095819362	1.371172424	-1.251275959
12786378	MSX2	540230	NM_001079614	1.095333349	-1.015640619	1.112465041
12812568	CTF1	514803	NM_001192384	1.091642146	1.115971358	-1.022286802
12907694	PIM2	508424	NM_001206378	1.091105043	2.067704469	-1.895055368
12727811	LOC514057	514057	XM_001787715	1.090817688	-1.114016036	1.215188397
12772798	SRSF1	615796	NM_001076394	1.085756056	-1.216368196	1.320679135
12904823			ENSBTAT00000064281	1.084620242	-1.351920204	1.466320018
12904630	HTATSF1	526910	NM_001206414	1.084379692	-1.376266633	1.492395588
12870826	C6H4orf19	511424	NM_001104973	1.083327914	1.176988317	-1.086456189
12844912	PPOX	515770	NM_001192426	1.081047564	1.049382037	1.030175404
12787557	SH3PXD2B	518356	XM_010816700	1.080867741	1.203711788	-1.113653172
12710196	RB1	534712	NM_001076907	1.079400305	1.184780663	-1.097628617
12717355	LOC782950	782950	XR_083823	1.078547713	-1.355138211	1.461581218
12808543	ABHD3	539795	NM_001076187	1.078271139	-1.189916221	1.283052319
12804265	MIR877	100313087	NR_031221	1.077561342	1.17385971	-1.089366947
12752457	PVR	526865	XM_005195348	1.075889565	1.337065369	-1.242753357
12722742	KLF10	522795	NM_001168462	1.075747882	1.38913807	-1.291323082
12828057	ERO1LB	514361	NM_001206730	1.073937474	1.120917423	-1.043745516
12847968	LOC782233	782233	XR_805429	1.072375378	1.034282291	1.036830455
12771139	LOC524719	524719	XM_010824247	1.069384003	1.348550922	-1.261053952
12889102	ATP6V1G1	281641	NM_174245	1.068628204	-1.001130468	1.069836255
12789065	IGF1R	281848	NM_001244612	1.06836158	-1.08071042	1.154589492
12842040	MIR186	791032	NR_031376	1.067110814	1.273271431	-1.193195135
12854683	SOSTDC1	523184	NM_001046265	1.06583196	1.01450783	1.050590177
12896338	CNR1	100299449	NM_001242341	1.065625123	-2.091430418	2.228680795
12727546	HBG	511735	NM_001014902	1.064879362	-1.515853153	1.614200738
12738945	TRAF5	507234	NM_001105340	1.064857219	1.268980588	-1.191690835
12859746	CCDC134	516012	NM_001205826	1.06386862	2.041231768	-1.918687824
12846895	TCEANC2	539385	NM_001104992	1.063146197	1.142409594	-1.074555501
12875352	ANK2	539238	XM_010805941	1.062578922	1.771178973	-1.66686816
12848065				1.061982505	-1.334963225	1.417707591
12891999	LOC104968438	104968438	XM_003586368	1.061342283	-1.073550457	1.139404493
12728123	SESN3	515914	XM_002692957	1.059600184	1.138757061	-1.074704476
12885908	ZNF354A	524106	NM_001256565	1.059438616	1.038240017	1.020417821
12901612	LOC100850276	100850276	XM_005211029	1.059181626	1.396359095	-1.318337725
12832994	LOC504490	504490	XM_002699110	1.057692311	1.026981362	1.029904095
12877198	VCAN	282662	NM_181035	1.057333135	1.104198847	-1.044324452
12893495	HPGD	512259	NM_001034419	1.056834889	-1.336083339	1.412019488
12831768	LOC613739	613739	XM_002699259	1.056776287	-1.18872912	1.256220746
12902364	LOC100295153	100295153	XR_816739	1.056175807	1.944811056	-1.841370578
12758513	RHPN2	533687	NM_001083470	1.055546401	1.038383958	1.016528032
12781058	SESN2	509863	NM_001024507	1.055495187	1.398790594	-1.325245829

ProbeID	Gene_Symbol	Gene_ID	mRNA Accession	CM12H/CM0H.fc	CM12R/CM0H.fc	CM12H/CM12R.fc
12718301	BEND7	504404	XM_005214313	1.053762686	-1.05505631	1.111778972
12842414	LOC531090	531090	XM_010803765	1.053178518	1.07505465	-1.020771532
12883575	ST8SIA4	407768	NM_001001163	1.052857364	-1.215003104	1.279224965
12901609	LOC788043	788043	XR_802924	1.05280628	1.036837641	1.015401291
12842026	SCLY	790815	NM_001083804	1.051529992	-1.358721713	1.428736632
12805318	CYP39A1	511195	NM_001098938	1.051435244	1.178474058	-1.120824192
12887830	ADAMTSL5	100337439	XM_010800165	1.050298932	1.473645999	-1.40307293
12792547	CCDC88C	515039	NM_001102017	1.049971377	-1.06434805	1.117534988
12725230	CA13	513850	XM_002692829	1.049731236	1.220811113	-1.162974932
12818608	DNAH3	786654	NM_001099199	1.046781259	-1.057523703	1.106995993
12856483	MIR2284H	100313467	NR_031257	1.046316995	-1.059335812	1.108401063
12780953	ZNF593	508477	NM_001192186	1.046186457	1.164628638	-1.113213261
12698356	ASS1	280726	NM_173892	1.04516448	1.239114925	-1.185569304
12836852	ARHGEF2	505940	NM_001098881	1.043940871	1.41558659	-1.356002652
12693774	TRD@	407199	BC113229	1.043181363	-1.579743139	1.6479586
12910177	MID1IP1	615572	NM_001076383	1.043181363	1.005135499	1.037851477
12734027	LOC790683	790683	XM_002693137	1.041043245	1.32163152	-1.269526051
12690804	ATL1	535424	NM_001034631	1.040754647	1.123975041	-1.079961588
12775056	HTR1D	407136	XM_005197607	1.039665909	-1.193716296	1.241066138
12840828	PYGO2	540401	NM_001102242	1.03934167	1.48498669	-1.428776246
12842868	DNAJC6	317659	NM_174836	1.037729189	1.060364297	-1.021812153
12681285	MECOM	532209	XM_003581740	1.035494578	1.137321393	-1.098336406
12836019	PTGER3	282330	NM_181032	1.0339884	-1.18389407	1.224132734
12825725			ENSBTAT00000025237	1.032734924	-1.223996981	1.26406443
12699109	MRPL53	505728	NM_001045926	1.031125546	1.310239003	-1.270688141
12885154	USE1	512890	NM_001075584	1.03089686	1.134227468	-1.100233701
12836290	CRABP2	493998	NM_001008670	1.030632505	2.176801718	-2.112102719
12879073	PCYOX1L	514598	NM_001075673	1.02976133	1.29440582	-1.256995949
12829596	CTSC	352958	NM_001033617	1.029411639	-1.255855086	1.292791843
12862774	GLIPR1	767905	NM_001076984	1.029076333	1.011909312	1.01696498
12872081	BMP3	539527	NM_001192268	1.029005005	1.600852071	-1.555728167
12703398			GENSCAN00000012309	1.028691222	-1.123897136	1.156143119
12684439	NUDT16	512320	NM_001075560	1.028163712	-1.029739917	1.058741216
12756629	ETHE1	509150	NM_001034344	1.027793191	1.244080638	-1.210438684
12720915	GGT7	615929	NM_001076401	1.027707705	-1.224064856	1.257980884
12712593	KBTBD6	538461	NM_001205540	1.027137981	1.525169938	-1.48487347
12774650	PRKRA	282875	NM_001045870	1.024606536	-1.54397565	1.581967542
12707871	SIRT3	614027	NM_001206669	1.023768839	-1.014866527	1.038988726
12892272	MIR27B	791000	NR_030910	1.023328968	2.035312046	-1.988912765
12786416	ADAMTS6	540722	NM_001193016	1.02240727	1.468272758	-1.436093817
12704122	ATP6V1B1	338059	NM_176654	1.021507644	-1.006341525	1.027985561
12811207				1.020792759	-1.744519834	1.780793214
12739704	KMO	515996	NM_001243298	1.019965249	-1.163499024	1.186728572
12856205	TPK1	788066	NM_001080370	1.01995111	1.469066802	-1.440330608
12782544	ARHGEF10L	529043	NM_001046297	1.019428082	1.114973947	-1.093724969
12785993	LOC530348	530348	XM_002696335	1.019364488	1.317469899	-1.292442413
12760429	ABR	515556	XM_005220104	1.017931161	-1.268321067	1.291063536
12682788	LOC783884	783884	XM_001252341	1.016253274	-1.084657832	1.102287073
12698826	IL18R1	407221	XM_005193845	1.015598381	1.213891941	-1.195248007
12906798			GENSCAN00000043039	1.014381261	1.100058312	-1.084462375
12691723	ISCA2	613290	NM_001038683	1.014353137	-1.189198872	1.206267607
12901286	SLC16A10	541240	NM_001192847	1.013727575	1.324024667	-1.306095148
12787924	FAM105A	534389	NM_001102171	1.012758364	1.068724502	-1.055261097
12760005	LOC100848201	100848201	XM_010827389	1.010080307	1.090757202	-1.079871763
12786245	CPEB4	538794	XM_003583671	1.009548345	1.064333295	-1.054266792
12901705			GENSCAN00000022835	1.009198523	1.063669536	-1.053974527
12835544	LOC101903478	101903478	XM_010803745	1.009156552	-1.534458896	1.548509249
12692693	TYRO3	788224	NM_001191228	1.008932739	-1.005128532	1.014107083
12807083	LOC785639	785639	XM_001790501	1.008660034	-1.17337975	1.183541259
12825721	MIR2403	100313193	NR_031302	1.00858313	-1.26218204	1.273015513
12703615	MIR2295	100313460	NR_031092	1.007521063	-1.563121584	1.574877921
12910512	ZXDB	783265	XM_005228277	1.005713932	-1.165169627	1.171827328
12907723	SAT1	508861	NM_001034333	1.005609372	1.054252176	-1.048371471
12809579	FAM210A	511554	NM_001080259	1.00554664	-1.062549461	1.068443041
12694950	LOC515823	515823	XR_082839	1.004933475	-1.028170839	1.033243294
12684414	DPH3	511579	NM_001113299	1.004905612	1.173095121	-1.167368464
12762788	MBTD1	511415	NM_001206222	1.004334606	1.018354594	-1.01395948
12746496	C17H4orf33	513251	XM_003587131	1.004258032	1.39276321	-1.386857925
12867326	XPOT	535264	NM_001206076	1.002804239	1.000214899	1.002588784
12899085	LOC782807	782807	XM_001253329	1.002672181	-1.067081227	1.069932661

ProbeID	Gene_Symbol	Gene_ID	mRNA Accession	CM12H/CM0H.fc	CM12R/CM0H.fc	CM12H/CM12R.fc
12689424	TMEM62	515078	NM_001205497	1.002505395	1.490049233	-1.486325402
12712812	SPRYD7	615298	NM_001038206	1.00162328	1.12756453	-1.125737142
12678550	SST	280932	NM_173960	1.001463611	-1.145795836	1.147472835
12896371	LAMA4	529670	NM_001205965	1.001394197	1.103976911	-1.102439893
12850876	SLC26A4	530241	XM_002686803	1.001234563	-1.13492739	1.13632853
12861991	LIMA1	540637	NM_001192754	1.000825185	1.286445198	-1.285384517
12683292	ECE2	281134	NM_001314064	1	-1.077374631	1.077374631
12852487			GENSCAN00000032320	-1.000402106	-1.118488172	1.118038602
12793718	TM6SF1	616003	NM_001102295	-1.000651771	-1.177796262	1.177029109
12907669	MOSPD2	508293	NM_001101896	-1.000873747	1.083891239	-1.084838286
12892238	MAMDC2	788176	XM_002689639	-1.00569999	1.328648976	-1.336222262
12726242	PLEKHF2	782597	NM_001098150	-1.00633455	-1.114278607	1.107264584
12857056	MFAP5	281908	NM_174386	-1.007625823	-1.376524225	1.366106539
12697937			NM_001105489	-1.007800446	-1.130279835	1.12153139
12739104	AGTRAP	508521	NM_001075363	-1.009422395	1.867735291	-1.885333831
12705362	CYP1B1	511470	NM_001192294	-1.010948847	1.075583851	-1.087360254
12741511	SYT14	787880	XM_002694296	-1.011719951	1.300286918	-1.315526217
12787307	GPX8	511575	NM_001046088	-1.01313751	-1.582834043	1.562309191
12710821	SCEL	784362	NM_001102332	-1.01395948	1.131541894	-1.14733763
12730248	LOC783497	783497	XM_002692947	-1.014121142	1.350608929	-1.369681069
12857869	FAM118A	505415	NM_001038035	-1.019421015	2.130762802	-2.172144379
12690776	C10H15orf61	535251	XM_002690478	-1.021620939	-1.147122927	1.122845943
12697035			GENSCAN00000022865	-1.021953816	-1.155157845	1.130342513
12906380	F8A1	101902099	XM_005227653	-1.021989235	1.009996295	-1.03220534
12801086			ENSBTAT00000007026	-1.022130923	1.221225822	-1.248252676
12852253	TMEM60	767911	NM_001076988	-1.023839804	1.131236049	-1.158204494
12841663	LOC782190	782190	XM_001250777	-1.024826723	-1.374922213	1.341614327
12684477	U2AF1	512680	NM_001080268	-1.026276873	-1.004738455	-1.021436841
12864664	ARSA	505514	NM_001075205	-1.026433385	1.189454429	-1.220895736
12721874	OPLAH	408006	NM_001001173	-1.02734447	-1.15052314	1.119900066
12679281	C1H3orf33	507040	XM_002684988	-1.027636473	1.199555576	-1.232707061
12799059	IRAK2	515034	NM_001075696	-1.02787156	1.035042495	-1.063890743
12767646	CYB561	317663	NM_174837	-1.030653937	-1.296992377	1.258416943
12685960	MME	536741	NM_001192884	-1.030803971	-1.03847753	1.007444246
12816951	CYP3A24	517246	XM_005225269	-1.031625972	-1.096222005	1.062615749
12729981	LOC616011	616011	XR_811737	-1.032863783	-1.260206364	1.220108967
12814986	EARS2	100300732	XM_002698024	-1.03303562	1.032827987	-1.0669481
12812085	GNPTG	508713	NM_001017428	-1.033845068	1.097415608	-1.134557714
12896970	TNFAIP3	508105	NM_001192170	-1.034210602	2.16089654	-2.234822112
12738131	MIR2284N	100313140	NR_030870	-1.034353984	-2.038983358	1.971262632
12698798	ST3GAL5	404164	NM_205807	-1.03478425	2.099346056	-2.172370233
12892284	MIR23B	791065	NR_030945	-1.038383958	1.204821984	-1.25106782
12712820	PARP4	615359	XM_002691868	-1.038607105	1.575401987	-1.636223697
12681492	CCDC39	534432	NM_001206056	-1.040747433	-1.125932235	1.081849639
12753926	CEBPG	617530	NM_001034801	-1.041100975	-1.187716076	1.140826975
12872147	THAP6	539787	NM_001103290	-1.04134636	-1.000936187	-1.040372377
12739733	KIFAP3	518402	NM_001035329	-1.04143298	1.053280724	-1.096921284
12821440			ENSBTAT00000027838	-1.041476293	-1.604473542	1.540576154
12791646	WARS	281576	NM_174218	-1.042718695	-1.019194926	-1.023080737
12755423				-1.043499566	-1.055107503	1.011124046
12805924	LRFN2	524447	NM_001192595	-1.044100076	1.063433633	-1.110331137
12716894			NM_001076302	-1.045613737	1.101187394	-1.151416666
12864984	PMCH	508013	NM_001080240	-1.046723214	1.140431663	-1.193716296
12844669	LOC513884	513884	XM_002685947	-1.046948154	-1.235324445	1.179928958
12835571	MIR2887-1	100498812	NR_036352	-1.046962668	-1.316182915	1.257144076
12814684	NME4	789324	NM_001077906	-1.047078786	-1.092270362	1.043159671
12732436	NCR3LG1	523303	NM_001206792	-1.047608739	1.004891682	-1.052733308
12744054	PCDH18	539464	NM_001102229	-1.051668486	-1.166373624	1.109069673
12812393	PHKG2	512670	NM_001046128	-1.052127832	1.529552896	-1.609285172
12879187	STXBP2	515618	NM_001046208	-1.052689527	1.324612153	-1.39440534
12848001	ST6GALNAC3	782689	XM_003581999	-1.052944942	-1.186555843	1.126892581
12786203	HCN1	538255	NM_001206580	-1.053434052	1.341214514	-1.412881039
12678456	VEPH1	100337421	XM_005201776	-1.05383573	-1.229677501	1.166858806
12683046	CD200R1L	100335828 // 516008	XM_002684750	-1.055831783	-1.087367791	1.029868402
12784678	LOC785151	785151	XR_802935	-1.056131883	1.384975063	-1.46271632
12727383	FAM111B	509351	NM_001205654	-1.056930124	-1.281488027	1.212462393
12736315	PLA2G4A	525072	NM_001075864	-1.057780291	-1.183647912	1.118992216
12815026	MIR1225	100313098	NR_031237	-1.058051609	-1.295716415	1.224624966
12803613	EEF1E1	617105	NM_001040599	-1.05835234	-1.342944795	1.268901427
12832191	E2F8	786629	NM_001191234	-1.060511305	1.262540791	-1.338938782

ProbeID	Gene_Symbol	Gene_ID	mRNA Accession	CM12H/CM0H.fc	CM12R/CM0H.fc	CM12H/CM12R.fc
12811895	IQCK	506290	NM_001192088	-1.060650981	2.453906024	-2.602737833
12768342	ITGB4	506995	NM_001193257	-1.062144462	1.002283056	-1.064569398
12682849			GENSCAN00000007056	-1.06293988	1.041700105	-1.107264584
12761288	CACNB1	327703	NM_175819	-1.063109351	1.111671089	-1.18182793
12908490	RENBP	516214	NM_001046223	-1.064746509	-1.155253932	1.085003728
12812034	LYRM1	508417	NM_001205637	-1.066016671	-1.087616543	1.020262227
12709043	POSTN	281960	NM_001040479	-1.06719218	-1.349588888	1.264616545
12777810	RHBDD1	533985	NM_001077995	-1.067887744	-1.044809558	-1.022088414
12699877	REG3G	513652	NM_001024534	-1.069102368	1.11853469	-1.195828086
12870968	PYURF	100996942	NM_001038102	-1.070414823	1.090968918	-1.167789302
12904911	HNRNPH2	534001	NM_001076008	-1.070748755	-1.025331199	-1.044295498
12871379	OTOP1	525643	NM_001206784	-1.071067943	1.891538315	-2.025966052
12750986	AARS	510933	NM_001101075	-1.071119913	1.001199864	-1.072405111
12837225	SLAMF9	507627	NM_001302662	-1.071825467	1.21029606	-1.29722614
12724014	MIR2311	100313132	NR_031164	-1.07204837	-1.439651882	1.342898252
12899131	PLN	100125240	NM_001103319	-1.073632315	1.430917009	-1.53627874
12896057	TMEM246	786832	NM_001102342	-1.074413993	-1.003548262	-1.07061517
12688778	PSME2	509857	NM_001014889	-1.075308038	1.217236922	-1.308904646
12857834	APOL3	505078 // 512905	NM_001100297	-1.076732593	-1.650359131	1.532747445
12895699	SLC25A51	781425	NM_001099394	-1.077718205	-1.008681009	-1.068443041
12853428	CYP51A1	505060	NM_001025319	-1.07823377	-1.205072546	1.117635692
12744806	ZNF140	785125	XM_002694443	-1.078293561	1.006090441	-1.084860845
12776426			ENSBTAT00000038024	-1.08022362	-2.100000978	1.944042825
12871600	UNC5C	533256	XM_002688134	-1.081212428	1.023570164	-1.106696782
12902925	CITED1	282182	NM_174518	-1.083132696	-1.06703685	-1.015084621
12687438	PPP2R3A	787607	XM_005195983	-1.083245317	1.133072362	-1.22739533
12718453	PLTP	505640	NM_001035027	-1.083297878	-1.216823567	1.123258516
12857324	PLBD1	317710	NM_001101044	-1.083335423	1.252977059	-1.357394432
12801369	NEU1	505554	NM_001083642	-1.084507477	1.448058613	-1.570430394
12842719	TUFT1	282104	NM_174479	-1.084928524	1.947752005	-2.113171708
12729417	C15H11orf1	538766	NM_001038177	-1.086569156	1.01395948	-1.101737097
12841009	FAM69A	541171	NM_001083500	-1.087262278	-1.037441509	-1.048022725
12897007	HEBP2	509223	NM_001192213	-1.08739794	-1.030153982	-1.055568351
12727554	LOC511823	511823	XM_002693233	-1.08773717	1.02129525	-1.110900804
12743278	ANKRD50	527956	NM_001205949	-1.087812569	-1.364091103	1.253976229
12718157	EIF6	286811	NM_174830	-1.088136843	1.144160941	-1.245003674
12700517	IL1RL1	520709	NM_001206302	-1.089057404	2.361085033	-2.571357136
12865053	KCNA5	508960	NM_001015552	-1.089321643	-1.019880415	-1.068087618
12688871	LOC510673	510673	XR_802492	-1.092300646	2.015810469	-2.201871078
12682512	FAM162A	617104	ENSBTAT00000014796	-1.092399077	1.103280782	-1.205222908
12878366	TSPAN17	509386	NM_001014880	-1.094164763	-1.036543024	-1.055590301
12831992			ENSBTAT00000030370	-1.094248192	-1.315334742	1.202044245
12869954	CENPE	281681	XM_010805938	-1.094756488	1.878290226	-2.056270412
12687084	PIGP	767951	NM_001077022	-1.096153621	-1.132475626	1.033135871
12743116	LOC101909508	101909508	XM_005193460	-1.096799638	1.206652284	-1.323455788
12842647	SARS	281476	NM_174175	-1.097948207	-1.055027058	-1.04068251
12825103	BRF2	512789	NM_001015582	-1.098770437	1.075501845	-1.181729633
12740247	FLVCR1	533317	NM_001206019	-1.099433238	1.298755627	-1.427895104
12726994	LOC504551	504551	XM_001787386	-1.100439629	1.871117295	-2.059051622
12764250	AKAP1	532072	XM_002695587	-1.100500652	1.141974155	-1.256743302
12875211	SGCB	535372	NM_001102188	-1.101278992	-1.005414221	-1.095348533
12829623	NOX4	378474	NM_001304775	-1.10175237	-1.024876449	-1.07500994
12876963	ICAM1	281839	ENSBTAT00000013608	-1.102439893	2.070960447	-2.283109414
12894613	ECM2	533916	NM_001034597	-1.102860258	-1.221640672	1.107702144
12785247	SEMA5A	506636	XM_002696441	-1.103724418	-1.218596076	1.104076394
12832069	ASRGL1	767970	NM_001077035	-1.104573943	1.305045403	-1.441519147
12835631	LOC101904240	101904240	XR_234021	-1.104841947	-2.474951064	2.240095129
12699294	KRCC1	507166	NM_001075303	-1.106029603	1.01870759	-1.126720752
12845896	OR10J1	530601	XM_002685875	-1.106781167	-1.043268136	-1.060878914
12681463	NCEH1	534212	NM_001123034	-1.107356688	1.215415841	-1.34589886
12699923	EPCAM	514039	NM_001035290	-1.108769902	1.059732395	-1.174999384
12767419	VAMP2	282116	NM_174483	-1.110015637	1.202911079	-1.335250107
12726511	LOC100336916	100336916	XM_002693681	-1.110608236	-1.033551301	-1.074555501
12696080	GALC	533428	NM_001206020	-1.111031715	1.269156518	-1.410073143
12905202	RAB39B	537560	NM_001076095	-1.113290426	-1.042675331	-1.067724912
12848486	LOC100295719	100295719	XM_002686921	-1.113514234	2.087679143	-2.324660441
12759367	ETFB	617210	NM_001038582	-1.114340398	-1.076568412	-1.035085542
12681995	FBXO25	540328	NM_001076211	-1.115020319	-1.070429663	-1.041656783
12848622	MIR196B	100313452	NR_030829	-1.115785726	1.18170506	-1.318529637
12866165	CKAP4	515784	XM_002687658	-1.115994564	-1.222487743	1.09542446

ProbeID	Gene_Symbol	Gene_ID	mRNA Accession	CM12H/CM0H.fc	CM12R/CM0H.fc	CM12H/CM12R.fc
12752612	WDR62	530449	XM_002694947	-1.116791604	1.882852519	-2.102753884
12870010	CXCL5	281735	NM_174300	-1.11721744	1.049898601	-1.172965028
12693035			ENSBTAT00000064250	-1.117263905	-1.009912289	-1.10629796
12900957	TPD52L1	534629	NM_001076033	-1.118635484	-1.213504956	1.084808208
12731429	LOC507428	507428	XM_002693191	-1.119822444	-1.142219564	1.020000599
12855561	ETV1	540846	NM_001046492	-1.120327088	-1.232595988	1.100210823
12734319			ENSBTAT00000034028	-1.120785347	-1.182671988	1.05521721
12783701	TYW5	539498	XM_002685484	-1.121018432	-1.157690812	1.032713449
12883735	SPRY4	504593	NM_001081512	-1.121189392	2.000138634	-2.24253422
12806109	CYP39A1	511195	NM_001098938	-1.12220792	-1.05900544	-1.059680978
12791559	LOC100301305	100301305	XM_005221785	-1.122807029	1.087518543	-1.221073464
12875732	LOC615303	615303	XM_002688321	-1.124310096	-1.339700025	1.191575198
12832525	CLCF1	100336481	XM_005193325	-1.124395823	-1.188416054	1.05693745
12719021	ITGA8	511976	XM_002692081	-1.127001941	-1.365898234	1.21197505
12877798	MCOLN1	505738	NM_001075222	-1.12747856	1.849249646	-2.084989329
12890470	RECK	517232	NM_001192465	-1.128729668	1.162733123	-1.312411372
12817119	TEKT5	521940	NM_001046259	-1.128987881	-1.589166184	1.407602518
12709775	LOC522140	522140	XR_810126	-1.130209327	-1.111933108	-1.016436438
12909398	SMARCA1	535439	NM_001191542	-1.130217161	1.931002457	-2.182452115
12844355	LOC512286	512286	XM_003581921	-1.132083208	-1.154877636	1.02013494
12829688	SLC25A22	504371	NM_001075159	-1.134683548	1.04389022	-1.184485058
12681445	NSUN3	533791	NM_001076001	-1.135375881	-1.216823567	1.071736318
12797711	LOC100138407	100138407	XM_010799506	-1.135438842	1.152510584	-1.308605283
12855735	ZC3HAV1	614589	XM_003582128	-1.135588386	-1.135745823	1.000138639
12688364	SLC7A7	504220	NM_001075151	-1.138031113	1.262812109	-1.43711947
12840420	HIST2H2BE	537985	NM_001099384	-1.139286033	2.604650861	-2.967442346
12910691	LOC104968697	104968697	XM_010799727	-1.139933765	-1.036701101	-1.09957804
12875026	AP1AR	533664	NM_001081532	-1.140803252	-1.0648646	-1.071312966
12683322	AGTR1	281607	NM_174233	-1.141649663	1.108147558	-1.265116286
12745970	GATSL3	506974	NM_001081514	-1.14328097	-1.195869531	1.045997933
12853998	SLC26A3	512856	NM_001083676	-1.143447399	-1.06507129	-1.073587664
12875228	LOC536367	536367	XM_002688145	-1.143677269	1.025352521	-1.172672371
12700406	ADD2	518896	NM_001099009	-1.146018235	1.121469201	-1.285224154
12882838	MIR2454	100313227	NR_030812	-1.149032815	-1.282323264	1.1160023
12872629	LOC786899	786899	XM_010799338	-1.151169282	-1.149247876	-1.001671881
12816697	C25H16orf13	514636	NM_001038106	-1.152079281	-1.21550009	1.055048997
12773826	CD302	100126272	NM_001110191	-1.15362953	-1.120428045	-1.029632859
12736229	MXRA8	522392	NM_001075830	-1.154285418	1.417687937	-1.636416512
12872974	MIR2447	100313222	NR_031260	-1.157161316	1.946146074	-2.252004952
12698660	EPAS1	282711	NM_174725	-1.157474171	1.508055997	-1.745535865
12820938	RPP30	615098	NM_001035461	-1.158108161	1.417756725	-1.641915635
12878593	LONP1	510796	NM_001015569	-1.158453391	1.128236879	-1.307009838
12846227	CGN	533725	NM_001192786	-1.159047747	1.208828852	-1.401090358
12871619	NSUN7	533295	XM_002688230	-1.160100667	1.154613501	-1.339467893
12779988	CRYGA	100335856	NM_001243570	-1.160728051	-1.328345097	1.14440682
12692366	VPS13C	783566	XM_001251843	-1.161484579	1.111301286	-1.290759306
12808614	TMEM241	614734	XM_002697719	-1.161540936	1.055363505	-1.225847913
12805255	BOLA-NC1	510417	NM_001105616	-1.1634587	-1.031962109	-1.127423856
12880685	WIZ	535200	XM_003582372	-1.166017952	1.127572345	-1.314769596
12802930	TMEM63B	534891	NM_001163939	-1.166422133	1.748635993	-2.039647724
12760792	SERPINF1	281386	NM_174140	-1.167465567	-1.470116004	1.259237142
12803670	LOC617875	617875	NM_001099724	-1.167546492	1.030025461	-1.202602614
12837217	LOC104968660	104968660	XM_005197920	-1.168671936	1.208611019	-1.412469779
12742553	ACAD10	511425	NM_001192292	-1.172371661	1.14719449	-1.34493831
12873015	LOC100336621	100336621	XM_002688163	-1.17308699	1.063470489	-1.247543395
12846183	DDR2	533523	NM_001083720	-1.174461971	1.00089456	-1.175512598
12829936	SLC37A2	506687	NM_001024486	-1.176580475	1.862873874	-2.191821027
12758642	FAM192A	534394	NM_001014944	-1.177102538	1.270089356	-1.495025405
12887049	NWD1	537999	NM_001102210	-1.178082032	-2	1.697674649
12718608	PPDPF	506990	NM_001075294	-1.179029651	-1.808871595	1.534203651
12734888	MTHFR	497032	NM_001011685	-1.179618211	1.163668396	-1.372684431
12866308	ALDH1L2	516864	NM_001191391	-1.180935361	-2.021953753	1.712162935
12819767	DPCD	516908	NM_001075745	-1.181246455	1.229455911	-1.452290437
12797682	LOC789829	789829	XM_005222618	-1.183721754	-1.11142454	-1.065049143
12716231	UCKL1	534046	XM_002692400	-1.184394749	-1.24196972	1.048611302
12826182	ZNF32	512392	NM_001046119	-1.184788876	-2.056327425	1.735606627
12826569	DDIT4	529235	NM_001075922	-1.184903854	-1.358326217	1.146359861
12848889	NPVF	281451	NM_174168	-1.186095356	-1.049025683	-1.130663791
12680157	PLCH1	513565	XM_010801458	-1.186539394	1.076307266	-1.277080972
12828047	OR5L2	512400	XM_002698777	-1.187197534	-1.435387239	1.209055105

ProbeID	Gene_Symbol	Gene_ID	mRNA Accession	CM12H/CM0H.fc	CM12R/CM0H.fc	CM12H/CM12R.fc
12735280	NOC2L	508638	NM_001034326	-1.187452661	1.476519081	-1.753296511
12907860	LOC509805	509805	XM_002699648	-1.189231844	-1.400867009	1.177959551
12848331	BMP8A	788948	NM_001206869	-1.192905699	1.120863037	-1.337083904
12871075	BDH2	515321	NM_001034488	-1.193145512	1.250590965	-1.492136998
12828451	ANXA7	533360	NM_001075991	-1.194684771	1.357996725	-1.622378007
12816081	ARPC1A	508402	NM_001075359	-1.19494979	-1.174763218	-1.017183525
12704737	RRM2	508167	NM_001244181	-1.194982922	-1.193666652	-1.001102712
12779857	LOC100137987	100137987	XR_804899	-1.195579446	1.011825147	-1.209717349
12892676	ALDH1A1	281615	NM_174239	-1.195770065	-1.317259879	1.101599645
12700208	OLFML2A	516730	XM_002691603	-1.195852952	-1.112287701	-1.075129169
12739991	IL24	526285	XM_010813062	-1.197063762	-1.032420004	-1.159473623
12892762	VLDLR	282123	NM_174489	-1.197760948	-1.223420198	1.021422681
12835617	MIR2414	100313201	NR_031310	-1.200229255	-1.32397878	1.103104907
12742473	TMEM154	510523	XM_002694322	-1.201536104	-1.133292292	-1.06021731
12877266	NDUFA11	326346	NM_175718	-1.203628356	-2.132535858	1.77175608
12862666	ADM2	618896	NM_001193200	-1.203970464	-1.070385146	-1.12480117
12870423	SEC24D	504422	XM_002688058	-1.207907517	-1.055985482	-1.143867542
12804072			GENSCAN00000042013	-1.208208968	-1.333141577	1.103403147
12726834	MMP7	286794	NM_001075130	-1.209273019	-1.124863544	-1.075039746
12874466	SGMS2	520954	NM_001205877	-1.209507739	-1.226238834	1.01383298
12701582	INPP4A	535487	NM_001206848	-1.209901835	1.147528512	-1.388396853
12702319	PHYHD1	540828	NM_001076243	-1.210094738	1.123056103	-1.35900428
12692930	LOC100296102	100296102	XM_002690566	-1.210656847	-1.238685555	1.023151654
12808208	SERPINB5	534323	NM_001098080	-1.210765943	-1.3689787	1.130671628
12789434	TRAF3	506182	NM_001205586	-1.211613871	1.690910149	-2.048730191
12853822	NFE2L3	510807	NM_001077899	-1.216899479	-1.064562019	-1.143098718
12864745	C1QTNF6	506413	NM_001101872	-1.217135679	-1.341177328	1.101912754
12683407	ST6GAL1	282073	XM_005201456	-1.218342703	-1.256220746	1.03108981
12701879	ALK	536642	XM_002691492	-1.218418709	-1.485027863	1.218815709
12735094	MR1	506206	NM_001190298	-1.21869744	1.747908907	-2.130172111
12801335	ECI2	505355	NM_001034242	-1.219255094	-1.428281155	1.171437513
12871498	NPFFR2	530560	XM_002688346	-1.220278122	1.428588091	-1.743274793
12842441	MIR197	100313015	NR_030830	-1.220921124	1.095287796	-1.337260007
12857400	STAT6	353105	NM_001205501	-1.221293543	1.651766779	-2.017292102
12819230	HOGA1	506001	NM_001075237	-1.221513662	-1.123803657	-1.086945798
12872195	PPM1K	540329	NM_001046474	-1.222199673	-1.034397003	-1.181557632
12730503	LOC100140748	100140748	XM_001787493	-1.223980013	-1.277691909	1.043882984
12803467	LOC574091	574091	NM_001025347	-1.224064856	1.226238834	-1.500995862
12795198	THOC7	510250	ENSBTAT00000046670	-1.224582524	1.176556009	-1.440789927
12739457	LOC514121	514121	XM_010800116	-1.22532971	-1.63808475	1.336852226
12855679	AGMO	613658	NM_001192973	-1.226306833	-1.030832551	-1.18962758
12824834	VEGFC	282122	NM_174488	-1.227922919	-1.400624278	1.140645114
12699276	PRKCE	507041	NM_001111120	-1.228910628	1.508160531	-1.853394506
12779933	HOXD3	100295744	XM_010801922	-1.231050547	1.30651166	-1.608381893
12848620	MIR2422	100313434	NR_030879	-1.23105908	-2.113493976	1.716809542
12771467	ASGR2	531519	NM_001075952	-1.231562633	-1.592805381	1.293320647
12881959	LOC618071	618071	NM_001206718	-1.232057852	1.540277186	-1.897710602
12866697	LOC524576	524576	XM_002687704	-1.232382414	-1.617650566	1.312620618
12764948	NAGLU	789125	NM_001102226	-1.23255327	1.63746038	-2.018257146
12830405	ALDH3B1	511469	NM_001075518	-1.233869657	1.547007295	-1.908805361
12894591	C8H9orf40	533577	XM_002689652	-1.234690974	-1.615678334	1.308569001
12824974	RPL15	507151	ENSBTAT00000020729	-1.235315882	-1.275745011	1.032727766
12882286	PALM	786096	XM_010800088	-1.235435764	-1.278099363	1.03453324
12688861	SRSF5	510474	NM_001098929	-1.237955967	1.256760724	-1.555814438
12786629	PPAP2A	617172	NM_001080329	-1.239011862	1.174852792	-1.455656546
12829250	ANO9	512371	XM_003588068	-1.241522149	-1.04298615	-1.190353438
12741058	FAM129A	614787	NM_001191282	-1.242176345	1.125261259	-1.397772918
12726622			ENSBTAT00000010545	-1.243046271	-1.054127956	-1.179217631
12870149	PDGFRA	282301	NM_001192345	-1.243192754	1.112734959	-1.383344038
12801040				-1.244546384	1.14423232	-1.424050196
12850240	STEAP1	515156	NM_001205806	-1.24645431	-1.159771025	-1.074741723
12712871	SAP18	615692	NM_001035467	-1.248460347	1.122192363	-1.401012667
12713867	PLCB1	287026	NM_174817	-1.249724422	1.779719651	-2.224159113
12738576	PTGS2	282023	NM_174445	-1.249733085	1.076695277	-1.345581709
12880368	LOC789086	789086	XM_001255941	-1.25035694	-2.241166759	1.792421579
12880677	MRI1	534734	NM_001046387	-1.251978685	-1.294199478	1.033723252
12713409	SLC32A1	100301361	XM_002692459	-1.25260366	-2.000748739	1.597271988
12735535	GLRX2	513762	NM_001040523	-1.253263696	-1.110454284	-1.128604494
12779348	STPG1	616738	NM_001102304	-1.253289757	1.90757529	-2.390744572
12801306	RNF39	504845	XM_003583890	-1.254367426	-1.326596845	1.057582346

ProbeID	Gene_Symbol	Gene_ID	mRNA Accession	CM12H/CM0H.fc	CM12R/CM0H.fc	CM12H/CM12R.fc
12829890	RPS6KB2	506083	NM_001205582	-1.254932703	-1.080583082	-1.161347724
12681617	SLC9A9	535743	NM_001076068	-1.256203331	-1.254480461	-1.001373374
12869291	NCAPD2	786159	NM_001206711	-1.259542672	1.601062914	-2.016607061
12825905	SFTPD	282072	NM_181026	-1.260608242	-1.234160478	-1.021429761
12852647			GENSCAN00000001951	-1.261368666	-2.233010446	1.770307529
12841059	LOC613418	613418	XM_002686600	-1.261674713	-2.062794351	1.634965281
12713857	BPIFA2A	286882	NM_174803	-1.262260781	-1.197611517	-1.053981833
12853311	GARS	408010	NM_001097566	-1.26228703	-1.098831368	-1.148754092
12908754	LOC522586	522586	XM_010799369	-1.262584548	-2.051287915	1.624673705
12786020	C1QTNF3	531659	NM_001101138	-1.263652691	-2.596791171	2.054988044
12811798	TAF6	505638	NM_001015525	-1.264502597	1.667665567	-2.10876744
12889086	TLR4	281536	NM_174198	-1.265011061	-1.147170635	-1.102722666
12857816	TOM1	504912	NM_001035015	-1.265949627	1.67204074	-2.116719351
12682147	XRN1	540834	NM_001206035	-1.267626742	1.072382811	-1.359381129
12827996	ANK3	511203	NM_001105620	-1.269693256	1.627311006	-2.06618581
12751650	PPP1R15A	514688	NM_001046178	-1.27059126	1.572936031	-1.998558773
12815049				-1.272503831	-1.008240631	-1.262103303
12836043	SLC6A9	515755 // 282368	NM_001242342	-1.272865516	1.15863006	-1.474780249
12725675	IMPAD1	532797	NM_001046335	-1.272927277	-1.185667921	-1.073595106
12864124	CYP2D14	282211	NM_174529	-1.274048325	1.161548987	-1.479869541
12892163	LOC785403	785403	XM_010798665	-1.274481119	-1.014071938	-1.25679557
12710595	RGCC	614348	NM_001102276	-1.275064298	-1.308251579	1.026027927
12807934	POLI	515909	NM_001024692	-1.275789226	1.841472688	-2.349331015
12796146	OXSR1	526949	NM_001075892	-1.276081081	1.219525564	-1.5562135
12883748	POLRMT	504757	NM_001205551	-1.277479376	1.588329245	-2.029057853
12884486	SPC24	509117	NM_001075391	-1.278533533	-1.091816194	-1.17101536
12710773	SLC46A3	781117	NM_001103303	-1.278861474	-1.316338017	1.029304615
12707523	OXER1	786807	XM_005193887	-1.281141653	-1.202235895	-1.065632509
12909086	LOC531038	531038	XM_005200747	-1.281390323	-1.025430703	-1.249611816
12708898	LOC100337053	100337053	XM_010810913	-1.284208987	-1.618401991	1.260232569
12804243	LOC100298767	100298767	XM_002702931	-1.286168802	-1.126931637	-1.14130153
12892432				-1.286195547	-1.74809065	1.359117324
12678900	CLDN1	414922	NM_001001854	-1.289838109	1.270908354	-1.639266027
12831035	FADS2	521822	NM_001083444	-1.290455149	-1.430094021	1.108209009
12709067	TRPC4	282102	NM_174478	-1.291475254	1.240335148	-1.60186215
12851128	KLHL7	534697	NM_001076037	-1.293410296	1.066164463	-1.378988093
12827718	ANXA8L1	281627	NM_174241	-1.294755781	1.110138748	-1.437358562
12789104	IDH3A	282446	NM_174644	-1.295949948	-1.01233024	-1.280165204
12909652	LRCH2	100336559	XM_003588142	-1.295994863	1.690089913	-2.190347845
12908989	LOC527430	527430	XR_802762	-1.298458586	1.74919363	-2.271255487
12774061	FBXO42	100848477 // 527807	XM_005197658	-1.298512588	1.475097177	-1.915432253
12723779			GENSCAN00000029163	-1.299773284	1.852880707	-2.408324842
12698714	SLC1A4	326577	NM_001081577	-1.299926452	1.087209524	-1.41329242
12720944	MRGBP	616297	NM_001206561	-1.300818788	-1.062394807	-1.22442126
12848893	SCIN	281478	NM_174177	-1.301350876	1.106474344	-1.439911357
12840641	MYCBP	539291	NM_001038565	-1.301648579	1.110107969	-1.44497046
12824734	MIR2398	100313428	NR_031172	-1.30321038	-2.017264137	1.547919022
12906724			GENSCAN00000004138	-1.303327816	-1.384264851	1.06210029
12906742	TENM1	100336965	NM_001256555	-1.303933234	-1.110546653	-1.174136386
12749523	CDH1	282637	NM_001002763	-1.306910187	1.229046926	-1.606253948
12834822	MACROD1	613568	NM_001046509	-1.307018897	-1.308795778	1.001359492
12885558	HSPA9	517535	NM_001034524	-1.307172919	1.069265411	-1.397714788
12794421	TLR9	282602	NM_183081	-1.307462891	1.505559787	-1.968463552
12763389	CYB5D2	515356	XM_002695757	-1.307943299	1.758079122	-2.299467807
12842734	VCAM1	282118	NM_174484	-1.310611413	1.648175648	-2.160117814
12887608	CD74	613384	NM_001034735	-1.310620497	-1.17871097	-1.111909986
12894487	CNTLN	532280	NM_001192721	-1.313904119	1.504756449	-1.977105697
12729535	RNF26	539611	XM_002693026	-1.316027832	1.682725673	-2.214513819
12848511			ENSBTAT00000023762	-1.319809768	-2.119861485	1.606187147
12837750	CC2D1B	511350	XM_010803562	-1.320139145	1.526777679	-2.015558979
12897765	LAMA4	529670	NM_001205965	-1.320459451	-1.244149626	-1.061334926
12841065	LINGO4	613529	XM_002684068	-1.322859645	-1.32464888	1.001352551
12708750	MIR19A	100170926	NR_030794	-1.323043045	1.665932561	-2.204100488
12906107	PABPC1L2A	784438 // 521086	XM_010821834	-1.3235567	-1.683075622	1.271630919
12824697	LOC100336146	100336146	XM_010799201	-1.324713154	-1.081309859	-1.225100411
12901037	LCA5	537291	XM_005210689	-1.324832528	1.54428604	-2.045920378
12787132	CENPH	505284	NM_001034236	-1.328658185	1.558383168	-2.070558552
12907898	DDX3X	510093	NM_001192962	-1.32920166	1.244270365	-1.653886235
12763074			ENSBTAT00000014061	-1.331064055	1.105677004	-1.471726917
12905875	TSPYL2	617179	XM_002700120	-1.333881033	1.459435043	-1.946712722

ProbeID	Gene_Symbol	Gene_ID	mRNA Accession	CM12H/CM0H.fc	CM12R/CM0H.fc	CM12H/CM12R.fc
12806486	BLA-DQB	539241	NM_001034668	-1.339709311	1.07466723	-1.439741695
12774447	YARS	281581	NM_174220	-1.340907761	1.185043485	-1.589034007
12906911	LOC782803	782803	XM_001787351	-1.341558531	-1.44413939	1.076463946
12847429	PIFO	615099 // 617486	XM_002686144	-1.342042165	-1.578736065	1.176368452
12796360	WNT7A	533782	NM_001192788	-1.34241431	-1.501266394	1.118333127
12864151	SLC2A3	282358	NM_174603	-1.343121669	-1.046200961	-1.283808484
12819189	TLX1	505304	NM_001192057	-1.344649346	-2.108285139	1.567907012
12735234				-1.3450968	-1.3450968	1
12887709	HAVCR1	613999 // 614846	XM_010798531	-1.345180714	2.406022277	-3.236534764
12777248	CALCRL	527140	NM_001102107	-1.34689744	-1.55360527	1.153469614
12805515	GTPBP2	513605	NM_001083421	-1.34773794	1.017909994	-1.371875918
12770993	PLD2	522159	NM_001075827	-1.347765966	1.552066099	-2.091821865
12871633	ELOVL6	533333	NM_001102155	-1.348513533	-1.367063256	1.013755682
12765895	ULBP27	785947	NM_001168616	-1.349009025	-1.232954876	-1.094126843
12703851	ACTG2	281595	NM_001013592	-1.349935054	-1.346290737	-1.002706931
12749590	HAS3	286824	NM_001192867	-1.353626765	1.496020557	-2.025053468
12713731	PROCR	282005	NM_174437	-1.354124136	-1.472920945	1.08772963
12723461	LOC101903806	101903806	XM_010812106	-1.355589155	-2.115868548	1.560847946
12713107	RCBTB1	785222	NM_001191212	-1.357808479	1.011509591	-1.3734363
12815225	TNP2	281538	NM_174200	-1.357817891	-2.044601947	1.505799828
12909019	LOC528342	528342	XM_002699647	-1.358128512	-1.196085068	-1.135478194
12856851	KRT8	281269	NM_001033610	-1.358420372	-1.13779449	-1.193906618
12731267	CD59	505574	NM_001037446	-1.358486285	-1.068287529	-1.271648547
12785148	SEPP1	282066	NM_174459	-1.359597864	1.496580622	-2.034747816
12908119	CXHXorf57	511907	XM_001789923	-1.360927299	-1.013193692	-1.34320546
12896865	ABRACL	505914	NM_001166561	-1.36223915	-1.19043595	-1.144319566
12807507	DSC3	281129	NM_001178103	-1.36351446	-1.295716415	-1.052324755
12908260	CA5B	514494	NM_001080908	-1.364696367	1.05363122	-1.437886699
12895307	C8H9orf152	614478	NM_001206177	-1.365547975	-2.120405224	1.552787059
12878948	ERAP2	513572	NM_001075628	-1.365898234	1.339588597	-1.829741698
12711148	EDNRB	281750	NM_174309	-1.365973978	-1.072338213	-1.273827568
12715490	MKX	521239	NM_001192534	-1.36804909	-1.660376019	1.213681607
12887402	LMNB1	540643	NM_001103295	-1.370269818	-1.052631155	-1.301756851
12832203	LOC787455	787455	XM_001254846	-1.37124846	-1.119853492	-1.224489158
12755837	SLC1A5	282355	NM_174601	-1.371733289	1.0244219	-1.405233622
12875869	PROM1	618054	NM_001245952	-1.371913955	-1.151209179	-1.191715615
12843247	PHGDH	505103	NM_001035017	-1.373512462	-1.461773718	1.064259523
12882175			ENSBTAT00000054971	-1.374074284	-1.172192897	-1.172225397
12905311	TAF9B	538983	NM_001192149	-1.377421403	1.350047343	-1.859584106
12859619	USP18	515202	NM_001017940	-1.377822459	1.289006912	-1.776022674
12896617			ENSBTAT00000064900	-1.377927517	-2.183995676	1.584985893
12848599				-1.380508719	-1.025857256	-1.345712292
12848438	LOC101904529	101904529	XR_225878	-1.381456371	-1.061342283	-1.30161249
12681567	CD200	534910	NM_001034620	-1.381829867	-1.176604941	-1.174421268
12737673	C16H1orf21	781721	NM_001081547	-1.3820885	-2.121125525	1.53472482
12690936	KLHDC1	537136	NM_001192897	-1.382768841	1.069762102	-1.479233702
12894605	CD274	533834	NM_001163412	-1.382883862	1.474657585	-2.039280176
12903493	GPRASP1	100124524 // 507696	XM_010800428	-1.383391982	-1.162169099	-1.190353438
12780852	MARCH7	507865	NM_001100317	-1.383986624	1.200894988	-1.6620226
12684718	CCDC80	515235	NM_001098982	-1.388695217	-1.249785061	-1.111147237
12685704	BDH1	534090	NM_001034600	-1.390053105	-1.524007497	1.096366385
12876521	MIR2456	100313300	NR_030809	-1.390544583	1.775136542	-2.468406502
12806652			GENSCAN00000024182	-1.392618409	-1.156359511	-1.204312669
12802848	UHRF1BP1	534225	XM_002697198	-1.39554631	1.029368828	-1.43653187
12852542	LOC789101	789101	XM_010804711	-1.395643045	-1.667434395	1.194742739
12764323	LRRC59	532659	NM_001034578	-1.396378453	-1.093459662	-1.27702786
12682962	LOC100295890	100295890	XR_804437	-1.398170208	-1.071981494	-1.30428577
12811259				-1.40051749	-3.007385894	2.147339049
12703833	IL1A	281250	NM_174092	-1.400643695	1.240954311	-1.738134832
12832473	LOC100301071	100301071	XM_002699098	-1.401721756	-1.139136001	-1.230513086
12730068	LOC618213	618213	XR_811567	-1.402246517	-2.029733054	1.447486607
12873374			GENSCAN00000028999	-1.403024304	-1.573579426	1.121562486
12856116	THSD7A	782187	NM_001206743	-1.405369993	-1.317570355	-1.066637533
12825182	SLC20A2	518905	NM_001080280	-1.406441943	-1.043188594	-1.348214456
12702138	DNAJC27	540033	NM_001098109	-1.407895251	-1.152223031	-1.221894732
12895413	AAED1	616897	NM_001076436	-1.409477062	-1.196723616	-1.177779934
12896505	LAMA4	529670	NM_001205965	-1.409672471	-1.05373347	-1.337788455
12866832	METTL20	530461	XM_005198364	-1.409994954	-1.070912049	-1.316630022
12721560	MIR2310	100313355	NR_031046	-1.411129118	-2.20094028	1.559701556
12763498	LOC517569	517569	XM_002695737	-1.414566499	-1.084439824	-1.304421387

ProbeID	Gene_Symbol	Gene_ID	mRNA Accession	CM12H/CM0H.fc	CM12R/CM0H.fc	CM12H/CM12R.fc
12760480	MIR2348	100313158	NR_031289	-1.415812286	-2.344125807	1.655675565
12823815	DLC1	511433	NM_001102493	-1.418680778	1.4175995	-2.011121161
12846912	MLLT11	539519	NM_001076169	-1.421850716	1.06355895	-1.512222054
12763615	FAM20A	521099	XM_002707768	-1.422590072	-1.279136299	-1.112148934
12859097	METTL1	511197	NM_001046077	-1.424622815	1.001574683	-1.426866144
12882858	LOC100335642	100335642	XM_002683636	-1.427598212	1.668128006	-2.381416559
12725851	WISP1	539163	XM_005215320	-1.428241556	-1.169733599	-1.220997291
12803716	HIST1H2AM	618824	XM_002697505	-1.428241556	1.586469736	-2.265862003
12819166	PTPRE	505285	NM_001205531	-1.429776852	1.409398907	-2.015125931
12681523	BCHE	534616	NM_001076906	-1.430986439	1.069836255	-1.530921172
12898542	RWDD2A	539733	NM_001205704	-1.433209985	1.061437924	-1.521263431
12786321	ESM1	539571	NM_001098101	-1.433259657	-1.283568242	-1.116621314
12885042	TYK2	512484	NM_001113764	-1.437079625	1.401265178	-2.013729637
12745292	GLRB	281198	NM_174071	-1.438664309	1.010311378	-1.453498921
12783551	KHDRBS1	538775	NM_001046442	-1.44323877	1.62054603	-2.33883486
12888848	MIR2466	100313235	NR_031006	-1.44379909	-1.086426067	-1.328943712
12737340	SMYD3	616050	NM_001076406	-1.444419698	1.062608383	-1.53485248
12759917	GYS1	511047 // 786335	NM_001024515	-1.444549859	1.480916194	-2.13925728
12818411	MSRB1	618441	NM_001034810	-1.446152805	1.596663175	-2.309018929
12788683				-1.44716558	-2.199430478	1.519819507
12728837	LOC531237	531237	XM_002692896	-1.448158989	-1.661550337	1.147353536
12770563	COG1	515788	NM_001192427	-1.448851768	1.409545452	-2.04222242
12769400	CCR7	510668	NM_001024930	-1.450972322	1.695628358	-2.460309817
12685403	NLGN1	530042	NM_001205973	-1.451716759	-1.822577295	1.255463425
12848091	ADAM15	784605	NM_001205442	-1.451867705	-1.080950156	-1.343140289
12906591	MIR2486	100313248	NR_031275	-1.452562258	-2.177088417	1.498791811
12727485	LOC510901	510901	XM_005216558	-1.453025478	-1.7126021	1.17864561
12848523			ENSBTAT00000053230	-1.453589597	-2.116132554	1.455797811
12843331	GADD45A	505463	NM_001034247	-1.455111797	1.370877824	-1.994780493
12787721	IL31RA	522636	NM_001192563	-1.459981411	1.640709703	-2.395405667
12704110	SLC8A1	337925	NM_176632	-1.462209469	1.154549477	-1.688193179
12762177	COPS3	507932	NM_001098904	-1.464045103	1.445601592	-2.116425932
12825497	SORBS2	614391	NM_001079787	-1.465933846	1.871869683	-2.744037124
12857108	TIMP3	282094	NM_174473	-1.467133344	1.032298356	-1.514519339
12804444	EDN1	281137	NM_181010	-1.467957296	2.223619594	-3.264178607
12826173	LOC510670	510670	NM_001105618	-1.468588288	1.128604494	-1.657455343
12870553	TACC3	506194	NM_001100305	-1.469413058	1.632451353	-2.398745334
12687887	FOS	280795	NM_182786	-1.469688084	1.655595233	-2.433208586
12897508	PDE7B	522509	NM_001102068	-1.471227141	1.689316912	-2.485368891
12821369	SCD	280924	NM_173959	-1.471267933	-1.177510562	-1.249473237
12856557	KRT77	100336698	XM_002687245	-1.471543306	-2.234930549	1.518766413
12678851	NDUFB4	327706	NM_175822	-1.47312515	-2.228588109	1.512830128
12870067	PDE5A	281972	NM_174417	-1.479797739	-1.07656095	-1.374560112
12685825	ERG	535231	NM_001102183	-1.480351731	1.377917966	-2.039803245
12732587	ZNF215	529157	NM_001076882	-1.481747887	-1.481747887	1
12734323			ENSBTAT00000034028	-1.481963587	-1.173786483	-1.262549542
12783122	FAM76A	535402	NM_001015660	-1.483011721	1.460285037	-2.165619825
12891947	LOC616977	616977	XM_005199128	-1.485161684	1.556731355	-2.31199776
12678517	MX1	280872	NM_173940	-1.485563218	-1.181885274	-1.256943673
12902566	MIR502B	100313383	NR_031326	-1.488346049	-2.23225215	1.499820658
12873740	SDAD1	507675	NM_001102489	-1.489749744	1.606187147	-2.392816892
12840882	EXTL2	540527	NM_001076224	-1.490648391	1.237338299	-1.844436345
12857450	KRT6B	404115	NM_001257404	-1.490679389	-1.494393412	1.002491497
12835816	DPYD	281124	NM_174041	-1.490896389	-1.392994923	-1.07028128
12893429	TDH	511957	NM_001046104	-1.492323178	1.554434687	-2.319718912
12754736	CMTM3	787512	NM_001099399	-1.492426622	1.231741913	-1.838284422
12883402	ANXA6	327685	NM_001103224	-1.493368287	1.008492252	-1.506050347
12834862	LOC614402	614402	XM_010821155	-1.495004679	-2.016956543	1.349130588
12879927	DAPK3	525506	NM_001101124	-1.495543631	1.345553729	-2.012334309
12908337	DNASE1L1	515176	NM_001038634	-1.496985243	1.518271711	-2.272830347
12832208	LOC100336475	100336475	XM_001255100	-1.498241304	1.389898952	-2.082404017
12803409	TFAP2B	540867	NM_001076247	-1.500340546	1.363977646	-2.046430966
12746511	LOC100852110	100852110	XM_005195105	-1.502234462	-1.842787861	1.226697901
12767023	CCL2	281043	NM_174006	-1.50478774	-1.257980884	-1.196192851
12759042	MT1E	613358 // 768319	ENSBTAT00000002088	-1.504860755	-2.774908114	1.843963374
12870804	KIAA1211	782369	XM_005198628	-1.50491291	-1.85511677	1.232707061
12803653			ENSBTAT00000015992	-1.507000609	-1.132216615	-1.331017925
12833745				-1.507637933	-2.369643451	1.571758974
12697060	C10H14orf105	614197	XM_005192421	-1.509258577	-1.038599906	-1.453166487
12689010	DCAF4	511629	NM_001024520	-1.51077624	1.569255211	-2.370793488

ProbeID	Gene_Symbol	Gene_ID	mRNA Accession	CM12H/CM0H.fc	CM12R/CM0H.fc	CM12H/CM12R.fc
12744061	HIC2	539541	NM_001192270	-1.510912381	-1.484616183	-1.017712456
12868043	MDM2	540378	NM_001099107	-1.51365876	1.564368076	-2.367919441
12755848	SLC7A5	282369	NM_174613	-1.521210709	-1.119496486	-1.358834733
12751621	LPCAT2	514646	NM_001205797	-1.522582076	1.329892839	-2.024871
12827408	TMEM26	100125281	NM_001103163	-1.522698172	-1.830832745	1.2023609
12792414	MTHFS	513372	NM_001075616	-1.523764553	-1.393555055	-1.093436924
12729909	PTPMT1	614890	NM_001076353	-1.523764553	-1.181180955	-1.290034814
12812019	NDE1	508088	NM_001100321	-1.525095938	-1.125386061	-1.355175784
12726238	LOC782385	782385	XM_002692707	-1.525391959	-1.206735925	-1.26406443
12904529	TMEM164	524183	NM_001031762	-1.525698613	-1.167789302	-1.306484492
12813989	BAIAP2L1	615412	XM_003584061	-1.531282007	-1.235983942	-1.238917396
12822595	LOC532031	532031	XM_002698595	-1.531993313	-2.100743471	1.37124846
12684618	CBS	514525	NM_001102000	-1.532322536	-1.26366145	-1.212605272
12854099	ASNS	514209	NM_001075653	-1.532503108	-1.334389646	-1.148467475
12824044	SMIM19	515895	NM_001098040	-1.532811192	1.375894641	-2.108986704
12733912	LOC785144	785144	NM_001206667	-1.533608247	-1.449042591	-1.058359676
12864496	NAB2	504755	NM_001045897	-1.534022879	-2.131176383	1.38927288
12706002	MTHFD2	517539	NM_001075755	-1.536800613	-1.152199071	-1.333797823
12692926			ENSBTAT00000064523	-1.537375945	-2.173982007	1.414086135
12802397	CDSN	522351	NM_001077950	-1.540085023	-1.50257812	-1.024961699
12712739	TEX30	613895	NM_001076295	-1.540095699	-1.118720779	-1.376657811
12901187	BCLAF1	540648	NM_001098117	-1.54073634	1.235204574	-1.903124574
12728735	LOC528807	528807	XM_002693269	-1.541227678	-1.318200662	-1.16919049
12893297	NMRK1	510456	XM_002689653	-1.550109359	1.510137589	-2.34087841
12869838	CXCL8	280828	NM_173925	-1.554639416	-1.260809229	-1.233048887
12806992	GSTA3	768055 // 777644	ENSBTAT00000005618	-1.557055102	-2.053492958	1.318831271
12756919	MGC138914	512219	NM_001083673	-1.559063835	-2.092590475	1.342209618
12905630	CDK16	613810	NM_001101226	-1.559928604	1.286133142	-2.006275877
12811225				-1.560501777	-2.058537885	1.31915126
12864595	MFNG	505267	NM_001101051	-1.560512593	-1.402168763	-1.112927798
12825327	LOC533821	533821	XM_005226034	-1.564389762	1.707055542	-2.670500214
12899272	PTPRK	509657	NM_001191537	-1.568167863	-1.143930973	-1.370858819
12791431	MIR345	791018	NR_031357	-1.569461893	-2.215450357	1.411598694
12789500	ISG20	506604	XM_002696514	-1.57332858	-2.108869761	1.340387372
12739381	FBXO6	513023	NM_001034433	-1.579984056	-1.168501835	-1.352145122
12806178	MIC1	533051	NM_001127317	-1.580553643	-2.010549701	1.272054074
12762208	TMEM106A	508269	NM_001014870	-1.582845015	1.578757951	-2.498929153
12862571	MAFF	617914	NM_001103300	-1.584480605	1.373502941	-2.176288771
12896514	ESR1	407238	NM_001001443	-1.587635801	-1.297244123	-1.22385276
12738141	LOC101905630	101905630	XM_005200961	-1.587954968	-2.032281148	1.279810316
12688416	CHAC1	505991	NM_001098882	-1.588439343	1.097560145	-1.743407716
12893194	AADAT	508929	NM_001015551	-1.58898995	1.226995536	-1.949683575
12836500	CITED4	504742	NM_001080726	-1.592330711	1.443618964	-2.298718811
12819467				-1.594020297	-1.39477267	-1.14285312
12732109	LOC515027	515027	XM_002693199	-1.594230241	-1.955367799	1.226527856
12814286	PRR35	101902553	XM_005196865	-1.594517576	-1.206652284	-1.321439157
12825859	PLAU	281408	NM_174147	-1.59692881	1.211529891	-1.934726988
12786624	ENC1	617091	NM_001078067	-1.598822743	1.348298565	-2.15569041
12829803	LOC505546	505546	XM_002699202	-1.599410207	-2.093809227	1.309113333
12905482	ARAF	540421	NM_001014964	-1.604462421	1.274551793	-2.044970455
12849229	HGF	282879	NM_001031751	-1.605252227	-1.545549649	-1.038628703
12791245	LOC617694	617694	XR_813961	-1.606420962	-2.267495991	1.41152042
12826638	FAM213A	534049	NM_001034599	-1.608393042	-1.208351346	-1.331064055
12843892	CYR61	508941	NM_001034340	-1.610501495	1.578725122	-2.54253917
12807392	OSBPL1A	100336844	XM_002697723	-1.610724773	1.350449789	-2.17520293
12897798			GENSCAN00000029329	-1.611472982	-1.767830555	1.097027735
12769340	C19H17orf85	510317	XM_002695755	-1.611774597	1.290669841	-2.080268862
12787973	CDH6	537946	NM_001034640	-1.616215977	-1.776823033	1.099372274
12803691	LOC618034	618034	XM_002697413	-1.617729057	-2.190772992	1.354227386
12761426	MYL4	504201	NM_001075149	-1.618435645	-2.005288762	1.239029039
12813690	PLK1	538238	NM_001038173	-1.620961696	1.42416865	-2.30852283
12873299	EVC	282874	NM_174747	-1.622737901	-1.298404585	-1.249793724
12710489	THSD1	541228	NM_001014967	-1.623210385	1.546406922	-2.510143774
12752602	LOC530319	530319	XR_803009	-1.627085428	-1.542296346	-1.054975869
12863649	MIR2437	100313217	NR_031054	-1.633798428	-2.190772992	1.340907761
12815621	ABCA3	505787	NM_001113746	-1.638641207	-1.187979549	-1.379351361
12738172			NM_174771	-1.646542784	-2.124848527	1.290490928
12868434	CCND2	615414	NM_001076372	-1.647353304	-1.590290138	-1.035882236
12878534	DOT1L	510442	XM_003586273	-1.650462089	1.224293961	-2.020650768
12889824	TTC39B	508428	XM_002689579	-1.65457421	-1.132577677	-1.460892479

ProbeID	Gene_Symbol	Gene_ID	mRNA Accession	CM12H/CM0H.fc	CM12R/CM0H.fc	CM12H/CM12R.fc
12693777	BMP4	407216	NM_001045877	-1.66053715	-1.08335795	-1.532768694
12847127	ST6GALNAC5	540995	NM_001192804	-1.662806163	-1.920790269	1.155149838
12815104	LOC101904761	101904761	XR_240129	-1.667341936	-2.140948363	1.284048771
12750559			GENSCAN00000014380	-1.667850527	1.349485991	-2.250740921
12823293	MIR107	791005	NR_031010	-1.670523178	1.10254688	-1.841830118
12878044	CIRBP	507120	NM_001034278	-1.672620326	1.396165532	-2.335254847
12852779	PODXL	616366	XM_002686960	-1.673513281	-2.030197386	1.213134911
12693130	TCRA	785621	BC146053	-1.676497114	-1.272380353	-1.317606886
12860825	KRT73	531981	NM_001111104	-1.680615855	-1.715941061	1.021019203
12884646	LOC509641	509641	XM_002688615	-1.682550726	-1.472614692	-1.142560057
12890767	SLC31A2	526609	NM_001034556	-1.683273959	1.219703092	-2.053094452
12889817	ERCC6L2	508357	NM_001082606	-1.68812297	1.329478089	-2.2443225
12887236	MAST1	539825	NM_001191528	-1.691754235	-2.293323694	1.355589155
12895371	LURAP1L	616371	NM_001101256	-1.692892073	-1.430381517	-1.183524852
12797637	TMEM158	788085	NM_001081626	-1.692892073	-1.188671444	-1.424188393
12719955	GTPBP4	524743	NM_001192601	-1.69339672	1.226357835	-2.076710336
12862854	LOC781146	781146	NM_001080336	-1.693537579	-2.609837534	1.54105676
12908168	MAGED1	512562	NM_001046125	-1.694982056	-1.034146087	-1.63901607
12683750	TFRC	504698	NM_001206577	-1.696615915	-1.007409332	-1.684137581
12775831	TFPI	508763	NM_001244204	-1.696874655	-1.381590435	-1.228203824
12798062	CAMP	317650	NM_174831	-1.69762758	-1.515895182	-1.119884541
12816921	MVP	516456	NM_001035317	-1.699605597	-1.085402397	-1.565876031
12703161	GTF3C5	783869	NM_001191410	-1.699617378	1.299899421	-2.209331646
12832085	C29H11orf86	768031	NM_001077090	-1.700194738	-2.146133765	1.26228703
12890045	CDK20	510920	NM_001098933	-1.701102414	1.254019689	-2.13321592
12703348	ACTR1B	100125305	NM_001103338	-1.704100002	1.263678968	-2.153435332
12829160	LOC100296082	100296082	XR_816485	-1.705092492	-1.465811918	-1.16324098
12811261				-1.705518022	-2.944086378	1.726212412
12842453	MIR760	100313318	NR_031382	-1.706180169	-2.416283945	1.416195071
12877406	EGR1	407125	NM_001045875	-1.706487681	1.175911919	-2.006679204
12784953				-1.709625107	-2.163189419	1.265300451
12735710	ERRFI1	516303	NM_001077930	-1.712305355	1.305552071	-2.235503803
12888051	GDF15	618677	NM_001206298	-1.712459656	1.019972319	-1.746661447
12756723	C18H19orf54	509803	NM_001164025	-1.716024321	1.196723616	-2.053606831
12894099	LOC520638	520638	XM_002689448	-1.716036215	-2.814522146	1.640129806
12693229			ENSBTAT00000050159	-1.719381871	-2.01919466	1.174372426
12694411	LOC510112	510112	XM_002690711	-1.722351977	-2.540372398	1.474943816
12808491	ZNF521	538792	NM_001105419	-1.72396442	-2.06239404	1.196308935
12892466	MIR2474	100313239	NR_030917	-1.72500435	-2.111634292	1.224132734
12820727	HHEX	539542	NM_001105424	-1.726344034	1.35760144	-2.343687146
12826008	TBCE	505066	NM_001038032	-1.72927822	1.405282324	-2.430124116
12790204	CEMIP	519047	XM_002696612	-1.729925609	1.686275196	-2.917130646
12819589	SFXN2	513450	NM_001034446	-1.730393319	1.491444197	-2.580785074
12906399	LOC100851595	100851595	XM_010805270	-1.732445538	-2.011567291	1.161114302
12903893			ENSBTAT00000063298	-1.732493572	1.237535576	-2.144022432
12790698	PPP4R4	537521	XM_001790303	-1.742888165	1.30437618	-2.273381806
12902527	CCNB3	100301478	XM_003584903	-1.743383548	2.321102124	-4.046571256
12804310	LOC104975716	104975716	XR_815042	-1.747666612	-1.06553649	-1.640175281
12703617			ENSBTAT00000013286	-1.749302754	-1.931591473	1.104206501
12730328	LOC784741	784741	NM_001206638	-1.749678677	-1.396494605	-1.252907581
12902169			XM_005201066	-1.755497571	-2.54053088	1.447185642
12746326	ANKRD13A	511883	NM_001205709	-1.75625216	1.356839429	-2.382952179
12878509	ATP6AP1L	510390	NM_001207027	-1.759993377	1.27335969	-2.241104622
12892664	TSPY	281554	NM_001244608	-1.764928819	-2.33155102	1.321045356
12815216	PRM1	281423	NM_174156	-1.769829031	-2.036949199	1.150929928
12821481	PRKG1	282004	NM_174436	-1.770614327	1.018100513	-1.802663354
12807830	SERPINB10	510205	NM_001098925	-1.771792923	-1.330123312	-1.332051628
12816143	HAGH	509274	NM_001035274	-1.774718245	1.147520558	-2.036525671
12824540	DEFB7	768320	NM_001102362	-1.777278784	-2.212089872	1.244649907
12730088			GENSCAN00000016365	-1.780422947	-2.04973869	1.151265037
12898821	LOC614138	614138	XR_802042	-1.788066013	-2.473510459	1.383344038
12867247	ACVRL1	534536	NM_001083479	-1.790335541	-2.06781913	1.154989711
12871728	LOC534181	534181	XM_005198640	-1.79163903	-1.852302853	1.033859401
12804010	H2B	519934 // 787581 // 615043	NM_001105643	-1.795368515	-1.463568226	-1.226706404
12736341	LOC525101	525101	XM_002694075	-1.795891262	-2.159803408	1.202635958
12848526	LOC100295614	100295614	XR_806392	-1.796899847	-3.317462137	1.846214269
12852910	CD36	281052	NM_001278621	-1.796999491	1.250001651	-2.24625233
12685337	ANKRD28	529062	NM_001205960	-1.801664023	1.40136231	-2.524784056
12788050	NLN	538650	NM_001033989	-1.804451041	1.191492607	-2.149990075
12878231	DDX46	508660	XM_005199046	-1.807605686	1.337473215	-2.417624189

ProbeID	Gene_Symbol	Gene_ID	mRNA Accession	CM12H/CM0H.fc	CM12R/CM0H.fc	CM12H/CM12R.fc
12706587	MAPRE3	528839	NM_001075917	-1.808432813	1.190039945	-2.152107285
12721596	HAS2	281220	NM_174079	-1.809134915	-1.17566742	-1.538815216
12731091	LOC504200	504200	XM_002693829	-1.811267966	-2.614508934	1.443468875
12817732	PSPH	533630	NM_001046355	-1.812209817	-1.511362781	-1.199056799
12688328	ATP5S	493709	NM_001007812	-1.814535145	-2.049142053	1.129293119
12906561	LOC100300684	100300684	XM_002699503	-1.82776425	-2.281511617	1.248252676
12882714	CTH	539159	NM_001024567	-1.832965968	-1.35680181	-1.350945992
12801726	FBXO9	511798	NM_001034412	-1.833715725	-1.186021366	-1.546106822
12843293	THRAP3	505228	NM_001205565	-1.835686888	1.488603982	-2.73261081
12884126	OR1I1	507573	XM_002688604	-1.836081375	-2.223295946	1.210891835
12850461	LHFPL3	520147	NM_001104984	-1.837711121	-2.028579721	1.103862135
12833840	FADS3	515925	NM_001083691	-1.848314168	1.278117082	-2.36236191
12891041	PSAT1	533044	NM_001102150	-1.855001046	-1.966567901	1.060143823
12772434	ENO3	540303	NM_001034702	-1.856467424	-1.777278784	-1.044556116
12868116	C3AR1	540702	NM_001083752	-1.862654375	1.28158574	-2.387151286
12710343	HS6ST3	538566	NM_001205484	-1.864139725	-1.480916194	-1.258774624
12893731	IFT74	514024	NM_001205775	-1.867787076	1.138031113	-2.125599804
12864606	USP5	505280	NM_001192056	-1.868421563	1.400934981	-2.617537126
12811263	FUS	280796	NM_173912	-1.871739939	1.561767829	-2.923223222
12781280	LDLRAP1	511199	NM_001083668	-1.874375497	1.562276704	-2.928293174
12790928	LRFN5	539366	NM_001081610	-1.875441159	-2.075285754	1.106558712
12821842	SFXN4	508473	NM_001035060	-1.875493158	1.071632321	-2.009839087
12712708	LCP1	540990	NM_001034720	-1.875636163	-1.101630189	-1.702600548
12878696	OR2V1	511586	XM_002689049	-1.879071548	-2.001081602	1.064931032
12692273	LOC781758	781758	XM_002690701	-1.879266929	-2.556606278	1.36042743
12698604			ENSBTAT00000025313	-1.883518234	-1.041757871	-1.808019202
12865769	NCKAP1L	513641	NM_001143876	-1.902188211	1.515054824	-2.881919425
12719918	FERMT1	524427	NM_001206179	-1.912593132	1.113776687	-2.130201641
12809470	ATP9B	510301	NM_001080255	-1.922268681	1.139443982	-2.190317481
12786260	PLK2	539449	NM_001192245	-1.92324159	1.179250326	-2.267983273
12713287	WFDC10A	100296236	XM_005192497	-1.929530702	-2.033704403	1.053989139
12835715	F3	280686	NM_173878	-1.929597576	1.01179008	-1.952347686
12901838			NM_001034793	-1.931122923	1.096191612	-2.116880749
12761717	MSI2	505542	NM_001206656	-1.931564696	1.167101472	-2.254332
12800454	MAPKAPK3	615215	NM_001034779	-1.937948188	1.163805525	-2.255394808
12896174			GENSCAN00000000783	-1.941403503	-1.593280193	-1.21849472
12800430	NICN1	614730	NM_001034767	-1.941793789	1.036126391	-2.011943791
12866349	INHBE	517587	NM_001205842	-1.941874547	-1.367584522	-1.419930188
12880562	MAPK9	534125	NM_001046369	-1.942184153	1.213647957	-2.35712783
12903971	LOC513925	513925	XR_803719	-1.948184078	-2.004871817	1.029097732
12859705	LOC515697	515697	NM_001102030	-1.948535208	-1.944581903	-1.002032985
12682595	B3GALNT1	767844	NM_001076963	-1.953552463	-1.804713718	-1.082472219
12866101	DUSP6	515310	NM_001046195	-1.954771533	1.132938854	-2.214636621
12681726	BTD	537669	NM_001102206	-1.965954592	-1.414939139	-1.389426964
12760464	MIR744	100313080	NR_030988	-1.967699617	-2.285864679	1.161693919
12817714	IGFALS	532494	NM_001075963	-1.972000613	-2.142596228	1.086508906
12822177	FBXW4	513977	NM_001101985	-1.972137306	1.397385427	-2.755835932
12888738	TMEFF1	100139803	NM_001205476	-1.977201629	-2.600177299	1.315079485
12806778	HIST1H2BI	616776	XM_002697461	-1.979917065	1.033665932	-2.046572819
12872256	DKK2	541161	NM_001082615	-1.982210257	1.012021542	-2.006039481
12821871	ANKRD1	510376	NM_001034378	-1.983804695	2.147279513	-4.25978318
12903830	KIAA2022	512493	XM_002699975	-1.984698691	1.014782116	-2.014036738
12744947	HRK	787139	NM_001191241	-1.986914786	-2.839702905	1.429202161
12778525	SLC25A12	539494	NM_001101194	-1.988747339	1.065447865	-2.118906606
12850012	NT5C3A	511858	NM_001037597	-1.991119763	-1.19409697	-1.667469069
12881383	SH3RF2	540718	NM_001083753	-1.995527278	1.39056386	-2.774908114
12679507	LOC508623	508623	XR_804310	-1.999182254	-2.081148627	1.04099995
12688601	OR10G2	507759	XM_002690730	-2.00120644	-1.449976984	-1.38016428
12858096	ISX	506388	XM_010805321	-2.002649576	-1.625270667	-1.232194499
12794639	MGLL	505290	NM_001206681	-2.002663457	-1.813755514	-1.104152926
12777117	LOC524236	524236	XM_005197411	-2.00316325	-1.448540478	-1.382883862
12855153	ICA1	535346	NM_001038168	-2.00319102	-1.134541986	-1.765638508
12901412	FAM26E	101903708	XM_005199378	-2.00406597	-1.773562289	-1.129966499
12682999	LOC100300014	100300014	XM_010801720	-2.006693113	-1.876884666	-1.069161654
12830844	CARS	515715	NM_001103249	-2.006804391	-1.068383796	-1.878355324
12812007	LOC790390	790390	XM_010800284	-2.007931426	1.383555004	-2.778083572
12717216	BPIFB2	618574	NM_001193194	-2.009825156	-1.83711253	-1.09401309
12736694	TGFB2	534069	NM_001113252	-2.012934181	-1.07460764	-1.873180598
12836423	DIRAS3	504559	NM_001034215	-2.015125931	1.599942436	-3.224085492
12782572	LOC101908056	101908056	XM_010802213	-2.015712664	-1.475352814	-1.366258054

ProbeID	Gene_Symbol	Gene_ID	mRNA Accession	CM12H/CM0H.fc	CM12R/CM0H.fc	CM12H/CM12R.fc
12726287			GENSCAN00000020389	-2.016425355	-1.20109478	-1.678822845
12683570	MYLK	338037	NM_176636	-2.016565127	1.323235642	-2.668390851
12809196	SERPINB2	505184 // 281376	NM_001192079	-2.017473887	-1.222191201	-1.650702349
12901337	MARCKS	613548	NM_001076276	-2.020202623	-1.29971022	-1.554348493
12788836	MIR431	100313313	NR_031063	-2.020482702	-2.040340592	1.00982829
12716781	BMP7	540595	NM_001206015	-2.020650768	-1.283692806	-1.574092149
12897460	PHACTR2	522158	XM_002690297	-2.021785579	-1.528810932	-1.322456254
12810552	ATP8B1	541187	NM_001192839	-2.022766794	1.110177223	-2.245629623
12724971	CYC1	512500	NM_001038090	-2.025025395	1.030768247	-2.087331875
12700001	SEPT10	514603	NM_001046176	-2.025193838	-1.154101412	-1.754779795
12755369	FAM83E	100851343	XM_003584863	-2.026345247	-1.345861544	-1.505611967
12879301	LOC101904981	101904981	XM_010798262	-2.027455149	-1.146828768	-1.76787957
12813389	MAPK3	531391	NM_001110018	-2.027525417	1.222055663	-2.477748918
12769620	SPATA20	511363	NM_001083407	-2.030605522	-1.474943816	-1.376734151
12872721				-2.030915198	-2.021813607	-1.004501696
12869377	LOC787600	787600	XM_001254950	-2.034014551	-2.400541704	1.180198885
12902629	LOC517820	517820	XM_010821425	-2.034959384	-1.537716983	-1.323364056
12788826	MIR2284F	100313169	NR_030981	-2.037556408	1.052718714	-2.144973762
12708824	MIR2300B	100313278	NR_031038	-2.043142742	-2.250709719	1.10159201
12738095	LOC100299783	100299783	XM_002694090	-2.04444606	-1.40806116	-1.45195828
12884966			GENSCAN00000034208	-2.046572819	-2.12875512	1.04015606
12819502	SFXN3	511755	NM_001101946	-2.046728868	1.000305031	-2.047353184
12835548	GBP2	514143	XM_010803354	-2.048275818	-1.596320128	-1.283123468
12772679	GAS7	614517	NM_001102280	-2.050633971	-1.480392775	-1.385195878
12802627	LOC528373	528373	XM_002697495	-2.053194072	-1.7951943	-1.143716907
12902249	TCEAL7	100138330 // 100139177	XM_001790056	-2.054418359	-1.292334915	-1.589695005
12749115	LOC617141	617141	XM_010815466	-2.057952951	-2.614055913	1.270221417
12807288				-2.059808191	-1.583733951	-1.300602408
12719139	CASS4	512832	XM_005194419	-2.060922137	-1.892613735	-1.088929082
12719676	SLC17A9	518062	NM_001100378	-2.065784841	-1.034318137	-1.997243176
12845601			ENSBTAT00000065314	-2.066787409	-1.492974992	-1.384341613
12870781	DDIT4L	510906	NM_001081519	-2.067604146	-1.597338418	-1.29440582
12703609	MIR2296	100313121	NR_031090	-2.067962465	-1.645561562	-1.256691037
12731528	LOC508808	508808	XM_002693215	-2.069683263	-2.000610063	-1.034526069
12688814	IPO4	510372	NM_001083661	-2.070529848	-1.080687948	-1.915936837
12786958	ITGA2	281872	NM_001166499	-2.071391136	-1.238771417	-1.67213346
12910823	MIR92A-2	791023	NR_031359	-2.072338967	-1.898671081	-1.091468126
12784009	DAPL1	574085	NM_001025346	-2.07243952	-1.663497848	-1.245832402
12687068	LOC618840	618840	XR_804063	-2.074782347	-2.270090793	1.094134426
12803265	LOC616819	616819	XM_010818465	-2.075213831	-1.023584354	-2.027398937
12851861	GSAP	615147	NM_001193061	-2.07640807	-1.432256614	-1.449745841
12875191	SCFD2	535130	NM_001078005	-2.077574195	-1.03662206	-2.004177102
12713997	LOC407163	407163	XM_005200225	-2.077660601	-1.924214992	-1.079744524
12771970	KRT35	536311	NM_001076073	-2.077891034	-1.60198429	-1.29707329
12750214	LOC506634	506634	XM_003587272	-2.078942707	-1.431085631	-1.452703222
12754716	BOSTAUV1R414	787174	XM_010815328	-2.079879577	-1.887883841	-1.101698914
12889336	GRHPR	504764	NM_001192037	-2.081523722	1.281123893	-2.666689773
12800654	FAM19A1	782935	NM_001099727	-2.08262054	-2.253472743	1.082037126
12796196	WNT5A	530005	NM_001205971	-2.088692337	-1.412088001	-1.479151678
12732069	LOC513948	513948	XM_002693766	-2.090415895	-1.599011151	-1.307317897
12696603	GREM1	539079	NM_001082450	-2.09372215	-1.416872559	-1.477706754
12772448	FAM64A	540455	NM_001099109	-2.099855422	1.119791396	-2.351400035
12896979	MICAL1	508306	NM_001081582	-2.101034716	-1.356059048	-1.549368163
12806768	HIST1H2AH	616634	XM_010818503	-2.101063842	-1.23337371	-1.703509508
12902242	LOC101906965	101906965	XM_010821798	-2.101151225	-1.562330849	-1.344882376
12717610	CENPB	788381	XM_001255460	-2.102957946	-1.036018669	-2.02984561
12753328	FOXL1	538955	XM_005192656	-2.108986704	-1.570789653	-1.34262834
12845168	SLC50A1	520463	XM_003581932	-2.109293713	1.57296874	-3.317853074
12828476	TSNAX	533927	NM_001076006	-2.110609969	1.063330442	-2.244275831
12773167	RAC3	619066	ENSBTAT00000031159	-2.112864136	-2.1138749	1.000478386
12846849	LOC786781	786781	XM_001254364	-2.114080042	-1.471329122	-1.436850538
12723720	LOC782052	782052	XR_811321	-2.11608855	-2.107525373	-1.004063143
12874623	HNRNPD	527471	NM_001098064	-2.116807385	1.096655201	-2.321407829
12806766	LOC616611	616611	XR_814900	-2.117673245	-1.530825672	-1.383353627
12781514	SFT2D3	514114	XM_005197407	-2.121243149	-1.775308811	-1.194858683
12793839	BRF1	618161	XM_005196352	-2.12200786	1.060246705	-2.249851842
12735269	ELF3	508503	NM_001098909	-2.124465625	-1.74322646	-1.21869744
12863077	PRICKLE1	785152	NM_001102534	-2.12459816	1.185782985	-2.519312347
12791021	MAN2C1	541086	NM_001110085	-2.125835554	-1.836705091	-1.157418011
12703346	MIR181B-2	791012	NR_031367	-2.130482203	-3.606976441	1.69303289

ProbeID	Gene_Symbol	Gene_ID	mRNA Accession	CM12H/CM0H.fc	CM12R/CM0H.fc	CM12H/CM12R.fc
12875311	MAPK10	537631	NM_001083728	-2.135819902	-2.299276551	1.076531101
12886108	CHSY3	528149	NM_001192652	-2.145167052	-1.507251327	-1.423231158
12798047	SLC6A6	282366	NM_174610	-2.146862807	-1.349588888	-1.590753174
12715724	LOC524176	524176	XM_005192491	-2.149036521	-1.001192924	-2.146475938
12736336	LOC525100	525100	XM_002694074	-2.153689097	-2.050363924	-1.050393577
12869667	SLC39A8	508193	NM_001205630	-2.154659651	-1.842225925	-1.169595771
12848566	LOC100299757	100299757	XM_005205556	-2.155182438	-1.079969074	-1.995596439
12693052			GENSCAN00000040356	-2.164944439	-1.660272442	-1.303969387
12876554	C7H5orf63	100335582	XM_002688977	-2.167421887	-1.679905408	-1.29020472
12719715	LOC519069	519069	XM_002692063	-2.179655301	-1.232672883	-1.768234972
12873877			GENSCAN00000015031	-2.180698018	-1.077882561	-2.023131366
12831879	SLC36A4	616864	NM_001282523	-2.182467242	-1.166591931	-1.870806051
12682761	KRTAP10-8	783679	NM_001101290	-2.182663912	-1.319663404	-1.65395502
12732213	SLC43A3	516840	NM_001038126	-2.189376392	1.683308962	-3.685396902
12730326	LOC784603	784603	XM_005194618	-2.189831707	-1.356754788	-1.614021728
12807081	LOC785277	785277	XM_001253348	-2.190120122	-1.819194755	-1.203895359
12865056	SLC35E3	509009	NM_001083654	-2.191957764	-1.180002569	-1.857587282
12841606	NEGR1	781106	NM_001205414	-2.193553662	-1.796040646	-1.221327405
12734210			GENSCAN00000001426	-2.194085886	-1.970606881	-1.113406183
12765029	PYCR1	539606	NM_001014957	-2.198607387	-1.445681755	-1.520810081
12802519	HIST1H2BB	525512 // 614958	XM_002697525	-2.198714067	-1.241410282	-1.771142143
12753547	LONP2	541085	NM_001034723	-2.199339009	1.336509414	-2.939437291
12811184	MIR940	100313089	NR_031219	-2.199979377	-1.085417444	-2.02685095
12683071	LSAMP	100336456	NM_001205368	-2.207678362	-1.228297473	-1.797348288
12692745	GPR65	788507	NM_001101303	-2.210097475	-1.434979374	-1.540159751
12687982	GLCE	281195	NM_174070	-2.211936547	1.161798603	-2.56982479
12757460	DDX19B	517438	NM_001046230	-2.212411889	1.26111514	-2.790106129
12745282	LIF	280840	NM_173931	-2.215972534	1.108101473	-2.455522428
12726440	LOC100300167	100300167	XM_010812313	-2.216295117	1.082794902	-2.399793053
12686720	TBC1D5	614554	NM_001205489	-2.218354606	1.306375826	-2.898004831
12720693	GDF5	539559	NM_001192273	-2.218862087	-1.841102566	-1.205181139
12689502	PTGDR	515331	NM_001098034	-2.22257176	1.071067943	-2.380525363
12852736	MIR320B	100313207	NR_030877	-2.232515203	1.307834512	-2.919760432
12801577	HIST1H1D	509275	NM_001101066	-2.233784482	2.066171488	-4.615381808
12809644	SERPINB8	513825	NM_001035287	-2.23437293	-1.443949213	-1.547404098
12786562	LOC614206	614206	XM_005196157	-2.234481345	-1.553669884	-1.438195699
12817585	MYH11	530050	NM_001102127	-2.238589501	-1.986074856	-1.127142562
12869024				-2.241213364	-1.331986998	-1.682609039
12726408	FJX1	100297910	XM_003584883	-2.246174482	-1.135816677	-1.977585404
12893945	LOC100335490	100335490	XM_002704930	-2.246688327	-2.012376155	-1.116435574
12901874			XM_010800841	-2.250335333	-1.874180625	-1.200703552
12709498	VPS36	511223	NM_001098939	-2.251021755	-1.228493309	-1.832343521
12765398	PRR11	615857	NM_001098137	-2.254394505	1.458302487	-3.287589112
12730037	IL18BP	617470	XM_002693432	-2.261891928	-1.430312116	-1.581397446
12756039	MT1A	404071	NM_001040492	-2.267998993	-1.886968056	-1.201927604
12844465	MFSD2A	512633	NM_001101959	-2.274106785	-1.002887654	-2.26755886
12884704	LOC510252	510252	XR_801371	-2.27497391	-1.077658445	-2.111034271
12809133	BCL2	281020	NM_001166486	-2.279361895	1.445631653	-3.295117703
12694003	EID1	506056	NM_001098377	-2.279851727	-1.279854671	-1.781336411
12853855	GIMAP7	100125415 // 614871 // 510988 // 530031	NM_001080257	-2.28220755	-1.377402308	-1.656892497
12775434	ATIC	506343	NM_001075254	-2.285769615	1.020545143	-2.332731078
12687143			ENSBTAT00000015298	-2.287877805	-1.980905421	-1.154965694
12892548	TRPM3	100337068 // 540699	XM_010807877	-2.295470673	-1.290714573	-1.778449489
12756033	MT2A	404070	NM_001075140	-2.296743905	-1.536406529	-1.494880333
12878157	PTTG1	100848911 // 508043	XM_003582470	-2.297938201	1.252759953	-2.878764954
12780762	DNPEP	506882	NM_001045952	-2.29961126	1.109638693	-2.551737632
12778346	CCNYL1	538167	XM_002685539	-2.29965908	-1.022067161	-2.250007796
12693599	PCK2	282856	NM_001205594	-2.304541903	-1.166988222	-1.974777346
12738957	SCCPDH	507289	NM_001034288	-2.305995983	1.30291232	-3.004510576
12730578	MIR2284D	100313461	NR_031104	-2.311036427	-1.582680452	-1.460204064
12713419	MIR296	100313259	NR_031141	-2.320281748	-1.996246669	-1.162322164
12870641	SLC39A8	508193	NM_001205630	-2.324128763	-2.04338351	-1.137392345
12718209	TRDMT1	353353	NM_181812	-2.326675479	-1.485130801	-1.566646842
12678443	LOC100337296	100337296	ENSBTAT00000065610	-2.332585559	-1.57143217	-1.48436923
12721303	DEFB122A	785417	NM_001102339	-2.34565364	-1.935800126	-1.211723054
12825663	LOC789175	789175	NM_001114862	-2.353389312	-3.077417114	1.30765322
12734691	PFKFB2	287019	NM_174812	-2.358353526	1.322960512	-3.120008588
12822288	ACTA2	515610	NM_001034502	-2.358942087	-1.784302226	-1.322052987
12748263				-2.362836822	-1.563533359	-1.511216124
12807161				-2.363983556	-1.287301511	-1.836386842

ProbeID	Gene_Symbol	Gene_ID	mRNA Accession	CM12H/CM0H.fc	CM12R/CM0H.fc	CM12H/CM12R.fc
12724247	FABP4	281759	NM_174314	-2.366737988	-3.750778323	1.584788152
12770990	HS3ST3A1	521959	NM_001192548	-2.366820014	-1.677647948	-1.410796596
12722803	TNFRSF11B	523822	NM_001098056	-2.374460901	-1.461793983	-1.624347157
12692919			ENSBTAT00000065378	-2.391987748	1.004954372	-2.403838545
12803578			ENSBTAT00000033365	-2.397531882	-1.008072919	-2.378331802
12763571	CACNG4	519331	XM_002696227	-2.39992613	-1.123616722	-2.135893925
12753770	SELV	615288	NM_001163244	-2.412117189	-1.162056327	-2.07573173
12872534	CLRN2	617290	NM_001193151	-2.413538767	-1.605708489	-1.503098965
12876045	NMU	782109	NM_001191153	-2.423429309	-1.502130339	-1.613328249
12805927	HIST1H2AC	506900 // 524808	XM_005196641	-2.440437831	-1.29362548	-1.88651033
12770208	ALDH3A2	513967	NM_001101984	-2.448587922	1.103670867	-2.702435153
12910560	LOC784970	784970	XR_803515	-2.454807676	-2.233258108	-1.099204641
12820507	CYP2C18	535243	NM_001076051	-2.457753111	-2.872964169	1.168939287
12897025	HDDC2	509282	NM_001075401	-2.476649998	-1.568591839	-1.578900218
12733686	C15H11orf70	617430	NM_001046598	-2.486661272	-1.133284436	-2.194207555
12807157			GENSCAN00000024173	-2.494014754	-2.337878573	-1.066785411
12726701	MMP1	281308	NM_174112	-2.500904556	-1.454940344	-1.718905223
12886016	MFSD12	526668	XM_605041	-2.507674333	1.235041911	-3.097082899
12697717	LOC785623	785623	XM_001253583	-2.512267349	-2.02988782	-1.237638516
12829117	LOC100139585	100139585	XM_002699010	-2.523366916	-2.151361551	-1.172916247
12711036	FAM155A	616912	NM_001083776	-2.528444305	-1.163273232	-2.173560119
12752079	PRMT1	520388	NM_001015624	-2.543596802	-1.212201892	-2.098327694
12824843	ANGPT2	282141	NM_001098855	-2.575316952	-2.227059341	-1.156375542
12713105	LOC784887	784887	XM_001253067	-2.576709686	-3.320475837	1.288649573
12842694	PSMD4	282016	NM_001013598	-2.58746617	-1.101981497	-2.348012355
12882777			GENSCAN00000031768	-2.588363073	-1.369215947	-1.890397989
12901355	GRIK2	615226	NM_001193063	-2.590337353	-1.219762273	-2.123641147
12848966	INHBA	281867	NM_174363	-2.610778385	-1.000610156	-2.609186375
12793912	LOC101903487	101903487	XM_010816914	-2.611665266	-2.737464015	1.048168022
12700766	IL36A	523429	XM_003582787	-2.617627845	-1.471471908	-1.778917987
12848624			GENSCAN00000029973	-2.620677815	-2.673445006	1.02013494
12728368	LOC521645	521645	XM_002693606	-2.625532402	-2.513225285	-1.04468645
12783344	UBXN4	536181	NM_001035414	-2.635615653	-1.158140271	-2.275730944
12804914	LOC505183	505183	XM_002697515	-2.638064795	-1.216089997	-2.169300628
12842602	S1PR1	281135	NM_001013585	-2.655972115	-1.712756428	-1.550700421
12903604	RBM3	509771	NM_001303463	-2.658864026	-1.517230207	-1.752446013
12777867	KCNH7	534542	XM_005202503	-2.669981971	1.173941079	-3.134401515
12726322	LOC101905705	101905705	XM_005216146	-2.674594169	-1.722889291	-1.552388876
12863669	LOC101905711	101905711	XM_010805440	-2.675447093	-1.789615926	-1.494983954
12689969	MYEF2	524920	NM_001102085	-2.682391841	1.359211534	-3.645937929
12697362	CLN6	617615	NM_001109984	-2.712061699	1.138031113	-3.086410593
12787048	FST	327681	NM_175801	-2.712494101	-1.268919018	-2.137641616
12824802	DEFB1	281743	ENSBTAT00000009756	-2.727917474	-2.572765561	-1.0603055
12821454	NT5C2	281951	NM_174405	-2.730092816	-1.438983451	-1.897237119
12831187	LOC525938	525938	XM_002699398	-2.738868499	-2.559975494	-1.069880749
12692472			GENSCAN00000022855	-2.740710604	-3.041514749	1.10975407
12794223	MIR138-1	100313000	NR_030863	-2.741641623	-2.121743121	-1.292164728
12723468	C14H8orf37	614744	XM_002692813	-2.75833943	1.496010188	-4.126503889
12803695	LOC104975684	104975684	XM_010818515	-2.773158354	-1.415616026	-1.958976376
12899445	CTGF	281103	ENSBTAT00000008357	-2.782381027	1.50067337	-4.175445111
12806854	HIST1H1E	515957 // 617854	XM_002697518	-2.785777433	1.104803657	-3.077737096
12892357			GENSCAN00000021974	-2.815888092	-1.686310262	-1.669851721
12879015	LOC514002	514002	XR_801241	-2.830898454	-2.719176879	-1.041086542
12788824	MIR2366	100313168	NR_030949	-2.835847593	-3.516621345	1.240060063
12732697	SLC35F2	533008	XM_002692963	-2.838109003	-1.473819656	-1.925682692
12718026	THBD	281529	NM_001166522	-2.923628495	-1.065558647	-2.743751836
12765414	GDPD1	615890	NM_001076400	-2.929023971	1.387002127	-4.062562477
12850085	SEMA3C	512660	NM_001101082	-2.941026941	-1.268171623	-2.319107988
12786382	LOC787920	787920	XM_001255135	-2.950133007	-1.829221777	-1.612780388
12825711	LOC100298658	100298658	XM_002698957	-2.961134455	-2.476907513	-1.195496578
12862577	H1F0	617975	NM_001076487	-3.008365798	1.221039609	-3.673333797
12873900	HERC3	510924	NM_001083663	-3.027086707	-1.424928965	-2.124377272
12814450	GBAS	767938	NM_001077011	-3.028975691	-1.101340062	-2.750263789
12794248	ZBTB47	100335506	XM_002702725	-3.198310487	-1.51077624	-2.116998137
12743103	SLC7A11	524078	XM_010813777	-3.328794939	-2.044516917	-1.6281572
12893440	LOC101903205	101903205	XR_801688	-3.562080202	-3.644573511	1.023158746
12802001	HIST1H1C	513971	ENSBTAT00000015499	-3.685831196	1.720729115	-6.342317054
12848557			ENSBTAT00000065831	-4.074378473	-4.378692081	1.074689578
12712983	FAM155A	616912	NM_001083776	-4.183324755	1.273748105	-5.328501979
12848835	IL6	280826 // 517016	NM_173923	-4.325449305	-1.694970307	-2.5519322

ProbeID	Gene_Symbol	Gene_ID	mRNA Accession	CM12H/CM0H.fc	CM12R/CM0H.fc	CM12H/CM12R.fc
12792419	SLC25A21	513423	NM_001015587	-4.341459122	-2.768433754	-1.568200473
12732324			ENSBTAT00000065837	-5.083421996	-2.743865948	-1.852649544
12755458	MIR2330	100313147	NR_030854	-9.466427007	-2.010173462	-4.709258771
