# Supplementary material for: Establishment and characterization of Hanwoo cumulus cell line for heat stress studies
Source: Anim Biosci. 2026 Jun 15;39(7):250896. doi: 10.5713/ab.250896 (PMC13353149; doi:10.5713/ab.250896)
Supplement: Supplementary file 4 [file ab-250896-Supplementary-4.pdf]

Supplement 4. Proteomics cumulus cells normalized raw data

Accession	Gene_Symbol	Gene_ID	Ensembl_Gene ID	log2(ratio(CM12H/CM0H))	log2(ratio(CM12R/CM0H))	log2(ratio(CM12H/CM12R))
A0AAA9TCQ9	AHNAK	A0AAA9TCQ9	ENSBTAG00000013468	0.010829933	0.065277717	0.054447784
E1BF59	PLEC	E1BF59	ENSBTAG00000011922	-0.017785939	-0.058270605	-0.040484665
A0A3Q1NIP5	PLEC	A0A3Q1NIP5	ENSBTAG00000011922	-0.004543925	-0.084064265	-0.07952034
A0A3Q1M2E4	PLEC	A0A3Q1M2E4	ENSBTAG00000011922	-0.047708009	-0.161569279	-0.11386127
A0A3Q1M6Q7	PLEC	A0A3Q1M6Q7	ENSBTAG00000011922	0.228234523	0.027987202	-0.200247321
E1BDX8	DYNC1H1	E1BDX8	ENSBTAG00000016598	-0.022847069	-0.046061782	-0.023214714
A0AAA9S484	MACF1	A0AAA9S484	ENSBTAG00000021024	-0.115731146	-0.091181007	0.024550139
A0AAA9RW41	TLN1	A0AAA9RW41	ENSBTAG00000025868	-0.078739521	-0.108616562	-0.029877041
F1MQ37	MYH9	F1MQ37	ENSBTAG00000010402	-0.495759513	-0.156155973	0.33960354
F1N169	FLNA	bta:281165; F1N169	ENSBTAG00000011190	-0.029211921	-0.059027576	-0.029815654
E1BKX7	FLNB	bta:613533; E1BKX7	ENSBTAG00000022004	0.015724352	0.022225918	0.006501566
E1BFB0	SPTAN1	E1BFB0	ENSBTAG00000015327	0.001690328	-0.123533434	-0.125223762
A0A3Q1NKL1	SPTAN1	A0A3Q1NKL1	ENSBTAG00000015327	0.023541001	-0.197739945	-0.221280946
A0A3Q1LV98	FLNC	A0A3Q1LV98	ENSBTAG00000006253	0.134824424	0.156119202	0.021294778
A0A3Q1MXU7	SPTBN1	A0A3Q1MXU7	ENSBTAG00000006995	0.020225028	-0.083860605	-0.104085633
Q27991	MYH10	bta:317655; Q27991		-0.440474359	0.008228321	0.44870268
A0A3Q1N191	GCN1	A0A3Q1N191	ENSBTAG00000017379	-0.096264718	-0.058475889	0.03778883
A0A3Q1MTY6	MAP1B	A0A3Q1MTY6	ENSBTAG00000001961	0.077489371	0.057333175	-0.020156196
F1N7I5	NUMA1	F1N7I5	ENSBTAG00000018449	0.001547126	0.073923182	0.072376055
A0AAA9T5L8	GOLGB1	A0AAA9T5L8	ENSBTAG00000008862	0.176697007	0.189116266	0.012419259
A0A3Q1M9Z4	MAP1A	A0A3Q1M9Z4	ENSBTAG00000014001	0.08482489	0.012268837	-0.072556053
A0A3Q1LMV9	EPPK1	A0A3Q1LMV9	ENSBTAG00000045727	-0.039966407	-0.007905198	0.032061209
A0A3Q1LLS4	RANBP2	A0A3Q1LLS4	ENSBTAG00000000152	0.014619789	-0.026967048	-0.041586837
A0AAA9SF14	CLTC	A0AAA9SF14	ENSBTAG00000016708	0.082174513	0.09838474	0.016210227
E1BGJ0	LRP1	E1BGJ0	ENSBTAG00000010830	-0.090148353	-0.088517268	0.001631086
A0A3Q1MIB0	IQGAP1	A0A3Q1MIB0	ENSBTAG00000013060	0.003765191	0.033538115	0.029772923
A0AAA9RXD6	RRBP1	A0AAA9RXD6	ENSBTAG00000015438	0.181622816	0.096455432	-0.085167383
F1MQ21	NES	F1MQ21	ENSBTAG00000015794	0.068537843	0.096942928	0.028405086
A0AAA9S236	MKI67	A0AAA9S236	ENSBTAG00000002444	0.197577373	0.166470195	-0.031107178
A0AAA9TU63	UBR4	A0AAA9TU63	ENSBTAG00000015240	-0.112187147	-0.044774226	0.067412921
A0A3Q1LIB4	VCL	A0A3Q1LIB4	ENSBTAG00000021879	-0.042739433	-0.027428489	0.015310945
A0AAA9T4R9	TPR	A0AAA9T4R9	ENSBTAG00000060237	-0.022346159	-0.016727086	0.005619073
E1BNR0	APOB	E1BNR0	ENSBTAG00000008505	-0.553294585	-0.104116945	0.44917764
A0AAA9TDR0	HSPG2	A0AAA9TDR0	ENSBTAG00000017122	-0.497667718	-0.504406147	-0.006738429
F1N647	FASN	F1N647	ENSBTAG00000015980	-0.145197916	-0.217980861	-0.072782946
A0AAA9SFW6	KTN1	A0AAA9SFW6	ENSBTAG00000017713	-0.022888291	-0.030599133	-0.007710842
A0AAA9TMZ2	EPRS1	A0AAA9TMZ2	ENSBTAG00000006898	0.022855053	0.052313146	0.029458093
A0AAA9SKJ1	A2M	A0AAA9SKJ1	ENSBTAG00000018137	-0.143503824	0.376639209	0.520143033
G3X757	VCP	G3X757	ENSBTAG00000015000	0.074072284	-0.010073722	-0.084146006
F1N3I4	MYOF	F1N3I4	ENSBTAG00000016918	-0.065792314	-0.031026896	0.034765418
A0A3Q1MSB2	AKAP12	A0A3Q1MSB2	ENSBTAG00000014788	0.048094288	0.084423061	0.036328773
B8Y9S9				-0.44366633	-0.568643925	-0.124977595
A0A3Q1MPS4	ACTN1	A0A3Q1MPS4	ENSBTAG00000018255	-0.066923552	-0.105171022	-0.038247469
P48616	VIM	bta:280955; P48616; Q17QM7	ENSBTAG00000018463	0.044167583	-0.068895081	-0.113062664
A0A3Q1M820	SACS	A0A3Q1M820; bta:539378	ENSBTAG00000001867	-0.448888858	-0.314991323	0.133897536
F1MRT9	UTRN	F1MRT9	ENSBTAG00000009665	-0.031346237	-0.003267714	0.028078523
A0A3Q1MYL9	MAP4	A0A3Q1MYL9	ENSBTAG00000015839	0.062702499	0.095256999	0.0325545
A0AAA9SGA6	HUWE1	A0AAA9SGA6	ENSBTAG00000006122	-0.019011969	-0.032322422	-0.013310454
Q3SYU2	EEF2	bta:281138; Q3SYU2	ENSBTAG00000004258	-0.159074123	-0.220112136	-0.061038013
Q76LV1	HSP90AB1	bta:767874; Q76LV1	ENSBTAG00000000778	0.369433532	0.173360809	-0.196072723
Q95M18	HSP90B1	bta:282646; Q3MHX8; Q95M18	ENSBTAG00000003362	0.559178475	0.486422133	-0.072756342
A0AAA9SDV9				-0.740424778	-1.408358842	-0.667934065
F1MD83				-0.821662759	-0.743912532	0.077750226
A0A140T856	COPA	A0A140T856	ENSBTAG00000004333	0.10557337	0.159805682	0.054232312
E1BPX9	LRPPRC	E1BPX9	ENSBTAG00000016368	-0.31937239	-0.337034987	-0.017662597
E1B7R4	EIF3A	E1B7R4	ENSBTAG00000002528	-0.005418579	-0.083496493	-0.078077914
A7Z025	PRPF8	A7Z025; bta:507371	ENSBTAG00000011488	-0.095443485	-0.062922343	0.032521142
E1BBY7	HSPA4	bta:536558; E1BBY7	ENSBTAG00000015683	0.209411929	0.24089016	0.031478231
E1BH78	SNRNP200	bta:510529; E1BH78	ENSBTAG00000000848	-0.069953534	-0.065385631	0.004567904
Q2HJ49	MSN	bta:540426; Q2HJ49	ENSBTAG00000003418	0.058181071	0.066709342	0.008528271
P08169	IGF2R	bta:281849; P08169		0.055684133	0.121629444	0.065945312
A0AAA9T3C7				-0.671479228	-0.278535499	0.392943728
A0A3Q1NND0	HDLBP	A0A3Q1NND0	ENSBTAG00000004315	-0.0415482	-0.134204425	-0.092656225
E3W9A2	CLIP1	bta:534624; E3W9A2	ENSBTAG00000016779	0.236720052	0.226469956	-0.010250097
A0A3Q1LQZ3	CLIP1	A0A3Q1LQZ3	ENSBTAG00000016779	0.164248379	0.181542303	0.017293924
A0A140T897	ALB	A0A140T897; bta:280717	ENSBTAG00000017121	-0.50003124	0.257339387	0.757370627
A0AAA9U1R4	TPM1	A0AAA9U1R4	ENSBTAG00000005373	-0.245859262	-0.184031948	0.061827314
Q3ZC87	PKM2	Q3ZC87		0.073704005	0.193941883	0.120237877
F1N3F6	CHD4	F1N3F6	ENSBTAG00000014734	0.033980649	-0.037738642	-0.071719291
A0A3Q1MH82	LMO7	A0A3Q1MH82	ENSBTAG00000010693	0.143143117	0.099535674	-0.043607443
A0AAA9SJJ8	CAD	A0AAA9SJJ8	ENSBTAG00000017894	-0.00293828	-0.097126555	-0.094188275
F1MX04	EIF4G1	F1MX04	ENSBTAG00000012881	-0.08419835	-0.15707723	-0.07287888
F1MPE5	IPO5	F1MPE5	ENSBTAG00000039374	-0.058230228	-0.080511676	-0.022281447
F1MLW0	CALD1	F1MLW0	ENSBTAG00000013953	-0.009126886	0.002597111	0.011723997
A5D7D1	ACTN4	A5D7D1; bta:522269	ENSBTAG00000011215	-0.001332744	-0.01203925	-0.010706506
A0AAA9SIS5	LMNA	A0AAA9SIS5	ENSBTAG00000017574	0.126270861	0.031377815	-0.094893047
A0AAA9TE87	UGGT1	A0AAA9TE87	ENSBTAG00000005444	-0.022767885	0.025376563	0.048144448
A5PKJ3	DCTN1	A5PKJ3; bta:511045		0.021936992	0.007349456	-0.014587536
A0JN39	COPB1	A0JN39; bta:535533	ENSBTAG00000006556	0.029287227	0.09011242	0.060825193
G5E641				-0.780387874	-0.333151553	0.447236322
P02453	COL1A1	bta:282187; P02453; Q3MHM2	ENSBTAG00000013103	-0.548242507	-1.566283948	-1.01804144
P56701	PSMD2	A7E3R5; bta:539784; P56701; Q2HJE3; Q9TRA7	ENSBTAG00000005660	0.004262027	-0.024392243	-0.028654269
A0A3Q1MVZ1	PARP1	A0A3Q1MVZ1	ENSBTAG00000000837	0.125913306	0.125913306	0
A0A3Q1MJM1	HYOU1	A0A3Q1MJM1	ENSBTAG00000010322	0.340107537	0.170816269	-0.169291268
A0A3Q1LJA4	DCTN1	A0A3Q1LJA4	ENSBTAG00000004887	-0.330325826	-0.363456419	-0.033130593
A0AAF7A102	ANXA6	A0AAF7A102	ENSBTAG00000014809	-0.00280135	0.040019932	0.042821283
A0A3Q1MHX1	ECPAS	A0A3Q1MHX1	ENSBTAG00000006712	0.01647101	-0.022767885	-0.039238895
E1BKE7	ITPR3	E1BKE7	ENSBTAG00000013245	-0.159711189	-0.158280653	0.001430536
A0AAF6Z0Y8	ANXA6	A0AAF6Z0Y8	ENSBTAG00000014809	0.05125812	0.062105502	0.010847382

Accession	Gene_Symbol	Gene_ID	Ensembl_Gene ID	log2(ratio(CM12H/CM0H))	log2(ratio(CM12R/CM0H))	log2(ratio(CM12H/CM12R))
A0A3Q1M9W3	AFDN	A0A3Q1M9W3	ENSBTAG00000005557	0.033007106	0.022665162	-0.010341944
A0AAA9SCC2	DST	A0AAA9SCC2	ENSBTAG00000021237	-0.186365655	-0.120533665	0.06583199
F2Z4J5	DNMT1	F2Z4J5	ENSBTAG00000002736	-0.07019543	-0.16411938	-0.093923949
F1MDU7	TOP2A	bta:787696; F1MDU7	ENSBTAG00000019262	-0.056301596	-0.028475169	0.027826427
E1BKX3	MYBBP1A	bta:513045; E1BKX3	ENSBTAG00000007430	-0.01449957	-0.094859186	-0.080359617
A7MBI5	DPYSL3	A7MBI5; bta:509796	ENSBTAG00000010661	0.064911944	0.060939399	-0.003972545
A0AAA9S4P4	DST	A0AAA9S4P4	ENSBTAG00000021237	-0.0959833	-0.104639496	-0.008656196
A0AAA9T5V4	AARS1	A0AAA9T5V4	ENSBTAG00000019810	-0.05267454	-0.007006789	0.045667751
A0AAA9TRW0	CALD1	A0AAA9TRW0	ENSBTAG00000013953	0.186706307	-0.065318338	-0.252024646
F1MM34	CTNNA1	F1MM34	ENSBTAG00000014182	0.222392421	0.037139136	-0.185253285
F1N1G7	KIF5B	F1N1G7	ENSBTAG00000002280	-0.007231569	-0.043943348	-0.036711778
G3N0R5	HSPD1	G3N0R5	ENSBTAG00000012586	0.217387403	0.212364961	-0.005022442
F1N401	COL12A1	F1N401	ENSBTAG00000020056	-0.475931908	-0.856063792	-0.380131884
A0AAA9T7M1	EEA1	A0AAA9T7M1	ENSBTAG00000000421	0.05920358	0.097364575	0.038160995
A0A140T886	COPG1	A0A140T886; bta:338055	ENSBTAG00000013212	-0.111369814	0.071530515	0.182900329
A0AAA9SJL9	PARP1	A0AAA9SJL9	ENSBTAG00000000837	0.087914658	0.158441654	0.070526996
V9GW27				-0.087728849	-0.001419277	0.086309572
A0AAA9SHQ7	UBA1	A0AAA9SHQ7	ENSBTAG00000016764	0.068243176	0.050358297	-0.017884878
A0AAA9SYD6	CENPF	A0AAA9SYD6	ENSBTAG00000024449	0.153283497	0.19584396	0.042560462
Q28141	DHX9	bta:281115; Q28141		-0.078387899	-0.004167634	0.074220264
Q29443	TF	bta:280705; Q0IIK2; Q29443		-0.080489918	-0.036812666	0.043677252
A7MBA2	PSMD1	A7MBA2; bta:511803	ENSBTAG00000005119	-0.03487046	0.027020214	0.061890674
G3X6N3	TF	G3X6N3	ENSBTAG00000007273	-0.299907487	0.329430426	0.629337913
A0A3Q1MW77	ROCK2	A0A3Q1MW77	ENSBTAG00000005847	0.158827293	0.10491501	-0.053912282
A0A3Q1LW78	STIP1	A0A3Q1LW78	ENSBTAG00000015663	0.303254254	0.296758543	-0.006495711
F1MD77	LAMC1	F1MD77	ENSBTAG00000011966	0.031507801	-0.545434137	-0.576941938
A0A3Q1N4H7	TNKS1BP1	A0A3Q1N4H7; bta:783548	ENSBTAG00000016469	-0.068108387	-0.027656343	0.040452044
A0A3Q1LMS5	HSPA8	A0A3Q1LMS5	ENSBTAG00000013162	0.436509477	0.257254159	-0.179255317
A0A3Q1MJD5	HNRNPM	A0A3Q1MJD5; bta:515290	ENSBTAG00000021039	0.036957756	0.022855053	-0.014102703
F1MYX5	LCP1	F1MYX5	ENSBTAG00000007079	0.028314282	-0.010435453	-0.038749734
A0AAA9S1P6	MCM4	A0AAA9S1P6	ENSBTAG00000017021	0.064567168	0.041040684	-0.023526484
Q9XSX1				0.028041603	0.018288919	-0.009752683
B0JYQ0	ALB	B0JYQ0		-0.396477517	0.247310054	0.64378757
A0AAA9RTW0	SMCHD1	A0AAA9RTW0	ENSBTAG00000003354	-0.251736383	-0.116630359	0.135106024
F1MHU9	CNOT1	F1MHU9	ENSBTAG00000020782	-0.052626931	-0.058594654	-0.005967723
A0AAF6ZGM3	TPM2	A0AAF6ZGM3	ENSBTAG00000011424	-0.171938131	-0.129821973	0.042116159
F1MEN1				-0.824632913	-0.756728849	0.067904065
Q0VCX2	HSPA5	A7E3V5; bta:415113; Q0VCX2	ENSBTAG00000007662	0.52122641	0.340351918	-0.180874492
E1BMZ9	SMC4	E1BMZ9	ENSBTAG00000005862	-0.317615102	-0.226718347	0.090896755
Q3ZBH0	CCT2	bta:505313; Q3ZBH0	ENSBTAG00000019156	0.122436076	0.095683348	-0.026752728
A0A3Q1MQV3	THBS1	A0A3Q1MQV3	ENSBTAG00000002006	-0.544619613	-0.505374236	0.039245377
A0A3Q1LZN8	COL1A2	A0A3Q1LZN8	ENSBTAG00000013472	-0.363420224	-1.584219035	-1.220798811
F2Z4C6	EIF4G2	F2Z4C6	ENSBTAG00000020308	0.00284275	0.014158063	0.011315313
A0A3Q1M168	PYGL	A0A3Q1M168	ENSBTAG00000011494	0.033063208	0.129326451	0.096263243
A0AAA9TXI1	NUP153	A0AAA9TXI1; bta:513270	ENSBTAG00000017128	0.22059914	0.157324965	-0.063274175
E1BLD1	HECTD1	bta:536240; E1BLD1	ENSBTAG00000032477	0.131514181	-0.172408126	-0.303922307
A4FV23	DDX21	A4FV23; bta:781917		-0.159899491	-0.254172468	-0.094272976
A0AAA9T089	UPF1	A0AAA9T089	ENSBTAG00000020922	0.072034789	0.095758983	0.023724194
A8E4L8	LMO7	A8E4L8; F1N6J9		0.169301133	0.142428272	-0.026872862
E1BKT0	LUZP1	bta:516426; E1BKT0	ENSBTAG00000009502	0.063846482	0.029081064	-0.034765418
A0AAA9RYK8	EIF5B	A0AAA9RYK8	ENSBTAG00000034255	0.046778405	0.046778405	0
Q863B3	SND1	bta:404098; Q3MHZ9; Q863B3	ENSBTAG00000010692	0.034689421	0.033130593	-0.001558828
A0AAA9TE63	MATR3	A0AAA9TE63	ENSBTAG00000006756	-0.002834372	0.015490286	0.018324658
A0AAA9TK25	IARS1	A0AAA9TK25	ENSBTAG00000000974	-0.148452438	-0.065453061	0.082999377
Q0VCK0	ATIC	bta:506343; Q0VCK0		-0.017841313	-0.04918755	-0.031346237
A7Z065	SMC3	A7Z065	ENSBTAG00000013905	0.070954647	0.061029433	-0.009925214
Q2UVX4	C3	bta:280677; Q2KIZ4; Q2UVX4	ENSBTAG00000017280	-0.413627929	0.012636826	0.426264755
A0A3Q1LJT3	GLG1	A0A3Q1LJT3	ENSBTAG00000002303	0.016965402	0.0388541	0.021888698
A4FUD0	MTHFD1	A4FUD0; bta:534382	ENSBTAG00000009641	-0.136075819	-0.099442923	0.036632897
F1MEN8	PDIA4	F1MEN8	ENSBTAG00000017143	0.571970481	0.534714311	-0.03725617
Q3ZCH0	HSPA9	A5D9A6; bta:517535; Q3ZCH0	ENSBTAG00000011419	-0.013713934	-0.045759661	-0.032045727
G3X6B2	PRRC2C	G3X6B2	ENSBTAG00000001821	-0.030286695	-0.019199647	0.011087048
A0AAA9S4Y0	TJP1	A0AAA9S4Y0	ENSBTAG00000015398	0.197102929	0.169752441	-0.027350488
Q3ZBH9	MCM7	bta:539924; Q3ZBH9	ENSBTAG00000030965	0.01282404	0.019899557	0.007075517
F1MD74	CAST	F1MD74	ENSBTAG00000000874	-0.106651626	-0.003430905	0.10322072
O97593	SMC1A	bta:282370; O97593	ENSBTAG00000017761	0.128048361	-0.049803556	-0.177851917
A0AAF6ZVV1	PC	A0AAF6ZVV1	ENSBTAG00000019700	0.022653184	0.093911868	0.071258683
A0A3Q1LUJ8	ATP1A1	A0A3Q1LUJ8	ENSBTAG00000001246	0.145137191	0.184595801	0.03945861
A5D7A2	GARS1	A5D7A2; bta:408010	ENSBTAG00000018972	-0.034646143	-0.00981429	0.024831853
F1MJK3	LOC506828	F1MJK3	ENSBTAG00000038461	-0.529320927	0.048153022	0.577473949
A0A3Q1MPF1	GDI2	A0A3Q1MPF1	ENSBTAG00000005316	-0.057251176	-0.024478976	0.0327722
A7MBJ5	CAND1	A7MBJ5; bta:538086	ENSBTAG00000009939	0.057333175	0.119394143	0.062060968
A0A3Q1MLL6	PPP1R12A	A0A3Q1MLL6	ENSBTAG00000014609	0.024247546	0.028748938	0.004501392
A0A3Q1MXS2	ITGAV	A0A3Q1MXS2	ENSBTAG00000019929	-0.010492374	0.022229947	0.032722321
A0A3Q1LVC7	EZR	A0A3Q1LVC7	ENSBTAG00000010347	0.072862873	0.110971139	0.038108265
A6QR15	TPM4	A6QR15; bta:535277	ENSBTAG00000004553	-0.003227507	-0.059229551	-0.056002044
F1MX61	SF3B1	bta:510177; F1MX61	ENSBTAG00000008915	0.042393698	0.045176139	0.002782441
Q3T0K2	CCT3	bta:504735; Q3T0K2	ENSBTAG00000006370	0.086335169	0.035156445	-0.051178724
A0A3Q1LTK7	ERC1	A0A3Q1LTK7	ENSBTAG00000013658	0.030282567	0.037399972	0.007117405
Q3ZCI9	CCT8	bta:281047; Q3ZCI9	ENSBTAG00000014233	0.050434722	0.061111514	0.010676793
A0A3Q1N4A7	CIT	A0A3Q1N4A7	ENSBTAG00000008963	-0.139274789	-0.188856817	-0.049582029
F1MF78	SYNE2	F1MF78	ENSBTAG00000025450	-0.067552772	-0.070773074	-0.003220303
F1ME65	CKAP4	bta:515784; F1ME65	ENSBTAG00000011913	0.149912609	0.007812447	-0.142100162
F1MY41	SMC2	bta:539217; F1MY41	ENSBTAG00000008772	-0.1828977	-0.173040302	0.009857398
F1MY84	CSPG4	F1MY84	ENSBTAG00000012219	-0.102974758	-0.146078259	-0.043103502
A0A3Q1M0D6	SRRM2	A0A3Q1M0D6; bta:539515	ENSBTAG00000012683	0.291584867	-0.023060552	-0.314645419
E1BE98	XPO1	bta:510568; E1BE98	ENSBTAG00000013254	0.138063902	0.136662547	-0.001401355
Q3SZ54	EIF4A1	bta:504958; Q3SZ54	ENSBTAG00000000132	-0.200897297	-0.12882262	0.072074677
F1N6Y1	GANAB	bta:540155; F1N6Y1	ENSBTAG00000016956	-0.002999367	0.002993144	0.005992511
F1MNT4	LAMB1	F1MNT4	ENSBTAG00000011412	-0.035929857	-0.402448103	-0.366518246
E1BDU8	LIMA1	bta:540637; E1BDU8	ENSBTAG00000012342	0.090998121	0.162240202	0.071242081

Accession	Gene_Symbol	Gene_ID	Ensembl_Gene ID	log2(ratio(CM12H/CM0H))	log2(ratio(CM12R/CM0H))	log2(ratio(CM12H/CM12R))
A0AAA9SFD3	GOLGA3	A0AAA9SFD3	ENSBTAG00000009755	-0.021226892	-0.002812272	0.01841462
A0AAA9SMJ2	ACTB	A0AAA9SMJ2	ENSBTAG00000026199	0.062894791	-0.001495796	-0.064390587
A0A3Q1M1Q5	CTNND1	A0A3Q1M1Q5	ENSBTAG00000002411	0.010669743	0.015218443	0.004548701
A0A3Q1M5Q7	NASP	A0A3Q1M5Q7	ENSBTAG00000015346	0.04094102	0.103835811	0.062894791
A0AAA9RWG3	TPM2	A0AAA9RWG3	ENSBTAG00000011424	-0.46630617	-0.609911472	-0.143605303
F1N365	FLII	F1N365	ENSBTAG00000016161	-0.070454199	-0.131677061	-0.061222862
A0AAA9RY84	NAA15	A0AAA9RY84	ENSBTAG00000004745	0.005532874	0.040988411	0.035455538
A0JN43	FAM62A	A0JN43; bta:520669; F1N785		-0.063658637	-0.101337254	-0.037678617
F6QQ60	TPM4	F6QQ60	ENSBTAG00000004553	0.020375174	0.066324759	0.045949586
A0AAA9TWX8	DDX3X	A0AAA9TWX8	ENSBTAG00000035907	-0.268801683	-0.196762208	0.072039475
A4FUD9	MCM3	A4FUD9; bta:281302		0.044348626	0.06171327	0.017364643
Q08DS7	AP2B1	bta:282183; Q08DS7	ENSBTAG00000020316	-0.059933646	0.002932308	0.062865954
A0A3Q1LK06	LRRFIP1	A0A3Q1LK06	ENSBTAG00000005354	-0.017214555	-0.195656968	-0.178442413
A0A3Q1MVC6	RAI14	A0A3Q1MVC6	ENSBTAG00000007071	0.160790172	0.024531006	-0.136259165
Q9TRY0	FKBP4	A5D9B2; bta:508535; Q3T0C4; Q9TRX9; Q9TRY0		0.364836686	0.317839207	-0.04699748
A7E3Q8	PLS3	A7E3Q8	ENSBTAG00000011613	-0.028249477	-0.129532813	-0.101283336
A7E3W7	DDX1	A7E3W7	ENSBTAG00000009906	-0.025435047	-0.055687375	-0.030252328
F1MXF3	SYNE1	F1MXF3	ENSBTAG00000009362	-0.008258151	-0.045166316	-0.036908165
A0AAA9TC88	ACO2	A0AAA9TC88	ENSBTAG00000006429	-0.020547377	-0.011705501	0.008841877
A2VDN7	HNRNPU	A2VDN7; bta:539950; F1MFT4	ENSBTAG00000007606	0.038316932	0.071384633	0.033067702
K4JDT2	A2M	K4JDT2		-0.081877007	0.535709769	0.617586776
Q2KJD0	TUBB5	A5D9A3; bta:615087; Q2KJD0	ENSBTAG00000006969	0.139364551	0.115252095	-0.024112456
E1BMW2	AP2A1	E1BMW2	ENSBTAG00000019180	-0.166440008	-0.04324681	0.123193197
Q3T0P6	PGK1	bta:507476; Q3T0P6	ENSBTAG00000000894	0.092841021	0.128628585	0.035787564
Q2NKZ1	CCT7	bta:514355; Q2NKZ1	ENSBTAG00000012756	0.017819738	0.038111142	0.020291404
A0A3Q1M1I5	EIF4G3	A0A3Q1M1I5	ENSBTAG00000040215	0.172399911	0.257450288	0.085050377
A0AAA9SZH0	DNAJC13	A0AAA9SZH0	ENSBTAG00000002966	-0.094307159	-0.010514222	0.083792937
A0AAA9SDW8	GART	A0AAA9SDW8	ENSBTAG00000009188	0.015121287	0.030085726	0.014964439
A0AAA9SZZ0	SEC31A	A0AAA9SZZ0	ENSBTAG00000020525	0.27647278	0.367490672	0.091017892
A0AAF6YPM1	OGDH	A0AAF6YPM1	ENSBTAG00000006029	-0.199824664	-0.06115838	0.138666284
A0A3Q1M0F0	HK1	A0A3Q1M0F0	ENSBTAG00000012380	0.014937442	-0.00250468	-0.017442121
A0AAA9T8D8	PDS5A	A0AAA9T8D8	ENSBTAG00000017809	0.009771557	0.01809482	0.008323264
A0A3Q1LHC9	MCM2	A0A3Q1LHC9	ENSBTAG00000014380	0.013979712	0.044266408	0.030286695
A0AAA9RXY5	SEC31A	A0AAA9RXY5	ENSBTAG00000020525	0.09010389	0.168029779	0.077925889
Q3SYU9	MVP	bta:516456; Q3SYU9		-0.256395764	-0.129661131	0.126734633
A7E3V3	IMMT	A7E3V3		0.015581541	-0.034591748	-0.050173289
F1MIF2	AP1B1	F1MIF2	ENSBTAG00000001189	0.033341075	0.042301943	0.008960868
Q32LP2	RDX	bta:517111; Q32LP2	ENSBTAG00000004672	0.107814484	0.058949661	-0.048864823
E1BE13	BIRC6	E1BE13	ENSBTAG00000027932	-0.365744327	-0.294447358	0.071296969
F1MWN1	CSE1L	F1MWN1	ENSBTAG00000005957	0.005852726	0.108694436	0.102841709
A0A452DII8	ATP5F1B	A0A452DII8	ENSBTAG00000013315	0.044660127	0.040398101	-0.004262027
A5D7D9	DHX15	A5D7D9; bta:512327	ENSBTAG00000008896	-0.052841127	0.03839829	0.091239417
A0AAA9TCS0	ENO1	A0AAA9TCS0	ENSBTAG00000013411	0.140147706	0.186364112	0.046216406
A0A3Q1LYI6	IMMT	A0A3Q1LYI6	ENSBTAG00000003307	-0.018708527	-0.025969212	-0.007260685
A0AAA9T403	SUPT16H	A0AAA9T403	ENSBTAG00000020421	0.024685498	0.062264796	0.037579298
A0AAA9S717	DNM1L	A0AAA9S717	ENSBTAG00000011395	0.112909647	0.118262882	0.005353234
A0AAA9T689	ANLN	A0AAA9T689	ENSBTAG00000009218	-0.142141845	-0.370223652	-0.228081807
A0AAA9T8P4	AP1B1	A0AAA9T8P4	ENSBTAG00000001189	-0.004626498	-0.006171967	-0.001545469
A0A3Q1NE05	KHSRP	A0A3Q1NE05	ENSBTAG00000021018	0.088355874	0.068218229	-0.020137646
A0AAA9TT10	DDB1	A0AAA9TT10	ENSBTAG00000021569	-0.125776971	-0.140619643	-0.014842672
F1MNF8	TUBA1A	bta:404053; F1MNF8	ENSBTAG00000001489	0.040557347	0.046470039	0.005912693
E1B8K6				0.045152036	0.08631778	0.041165744
A0AAA9TV68	TOP1	A0AAA9TV68; bta:534799	ENSBTAG00000007960	0.252423585	0.293925288	0.041501704
A0A3Q1LJL3	OPA1	A0A3Q1LJL3; bta:524142	ENSBTAG00000019514	0.010627636	-0.04193783	-0.052565466
A0A3Q1MEP1	CEP170	A0A3Q1MEP1	ENSBTAG00000007566	0.101988784	0.022244637	-0.079744146
A0A3Q1LVF3	SUPT6H	A0A3Q1LVF3	ENSBTAG00000009036	0.219502985	0.142271555	-0.07723143
Q3SZ00	HADHA	Q3SZ00	ENSBTAG00000015038	-0.05467087	-0.005811468	0.048859401
Q27966-3	MYO1C	Q27966-3		-0.046829132	0.087836645	0.134665777
Q2KJH6	SERPINH1	bta:510850; Q2KJH6	ENSBTAG00000001027	0.381063219	0.31587905	-0.065184169
A0A3Q1NB13	ABCF1	A0A3Q1NB13	ENSBTAG00000006927	0.060001907	0.072541102	0.012539196
O62654	DES	A2VDU8; bta:280765; O62654; O62655	ENSBTAG00000005353	-0.046886388	-0.319832677	-0.272946289
A0AAA9S7W6	USP7	A0AAA9S7W6	ENSBTAG00000005455	-0.045156181	-0.004308699	0.040847482
F1MWD3	CCT5	F1MWD3	ENSBTAG00000017889	0.091289297	0.063708488	-0.027580809
A0A3Q1NGQ6	ALDOA	A0A3Q1NGQ6	ENSBTAG00000012927	0.086054647	0.025115248	-0.060939399
A0AAF6YZT3	MOV10	A0AAF6YZT3	ENSBTAG00000014297	-0.076645958	-0.013636161	0.063009798
A0A3Q1MWL3	NCKAP1	A0A3Q1MWL3	ENSBTAG00000033662	0.051264472	-0.023762573	-0.075027045
F1MH31	NUP214	F1MH31	ENSBTAG00000021896	0.032926005	0.059317504	0.026391499
E1BHU1	TCOF1	bta:513591; E1BHU1	ENSBTAG00000015224	0.099224379	0.036876002	-0.062348377
G3X755	PLXNB2	G3X755	ENSBTAG00000014966	0.056194539	0.062664035	0.006469495
E1BCV4	NUP98	E1BCV4	ENSBTAG00000008432	-0.060502173	-0.005522284	0.054979889
A0A3Q1LZ02	HK2	A0A3Q1LZ02; bta:788926	ENSBTAG00000013108	0.114666942	0.114666942	0
E1BMD6	LPP	bta:524132; E1BMD6	ENSBTAG00000044001	0.088744212	0.145012431	0.05626822
A0A3Q1MMV9	AHCTF1	A0A3Q1MMV9	ENSBTAG00000013949	0.081418794	0.059413486	-0.022005308
A7Z014	TKT	A7Z014; F1MUX4	ENSBTAG00000003758	0.085315247	0.10086168	0.015546433
A0AAA9TM36	NCAPD2	A0AAA9TM36	ENSBTAG00000014730	-0.186151316	-0.204991398	-0.018840082
F1MJI7	LMNB2	bta:516326; F1MJI7	ENSBTAG00000013624	0.159643156	0.023916506	-0.135726649
A0AAA9SH52	TPM3	A0AAA9SH52	ENSBTAG00000033217	0.101920294	0.152718894	0.0507986
A0A3Q1LUN6	GMPS	A0A3Q1LUN6	ENSBTAG00000013013	-0.186190056	-0.100460182	0.085729874
A0AAA9S813	MYO18A	A0AAA9S813	ENSBTAG00000017380	0.019815457	0.070389328	0.050573871
P41541	USO1	bta:317724; P41541; Q32PH6	ENSBTAG00000017028	0.10228105	0.132140339	0.029859289
E1BLV6	VARS1	E1BLV6	ENSBTAG00000005631	-0.076145398	-0.050314465	0.025830933
Q2HJ81	TUBB6	bta:534206; Q2HJ81		-0.090117295	-0.031542469	0.058574826
A0A3Q1ML77	CSDE1	A0A3Q1ML77	ENSBTAG00000000678	-0.28581112	-0.231739296	0.054071823
A0AAA9S5Z7	TPM3	A0AAA9S5Z7	ENSBTAG00000033217	0.121136438	0.217872224	0.096735787
A0AAA9S0P0	LOC100141266	A0AAA9S0P0	ENSBTAG00000056851	-0.136273979	-0.223857533	-0.087583554
E1BKM4	PDCD6IP	E1BKM4	ENSBTAG00000017426	0.091017892	0.059275809	-0.031742083
A0AAA9SNY0	RARS1	A0AAA9SNY0	ENSBTAG00000014626	-0.00904514	0.041476636	0.050521776
A0A3Q1LXE1	PPFIBP1	A0A3Q1LXE1; bta:407130	ENSBTAG00000004283	-0.099318056	-0.040163269	0.059154787
E1BN47	CYFIP1	bta:100141021; E1BN47	ENSBTAG00000016894	-0.06630237	0.006805178	0.073107548

Accession	Gene_Symbol	Gene_ID	Ensembl_Gene ID	log2(ratio(CM12H/CM0H))	log2(ratio(CM12R/CM0H))	log2(ratio(CM12H/CM12R))
A0AAF6DM79	SAFB	A0AAF6DM79	ENSBTAG00000007875	0.085701094	0.038395379	-0.047305715
F1N7G8	PDS5B	F1N7G8	ENSBTAG00000011597	-0.027207959	-0.043197689	-0.015989731
A0AAA9T7D8	DPP3	A0AAA9T7D8	ENSBTAG00000022242	0.005753527	-0.03058832	-0.036341847
A0A3Q1MY58	PALM2	A0A3Q1MY58	ENSBTAG00000014471	0.192106122	0.218281005	0.026174883
Q3ZBV8	TARS1	bta:510075; Q3ZBV8	ENSBTAG00000014261	0.031562808	0.131536592	0.099973783
A0A3Q1N365	GOLGA4	A0A3Q1N365	ENSBTAG00000016563	0.014355293	-0.027674958	-0.042030251
A0A3Q1MQ84	TRIP11	A0A3Q1MQ84	ENSBTAG00000026953	0.095185524	0.039348667	-0.055836856
F1N6D5	EFTUD2	F1N6D5	ENSBTAG00000013526	-0.084120621	0.007027267	0.091147888
E1BFV0	KPNB1	bta:507804; E1BFV0	ENSBTAG00000006586	0.061460002	0.107220957	0.045760955
A0AAA9U0Z2	MSH2	A0AAA9U0Z2	ENSBTAG00000002742	-0.255559285	-0.180839932	0.074719353
A0A3Q1N1M4	ACACA	A0A3Q1N1M4	ENSBTAG00000017567	0.006751042	-0.069307036	-0.076058078
A0A3Q1MR43	DDX46	A0A3Q1MR43	ENSBTAG00000021520	-0.139747115	-0.132501017	0.007246098
A0A3Q1LIW3	AP2A2	A0A3Q1LIW3	ENSBTAG00000009915	-0.041730565	0.069881426	0.111611991
E1BGC5	YLPM1	bta:540849; E1BGC5	ENSBTAG00000008287	0.017107256	-0.015861874	-0.032969131
A0A3Q1LWK1	EPB41L2	A0A3Q1LWK1	ENSBTAG00000005960	0.177304532	0.189520482	0.01221595
A0AAA9SAT9	ITGA6	A0AAA9SAT9; bta:535043	ENSBTAG00000017266	-0.018223759	-0.140978625	-0.122754866
A0AAA9SHL0	DOCK7	A0AAA9SHL0	ENSBTAG00000019877	-0.071892918	-0.048021515	0.023871404
A0A3Q1M2R2	MIA3	A0A3Q1M2R2	ENSBTAG00000018824	0.197124966	0.016472434	-0.180652531
Q2KIZ8	MCM6	bta:517812; Q2KIZ8		-0.030786125	-0.011119092	0.019667033
A0A3Q1M1Z2	LOC100141266	A0A3Q1M1Z2	ENSBTAG00000056851	-0.104998956	-0.062078089	0.042920867
A0AAA9TNY2	LMNB1	A0AAA9TNY2	ENSBTAG00000002882	0.037152998	-0.036525876	-0.073678874
F1MC31	SF3B2	F1MC31	ENSBTAG00000002778	0.189162298	0.081159648	-0.10800265
F1ME38	UBA6	F1ME38	ENSBTAG00000006837	-0.12882998	-0.05166212	0.077167861
P35605	COPB2	A4IFE1; bta:281706; P35605	ENSBTAG00000014843	0.072074677	0.116779449	0.044704772
F1MCZ3	SCIN	F1MCZ3	ENSBTAG00000009786	0.038303888	0.063286245	0.024982357
E1BGE5	IPO7	bta:513707; E1BGE5	ENSBTAG00000027722	-0.115365903	-0.025606656	0.089759247
E1BGH0	ABCF1	E1BGH0	ENSBTAG00000006927	-0.041253685	-0.051199534	-0.00994585
F1MMA0	ILF3	bta:614936; F1MMA0	ENSBTAG00000040076	-0.129813998	-0.077857321	0.051956677
A0A3Q1N716	KIAA1217	A0A3Q1N716	ENSBTAG00000018395	0.005992511	0.138731291	0.13273878
P11024	NNT	A4FUB4; bta:280878; P11024	ENSBTAG00000011885	0.096040567	0.11450463	0.018464063
E1BMC4	GBF1	E1BMC4	ENSBTAG00000006014	0.070659774	0.052974465	-0.017685309
A1A4J1	PFKL	A1A4J1; bta:508683	ENSBTAG00000010658	-0.001874848	0.024153862	0.026028709
A0AAA9TW48	TRIP12	A0AAA9TW48	ENSBTAG00000021653	-0.126941831	-0.125530882	0.001410949
A0AAA9SJ11	ACADVL	A0AAA9SJ11	ENSBTAG00000003072	-0.068400775	-0.034522765	0.03387801
A0AAA9T4D7	CAPN1	A0AAA9T4D7	ENSBTAG00000010230	-0.113582176	-0.084337356	0.02924482
E1BNQ9	HNRNPUL2	bta:616476; E1BNQ9	ENSBTAG00000002510	0.048396825	0.055814296	0.007417472
P48644	ALDH1A1	A7E3P6; bta:281615; P48644; Q3MHL5	ENSBTAG00000008103	-0.12763328	-0.109711372	0.017921908
P61286	PABPC1	bta:282296; P61286; Q3T0J7	ENSBTAG00000046358	-0.074998148	-0.057991723	0.017006425
G3X6W2	RAD50	bta:788127; G3X6W2	ENSBTAG00000011252	-0.027324587	-0.055176707	-0.027852121
Q2KJ47	EHD2	bta:538348; Q2KJ47	ENSBTAG00000021191	0.119247	-0.061827314	-0.181074315
Q3SZ57	AFP	bta:506011; Q3SZ57	ENSBTAG00000017131	-0.34681927	0.344230489	0.691049759
Q27971	CAPN2	A4IFD3; bta:281662; Q27971	ENSBTAG00000012778	0.034512292	0.021105633	-0.013406659
A0A3Q1MFI5	DPYSL2	A0A3Q1MFI5	ENSBTAG00000018373	0.142267518	0.208464261	0.066196743
F1N3P2	USP5	F1N3P2	ENSBTAG00000019780	-0.114089343	-0.188089925	-0.074000581
A0A3Q1NAH5	SPAG9	A0A3Q1NAH5	ENSBTAG00000012293	-0.043206931	-0.029177371	0.01402956
A4FUC8	CARS1	A0A3Q1MBW4; A4FUC8; bta:515715; F1N5G7	ENSBTAG00000010777	-0.056694723	-0.11872392	-0.062029197
E1BPX1	VPS13C	E1BPX1	ENSBTAG00000038920	-0.04226948	-0.055195654	-0.012926174
A0AAF6DLX9	HSD17B4	A0AAF6DLX9	ENSBTAG00000006978	0.006068127	-0.004567904	-0.010636031
E1BJH1	EDC4	bta:513171; E1BJH1	ENSBTAG00000006417	0.027221469	0.059809395	0.032587927
A0AAA9SJ67	LARP1	A0AAA9SJ67	ENSBTAG00000008443	-0.008379666	-0.094967351	-0.086587685
F1MF76	LOC515570	bta:515570; F1MF76	ENSBTAG00000017759	-0.004220466	-0.035553673	-0.031333207
A0AAA9T6M2	ANXA2	A0AAA9T6M2	ENSBTAG00000009615	0.219423912	0.211975805	-0.007448107
E1BAF6	PRRC2A	bta:538553; E1BAF6	ENSBTAG00000019682	-0.024416527	0.073573264	0.097989791
A0AAA9SZP1	MSH6	A0AAA9SZP1	ENSBTAG00000001424	-0.166281173	-0.178812805	-0.012531632
A0A3Q1M1V8	TPM1	A0A3Q1M1V8	ENSBTAG00000005373	0.155481265	0.182034684	0.026553419
E1BQ37	SFPQ	E1BQ37	ENSBTAG00000016328	0.038703529	0.058370563	0.019667033
E1BP91	NPEPPS	bta:534122; E1BP91	ENSBTAG00000011435	0.183878768	0.275486147	0.091607379
P81948	TUBA4A	bta:777775; P81948; Q17QT1; Q5E986		-0.027238601	0.065068574	0.092307175
A0A3Q1MFI2	FAM120A	A0A3Q1MFI2	ENSBTAG00000008696	-0.070389328	-0.028912692	0.041476636
A0A3Q1M384	SMARCA5	A0A3Q1M384	ENSBTAG00000003399	-0.230005605	-0.152003093	0.078002512
A0A3Q1MME4	HNRNPA2B1	A0A3Q1MME4	ENSBTAG00000005726	0.116829955	0.122228259	0.005398304
A0AAA9S896	LONP1	A0AAA9S896	ENSBTAG00000001795	0.043336408	0.043336408	0
F1N693	CUX1	F1N693	ENSBTAG00000010659	-0.082952789	-0.11872392	-0.035771131
F1N0E5	CCT4	F1N0E5	ENSBTAG00000004531	0.088286532	0.094859186	0.006572654
A0A3Q1LIF3	MCM5	A0A3Q1LIF3	ENSBTAG00000015595	0.018262206	0.059995965	0.041733758
A0A3Q1N4S4	NUP205	A0A3Q1N4S4	ENSBTAG00000011127	-0.121306296	-0.111845967	0.009460329
F1MTG3	WASHC2A	bta:513740; F1MTG3	ENSBTAG00000018915	-0.030469877	0.064851144	0.095321021
F1N2K8	PPL	F1N2K8	ENSBTAG00000002178	0.080556354	0.011382268	-0.069174086
G5E531	TCP1	G5E531	ENSBTAG00000002829	-0.133992461	0.018164923	0.152157384
E1B6X6	BAZ1B	bta:508442; E1B6X6	ENSBTAG00000005197	-0.180572246	-0.067114196	0.11345805
F1N4K1	PFAS	F1N4K1	ENSBTAG00000031509	-0.053874241	0.017520099	0.07139434
Q3SZN2	SEC23B	bta:535071; Q3SZN2	ENSBTAG00000018295	-0.237499314	-0.200846891	0.036652423
A7MB16	EIF3B	A7MB16; bta:789999	ENSBTAG00000007474	-0.105205596	-0.081481402	0.023724194
A0A3Q1LUD9	DIAPH1	A0A3Q1LUD9	ENSBTAG00000030499	0.073103713	0.096162728	0.023059016
Q0VCX4	CTNNB1	A7E3R2; bta:539003; Q0VCX4		0.009944761	-0.117260304	-0.127205065
Q2T9S0	TUBB3	bta:768070; Q2T9S0		0.110469593	0.159780444	0.049310851
P62261	YWHAE	bta:282125; P29360; P42655; P62261; Q3ZC40; Q63631	ENSBTAG00000005664	0.093392036	0.108852141	0.015460105
A4FV12	UBA2	A4FV12; bta:507005	ENSBTAG00000002573	0.019587805	0.072482754	0.052894948
F1N3R4	LETM1	F1N3R4	ENSBTAG00000020108	0.059647463	0.076246247	0.016598784
A0AAF7AFD7	EHD1	A0AAF7AFD7	ENSBTAG00000050712	0.07075666	0.069286771	-0.001469888
A0A3Q1M5W5	FAT1	A0A3Q1M5W5	ENSBTAG00000020657	-0.109929775	-0.119348927	-0.009419152
P21856	GDI1	A4FUY4; bta:281188; P21856	ENSBTAG00000012125	-0.080005643	0.039801008	0.119806651
A0AAA9TK27	MDN1	A0AAA9TK27	ENSBTAG00000001644	-0.121867775	-0.111309583	0.010558192
A0AAA9S9R4	PSMC3	A0AAA9S9R4	ENSBTAG00000021744	0.025261843	-0.005674324	-0.030936167
A0A3Q1MR81	MYO1B	A0A3Q1MR81	ENSBTAG00000011256	-0.025887562	0.031236837	0.057124399
F1MHM5	YARS1	F1MHM5	ENSBTAG00000018065	0.049505185	0.181432102	0.131926917
A0AAA9T3T4	UNC45A	A0AAA9T3T4	ENSBTAG00000018639	0.024982357	-0.008847041	-0.033829397

Accession	Gene_Symbol	Gene_ID	Ensembl_Gene ID	log2(ratio(CM12H/CM0H))	log2(ratio(CM12R/CM0H))	log2(ratio(CM12H/CM12R))
A4FUZ3	PSMC1	A4FUZ3; F1N550	ENSBTAG00000005426	0.047305715	-0.039892727	-0.087198442
A0A452DIS2	ITGB1	A0A452DIS2	ENSBTAG00000015910	-0.008964845	-0.167692467	-0.158727623
P51176	TGM2	bta:281528; P51176; Q3ZBH7		-0.104076184	-0.03742485	0.066651334
P68103	EEF1A1	A5PK01; bta:282220; P04719; P04720; P68103; Q2KJJ3	ENSBTAG00000014534	-0.016119665	-0.014646776	0.001472889
A0AAA9T5M5	BOD1L1	A0AAA9T5M5	ENSBTAG00000004316	-0.133743706	0.013383167	0.147126873
Q3MHH4	QARS1	bta:514586; Q3MHH4	ENSBTAG00000018928	-0.116917752	-0.057274805	0.059642946
A0A3S5ZPM3	PGD	A0A3S5ZPM3	ENSBTAG00000013527	0.027192963	0.08297042	0.055777457
F1MM55	NUP133	F1MM55	ENSBTAG00000004965	-0.105332202	-0.095032449	0.010299753
O02751	CFDP2	bta:286876; O02751; O02752; O18732; Q7JFY0; Q8I032		0.05246742	0.0115071	-0.04096032
A0AAF6YIC2	HNRNPA1	A0AAF6YIC2	ENSBTAG00000001553	-0.018253319	-0.015430041	0.002823279
Q3SZ62	PGAM1	bta:404148; Q3SZ62	ENSBTAG00000012697	0.135456785	0.093900139	-0.041556646
A5PJZ5	NUP93	A5PJZ5; bta:510004	ENSBTAG00000006611	0.001393235	-0.005586435	-0.00697967
A0JN67	GLS	A0JN67; bta:525335		0.098563834	0.102270976	0.003707141
A0A3Q1LG90	PALLD	A0A3Q1LG90	ENSBTAG00000001081	-0.012809807	-0.007992791	0.004817016
A0AAA9RXX2	RBBP6	A0AAA9RXX2; bta:282034	ENSBTAG00000009441	0.225945087	0.17884808	-0.047097007
A0AAA9SE56	PTK7	A0AAA9SE56	ENSBTAG00000012761	-0.045562577	-0.046848975	-0.001286398
A6H7I5	DNM2	A6H7I5; bta:511691		0.057647659	0.13372436	0.076076701
Q2KJC1	CDC5L	bta:767817; Q2KJC1	ENSBTAG00000020012	0.090998121	0.095126006	0.004127886
E1BNE5	RAB11FIP5	E1BNE5	ENSBTAG00000006162	0.097202159	0.102641167	0.005439008
A0A3Q1MA79	LAMA5	A0A3Q1MA79	ENSBTAG00000003061	-0.12817708	-0.371448231	-0.243271151
F1MJ56	MYO1D	F1MJ56	ENSBTAG00000015527	-0.560300446	-0.297266041	0.263034406
A0A3Q1LLF1	TNC	A0A3Q1LLF1	ENSBTAG00000000575	-0.161423234	-0.458795426	-0.297372192
P10096	GAPDH	bta:281181; P10096; P79130; Q3ZCB1	ENSBTAG00000014731	0.04336104	0.077404504	0.034043464
A0A3S5ZP34	POR	A0A3S5ZP34	ENSBTAG00000017082	0.13439594	0.147300275	0.012904335
F1MQ01	ATP2B4	F1MQ01	ENSBTAG00000014059	0.0648681	0.09262714	0.027759039
A0AAA9S9R1				-0.850184466	-0.395736943	0.454447522
A0A3Q1ME65	STAT1	A0A3Q1ME65	ENSBTAG00000007867	-0.122029633	0.002656898	0.124686531
A0A3Q1MDH5	ACAA2	A0A3Q1MDH5	ENSBTAG00000002863	0.171556456	0.123815867	-0.047740589
A0A3Q1M6W0	NCOR2	A0A3Q1M6W0	ENSBTAG00000024603	-0.237375451	-0.301190548	-0.063815098
F1N7H0	DNAJC7	F1N7H0	ENSBTAG00000002654	0.246897837	0.139007114	-0.107890723
A0A3Q1LQ12	EPB41L2	A0A3Q1LQ12	ENSBTAG00000005960	0.531658351	-0.057310843	-0.588969195
E1BJ31	EPHA2	bta:512798; E1BJ31	ENSBTAG00000000815	0.019606576	0.167546882	0.147940306
Q3MHK9	FSCN1	bta:507342; F1N089; Q3MHK9	ENSBTAG00000003191	-0.058893689	0.138175952	0.197069641
Q0VCU1	ACO1	bta:512995; Q0VCU1		0.036641844	0.144095347	0.107453503
A0AAA9TEU1				0.040098069	-0.080514606	-0.120612674
P62739	ACTA2	bta:515610; P03996; P04108; P62739; Q3ZCA0; Q862W5	ENSBTAG00000014614	-0.016044967	-0.122029633	-0.105984666
A7Z066	CANX	A7Z066; bta:407129	ENSBTAG00000048107	0.262504101	0.261177484	-0.001326616
E1BIA7	CLUH	E1BIA7	ENSBTAG00000011786	-0.202178149	-0.110547673	0.091630475
A0A3Q1M7S1	FBN1	A0A3Q1M7S1	ENSBTAG00000002278	-0.311944006	-0.52286638	-0.210922373
E1BCK1	RIF1	E1BCK1	ENSBTAG00000021020	-0.263994922	-0.241117217	0.022877705
F1MCT8	SYNCRIP	F1MCT8	ENSBTAG00000006672	0.002798633	-0.065914117	-0.06871275
A0AAA9T461				0.065140605	0.158342581	0.093201977
A0A452DJG1	ATP6V1A	A0A452DJG1; bta:282147	ENSBTAG00000002703	0.092101584	0.022392612	-0.069708972
F6Q9Q9	P4HB	F6Q9Q9	ENSBTAG00000006045	0.199795531	0.136680146	-0.063115385
Q28006				-0.081864647	-0.078284757	0.00357989
F1MF50	ROCK1	F1MF50	ENSBTAG00000008403	-0.033105974	0.016919486	0.05002546
F1MUF1	XPO5	F1MUF1	ENSBTAG00000018970	-0.091651574	-0.080722013	0.01092956
A0JN52	SF3B3	A0JN52; bta:504962	ENSBTAG00000010627	-0.072526259	-0.079560378	-0.007034119
A0AAA9TFB6				-0.052974465	-0.029034613	0.023939852
Q1RMU3	P4HA1	bta:518288; Q1RMU3	ENSBTAG00000032996	0.269560921	0.093024816	-0.176536105
A0AAA9SJ37	SHMT2	A0AAA9SJ37	ENSBTAG00000031500	-0.150257383	-0.260376396	-0.110119012
A0A3Q1MK49	AKAP9	A0A3Q1MK49	ENSBTAG00000007442	0.043111625	0.014514157	-0.028597468
E1BJ59	ATXN2L	E1BJ59	ENSBTAG00000032087	0.072366977	0.131825212	0.059458234
A0AAA9SQC6	ARHGEF2	A0AAA9SQC6	ENSBTAG00000000113	-0.09011242	-0.088704225	0.001408194
A0A140T871	GLUD1	A0A140T871; bta:281785	ENSBTAG00000007540	-0.010992015	-0.037438685	-0.02644667
P62194	PSMC5	A6H7C5; bta:282015; O35051; P47210; P52915; P52916; P62194	ENSBTAG00000021061	0.042560462	-0.054281661	-0.096842123
F1N533	DDX18	bta:519254; F1N533	ENSBTAG00000015077	0.046383865	0.007124434	-0.03925943
A0A3Q1MJ74	PDLIM5	A0A3Q1MJ74	ENSBTAG00000012818	-0.1303372	-0.175985597	-0.045648397
A0A3Q1N9S5	HNRNPK	A0A3Q1N9S5	ENSBTAG00000021131	0.024499184	-0.068880161	-0.093379345
A0AAA9S3H4				-0.244143168	-0.069149722	0.174993446
P15690	NDUFS1	bta:288380; P15690; Q0VCP7	ENSBTAG00000021976	-0.148352679	-0.125103733	0.023248946
A2VDN6	SF3A1	A2VDN6; bta:504381	ENSBTAG00000003390	-0.023708427	-0.052108853	-0.028400426
A0A3Q1MH83	GAPVD1	A0A3Q1MH83	ENSBTAG00000011544	0.03522523	-0.020952371	-0.056177601
A7E307	DDX17	A7E307; bta:514323		0.262732302	0.354897226	0.092164923
F1MSF1	RRM1	F1MSF1	ENSBTAG00000013111	-0.193001079	-0.261540159	-0.06853908
Q08E34	TOMM70	bta:507707; Q08E34	ENSBTAG00000014302	-0.009790503	-0.023893206	-0.014102703
Q8MJG1	PSIP1	A7E383; bta:282011; Q0VCL7; Q8MJG1	ENSBTAG00000007872	0.119494224	0.166845609	0.047351385
A0A3Q1MJD6	TPX2	A0A3Q1MJD6	ENSBTAG00000018775	0.221186659	0.152003093	-0.069183565
E1BCW3	PFKP	E1BCW3	ENSBTAG00000002683	0.020177882	0.027316417	0.007138535
A0A3Q1M5A0	FXR1	A0A3Q1M5A0	ENSBTAG00000016457	0.158512393	0.083532623	-0.07497977
Q0V888	COPG	Q0V888		-0.258624738	-0.274859841	-0.016235103
A4IFM8	ACTA1	A4IFM8		0.066733989	0.005564887	-0.061169102
A0A3Q1LNN6	KIF4A	A0A3Q1LNN6	ENSBTAG00000012861	-0.458547163	-0.249181422	0.209365741
A0AAA9SNZ2	USP14	A0AAA9SNZ2	ENSBTAG00000019214	-0.056344592	0.069962558	0.126307151
A5D7C6	PREP	A5D7C6	ENSBTAG00000016281	-0.081805718	-0.038172502	0.043633216
A0AAA9SAU9	UBAP2L	A0AAA9SAU9	ENSBTAG00000004271	-0.048999401	-0.077568553	-0.028569152
F6RN21	PABPC4	F6RN21	ENSBTAG00000017125	-0.102051271	-0.109624491	-0.00757322
A0A3Q1M8Y4	SKIC3	A0A3Q1M8Y4	ENSBTAG00000009131	-0.189779744	-0.066021969	0.123757775
Q2HJG5	VPS35	bta:521864; Q2HJG5	ENSBTAG00000002493	0.123382416	0.161311871	0.037929456
P12378	UGDH	A6QR10; bta:281564; O77806; P12378	ENSBTAG00000014521	0.134878054	0.125651102	-0.009226953
A0A3Q1MEH9	TJP2	A0A3Q1MEH9	ENSBTAG00000011770	0.021752799	-0.03523718	-0.056989979
A7YWL9	GSPT1	A7YWL9		-0.076647382	0.034047481	0.110694863
F1MVW5	SEC23A	F1MVW5	ENSBTAG00000003708	0.046148461	0.052812191	0.00666373
P10881	SSB	bta:338071; P10881; Q3ZBL2	ENSBTAG00000008716	-0.054750204	-0.073931617	-0.019181413

Accession	Gene_Symbol	Gene_ID	Ensembl_Gene ID	log2(ratio(CM12H/CM0H))	log2(ratio(CM12R/CM0H))	log2(ratio(CM12H/CM12R))
A0AAF6Z672	TUFM	A0AAF6Z672	ENSBTAG00000019216	0.042242449	0.008841877	-0.033400572
E1BN24	ZNFX1	E1BN24	ENSBTAG00000020166	0.022675438	-0.06874759	-0.091423028
A7E3Q2	HSPA2	A7E3Q2	ENSBTAG00000007807	0.109260183	0.047016627	-0.062243557
Q3SYW6	EIF3C	bta:534882; Q3SYW6	ENSBTAG00000006543	-0.06009187	-0.031830534	0.028261335
A0AAA9U1R6	SWAP70	A0AAA9U1R6	ENSBTAG00000001987	0.088390586	0.104785025	0.016394438
A7MBB7	NFKB2	A7MBB7; bta:526392; F6Q9X9		-0.159031294	0.026993605	0.186024899
Q5E9T9	RTCB	bta:525106; Q5E9T9	ENSBTAG00000011070	0.072482754	0.038913221	-0.033569533
Q9GMB8	SARS1	bta:281476; Q9GMB8	ENSBTAG00000012962	0.020386986	0.032482311	0.012095325
E1BDU0	NUP107	bta:504823; E1BDU0	ENSBTAG00000006911	-0.002851177	0.001423478	0.004274655
E1B801	ARHGAP35	bta:540310; E1B801	ENSBTAG00000015543	-0.056244111	0.03493379	0.091177901
A6QL77	P4HA1	A6QL77; E1BLJ7	ENSBTAG00000032996	0.408084739	0.346381139	-0.0617036
A0AAF6DLQ5	MTREX	A0AAF6DLQ5	ENSBTAG00000010518	-0.203091865	-0.224215157	-0.021123292
A0A3Q1MB56	SEPTIN9	A0A3Q1MB56; bta:100140583	ENSBTAG00000002633	0.056116604	0.132450296	0.076333692
F1MFA3	PITRM1	F1MFA3	ENSBTAG00000002685	-0.260205843	-0.278821521	-0.018615678
A7MB83	PLOD2	A7MB83; bta:533642	ENSBTAG00000007331	-0.026160192	0.097719821	0.123880012
E1BJG3	GEMIN5	E1BJG3	ENSBTAG00000012095	-0.127908367	-0.083514248	0.044394119
A0A3Q1M5H7	MRC2	A0A3Q1M5H7	ENSBTAG00000015739	-0.133445307	-0.117325642	0.016119665
A0A3Q1MWC7	ME1	A0A3Q1MWC7	ENSBTAG00000005681	0.063823577	0.062135227	-0.00168835
A0A3Q1M326	SYNCRIP	A0A3Q1M326	ENSBTAG00000006672	0.028768551	0.02019806	-0.008570491
A0A3Q1NI32	THRAP3	A0A3Q1NI32; bta:505228	ENSBTAG00000024095	-0.125403923	0.02629445	0.151698374
A0A3Q1NNE9	COBLL1	A0A3Q1NNE9	ENSBTAG00000005501	-0.064385921	-0.077377942	-0.01299202
A7Z055	PLAA	A7Z055; bta:506073; F1MF89	ENSBTAG00000013011	-0.039991975	0.066700401	0.106692377
A0A3Q1MH62	PAPSS1	A0A3Q1MH62	ENSBTAG00000012961	-0.018671277	-0.039052777	-0.0203815
A0A3Q1M2H6	CUL1	A0A3Q1M2H6; bta:407228	ENSBTAG00000008736	-0.075868954	0.037929456	0.11379841
A0A3Q1M7L5	HSPA4L	A0A3Q1M7L5	ENSBTAG00000015692	0.329555916	0.337831436	0.00827552
Q3SYZ5	DDX5	Q3SYZ5		-0.451630236	-0.318858233	0.132772003
A4IFB8	IPO4	A4IFB8; bta:510372; F1MGQ4		-0.204841961	-0.271166721	-0.066324759
A0AAF6YY96	ACAT1	A0AAF6YY96	ENSBTAG00000012885	-0.018916091	0.046932612	0.065848703
A6H7B6	DDX24	A6H7B6; bta:529613	ENSBTAG00000003423	0.298371131	0.112338876	-0.186032255
A0A3Q1LYQ6	NEXN	A0A3Q1LYQ6	ENSBTAG00000008921	0.045876522	-0.001165814	-0.047042336
F1MTP5	WDR1	F1MTP5	ENSBTAG00000013607	-0.101283336	-0.01227833	0.089005006
Q2KJG3	NARS	bta:616033; Q2KJG3		0.027928744	0.078002512	0.050073768
A0AAA9RW37	NIBAN2	A0AAA9RW37	ENSBTAG00000012847	-0.064630059	-0.124781625	-0.060151566
A0A3Q1ME79	XPO5	A0A3Q1ME79	ENSBTAG00000018970	-0.080098539	-0.131483925	-0.051385386
A0AAA9SZ98	NSUN2	A0AAA9SZ98; bta:516044	ENSBTAG00000015466	-0.239071162	-0.281631625	-0.042560462
Q0VCR7	MTHFD1L	A6QNK9; bta:534296; Q0VCR7		-0.168681835	-0.204061122	-0.035379287
Q3T122	EIF3D	bta:515226; Q3T122	ENSBTAG00000001988	-0.023549074	-0.008267616	0.015281458
A0A3Q1M8V2	UBR5	A0A3Q1M8V2	ENSBTAG00000021209	-0.290525213	-0.208063053	0.08246216
A1L5B6	ANXA5	A1L5B6; bta:281626		0.20321432	0.208470045	0.005255725
F1MBW3	ACSL4	F1MBW3	ENSBTAG00000018986	-0.262795371	-0.00598878	0.256806591
A4IFB1	SRRT	A4IFB1; bta:527938		0.012723508	0.016939862	0.004216355
A0A3Q1LPZ9	CLASP1	A0A3Q1LPZ9	ENSBTAG00000019781	0.038023446	0.015612199	-0.022411247
F1MJB0	FILIP1L	F1MJB0	ENSBTAG00000013664	0.264148886	0.086544387	-0.1776045
Q2HJI1	SDHA	Q2HJI1		-0.143708053	-0.040888831	0.102819222
A0AAF6DLK2	IQGAP3	A0AAF6DLK2	ENSBTAG00000006882	-0.382544025	-0.102116702	0.280427323
A0A3Q1MRG1	CUL3	A0A3Q1MRG1	ENSBTAG00000021769	0.029982866	0.052415894	0.022433028
A0AAA9SL11	ITGB3	A0AAA9SL11	ENSBTAG00000009987	0.084135335	0.155201349	0.071066014
Q3ZBU7	TUBB4A	bta:540236; Q3ZBU7		0.354943152	0.491853096	0.136909944
Q2TBU9	RUVBL2	bta:511048; Q2TBU9		0.027192963	0.019690632	-0.007502331
E1BDS6	TP53BP1	E1BDS6	ENSBTAG00000021304	-0.073275095	0.085175367	0.158450462
Q2KJH7	ALDH18A1	bta:514759; Q2KJH7	ENSBTAG00000011832	0.004228713	0.09028336	0.086054647
E1B7F5	WDHD1	bta:540681; E1B7F5	ENSBTAG00000019120	-0.151427627	0.043373409	0.194801036
A0A3Q1MDF7	GTF2I	A0A3Q1MDF7	ENSBTAG00000009780	-0.094422395	-0.14483402	-0.050411626
F6QI68	PPP2R1A	F6QI68	ENSBTAG00000019851	0.056075924	0.043327432	-0.012748493
A0A3Q1M4Y9	USP15	A0A3Q1M4Y9	ENSBTAG00000010428	-0.00937253	-0.066923552	-0.057551023
A1L505	UBAP2L	A1L505; bta:100125221		-0.17417966	-0.168899892	0.005279768
A0A3Q1M7L2	ANKHD1	A0A3Q1M7L2	ENSBTAG00000010871	-0.101445264	-0.308001137	-0.206555873
A0AAA9S1Z4	SAFB2	A0AAA9S1Z4	ENSBTAG00000001790	0.027985774	0.025065337	-0.002920436
Q5E9D0	EIF2S2	bta:520094; Q0IIJ6; Q5E9D0		0.121745272	0.090839231	-0.030906042
A0AAA9S8Q5				-0.648223514	-0.630693589	0.017529925
Q32LP6	STAT3	bta:508541; Q32LP6		0.029413762	-0.013434402	-0.042848163
F1MI56	ANKRD17	F1MI56	ENSBTAG00000004912	0.045453818	-0.107453503	-0.152907321
A0A3Q1LQF0	PSMC6	A0A3Q1LQF0	ENSBTAG00000015732	-0.055979672	-0.0660034	-0.010023728
A0AAF6DMH3	FUBP1	A0AAF6DMH3	ENSBTAG00000008926	0.198287225	0.08061846	-0.117668764
A0AAA9STY1	GFPT1	A0AAA9STY1	ENSBTAG00000017626	-0.02774499	-0.010726407	0.017018584
E1BNG3	ascc3	bta:538416; E1BNG3	ENSBTAG00000020482	-0.059926729	0.010397847	0.070324576
A0AAA9SFG0	MYO1E	A0AAA9SFG0; bta:523485	ENSBTAG00000021538	-0.143181318	-0.15336509	-0.010183772
A0AAA9SUI7	PALLD	A0AAA9SUI7	ENSBTAG00000001081	-0.028110707	0.011417657	0.039528364
A0A3Q1NCG2	COBLL1	A0A3Q1NCG2	ENSBTAG00000005501	-0.08861931	-0.033015057	0.055604253
A0AAA9RWB8	TRIO	A0AAA9RWB8	ENSBTAG00000005514	-0.135557129	-0.164890623	-0.029333494
A5PKI0	APPL1	A5PKI0; bta:517850	ENSBTAG00000006824	0.05270705	0.193148256	0.140441206
A0AAA9U0H2	IDE	A0AAA9U0H2	ENSBTAG00000019759	-0.134728969	-0.109278341	0.025450628
A0AAA9ST00	PTPN11	A0AAA9ST00	ENSBTAG00000002048	-0.005799787	-0.024811755	-0.019011969
P00727	LAP3	bta:781648; P00727; Q2HJH5; Q2PC24	ENSBTAG00000005989	0.096542028	0.170407266	0.073865238
G3MXU7	PHLDB1	G3MXU7	ENSBTAG00000016197	0.014241923	-0.05246742	-0.066709342
E1BMZ5	EXOSC10	E1BMZ5	ENSBTAG00000002590	-0.041127885	-0.150115034	-0.108987149
F1MV07	SEC24D	F1MV07	ENSBTAG00000003619	-0.20624838	-0.072892521	0.133355859
A0AAA9TZ00	HNRNPA3	A0AAA9TZ00; bta:782669	ENSBTAG00000021580	0.084423061	0.089539006	0.005115945
Q3MHE8	SRPRA	bta:281504; Q3MHE8	ENSBTAG00000014105	-0.019821441	0.001405451	0.021226892
A0AAA9SB13	LDHA	A0AAA9SB13	ENSBTAG00000008683	0.108742693	0.15140059	0.042657897
Q17R04	CCAR1	bta:767587; F1MMZ9; Q17R04	ENSBTAG00000033457	0.086827353	0.099844238	0.013016886
A0AAA9SGU6	GOT2	A0AAA9SGU6	ENSBTAG00000007172	0.069523104	0.052088511	-0.017434593
Q1JPJ8	THOP1	A6QQT3; bta:510889; Q1JPJ8		-0.090288885	-0.053199567	0.037089319
A0AAA9S386	HADHB	A0AAA9S386	ENSBTAG00000010083	-0.149480899	-0.081794091	0.067686808
O77588	PLOD1	bta:281409; O77588; Q1JPK0		0.237482967	0.333598088	0.096115121
F1N6K7	SEC16A	bta:519689; F1N6K7	ENSBTAG00000023933	0.004391769	0.004391769	0
A5PJZ1	SLC25A24	A5PJZ1; bta:534742	ENSBTAG00000003752	0.009870244	0.081820086	0.071949842
Q08E58	TTLL12	bta:511319; F1N5K2; Q08E58	ENSBTAG00000001708	0.106029436	0.121781101	0.015751666
F1MHN8	ALCAM	F1MHN8	ENSBTAG00000000088	0.041594089	-0.034701091	-0.07629518
F1N405	RTN4	F1N405	ENSBTAG00000011104	0.045853894	0.039615203	-0.006238691

Accession	Gene_Symbol	Gene_ID	Ensembl_Gene ID	log2(ratio(CM12H/CM0H))	log2(ratio(CM12R/CM0H))	log2(ratio(CM12H/CM12R))
E1BF29	KIF11	bta:508467; E1BF29	ENSBTAG00000009383	0.108566357	0.240451998	0.13188564
A0A3Q1M070	RRP12	A0A3Q1M070	ENSBTAG00000002191	-0.289633946	-0.398705645	-0.109071699
A0A452DHZ5	NUCB1	A0A452DHZ5	ENSBTAG00000002378	0.066962213	0.170110736	0.103148523
E1BIU0	SEC24C	E1BIU0	ENSBTAG00000002791	0.035046947	0.13804094	0.102993993
A0A3Q1ND98	ATP2B1	A0A3Q1ND98	ENSBTAG00000009552	0.16774914	0.237308704	0.069559564
Q6Q137	SEPTIN7	bta:408000; Q6Q137	ENSBTAG00000001182	0.037637657	0.090823592	0.053185935
A0A3Q1MP70	GPI	A0A3Q1MP70	ENSBTAG00000006396	0.080711799	0.056143078	-0.024568721
Q3SWY3	IMPDH2	bta:511969; Q3SWY3		-0.138024917	-0.300262201	-0.162237284
A0AAF7AMW6	ARCN1	A0AAF7AMW6	ENSBTAG00000006690	0.062464087	0.090713564	0.028249477
Q3T030	PSMC4	bta:510029; Q3T030	ENSBTAG00000009414	0.012773576	-0.034626343	-0.047399919
A6QP73	DDX31	A6QP73; bta:507640		-0.05708737	0.05221752	0.109304891
A5D7J6	CALR	A5D7J6	ENSBTAG00000015114	0.335633243	0.184616704	-0.151016539
E1BB38	SRP72	bta:509430; E1BB38	ENSBTAG00000010593	-0.026887689	0.046898615	0.073786304
Q5E9F9	PSMC2	bta:530186; Q3T0H8; Q5E9F9	ENSBTAG00000046712	0.039528364	0.014241923	-0.025286442
A0AAA9SIE1	INF2	A0AAA9SIE1	ENSBTAG00000007187	-0.288641062	-0.211855682	0.07678538
A0A3Q1MCX8	SHTN1	A0A3Q1MCX8; bta:532603	ENSBTAG00000007578	-0.022739972	0.016203225	0.038943197
Q2TA49	VASP	bta:514902; Q2TA49	ENSBTAG00000019604	-0.056094562	0.00152747	0.057622032
Q3MHF7	MTAP	bta:782907; F1N1J3; Q3MHF7	ENSBTAG00000025929	0.043426378	0.11956434	0.076137962
E1BCR2	DIDO1	E1BCR2	ENSBTAG00000006002	0.069082134	0.10443741	0.035355276
E1BGF3	HCFC1	E1BGF3	ENSBTAG00000011904	-0.058587026	0.024811755	0.083398782
Q9TS87	TAGLN	bta:513463; Q2KJH8; Q9TS87	ENSBTAG00000007196	-0.015673878	-0.185777974	-0.170104096
A0A3Q1LUN1	MPRIP	A0A3Q1LUN1	ENSBTAG00000010534	0.046259525	-0.009758966	-0.056018492
A0A3Q1M334	STK10	A0A3Q1M334; bta:526376	ENSBTAG00000017457	0.050676887	0.047828525	-0.002848362
F1MPN0	FNDC3B	bta:615534; F1MPN0	ENSBTAG00000019376	-0.278069734	-0.120471603	0.157598131
A0AAA9RXM7	HLTF	A0AAA9RXM7	ENSBTAG00000019704	-0.261274562	-0.121990524	0.139284037
F1MMK8	KARS1	F1MMK8	ENSBTAG00000015677	0.089925679	0.050006669	-0.03991901
F1MKJ5	WASHC5	bta:508040; F1MKJ5	ENSBTAG00000010275	-0.05945385	0	0.05945385
A0A452DJ96	PGM1	A0A452DJ96	ENSBTAG00000019011	-0.005864622	-0.019147842	-0.01328322
E1BIU7	PTPN23	E1BIU7	ENSBTAG00000002774	-0.165465353	-0.001489618	0.163975735
Q3T102	EIF3E	bta:534165; Q1JP76; Q3T102		0.016630675	-0.072900547	-0.089531222
Q3MHL7	CCT6A	A5D9B9; bta:521540; Q3MHL7	ENSBTAG00000008184	0.071308535	0.063410549	-0.007897985
F1MMJ2	NCAM1	F1MMJ2	ENSBTAG00000005710	-0.049068043	0.006875446	0.055943489
Q3SYU7	TNPO1	bta:767620; Q3SYU7	ENSBTAG00000007979	0.1451515	0.1451515	0
P17248	WARS1	bta:281576; P17248; Q3SZK9	ENSBTAG00000004679	-0.033861958	-0.010797768	0.02306419
F1MET0	PLOD3	bta:514996; F1MET0	ENSBTAG00000000342	0.148098639	0.221180989	0.07308235
Q17R07	ARFGAP3	bta:532778; Q17R07	ENSBTAG00000001057	0.230819781	0.226936346	-0.003883434
Q3SYZ4	DARS1	bta:510162; Q3SYZ4		-0.182591413	-0.174435965	0.008155449
Q08DQ9	DLGAP5	bta:509161; Q08DQ9	ENSBTAG00000002331	0.156941778	0.136409145	-0.020532633
A0AAA9TSD9	AP1G1	A0AAA9TSD9	ENSBTAG00000002769	-0.12388129	0.009743274	0.133624564
A0AAF6YT68	AP3D1	A0AAF6YT68	ENSBTAG00000009034	0.065217659	0.126086727	0.060869067
E1B9R8	SLFN11	bta:521795; E1B9R8	ENSBTAG00000019437	-0.04822828	0.19905378	0.24728206
A0A3Q1NB02	SLTM	A0A3Q1NB02	ENSBTAG00000011319	0.012140563	-0.030114273	-0.042254835
A0A3Q1LRB7	KIF1B	A0A3Q1LRB7	ENSBTAG00000054239	0.299853642	-0.151655414	-0.451509056
A0A3Q1MN33	GCLC	A0A3Q1MN33	ENSBTAG00000015571	0.05504926	0.145936056	0.090886796
A0A3Q1LT42	AAK1	A0A3Q1LT42	ENSBTAG00000009343	0.11144273	0.147711484	0.036268754
A0AAA9T569	LOC515333	A0AAA9T569	ENSBTAG00000032603	0.296942815	0.505843784	0.208900969
A6QQ11	PGM2	A6QQ11; bta:506980		-0.023057261	0.205252218	0.228309479
F1MYS7	EIF4B	F1MYS7	ENSBTAG00000006883	0.121949087	0.107791024	-0.014158063
Q17QG2	NUDC	bta:513277; Q17QG2		0.185310165	0.103747925	-0.08156224
A0AAA9SXS5	FERMT2	A0AAA9SXS5	ENSBTAG00000014501	0.074864787	0.170138434	0.095273647
A0AAA9S1S4	HNRNPR	A0AAA9S1S4	ENSBTAG00000016578	0.064641252	0.13322332	0.068582068
F1N4N6	SMARCC2	F1N4N6	ENSBTAG00000014697	0.290613646	0.239936758	-0.050676887
A0A3Q1MGY6	NCAM1	A0A3Q1MGY6	ENSBTAG00000005710	0.004293738	0.160577829	0.156284091
A4IFJ5	PLA2G4A	A4IFJ5; bta:525072; Q1XD55	ENSBTAG00000013298	-0.018505469	0.164256251	0.18276172
O62768	TXNRD1	bta:282388; O62768	ENSBTAG00000013912	0.089146732	0.090563222	0.00141649
E1BLB6	PRKDC	E1BLB6	ENSBTAG00000017019	-0.271027197	-0.211364275	0.059662921
Q2KJ46	PSMD3	bta:504937; Q2KJ46	ENSBTAG00000021461	0.054207074	-0.041199117	-0.095406191
A3KN39	NCAPG	A3KN39; bta:531234		-0.003707141	-0.242691983	-0.238984842
F1N6Z0	PSMD5	F1N6Z0	ENSBTAG00000016952	-0.006763702	0.001348944	0.008112646
G3N2F2	P4HA2	G3N2F2	ENSBTAG00000001116	-0.060449618	-0.143306551	-0.082856932
A0AAA9SB04	EIF2A	A0AAA9SB04	ENSBTAG00000009106	-0.198206032	-0.161531585	0.036674447
A0AAF7AIK6	ASNS	A0AAF7AIK6	ENSBTAG00000003222	-0.106785413	0.011763449	0.118548862
E1B9N7	DHX29	bta:512470; E1B9N7	ENSBTAG00000010510	-0.136653463	-0.119616608	0.017036854
F1MTY9	HMOX2	A0A8J8Y7Y8; bta:510243; F1MTY9; M5FHP2	ENSBTAG00000018937	0.221289865	0.25400544	0.032715575
E1BNV7	MYO9B	E1BNV7	ENSBTAG00000011125	-0.164507571	-0.079170213	0.085337357
A0AAF6YJJ1	PSMD12	A0AAF6YJJ1	ENSBTAG00000002423	-0.113062664	0.014427071	0.127489735
A0AAA9S255	ADAR	A0AAA9S255	ENSBTAG00000007519	0.029808809	0.171597304	0.141788495
A0A3Q1MNQ1	TUT1	A0A3Q1MNQ1	ENSBTAG00000011846	-0.031800028	-0.077242999	-0.045442971
Q3ZC41	ACAA1	bta:508324; Q3ZC41	ENSBTAG00000018283	0.028362223	-0.086991819	-0.115354042
Q1RMR9	PACSIN2	bta:540107; Q1RMR9	ENSBTAG00000019290	0.132418697	0.163305743	0.030887045
A5D7P3	PAFAH1B1	A5D7P3	ENSBTAG00000016806	0.045166316	0.041868726	-0.00329759
A0AAA9TPF4				-0.585546706	-0.194816177	0.390730529
A0AAA9TNL3	SLK	A0AAA9TNL3	ENSBTAG00000001017	-0.125132292	-0.106128351	0.019003942
A0A3Q1NC75	CALU	A0A3Q1NC75	ENSBTAG00000016481	0.19636867	0.077919886	-0.118448784
F1MAY4	CCDC88A	F1MAY4	ENSBTAG00000017469	0.017842746	-0.038994132	-0.056836877
A7E306	NID2	A7E306; bta:521854		-0.084160448	-0.166772553	-0.082612105
F1N3I3	UBE2O	bta:789422; F1N3I3	ENSBTAG00000020115	-0.14839184	-0.2393655	-0.09097366
E1BGE2	UTP20	bta:510487; E1BGE2	ENSBTAG00000003239	-0.465604906	-0.32779209	0.137812816
A0AAA9SJ92	RPS6KA1	A0AAA9SJ92	ENSBTAG00000014447	0.123220193	0.187192522	0.063972329
F1MJQ4	P4HA2	F1MJQ4	ENSBTAG00000001116	0.049573061	-0.044477073	-0.094050134
F1MTX7	DARS1	F1MTX7	ENSBTAG00000009949	-0.243745662	-0.169583171	0.074162491
A5PJD6	ATL3	A5PJD6; bta:515124		-0.060784509	-0.063743884	-0.002959375
Q2YDF3	DDX19B	bta:517438; Q2YDF3		-0.204281819	-0.172131295	0.032150524
Q148D3	FH	bta:520260; Q148D3	ENSBTAG00000021688	0.035261104	0.001486548	-0.033774556
Q2TBI4	TRAP1	bta:514472; Q2TBI4	ENSBTAG00000020109	0.008453315	-0.072439665	-0.080892981
F6PUM3	ZYX	F6PUM3	ENSBTAG00000017970	-0.072011245	-0.010847382	0.061163863
F1N3H1	CALU	F1N3H1	ENSBTAG00000016481	0.141375458	-0.056995366	-0.198370823
F1MF97	NID2	F1MF97	ENSBTAG00000021945	-0.075338544	-0.041529573	0.03380897
Q2YDE9	TES	A4D7R3; bta:534965; Q2YDE9	ENSBTAG00000021204	0.088713986	0.086711633	-0.002002353
A2VE14	SAE1	A2VE14; bta:505512	ENSBTAG00000002676	0.05316571	0.07760657	0.024440859

Accession	Gene_Symbol	Gene_ID	Ensembl_Gene ID	log2(ratio(CM12H/CM0H))	log2(ratio(CM12R/CM0H))	log2(ratio(CM12H/CM12R))
Q3ZBH5	PA2G4	bta:540272; F1MLI9; Q3ZBH5	ENSBTAG00000010451	-0.048815025	-0.139562066	-0.090747041
E1BCZ9	ADNP	bta:533757; E1BCZ9	ENSBTAG00000000340	-0.07688309	-0.063279788	0.013603302
P15497	APOA1	A5D7M9; bta:281631; P15497; Q3SZD9		-0.017520099	-0.230452191	-0.212932092
F6RAC6	SRP68	bta:538777; F6RAC6	ENSBTAG00000009228	0.004112198	-0.004123952	-0.00823615
E1BHM5	RBM25	E1BHM5	ENSBTAG00000017177	0.045152036	0.037046987	-0.00810505
E1BKT3	ERBIN	E1BKT3	ENSBTAG00000000415	0.017522827	-0.100375426	-0.117898253
A0AAA9S692	AP3B1	A0AAA9S692	ENSBTAG00000005016	0.027116128	0.056831223	0.029715095
G3MWG4	IARS2	G3MWG4	ENSBTAG00000012582	-0.03796785	-0.027647045	0.010320806
Q2KJI7	AFG3L2	bta:515757; Q2KJI7		0.012773576	0.064130337	0.051356761
A0AAA9T2V7	KIF15	A0AAA9T2V7	ENSBTAG00000016726	-0.143364175	-0.154894267	-0.011530092
A0A3Q1M302	KNL1	A0A3Q1M302	ENSBTAG00000053398	-0.080676646	-0.161879773	-0.081203127
A0A3Q1M5R4	LDHB	A0A3Q1M5R4	ENSBTAG00000019603	0.048094288	0.05399489	0.005900601
A7MB01	SART3	A7MB01; bta:505922		0.025455581	0.168077438	0.142621857
F1MKX7	XRN2	bta:517417; F1MKX7	ENSBTAG00000011304	-0.316051529	-0.255751018	0.06030051
Q2NL22	EIF4A3	bta:515145; Q2NL22	ENSBTAG00000016023	-0.14360813	0.01918702	0.16279515
A0A3Q1MF35	PRPF40A	A0A3Q1MF35	ENSBTAG00000048151	0.00277976	0.012466957	0.009687197
A0A3Q1MM65				-0.085597374	-0.062360666	0.023236709
P61157	ACTR3	A5PJ90; bta:281597; P32391; P61157; Q08DS8		0.051087104	0.052520483	0.001433378
A7Z089	NUP160	A7Z089; bta:540111		-0.004418671	-0.028216242	-0.023797571
P68102	EIF2S1	bta:327694; P05199; P68102; Q3SWZ1	ENSBTAG00000016311	-0.110977229	-0.073502524	0.037474705
P33097	GOT1	bta:281206; P33097; Q2KJF2; Q5E9R4	ENSBTAG00000011960	0.045846504	0.044518667	-0.001327837
F6RPT3	RAP1GDS1	F6RPT3	ENSBTAG00000007522	-0.053888432	-0.163657424	-0.109768992
A0AAA9RSD3	PRKCSH	A0AAA9RSD3	ENSBTAG00000008202	0.152951923	0.048223947	-0.104727976
A0AAF6DLW2	FARSB	A0AAF6DLW2	ENSBTAG00000011649	0.005532874	-0.008339301	-0.013872175
A0AAA9SL80	RPAP3	A0AAA9SL80	ENSBTAG00000018690	0.014721506	-0.01337903	-0.028100536
Q08E14	COL3A1	bta:510833; F1MXS8; Q08E14	ENSBTAG00000021466	0.032494505	-0.547656334	-0.58015084
F2Z4E7	ILF2	F2Z4E7	ENSBTAG00000012471	-0.070734512	-0.030696797	0.040037714
A0A3Q1M2B9	ARID1A	A0A3Q1M2B9	ENSBTAG00000001024	0.114071767	0.059623983	-0.054447784
A0A3Q1M0G8	PAK2	A0A3Q1M0G8; bta:513673	ENSBTAG00000047856	0.025560226	-0.017295253	-0.042855479
Q0IIM6	USP8	bta:538743; F1N3U9; Q0IIM6	ENSBTAG00000011916	-0.184249865	-0.078999389	0.105250476
F1MCA8	TMPO	F1MCA8	ENSBTAG00000004240	0.003174248	0.020508859	0.017334611
A0A3Q1MMR9	PPP6R3	A0A3Q1MMR9	ENSBTAG00000009584	-0.067176708	-0.146325763	-0.079149055
Q08D98	PPP2R5D	bta:100134966; Q08D98		0.139506671	0.185935774	0.046429103
Q2KI84	HARS1	bta:510937; Q2KI84		0.057947349	0.03360353	-0.024343819
A0AAF6YW85	CNN1	A0AAF6YW85	ENSBTAG00000011207	0.137503524	0.193132393	0.055628869
F1N032	DENND4C	bta:536760; F1N032	ENSBTAG00000003097	-0.090286036	-0.110812532	-0.020526495
A0AAA9SWC2	TBCD	A0AAA9SWC2	ENSBTAG00000015414	-0.082544957	-0.146539811	-0.063994854
Q3MHR7	ARPC2	bta:540838; Q3MHR7	ENSBTAG00000010386	-0.015877745	0.010013789	0.025891533
A0A3Q1M9F3	NUP155	A0A3Q1M9F3	ENSBTAG00000002458	-0.080570209	-0.05708737	0.023482839
A0AAF6YQK3	COPS4	A0AAF6YQK3	ENSBTAG00000006950	0.056639071	0.017226414	-0.039412657
E1BP72	CDC42BPB	E1BP72	ENSBTAG00000016456	-0.124522357	-0.026600662	0.097921695
A0JBZ9	p97bcnt2	A0JBZ9; bta:514131	ENSBTAG00000030600	0.089544457	0.184732477	0.095188021
A0AAA9SSX1	RNH1	A0AAA9SSX1	ENSBTAG00000046869	-0.063242434	0.077659666	0.1409021
E1BH06				-0.156659133	-0.060637943	0.096021189
A0AAF6Z0U5	EIF4A2	A0AAF6Z0U5	ENSBTAG00000014724	0.340640355	0.564731564	0.224091209
G3N3P0	BICD2	bta:282447; G3N3P0	ENSBTAG00000046549	0.097458359	-0.031478231	-0.12893659
F1MQU4	BCLAF1	F1MQU4	ENSBTAG00000007802	-0.059181336	-0.063502942	-0.004321606
A0AAA9TYH2	MRE11	A0AAA9TYH2	ENSBTAG00000008925	-0.037224011	-0.012301595	0.024922415
A0AAA9T7E3	RAB3GAP1	A0AAA9T7E3	ENSBTAG00000013449	-0.030012569	0.044565552	0.074578121
A0A3Q1NC33	TCERG1	A0A3Q1NC33	ENSBTAG00000003259	-0.02986464	-0.05742828	-0.02756364
A0AAF6YHR8	ANXA4	A0AAF6YHR8	ENSBTAG00000001105	0.040746342	0.038918989	-0.001827353
A0A3Q1MJD9	ANXA3	A0A3Q1MJD9	ENSBTAG00000010153	-0.041503552	-0.091101078	-0.049597526
F1MUP9	VAT1	F1MUP9	ENSBTAG00000007390	-0.016119665	0.042125476	0.058245141
Q8SPJ1	JUP	bta:445543; Q17QY6; Q8SPJ1	ENSBTAG00000017685	0.141472426	0.098019319	-0.043453107
A0AAA9SDI3	ABCA2	A0AAA9SDI3	ENSBTAG00000047254	-1.971771709	-1.950117343	0.021654366
A0AAA9TGB2	NF1	A0AAA9TGB2	ENSBTAG00000050242	-0.142302229	-0.128769815	0.013532413
E1BMM0	NCBP1	bta:100124428; E1BMM0	ENSBTAG00000009732	0.181218511	0.172887236	-0.008331275
A0A3Q1LTM9	GOLGA2	A0A3Q1LTM9; bta:508805	ENSBTAG00000011317	0.01905474	-0.005490758	-0.024545498
Q9XSK7	HDGF	bta:327953; Q9XSK7	ENSBTAG00000039793	0.101736582	0.015647605	-0.086088977
A0A3Q1MT69	XPO7	A0A3Q1MT69	ENSBTAG00000047628	-0.081993372	0.145452277	0.227445649
Q2KI42	PSMD11	A1L5A1; bta:513461; F1MXM5; Q2KI42		0.017039903	-0.028854863	-0.045894766
A0AAA9TG38	SEC63	A0AAA9TG38	ENSBTAG00000016562	0.042524617	0.052962766	0.010438149
A0AAA9SW45	U2SURP	A0AAA9SW45; bta:505019	ENSBTAG00000009154	0.008453315	-0.015627573	-0.024080889
F1N7U2	NPLOC4	F1N7U2	ENSBTAG00000019105	-0.01504388	0.029625169	0.044669049
A0AAA9TZH9	KIF1B	A0AAA9TZH9	ENSBTAG00000054239	0.082214763	-0.073063462	-0.155278225
A0A3Q1MD15	ANKFY1	A0A3Q1MD15	ENSBTAG00000021091	-0.212389013	-0.166078279	0.046310735
Q28156	PDE5A	bta:281972; Q28156	ENSBTAG00000024888	-0.20139443	-0.150009043	0.051385386
Q0P5L6	ZW10	A0A8J8YST6; bta:511084; F1N3Z5; Q0P5L6; V6F7Q3		-0.139724764	-0.132948354	0.00677641
A0AAF6YKW6	RPL3	A0AAF6YKW6	ENSBTAG00000003228	-0.026808797	-0.074589316	-0.04778052
Q95L54	ANXA8	bta:281627; Q95L54	ENSBTAG00000018499	0.013175389	-0.007583171	-0.02075856
Q148J8	IDH3A	F1MN74; Q148J8	ENSBTAG00000006227	0.013610432	-0.008228321	-0.021838753
A6QL85	RCC2	A6QL85; bta:509120; F1MZE9	ENSBTAG00000008579	-0.071525756	-0.080077619	-0.008551863
A0AAA9TW40	CDC37	A0AAA9TW40	ENSBTAG00000011699	0.160464672	0.121194555	-0.039270118
Q2HJ89	FKBP10	bta:535310; Q2HJ89	ENSBTAG00000011454	-0.035759763	-0.197699668	-0.161939904
A6H742	PLS1	A6H742; bta:616560	ENSBTAG00000023429	-0.062670858	-0.032487853	0.030183005
A0A3Q1MY28	DYNC1I2	A0A3Q1MY28	ENSBTAG00000002646	0.058946007	0.003918595	-0.055027412
A0A3S5ZPK0	OGT	A0A3S5ZPK0	ENSBTAG00000000902	0.085960816	-0.010547165	-0.096507981
E1BP90	ARFGEF1	E1BP90	ENSBTAG00000014691	-0.108106664	-0.026993605	0.081113059
A0AAA9TIX2	SMARCA4	A0AAA9TIX2	ENSBTAG00000019220	0.094387126	0.012531632	-0.081855494
E1B726	PLG	E1B726	ENSBTAG00000001271	-0.288179304	0.188584457	0.47676376
E1BKY9	CPOX	bta:540283; E1BKY9	ENSBTAG00000004124	0.11783649	0.069054117	-0.048782373
A0AAF6YL22	DDX19A	A0AAF6YL22	ENSBTAG00000003570	-0.026047358	-0.004308699	0.021738659
F1MUT3	XDH	F1MKA4; F1MUT3	ENSBTAG00000012519	-0.3825415	-0.198116929	0.184424571
E1BKW5	SEC23IP	E1BKW5	ENSBTAG00000007441	0.141497432	0.156547019	0.015049587
E1BMN2	DIAPH3	E1BMN2	ENSBTAG00000012443	-0.110070778	-0.225929307	-0.11585853
E1BI26	MDC1	E1BI26	ENSBTAG00000025526	0.053291194	0.036186538	-0.017104656

Accession	Gene_Symbol	Gene_ID	Ensembl_Gene ID	log2(ratio(CM12H/CM0H))	log2(ratio(CM12R/CM0H))	log2(ratio(CM12H/CM12R))
A0AAF6YZK2	EIF3H	A0AAF6YZK2	ENSBTAG00000014032	-0.017107256	-0.102778597	-0.085671341
O77834	PRDX6	bta:282438; O77834; Q5E9F3	ENSBTAG00000004855	0.224203146	0.253756592	0.029553446
Q5E9A3	PCBP1	bta:509917; Q5E9A3	ENSBTAG00000008985	0.044311474	0.02433845	-0.019973024
A0A3Q1LKM4	ANKLE2	A0A3Q1LKM4	ENSBTAG00000033339	0.13439594	-0.083466823	-0.217862763
P00432	CAT	bta:531682; P00432; Q3SZ80	ENSBTAG00000020980	0.10864126	0.107145464	-0.001495796
Q07130	UGP2	bta:281565; Q07130; Q17QU0	ENSBTAG00000000111	0.099535674	0.131244533	0.03170886
P80724	BASP1	bta:286842; O02692; P80724; Q08E19	ENSBTAG00000048565	0.208502034	0.169925001	-0.038577032
A0AAA9TRF6	RAB11FIP5	A0AAA9TRF6	ENSBTAG00000006162	0.040718461	-0.069082134	-0.109800595
A7MBG8	RUVBL1	A7MBG8; bta:511475; F1MBF3	ENSBTAG00000020998	0.013229084	-0.055697805	-0.06892689
E1BP36	MMS19	A6QPX1; bta:533499; E1BP36; F1MUW1	ENSBTAG00000012704	-0.101051906	-0.090471591	0.010580315
E1BNH2	CTH	E1BNH2	ENSBTAG00000014791	-0.251360646	-0.130413488	0.120947158
A0AAA9SX99	PAPSS2	A0AAA9SX99	ENSBTAG00000003196	-0.0890603	-0.072336942	0.016723358
G3MXG4	ARHGEF40	bta:527362; G3MXG4	ENSBTAG00000013401	-0.022815895	-0.001509885	0.02130601
P31408	ATP6V1B2	A4FUX5; bta:338082; P31408; Q28058	ENSBTAG00000018646	0.085247287	0.059896954	-0.025350333
P37980	PPA1	bta:280701; P37980; Q1RMH6	ENSBTAG00000007836	0.143446521	0.218687893	0.075241372
F1N3G2	NOP2	F1N3G2	ENSBTAG00000014733	0.011757971	-0.159631547	-0.171389518
E1BI98	COL6A1	bta:511422; E1BI98	ENSBTAG00000011802	-0.225837665	-0.348627343	-0.122789678
Q3MHL4	AHCY	A5D9B8; bta:508158; Q3MHL4	ENSBTAG00000018101	-0.107817734	0.033128782	0.140946516
A6H768	GALK1	A6H768; bta:530855; Q1JP66		-0.06115838	-0.143364175	-0.082205795
F1N719	PPP5C	bta:510245; F1N719	ENSBTAG00000008014	-0.041279739	-0.053488797	-0.012209058
A0AAA9SCC8	SLC25A12	A0AAA9SCC8	ENSBTAG00000016762	-0.212114942	-0.135055985	0.077058957
A6QQR4	ERCC6L	A6QQR4; bta:782916; Q0V8L7	ENSBTAG00000005607	0.16392139	0.11497131	-0.04895008
A0A3Q1M6R3	ELP1	A0A3Q1M6R3	ENSBTAG00000004991	-0.086508993	-0.075206344	0.011302649
F1N650	ANXA1	F1N650	ENSBTAG00000015978	0.109147614	0.100056878	-0.009090736
Q3MHN5	GC	bta:530076; Q3MHN5		-0.464926375	0.091253225	0.5561796
Q24JZ4	MTDH	bta:509788; Q24JZ4	ENSBTAG00000003098	0.099929799	-0.060372618	-0.160302417
A5PK07	WDR44	A5PK07		-0.11323805	-0.162047356	-0.048809305
A5D7C4	TNPO3	A5D7C4; bta:537183	ENSBTAG00000004992	-0.058198247	-0.058198247	0
F1MW01	ESPL1	bta:506740; F1MW01	ENSBTAG00000008934	0.082138287	0.207324973	0.125186687
Q2NKY7	SEPTIN2	bta:511612; Q2NKY7	ENSBTAG00000002608	0.048749648	0.03788476	-0.010864888
E1BGC3	WAPL	bta:534322; E1BGC3	ENSBTAG00000012213	0.233785289	0.203930885	-0.029854404
F1N5M2	GC	F1N5M2	ENSBTAG00000013718	-0.444868248	0.266533062	0.711401309
E1BFB4	MTOR	E1BFB4	ENSBTAG00000015325	-0.078474058	-0.084460353	-0.005986295
E1BCF8	ZC3HAV1	bta:614589; E1BCF8	ENSBTAG00000021617	-0.057209789	-0.165691834	-0.108482045
A0A3Q1MF39	NBAS	A0A3Q1MF39	ENSBTAG00000014081	-0.10637501	-0.124637217	-0.018262206
A0AAA9T8S7	NFKB1	A0AAA9T8S7	ENSBTAG00000020270	0.069162025	0.003216713	-0.065945312
A0A3Q1MR04	NSF	A0A3Q1MR04; bta:504457	ENSBTAG00000008442	0.009277814	0.052229959	0.042952145
F1N1Z2	USP47	F1N1Z2	ENSBTAG00000010919	-0.129963845	-0.080353397	0.049610448
Q5E947	PRDX1	A6QLL7; Q5E947	ENSBTAG00000003642	0.096623889	0.112333972	0.015710083
A7MBC2	PDXDC1	A7MBC2; bta:505868		-0.023549074	-0.026344995	-0.002795921
A0AAF6ZF08	MAPRE1	A0AAF6ZF08	ENSBTAG00000003908	0.098915026	0.059265949	-0.039649077
A0AAA9SKH3	GIGYF2	A0AAA9SKH3	ENSBTAG00000014079	-0.054301384	-0.124493096	-0.070191712
A0A3Q1MJF5	SLC25A13	A0A3Q1MJF5	ENSBTAG00000003773	-0.133759323	0.104433532	0.238192855
G5E567	IPO9	G5E567	ENSBTAG00000004452	-0.185406252	-0.135726649	0.049679603
A0AAA9T365	HNRNPL	A0AAA9T365	ENSBTAG00000001055	0.005501227	0.023236709	0.017735482
A6QQS8	NOP2	A6QQS8; bta:541169		0.087177074	-0.142856758	-0.230033832
F1N1M7	CPT2	F1N1M7	ENSBTAG00000014649	-0.022625059	-0.030246287	-0.007621228
E1BLR9	CPD	E1BLR9	ENSBTAG00000012371	0.042114874	0.054938914	0.01282404
A0AAA9THK8	POLA1	A0AAA9THK8	ENSBTAG00000008170	-0.257955766	-0.210930431	0.047025335
Q3ZBX5	AIMP1	bta:505126; F6PWM7; Q3ZBX5	ENSBTAG00000004230	0.103493422	0.095373166	-0.008120256
E1BBH1	PHLDB2	E1BBH1	ENSBTAG00000001209	0.29498503	0.324468052	0.029483022
A1L5B7	SERBP1	A1L5B7; F1MFK6		0.191463348	0.536921601	0.345458253
A0A3Q1MG97	LNPEP	A0A3Q1MG97	ENSBTAG00000019900	-0.080949108	0.130191039	0.211140147
A5D9H5	HNRNPD	A5D9H5; bta:527471	ENSBTAG00000013952	-0.175218674	-0.091474005	0.08374467
A5D7P7	ABCB6	A5D7P7; bta:783257	ENSBTAG00000020607	-0.813152767	-0.741380885	0.071771882
A0AAF6DM04	SERBP1	A0AAF6DM04	ENSBTAG00000012957	0.038003572	-0.095219748	-0.13322332
A0AAA9T5N1	EEF1D	A0AAA9T5N1	ENSBTAG00000014643	0.012466957	-0.084509524	-0.096976481
A0AAA9S435	PRPF6	A0AAA9S435	ENSBTAG00000011656	-0.15065415	-0.090502584	0.060151566
Q2KJA2	ABCF2	bta:513061; Q1JPK5; Q2KJA2		-0.039048726	-0.171919052	-0.132870325
E1BJQ1	GLDC	bta:507688; E1BJQ1	ENSBTAG00000025903	-0.272115128	-0.196691911	0.075423217
E1B7Q0	HOOK3	bta:524648; E1B7Q0	ENSBTAG00000007634	0.159364861	0.087790466	-0.071574396
A0AAA9SA75	SYTL2	A0AAA9SA75	ENSBTAG00000000460	-0.018229241	0.122724711	0.140953953
F1N7T2	MAN2A1	F1N7T2	ENSBTAG00000025029	0.11318099	0.141920886	0.028739895
F1MXE4	PSMD6	F1MXE4	ENSBTAG00000015112	0.089637212	-0.012383724	-0.102020937
A1L5A7	DBN1	A1L5A7	ENSBTAG00000011400; ENSBTAG00000069499	-0.157436979	-0.13195495	0.025482029
A0A3Q1MMI6	EPS15L1	A0A3Q1MMI6	ENSBTAG00000009990	0.059484433	0.08446679	0.024982357
A0A3Q1MTV4	GPD2	A0A3Q1MTV4	ENSBTAG00000009770	0.153565759	0.258288694	0.104722935
F1N0F7	EIF5	bta:504752; F1N0F7	ENSBTAG00000016282	-0.108268111	-0.088195508	0.020072603
E1BJV0	EHD4	bta:505206; E1BJV0	ENSBTAG00000006792	-0.062260059	-0.169307542	-0.107047482
Q3ZC50	CCDC47	A5D9E2; bta:540167; Q3ZC50	ENSBTAG00000011834	0.069911693	0.033980649	-0.035931044
A0AAA9T9F6	CUL4B	A0AAA9T9F6	ENSBTAG00000018569	-0.012174714	-0.00303406	0.009140654
A6QLD1	EXOC4	A6QLD1; bta:537690; F1MNJ7	ENSBTAG00000014112	0.086724701	0.088200604	0.001475903
P12763	AHSG	A6QLF7; bta:280988; P12763		-0.697072867	-0.120081077	0.57699179
A5PJY9	CPNE3	A5PJY9; bta:615304; E1B819	ENSBTAG00000015536	-0.078242023	-0.020258839	0.057983184
F1MYA8	ATP13A1	bta:534369; F1MYA8	ENSBTAG00000007128	-0.093751356	-0.074797031	0.018954326
Q3SZH7	LTA4H	bta:507130; Q3SZH7	ENSBTAG00000016415	0.103205282	0.209365741	0.106160459
A0A3Q1LY87	ENAH	A0A3Q1LY87	ENSBTAG00000016185	0.096797591	0.145093148	0.048295557
A0A3Q1MN56	PHLDB2	A0A3Q1MN56	ENSBTAG00000001209	-0.059106617	-0.04673806	0.012368557
A0AAA9U1C1	TFRC	A0AAA9U1C1	ENSBTAG00000032719	-0.15704371	-0.054945523	0.102098188
A0A3Q1MR38	PRMT1	A0A3Q1MR38	ENSBTAG00000006646	-0.145458392	-0.147500424	-0.002042032
A0AAA9RVW3	UGGT2	A0AAA9RVW3	ENSBTAG00000004401	-0.283025881	-0.315599872	-0.032573991
A0AAA9SSF0	SCFD1	A0AAA9SSF0; bta:100140328	ENSBTAG00000017565	-0.043106573	0.047909985	0.091016558
F1N331	HMMR	F1N331	ENSBTAG00000014773	0.482686192	0.438370003	-0.044316188
A0A3Q1MJ08	NEDD4L	A0A3Q1MJ08	ENSBTAG00000005412	-0.010673502	-0.035889787	-0.025216286
E1B864	ITGB4	E1B864	ENSBTAG00000018169	-0.179962678	-0.257102076	-0.077139398
A0AAA9TH40	AIFM1	A0AAA9TH40	ENSBTAG00000006838	0.078656944	0.143427199	0.064770255
A5PKK6	AHCYL1	A5PKK6; bta:505511		-0.151487753	-0.086013916	0.065473837

Accession	Gene_Symbol	Gene_ID	Ensembl_Gene ID	log2(ratio(CM12H/CM0H))	log2(ratio(CM12R/CM0H))	log2(ratio(CM12H/CM12R))
A0AAA9TRR7	HIP1R	A0AAA9TRR7	ENSBTAG00000021595	-0.15929345	-0.130973554	0.028319896
Q2HJ33	OLA1	bta:509966; Q2HJ33	ENSBTAG00000006970	0.066375678	0.05504926	-0.011326418
Q9XSA7	CLIC4	bta:286823; Q3ZBJ4; Q9XSA7	ENSBTAG00000009470	0.123534993	0.057350443	-0.066184549
F1MZ21	SART1	bta:615704; F1MZ21	ENSBTAG00000003094	0.152703261	-0.036885068	-0.189588329
G3N2L2	RCN1	G3N2L2	ENSBTAG00000047362	0.149174972	0.055428475	-0.093746496
A0A3Q1MUN6	SEPTIN11	A0A3Q1MUN6	ENSBTAG00000021372	-0.070144824	-0.032977322	0.037167502
A0A3Q1MNL1	CUL4A	A0A3Q1MNL1	ENSBTAG00000020093	-0.237180368	-0.200382751	0.036797618
E1B8H0	PEAK1	E1B8H0	ENSBTAG00000005145	0.163636247	0.071366929	-0.092269318
Q0VCV0	RPA1	A0A3Q1MBP4; bta:504844; Q0VCV0	ENSBTAG00000009711	-0.151351617	0.065914117	0.217265734
A0AAA9SYH6	CACYBP	A0AAA9SYH6	ENSBTAG00000001107	0.448534411	0.350687088	-0.097847323
F1N153				-0.228341688	-0.179978666	0.048363022
A0A140T8A5	IDH1	A0A140T8A5; bta:281235	ENSBTAG00000020527	0.102726302	0.146669649	0.043943348
A0A3Q1LW48	EMC1	A0A3Q1LW48	ENSBTAG00000011950	0.115355661	-0.082926991	-0.198282652
A5PKM3	SPTLC2	A5PKM3; bta:537972	ENSBTAG00000020480	-0.119581616	-0.080053252	0.039528364
A0A3Q1LVN5	DDX42	A0A3Q1LVN5	ENSBTAG00000021058	-0.128924539	-0.014412658	0.114511881
Q0VCX5	ETF1	bta:541077; Q0VCX5	ENSBTAG00000011415	-0.150158215	0.028786319	0.178944534
F1MD37	PPFIA1	F1MD37	ENSBTAG00000022188	0.099324738	0.034388726	-0.064936012
A0A3Q1NN09	RBM10	A0A3Q1NN09	ENSBTAG00000002890	-0.079460516	-0.049147649	0.030312867
Q3T0G3	AHSA1	bta:539220; Q3T0G3	ENSBTAG00000020477	0.375373282	0.313754163	-0.061619118
A0AAA9SY77	ZC3H4	A0AAA9SY77	ENSBTAG00000009286	0.051981255	0.09367639	0.041695135
A0A3Q1LMN6	PXDN	A0A3Q1LMN6; bta:515860	ENSBTAG00000050986	-0.010904776	0	0.010904776
A0A3Q1M4B7	ESYT2	A0A3Q1M4B7	ENSBTAG00000005483	-0.017821277	-0.12963528	-0.111814003
Q29RL9	TCEA1	bta:505722; Q29RL9		0.043027284	0.026523443	-0.016503841
A0AAA9RW91	KIF23	A0AAA9RW91	ENSBTAG00000009983	-0.069610851	0.052821325	0.122432176
A0A3Q1MRT9	LRRC40	A0A3Q1MRT9; bta:526961	ENSBTAG00000008243	-0.043370852	-0.024416527	0.018954326
Q8WN55	PTBP1	A0A3Q1M994; bta:282018; Q8WN55	ENSBTAG00000045828	0	0.069489655	0.069489655
Q24JZ7	OXCT1	bta:535804; F1MTP3; Q24JZ7	ENSBTAG00000033186	0.054255796	0.111351236	0.05709544
A0AAA9S704	ACOX1	A0AAA9S704	ENSBTAG00000030174	-0.282111199	-0.221725279	0.06038592
A0A3Q1M7N2	DHX30	A0A3Q1M7N2	ENSBTAG00000015833	-0.320367581	-0.29716019	0.023207391
A0A3Q1N6T1	CYB5R3	A0A3Q1N6T1	ENSBTAG00000016516	0.164052656	0.113327684	-0.050724972
A0AAA9TSW3	CTPS1	A0AAA9TSW3	ENSBTAG00000001626	0.042983171	0.066783629	0.023800458
A6H7H6	CDH17	A6H7H6; bta:507526	ENSBTAG00000021964	0.043284098	-0.029594782	-0.07287888
A0A3Q1M507	CPT1A	A0A3Q1M507; bta:506812	ENSBTAG00000021999	-0.014059944	0.134957341	0.149017285
Q5E9E2	MYL9	bta:531505; Q5E9E2	ENSBTAG00000016024	0	-0.070091896	-0.070091896
A0AAA9SRV2	ATXN2	A0AAA9SRV2	ENSBTAG00000022069	-0.058326703	-0.064006612	-0.005679909
Q2T9U1	SRP54	bta:614017; Q2T9U1	ENSBTAG00000019085	-0.079626175	-0.121841123	-0.042214948
F1MSJ6	TBC1D8B	F1MSJ6	ENSBTAG00000046314	-0.105594659	-0.083328384	0.022266276
A0A3Q1M2P8	EPS8	A0A3Q1M2P8	ENSBTAG00000000369	0.06666394	-0.095532437	-0.162196376
A0A3Q1M7H7	DNPEP	A0A3Q1M7H7	ENSBTAG00000021514	-0.140433705	-0.179364697	-0.038930993
P28339	POLD1	bta:281990; P28339		-0.376944328	-0.157912484	0.219031844
A0A3Q1MMW5				-0.115033243	-0.097823953	0.01720929
A4FUY3	LOC539976	A4FUY3; bta:539976		-0.026752728	-0.127942552	-0.101189824
E1BJG5	TRIM28	E1BJG5	ENSBTAG00000006422	0.019365325	0.029686131	0.010320806
P07857	SCP2	bta:508918; P07857; Q3SYV0		0.051912089	0	-0.051912089
A0AAA9TCP2	DCTN2	A0AAA9TCP2	ENSBTAG00000010624	-0.026499225	0.037437301	0.063936527
F1MHP6	ADSL	F1MHP6	ENSBTAG00000000077	-0.028408415	0.014660307	0.043068722
F1N2Y2	COL5A2	F1N2Y2	ENSBTAG00000027020	-0.330293037	-0.577902811	-0.247609774
E1BMC6	SLMAP	E1BMC6	ENSBTAG00000021164	-0.157473775	-0.231783472	-0.074309697
F1MG56	PPP2R2A	F1MG56	ENSBTAG00000018317	0.134334732	0.095189442	-0.03914529
A0AAA9TXG2	COL6A2	A0AAA9TXG2	ENSBTAG00000019269	-0.098279518	-0.611597627	-0.51331811
A0AAF7AGM7	TAGLN2	A0AAF7AGM7	ENSBTAG00000002068	-0.043768753	-0.031127314	0.012641439
A0AAA9T8H1	SUGP2	A0AAA9T8H1	ENSBTAG00000013288	0.520484693	0.607085312	0.086600618
F1MS08	SLC12A2	F1MS08	ENSBTAG00000009637	-0.119894124	-0.134974697	-0.015080573
A0AAA9T5C9	MAPK1	A0AAA9T5C9	ENSBTAG00000010312	0.119654316	0.115231129	-0.004423187
A0AAA9SUJ2	APEX1	A0AAA9SUJ2	ENSBTAG00000002745	0.049070607	0.171355537	0.12228493
Q3ZC44	HNRNPAB	bta:513410; Q3ZC44	ENSBTAG00000013627	0.022997032	-0.027533184	-0.050530216
A0AAA9TGR1	GFM1	A0AAA9TGR1	ENSBTAG00000027795	-0.25763307	-0.247318853	0.010314218
A0AAA9SQ91	PSIP1	A0AAA9SQ91	ENSBTAG00000007872	-0.120718939	-0.070040556	0.050678383
P63103	YWHAZ	bta:287022; P29213; P29312; P63103; Q3ZCF9	ENSBTAG00000000236	0.14340707	0.092216371	-0.051190699
A5D7S0	NAPA	A5D7S0; bta:518407		0.064964729	0.033569533	-0.031395196
A0AAA9RSP8	ASPH	A0AAA9RSP8	ENSBTAG00000026283	0.098996247	-0.013546532	-0.112542779
A0A3Q1N7W0	PRPF3	A0A3Q1N7W0	ENSBTAG00000013946	0.096532795	0.00841224	-0.088120555
A0AAA9T6Y7	MON2	A0AAA9T6Y7	ENSBTAG00000011068	-0.056131875	0.009847786	0.065979661
Q04467	IDH2	bta:327669; Q04467; Q3ZCD4	ENSBTAG00000014093	-0.023103069	-0.067918598	-0.044815529
Q59A30	son	bta:516462; Q59A30		-0.056100474	-0.047753131	0.008347343
F1MU12				0.363932237	-0.615956067	-0.979888304
E1BKC4	NIPBL	bta:539566; E1BKC4	ENSBTAG00000000579	-0.110869221	-0.050867314	0.060001907
F6QZ79				-0.095635279	-0.055742552	0.039892727
A0AAA9SV11	RPN2	A0AAA9SV11	ENSBTAG00000014648	-0.077846587	-0.127573636	-0.049727049
A0AAA9STQ6	G3BP1	A0AAA9STQ6	ENSBTAG00000020309	-0.131244533	-0.121048766	0.010195767
F1ME46	LMAN1	F1ME46	ENSBTAG00000006833	-0.005444139	-0.068149592	-0.062705453
A0AAA9TIZ3	NAV1	A0AAA9TIZ3	ENSBTAG00000021919	-0.02075856	-0.14344108	-0.12268252
A0A3Q1M166	HSPA12A	A0A3Q1M166; bta:540050	ENSBTAG00000013779	0.003584337	-0.054861935	-0.058446272
F1MJP7	UAP1	F1MJP7	ENSBTAG00000010206	-0.076941857	-0.056936165	0.020005692
Q1RMR3	CTTN	bta:506939; Q1RMR3	ENSBTAG00000006071	0.033637597	0.062263437	0.028625841
A0AAA9TPR5	DPP9	A0AAA9TPR5	ENSBTAG00000021134	-0.140280608	-0.116844053	0.023436555
E1BNS3	BRD4	E1BNS3	ENSBTAG00000002495	0.095706355	0.001433378	-0.094272976
F6PTU3	SUN2	F6PTU3	ENSBTAG00000003693	-0.083978393	-0.111546653	-0.02756826
F6R6W2	DDX23	F6R6W2	ENSBTAG00000021810	0.104814905	0.046262723	-0.058552182
G3MZI7	COL5A1	G3MZI7	ENSBTAG00000047998	-0.233363539	-0.967069725	-0.733706186
Q08DZ4	SMTN	bta:528013; F1N031; Q08DZ4		-0.277755912	-0.300123725	-0.022367813
F1MSZ6	SERPINC1	F1MSZ6	ENSBTAG00000004362	-0.537416916	-0.470629825	0.066787091
A0AAA9T691	PPP4R1	A0AAA9T691	ENSBTAG00000009822	-0.036674447	-0.038160995	-0.001486548
A1L5C2	ADFP	A1L5C2		0.359967456	0.266929559	-0.093037897
A0AAA9SEC9	SPECC1	A0AAA9SEC9	ENSBTAG00000000928	-0.145002389	-0.064583706	0.080418682
A0A3Q1N369	CD2AP	A0A3Q1N369	ENSBTAG00000000322	0.083681722	0.003374727	-0.080306996
Q2HJ94	DNAJA2	bta:360006; Q2HJ94	ENSBTAG00000003757	0.177465103	0.004159623	-0.173305479
A5PK45	COLGALT1	A5PK45; bta:513167	ENSBTAG00000012678	0.086525844	0.127917475	0.041391631
A7Z035	CLINT1	A7Z035; bta:538540	ENSBTAG00000016199	0.114770187	0.157963704	0.043193517
A0AAA9S063	MAP4K4	A0AAA9S063	ENSBTAG00000013023	-0.022048749	0.008726005	0.030774753

Accession	Gene_Symbol	Gene_ID	Ensembl_Gene ID	log2(ratio(CM12H/CM0H))	log2(ratio(CM12R/CM0H))	log2(ratio(CM12H/CM12R))
Q3SYU6	CNN2	bta:539019; Q3SYU6	ENSBTAG00000020764	-0.106414268	-0.066649035	0.039765233
P68509	YWHAH	bta:282126; P11576; P68509; P70198; Q3ZC14	ENSBTAG00000031134	0.061692322	0.048209093	-0.013483229
F6Q8A8	NUCB2	F6Q8A8	ENSBTAG00000017468	0.068421732	0.087389236	0.018967505
A7YWC4	ATAD3	A7YWC4; bta:784353	ENSBTAG00000002219	-0.047488926	-0.106915204	-0.059426278
F1MLJ5	OSBPL3	F1MLJ5	ENSBTAG00000019342	0.026523443	0.09011242	0.063588977
A0A3Q1MXM6	NEDD4	A0A3Q1MXM6; bta:507781	ENSBTAG00000013793	-0.02629445	-0.004120027	0.022174424
Q2KJ42	NONO	bta:615175; Q2KJ42	ENSBTAG00000012152	-0.087548474	-0.102180395	-0.014631921
A0AAF6Z3R0	YBX1	A0AAF6Z3R0	ENSBTAG00000017368	-0.080011514	-0.066849481	0.013162033
Q2KIK0	SUGT1	bta:515509; Q2KIK0		0.085167383	0.184090317	0.098922934
G3MYQ1	FHOD1	bta:787862; G3MYQ1	ENSBTAG00000032427	-0.214580615	-0.204699104	0.009881512
A4IFE6	ABCE1	A4IFE6; bta:514991	ENSBTAG00000019854	-0.112813174	-0.114318333	-0.001505159
A0JN97	PRKCD	A0JN97; bta:505708		-0.175961391	-0.111091551	0.06486984
A0AAF6ZQF2	TUBG1	A0AAF6ZQF2	ENSBTAG00000048152	-0.052861814	-0.107734452	-0.054872637
E1BIX2	POLR1A	bta:536316; E1BIX2	ENSBTAG00000006481	-0.151523713	-0.045293387	0.106230326
A6H7G2	DBNL	A6H7G2; bta:514706		-0.011379062	0.05246742	0.063846482
A0A3Q1LKM6	TFG	A0A3Q1LKM6	ENSBTAG00000016363	0.057913303	0.083982682	0.026069379
A0AAA9TYA8	ITSN1	A0AAA9TYA8	ENSBTAG00000021997	-0.032653217	0.013530651	0.046183868
A6QLA8	TRIM25	A6QLA8; bta:510923; F1MTX8	ENSBTAG00000009948	0.111141027	0.179439114	0.068298086
A0A3Q1MUZ8	SH3KBP1	A0A3Q1MUZ8	ENSBTAG00000003727	0.099193357	0.070144824	-0.029048533
A0AAA9TV55	GNE	A0AAA9TV55	ENSBTAG00000001142	0.083951778	0.025116677	-0.058835101
A0AAF6ZAI1	CNDP2	A0AAF6ZAI1	ENSBTAG00000009841	-0.014911709	-0.10178916	-0.086877451
Q29RT6	KIF20A	bta:527854; Q29RT6		-0.077099112	0.095609743	0.172708855
Q0V7M0	KPNA6	bta:531602; Q0V7M0	ENSBTAG00000002896	0.103736218	0.065875131	-0.037861087
F1MG91	LOC281376	bta:281376; F1MG91	ENSBTAG00000023026	-0.296077969	-0.323639477	-0.027561508
Q3SYV6	KPNA2	bta:513592; Q3SYV6	ENSBTAG00000012225	0.019916728	-0.117463177	-0.137379905
A6QNL5	PDIA6	A6QNL5; bta:613927		0.390678927	0.412989089	0.022310162
P63243	RACK1	bta:327682; P25388; P63243; P99049; Q3T0R8		-0.136451805	-0.164182761	-0.027730956
F1MDS3	PCK2	bta:282856; F1MDS3	ENSBTAG00000011934	-0.147452001	-0.036350568	0.111101433
E1BBQ0	KNTC1	bta:506353; E1BBQ0	ENSBTAG00000019278	-0.489572155	-0.212080914	0.277491241
A0AAA9S5L9	BAG6	A0AAA9S5L9	ENSBTAG00000019685	-0.280277978	-0.306536044	-0.026258066
A0AAF6Z7A1	UBE4A	A0AAF6Z7A1	ENSBTAG00000019725	0.174535307	0.115477217	-0.059058089
F1MBP8	COPS5	bta:507179; F1MBP8	ENSBTAG00000002784	-0.087462841	-0.030526676	0.056936165
Q1RMW3	MTA2	bta:515389; Q1RMW3		-0.012862151	-0.037474705	-0.024612554
A0A3Q1N814	FAM114A2	A0A3Q1N814	ENSBTAG00000017078	0.125908897	0.066495412	-0.059413486
A0A3Q1NF03	RANGAP1	A0A3Q1NF03	ENSBTAG00000012481	0.004175676	-0.030996594	-0.03517227
E1B8Q8	XPOT	E1B8Q8	ENSBTAG00000026962	-0.157204343	-0.085729874	0.071474469
A0A3Q1M0B4	PTPN12	A0A3Q1M0B4; bta:531877	ENSBTAG00000003825	-0.214944315	-0.128443183	0.086501132
A0A3Q1LVH9	SH3PXD2B	A0A3Q1LVH9	ENSBTAG00000009019	-0.003191804	0.015853951	0.019045756
Q2PQT6				0.092841021	-0.028912692	-0.121753713
Q28104	COPE	A1L5B2; bta:338085; Q28104; Q2TBL7; Q9N286	ENSBTAG00000000810	-0.282496488	-0.21861859	0.063877897
A0AAA9T7E7	PNPT1	A0AAA9T7E7	ENSBTAG00000008909	-0.082383657	-0.044727806	0.037655851
Q2KHZ2	HBS1L	bta:541083; Q2KHZ2		0.132806737	0.161040418	0.028233681
A6QNS2	UBE4B	A0A3Q1LPU1; A6QNS2; bta:520833	ENSBTAG00000010401	-0.06652151	-0.224007077	-0.157485567
F1MYU1	SMARCC1	F1MYU1	ENSBTAG00000031567	0.0662156	-0.031395196	-0.097610797
A0AAF6YZS2	PRKAR2A	A0AAF6YZS2	ENSBTAG00000014205	0.128965557	0.199769512	0.070803955
A0A3Q1LGY1	PUF60	A0A3Q1LGY1	ENSBTAG00000037493	-0.012735988	0.016808288	0.029544276
Q1LZF9	MYL9	bta:505964; F1MLA6; Q1LZF9	ENSBTAG00000011473	-0.224737886	-0.339256911	-0.114519025
F1MMD5	XRCC6	F1MMD5	ENSBTAG00000006103	-0.114203723	-0.080011514	0.034192209
G1K1X0	UQCRC1	G1K1X0	ENSBTAG00000019096	-0.086975965	-0.112018552	-0.025042587
A0A3Q1MTX3	ADD1	A0A3Q1MTX3	ENSBTAG00000021128	-0.001509885	-0.072730324	-0.071220439
E1BGB2	PRRC2B	E1BGB2	ENSBTAG00000004400	-0.195465965	-0.209083533	-0.013617569
A6QLU8	NXN	A6QLU8; bta:531367	ENSBTAG00000000855	0.06247697	-0.041641337	-0.104118307
E1BB16	GCC2	bta:539484; E1BB16	ENSBTAG00000016038	-0.138319605	-0.108694436	0.029625169
A0AAA9TK37	ACTR1A	A0AAA9TK37	ENSBTAG00000021067	0.016201952	0.023506753	0.007304801
A6H7E3	PDLIM1	A6H7E3	ENSBTAG00000011182	0.020102904	-0.010837197	-0.0309401
E1BEI0	API5	E1BEI0	ENSBTAG00000014474	0.277344732	0.189803821	-0.087540911
A0A3Q1MN26	FOCAD	A0A3Q1MN26	ENSBTAG00000038181	-0.085900073	-0.075610814	0.010289259
A0A3Q1M1M5	TMEM43	A0A3Q1M1M5	ENSBTAG00000007360	-0.002990043	-0.02713613	-0.024146087
Q3SZI4	YWHAQ	A7E3X8; bta:768311; Q3SZI4	ENSBTAG00000002108	0.114922783	0.105576276	-0.009346507
A6H7H2	KLC1	A6H7H2; bta:508999	ENSBTAG00000017299	-0.014978563	-0.037046987	-0.022068423
F1MH07	MICAL1	A2VDL7; bta:508306; F1MH07		-0.15324626	-0.165737204	-0.012490944
F1MBA3	ITGA2	bta:281872; F1MBA3	ENSBTAG00000019289	-0.158947775	-0.06598841	0.092959366
F1MJM0	ZNF326	bta:533643; F1MJM0; Q0VCF6		0.079052563	0.092485575	0.013433012
F1N3S4	CUL4A	F1N3S4	ENSBTAG00000020093	0.033300009	-0.055236665	-0.088536675
Q08DU9	HP1BP3	bta:510194; Q08DU9	ENSBTAG00000018656	-0.072479375	0.15521265	0.227692025
G5E5X0	LIMD1	bta:617525; G5E5X0	ENSBTAG00000026097	-0.143029774	-0.034891544	0.10813823
A0AAF6YVA6	EIF3L	A0AAF6YVA6	ENSBTAG00000010790	-0.048264954	-0.092054978	-0.043790024
Q2HJD8	RBM39	bta:508722; Q2HJD8		0.251659616	0.150491627	-0.101167989
A0AAA9RXM0	UFL1	A0AAA9RXM0	ENSBTAG00000020410	0.072706445	0.072706445	0
A0A3Q1MQ76	SUPT5H	A0A3Q1MQ76	ENSBTAG00000010467	0.074299555	0.0456168	-0.028682755
Q3B7N3	IPO5	Q3B7N3	ENSBTAG00000039374	-0.045065172	0.039528364	0.084593536
F1MTS8	LTN1	F1MTS8	ENSBTAG00000000201	-0.108856302	-0.186779302	-0.077923001
A0A3Q1N7P8	ITPR2	A0A3Q1N7P8	ENSBTAG00000002313	-0.108805941	-0.068752691	0.04005325
A5D7E1	EHD3	A5D7E1; bta:510183	ENSBTAG00000001662	0.015963593	-0.179235179	-0.195198772
Q56JV9	RPS3A	A2V9E4; B0JYM2; bta:282053; Q56JV9	ENSBTAG00000009908	-0.12541347	-0.068775755	0.056637715
A0A3Q1LPP0	KIF1C	A0A3Q1LPP0	ENSBTAG00000001349	0.039057476	0.015750102	-0.023307373
A0A452DJI0	MICAL3	A0A452DJI0	ENSBTAG00000048271	-0.079779074	-0.094327383	-0.014548309
G3MZY8	POLR2A	bta:282312; G3MZY8	ENSBTAG00000046765	-0.28761513	-0.146046038	0.141569092
F1MHC2	STXBP2	F1MHC2	ENSBTAG00000009178	-0.01098305	0.026330109	0.037313158
F1MMW4	AKAP8L	bta:539700; F1MMW4	ENSBTAG00000010439	0.64202411	0.337105623	-0.304918487
A0A452DIX3	TPI1	A0A452DIX3	ENSBTAG00000019782	0.162180878	0.244713467	0.082532589
A0A3Q1M7R3	ATXN2	A0A3Q1M7R3	ENSBTAG00000022069	-0.26660733	-0.343758343	-0.077151013
A0AAA9TDA2	NUFIP2	A0AAA9TDA2; bta:781847	ENSBTAG00000033077	0.114139531	0.011541622	-0.102597909
A0A3Q1M1T4	CKAP2	A0A3Q1M1T4	ENSBTAG00000021162	0.474771035	0.530638985	0.05586795
Q58DN4	PPME1	bta:535390; Q58DN4	ENSBTAG00000027612	0.089539006	-0.01227833	-0.101817336
E1BMF9	LLGL1	E1BMF9	ENSBTAG00000004635	-0.145407905	-0.153656612	-0.008248708
A4IFC8	LIG1	A4IFC8; bta:100124507; F1MSC6		0.148370858	-0.041040684	-0.189411543
A0AAA9TKS4	NSFL1C	A0AAA9TKS4	ENSBTAG00000006533	0.086340849	0.026761068	-0.059579781

Accession	Gene_Symbol	Gene_ID	Ensembl_Gene ID	log2(ratio(CM12H/CM0H))	log2(ratio(CM12R/CM0H))	log2(ratio(CM12H/CM12R))
E1B7Q6	MED1	bta:520734; E1B7Q6	ENSBTAG00000013236	-0.2793824	-0.179641229	0.099741171
Q5E964	PSMD13	bta:513525; Q3T0Z1; Q5E964		-0.001377275	-0.011055189	-0.009677914
A0A3Q1MT26	PARD3	A0A3Q1MT26	ENSBTAG00000014991	-0.119900846	-0.256842572	-0.136941727
A0AAA9RX06	LYAR	A0AAA9RX06	ENSBTAG00000003653	0.126102722	0.056530732	-0.06957199
P08166	AK2	bta:280716; P08166; P08167; Q2KIJ7	ENSBTAG00000017605	0.043356369	-0.02134932	-0.064705689
A0A3Q1LPW5	KIF22	A0A3Q1LPW5	ENSBTAG00000013669	0.151457032	0.004088888	-0.147368144
F1MPE9	NOTCH2	F1MPE9	ENSBTAG00000007909	0.079759851	0.240073257	0.160313407
F1MRE5	ACBD3	bta:783375; F1MRE5	ENSBTAG00000013128	0.129196236	0.223259947	0.094063711
A0A3Q1MNI4	SSRP1	A0A3Q1MNI4	ENSBTAG00000000375	0.010482844	0.027356662	0.016873819
A0A452DJ13	NDUFV1	A0A452DJ13	ENSBTAG00000021776	-0.097550333	-0.09884481	-0.001294477
A0A3Q1MH34	DCTN4	A0A3Q1MH34	ENSBTAG00000015625	0.011669993	0	-0.011669993
F6RQC9	GNAI2	F6RQC9	ENSBTAG00000020645	-0.045954633	0.116796335	0.162750969
O02691	HSD17B10	bta:281809; O02691; Q2TBG6		0.045540784	0.081760972	0.036220188
A0AAA9SQ31	TXNL1	A0AAA9SQ31	ENSBTAG00000015731	-0.091209871	0.002848362	0.094058234
F1MI47	RBM14	F1MI47	ENSBTAG00000001225	0.154052169	0.050373523	-0.103678646
Q3T147	DDX39B	bta:540191; Q3T147		0.035208098	0.027626919	-0.007581179
A7MBD4	FARSA	A7MBD4; bta:522024	ENSBTAG00000021374	-0.036103974	-0.019328261	0.016775713
A0AAA9RW75	ITIH2	A0AAA9RW75	ENSBTAG00000014075	-0.394885836	0.122105314	0.51699115
G3MXV0	FKBP5	bta:535704; G3MXV0	ENSBTAG00000047502	0.282131902	0.208056804	-0.074075099
F1MPG7	NAT10	F1MPG7	ENSBTAG00000016747	0.004081177	-0.198300036	-0.202381213
F6RV75	TRIOBP	F6RV75	ENSBTAG00000002539	0.098924233	0.09177154	-0.007152692
A0AAA9TT27	ATRX	A0AAA9TT27	ENSBTAG00000038434	-0.316973213	-0.184725415	0.132247798
E1BHK2	ADD1	E1BHK2	ENSBTAG00000021128	-0.066466909	-0.17484307	-0.108376161
F6Q5B6	EIF2AK2	F6Q5B6	ENSBTAG00000008703	0.164238134	0.222195184	0.05795705
F1MYQ3	RIPOR1	F1MYQ3	ENSBTAG00000008860	-0.073773487	-0.043809896	0.029963591
Q8SQH5	SLC25A5	bta:282479; Q3SZD0; Q8SQH5		0.077318284	0.093650855	0.016332571
P11180	DLAT	bta:512723; F1N690; P11180; V6F7T1	ENSBTAG00000010709	0.005702359	-0.042030251	-0.047732611
A0A3Q1MTE4	ERO1A	A0A3Q1MTE4	ENSBTAG00000015716	0.131988353	0.064359246	-0.067629107
F1MZN7	SNX2	F1MZN7	ENSBTAG00000025358	0.094443204	0.196452668	0.102009464
A0AAF6ZJJ6	ITGA3	A0AAF6ZJJ6	ENSBTAG00000019498	-0.155002461	-0.140067596	0.014934865
Q2KJC8	FKBP9	bta:534182; Q2KJC8	ENSBTAG00000016707	0.003105911	-0.084841788	-0.087947699
A0A3Q1LY89	MYEF2	A0A3Q1LY89	ENSBTAG00000008771	-0.113267616	-0.014034109	0.099233507
A0AAA9S2J2	AGO2	A0AAA9S2J2	ENSBTAG00000001579	-0.039289131	-0.014427071	0.02486206
A0A3Q1LW57	FNDC3A	A0A3Q1LW57	ENSBTAG00000020681	-0.028123601	-0.00697967	0.021143931
Q2HJ58	PRPS1	A5PKB5; bta:781227; Q2HJ58		0.056812092	0.023578789	-0.033233303
A6QR01	LRRC47	A6QR01; bta:616209; F1MKE3		0.099054213	0.096355064	-0.00269915
A0AAA9SJ86	CUX1	A0AAA9SJ86	ENSBTAG00000010659	-0.416960837	-0.137849535	0.279111301
A0AAA9TGU4	FMNL3	A0AAA9TGU4	ENSBTAG00000016593	-0.163740613	-0.184204715	-0.020464103
F1N7G0	HNRNPLL	F1N7G0	ENSBTAG00000020567	-0.208586622	-0.025188332	0.18339829
A0A452DI31	ENO3	A0A452DI31	ENSBTAG00000005534	0.229365163	0.099623828	-0.129741334
A0AAA9S8L6	STAU1	A0AAA9S8L6	ENSBTAG00000000241	0.143789787	0.055613975	-0.088175811
Q2KHU8	EIF2S3	A5D972; bta:512350; Q2KHU8	ENSBTAG00000014337	-0.046145566	-0.030599133	0.015546433
A0A3Q1MS21	VDAC1	A0A3Q1MS21	ENSBTAG00000013113	0.065687903	0.001492701	-0.064195202
A0AAA9SVE9	TMX3	A0AAA9SVE9	ENSBTAG00000007567	0.113150586	-0.053469271	-0.166619857
Q08DH4	KLC2	bta:518136; Q08DH4		0.008267616	-0.139162748	-0.147430364
A0AAA9S1F3	GPS1	A0AAA9S1F3	ENSBTAG00000019317	-0.159743625	-0.167372913	-0.007629288
A0AAA9TR62	AGRN	A0AAA9TR62	ENSBTAG00000013191	-0.123639777	-0.17513836	-0.051498583
A0A3Q1MJM3	TOR1AIP1	A0A3Q1MJM3	ENSBTAG00000035226	0.117405954	0.110159857	-0.007246098
F1MYG0	OAT	F1MYG0	ENSBTAG00000006928	-0.002363137	-0.134790692	-0.132427555
F1MK73	YAP1	F1MK73	ENSBTAG00000039307	0.135173464	0.048681938	-0.086491526
A0AAA9T8F7	ALDH1L2	A0AAA9T8F7	ENSBTAG00000006326	-0.346681207	-0.343524324	0.003156883
P79136	CAPZB	bta:338052; P79136; Q3T012	ENSBTAG00000004554	0.08094752	0.15215447	0.07120695
A0AAF6YU44	USP10	A0AAF6YU44	ENSBTAG00000009446	-0.168285016	-0.204130215	-0.035845199
F1N5F2	BCKDHA	F1N5F2	ENSBTAG00000016037	-0.128514741	-0.131504783	-0.002990043
A5PJW8	POLR2B	A5PJW8; bta:538184; F1MPP6	ENSBTAG00000019366	-0.199672345	0.008387785	0.20806013
A0A3Q1MEC9	TNPO2	A0A3Q1MEC9	ENSBTAG00000011811	-0.080634002	-0.16604293	-0.085408928
Q3T0E7	PPP1CA	bta:516175; Q3T0E7	ENSBTAG00000012146	-0.094708584	-0.122073483	-0.027364899
B0JYL8	CFL1	B0JYL8; bta:534553		0.116202372	0.05246742	-0.063734952
Q2HJJ0	KLC4	Q2HJJ0		0.051912089	0.10057172	0.048659632
A0AAA9T205	RNF20	A0AAA9T205	ENSBTAG00000009516	0.088767857	-0.075461539	-0.164229396
E1BDM8	DDX6	bta:513906; E1BDM8	ENSBTAG00000009067	0.025194082	0.07287888	0.047684798
Q29RL2	CHORDC1	bta:505144; Q29RL2	ENSBTAG00000013615	0.48725418	0.419145794	-0.068108387
P28801	GSTP1	bta:281806; P28801; Q3SZU6		0.05684586	0.032042469	-0.024803391
P21282	ATP6V1C1	bta:338089; P21282; Q0VCX0	ENSBTAG00000013513	0.019405399	0.03562391	0.016218511
A7YY57	ARHGAP29	A7YY57; bta:504657	ENSBTAG00000004190	-0.140706294	-0.232584587	-0.091878293
Q32PJ8	HDAC1	bta:404126; Q32PJ8	ENSBTAG00000012698	-0.123110542	-0.124493096	-0.001382554
A0A3Q1M4F5	ANXA7	A0A3Q1M4F5	ENSBTAG00000020218	-0.017642961	0.054742965	0.072385925
A4IFN7	UBE3A	A4IFN7; bta:533136	ENSBTAG00000002487	0.035991678	-0.058998418	-0.094990096
F1N036	DNAJC3	F1N036	ENSBTAG00000012065	0.372585358	0.221746197	-0.15083916
F1MR50	SLC4A1AP	F1MR50	ENSBTAG00000018680	0.151218807	0.023332504	-0.127886303
A0AAA9S381	COPS2	A0AAA9S381	ENSBTAG00000013425	0.029315567	0.025163924	-0.004151643
A0A3Q1M1N0	AMBRA1	A0A3Q1M1N0	ENSBTAG00000004282	0.637827741	0.389751058	-0.248076683
P10103	HMGB1	A5D9G8; bta:282691; P10103; Q3ZC39	ENSBTAG00000018103	0.103353658	0.17995072	0.076597061
F6R9I4	BIN1	F6R9I4	ENSBTAG00000019177	0.027631119	-0.020065623	-0.047696742
A0AAA9TL16	DOCK6	A0AAA9TL16	ENSBTAG00000009569	-0.089498151	-0.07636773	0.013130421
A0AAA9S1Z8	BLMH	A0AAA9S1Z8	ENSBTAG00000021370	0.15137706	0.166792112	0.015415052
A0A3Q1N8A3	CAPZB	A0A3Q1MG13; A0A3Q1N8A3	ENSBTAG00000004554	-0.21964181	-0.184800061	0.034841749
A0A452DIW6	MICALL1	A0A452DIW6	ENSBTAG00000037703	-0.249287075	-0.163703384	0.085583691
A0AAA9TZG3	PRMT5	A0AAA9TZG3	ENSBTAG00000010890	-0.122856748	-0.105330695	0.017526053
E1BN58	OTUD4	E1BN58	ENSBTAG00000019857	0.019365325	-0.011183738	-0.030549063
F1N4V2	CTR9	bta:534265; F1N4V2	ENSBTAG00000015566	0.077035384	0.064928781	-0.012106603
P26882	PPID	bta:281420; P26882; Q28077; Q2HJ45	ENSBTAG00000016680	0.434110233	0.405405737	-0.028704496
F6QQ96	NUB1	F6QQ96	ENSBTAG00000004540	-0.013095658	0.178269868	0.191365526
F1MZK0	NMT1	F1MZK0	ENSBTAG00000006051	0.166592533	0.13965199	-0.026940543
Q3ZBX7	ANKRD1	bta:510376; Q3ZBX7	ENSBTAG00000011734	-0.765820457	-0.629200396	0.136620061
A2VE21	COPG2	A2VE21; bta:536616	ENSBTAG00000017245	-0.126437464	-0.091116928	0.035320536
A7MAZ5	H1-3	A7MAZ5; bta:509275		-0.206251114	0.142100162	0.348351276
Q32L92	CNN3	bta:538975; Q32L92	ENSBTAG00000020345	0.089267338	0.02720637	-0.062060968
F1MH70	NCAPH	F1MH70	ENSBTAG00000012925	-0.098739118	-0.207595419	-0.108856302

Accession	Gene_Symbol	Gene_ID	Ensembl_Gene ID	log2(ratio(CM12H/CM0H))	log2(ratio(CM12R/CM0H))	log2(ratio(CM12H/CM12R))
F1MDU9	HEATR1	F1MDU9	ENSBTAG00000044100	-0.152153054	-0.233909654	-0.0817566
A0A3Q1LUY1	SCRIB	A0A3Q1LUY1	ENSBTAG00000008421	-0.099424991	-0.170893838	-0.071468848
A0A452DHX6	AP4M1	A0A452DHX6	ENSBTAG00000002881	-0.864116572	-0.846394802	0.01772177
F1MZK4	BZW1	F1MZK4	ENSBTAG00000006049	-0.117279816	-0.329628571	-0.212348755
A0AAA9RZ48	ZFR	A0AAA9RZ48	ENSBTAG00000008414	0.098603399	-0.127056736	-0.225660135
A0AAA9T2D6	SMURF2	A0AAA9T2D6	ENSBTAG00000019853	-0.472487771	-0.527629326	-0.055141554
A0AAA9TMV7	SMG1	A0AAA9TMV7; bta:525143	ENSBTAG00000020735	-0.103361219	-0.045523926	0.057837293
Q0VCU8	EIF3J	A6QPP4; bta:539052; Q0VCU8		0.130917117	-0.015138597	-0.146055714
A0AAA9SQ71	PXN	A0AAA9SQ71	ENSBTAG00000018108	-0.044394119	0.034201484	0.078595603
A0A3Q1LW88	DCAF1	A0A3Q1LW88; bta:785711	ENSBTAG00000006501	-0.171701064	-0.025788681	0.145912382
A7Z057	YWHAG	A7Z057; bta:286862; F2Z4H9	ENSBTAG00000004077	0.225451223	0.156119202	-0.069332021
Q2HJH2	RAB1B	bta:540685; Q2HJH2		-0.063997998	0.011599621	0.075597619
A0A3Q1M4Z7	BCAR1	A0A3Q1M4Z7	ENSBTAG00000012002	-0.17625064	-0.033569533	0.142681107
A0A3Q1LY70	NRDC	A0A3Q1LY70; bta:511254	ENSBTAG00000021174	-0.466471437	-0.420347388	0.04612405
A0AAA9TE99	EFL1	A0AAA9TE99; bta:527607	ENSBTAG00000003410	0.037216897	-0.012622084	-0.049838982
F1N152	HTRA1	bta:282326; F1N152; O97658		-0.481479643	-0.573013192	-0.091533549
A0A3Q1N0G2	ABHD16A	A0A3Q1N0G2	ENSBTAG00000000578	-0.082694684	-0.119435231	-0.036740547
A0AAA9TG77	FKBP15	A0AAA9TG77	ENSBTAG00000005116	0.171886518	0.171886518	0
A0AAA9SU45	ARFGEF2	A0AAA9SU45	ENSBTAG00000007120	-0.02620507	-0.002888279	0.023316791
A0AAA9T1Q6	TOP2B	A0AAA9T1Q6	ENSBTAG00000004593	-0.136036739	-0.075308286	0.060728454
A0A3Q1MC76	DLG1	A0A3Q1MC76	ENSBTAG00000020378	0.064609081	0.082549562	0.01794048
F1N4E5	TOR1AIP1	F1N4E5	ENSBTAG00000035226	0.229526464	0.096802433	-0.132724031
G3N251	CENPE	G3N251	ENSBTAG00000009035	0.033505803	0.088100778	0.054594975
A0A3Q1M7Q2	TNIK	A0A3Q1M7Q2	ENSBTAG00000011742	-0.072231902	0.023820492	0.096052394
E1BPR4	SEC24B	E1BPR4	ENSBTAG00000010945	0.010154758	0.050073768	0.03991901
Q3SWW9	PPP1CB	bta:538829; Q3SWW9	ENSBTAG00000012447	0.020631333	0.045293387	0.024662054
Q3ZC32	OPTN	bta:534150; Q3ZC32	ENSBTAG00000016401	0.001449217	-0.060707259	-0.062156476
Q32LG3	MDH2	Q32LG3		0.047305715	-0.007089425	-0.05439514
A0AAA9TFJ4	KIF3A	A0AAA9TFJ4	ENSBTAG00000025477	-0.163388397	-0.177357581	-0.013969184
A0A3Q1N147	RAB1A	A0A3Q1N147	ENSBTAG00000016720	0.12592244	0.179466096	0.053543656
F1MIX6	DOCK1	F1MIX6	ENSBTAG00000031890	-0.223407332	-0.275874752	-0.05246742
P81644	APOA2	bta:505394; P81644; Q2NKV9	ENSBTAG00000009212	-0.855183447	-1.01079328	-0.155609833
A0A3Q1MIM4	HNRNPH1	A0A3Q1MIM4; bta:541202	ENSBTAG00000009389	0.017956226	-0.078543122	-0.096499348
P32007	SLC25A6	A1L5C3; A6QQX7; bta:282480; P32007		0.015347964	-0.05825092	-0.073598884
A0AAA9TG74	PPP2R1B	A0AAA9TG74	ENSBTAG00000020277	-0.22928429	-0.463591518	-0.234307228
A0A3Q1LHD8	RPS6KA3	A0A3Q1LHD8	ENSBTAG00000017639	0.002837159	-0.009974228	-0.012811387
A5D7B4	DENND2B	A5D7B4; bta:537907; F6QME3	ENSBTAG00000005356	-0.129451251	-0.090241222	0.039210029
P11966	PDHB	A6QQW6; bta:613610; P11966; Q58CN6	ENSBTAG00000021724	0.059742084	0.035365154	-0.02437693
A0A3Q1LYB8	WNK1	A0A3Q1LYB8	ENSBTAG00000005221	0.034832572	0.022389509	-0.012443063
A0A452DJA8				-0.261727025	-0.124223501	0.137503524
A0A3Q1NMR2	SPATS2L	A0A3Q1NMR2	ENSBTAG00000016092	-0.139045689	-0.263034406	-0.123988717
A0AAF6Z5Y5	FBLIM1	A0AAF6Z5Y5	ENSBTAG00000018725	-0.0135944	-0.048151622	-0.034557222
Q08E27	STRBP	bta:516282; Q08E27		0.099108145	-0.025878632	-0.124986777
E1B844	TIMM44	E1B844	ENSBTAG00000015567	-0.059344461	-0.121235135	-0.061890674
F1MVK2	SCYL1	F1MVK2	ENSBTAG00000006740	0.041317878	0.086587685	0.045269807
Q2KI14	NAA10	bta:613636; Q2KI14		0.008453315	-0.004245305	-0.01269862
A0A3Q1LX10	RCC1	A0A3Q1LX10	ENSBTAG00000003788	0.013885526	-0.051124465	-0.065009991
F1MH45	CDC42BPA	F1MH45	ENSBTAG00000022777	-0.034140141	-0.129831386	-0.095691245
O77784	IDH3B	bta:613338; O77784; O77785; Q3MI01	ENSBTAG00000018813	-0.005646563	0.026523443	0.032170006
F1MSR6	TSR1	F1MSR6	ENSBTAG00000004225	-0.06997953	-0.238650247	-0.168670718
F1MBS5	KDM1A	F1MBS5	ENSBTAG00000009500	-0.10246562	-0.123861913	-0.021396293
A0A3Q1MWG7	ME2	A0A3Q1MWG7	ENSBTAG00000016269	0.035771131	0.110360447	0.074589316
A7MB62	ACTR2	A7MB62; bta:538486	ENSBTAG00000009761	0.039811178	0.07287888	0.033067702
A0AAA9T768	RAB3GAP2	A0AAA9T768	ENSBTAG00000012585	-0.03093186	0.072469638	0.103401498
Q2KJ39	RCN3	bta:522073; Q2KJ39		-0.085348788	-0.236664065	-0.151315277
Q5EAC7	CIAPIN1	bta:535119; Q0VCL8; Q5EAC7		-0.148644081	-0.076392009	0.072252072
A6H7B5	COPS3	A6H7B5; bta:507932	ENSBTAG00000018973	-0.010083781	-0.051138849	-0.041055068
E1BCH9	VCPIP1	E1BCH9	ENSBTAG00000006695	-0.036954722	0.033063208	0.07001793
Q0P5M8	PMPCA	bta:767847; Q0P5M8	ENSBTAG00000001353	-0.214124805	-0.219907158	-0.005782353
A0AAA9SY31	CORO1C	A0AAA9SY31	ENSBTAG00000007993	-0.16314474	0.012589042	0.175733782
A0AAA9T271	TMEM214	A0AAA9T271	ENSBTAG00000017423	0.063174198	-0.073277607	-0.136451805
A0AAA9RZQ0	TTF2	A0AAA9RZQ0	ENSBTAG00000015392	-0.09698999	-0.167944637	-0.070954647
A0AAA9RUL8	MFGE8	A0AAA9RUL8	ENSBTAG00000003300	-0.042644337	0.080359617	0.123003954
Q95115	STAT5A	bta:282375; O46628; O97606; Q95115; Q9N0U8; Q9TUM1	ENSBTAG00000009496	-0.163135836	0.019526623	0.182662459
Q17QP9	PSMD14	bta:535683; Q17QP9	ENSBTAG00000021691	0.049388027	0.015704901	-0.033683126
P61763	STXBP1	bta:282378; P61763; Q28208; Q62759; Q64320; Q96TG8		0.080891452	0.006265786	-0.074625666
E1BPW1	OSBP	E1BPW1	ENSBTAG00000020332	-0.058255572	-0.051024003	0.007231569
A0AAF6YIQ2	CIAPIN1	A0AAF6YIQ2	ENSBTAG00000001854	0.1054163	0.127527001	0.022110702
A0AAA9TQX8	RPS3	A0AAA9TQX8	ENSBTAG00000005620	-0.351472371	-0.238731158	0.112741212
Q2LGB8	CASP8	bta:507481; Q2LGB8	ENSBTAG00000015718	-0.033858804	0.254955541	0.288814345
A0AAA9TCE5	LEMD3	A0AAA9TCE5	ENSBTAG00000039435	-0.012761022	-0.029953222	-0.0171922
F1MTR3	SIN3A	F1MTR3	ENSBTAG00000009985	-0.069751108	0.043319038	0.113070146
G3N3B7	PTK2	G3N3B7	ENSBTAG00000009578	0.047658618	0.131380444	0.083721826
A4IFE0	CCDC93	A4IFE0; bta:616839; F1MDJ4	ENSBTAG00000010221	0.142674967	0.091515111	-0.051159856
Q5E966	EIF3I	bta:521747; Q3SZ38; Q5E966	ENSBTAG00000014388	-0.099631181	-0.134431543	-0.034800362
A0A3Q1LPY4	PCBP2	A0A3Q1LPY4	ENSBTAG00000020757	-0.004175676	-0.016775713	-0.012600037
A0AAA9SIU0	FRYL	A0AAA9SIU0	ENSBTAG00000000137	-0.042644337	-0.083616118	-0.040971781
F1N7C1	HERC6	F1N7C1	ENSBTAG00000020536	-0.42619036	-0.27163043	0.154559931
E1BPA1	TBC1D4	bta:534649; E1BPA1	ENSBTAG00000005760	0.011518584	0.044131262	0.032612677
A0JN78	HEATR3	A0JN78; bta:539353; F1MLE2	ENSBTAG00000016640	-0.030222148	-0.12799293	-0.097770782
Q1RMX7	NANS	bta:540602; Q1RMX7	ENSBTAG00000019023	0.080865003	0.141073533	0.06020853
A0AAA9TIF9	GLRX3	A0AAA9TIF9	ENSBTAG00000005027	0.012226302	0.110747066	0.098520763
Q3SWY2	ILK	bta:540207; Q3SWY2	ENSBTAG00000000696	-0.191481145	-0.154722595	0.03675855
Q58DT4	PYCR1	bta:539606; Q58DT4	ENSBTAG00000000042	-0.159822598	-0.147677168	0.01214543
Q2NL05	KIF2A	bta:768014; Q2NL05		0.022068423	0.017956226	-0.004112198
Q5E9F1	BCAP31	bta:533949; Q5E9F1	ENSBTAG00000020547	0.272336853	0.236440196	-0.035896657

Accession	Gene_Symbol	Gene_ID	Ensembl_Gene ID	log2(ratio(CM12H/CM0H))	log2(ratio(CM12R/CM0H))	log2(ratio(CM12H/CM12R))
E1BBJ7	INPPL1	bta:783833; E1BBJ7	ENSBTAG00000019167	-0.15274199	-0.138035491	0.014706499
P62935	PPIA	P04374; P10110; P62935; Q29580; Q56JX4	ENSBTAG00000012003	-0.022720077	-0.021289541	0.001430536
A0A493UA87	AKR1B1	A0A493UA87; bta:317748	ENSBTAG00000009902	0.176549392	0.191577601	0.015028209
Q3SX26	TRIP6	bta:615869; Q3SX26	ENSBTAG00000024772	-0.10523472	0.033947332	0.139182052
F1N155	CLPX	F1N155	ENSBTAG00000008386	-0.303727916	-0.363375379	-0.059647463
A0AAA9T4H8	SUCLG2	A0AAA9T4H8	ENSBTAG00000009541	0.158503822	0.100652167	-0.057851656
G3X6R3	STAG2	G3X6R3	ENSBTAG00000009121	-0.046448923	-0.033233303	0.01321562
A5PJA6	STOML2	A5PJA6	ENSBTAG00000011388	-0.028842322	-0.113616075	-0.084773754
D3JUI8	RBMX	bta:509591; D3JUI8	ENSBTAG00000013308	-0.131951131	-0.156543119	-0.024591988
A0AAA9S0E1	NPM1	A0AAA9S0E1	ENSBTAG00000015316	0.030042331	0.030042331	0
Q3MHZ4	TGFB1I1	bta:515834; Q3MHZ4		-0.243499801	-0.3347438	-0.091243998
Q3SYS9	KIFBP	bta:527433; Q3SYS9	ENSBTAG00000014561	0.111958863	0.084425679	-0.027533184
Q3T005	PDLIM4	bta:515410; Q3T005	ENSBTAG00000015426	0.170846725	0.308350249	0.137503524
Q0VD16	MAP2K1	bta:533199; F2Z4G4; Q0VD16	ENSBTAG00000033983	-0.007557351	-0.024324202	-0.016766851
Q5XQN5	KRT5	bta:281268; Q5XQN5		-0.446945242	-0.304023632	0.142921611
A0A3Q1NBU3	AGPS	A0A3Q1NBU3	ENSBTAG00000009897	0.026808797	-0.031677212	-0.058486008
P56658	ADA	bta:280712; P56658; Q9MYY1		-0.003126101	-0.015698686	-0.012572585
F1MF74	MICAL2	A0A3Q1LSU4; F1MF74		-0.081838292	-0.226375935	-0.144537643
A5D7A5	GOLGA5	A5D7A5; bta:510412		0.070448602	0.013627963	-0.056820638
A0A3Q1MCE5	AACS	A0A3Q1MCE5; bta:505842	ENSBTAG00000005678	-0.092244911	0	0.092244911
Q1RMT6	DBN1	bta:505406; F1N3U4; Q1RMT6		-0.196742727	-0.0694851	0.127257627
A0A3Q1M8R6	PML	A0A3Q1M8R6	ENSBTAG00000015779	0.023458973	0.008841877	-0.014617096
A1L555	PSAP	A1L555		-0.116193018	-0.043589269	0.072603749
A0AAA9T9H6	XRCC5	A0AAA9T9H6	ENSBTAG00000010426	-0.129598326	0.0114842	0.141082526
A4FV36	SPATA20	A4FV36; bta:511363	ENSBTAG00000004155	-0.168827716	-0.188707687	-0.019879971
F1MMN6	ITGA1	F1MMN6	ENSBTAG00000016525	-0.054585138	-0.084564295	-0.029979157
E1BKQ2	RAB11FIP1	E1BKQ2	ENSBTAG00000011684	-0.104000193	-0.174060776	-0.070060583
A0AAA9TAD8	ADK	A0AAA9TAD8	ENSBTAG00000011072	0.048543852	0.040565795	-0.007978057
A0AAA9TTF2	DYNC1LI2	A0AAA9TTF2	ENSBTAG00000000458	0.064675677	0.028233681	-0.036441996
A4FUZ6	HSDL2	A4FUZ6; A6QPZ9; bta:404131	ENSBTAG00000031295	-0.073782195	-0.078156211	-0.004374016
E1BB48	GALNT2	E1BB48	ENSBTAG00000004870	0.028289044	0.039703834	0.01141479
A0AAA9SDW9	BTAF1	A0AAA9SDW9	ENSBTAG00000016104	-0.303906687	-0.141000316	0.162906371
A0A3Q1LN63	SERPINB1	A0A3Q1LN63	ENSBTAG00000011975	0.047466739	0.137957273	0.090490534
Q17QI3	ACAT2	Q17QI3	ENSBTAG00000002827	0.080389387	0.173882028	0.093492641
A0A3Q1N1J5	PLBD2	A0A3Q1N1J5	ENSBTAG00000019040	-0.11533061	-0.064770255	0.050560354
Q3ZBW4	PCNA	bta:515499; Q3ZBW4	ENSBTAG00000006065	0.148920881	0.041903953	-0.107016928
F1MBM9	ARHGEF7	F1MBM9	ENSBTAG00000020726	0.139551352	0.085946609	-0.053604744
A0A3Q1LN88	UACA	A0A3Q1LN88	ENSBTAG00000013982	-0.156581005	-0.064597867	0.091983139
A6QNT8	SEC24A	A6QNT8; bta:505089	ENSBTAG00000018322	-0.041013382	-0.017434593	0.023578789
Q2KJE7	PSME1	bta:510041; Q2KJE7	ENSBTAG00000021395	-0.022825805	-0.107837742	-0.085011937
A0AAA9S0H3	EML4	A0AAA9S0H3	ENSBTAG00000010935	0.036606471	0.188609565	0.152003093
A0A3Q1MH45	GSPT2	A0A3Q1MH45; bta:538724	ENSBTAG00000013311	0.151537633	0.093109404	-0.058428228
A0A3Q1LUV2	PGM3	A0A3Q1LUV2	ENSBTAG00000000679	0.072235817	0.063823577	-0.00841224
O02811	PI4KA	bta:282309; F1MLV0; O02811		-0.226396573	-0.128733314	0.097663259
A0A3Q1LPF0	APOE	A0A3Q1LPF0	ENSBTAG00000010123	-1.044349371	-1.177282502	-0.13293313
F1MGC0	SUCLA2	F1MGC0	ENSBTAG00000006759	-0.076392009	-0.164104186	-0.087712176
A0AAA9RZ74	SF1	A0AAA9RZ74	ENSBTAG00000001035	0.047549352	0.1960401	0.148490748
A0AAA9RVE8	RBM15	A0AAA9RVE8	ENSBTAG00000060532	-0.00434329	0.011518584	0.015861874
A0A3Q1MB23				-0.052184733	-0.094636874	-0.042452142
Q5E970	DDX39A	bta:614215; Q5E970	ENSBTAG00000021820	-0.039156966	-0.005955406	0.03320156
O46563	ATP6V1H	A0A3Q1M3I3; bta:282657; F1MZL6; O46562; O46563; Q3ZBD5	ENSBTAG00000003450	0.229753126	0.098059015	-0.131694111
E1BM12	SRM	bta:505916; E1BM12	ENSBTAG00000005304	-0.180020048	-0.268488836	-0.088468788
A0AAA9U092	SEPTIN6	A0AAA9U092	ENSBTAG00000004291	0.029968037	0.033142285	0.003174248
E1BJF6	SYMPK	E1BJF6	ENSBTAG00000012101	-0.036186538	-0.118568866	-0.082382328
B0JYK4	PRKAR2B	B0JYK4		0.111465271	0.124662573	0.013197302
F6PWU2	BTF3	F6PWU2	ENSBTAG00000010012	0.047643523	-0.212606978	-0.260250501
A0A3Q1LHG7	MARK2	A0A3Q1LHG7	ENSBTAG00000007825	-0.104371141	-0.049099854	0.055271287
Q3ZCJ2	AKR1A1	bta:618607; Q3ZCJ2	ENSBTAG00000000497	0.035333073	0.107660205	0.072327132
A0AAA9TZT4	RRM2	A0AAA9TZT4	ENSBTAG00000008216	-0.386249076	-0.586641029	-0.200391953
P23004	UQCRC2	P23004; Q3ZCG7; Q5E9C7	ENSBTAG00000021651	-0.092494688	-0.119435231	-0.026940543
E1BB46	SH3BP4	bta:520462; E1BB46	ENSBTAG00000013869	-0.057915259	0.025225976	0.083141235
A0AAA9SN47	SF1	A0AAA9SN47	ENSBTAG00000001035	0.190296049	0.254664868	0.064368819
A0A3Q1N3E6	GRIPAP1	A0A3Q1N3E6	ENSBTAG00000007788	-0.100870884	-0.020159085	0.080711799
F1MGJ5	SEL1L	F1MGJ5	ENSBTAG00000008083	-0.031891722	0.028515371	0.060407094
A0AAA9S5S3	MTMR6	A0AAA9S5S3	ENSBTAG00000003116	0.086414752	0.023554728	-0.062860024
Q2KI95	FHL2	bta:510008; Q2KI95	ENSBTAG00000001086	-0.063635234	-0.015647605	0.047987629
A0A3Q1N4H1	FANCI	A0A3Q1N4H1	ENSBTAG00000009097	-0.27472673	-0.116735721	0.157991009
Q3ZC13	AP2M1	bta:517446; Q3ZC13	ENSBTAG00000020106	0.113656782	0.127046159	0.013389377
A0AAA9SH40	TRRAP	A0AAA9SH40	ENSBTAG00000007113	-0.121405947	-0.065357201	0.056048746
A0AAA9SQZ6	GPATCH8	A0AAA9SQZ6	ENSBTAG00000038316	0.086060729	-0.01463327	-0.100694
P20000	ALDH2	bta:508629; P20000; Q1LZC6	ENSBTAG00000008743	0.025011888	0.02726434	0.002252452
E1BJ19	TAOK1	bta:536532; E1BJ19	ENSBTAG00000000827	0.130279197	0.055973638	-0.074305559
A0AAA9TYT8	ALDH4A1	A0AAA9TYT8	ENSBTAG00000030335	0.018339591	-0.015463117	-0.033802708
A0AAA9TMW1	EIF3M	A0AAA9TMW1	ENSBTAG00000005338	-0.102419111	-0.11504365	-0.012624539
A0AAA9TJ26	ACAD10	A0AAA9TJ26	ENSBTAG00000001164	-0.064747181	-0.077416374	-0.012669193
F1MUW6	TMF1	F1MUW6	ENSBTAG00000015554	0.163498732	0.008850918	-0.154647815
A0A3Q1LMM5	SRGAP2	A0A3Q1LMM5; bta:519843	ENSBTAG00000001483	0.005949266	0.008914725	0.002965458
A0A3Q1MNY8	PRP4K	A0A3Q1MNY8	ENSBTAG00000002086	0.167344558	-0.00546994	-0.172814499
F1MKF8	SQOR	F1MKF8	ENSBTAG00000010365	0.118386204	0.227790164	0.109403961
A0AAA9TLS4	SPTBN2	A0AAA9TLS4	ENSBTAG00000005929	-0.132247798	-0.161394144	-0.029146346
A0AAF7AS54	VRK1	A0AAF7AS54	ENSBTAG00000014230	-0.091792476	-0.041206751	0.050585725
G3MWV5	H1-4	G3MWV5	ENSBTAG00000047206	-0.175661371	0.001271657	0.176933028
F6Q807	HNRNPUL1	F6Q807	ENSBTAG00000003168	-0.1110899	0.056020086	0.167109986
F1MW06	NOP56	F1MW06	ENSBTAG00000018812	-0.227951947	-0.062365881	0.165586066
A0AAA9TGS6	BZW2	A0AAA9TGS6	ENSBTAG00000014262	-0.022110702	-0.204447939	-0.182337238
E1BL45	EHBP1L1	E1BL45	ENSBTAG00000009554	-0.060187006	-0.055343034	0.004843972
A6QP83	FUBP3	A6QP83; bta:541080	ENSBTAG00000013072	0.135420209	0.146496617	0.011076408
A0A3Q1LHD2	PBRM1	A0A3Q1LHD2; bta:506557	ENSBTAG00000014786	-0.14741347	-0.072526259	0.074887212
F1N6E9	RTF1	bta:518260; F1N6E9	ENSBTAG00000001671	0.222181886	0.06342538	-0.158756506

Accession	Gene_Symbol	Gene_ID	Ensembl_Gene ID	log2(ratio(CM12H/CM0H))	log2(ratio(CM12R/CM0H))	log2(ratio(CM12H/CM12R))
A0AAA9SME9	POGLUT2	A0AAA9SME9	ENSBTAG00000008880	-0.30461821	-0.165911939	0.138706271
P48452-2	PPP3CA	P48452-2		0.059562434	0.044900773	-0.014661661
A0A3Q1LQ03	SON	A0A3Q1LQ03	ENSBTAG00000008482	0.067498761	-0.094708584	-0.162207345
A0AAA9SLN8	KIF14	A0AAA9SLN8	ENSBTAG00000021874	0.037140735	0.020117588	-0.017023148
A0A3Q1N6B7	LRCH3	A0A3Q1N6B7	ENSBTAG00000032839	-0.089498151	-0.11276793	-0.023269779
A0A3Q1M3I7	PPP1R9B	A0A3Q1M3I7	ENSBTAG00000049864	0.081769556	0.065484472	-0.016285084
A0AAA9TKA2	CLIC1	A0AAA9TKA2	ENSBTAG00000013533	0.032924638	0.0026618	-0.030262839
Q3SWX9	RAD21	bta:540966; Q3SWX9	ENSBTAG00000007303	0.099144646	0.007312205	-0.091832441
A0AAA9SLV9	HSPA14	A0AAA9SLV9	ENSBTAG00000016451	-0.00269915	-0.064804652	-0.062105502
Q2TBG8	UCHL3	bta:520170; Q2TBG8		0.204207433	0.511046191	0.306838759
A0A3Q1LWN6	MYH11	A0A3Q1LWN6	ENSBTAG00000015988	-0.421485309	-0.367576146	0.053909164
A0A3Q1NL10	SORBS2	A0A3Q1NL10	ENSBTAG00000016486	-0.010766431	0.088408974	0.099175405
A0AAF6YJJ5	NDUFS2	A0AAF6YJJ5	ENSBTAG00000002203	-0.150289679	-0.094310007	0.055979672
A0A3Q1LZH0	PKN2	A0A3Q1LZH0	ENSBTAG00000021900	-0.105717721	-0.096605932	0.009111788
A0AAA9RYK0	TWF2	A0AAA9RYK0	ENSBTAG00000054131	-0.090990768	-0.017452169	0.073538599
F1MFS4	SRGAP1	bta:539452; F1MFS4	ENSBTAG00000011892	-0.084972252	-0.090819048	-0.005846796
P02253	H1-2	A3KN02; bta:513971; P02253	ENSBTAG00000011677	-0.230452191	0.316290987	0.546743178
P34955	SERPINA1	bta:280699; P34955; Q3SZS3	ENSBTAG00000018843	-0.350065602	0.393288909	0.743354512
F1MUH9	CTTNBP2NL	bta:538981; F1MUH9	ENSBTAG00000021602	-0.023347312	0.020291404	0.043638716
F1MWP1	BUB1B	F1MWP1	ENSBTAG00000007237	-0.113776356	0.18203025	0.295806606
F1MC11	KRT14	F1MC11	ENSBTAG00000007583	-0.251275237	-0.166009951	0.085265286
A0AAF7AML4	RPAP1	A0AAF7AML4	ENSBTAG00000001693	-0.035477895	0.09739243	0.132870325
A0A3Q1LLS1	CTPS2	A0A3Q1LLS1	ENSBTAG00000017017	0.122644914	0.121055167	-0.001589747
A0A3Q1MIY3	CAPNS1	A0A3Q1MIY3	ENSBTAG00000014872	0.002971567	0.056869522	0.053897956
F1MK70	SPATS2	bta:617860; F1MK70	ENSBTAG00000004660	0.112657801	0.011669993	-0.100987808
P67774	PPP2CA	A6QLI7; bta:282320; P05323; P13197; P67774	ENSBTAG00000000469	0.063215295	0.205548911	0.142333616
A0AAA9T615	PCMT1	A0AAA9T615	ENSBTAG00000010204	0.109014586	0.148026899	0.039012313
Q2KIT4	DNAJB4	bta:541274; Q2KIT4	ENSBTAG00000021752	0.46738209	0.274737012	-0.192645078
A0A3Q1M986	GALNT2	A0A3Q1M986	ENSBTAG00000004870	0.179954356	0.115982027	-0.063972329
A4FV40	EIF2B3	A4FV40; bta:534063; E1BB97	ENSBTAG00000019010	-0.116292135	-0.122360262	-0.006068127
Q27977	ITGA5	F1MK44; Q27977	ENSBTAG00000013745	-0.145658631	-0.101904101	0.04375453
A0AAA9T986	STIM1	A0AAA9T986	ENSBTAG00000013109	0.066783629	0.18407549	0.117291861
F1MQI1	TLN2	F1MQI1	ENSBTAG00000003667	0.144663315	0.046610942	-0.098052373
A0AAA9RRS3	TMOD3	A0AAA9RRS3	ENSBTAG00000048622	-0.066533751	-0.13034172	-0.063807969
A0A3Q1LYB3	COPS6	A0A3Q1LYB3	ENSBTAG00000002871	-0.033358597	0.0109503	0.044308897
A5D9B4	HNRNPH2	A5D9B4	ENSBTAG00000007499	0.012551317	-0.018324658	-0.030875975
A0AAA9SHR8	CAPG	A0AAA9SHR8	ENSBTAG00000005432	0.021143931	-0.004266228	-0.025410159
A0AAF6DLX8	KIF2C	A0AAF6DLX8	ENSBTAG00000015280	0.069540933	0.087612506	0.018071573
G5E5C8	TALDO1	bta:513453; G5E5C8	ENSBTAG00000010336	0.051994638	0.045596866	-0.006397771
F1MV75	WDR48	F1MV75	ENSBTAG00000003745	0.226536115	0.351952055	0.12541594
P00514	PRKAR1A	A5D9F4; bta:615074; P00514; Q17QP8	ENSBTAG00000008621	0.264266952	-0.196243087	-0.460510039
A0A3Q1LUG9	TXNDC5	A0A3Q1LUG9; bta:615027	ENSBTAG00000019237	0.061400545	-0.045751808	-0.107152353
Q3T0C7	STMN1	bta:616317; Q3T0C7	ENSBTAG00000013761	0.079159538	0.083317163	0.004157625
Q0P594	PPP2CB	bta:524361; Q0P594	ENSBTAG00000009245	0.067227942	0.106110602	0.038882661
Q29RK1	CS	bta:280682; Q29RK1	ENSBTAG00000004371	0.063699379	0.001415101	-0.062284278
Q3SWY7	SF3A3	bta:523250; Q3SWY7	ENSBTAG00000007299	0.091608758	0.001410949	-0.090197809
G3N2U8	CTNNA2	G3N2U8	ENSBTAG00000031669	0.013175389	-0.118158368	-0.131333757
A2T1U6	AP-B	A2T1U6		-0.016761791	-0.090889141	-0.07412735
F1MI41	SYNRG	F1MI41	ENSBTAG00000010292	0.214178239	0.192645078	-0.021533162
F1MEX9	ACSL3	bta:100138312; F1MEX9	ENSBTAG00000017258	0.246591643	0.337739531	0.091147888
Q0V8S0	HGS	bta:511582; Q0V8S0; Q2NKZ6		0.037637657	0.149299481	0.111661823
Q08DQ2	GFPT2	bta:530101; Q08DQ2		-0.046755323	0.112297773	0.159053096
A4FV97	RSL1D1	A4FV97	ENSBTAG00000014588	-0.09934781	0.002627861	0.101975671
Q1LZB6	CAPRIN1	bta:535571; Q1LZB6	ENSBTAG00000016744	0.001282966	-0.215563083	-0.216846049
A0AAA9TU20	NUP50	A0AAA9TU20	ENSBTAG00000017652	0.016000479	-0.085620426	-0.101620904
A0A3Q1LP50	LARP7	A0A3Q1LP50	ENSBTAG00000009632	-0.016591553	0.137230828	0.153822381
F1N746	RABGAP1	bta:519260; F1N746	ENSBTAG00000039172	0.059011215	0.014617096	-0.044394119
A0AAF7AMR3	NT5C2	A0AAF7AMR3	ENSBTAG00000012858	-0.240694867	-0.398288345	-0.157593478
F1N1R4	DIS3	F1N1R4	ENSBTAG00000019889	-0.280829989	-0.140538648	0.140291341
A0AAA9TZA6	FTO	A0AAA9TZA6	ENSBTAG00000012501	0.01165036	-0.059706246	-0.071356606
F1MLG3	TRAPPC8	F1MLG3	ENSBTAG00000007696	-0.005217709	-0.034257382	-0.029039673
Q5E959	STRAP	bta:510201; Q5E959	ENSBTAG00000014175	0.085202323	0.087785484	0.002583161
A0AAA9S5I1	ACTR10	A0AAA9S5I1	ENSBTAG00000014392	-0.183424191	-0.24962828	-0.066204089
Q17QQ2	TPMT	bta:511644; Q17QQ2	ENSBTAG00000019300	0.188003696	0.303898058	0.115894362
A0AAA9RXW5	PCBP2	A0AAA9RXW5	ENSBTAG00000020757	-0.077305726	0.109998418	0.187304144
Q0VCY1	VAPA	bta:516024; Q0VCY1	ENSBTAG00000017279	0.102985223	0.092716888	-0.010268335
P56652	ITIH3	A7MB92; bta:508355; P56652; Q0V8M9	ENSBTAG00000007846	-0.193941883	0.27583168	0.469773563
A7MBG0	ADSS2	A7MBG0; bta:522529	ENSBTAG00000013885	-0.00992684	-0.021595148	-0.011668308
Q1LZD9	PSPC1	bta:506696; Q1LZD9		-0.14281632	-0.063944929	0.078871391
A0A3Q1LMX5	ABCC1	A0A3Q1LMX5	ENSBTAG00000021090	-0.047696742	0.007312205	0.055008947
E1BMU6	AKAP1	bta:532072; E1BMU6	ENSBTAG00000025320	-0.208554658	-0.08899193	0.119562728
A0A3Q1LN12	SEPTIN8	A0A3Q1LN12	ENSBTAG00000008609	0.108673159	0.173796177	0.065123018
G3X6E6	TANC1	bta:507983; G3X6E6	ENSBTAG00000004177	0.023323522	0.024975148	0.001651626
A0A3S5ZPF7	PSAT1	A0A3S5ZPF7	ENSBTAG00000013960	-0.09069831	0.003443188	0.094141498
Q10741	ADAM10	A8E663; bta:282132; Q10741	ENSBTAG00000005481	0.077250521	-0.148656385	-0.225906906
Q3SX08	EMD	Q3SX08	ENSBTAG00000022314	-0.026386766	-0.054861935	-0.028475169
A0AAF6YQ83	DRG2	A0AAF6YQ83	ENSBTAG00000006517	-0.196857329	-0.037281016	0.159576313
A0A3Q1MKT3	GPSM1	A0A3Q1MKT3	ENSBTAG00000018799	-0.103304498	-0.165836281	-0.062531783
A0A3Q1M3Z5	CTSD	A0A3Q1M3Z5	ENSBTAG00000007622	0.099087214	0.171045541	0.071958328
A4IFH7	MAP2K3	A4IFH7; bta:516039; F6S1D0	ENSBTAG00000010576	-0.110456648	-0.175503945	-0.065047297
E1BPP1	TTK	E1BPP1	ENSBTAG00000005456	0.131510578	0.110407885	-0.021102693
A0A3Q1MLW0	PPP1CC	A0A3Q1MLW0	ENSBTAG00000011198	-0.332377599	-0.136542759	0.19583484
F1MKX4	PSME4	F1MKX4		-0.200174382	-0.219086386	-0.018912004
A6QR55	USP4	A6QR55; bta:508042	ENSBTAG00000011899	-0.192292814	-0.073351742	0.118941073
A0A3Q1MMU8	ABR	A0A3Q1MMU8	ENSBTAG00000008424	-0.05079319	-0.081820086	-0.031026896
Q2KJ77	HSPBP1	bta:512757; Q2KJ77	ENSBTAG00000003644	0.049147649	0.068347296	0.019199647
A0A5H1ZRH6	CDH2	A0A5H1ZRH6	ENSBTAG00000021190	0.288091863	0.012904335	-0.275187528

Accession	Gene_Symbol	Gene_ID	Ensembl_Gene ID	log2(ratio(CM12H/CM0H))	log2(ratio(CM12R/CM0H))	log2(ratio(CM12H/CM12R))
Q58DM8	ECHS1	bta:281748; Q2TBV2; Q4PS76; Q58DM8		0.016608918	-0.046293652	-0.06290257
A0AAA9TS26	CNP	A0AAA9TS26	ENSBTAG00000025762	0.131166996	0.199538894	0.068371898
A0AAA9SUZ5	CKB	A0AAA9SUZ5	ENSBTAG00000035998	0.075004428	-0.027030206	-0.102034634
E1B7G3	STUB1	E1B7G3	ENSBTAG00000019136	-0.109912624	-0.090354777	0.019557846
A0A452DI66	F2	A0A452DI66	ENSBTAG00000007148	-0.481774433	-0.666779448	-0.185005016
Q3T165	PHB1	bta:530409; Q3T165	ENSBTAG00000017120	-0.015281458	-0.088326782	-0.073045325
F1N1A3	UBQLN1	F1N1A3	ENSBTAG00000014827	-0.023995481	0.019206669	0.04320215
E1BNG7	TUBGCP3	E1BNG7	ENSBTAG00000044184	-0.152882517	-0.003961636	0.148920881
Q2KII9	DYNC1LI1	bta:510330; F1N4N4; Q2KII9	ENSBTAG00000014700	-0.024416527	0.026808797	0.051225323
Q5E9J1	HNRNPF	bta:506917; Q5E9J1	ENSBTAG00000008853	0.147557188	0.111031312	-0.036525876
F1MUD4	USP19	F1MUD4	ENSBTAG00000018921	0.208131728	0.225767148	0.01763542
E1BA93	SYNPO	E1BA93	ENSBTAG00000013744	0.013617569	0.10408916	0.090471591
E1BE82	PUM1	E1BE82	ENSBTAG00000018979	-0.007455805	-0.008951601	-0.001495796
Q0VC01	HDAC2	bta:407223; Q0VC01		-0.081418794	-0.204981738	-0.123562944
A0A3Q1LYL5	PCCA	A0A3Q1LYL5	ENSBTAG00000010672	0.008933125	0.011898583	0.002965458
A0AAA9TES1	RAB5C	A0AAA9TES1	ENSBTAG00000006982	-0.019899557	0.14178145	0.161681007
A0A3Q1LPE3	PCCB	A0A3Q1LPE3	ENSBTAG00000015221	-0.187945409	-0.076491045	0.111454364
Q9BGI3	PRDX2	bta:286793; Q3T0L0; Q9BGI3		0.033166864	0.095645575	0.062478712
A0AAF7AHP7	EIF5A	A0AAF7AHP7	ENSBTAG00000002018	-0.022498624	-0.078855421	-0.056356796
A0AAA9RWC4	VPS26A	A0AAA9RWC4	ENSBTAG00000016882	0.018859027	0.03129613	0.012437103
E1BK29	COG1	E1BK29	ENSBTAG00000006087	-0.114711659	-0.093547049	0.02116461
F6R2J7	SETD3	F6R2J7	ENSBTAG00000018310	-0.182465298	-0.259180264	-0.076714966
Q1JQE0	SNW1	bta:326578; Q1JQE0	ENSBTAG00000008136	0.153679671	-0.05267454	-0.206354211
A0A3Q1MGW7	STRN	A0A3Q1MGW7	ENSBTAG00000018590	0	0.070098198	0.070098198
A5PJR5	CSTF3	A5PJR5; bta:508491; E1BGY7		-0.027451685	0.114663547	0.142115233
E1BC55	KIAA1671	E1BC55	ENSBTAG00000044022	-0.130038816	-0.193138616	-0.0630998
F1N206	DLD	bta:533910; F1N206	ENSBTAG00000001908	0.027250034	0.15627594	0.129025906
Q3MHG1	SPTLC1	bta:614165; Q3MHG1	ENSBTAG00000002220	-0.084064265	0.168243734	0.252307999
A0AAF6YS25	GNPDA1	A0AAF6YS25	ENSBTAG00000007865	-0.025192091	0.19216474	0.217356831
Q08DE9	CUL2	bta:535219; F1MWA4; Q08DE9		0.033341075	0.173158028	0.139816953
P10895	PLCD1	A2VDM2; bta:281986; P10895	ENSBTAG00000037726	0.023189627	-0.020177882	-0.043367509
A0A3Q1M3Y2	CHD3	A0A3Q1M3Y2	ENSBTAG00000014377	-0.267824765	-0.162967451	0.104857314
F1MFC6	FARP1	F1MFC6	ENSBTAG00000000146	-0.15785582	-0.049916999	0.107938821
F1MJ80	NAMPT	F1MJ80	ENSBTAG00000015509	-0.037544369	0.017104656	0.054649025
F1N565	ZC3H14	F1N565	ENSBTAG00000030453	0.051555188	-0.078711976	-0.130267164
E1BPP6	MCCC2	E1BPP6	ENSBTAG00000044160	-0.087947699	-0.020833503	0.067114196
F1N7X3	NAP1L4	F1N7X3	ENSBTAG00000022160	-0.005652094	0.066152335	0.071804429
Q05B79	DHX36	bta:509583; Q05B79		-0.035170778	-0.01366055	0.021510229
A0AAA9T043	THADA	A0AAA9T043; bta:514187	ENSBTAG00000003555	-0.251352601	-0.284303023	-0.032950422
A0AAA9SUD1	ACADS	A0AAA9SUD1	ENSBTAG00000007484	-0.135614133	0.095652239	0.231266372
F6RFP6	BSG	F6RFP6	ENSBTAG00000016648	0.270826662	0.107779093	-0.163047569
E1BME9	NDUFV3	E1BME9	ENSBTAG00000021491	0.081805718	0.046495707	-0.035310011
Q2HJ54	PITPNA	bta:616550; Q2HJ54; Q9TR37	ENSBTAG00000011480	-0.04673105	-0.084322938	-0.037591888
A0AAA9TU53	DCBLD2	A0AAA9TU53	ENSBTAG00000014889	0.170997557	0.305222405	0.134224847
A0AAA9T5N2	PHB2	A0AAA9T5N2	ENSBTAG00000013123	-0.089443087	-0.135577546	-0.046134458
F1N6W6	SNX9	F1N6W6	ENSBTAG00000006323	-0.158931082	-0.18150893	-0.022577848
A0AAA9SHB3	TP53BP2	A0AAA9SHB3	ENSBTAG00000012781	0.214175516	0.036305264	-0.177870253
A0AAA9SWE4	ANXA11	A0AAA9SWE4	ENSBTAG00000020940	-0.010183772	0.101226002	0.111409774
E1BF68	DHX16	bta:506405; E1BF68	ENSBTAG00000006960	0.00410051	0	-0.00410051
A0A3Q1NM85	KIF13B	A0A3Q1NM85	ENSBTAG00000017232	-0.063193826	-0.00737953	0.055814296
Q148I9	AMACR	Q148I9		-0.037116583	-0.23114348	-0.194026896
A0A3Q1N4F3	GPHN	A0A3Q1N4F3	ENSBTAG00000004171	-0.033008762	-0.082542148	-0.049533386
A0A3Q1MKB1	CPSF1	A0A3Q1MKB1	ENSBTAG00000008355	-0.16651725	-0.140495993	0.026021257
Q1LZH7	KANK2	bta:767586; Q1LZH7	ENSBTAG00000009568	0.080170349	0.044643915	-0.035526434
Q5E9D4	BCAS2	bta:507944; Q5E9D4	ENSBTAG00000016705	-0.047129087	-0.015985704	0.031143383
A0AAA9TN35	OSBPL9	A0AAA9TN35	ENSBTAG00000021170	-0.068358793	-0.002905731	0.065453061
Q3SWX8	RBBP7	bta:537402; Q3SWX8	ENSBTAG00000002820	0.126488529	0.031271462	-0.095217067
A7MB23	PICALM	A7MB23; bta:513579	ENSBTAG00000001657	-0.114559764	-0.00947068	0.105089084
A0AAF7A2L6	CYRIB	A0AAF7A2L6	ENSBTAG00000020801	-0.013490234	0.01926586	0.032756093
A0A3Q1MNT5	EXOC7	A0A3Q1MNT5	ENSBTAG00000007910	0.106581311	-0.009378332	-0.115959643
Q5E9X4	LRRC59	bta:532659; Q17QQ7; Q5E9X4	ENSBTAG00000006072	0.154446269	0.021573412	-0.132872857
E1BND0	USP24	bta:532257; E1BND0	ENSBTAG00000016990	-0.242229791	-0.220155338	0.022074453
Q3SWZ6	SBDS	bta:513237; Q3SWZ6	ENSBTAG00000004051	0.114206904	0.09887525	-0.015331654
A0AAA9S7S4	WDR26	A0AAA9S7S4	ENSBTAG00000011785	-0.047071834	0.048301332	0.095373166
G5E589	PSMB1	G5E589	ENSBTAG00000007685	0.124378846	0.072756342	-0.051622503
A0AAF6YQA7	PSMA1	A0AAF6YQA7	ENSBTAG00000006564	0.08419835	0.072080449	-0.012117902
A0A3Q1MH18	PIEZO1	A0A3Q1MH18	ENSBTAG00000020944	-0.104451164	-0.10609339	-0.001642226
A0AAF6ZDQ7	PRPSAP1	A0AAF6ZDQ7	ENSBTAG00000030172	0.019695801	-0.014950341	-0.034646143
A6QR19	ENO2	A6QR19; bta:526006; F1N123		0.045415458	0.081081308	0.035665849
A0A3Q1LXA8	PPA2	A0A3Q1LXA8	ENSBTAG00000002428	0.019840912	-0.023014566	-0.042855479
E1BF96	PPP1R10	bta:510825; E1BF96	ENSBTAG00000006933	-0.091056543	0.049563101	0.140619643
A0A3Q1LXV4	DNAJC10	A0A3Q1LXV4	ENSBTAG00000008390	0.020137646	0.025840005	0.005702359
A0AAA9SEE8	MYCBP2	A0AAA9SEE8	ENSBTAG00000018851	0	0.002856823	0.002856823
E1B8E7	HTT	E1B8E7	ENSBTAG00000001506	-0.239465935	-0.248743748	-0.009277814
F6RWK1	ARHGAP1	bta:512817; F6RWK1	ENSBTAG00000016501	-0.002820519	-0.054556164	-0.051735645
A5PKI2	ZC3H11A	A5PKI2; bta:529699; F6QB05		0.112991269	-0.020814717	-0.133805986
F1MJ55	CSNK2A2	F1MJ55	ENSBTAG00000013784	0.010175221	0.050173289	0.039998068
A0AAA9T5P2	FAM98B	A0AAA9T5P2	ENSBTAG00000021870	0.081196083	0.066769012	-0.014427071
A0A3Q1MNU7	RBM26	A0A3Q1MNU7	ENSBTAG00000017573	0.247372005	0.158320817	-0.089051188
A0AAF6Z704	RPL10A	A0AAF6Z704	ENSBTAG00000019494	-0.122300565	-0.119435231	0.002865334
A0A3Q1N9E9	LAMA4	A0A3Q1N9E9; bta:529670	ENSBTAG00000008817	-0.114804475	-0.279819563	-0.165015087
A0AAA9SBK0	PAWR	A0AAA9SBK0	ENSBTAG00000043949	0.112474729	0.081487037	-0.030987692
A0A3Q1LN78	ARFIP1	A0A3Q1LN78	ENSBTAG00000008438	0.015028209	-0.00757322	-0.022601429
A0A140T832	RPS17	A0A140T832	ENSBTAG00000000622	-0.063952633	-0.111031312	-0.04707868
Q3SX47	HNRPC	bta:510852; Q3SX47		0.186190056	0.274033405	0.087843349
A0A3Q1MVT2	SRSF1	A0A3Q1MVT2	ENSBTAG00000014766	0.11075443	0.147661006	0.036906575
E1BE97	KDM3B	E1BE97	ENSBTAG00000010063	-0.207767806	-0.192645078	0.015122728
A0AAF6Z7G1				-0.18766167	-0.177746202	0.009915469
G3N314	DNAAF5	G3N314	ENSBTAG00000048140	-0.046764467	-0.247420088	-0.200655621
F1MRK2	SPEN	F1MRK2	ENSBTAG00000018729	0.030171528	-0.153389634	-0.183561162

Accession	Gene_Symbol	Gene_ID	Ensembl_Gene ID	log2(ratio(CM12H/CM0H))	log2(ratio(CM12R/CM0H))	log2(ratio(CM12H/CM12R))
A7YW33	POLDIP3	A7YW33; bta:512693; F1N375	ENSBTAG00000016511	0.085442936	-0.067185242	-0.152628178
A0AAF6ZHS4	DDAH1	A0AAF6ZHS4	ENSBTAG00000034776	-0.146196666	-0.148196244	-0.001999578
P79126	PPM1G	bta:286880; P79126; Q3ZBB4	ENSBTAG00000019522	-0.384480032	-0.062206423	0.322273609
A0AAA9SB79	TP53	A0AAA9SB79	ENSBTAG00000001069	0.122823628	0.019343687	-0.103479941
A0A140T894	YWHAB	A0A140T894; bta:286863	ENSBTAG00000016846	0.176233903	0.111093298	-0.065140605
A0AAF6YYL2	SLC25A4	A0AAF6YYL2	ENSBTAG00000013208	-0.009037045	-0.015526913	-0.006489867
E1BC78	ATAD2	E1BC78	ENSBTAG00000002002	-0.244319939	-0.587640606	-0.343320667
E1BG87	HAUS6	E1BG87	ENSBTAG00000019843	0.009504849	0.005439008	-0.004065841
P68399	CSNK2A1	bta:282419; P19138; P20426; P68399; Q14013; Q2KI05	ENSBTAG00000012341	0.039791544	0.042495753	0.002704209
F1MHQ5	RNMT	F1MHQ5	ENSBTAG00000009142	0.123477395	0.19228291	0.068805515
P02584	PFN1	bta:513895; P02584; Q3ZCH4; Q5E942	ENSBTAG00000004915	0.065265771	0.052726576	-0.012539196
A4FUB9	ACIN1	A4FUB9		0.124704765	0.002828815	-0.121875951
Q2KIL3	ALAD	Q2KIL3		0.00877021	0.039052777	0.030282567
G3MX37	ZC3H18	G3MX37	ENSBTAG00000019520	0.197411898	0.064641252	-0.132770646
F6RZ38	CHERP	F6RZ38	ENSBTAG00000005289	0.035518659	0.036921376	0.001402718
Q3T0S6	RPL8	B0JYM1; bta:535056; Q3T0S6	ENSBTAG00000026327	-0.1214198	-0.132724031	-0.011304231
Q17QJ1	ACSF2	bta:768237; Q17QJ1	ENSBTAG00000021301	-0.244484009	-0.074000581	0.170483428
F1MLE1	DIP2B	F1MLE1	ENSBTAG00000005246	0.011427347	0.004295869	-0.007131478
A0A3Q1LSQ0	TACC1	A0A3Q1LSQ0	ENSBTAG00000004238	0.031416711	-0.318572634	-0.349989345
A0AAA9SDZ0	STK24	A0AAA9SDZ0	ENSBTAG00000032234	0.102166889	0.048171999	-0.05399489
A0AAA9TU27	VDAC2	A0AAA9TU27	ENSBTAG00000012975	0.011337544	0.033748791	0.022411247
F1MDN4	RPLP0	F1MDN4	ENSBTAG00000017389	-0.031290228	-0.103193185	-0.071902957
Q3MHP5	DRG1	bta:540161; Q3MHP5		-0.040684436	0.066776478	0.107460914
F1MB84	GRHPR	bta:504764; F1MB84	ENSBTAG00000019299	-0.140329276	-0.145250356	-0.004921079
A0A3Q1LK04	UCHL1	A0A3Q1LK04	ENSBTAG00000005078	0.105215033	0.285940317	0.180725284
E1BEP4	ASH2L	E1BEP4	ENSBTAG00000000978	0.001348944	0.10662598	0.105277037
A0A452DKI9	XPNPEP1	A0A452DKI9	ENSBTAG00000004438	-0.202473695	-0.116527087	0.085946609
A0AAA9S879	ARHGAP27	A0AAA9S879	ENSBTAG00000000571	-1.725019989	-1.823474587	-0.098454598
A0A3Q1M7L9	ATF7IP	A0A3Q1M7L9	ENSBTAG00000003221	-0.585542479	-0.478254405	0.107288074
A0AAA9SIE7				0.101481318	0.045986205	-0.055495113
F1MV22	PPAT	F1MV22	ENSBTAG00000010571	-0.013504264	-0.092512139	-0.079007874
A0AAA9TMF7	SAMHD1	A0AAA9TMF7	ENSBTAG00000022007	0.008654465	-0.031593125	-0.04024759
Q3ZBG0	PSMA7	bta:505050; Q3ZBG0	ENSBTAG00000005127	0.062558313	0.047843632	-0.014714681
A0AAA9TFV5	PTPN1	A0AAA9TFV5	ENSBTAG00000006616	-0.114879803	0.050724972	0.165604776
A0AAA9TMK4	APMAP	A0AAA9TMK4	ENSBTAG00000004278	0.007845017	0.017203072	0.009358055
Q2HJ88	RTCA	bta:510469; Q2HJ88	ENSBTAG00000006321	-0.113634106	-0.057856649	0.055777457
A0AAA9SRL1	PCDH7	A0AAA9SRL1	ENSBTAG00000000061	-0.012502972	-0.085475836	-0.072972864
Q2HJ55	SAMM50	bta:618777; Q2HJ55	ENSBTAG00000045957	-0.148717111	-0.166732268	-0.018015157
A0A3Q1MCL9	SPECC1L	A0A3Q1MCL9	ENSBTAG00000021656	0.06099063	0.092084399	0.031093769
Q0VD48	VPS4B	bta:539357; Q0VD48	ENSBTAG00000010492	0.129463005	0.14093579	0.011472784
A0AAA9SHG7	ARFGAP2	A0AAA9SHG7	ENSBTAG00000009574	0.027605941	-0.005318697	-0.032924638
A0AAA9S251	ACTL6A	A0AAA9S251	ENSBTAG00000002460	0.034426027	0.046743669	0.012317642
A0A452DJ82	ARHGDIA	A0A452DJ82	ENSBTAG00000030209	0.028261335	0.035241006	0.00697967
A0A3Q1NCA0	MAP4K4	A0A3Q1NCA0	ENSBTAG00000013023	-0.077928575	0.039384476	0.117313051
E1B7R0	DIS3L2	bta:519222; E1B7R0	ENSBTAG00000011964	-0.018059972	-0.052007303	-0.033947332
P07688	CTSB	bta:281105; P07688; Q3ZC03	ENSBTAG00000012442	0.100712031	0.214346137	0.113634106
A0AAA9TFI9	NCLN	A0AAA9TFI9	ENSBTAG00000020759	-0.03638415	-0.03496766	0.00141649
A6H767	NAP1L1	A6H767; bta:790872	ENSBTAG00000061413	0.015893646	-0.097668912	-0.113562559
A0AAA9SKD1	CDK2	A0AAA9SKD1	ENSBTAG00000004021	0.113321824	0.168122759	0.054800934
F1N1H2	MAP1S	F1N1H2	ENSBTAG00000020709	0.081149067	0.075181344	-0.005967723
Q2KJH9	ALDH9A1	bta:537539; Q2KJH9		-0.077185815	0.094844333	0.172030148
A0AAA9SJZ2	XRN1	A0AAA9SJZ2	ENSBTAG00000011524	-0.282716772	-0.223696258	0.059020514
A0A3Q1MBT8	BPTF	A0A3Q1MBT8	ENSBTAG00000014598	0.048453268	-0.022068423	-0.070521691
Q2KJ80	VPS33A	bta:510197; Q2KJ80		-0.051138849	-0.001436232	0.049702617
Q3ZBG1	RAB14	bta:539180; F1MY39; Q3ZBG1		0.011438673	0.045220393	0.03378172
A4FUA8	CAPZA1	A4FUA8; bta:534656	ENSBTAG00000014295	0.084808388	0.041659152	-0.043149236
F1MTI2	CDKN2AIP	F1MTI2	ENSBTAG00000000957	-0.003825089	0.051284547	0.055109636
G3N3U9	MRPS22	G3N3U9	ENSBTAG00000047608	-0.168122759	-0.251538767	-0.083416008
E1BLG8	AMPD2	bta:514185; E1BLG8	ENSBTAG00000013018	-0.143574471	-0.167109986	-0.023535514
A7MB69	DAPK3	A7MB69; bta:525506	ENSBTAG00000020417	0.088337467	-0.013073054	-0.101410521
A0A3Q1M1S1	NCOA5	A0A3Q1M1S1	ENSBTAG00000014186	0.100283959	0.013966179	-0.08631778
F1MYV9	OXSR1	bta:526949; F1MYV9	ENSBTAG00000014927	-0.020091418	0.09646284	0.116554257
A0AAA9SI49	KPNA4	A0AAA9SI49	ENSBTAG00000017416	0.068045387	-0.017870677	-0.085916064
A0AAA9SVG0	CDR2L	A0AAA9SVG0	ENSBTAG00000039466	0.197128564	0.068746167	-0.128382397
P84080	ARF1	A5D7F1; bta:338058; P10947; P32889; P84080; Q3SZG1	ENSBTAG00000007725	0.033133744	-0.032421478	-0.065555222
Q0VCQ9	RCN2	bta:512717; F1MQB4; Q0VCQ9	ENSBTAG00000015780	0.199735313	0.069696497	-0.130038816
F1MNY2	CEMIP2	bta:515491; F1MNY2	ENSBTAG00000005090	-0.286397359	-0.291063763	-0.004666403
A0AAA9RXF3	IKBIP	A0AAA9RXF3	ENSBTAG00000021660	-0.037814736	-0.173698165	-0.135883428
A0A3Q1LRF2	UPF2	A0A3Q1LRF2	ENSBTAG00000000288	0.140543978	0	-0.140543978
F2Z4H8	TIAL1	F2Z4H8	ENSBTAG00000004080	0.208063053	0.072260435	-0.135802618
E1BDV7	NAA25	E1BDV7	ENSBTAG00000006666	-0.13188564	-0.130545468	0.001340172
Q2KIW9	CMPK1	bta:509965; Q2KIW9		0.140609435	0.130703692	-0.009905743
A4IF71	PKP2	A4IF71; bta:537784; F1N7H1	ENSBTAG00000002651	-0.032625147	-0.125367967	-0.092742819
A0A3Q1MBN8	EXOC1	A0A3Q1MBN8	ENSBTAG00000032637	-0.076335345	-0.06133838	0.014996965
A0AAA9U0F6	SH3GL1	A0AAA9U0F6	ENSBTAG00000006007	-0.114111675	-0.136116983	-0.022005308
A0AAA9TRI5	IGF2BP2	A0AAA9TRI5	ENSBTAG00000007666	-0.00869096	-0.001444862	0.007246098
A0AAA9TE19	NUP54	A0AAA9TE19	ENSBTAG00000010955	0.177809059	0.128807158	-0.0490019
Q0VBZ0	CSK	bta:509246; Q0VBZ0	ENSBTAG00000021424	0.018328496	0.045391903	0.027063407
Q58DQ3	RPL6	A3KMZ3; bta:511051; Q58DQ3	ENSBTAG00000031723	-0.11900718	-0.117476469	0.001530711
G5E5P2	CASK	G5E5P2	ENSBTAG00000020136	0.14059833	0.149160344	0.008562014
A0A3Q1M2A3	PPIP5K2	A0A3Q1M2A3	ENSBTAG00000001485	-0.027451685	0.064983945	0.09243563
A0A3Q1LPM7	MAP3K7	A0A3Q1LPM7	ENSBTAG00000002625	0.067462968	0.027364899	-0.040098069
F1N4Q2	METAP2	F1N4Q2	ENSBTAG00000019944	-0.033662167	-0.320597809	-0.286935642
F1MQ96	PRKCI	bta:528478; F1MQ96	ENSBTAG00000001403	-0.092523776	-0.10003783	-0.007514054
A0A3Q1MNX0	RASA1	A0A3Q1MNX0	ENSBTAG00000009565	-0.249160973	-0.19095519	0.058205783
A0A3Q1MHS1	PRPS2	A0A3Q1MHS1	ENSBTAG00000047997	-0.102098188	-0.129843178	-0.02774499
Q5E946	PARK7	bta:511268; Q3ZC96; Q5E946	ENSBTAG00000020518	0.076512894	0.076512894	0

Accession	Gene_Symbol	Gene_ID	Ensembl_Gene ID	log2(ratio(CM12H/CM0H))	log2(ratio(CM12R/CM0H))	log2(ratio(CM12H/CM12R))
O18789	RPS2	A2V9C7; bta:286867; O18789; Q3T0W5		-0.102401007	-0.132835683	-0.030434675
A0AAA9S628	CC2D1A	A0AAA9S628	ENSBTAG00000006068	0.037358251	0	-0.037358251
A0AAA9S2X0	CMTR1	A0AAA9S2X0	ENSBTAG00000017091	-0.174637129	-0.018235571	0.156401557
Q29S22	DDX47	bta:534721; Q29S22	ENSBTAG00000019608	-0.110981045	-0.256514306	-0.145533261
Q0VC68	SEPTIN5	bta:615408; Q0VC68	ENSBTAG00000008320	-0.036926569	-0.003476375	0.033450194
A0A3Q1MHE5	DLST	A0A3Q1MHE5	ENSBTAG00000006463	0.06340917	0.066291679	0.002882509
Q2T9V5	TRMT6	bta:505816; Q2T9V5		-0.178369471	-0.09752507	0.0808444
F1MVW4	XPO4	bta:506392; F1MVW4	ENSBTAG00000003710	-0.072116992	-0.107505625	-0.035388634
Q5W9D7	QKI	bta:493722; Q5W9D7	ENSBTAG00000011593	-0.087652682	-0.128571336	-0.040918654
F1N2K1	PCYOX1	A6QPR1; bta:100125835; F1N2K1	ENSBTAG00000002783	-0.075802824	-0.087237719	-0.011434895
A0A3Q1MLQ2				-0.025921459	0.051791209	0.077712669
F6QNE3	KIFC1	F6QNE3	ENSBTAG00000001631	-0.120006529	0.098476426	0.218482955
A0A3Q1NC60	ZMYND8	A0A3Q1NC60	ENSBTAG00000013114	-0.011825435	0.052046748	0.063872183
A7YWH5	DKC1	A7YWH5; bta:525619		-0.225010959	-0.050829944	0.174181015
A0AAA9T915	GTPBP4	A0AAA9T915	ENSBTAG00000014917	-0.515381632	-0.453644174	0.061737458
A0AAA9TSF9	RNF214	A0AAA9TSF9	ENSBTAG00000019358	-0.140668024	-0.126795849	0.013872175
A0AAA9U1D0	PRPF4	A0AAA9U1D0	ENSBTAG00000004571	0.043027284	0.001408194	-0.041619089
A5PJU0	NOL6	A5PJU0; bta:782674	ENSBTAG00000003323	-0.125296278	-0.23188671	-0.106590431
A0A3Q1M4P9	FAM83H	A0A3Q1M4P9	ENSBTAG00000038682	0.04026387	0.069741071	0.029477201
A0A3Q1MJU0	RAB2A	A0A3Q1MJU0	ENSBTAG00000000948	-0.044966731	0.036182336	0.081149067
A6H769	RPS7	A6H769; bta:505507	ENSBTAG00000016224	-0.281539875	-0.250360576	0.031179299
Q08E38	PRPF19	bta:513868; Q08E38	ENSBTAG00000014366	0.032191286	0.156877816	0.124686531
Q3SX41	LUC7L3	bta:539955; Q3SX41	ENSBTAG00000002279	0.2635363	0.10734541	-0.15619089
Q1RMM1	ZC3H15	bta:535102; Q1RMM1	ENSBTAG00000021762	0.221087405	0.024146087	-0.196941318
Q29466	ATP6V0A1	bta:286768; Q29466		0.145386325	0.155541083	0.010154758
A0AAF6Z0I1	SPARC	A0AAF6Z0I1	ENSBTAG00000014835	0.253228648	-0.545286727	-0.798515375
Q08DT0	DDX10	bta:528483; F1MQL8; Q08DT0	ENSBTAG00000002382	-0.210977814	-0.103649231	0.107328584
A0A3Q1M6A2	CD109	A0A3Q1M6A2	ENSBTAG00000013222	-0.234384201	-0.265516818	-0.031132617
E1BDK6	LAMB2	E1BDK6	ENSBTAG00000022635	-0.165868415	-0.266986042	-0.101117627
Q0P5I7	UBA3	bta:512647; F1MIY7; Q0P5I7		-0.220378328	-0.202178149	0.018200179
A6QLN5	PI4K2A	A6QLN5; bta:507860	ENSBTAG00000002010	0.383797987	0.195771179	-0.188026808
F1N703	UBE3C	F1N703	ENSBTAG00000008025	0.305827568	0.320905753	0.015078185
A0AAA9S105	DNAJC2	A0AAA9S105	ENSBTAG00000003784	-0.016301812	-0.020406211	-0.004104398
A0A3Q1M133	FYCO1	A0A3Q1M133	ENSBTAG00000015698	-0.051821196	-0.116568377	-0.064747181
F6REA4	ACY1	F6REA4	ENSBTAG00000032304	-0.154131676	-0.071346801	0.082784875
P40673	HMGB2	bta:540444; P40673; Q32L56	ENSBTAG00000015101	0.006355495	0.114742086	0.108386591
Q3ZBD0	PSMD7	bta:512456; Q3ZBD0	ENSBTAG00000018425	0.005630037	0.066152335	0.060522298
Q28009	FUS	bta:280796; Q0P5J2; Q28009		-0.149926928	0.021415955	0.171342884
Q3SZ56	NUP88	bta:504786; Q3SZ56		-0.065318338	-0.027843633	0.037474705
A0A7S6SNS8				0.05626822	-0.006160985	-0.062429204
F1MND5	RBM12	bta:100138139; F1MND5	ENSBTAG00000044015	-0.0442623	-0.008461579	0.035800721
A5D9C3	ZC3HC1	A5D9C3		0.061986887	0.093988828	0.032001942
A0AAA9RYV2	ZPR1	A0AAA9RYV2	ENSBTAG00000002650	-0.048822479	-0.098002475	-0.049179996
A6QR33	VPS18	A6QR33; bta:518781	ENSBTAG00000011567	0.01779297	0.002980776	-0.014812194
A0A3Q1MAU7	ESD	A0A3Q1MAU7	ENSBTAG00000003415	-0.009257968	0.137783143	0.147041111
A0A140T8B2	THOC5	A0A140T8B2	ENSBTAG00000013150	-0.070596033	-0.064819468	0.005776564
A0AAA9T703	ASL	A0AAA9T703	ENSBTAG00000015314	0.065095028	0.016052405	-0.049042623
Q3T0S7	RTRAF	bta:513083; Q3T0S7	ENSBTAG00000021944	0.186726321	-0.094586819	-0.28131314
P00517	PRKACA	bta:282322; P00517	ENSBTAG00000006642	-0.136753877	0	0.136753877
A6QQP4	SCARB2	A6QQP4; bta:533177; F1N1Q4		0.120294234	0.314646756	0.194352522
F1MS45	CPNE2	bta:782388; F1MS45	ENSBTAG00000002233	-0.018033923	0.008933125	0.026967048
A0AAA9T1Y3	PTBP3	A0AAA9T1Y3	ENSBTAG00000016750	-0.110676231	-0.061979669	0.048696562
E1BPC3	ATP8B1	bta:541187; E1BPC3	ENSBTAG00000005738	-0.640078283	-0.573596802	0.066481481
A0A3Q1ML81	HTATSF1	A0A3Q1ML81; bta:526910	ENSBTAG00000054942; ENSBTAG00000055212	0.178947131	0.144389909	-0.034557222
A0AAF6Z657	PDIA5	A0AAF6Z657	ENSBTAG00000018877	0.052418534	0.01903678	-0.033381754
A6QL88	SACM1L	A6QL88; bta:530577	ENSBTAG00000003501	-0.060803279	-0.018437248	0.042366031
F1N2F1	NIBAN1	F1N2F1	ENSBTAG00000014548	0.101437255	0.120657457	0.019220202
A0AAA9TUL1	RABGGTA	A0AAA9TUL1	ENSBTAG00000018796	-0.142019005	-0.115262547	0.026756458
Q3B7M5	LASP1	bta:532851; Q3B7M5	ENSBTAG00000030587	0.038925512	0.055527479	0.016601967
A0A3Q1MFG3	RPSA	A0A3Q1MFG3	ENSBTAG00000009757	-0.087381317	-0.062648796	0.024732521
A0AAA9SCD4	MTHFD2	A0AAA9SCD4	ENSBTAG00000004881	-0.14917942	-0.225019362	-0.075839942
A0AAA9T3X0	PRKAA1	A0AAA9T3X0	ENSBTAG00000000013	-0.064390587	0.007138535	0.071529123
E1BM23	ARHGAP5	E1BM23	ENSBTAG00000027024	-0.003694484	-0.041169189	-0.037474705
E1BDU9	GATAD2B	E1BDU9	ENSBTAG00000006678	-0.002734968	-0.096026888	-0.09329192
A0AAA9S219	H1-5	A0AAA9S219; bta:527304	ENSBTAG00000067558	-0.029146346	0.04772873	0.076875076
A0AAA9TQ63	FXR2	A0AAA9TQ63	ENSBTAG00000005462	0.039183263	-0.069751108	-0.108934372
E1BDK7	ELAC2	bta:509256; E1BDK7	ENSBTAG00000021939	-0.02813579	-0.040818905	-0.012683115
Q5BIP4	TOM1	bta:504912; F1MNE8; Q5BIP4	ENSBTAG00000045785	-0.057748506	0.012881291	0.070629797
A0AAA9TBQ5	PRMT3	A0AAA9TBQ5	ENSBTAG00000013421	-0.278216604	-0.112940271	0.165276333
Q29RZ2	PPWD1	bta:517560; Q29RZ2	ENSBTAG00000009514	0.273128796	0.286304185	0.013175389
Q3SZ71	PMPCB	bta:534546; Q3SZ71		-0.135249117	-0.226157613	-0.090908496
A3KN51	TSG101	A3KN51; bta:507659	ENSBTAG00000013563	0.035358175	0.012397026	-0.022961148
A0A3Q1M3K7	RAB7A	A0A3Q1M3K7	ENSBTAG00000010193	0.113613624	0.053128495	-0.060485129
A0AAA9TSW0	ERP44	A0AAA9TSW0	ENSBTAG00000009804	0.123853469	-0.023176322	-0.147029791
A0A3Q1LG54	PPP1R21	A0A3Q1LG54	ENSBTAG00000002570	0.007217099	-0.100362788	-0.107579887
A5D7A0	EFHD2	A5D7A0; bta:514259	ENSBTAG00000009048	0.110768071	0.1458782	0.035110129
A0AAF6Z1S7	SMU1	A0AAF6Z1S7	ENSBTAG00000019564	0.102991232	-0.11733182	-0.220323052
F1N0S9	FAM98A	F1N0S9	ENSBTAG00000008548	0.105310843	-0.01504388	-0.120354723
G5E5T1	EPS8L2	G5E5T1	ENSBTAG00000022147	-0.031148699	-0.002804073	0.028344626
A0A3Q1LV44	ALDH3A2	A0A3Q1LV44	ENSBTAG00000039161	-0.038708774	-0.142198612	-0.103489838
A0AAA9S6D6	AFAP1	A0AAA9S6D6	ENSBTAG00000012464	-0.223679967	-0.16190377	0.061776198
P04409	PRKCA	bta:282001; P04409		0.017175144	-0.015634416	-0.03280956
F6PZZ7	VPS4A	F6PZZ7	ENSBTAG00000001659	0.087806381	0.053040963	-0.034765418
Q5EA53	GTF2F1	A6H7C0; bta:505702; Q5EA53	ENSBTAG00000021016	0.081071611	-0.072684454	-0.153756065
A0AAA9S6L2	ATP5PB	A0AAA9S6L2	ENSBTAG00000006441	-0.01926586	0.034905615	0.054171474
Q3SWX5	CDH6	bta:537946; Q3SWX5	ENSBTAG00000012992	-0.008988783	0.069962558	0.078951341
A5PJR9	GNL1	A5PJR9; bta:532528; F1MWB9	ENSBTAG00000025516	0.018838505	0.061861354	0.043022849
F1N399	SNX1	F1N399	ENSBTAG00000002014	-0.02739833	-0.00429161	0.023106721
F1N091	CCDC6	F1N091	ENSBTAG00000003189	0.209453366	0.123382416	-0.08607095

Accession	Gene_Symbol	Gene_ID	Ensembl_Gene ID	log2(ratio(CM12H/CM0H))	log2(ratio(CM12R/CM0H))	log2(ratio(CM12H/CM12R))
A2VE06	RPS4Y1	A2VE06; bta:286873	ENSBTAG00000025617	-0.072824368	-0.180484573	-0.107660205
Q3SZK8	NHERF1	bta:505242; Q3SZK8	ENSBTAG00000011382	0.265659875	0.183004828	-0.082655047
A2VDZ9	VAPB	A2VDZ9; bta:326580	ENSBTAG00000017424	0.12995274	0.119919053	-0.010033687
A0AAA9TE78	ADRM1	A0AAA9TE78	ENSBTAG00000003058	0.010103959	-0.023363993	-0.033467952
E1BJ75	CRTAP	bta:540924; E1BJ75	ENSBTAG00000021442	-0.053833479	-0.188788071	-0.134954592
A0JNM0	ATXN7L3B	A0JNM0; bta:777786	ENSBTAG00000035083	-0.841379028	-0.778397447	0.06298158
A0A3Q1MRJ9	NECTIN2	A0A3Q1MRJ9	ENSBTAG00000015318	0.112274785	0.215062104	0.102787318
A0A3Q1MXH0	CUL5	A0A3Q1MXH0	ENSBTAG00000012882	-0.060957544	0.04161219	0.102569734
Q3T145	MDH1	bta:535182; Q3T145	ENSBTAG00000019295	0.100058863	0.094188275	-0.005870588
E1BPL8	MME	bta:536741; E1BPL8	ENSBTAG00000002075	0.032647234	0	-0.032647234
P02694	RBP1	bta:537379; P02694; Q53J07; Q9TS50		0.033166864	0.00912139	-0.024045474
A0AAF6Z2Q2	METAP1	A0AAF6Z2Q2	ENSBTAG00000016004	0.004131826	-0.041982449	-0.046114276
Q3MHX3	RBM4	A5D970; bta:767937; Q3MHX3	ENSBTAG00000031688	0.480516913	0.141508334	-0.339008579
E1BH11	SH3BP1	bta:100297734; E1BH11	ENSBTAG00000030257	-0.050460052	0.053242021	0.103702073
A0AAA9SI20	ATXN10	A0AAA9SI20	ENSBTAG00000009351	0.172123845	-0.021487345	-0.19361119
Q17QH2	MAP2K2	bta:510434; F1MYR6; Q17QH2	ENSBTAG00000024450	-0.011202347	0.014276373	0.02547872
Q0IIG7	RAB5A	bta:539764; Q0IIG7		-0.095502087	-0.104371141	-0.008869055
E1BF82	NDC80	bta:538789; E1BF82	ENSBTAG00000021673	0.131603949	0.005501227	-0.126102722
Q3SYT5	PNN	bta:100296970; F1N3M1; Q3SYT5	ENSBTAG00000026995	0.155388908	0.053242021	-0.102146887
E1BKV4	BACE2	E1BKV4	ENSBTAG00000000394	0.620654208	0.081071611	-0.539582597
A4FUG8	RABEP2	A4FUG8; bta:511736	ENSBTAG00000006542	0.017424065	-0.066363118	-0.083787183
A4IF81	HNRPDL	A4IF81; bta:534770		-0.38545232	-0.232263531	0.153188789
A5D7P5	TUBGCP2	A5D7P5; bta:781376		-0.359051309	-0.257291512	0.101759797
A0A3S5ZPD8	GATAD2A	A0A3S5ZPD8	ENSBTAG00000003034	0.070721173	-0.102525716	-0.173246889
A4FUY8	IK	A4FUY8; bta:100125318; F1MWA0		0.251206119	-0.133490076	-0.384696195
A0AAA9SDI5	ALDH6A1	A0AAA9SDI5	ENSBTAG00000018469	-0.018635717	0.013820495	0.032456212
Q3T0V9	DERA	bta:534558; Q3T0V9	ENSBTAG00000022167	0.004597014	-0.007694392	-0.012291406
A0A3Q1LPP5	EIF2B4	A0A3Q1LPP5	ENSBTAG00000019510	-0.124781625	-0.074723918	0.050057707
F1MZC0	AKR7A2	F1MZC0	ENSBTAG00000012289	0.055495113	0.095758983	0.04026387
A0AAA9TUA9	USP48	A0AAA9TUA9	ENSBTAG00000006940	-0.354916507	-0.148029506	0.206887001
E1BE68	PIK3C2A	E1BE68	ENSBTAG00000004901	-0.105442315	-0.070978905	0.03446341
Q17QL4	MAGED2	bta:514372; Q17QL4	ENSBTAG00000039888	-0.06586565	-0.472607007	-0.406741358
A0A3Q1NKI3	KPNA3	A0A3Q1NKI3; bta:540719	ENSBTAG00000004850	0.149331565	0.10821904	-0.041112525
A0A3Q1LV49	RNF213	A0A3Q1LV49	ENSBTAG00000022489	-0.162303774	-0.058214614	0.10408916
A0AAA9U1A2	NUSAP1	A0AAA9U1A2	ENSBTAG00000010774	0.438963339	0.320318842	-0.118644496
F1MIB2	GAK	F1MIB2	ENSBTAG00000019188	-0.01153503	0.057908578	0.069443608
A0AAA9S909	RFC2	A0AAA9S909	ENSBTAG00000018589	-0.031057257	0.035862335	0.066919592
E1BMX5	NRP1	E1BMX5	ENSBTAG00000019866	-0.08155963	0.21469803	0.296257659
Q3SZ99	AIP	bta:360190; F1MPM7; Q3SZ99		-0.066726114	-0.021900915	0.044825199
F1MYN2	CGN	F1MYN2	ENSBTAG00000017564	0.064130337	0.114559764	0.050429427
A6QLS8	WDR11	A6QLS8; bta:525169	ENSBTAG00000021540	-0.033659311	-0.041419927	-0.007760616
P80311	PPIB	bta:281419; P80311; Q5E9F4	ENSBTAG00000016822	0.186640095	0.158728957	-0.027911138
A0A3Q1NNK5	RBM27	A0A3Q1NNK5	ENSBTAG00000011642	0.048626968	0.108831603	0.060204634
Q58D72	ATL1	bta:535424; Q3ZBC1; Q58D72	ENSBTAG00000014312	-0.050123479	-0.008233912	0.041889568
A0AAF6ZDE8	SNX5	A0AAF6ZDE8	ENSBTAG00000003422	-0.1689671	-0.133217075	0.035750025
A6H7A9	LOC790875	A6H7A9; bta:790875		-0.054647396	-0.003463855	0.051183541
Q58CQ2	ARPC1B	bta:326600; Q0V8Q8; Q58CQ2; Q58DS1	ENSBTAG00000046248	-0.103093493	-0.038653431	0.064440062
A5D7U5	GLB1	A5D7U5		0.098879456	0.14410871	0.045229254
A0A3Q1M2Z8	RAPH1	A0A3Q1M2Z8	ENSBTAG00000027201	0.03280769	-0.006334566	-0.039142256
F1MFF9	CHD5	F1MFF9	ENSBTAG00000040477	-0.061107463	-0.661982203	-0.60087474
P12234-2	SLC25A3	P12234-2		-0.001569	0.003132889	0.004701889
P01017	AGT	A0A3Q1LGY9; P01017	ENSBTAG00000012393	-0.370601942	0.019018854	0.389620796
G3N1K4	MED12	G3N1K4	ENSBTAG00000021351	-0.061029433	-0.046262723	0.014766711
A0AAA9TFP3	PDZRN3	A0AAA9TFP3	ENSBTAG00000018644	0.233334172	-0.035096375	-0.268430546
E1BMF1	TM9SF3	bta:614147; E1BMF1	ENSBTAG00000016298	0.084236537	0.193405792	0.109169255
A0A3Q1MRX3	NVL	A0A3Q1MRX3	ENSBTAG00000006718	-0.029630964	-0.149987181	-0.120356217
A0AAF6YIM3	LGALS3BP	A0AAF6YIM3	ENSBTAG00000001368	0.036525876	0.215056668	0.178530792
F1MHD9	LARP4	F1MHD9	ENSBTAG00000017164	-0.064907263	-0.352145937	-0.287238674
F1MY62	GBP1	F1MY62	ENSBTAG00000001143	0.144219287	0.263432446	0.119213158
F1MBI1	PSMB7	F1MBI1	ENSBTAG00000003067	0.019736218	0.009243844	-0.010492374
A0AAA9TVM3	SNX6	A0AAA9TVM3	ENSBTAG00000012873	0.01771753	0.054966459	0.037248929
A6QLX2	FBL	A6QLX2; bta:538548		-0.317702557	-0.106714899	0.210987658
A0A3Q1LQH8	GNPAT	A0A3Q1LQH8	ENSBTAG00000010773	-0.095356974	0.051787237	0.14714421
A0A3Q1LM83	PPP4R3B	A0A3Q1LM83	ENSBTAG00000000545	-0.221265756	-0.148250959	0.073014797
F1N371	DAB2	bta:509221; F1N371	ENSBTAG00000016152	0.084619682	-0.069977188	-0.15459687
A7MB35	PDHA1	A7MB35; bta:407109	ENSBTAG00000019852	0.085620426	0.112190146	0.02656972
A7E3S8	ST13	A7E3S8; bta:510494	ENSBTAG00000006202	0.238869935	0.224957628	-0.013912307
A5PKF6	SMARCE1	A5PKF6; bta:540910		0.129145151	0.039658766	-0.089486386
A0A452DJ17	TWF1	A0A452DJ17	ENSBTAG00000020938	-0.110972786	-0.028071757	0.082901029
P08760	AK3	bta:281613; P08760; Q29RI4	ENSBTAG00000017147	0.112894056	0.091010939	-0.021883118
A0A3Q1MC72	CELF1	A0A3Q1MC72	ENSBTAG00000002520	-0.033423002	0.116324118	0.14974712
E1BM26	PSME3	E1BM26	ENSBTAG00000019918	-0.077862152	-0.059314852	0.018547299
E1BKZ1	GSR	bta:506406; E1BKZ1	ENSBTAG00000007115	-0.056075924	0.124237565	0.180313489
E1B768	LARS2	E1B768	ENSBTAG00000007315	-0.316814816	-0.253663883	0.063150933
Q1RMW4	APPL2	bta:515605; Q1RMW4		-0.170286806	-0.016005858	0.154280948
F1MLP7	SNRNP40	F1MLP7	ENSBTAG00000016817	-0.005906641	0.062014431	0.067921072
E1BNE7	CAVIN1	E1BNE7	ENSBTAG00000039684	0.219984585	0.013039295	-0.20694529
A7E3T7	MAT2A	A7E3T7; bta:527403	ENSBTAG00000020873	-0.226169112	-0.220884509	0.005284603
A5D7G6	STT3B	A5D7G6; bta:504474	ENSBTAG00000007438	0.154600205	0.136321471	-0.018278734
A0AAA9S166	TASOR	A0AAA9S166	ENSBTAG00000019748	-0.133546189	-0.140354578	-0.006808389
A0AAA9SZ05	AP1M1	A0AAA9SZ05	ENSBTAG00000013329	0.005998741	0.076102979	0.070104238
A0AAA9RVE1	CRKL	A0AAA9RVE1	ENSBTAG00000065402	0.150573975	0.11584343	-0.034730545
A0A3Q1LUI6	MSH3	A0A3Q1LUI6	ENSBTAG00000007684	0.03058832	0.119999776	0.089411456
Q17QZ9	IFIT5	bta:515091; Q17QZ9	ENSBTAG00000017367	0.130872225	0.143846189	0.012973964
F6REL8	MICALL2	F6REL8	ENSBTAG00000003049	0.041875355	0.244569212	0.202693857
A0AAA9RYE2	BUB3	A0AAA9RYE2	ENSBTAG00000017913	0.149117702	0.075801025	-0.073316678
A6H737	LOXL2	A6H737; bta:532684; F6RUG1		-0.154741955	-0.513400965	-0.358659009
A0A3S5ZPM2	HAT1	A0A3S5ZPM2	ENSBTAG00000010118	0.181818404	0.250872521	0.069054117
G3MWR4	ATP1B1	G3MWR4	ENSBTAG00000002688	0.270333926	0.248323622	-0.022010304
P11017	GNB2	A5D7A9; bta:281202; P11017	ENSBTAG00000006495	0.053604744	0.073924728	0.020319984

Accession	Gene_Symbol	Gene_ID	Ensembl_Gene ID	log2(ratio(CM12H/CM0H))	log2(ratio(CM12R/CM0H))	log2(ratio(CM12H/CM12R))
F1MTK4	LPCAT1	F1MTK4	ENSBTAG00000000944	0.206019127	0.09884481	-0.107174316
F6R5L6	ELOA	F6R5L6	ENSBTAG00000026585	0.042828972	0.012174714	-0.030654258
F6RM04	RBM15B	bta:508868; F6RM04	ENSBTAG00000006500	-0.026073512	-0.090336737	-0.064263225
A0AAA9TX78	GSS	A0AAA9TX78	ENSBTAG00000003504	0.034765418	0.070170754	0.035405336
Q2TBX2	VBP1	bta:508727; Q2TBX2		0.042629853	-0.056285173	-0.098915026
A0AAF6DLT1	SUPV3L1	A0AAF6DLT1	ENSBTAG00000001044	0.167133058	0.053627185	-0.113505873
E1BCI2	RNF40	E1BCI2	ENSBTAG00000003421	-0.010908899	-0.044139429	-0.03323053
Q3SWY5	NOSIP	bta:511234; Q3SWY5	ENSBTAG00000014450	-0.148761809	-0.35740599	-0.208644181
Q3T0Q8	UTP14A	bta:505324; Q3T0Q8	ENSBTAG00000013671	-0.192131206	-0.423456752	-0.231325546
A0AAA9T1J2	P3H3	A0AAA9T1J2	ENSBTAG00000003598	-0.085949613	-0.145736335	-0.059786722
Q5E995	RPS6	bta:787914; Q5E995		-0.053859311	-0.015385163	0.038474148
A0A3Q1MAH9	IRGQ	A0A3Q1MAH9	ENSBTAG00000052481	0.088265751	-0.021396293	-0.109662044
P62894	CYCS	bta:100847700; bta:510767; P00006; P62894; Q2KJD4	ENSBTAG00000022613; ENSBTAG00000063185	0.251662333	0.024504964	-0.22715737
Q9N2I2	SERPINA5	A7E3W0; bta:338050; Q9N2I2		-0.362920036	-0.313380575	0.049539461
Q2KID6	PLRG1	bta:533951; Q2KID6		0.021172005	0.027164517	0.005992511
A0AAF6ZNJ0	UQCRFS1	A0AAF6ZNJ0	ENSBTAG00000046786	-0.10734541	-0.162604315	-0.055258905
F1ML98	BICD1	F1ML98	ENSBTAG00000006734	-0.039715993	-0.279336218	-0.239620225
E1BN25	BAZ1A	E1BN25	ENSBTAG00000020164	-0.319936797	-0.221064584	0.098872213
Q2TBQ5	RPL7A	A5D9D0; bta:513128; Q2TBQ5	ENSBTAG00000011559	-0.242628195	-0.118448784	0.124179411
Q1RMS5	NXF1	bta:512136; Q1RMS5	ENSBTAG00000009488	0.125648514	0.06216498	-0.063483534
A0AAA9S5T9	ELAVL1	A0AAA9S5T9	ENSBTAG00000000613	0.19759996	0.146699062	-0.050900898
A0AAF7AP66	LRRC8C	A0AAF7AP66	ENSBTAG00000000900	-0.190313763	-0.174867202	0.015446561
E1BIX3	CAPN7	E1BIX3	ENSBTAG00000006479	0.136624992	0.072228328	-0.064396665
A0AAA9TTC5	PRC1	A0AAA9TTC5	ENSBTAG00000018643	0.234278202	0.075309262	-0.158968941
E1BKM0	FIP1L1	E1BKM0	ENSBTAG00000020653	-0.232807618	-0.29919884	-0.066391222
A6QR56	ALDH16A1	A6QR56; bta:506329		-0.039938745	-0.07754667	-0.037607924
A0AAA9TCR0	STK3	A0AAA9TCR0	ENSBTAG00000026234	-0.063317111	-0.086516727	-0.023199616
A6QP36	LMAN2	A6QP36; bta:790870	ENSBTAG00000008034	0.29676688	0.276571345	-0.020195536
A0A3Q1M3W4	RPS15	A0A3Q1M3W4	ENSBTAG00000019718	-0.064011758	-0.142468877	-0.078457119
F1MY06	TNRC6B	bta:528167; F1MY06	ENSBTAG00000021076	-0.007463519	0.039649077	0.047112596
A7MB49	DDX20	A7MB49; bta:528433		-0.102242419	0.02811838	0.1303608
F1MU62	CPSF7	F1MU62	ENSBTAG00000015263	-0.043311171	-0.040565795	0.002745377
A0A3Q1MC04	TRIM47	A0A3Q1MC04	ENSBTAG00000018920	-0.043068722	-0.120743058	-0.077674336
A0AAA9SMQ6	PLCG1	A0AAA9SMQ6	ENSBTAG00000017584	0.057638626	-0.011809302	-0.069447928
A0A3Q1LR68	ITCH	A0A3Q1LR68	ENSBTAG00000000308	-0.279713147	-0.11501666	0.164696487
A0AAA9RWR1	UROD	A0AAA9RWR1; bta:504914	ENSBTAG00000012644	-0.170097914	-0.20634448	-0.036246566
F1N5K1	CDK11	F1N5K1	ENSBTAG00000010737	-0.044351467	0.086097299	0.130448766
Q769I5	MET	A4D7R6; bta:280855; Q769I5		-0.138102277	-0.191790148	-0.053687871
A0AAF6Z3T2	PYCR3	A0AAF6Z3T2	ENSBTAG00000016810	-0.074600954	-0.007145607	0.067455347
A0AAA9S9T4	ITPR1	A0AAA9S9T4	ENSBTAG00000020455	0.011366255	0.279244633	0.267878378
E1BC65	RBM6	bta:100296443; E1BC65	ENSBTAG00000006328	-0.290219235	-0.171368418	0.118850817
F1MGJ4	STAT6	bta:353105; F1MGJ4	ENSBTAG00000006335	-0.221245696	-0.069082134	0.152163562
A0A452DIC9	GNPDA2	A0A452DIC9	ENSBTAG00000000287	-0.165102618	-0.059907844	0.105194774
F2Z4E9	CPSF6	F2Z4E9	ENSBTAG00000007323	0.025814318	-0.051556611	-0.077370928
P61223	RAP1B	bta:327708; P09526; P61223; Q3T0M4	ENSBTAG00000008967	0.214278975	0.258556301	0.044277326
A0A3Q1MWV1	MAD1L1	A0A3Q1MWV1	ENSBTAG00000002474	-0.290767163	-0.28308097	0.007686193
A6QQS0	DPF2	A6QQS0; bta:513235	ENSBTAG00000003077	0.221679099	0.053893008	-0.167786091
A0A3Q1MRR8	DIAPH2	A0A3Q1MRR8	ENSBTAG00000050709	-0.240016897	-0.262439603	-0.022422706
A0AAF6YQN7	STT3A	A0AAF6YQN7	ENSBTAG00000007203	0.005674324	-0.05504926	-0.060723584
A0A452DID1	TPT1	A0A452DID1	ENSBTAG00000010156	-0.394379889	-0.422515679	-0.02813579
G3MZK6	MAP7D3	G3MZK6	ENSBTAG00000014170	0.121867775	0.200382751	0.078514976
F1N4Q3	GALNT7	F1N4Q3	ENSBTAG00000016845	-0.079187028	-0.079187028	0
A0A3Q1LUG2	APP	A0A3Q1LUG2	ENSBTAG00000017753	-0.115829481	-0.294367613	-0.178538132
A0JN38	SCFD2	A0JN38; bta:535130		-0.141152573	-0.300720782	-0.159568209
A5PKL2	RRAS2	A5PKL2; bta:519269	ENSBTAG00000011905	0.084337356	0.108407905	0.024070549
Q24JZ8	U2AF2	bta:507895; G5E532; Q24JZ8	ENSBTAG00000002830	0.014241923	0.050626073	0.03638415
A0AAA9SZI0	AFF4	A0AAA9SZI0	ENSBTAG00000009482	0.042710095	0.018841434	-0.023868661
A0A3Q1M245	KIF13A	A0A3Q1M245	ENSBTAG00000019217	0.062896667	0.068316943	0.005420276
Q24K07	VPS11	bta:540381; Q24K07		0.001586251	0.029844511	0.02825826
E1B928	ZNF598	E1B928	ENSBTAG00000001023	-0.041283681	-0.086761312	-0.045477631
Q32KL2	PSMB5	bta:534640; Q32KL2	ENSBTAG00000012726	-0.089600695	0.083015902	0.172616597
F1MLE8	SEC22B	F1MLE8	ENSBTAG00000007712	0.15759649	0.206984517	0.049388027
Q0V8A7	ARFGAP1	Q0V8A7		0.033034779	0.18645715	0.153422371
A0AAA9SIX4	VPS39	A0AAA9SIX4	ENSBTAG00000032055	0.079774439	-0.061336865	-0.141111304
A0A3Q1LHQ2	MMUT	A0A3Q1LHQ2	ENSBTAG00000014272	-0.316209411	-0.144523951	0.171685459
F1MUT0	SETD7	F1MUT0	ENSBTAG00000003581	0.118644496	0.03805419	-0.080590306
A6QLY3	P3H2	A6QLY3; bta:511799	ENSBTAG00000021025	-0.109984116	-0.199983734	-0.089999618
Q3T0D7	SAR1A	bta:517171; Q3T0D7	ENSBTAG00000016858	0.128885854	-0.015370262	-0.144256116
F1MBU2	SMPD4	F1MBU2	ENSBTAG00000002130	-0.122484007	-0.076062206	0.046421801
A0AAA9TC56	MEMO1	A0AAA9TC56	ENSBTAG00000007734	-0.010776484	0.02659421	0.037370694
Q2TBX4	HSPA13	bta:505907; Q2TBX4	ENSBTAG00000012908	0.257657228	-0.232950613	-0.490607841
F1MHM7	CEBPZ	F1MHM7	ENSBTAG00000039770	-0.338266832	-0.024908575	0.313358257
F1N2H2	SCAF11	bta:538632; F1N2H2	ENSBTAG00000001810	0.208130325	0.016218511	-0.191911815
Q5E987	PSMA5	bta:510155; Q5E987		-0.005840879	0.229867542	0.23570842
F1MQH8	RABEP1	bta:504785; F1MQH8	ENSBTAG00000011505	0.190869453	0.257700962	0.066831508
F1MPK4	HIBCH	F1MPK4	ENSBTAG00000007787	0.013504264	-0.045949586	-0.05945385
E1BMP8	SBNO1	bta:540582; E1BMP8	ENSBTAG00000020505	-0.016119665	0.060175515	0.07629518
A0A3S5ZPB0	ALDOC	A0A3S5ZPB0	ENSBTAG00000013099	0.182152704	0.208241933	0.026089229
A0AAA9T9J6	ARF4	A0AAA9T9J6	ENSBTAG00000016069	0.103001449	0.00266672	-0.100334729
A0AAF6DM82	POLR1B	A0AAF6DM82	ENSBTAG00000014166	-0.035956878	-0.065072264	-0.029115386
A7Z038	ABCD3	A7Z038; bta:526059		-0.055371264	-0.162993499	-0.107622234
E1BLZ8	EIF3F	bta:513793; E1BLZ8	ENSBTAG00000004861	0.056034035	0.065620084	0.009586049
A0AAF6YUC8	PGP	A0AAF6YUC8	ENSBTAG00000009951	0.041988068	0.100710507	0.058722439
A0AAA9TH74	PSPH	A0AAA9TH74	ENSBTAG00000013081	0.023541001	-0.034310655	-0.057851656
F1MR60	TFIP11	F1MR60	ENSBTAG00000012288	-0.029718163	0.105313544	0.135031707
P13696	PEBP1	bta:431786; P13696; Q3T0H9		0.13459964	0.172348916	0.037749277
A4FUD8	XAB2	A4FUD8; F6RY07		-0.274747826	-0.151571084	0.123176742
F1MUY2				-0.399095955	-0.342105977	0.056989979
A0A3Q1LNI4	ITPRID2	A0A3Q1LNI4	ENSBTAG00000000937	0.039528364	-0.094215342	-0.133743706

Accession	Gene_Symbol	Gene_ID	Ensembl_Gene ID	log2(ratio(CM12H/CM0H))	log2(ratio(CM12R/CM0H))	log2(ratio(CM12H/CM12R))
A0A3Q1M9D9	MOGS	A0A3Q1M9D9	ENSBTAG00000001844	-0.061266807	-0.048485372	0.012781435
A0A140T827	IMPDH1	A0A140T827; bta:504305	ENSBTAG00000000296	-0.275705078	-0.606596571	-0.330891493
A7Z023	CCDC132	A7Z023; bta:535248		0.093704453	0.071220439	-0.022484014
A0AAA9S0J8	NOP58	A0AAA9S0J8	ENSBTAG00000005517	-0.277205306	0.026866606	0.304071912
A0A3Q1M190	RFC4	A0A3Q1M190	ENSBTAG00000014727	-0.006827722	-0.072638906	-0.065811184
A5D7H2	STRN3	A5D7H2; bta:516375	ENSBTAG00000021845	0.061507407	0.108654549	0.047147142
F1MHA1	KMT2A	F1MHA1	ENSBTAG00000018093	-0.113347943	-0.054861935	0.058486008
E1BJP2	RACGAP1	E1BJP2	ENSBTAG00000012784	-0.011238188	0.026344995	0.037583183
A0AAA9SAB4	LUC7L2	A0AAA9SAB4; bta:516355	ENSBTAG00000017770	0.18686422	0.03819986	-0.14866436
F1MFJ6	TBL1XR1	bta:614346; F1MFJ6	ENSBTAG00000021910	-0.010681027	-0.054213922	-0.043532895
G3MY03	TSNAX	G3MY03	ENSBTAG00000047434	-0.181221158	-0.157962413	0.023258745
A0A3Q1LS42	HERC4	A0A3Q1LS42	ENSBTAG00000014029	-0.203813409	-0.033381754	0.170431655
A0A3Q1MDE2	AFG2A	A0A3Q1MDE2	ENSBTAG00000000125	0.107439268	-0.02815827	-0.135597538
F1MMS4	TERF2	F1MMS4	ENSBTAG00000007617	0.092331881	-0.022281447	-0.114613328
E1B8Q9	IPO8	bta:523408; E1B8Q9	ENSBTAG00000014106	0.054997277	0.050228033	-0.004769244
A0AAA9TLY4	PPIL2	A0AAA9TLY4	ENSBTAG00000021121	0.077182608	0.007061664	-0.070120944
Q32PD5	RPS19	bta:516063; Q32PD5	ENSBTAG00000011963	-0.081243796	-0.145685549	-0.064441753
A0A3Q1MD85	LRPAP1	A0A3Q1MD85	ENSBTAG00000000073	0.142504649	-0.120785803	-0.263290453
A0AAA9TJK9	CAB39	A0AAA9TJK9	ENSBTAG00000040055	0.163445448	0.058475889	-0.10496956
A0AAA9RSV1	PTCD3	A0AAA9RSV1	ENSBTAG00000006482	-0.260898661	-0.266601021	-0.005702359
Q9BGI2	PRDX4	bta:281999; Q32L06; Q9BGI2	ENSBTAG00000006165	0.035489085	0.012383724	-0.02310536
E1BPB3	EIF2B5	E1BPB3	ENSBTAG00000011414	-0.068243176	-0.025402848	0.042840328
Q1JPH6	EIF4H	A5D959; bta:517409; Q1JPH6		0.053617516	-0.085101137	-0.138718652
F1MM78	ZCCHC8	bta:539598; F1MM78	ENSBTAG00000006114	-0.060869067	-0.080126684	-0.019257617
F1MGU0	DAXX	F1MGU0	ENSBTAG00000016830	-0.02154388	-0.040479069	-0.018935189
Q07537	GALNT1	bta:282241; Q07537	ENSBTAG00000011206	0.100949388	0.149576356	0.048626968
Q3SZ73	ABHD11	bta:510109; Q3SZ73	ENSBTAG00000010339	-0.045944865	-0.110622923	-0.064678058
A0AAA9S5H4	NUP85	A0AAA9S5H4	ENSBTAG00000016126	-0.024786679	0.038512189	0.063298868
A0AAF6ZAT9	NIFK	A0AAF6ZAT9	ENSBTAG00000019387	-0.356286776	-0.339608035	0.016678741
E1BK00	CUL7	bta:504558; E1BK00	ENSBTAG00000012749	-0.146564895	-0.176877762	-0.030312867
E1BLF0	PRRC1	E1BLF0	ENSBTAG00000021977	0.073421069	0.067904065	-0.005517005
A0A3Q1LWU4	TNS3	A0A3Q1LWU4	ENSBTAG00000009655	0.119827698	0.153168773	0.033341075
Q08DF7	PDE12	bta:538860; Q08DF7	ENSBTAG00000015563	0.022464564	0.060351998	0.037887435
F6RDI0	RAD18	F6RDI0	ENSBTAG00000012142	0.105996647	0.005702359	-0.100294288
F1N6W9	COL18A1	F1N6W9	ENSBTAG00000023907	-0.20029865	-0.146078259	0.054220391
F1MZT2	CRNKL1	F1MZT2	ENSBTAG00000008472	0.024591988	0.009613425	-0.014978563
E1BKT9	DSP	E1BKT9	ENSBTAG00000015106	0.039892727	-0.060981875	-0.100874602
F1N338	HADH	F1N338	ENSBTAG00000002049	0.282730207	0.240865081	-0.041865126
A0A3Q1NNP6	ATP6V1E1	A0A3Q1NNP6	ENSBTAG00000014238	0.083945651	0.072034789	-0.011910863
P13621	ATP5PO	bta:281640; P13621; Q3T0Y7	ENSBTAG00000018278	0.017364643	0.047258753	0.029894109
G5E543	NDUFA10	G5E543	ENSBTAG00000003279	-0.086460461	-0.095662192	-0.009201731
A0AAA9RVW5	STK4	A0AAA9RVW5	ENSBTAG00000003257	0.252051065	0.103479941	-0.148571123
A0AAA9TNW6	LBR	A0AAA9TNW6	ENSBTAG00000008453	-0.052972027	-0.151315277	-0.09834325
A0AAA9TV32	INTS1	A0AAA9TV32	ENSBTAG00000000642	-0.216880281	-0.127849955	0.089030326
A5PJN8	SF3A2	A5PJN8; bta:789082		0.057333175	0.058773709	0.001440534
A0AAA9T5S5	EPB41L3	A0AAA9T5S5	ENSBTAG00000019251	0.07566427	0.081502785	0.005838515
G3N131	H1-1	bta:618164; G3N131	ENSBTAG00000059303	0.106499621	0.243839398	0.137339777
A0A3Q1LXP4	APOH	A0A3Q1LXP4	ENSBTAG00000001915	-0.256934206	0.163116612	0.420050818
A6QQX8	USP39	A6QQX8; bta:532985	ENSBTAG00000010691	-0.152471425	-0.007040985	0.14543044
A0AAA9TDE9	MLEC	A0AAA9TDE9	ENSBTAG00000020050	-0.001401355	0.110512451	0.111913806
Q3MHW6	SLC16A1	bta:505775; Q3MHW6	ENSBTAG00000015107	0.096806593	0.008436837	-0.088369756
A0AAF6DMI1	RBM45	A0AAF6DMI1	ENSBTAG00000006168	-0.204091605	-0.100412958	0.103678646
A0A3Q1LZ07	SDHB	A0A3Q1LZ07	ENSBTAG00000008314	0.108415667	0.149599102	0.041183435
A0A3Q1MSI2	NEMF	A0A3Q1MSI2	ENSBTAG00000004934	-0.136016975	-0.134531957	0.001485018
A0AAA9T405	RUFY1	A0AAA9T405; bta:528153	ENSBTAG00000019868	-0.077921473	-0.038434723	0.03948675
A0A3Q1LMC7	APC	A0A3Q1LMC7	ENSBTAG00000018852	-0.10207527	-0.292438019	-0.190362749
A0A3Q1LYY4	CRAT	A0A3Q1LYY4	ENSBTAG00000001932	-0.352706003	-0.236241202	0.116464801
F1MM88	NEK9	F1MM88	ENSBTAG00000016612	0.108059746	0.074962058	-0.033097688
A0AAA9TMJ0	WDR62	A0AAA9TMJ0	ENSBTAG00000058132	-0.14597268	-0.202492864	-0.056520184
F2Z4I5	ARL8B	F2Z4I5	ENSBTAG00000012902	-0.01247744	0.153089752	0.165567193
Q2TBV1	RFC3	bta:515602; Q2TBV1	ENSBTAG00000010787	0.117194099	0.106170587	-0.011023511
Q3T0B6	C1QBP	bta:518321; Q3T0B6	ENSBTAG00000012739	0.121379239	0.034262825	-0.087116414
A0AAA9TTA6	SFXN1	A0AAA9TTA6	ENSBTAG00000014536	0.103205282	0.152165185	0.048959903
Q1RMR2	SNRNP70	bta:535113; Q1RMR2	ENSBTAG00000048800	-0.002684085	-0.081358399	-0.078674314
F1MYW2	CAMSAP1	F1MYW2	ENSBTAG00000006008	0.036286206	0.030040763	-0.006245443
Q58DH8	FEN1	bta:616242; Q3MHF6; Q58DH8; Q58DS0	ENSBTAG00000000064	-0.187627003	-0.093265803	0.094361201
Q3SZM5	CMAS	bta:100336710; Q3SZM5	ENSBTAG00000010136	-0.140669479	-0.035059291	0.105610188
A0AAA9T1Q1	BLVRA	A0AAA9T1Q1	ENSBTAG00000013414	0.011119092	-0.033880893	-0.044999985
F1MS25	NDUFS3	F1MS25	ENSBTAG00000018483	-0.089716871	0.007436589	0.097153459
Q2HJ56	PWP1	bta:514147; Q2HJ56	ENSBTAG00000004654	-0.063086702	-0.005753527	0.057333175
Q3SYR7	RPL9	bta:282884; Q3SYR7; Q56K06	ENSBTAG00000014518	-0.098327114	-0.145075373	-0.046748259
A0AAA9S2E8	CALM1	A0AAA9S2E8	ENSBTAG00000025644	-0.023690106	0.453295214	0.47698532
E1B7Z4	LOC511531	E1B7Z4	ENSBTAG00000017670	-0.084618873	0.084605961	0.169224834
A0AAA9S913	SUCLG1	A0AAA9S913	ENSBTAG00000006075	-0.045466842	-0.019526623	0.025940218
A0AAF7AAJ5	BDH1	A0AAF7AAJ5	ENSBTAG00000000448	-0.053715637	-0.050678383	0.003037254
A0A3Q1MFL7	DSTN	A0A3Q1MFL7	ENSBTAG00000015434	-0.121071234	0	0.121071234
A0AAA9TUR4	ZZEF1	A0AAA9TUR4	ENSBTAG00000016316	-0.016678741	-0.072256089	-0.055577348
A0AAA9TW00	CIP2A	A0AAA9TW00	ENSBTAG00000017140	0.011450021	-0.057158422	-0.068608443
A0AAA9TPD6	ACSL1	A0AAA9TPD6	ENSBTAG00000004344	-0.161644851	-0.135249117	0.026395735
Q08DN8	FLOT1	bta:532573; Q08DN8	ENSBTAG00000009960	0.243557031	0.155481265	-0.088075767
Q3T0F4	RPS10	bta:540965; Q3T0F4	ENSBTAG00000015598	-0.08983141	-0.192825404	-0.102993993
A0A3Q1MPE4	TRNT1	A0A3Q1MPE4	ENSBTAG00000019534	0.076066658	0.127444017	0.051377359
A0A3Q1MUH0	MIA2	A0A3Q1MUH0	ENSBTAG00000006819	0.124738969	0.140830973	0.016092003
E1BJL3	BACH1	bta:531350; E1BJL3	ENSBTAG00000004139	-0.613126031	-0.453580176	0.159545856
A0AAA9SSZ3	ERLEC1	A0AAA9SSZ3	ENSBTAG00000008068	-0.074604936	-0.036166138	0.038438798
A0AAA9SV13	BRD2	A0AAA9SV13	ENSBTAG00000011074	0.251245208	-0.106576556	-0.357821764
A0A0K1L4Y9				-0.006308252	-0.108465447	-0.102157195
A0AAA9S956	PPP1R8	A0AAA9S956	ENSBTAG00000003866	0.017073513	-0.103835811	-0.120909324
A0A3Q1N9R6	RFC5	A0A3Q1N9R6	ENSBTAG00000007137	-0.20316946	-0.013408043	0.189761417

Accession	Gene_Symbol	Gene_ID	Ensembl_Gene ID	log2(ratio(CM12H/CM0H))	log2(ratio(CM12R/CM0H))	log2(ratio(CM12H/CM12R))
Q9N0F3	SARS2	A5D7G7; bta:282060; Q58CP7; Q58DN2; Q9N0F3		-0.049800747	-0.05402946	-0.004228713
A0AAA9SZA4	DNAJC8	A0AAA9SZA4	ENSBTAG00000018385	0.088355874	0.113840846	0.025484972
F1N2P6	FKBP8	F1N2P6	ENSBTAG00000016264	-0.083077888	0.064325327	0.147403215
P38408	GNA14	bta:281789; P38408		-0.222933263	-0.035029218	0.187904045
Q3ZCK9	PSMA4	bta:510423; Q3ZCK9	ENSBTAG00000014440	0.053222857	0.102089974	0.048867118
A0A3Q1MPB6	RANBP9	A0A3Q1MPB6	ENSBTAG00000021556	-0.100889681	-0.121353784	-0.020464103
F1MSB5	GORASP2	bta:513771; F1MSB5	ENSBTAG00000013218	0.148331909	0.169747864	0.021415955
A0AAA9TTJ9	YME1L1	A0AAA9TTJ9	ENSBTAG00000016445	-0.153030079	-0.139881375	0.013148704
Q08E52	PAK1	bta:533729; Q08E52	ENSBTAG00000010191	0.130032274	0.096691199	-0.033341075
E1BCI8	SLC12A4	E1BCI8	ENSBTAG00000018045	-0.128104826	-0.126633439	0.001471387
A0AAF6ZWM0	AASS	A0AAF6ZWM0	ENSBTAG00000012128	-0.097297201	0.016335373	0.113632574
A0A3Q1M6K7	PFKM	A0A3Q1M6K7	ENSBTAG00000000286	-0.092134281	-0.00733451	0.084799771
A0AAA9TM22	MASTL	A0AAA9TM22	ENSBTAG00000017271	0.100852602	0.198807817	0.097955215
E1BM34	ANAPC7	bta:535072; E1BM34	ENSBTAG00000016675	-0.086299846	-0.129283017	-0.042983171
A0AAF6YQE1	ASRGL1	A0AAF6YQE1	ENSBTAG00000006910	0.137985037	0.153944216	0.015959179
Q3SZC1	GRPEL1	bta:515580; Q3SZC1; Q9T2U7	ENSBTAG00000010181	0.109559925	-0.071912241	-0.181472166
Q5EAD0	BAIAP2	bta:507837; Q5EAD0	ENSBTAG00000019044	0.166067922	0.12508636	-0.040981562
Q3T0W4	PPP1R7	bta:505297; Q3T0W4	ENSBTAG00000000789	0.060564442	0.073331738	0.012767296
A0A3Q1LXS9	WDR70	A0A3Q1LXS9	ENSBTAG00000033095	-0.120113131	-0.108090603	0.012022528
Q3MI06	DNM2	Q3MI06		0.02713613	0.170601212	0.143465082
A0AAA9SKW7	GOLM1	A0AAA9SKW7	ENSBTAG00000031569	0.11146043	-0.247474932	-0.358935362
A0AAA9TKY6	THOC1	A0AAA9TKY6	ENSBTAG00000019215	0.060325645	-0.014398274	-0.074723918
F1N4H9	FUT8	F1N4H9	ENSBTAG00000019819	0.021177555	0.014592902	-0.006584653
P80513	MANF	A6QPB9; bta:540432; P80513		0.281631625	0.015751666	-0.265879959
F1MJ58	ECT2	F1MJ58	ENSBTAG00000023814	0.064488726	0.015035328	-0.049453398
E1BHR5	HEATR6	E1BHR5	ENSBTAG00000009635	-0.129698387	-0.082027914	0.047670473
E1BNM6	WBP11	bta:534416; E1BNM6	ENSBTAG00000014759	0.022991643	0.044218534	0.021226892
E1BLI8	MTCL2	E1BLI8	ENSBTAG00000021669	-0.018745926	-0.12566113	-0.106915204
E1BDV5	PELP1	bta:787413; E1BDV5	ENSBTAG00000018315	-0.262020565	-0.199543595	0.06247697
A0A3Q1MW62				-0.054588267	-0.249221649	-0.194633382
P62833	RAP1A	A4IFG1; bta:282031; P10113; P62833	ENSBTAG00000050904	0.301313065	0.27835908	-0.022953985
F1N1K2	TRIP4	F1N1K2	ENSBTAG00000014493	-0.042010467	-0.044677187	-0.00266672
E1BC36	CDK6	bta:511754; E1BC36	ENSBTAG00000044023	-0.101842141	-0.090346502	0.011495639
F1MT69	VPS36	F1MT69	ENSBTAG00000004307	-0.22569284	-0.163449283	0.062243557
A0A3Q1N590	MAP3K20	A0A3Q1N590	ENSBTAG00000001083	-0.006827722	-0.033071822	-0.026244099
F6QK60	SIRT2	F6QK60	ENSBTAG00000001776	0.080825524	0.042661152	-0.038164371
F1MNI4	RAB5B	F1MNI4	ENSBTAG00000014129	0.157775919	0.162343822	0.004567904
A0AAA9TXP4	MCM3AP	A0AAA9TXP4	ENSBTAG00000003148	-0.05047424	-0.075642467	-0.025168228
A0AAA9S5Q9	FHL1	A0AAA9S5Q9	ENSBTAG00000018513	-0.114913775	-0.048132496	0.066781279
E1BAD8	MID1	E1BAD8	ENSBTAG00000010152	0.101849254	0.048597873	-0.053251381
F1MXS4	POLA2	F1MXS4	ENSBTAG00000011455	-0.005559525	0.0138058	0.019365325
A0AAA9S6M2	CHCHD3	A0AAA9S6M2	ENSBTAG00000024723	0.050874048	0.024247546	-0.026626502
A0AAF7AP21	GNAS	A0AAF7AP21	ENSBTAG00000052413	-0.21400607	-0.112635198	0.101370872
A0AAF6YHC2	CYP20A1	A0AAF6YHC2	ENSBTAG00000000851	-0.051428239	-0.028344626	0.023083613
A0AAF6ZIP1	DDX27	A0AAF6ZIP1	ENSBTAG00000006671	0.017454876	-0.176525413	-0.193980289
A2VE51	BAG3	A2VE51; bta:782633		1.23691023	0.754729874	-0.482180356
Q3T0T7	SAR1B	bta:515999; Q3T0T7	ENSBTAG00000021226	-0.033630778	-0.100485753	-0.066854975
F2Z4E0	VGF	F2Z4E0	ENSBTAG00000009868	-0.278571726	-0.915474442	-0.636902716
A0A452DIQ6	ETFDH	A0A452DIQ6	ENSBTAG00000016679	0.06809998	0.050251979	-0.017848001
F1MC71	EXOC5	F1MC71	ENSBTAG00000013042	-0.082546437	-0.068957372	0.013589065
Q2KJ14	PAF1	bta:528744; Q2KJ14	ENSBTAG00000020428	0.200868709	0.100512778	-0.10035593
A0AAA9TRE2	VPS35L	A0AAA9TRE2	ENSBTAG00000019412	-0.303577069	-0.072781317	0.230795752
Q58DV0	PELO	bta:528728; Q1JPI6; Q58DV0		0.057863906	0.143957317	0.086093411
E1BE83	FANCD2	E1BE83	ENSBTAG00000010077	-0.070850326	-0.058797666	0.01205266
A0A3Q1M8V3	RASAL2	A0A3Q1M8V3	ENSBTAG00000015537	0.023438913	-0.124626655	-0.148065567
A0AAF6YU11	PSMA6	A0AAF6YU11	ENSBTAG00000009683	0.074995658	0.101215848	0.02622019
A2VDS0	PRPSAP2	A2VDS0; bta:506111	ENSBTAG00000014858	0.063792699	0.123458788	0.059666089
P62871	GNB1	bta:281201; P04697; P04901; P62871; Q3MHF1	ENSBTAG00000000215	0.174996606	0.228461257	0.053464652
Q9MZ08	BCAM	bta:282862; Q9MZ08	ENSBTAG00000009495	0.204514931	0.130703692	-0.073811239
Q3MHY1	CSRP1	bta:615329; Q3MHY1	ENSBTAG00000016057	-0.15575767	-0.024104557	0.131653113
A0A3Q1MUY5	IFI16	A0A3Q1MUY5; bta:506759	ENSBTAG00000011511	-0.043113106	0.037587368	0.080700474
E1BF14	POP1	bta:511306; E1BF14	ENSBTAG00000012596	-0.117152627	-0.101258981	0.015893646
A3KMY8	GUSB	A3KMY8; bta:515687	ENSBTAG00000000704	0.091489844	0.173475316	0.081985473
A0A3Q1M6Y9	ARHGAP12	A0A3Q1M6Y9	ENSBTAG00000012677	0.051244279	0.006505964	-0.044738315
E1BEW3	IPO11	E1BEW3	ENSBTAG00000018616	-0.039640728	0.039892727	0.079533455
A0AAA9S5G9	RECQL	A0AAA9S5G9	ENSBTAG00000021083	0.157852169	0.111309583	-0.046542586
A0A3Q1LVU1	FTH1	A0A3Q1LVU1	ENSBTAG00000011184	-0.138636975	-0.221343722	-0.082706747
E1B738	SHCBP1	bta:523367; E1B738	ENSBTAG00000033441	-0.046307805	0.069893215	0.116201021
A5PJM5	MCMBP	A5PJM5; bta:514863		0.243785882	0.028014376	-0.215771505
F1N2B8	PUM2	F1N2B8	ENSBTAG00000011155	0.021513093	-0.048209093	-0.069722186
F1MMH3	LUC7L	F1MMH3	ENSBTAG00000016568	0.286364234	0.136001499	-0.150362735
E1BEA2	EDC3	E1BEA2	ENSBTAG00000007308	0.105426354	0.093272154	-0.0121542
O62830	PPM1B	bta:281995; O62830; Q2T9W5	ENSBTAG00000000223	0.044854236	0.13058411	0.085729874
Q1JP79	ARPC1A	A6QLF5; bta:508402; Q1JP79	ENSBTAG00000004242	0.134261511	0.194647431	0.06038592
Q0II26	AIMP2	bta:520979; Q0II26	ENSBTAG00000007367	0.097555309	0.116846648	0.019291339
Q32LN7	SKIC8	bta:513208; Q32LN7		-0.027376439	-0.053874241	-0.026497802
Q0VCC3	BLOC1S4	bta:518544; Q0VCC3	ENSBTAG00000003766	-0.690297535	-0.865013955	-0.174716421
E1BJE7	MCM3AP	E1BJE7	ENSBTAG00000003148	0.029659974	-0.015782997	-0.045442971
E1B8X4	NAE1	E1B8X4	ENSBTAG00000010132	-0.16319768	-0.116209205	0.046988475
A0AAA9SEL9	TRANK1	A0AAA9SEL9	ENSBTAG00000021452	0.017756471	0.259556235	0.241799764
F1MJQ1	RAB7A	F1MJQ1	ENSBTAG00000010193	0.130161548	0.083926082	-0.046235466
A7MBI6	GLOD4	A7MBI6; bta:512334; E1BBM1		0.11179781	0.095495997	-0.016301812
A0A3Q1MKW0	VIRMA	A0A3Q1MKW0	ENSBTAG00000003789	-0.183742822	-0.173837079	0.009905743
A0AAA9SWL8	SERPINB8	A0AAA9SWL8	ENSBTAG00000001207	-0.14483402	-0.120171966	0.024662054
A7MB32	STK38L	A7MB32; bta:514787	ENSBTAG00000011798	0.012727071	0.052010799	0.039283728
A0A3Q1LKW6	MPDZ	A0A3Q1LKW6	ENSBTAG00000043961	-0.103835811	-0.155505942	-0.051670131
Q0P5H6	TBL2	bta:511758; F1MET7; Q0P5H6	ENSBTAG00000000321	0.055469089	0.044812013	-0.010657076
A0A3Q1MLS9	L1CAM	A0A3Q1MLS9	ENSBTAG00000013462	-0.027300721	-0.514424587	-0.487123866
Q32LE9	CSRP2	bta:539381; Q32LE9		0.010686679	0.078284757	0.067598078

Accession	Gene_Symbol	Gene_ID	Ensembl_Gene ID	log2(ratio(CM12H/CM0H))	log2(ratio(CM12R/CM0H))	log2(ratio(CM12H/CM12R))
A6QP40	LRRFIP1	A6QP40; bta:616994		0.08061846	0.08061846	0
E1BAX1	GOPC	bta:541186; E1BAX1	ENSBTAG00000016046	0.102168329	0.014047774	-0.088120555
A0AAA9SWP3	ACOT7	A0AAA9SWP3	ENSBTAG00000021202	0.134001835	0.028569152	-0.105432683
Q32KY4	CDK4	bta:510618; Q32KY4	ENSBTAG00000007160	0.069239083	0.034298242	-0.034940841
A0AAA9SHQ2	NAA35	A0AAA9SHQ2	ENSBTAG00000001016	0.034440645	0.062284278	0.027843633
E1BIW0	UBR1	bta:522464; E1BIW0	ENSBTAG00000021087	-0.128290513	-0.137792381	-0.009501868
F1MLD0	PANK4	F1MLD0	ENSBTAG00000002479	-0.234964123	-0.249290905	-0.014326781
Q3SYS6	CHP1	bta:512662; Q3SYS6	ENSBTAG00000027173	0.137122581	-0.001397283	-0.138519864
F1MWF0	HIP1	F1MWF0	ENSBTAG00000000781	-0.039438311	-0.034449159	0.004989152
F1N1A2	DHX57	F1N1A2	ENSBTAG00000014679	-0.170444798	-0.190864339	-0.020419541
A0A3Q1NHI6	FAM114A1	A0A3Q1NHI6	ENSBTAG00000033160	-0.181621566	-0.162530805	0.019090761
A0A3Q1MVR5	EHMT1	A0A3Q1MVR5	ENSBTAG00000012148	-0.232534432	-0.239974856	-0.007440424
Q29RK4	RAD23B	bta:530189; Q29RK4	ENSBTAG00000001926	-0.009035698	-0.013574857	-0.00453916
E1BEG4	ZFYVE16	E1BEG4	ENSBTAG00000021577	-0.057997724	-0.070850326	-0.012852602
A0AAF6DM61	VASN	A0AAF6DM61	ENSBTAG00000046339	0.336147916	0.151394875	-0.184753041
Q3SZ18	HPRT1	bta:281229; Q3SZ18	ENSBTAG00000014685	-0.053806444	-0.085900073	-0.03209363
A0AAA9S9P9	EP300	A0AAA9S9P9	ENSBTAG00000016198	-0.152147645	-0.094063711	0.058083934
F1MXZ0	GNS	F1MXZ0	ENSBTAG00000017465	0.209756266	0.300960665	0.091204399
Q9N181	NMT2	bta:282049; Q9N181		-0.170334916	-0.170334916	0
A0AAA9TM77	MRI1	A0AAA9TM77	ENSBTAG00000002524	0.009439696	-0.206693735	-0.216133431
F1MNI5	PTGS2	F1MNI5	ENSBTAG00000014127	0.263034406	0.357010554	0.093976148
A0AAF7APW4	ILVBL	A0AAF7APW4	ENSBTAG00000015186	-0.063792699	0.096263243	0.160055942
A5PKD6	GNB4	A5PKD6; bta:525962		0.006238691	0.121356716	0.115118025
A4FV84	MRTO4	A4FV84; bta:509932	ENSBTAG00000011951	-0.139505386	-0.273901327	-0.13439594
A0AAA9TWE2	CDK13	A0AAA9TWE2	ENSBTAG00000001528	0.1008175	-0.106915204	-0.207732703
A0A3Q1M9W0	NDRG1	A0A3Q1M9W0	ENSBTAG00000000711	0.087872756	0.148234624	0.060361868
Q3ZCA7	GNAI3	bta:539521; Q3ZCA7		0.029264595	0.106809616	0.077545022
G8JKZ0	DHODH	G8JKZ0	ENSBTAG00000019887	0.049314937	-0.024531006	-0.073845943
G3N093	CDCP1	bta:540622; G3N093	ENSBTAG00000012482	-0.252529969	-0.023777383	0.228752586
Q2TBV3	ETFB	bta:617210; Q2TBV3		0.056634017	0.061674005	0.005039988
A0AAF7ANF9	ANP32B	A0AAF7ANF9	ENSBTAG00000021367	0.080208131	0.295973554	0.215765423
A0A3Q1LXG3	CAPZA2	A0A3Q1LXG3	ENSBTAG00000004072	0.120612674	0.045455315	-0.07515736
A0AAA9TAD0	MRPS9	A0AAA9TAD0	ENSBTAG00000006150	-0.089784926	-0.064130337	0.025654589
A0AAF7A1X1	HSD3B1	A0AAF7A1X1	ENSBTAG00000006769	0.363392737	0.518271445	0.154878707
Q0VCW8	PDCL3	bta:514033; Q0VCW8		0.124912946	-0.159767086	-0.284680032
A0A3Q1LVA2	BCAT1	A0A3Q1LVA2	ENSBTAG00000013825	-0.043068722	-0.038350173	0.004718549
Q5E951	TBCB	bta:530447; Q3ZCC1; Q5E951		0.033409422	0.062660055	0.029250633
A0A3Q1LX22	PCM1	A0A3Q1LX22; bta:525337	ENSBTAG00000007236	-0.393723995	-0.411293332	-0.017569337
A0A3Q1N0U6	ANAPC1	A0A3Q1N0U6; bta:535565	ENSBTAG00000027991	-0.14577114	-0.176497655	-0.030726516
A0AAF6YHD2	ERAP2	A0AAF6YHD2	ENSBTAG00000039275	-0.275953785	0.002424698	0.278378484
A2VE08	KPNA1	A2VE08; bta:539679	ENSBTAG00000011143	0.179533775	0.243042931	0.063509155
A0A452DI44	PSMD4	A0A452DI44	ENSBTAG00000006101	-0.059917724	-0.027559483	0.032358242
A0A3Q1MMQ9	SUB1	A0A3Q1MMQ9	ENSBTAG00000021965	0.034535827	0.111299123	0.076763296
A0AAA9TWW9	SFXN3	A0AAA9TWW9	ENSBTAG00000005015	-0.067314112	-0.086501132	-0.01918702
Q1RMM9	NAGA	Q1RMM9		0.019677485	0.129128627	0.109451142
E1BNI8	NT5DC2	bta:514296; E1BNI8	ENSBTAG00000014784	-0.152003093	-0.050080619	0.101922474
F1MZS1	WWC2	F1MZS1	ENSBTAG00000012191	0.011416044	0.007145607	-0.004270437
E1BDW9	EXOC2	bta:513431; E1BDW9	ENSBTAG00000020215	-0.238462363	0.010289259	0.248751623
A0A3Q1NF64	SERPINB5	A0A3Q1NF64	ENSBTAG00000002019	-0.181511398	-0.155135983	0.026375415
F1N226	APLP2	bta:785005; F1N226	ENSBTAG00000016168	0.045642666	-0.214399631	-0.260042296
A0AAA9T2N9	EPM2AIP1	A0AAA9T2N9; bta:540067	ENSBTAG00000055342	-0.296471365	-0.073295509	0.223175856
A6QLP2	AHCYL2	A6QLP2; bta:532836		0.397120767	0.233471091	-0.163649676
F1N1B8	KANK1	bta:534869; F1N1B8	ENSBTAG00000010976	-0.10003783	-0.094398624	0.005639206
F6RNH4	PPP1R13L	F6RNH4	ENSBTAG00000020689	-0.097141158	-0.139014111	-0.041872953
A0AAA9SSL4	NAA50	A0AAA9SSL4	ENSBTAG00000007784	0.025045129	0.007956059	-0.01708907
A0AAF6YVY1	CTSH	A0AAF6YVY1	ENSBTAG00000010992	-0.129577715	-0.034075629	0.095502087
Q17QE2	LMCD1	bta:540474; Q17QE2	ENSBTAG00000005431	-0.077679364	-0.263499866	-0.185820503
E1BMF2	ZMPSTE24	E1BMF2	ENSBTAG00000044178	0.045194902	0.155476957	0.110282055
A4IFN6	SNX18	A4IFN6; bta:538366	ENSBTAG00000027924	0.021020698	0.152567639	0.131546941
G5E5A1	CAMSAP2	G5E5A1	ENSBTAG00000008153	0.074224585	-0.010492374	-0.084716959
Q3SWX2	ACOT9	bta:527845; Q3SWX2	ENSBTAG00000011258	-0.256556195	-0.186905764	0.069650432
A0A452DIL6	GPC1	A0A452DIL6	ENSBTAG00000015996	0.104163408	-0.196552416	-0.300715825
A4IFK3	LMOD1	A4IFK3; bta:532090	ENSBTAG00000021576	-0.372762121	-0.409735716	-0.036973596
P05131	PRKACB	bta:282323; P05131; P24256		-0.009056496	-0.031026896	-0.0219704
A0A3Q1LPH8	TAOK3	A0A3Q1LPH8; bta:534620	ENSBTAG00000012550	0.053687871	0.053687871	0
F2Z4J7	CBX5	bta:538885; F2Z4J7	ENSBTAG00000006246	0.242499257	0.12154691	-0.120952347
Q3T0Y1	OTUB1	bta:540984; Q3T0Y1	ENSBTAG00000003967	-0.071240727	-0.049056358	0.022184369
F1N2W0	PTGR1	F1N2W0	ENSBTAG00000011027	-0.038645928	0.119870473	0.1585164
Q3T108	PSMB4	bta:506203; Q3T108	ENSBTAG00000021288	-0.033915409	-0.016179927	0.017735482
A6H772	PPP4C	A6H772; bta:540398	ENSBTAG00000012928	-0.178720432	-0.097174083	0.081546348
A0AAA9TD90	COA7	A0AAA9TD90; bta:520518	ENSBTAG00000062331	0.22086171	0.146792036	-0.074069674
F1MWR3	ETFA	F1MWR3	ENSBTAG00000012490	0.208612624	0.16224266	-0.046369963
A0AAF6YJV2	GNAI1	A0AAF6YJV2	ENSBTAG00000002714	0.056260573	0.077634224	0.021373651
E1BG63	TMTC3	bta:522132; E1BG63	ENSBTAG00000009023	0.026995082	-0.006068127	-0.033063208
A0A452DIP2	GALE	A0A452DIP2	ENSBTAG00000005002	0.016535368	0.015164636	-0.001370732
A0AAA9RS28	DOCK9	A0AAA9RS28	ENSBTAG00000010395	-0.008551863	-0.043275441	-0.034723578
G3X6U1	ALDH1A2	bta:535075; G3X6U1	ENSBTAG00000010119	-0.264389685	-0.316857105	-0.05246742
A0AAF6Z178	RPS8	A0AAF6Z178	ENSBTAG00000015285	-0.158394423	-0.087381317	0.071013106
P13620	ATP5PD	bta:282710; P13620; Q3SX10	ENSBTAG00000021227	0.051174844	0.041365321	-0.009809523
F1N3Z8	PPP4R2	bta:504900; F1N3Z8	ENSBTAG00000002147	-0.151461643	-0.05210643	0.099355213
F1MMK0	COMT	F1MMK0	ENSBTAG00000019516	0.243241679	0.273254248	0.030012569
E1BPQ8	CDC73	bta:100139547; E1BPQ8	ENSBTAG00000016703	0.08246216	0.051190699	-0.031271462
A0AAA9T466	CORO7	A0AAA9T466	ENSBTAG00000009201	-0.100643077	-0.134266729	-0.033623652
F6RNL0	NOP14	F6RNL0	ENSBTAG00000001504	-0.104920602	-0.116126057	-0.011205455
E1BCS1	CNOT3	E1BCS1	ENSBTAG00000014900	-0.063110044	-0.036957756	0.026152288
Q08E42	LRRC8A	bta:505605; Q08E42	ENSBTAG00000013030	-0.162075902	-0.062948693	0.099127209
A0AAF6DMG5	SCYL2	A0AAF6DMG5	ENSBTAG00000013859	0.034051849	0.092828478	0.05877663
P61585	RHOA	bta:338049; P06749; P61585; Q3ZC72; Q9UEJ4	ENSBTAG00000004279	-0.070115598	-0.004112198	0.0660034
A0AAA9SA64	MESD	A0AAA9SA64	ENSBTAG00000001565	0.052889693	0.106351981	0.053462288
Q08DY0	ERICH5	bta:504339; Q08DY0	ENSBTAG00000018493	-0.027430972	0.184372814	0.211803786

Accession	Gene_Symbol	Gene_ID	Ensembl_Gene ID	log2(ratio(CM12H/CM0H))	log2(ratio(CM12R/CM0H))	log2(ratio(CM12H/CM12R))
A0AAA9TBT5	ACADM	A0AAA9TBT5	ENSBTAG00000024240	0.007174033	-0.023199616	-0.030373649
Q3MHL3	RBBP4	A5D9B1; bta:767940; Q3MHL3		0.189033824	0.088355874	-0.10067795
Q29RQ2	KHDRBS1	bta:538775; Q29RQ2	ENSBTAG00000012497	-0.106284793	-0.096026888	0.010257905
Q32KW2	UBXN1	bta:506676; Q32KW2	ENSBTAG00000010481	-0.105169515	-0.11497269	-0.009803175
P81623	ERP29	bta:613357; P81623; Q17QC3	ENSBTAG00000006665	0.145788096	0.109942897	-0.035845199
Q32LF9	CAMLG	bta:615658; F1MX41; Q32LF9		0.158893964	0.098593454	-0.06030051
A0A3Q1LR80	LARP4B	A0A3Q1LR80	ENSBTAG00000013899	0.083283133	0	-0.083283133
A0AAF6YR53				0.180207975	0.084181087	-0.096026888
A0AAA9THW9	PIK3C3	A0AAA9THW9	ENSBTAG00000026290	-0.060435854	0.014721506	0.07515736
B1VKB3	C/EBP-delta	B1VKB3		0.726181918	-0.060066569	-0.786248487
A6QPN5	MMP2	A6QPN5		0.074101962	-0.516167231	-0.590269193
A0AAF6DMH7	RIPK1	A0AAF6DMH7	ENSBTAG00000006378	-0.02147153	0.087462841	0.108934372
A0A3Q1MK17	MTA1	A0A3Q1MK17	ENSBTAG00000047606	-0.213244039	-0.262300397	-0.049056358
A4IF89	EXOC8	A4IF89; bta:540237	ENSBTAG00000006111	0.022390957	-0.004520197	-0.026911154
Q5E9A4	RAE1	bta:513080; Q5E9A4	ENSBTAG00000010934	-0.014706499	0.020340448	0.035046947
A0A3Q1LU41	OSBPL8	A0A3Q1LU41	ENSBTAG00000004427	-0.01603821	0.139479368	0.155517578
O62830-2	PPM1B	O62830-2		-0.119415167	-0.156504486	-0.037089319
A0AAA9U062	PEPD	A0AAA9U062	ENSBTAG00000031941	-0.16312259	-0.081245583	0.081877007
A0A3Q1M4Q5	PCYT1A	A0A3Q1M4Q5	ENSBTAG00000016979	-0.171157547	-0.095493278	0.07566427
Q0IIF2	EIF2B1	bta:516670; Q0IIF2		-0.083484986	0.069285295	0.15277028
A0AAF6YZF4	RPS11	A0AAF6YZF4	ENSBTAG00000013924	-0.251289198	-0.138814469	0.112474729
A7YY75	SEH1L	A7YY75; bta:506509	ENSBTAG00000010792	0.028105338	0.015681622	-0.012423715
A0AAA9SQQ3	LAS1L	A0AAA9SQQ3	ENSBTAG00000002210	-0.079108958	-0.121795357	-0.042686399
A0A3Q1MQL4	TUT7	A0A3Q1MQL4	ENSBTAG00000001737	-0.189318765	-0.201180662	-0.011861897
A0AAA9SZ86	SNX27	A0AAA9SZ86	ENSBTAG00000001942	0.10150612	0.145250356	0.043744236
A0AAA9TD15	ENSA	A0AAA9TD15	ENSBTAG00000007123	0.173304245	-0.234038862	-0.407343107
A0AAA9SWA2	GNAQ	A0AAA9SWA2; bta:536654	ENSBTAG00000009789	0.162237284	0.196251317	0.034014032
F6R1L1	PPP4R3A	F6R1L1	ENSBTAG00000005040	0.081986617	-0.011045784	-0.093032401
P52556	BLVRB	bta:281650; P52556; Q3T0T4		0.106494102	0.200544237	0.094050134
A0AAA9TRU0	DEK	A0AAA9TRU0	ENSBTAG00000012865	0.046675461	-0.038172502	-0.084847964
Q3MHE9	JSP.1	bta:407173; Q3MHE9		0.109823278	0.021573412	-0.088249866
A0AAA9TAT3	UBFD1	A0AAA9TAT3	ENSBTAG00000046033	0.115600657	0.149272389	0.033671732
F1MU05	PALS2	F1MU05	ENSBTAG00000015303	0.115053581	0.107406769	-0.007646811
A0AAA9TQV8	THUMPD3	A0AAA9TQV8	ENSBTAG00000003112	0.136543875	0.094804366	-0.041739508
A3KMZ0	STXBP3	A3KMZ0; bta:512490		-0.088718216	0.028400426	0.117118642
Q3SX44	DDAH2	bta:540386; Q3SX44	ENSBTAG00000013530	0.00732706	0.085560348	0.078233288
A0A3Q1LY00	PALS1	A0A3Q1LY00; bta:528109	ENSBTAG00000011767	0.195256291	0.082711051	-0.11254524
A5PKJ4	MAPK7	A5PKJ4; bta:537703		-0.018109381	-0.003002488	0.015106892
A0AAA9TUK9	DDOST	A0AAA9TUK9	ENSBTAG00000006085	-0.350846441	-0.061339824	0.289506617
A0A3Q1LVL2	HMOX1	A0A3Q1LVL2	ENSBTAG00000015582	0.150917952	0.094464684	-0.056453268
Q2KIS4	DHRS1	bta:528832; Q2KIS4	ENSBTAG00000010234	-0.288333814	-0.302914797	-0.014580984
A0AAA9ST05	TBK1	A0AAA9ST05	ENSBTAG00000017401	-0.191141487	-0.117484446	0.073657042
F1MQD1	CAPN5	F1MQD1	ENSBTAG00000005034	-0.115949381	0.100290814	0.216240194
Q9BGI1	PRDX5	bta:282885; Q3SZ78; Q9BGI1	ENSBTAG00000008648	0.119275148	0.223034332	0.103759184
E1BNQ2	DHX37	E1BNQ2	ENSBTAG00000020414	-0.219744611	-0.141122023	0.078622588
A2VE47	ADPGK	A2VE47; bta:518158	ENSBTAG00000001578	0.034796476	-0.053143947	-0.087940423
E1BGG6	RFX5	E1BGG6	ENSBTAG00000017580	-0.138476768	0.023436555	0.161913323
A0AAA9SH86	TAF15	A0AAA9SH86	ENSBTAG00000006916	-0.07287888	0.012735988	0.085614868
Q3T025	RPL17	bta:514046; Q3T025	ENSBTAG00000022902; ENSBTAG00000060385	-0.085620426	-0.071462363	0.014158063
A0A3Q1MF00	PRIM1	A0A3Q1MF00; bta:506240	ENSBTAG00000044000	-0.424595934	-0.318514899	0.106081035
A0AAF6YKI3	DNAJB11	A0AAF6YKI3	ENSBTAG00000003151	0.24948298	-0.007802588	-0.257285568
A0A3Q1MHB0	EGFR	A0A3Q1MHB0	ENSBTAG00000011628	-0.080090853	-0.158665392	-0.07857454
A0AAA9T950	MAP2K4	A0AAA9T950	ENSBTAG00000012103	0.022229947	0.109260183	0.087030236
A5PJI5	GET3	A5PJI5; bta:504586		0.088614387	0.068522969	-0.020091418
Q5E9R9	ASCC2	bta:505503; Q5E9R9		-0.256887421	-0.190710345	0.066177077
P02081				-0.501053495	-0.622473296	-0.1214198
E1B9S7	OTUD7B	E1B9S7	ENSBTAG00000001586	-0.028992203	-0.00453916	0.024453044
A0AAA9SFU7	PPP1R18	A0AAA9SFU7	ENSBTAG00000039695	-0.016582884	-0.080523705	-0.063940821
Q3T0C8	PDLIM2	bta:512907; Q3T0C8	ENSBTAG00000007369	0	0	0
F1MYN1	PLEKHA5	F1MYN1	ENSBTAG00000017571	0.065006131	-0.016210227	-0.081216358
A0A3Q1LT51	TAB1	A0A3Q1LT51	ENSBTAG00000019828	0.030282567	-0.01759406	-0.047876627
A0A452DKB6	COG7	A0A452DKB6	ENSBTAG00000032223	0.008120256	0.088181176	0.08006092
A0A452DIL1	PCNP	A0A452DIL1	ENSBTAG00000015069	0.271568792	0.229710696	-0.041858096
A0AAA9TPL7	PRPF31	A0AAA9TPL7	ENSBTAG00000007757	-0.067757596	-0.118181426	-0.050423831
O97902	ASAP1	bta:327705; O97902		0.224330877	0.168037892	-0.056292985
F1MQ89	COG2	bta:512213; F1MQ89	ENSBTAG00000020314	0.045043894	-0.070317838	-0.115361733
E1BI41	GIT2	bta:516048; E1BI41	ENSBTAG00000006506	0.00307611	0.14490305	0.14182694
A0AAA9S835	ERAP1	A0AAA9S835	ENSBTAG00000013557	-0.189695384	-0.098355072	0.091340312
A0AAA9RYG1	DSG2	A0AAA9RYG1	ENSBTAG00000021923	-0.182426014	-0.374695732	-0.192269718
E1BK49	RPRD2	E1BK49	ENSBTAG00000014984	0.140985541	0.051947828	-0.089037713
A0AAA9TTI7	MCU	A0AAA9TTI7	ENSBTAG00000003876	0.038930993	0.22869241	0.189761417
A0A173FDL2	BoLA	A0A173FDL2		-0.026702794	0.093401182	0.120103976
E1BJQ7	GIPC1	bta:519617; E1BJQ7	ENSBTAG00000000764	0.123489428	0.093211401	-0.030278027
G3X6L5	ARL8A	G3X6L5	ENSBTAG00000006574	-0.0694851	0.001506731	0.070991832
A0AAF6ZNP5	THUMPD1	A0AAF6ZNP5	ENSBTAG00000047673	0.114894852	0.103667596	-0.011227255
E1BGA6	COG3	E1BGA6	ENSBTAG00000017907	-0.038513654	0.055908741	0.094422395
A0A3Q1LWP2	PPP1R12C	A0A3Q1LWP2	ENSBTAG00000017847	-0.05718983	-0.00912139	0.04806844
A0AAA9TUH8	WDR12	A0AAA9TUH8	ENSBTAG00000015424	-0.125530882	-0.255041615	-0.129510733
A0A3Q1MA84	WIZ	A0A3Q1MA84	ENSBTAG00000010450	-0.228268988	-0.187627003	0.040641984
A6H7C4	CBX3	A6H7C4; bta:539788	ENSBTAG00000049437	0.07058806	-0.124898648	-0.195486708
Q76I82	RPS15A	bta:337888; Q76I82	ENSBTAG00000020733	-0.109316694	-0.092589608	0.016727086
A0A3Q1LP21	TBCE	A0A3Q1LP21	ENSBTAG00000013735	-0.345321321	-0.209274329	0.136046992
F1N194	ZNF622	F1N194	ENSBTAG00000016441	0.084454939	-0.204328486	-0.288783426
A0AAF6ZFR0	CSNK2B	A0AAF6ZFR0	ENSBTAG00000008837	0.066391222	0.113919592	0.04752837
A0A3Q1MQJ9				-0.054839387	0.103795412	0.1586348
F1N053	CTBP2	F1N053	ENSBTAG00000003397	0.141608676	-0.007312205	-0.148920881
F1MLB2	CHD8	F1MLB2	ENSBTAG00000020422	0.111141027	-0.068243176	-0.179384203
A0AAA9S9H0	RAN	A0AAA9S9H0	ENSBTAG00000055033	-0.057450272	0.069082134	0.126532406
E1BBG4	RIMOC1	E1BBG4	ENSBTAG00000004929	0.161646352	0.199754618	0.038108265
Q5E971	TMED10	A4FV04; bta:529761; Q5E971	ENSBTAG00000005694	0.173387669	0.169429656	-0.003958014

Accession	Gene_Symbol	Gene_ID	Ensembl_Gene ID	log2(ratio(CM12H/CM0H))	log2(ratio(CM12R/CM0H))	log2(ratio(CM12H/CM12R))
A5D7G0	YARS2	A5D7G0; bta:536956	ENSBTAG00000011399	-0.105502874	-0.060602101	0.044900773
E1BG26	ATP13A3	A0A3Q1LMJ5; E1BG26	ENSBTAG00000013204	0.184486555	0.208825005	0.02433845
Q2TBH2	RRAS	bta:616503; Q2TBH2	ENSBTAG00000020983	0.131034028	0.160209847	0.029175818
E1BG07	CWF19L1	E1BG07	ENSBTAG00000007594	0.200897297	0.093879047	-0.10701825
Q3ZBR5	TTC1	bta:504602; Q3ZBR5	ENSBTAG00000010321	0.206136531	0.129193211	-0.07694332
Q2TBV6	CCDC124	bta:510778; Q2TBV6	ENSBTAG00000004604	0.279371381	0.083591812	-0.195779569
A0A3Q1MYR8	KRT3	A0A3Q1MYR8	ENSBTAG00000045662	-0.277533976	-0.341902795	-0.064368819
Q17QJ7	PYCR2	bta:504987; Q17QJ7	ENSBTAG00000005835	0.016906776	-0.001417882	-0.018324658
Q2KIV3	RBM4B	bta:505756; Q2KIV3	ENSBTAG00000000423	-0.113470933	-0.147600795	-0.034129861
Q1JP69	GNL2	Q1JP69		-0.323963404	-0.214504512	0.109458893
Q5E9N0	BYSL	B0JYL5; bta:514128; Q5E9N0	ENSBTAG00000010101	-0.006253564	-0.257553603	-0.251300039
Q3T0Q4	NME2	bta:615447; Q3T0Q4	ENSBTAG00000047186	0.096499348	0.044794331	-0.051705017
E1BA82	UBAP2	bta:506577; E1BA82	ENSBTAG00000000695	0.001308567	-0.098881242	-0.100189809
A0AAA9SLR3	RELA	A0AAA9SLR3	ENSBTAG00000013895	-0.113562559	0.00573637	0.119298928
A0A3Q1LKF0	CCDC91	A0A3Q1LKF0	ENSBTAG00000012448	0.078355638	-0.165110082	-0.24346572
A0AAA9S1I7	ECD	A0AAA9S1I7	ENSBTAG00000000315	0.144081529	-0.207148385	-0.351229914
A0A3Q1N8Z4	HNRNPH3	A0A3Q1N8Z4	ENSBTAG00000014316	0.108482045	0.068576634	-0.039905411
G3MXD0	IRF2BP2	G3MXD0	ENSBTAG00000046841	-0.086920227	0.023378939	0.110299166
F1MEP1	QNG1	F1MEP1	ENSBTAG00000006478	-0.106915204	-0.010007173	0.096908031
A0JN47	PLXDC2	A0A3Q1LUJ4; A0JN47; bta:515731	ENSBTAG00000009475	-0.028727178	-0.101455728	-0.07272855
F1MNV5	KNG1	F1MNV5	ENSBTAG00000005122	-0.498730103	-0.00698643	0.491743673
F6QGZ0	LIMS1	F6QGZ0	ENSBTAG00000010971	-0.010971118	0.099535674	0.110506791
F1MPT7	RRS1	F1MPT7	ENSBTAG00000016716	-0.127254385	-0.249027548	-0.121773163
E1BIM8	SRC	E1BIM8	ENSBTAG00000008938	-0.160659105	-0.05903141	0.101627696
F6PSE2	NBN	F6PSE2	ENSBTAG00000013225	0.053737871	-0.070115598	-0.123853469
E1BA27	GLMN	E1BA27	ENSBTAG00000018737	-0.045442971	-0.101334172	-0.055891201
P34943	NDUFA9	bta:404188; P34943; Q3T0K3	ENSBTAG00000005465	-0.034703469	-0.023042647	0.011660823
F1N3B9	TRIP13	bta:506746; F1N3B9	ENSBTAG00000006972	-0.151852018	0.042868745	0.194720763
A0A3Q1MG38	CAP2	A0A3Q1MG38	ENSBTAG00000016924	0.375211519	0.395714399	0.02050288
A0AAA9TD87	NDRG3	A0AAA9TD87	ENSBTAG00000019621	0.157598131	0.106444043	-0.051154088
Q0VC97	CBR3	bta:516036; Q0VC97	ENSBTAG00000018688	0.035433693	0.115357238	0.079923544
A0A3Q1M7C3	CASP8AP2	A0A3Q1M7C3	ENSBTAG00000011313	-0.601923108	-0.769650554	-0.167727446
F1MYK6	SCAF8	F1MYK6	ENSBTAG00000031917	-0.063708488	-0.111614873	-0.047906385
Q1LZF8	CLCC1	bta:519085; Q1LZF8	ENSBTAG00000017129	0.231541858	-0.005852726	-0.237394585
F1MT41	PTGFRN	bta:538209; F1MT41	ENSBTAG00000008022	0.395648514	0.241869796	-0.153778718
E1BDX9	FAM50A	bta:515539; E1BDX9	ENSBTAG00000033032	0.048236186	0.055195654	0.006959469
Q0VD29	RRAGC	bta:617367; E1BFI0; Q0VD29	ENSBTAG00000009368	0.033435912	0.171990689	0.138554777
A5D7U9	DCPS	A5D7U9; bta:282640		-0.002686584	-0.089981367	-0.087294783
A6H788	SNRPA1	A6H788; bta:512584	ENSBTAG00000013593	-0.160253932	-0.168283795	-0.008029863
Q5E9G3	PSME2	bta:509857; Q3T038; Q5E9G3; Q9TR87; Q9TR88; Q9TR89; Q9TR90	ENSBTAG00000005814	0.039605881	-0.007188331	-0.046794211
A0AAA9T2P6	CTBP1	A0AAA9T2P6	ENSBTAG00000008480	-0.022787365	-0.075414295	-0.052626931
A0A3Q1MR25	PDK3	A0A3Q1MR25	ENSBTAG00000017832	0.003922146	0.289625627	0.285703481
Q0V898	NELFE	bta:540158; Q0V898		-0.186779302	0.043146792	0.229926094
Q29RH4	THOC3	bta:529231; Q29RH4	ENSBTAG00000035174	0.138539943	-0.02154388	-0.160083823
A0AAA9ST33	EXOC6B	A0AAA9ST33	ENSBTAG00000020799	-0.035118043	0.010083781	0.045201824
Q3SZJ9	PMM2	bta:510978; Q3SZJ9	ENSBTAG00000001440	0.114046682	0.058836495	-0.055210187
F1MNN1	HAUS3	F1MNN1	ENSBTAG00000024787	-0.011162106	-0.05236447	-0.041202363
A0A3Q1LL63	AASDHPPT	A0A3Q1LL63; bta:519816	ENSBTAG00000012279	-0.180020048	-0.277067821	-0.097047773
A0AAA9SSM4	CEP55	A0AAA9SSM4	ENSBTAG00000005129	0.172508162	0	-0.172508162
E1BQ32	CRK	E1BQ32	ENSBTAG00000005665	0.130087519	0.111378992	-0.018708527
A0AAA9SMA6	SRPK1	A0AAA9SMA6	ENSBTAG00000016838	-0.124486208	-0.124486208	0
A0AAA9T3P5	RSF1	A0AAA9T3P5	ENSBTAG00000020268	0.11516977	-0.096399069	-0.211568839
Q32LM0	SH3GLB1	bta:533918; Q32LM0		-0.084375163	-0.183589845	-0.099214682
A0AAA9TZL3	NDUFV2	A0AAA9TZL3	ENSBTAG00000004871	-0.059421086	-0.050219355	0.009201731
A0A3Q1LX83				0.05415577	0.087926507	0.033770737
A0AAA9SIH1	TAPBP	A0AAA9SIH1	ENSBTAG00000016828	-0.065653239	-0.146741731	-0.081088492
A6QLW1	PIK3R4	A6QLW1; bta:507414	ENSBTAG00000020787	0.107383535	0.174290194	0.066906659
A0A3Q1MVQ2	PCID2	A0A3Q1MVQ2	ENSBTAG00000021725	0.02923962	-0.061941386	-0.091181007
Q17Q89	PFDN6	bta:509914; Q17Q89	ENSBTAG00000010723	0.069193442	-0.212825931	-0.282019373
F1N0C7	NTN1	F1N0C7	ENSBTAG00000012137	-0.238657476	-0.412882615	-0.174225139
A0A3Q1MP16	AQR	A0A3Q1MP16	ENSBTAG00000012712	-0.169752441	-0.078002512	0.091749929
E1BPG0	MCAT	E1BPG0	ENSBTAG00000002413	-0.057333175	0.006231954	0.063565129
A0A3Q1MV30	LSG1	A0A3Q1MV30	ENSBTAG00000008461	0.079560378	-0.067853092	-0.14741347
A6QLZ0	LGALS3	A6QLZ0; bta:786492	ENSBTAG00000002326	0.373123858	0.792754468	0.419630611
A0AAF6Z9V5	IKBIP	A0AAF6Z9V5	ENSBTAG00000021660	0.06606901	-0.195158316	-0.261227326
E1BGS4	TXLNA	bta:511331; E1BGS4	ENSBTAG00000012867	0.097134183	-0.02419671	-0.121330894
A0A3Q1MDA4	DAG1	A0A3Q1MDA4	ENSBTAG00000011580	-0.038977139	-0.124290921	-0.085313782
F1MI35	HHIPL1	bta:100299504; F1MI35	ENSBTAG00000026913	0.082224131	-0.257262335	-0.339486466
E1BH39	KATNA1	bta:506715; E1BH39	ENSBTAG00000032024	0.104786707	-0.053074116	-0.157860823
Q2HJD7	HIBADH	bta:512002; Q2HJD7	ENSBTAG00000001036	0.133266531	0.097465809	-0.035800721
A0A140T867	KRT17	A0A140T867; bta:281889	ENSBTAG00000006806	-0.451749278	-0.484339778	-0.032590501
E1BC58	RAB2B	bta:528574; E1BC58	ENSBTAG00000044207	-0.025508533	-0.004468858	0.021039676
Q24K01	NUDCD3	bta:533678; Q24K01	ENSBTAG00000006325	0.26529038	0.175183464	-0.090106916
F1MMU4	H1-10	F1MMU4	ENSBTAG00000040116	0.109703919	0.236743108	0.127039189
A0AAF6YHP1	TM9SF4	A0AAF6YHP1	ENSBTAG00000001015	-0.10555865	0.00677005	0.1123287
A0AAA9T7H2	GEMIN4	A0AAA9T7H2	ENSBTAG00000013393	-0.083226907	0.080053252	0.163280159
A0A654IE56	CXNK1	A0A654IE56; bta:281193		-0.491823764	-0.426030113	0.065793651
A0A3Q1MP25	ACAD9	A0A3Q1MP25	ENSBTAG00000003242	-0.091824945	-0.060562724	0.031262221
Q02399	CDK5	bta:281066; Q02399; Q0VCN5; Q6LBE2		-0.038850929	0.041222663	0.080073592
A0A452DHU5	DAP3	A0A452DHU5	ENSBTAG00000001199	-0.316857105	-0.209303964	0.107553141
A7MB33	GTPBP1	A7MB33		-0.134680245	-0.084375163	0.050305082
Q3T0X6	RPS16	bta:506297; Q3T0X6	ENSBTAG00000021093	-0.295984828	-0.158337027	0.1376478
A7MAZ2	STX12	A7MAZ2; bta:505538; F1MIE7	ENSBTAG00000001192	-0.048309035	0.012613807	0.060922842
A0AAA9SKK8	MAP3K4	A0AAA9SKK8	ENSBTAG00000020079	0.031323311	-0.02943072	-0.060754031
A0AAF7ABH7	ITPK1	A0AAF7ABH7	ENSBTAG00000009845	0.323660765	0.420290973	0.096630208
P50291	FST	bta:327681; P50291; Q32XW5		-0.243728883	-0.587634654	-0.343905771
O18973	RABGEF1	bta:282335; O18973		0.080389387	0.06342538	-0.016964007
Q28165	PABPN1	A3KN38; bta:282298; Q28165	ENSBTAG00000006884	-0.005930923	-0.026883294	-0.020952371
F2Z4I2	AP3M1	F2Z4I2	ENSBTAG00000021883	0.082552927	0.122122993	0.039570066

Accession	Gene_Symbol	Gene_ID	Ensembl_Gene ID	log2(ratio(CM12H/CM0H))	log2(ratio(CM12R/CM0H))	log2(ratio(CM12H/CM12R))
Q148C9	HEBP1	bta:533227; Q148C9		0.080752198	0.010726407	-0.070025791
Q17QG3	RILPL1	bta:505840; Q17QG3		0.064756208	-0.171005267	-0.235761476
A5PKI3	FAM3C	A5PKI3; bta:615690	ENSBTAG00000007976	-0.044947406	-0.049520136	-0.00457273
F1MQY1	LIG3	F1MQY1	ENSBTAG00000018689	-0.229333647	-0.236067358	-0.006733711
A0AAA9TC35	MVD	A0AAA9TC35	ENSBTAG00000012059	-0.483082887	-0.381799551	0.101283336
G5E5S2	NELFB	G5E5S2	ENSBTAG00000021861	-0.023871404	-0.028391601	-0.004520197
Q2HJ18	VPS33B	A1L527; bta:526538; Q2HJ18		0	0.112778068	0.112778068
A0AAA9SD81	TM9SF2	A0AAA9SD81	ENSBTAG00000001589	0.042213525	0.046664001	0.004450477
Q2KHZ8	GBA1	bta:537087; Q2KHZ8		0.067638717	0.147213649	0.079574932
A0A3Q1MZJ3	THOC6	A0A3Q1MZJ3	ENSBTAG00000001415	-0.113039938	-0.092087567	0.020952371
A2VE52	REXO2	A2VE52; bta:540139	ENSBTAG00000005843	0.090986232	0.229648237	0.138662005
A0AAA9T8U5	CSAD	A0AAA9T8U5	ENSBTAG00000007829	-0.035489085	0.032001942	0.067491026
A0A3Q1MCY3	MYO10	A0A3Q1MCY3	ENSBTAG00000020126	-0.201693429	-0.166504	0.035189428
A0AAF6YUU7	NMRAL1	A0AAF6YUU7	ENSBTAG00000010015	0.004508426	0.046650093	0.042141668
A0AAA9SPH3	ARMC10	A0AAA9SPH3	ENSBTAG00000014161	-0.012035065	-0.075341633	-0.063306568
P38409	GNA11	bta:281788; P38409; Q2TA47	ENSBTAG00000012181	0.016855896	-0.050185757	-0.067041653
P48427	TBCA	bta:327683; P48427; Q32KV5	ENSBTAG00000003263	0.043633216	0.004423187	-0.039210029
E1BDN8	SREK1	bta:507801; E1BDN8	ENSBTAG00000007321	0.30651955	0.173991537	-0.132528013
A0AAA9TXU6	HELLS	A0AAA9TXU6	ENSBTAG00000005979	-0.183937092	-0.452342851	-0.268405759
F1N458	ARHGAP17	F1N458	ENSBTAG00000020043	0.056877087	0.070025791	0.013148704
F1N020	KIF3B	bta:508404; F1N020	ENSBTAG00000003215	0.115131455	0.065887936	-0.049243519
F1N2H6	CD276	bta:508656; F1N2H6	ENSBTAG00000019734	0.05399489	0.139724764	0.085729874
F1MND3	TEX10	F1MND3	ENSBTAG00000044003	-0.108961809	-0.012649136	0.096312673
Q3ZBU9	UBXN4	bta:536181; Q3ZBU9	ENSBTAG00000015169	-0.096761556	-0.158095158	-0.061333603
F1N1X8	UBE2Z	F1N1X8	ENSBTAG00000018802	-0.152582955	-0.113956189	0.038626766
F1MBS4	SLC25A10	F1MBS4	ENSBTAG00000055145	-0.096397332	0.003514484	0.099911816
A0A3Q1N3E8	UFD1	A0A3Q1N3E8	ENSBTAG00000004284	0.026499225	-0.025482029	-0.051981255
P00125	CYC1	bta:512500; P00125; Q2TBM2	ENSBTAG00000012232	-0.077846587	-0.104757741	-0.026911154
A0A3Q1NNG9	FCHO2	A0A3Q1NNG9	ENSBTAG00000017545	-0.029975059	-0.03796785	-0.007992791
A0AAA9U0A2	CCPG1	A0AAA9U0A2; bta:538508	ENSBTAG00000012741	0.179811859	-0.393272291	-0.57308415
G5E5M5	PGRMC1	G5E5M5	ENSBTAG00000019552	-0.15607563	-0.149294442	0.006781188
A0AAF6Z0W3	LANCL1	A0AAF6Z0W3	ENSBTAG00000015066	-0.040466592	0.122073483	0.162540075
A0A3Q1LNZ3	NUCKS1	A0A3Q1LNZ3	ENSBTAG00000008001	-0.102233658	-0.021936992	0.080296666
A6QLW0	BUB1	A6QLW0; bta:514777		0.391042018	0.194503024	-0.196538994
A0AAA9TJD5	STK39	A0AAA9TJD5	ENSBTAG00000017162	0.049234182	-0.137086218	-0.1863204
A0A3Q1MIT1	YTHDF3	A0A3Q1MIT1	ENSBTAG00000018557	0.08285043	-0.039683701	-0.122534132
A0A452DIS6	ERP27	A0A452DIS6	ENSBTAG00000006036	-0.050626073	0	0.050626073
A0A3Q1MFQ7	EWSR1	A0A3Q1MFQ7	ENSBTAG00000017755	-0.176030364	-0.035241006	0.140789358
A4IFE3	ACTR1B	A4IFE3; bta:100125305	ENSBTAG00000048041	-0.080891452	0.056754778	0.137646231
G5E547	SYAP1	G5E547	ENSBTAG00000003316	0.012454999	-0.063940821	-0.076395819
Q9MZ13	VDAC3	bta:282716; Q9MZ13		-0.084850482	-0.025580369	0.059270112
F6RA54	OGFR	F6RA54	ENSBTAG00000031718	-0.229388688	-0.10359389	0.125794798
A0AAA9SQN4	ARIH1	A0AAA9SQN4	ENSBTAG00000006708	0.187520046	0.115139863	-0.072380183
Q3ZC42	ADH5	bta:505515; Q3ZC42	ENSBTAG00000016007	0.103396434	0.138940551	0.035544117
A0AAA9RUF4	PPIE	A0AAA9RUF4	ENSBTAG00000047314	0.026891646	-0.051110866	-0.078002512
G5E518	CDK12	G5E518	ENSBTAG00000013238	-0.041499363	-0.091474005	-0.049974642
A0AAA9SBQ7	ATM	A0AAA9SBQ7	ENSBTAG00000003111	-0.275161217	-0.196504273	0.078656944
Q0V8C2	EXOC3	bta:513138; Q0V8C2	ENSBTAG00000008606	-0.004249473	-0.043068722	-0.038819249
A0A3Q1MNZ0	ATPAF1	A0A3Q1MNZ0; bta:505430	ENSBTAG00000000094	-0.306552869	-0.201702563	0.104850306
A0A3Q1N5G3	MSRA	A0A3Q1N5G3	ENSBTAG00000021632	-0.017243572	-0.074752573	-0.057509
A0A3Q1M3H2	LTBP2	A0A3Q1M3H2	ENSBTAG00000021957	-0.202404875	-0.348507367	-0.146102492
A5PKA3	CCDC80	A5PKA3; bta:515235		-0.628824872	-0.888354644	-0.259529773
F6QJJ8	PGRMC2	F6QJJ8	ENSBTAG00000010843	-0.056893485	-0.01245052	0.044442965
A0A3Q1MJT2	A1BG	A0A3Q1MJT2	ENSBTAG00000009735	-0.17493193	0.228776038	0.403707968
E1B7G6	ANKRD50	E1B7G6	ENSBTAG00000001117	-0.022775258	-0.078582024	-0.055806766
E1BNY8	GTF3C1	E1BNY8	ENSBTAG00000006126	0.024222102	-0.089754653	-0.113976754
F1MZV2	CHMP5	F1MZV2	ENSBTAG00000012383	0.243299709	0.258610654	0.015310945
P22292	SLC25A11	A5PJY2; bta:282523; P22292; Q5E9W4	ENSBTAG00000004910	0.036188308	0.045562577	0.00937427
F1MQ09	VEZT	F1MQ09	ENSBTAG00000014044	-0.06515268	-0.127917475	-0.062764795
Q58CU6				-0.019508159	-0.125050544	-0.105542385
E1BBS6	QSOX2	E1BBS6	ENSBTAG00000011129	-0.17071756	-0.087542078	0.083175483
F1MMA2	TTL	bta:514559; F1MMA2	ENSBTAG00000014159	-0.015957574	0.027154052	0.043111625
F1MDA6	VPS45	F1MDA6	ENSBTAG00000020635	0.150980579	-0.107589308	-0.258569887
E1BI11	MIDEAS	bta:505848; E1BI11	ENSBTAG00000009272	-0.223912648	-0.296233072	-0.072320424
Q3SWX4	NIPSNAP2	bta:767938; E1BL91; Q3SWX4	ENSBTAG00000031598	-0.157739463	-0.200024608	-0.042285145
A0AAA9RZZ1	GALM	A0AAA9RZZ1	ENSBTAG00000021102	0.163937642	0.369382487	0.205444846
Q3SZR3	ORM1	bta:497200; Q3SZR3		-0.581567124	0.134905299	0.716472422
F1MI29	XPO6	bta:510797; F1MI29	ENSBTAG00000010309	-0.231494628	-0.154453593	0.077041036
Q1RMW9	GOLPH3	bta:531359; Q1RMW9	ENSBTAG00000032848	0.115033243	-0.066427362	-0.181460605
E1BGC1	MCCC1	E1BGC1	ENSBTAG00000006548	-0.122073483	-0.034249562	0.087823921
A0AAA9RUI1	PPP6R1	A0AAA9RUI1	ENSBTAG00000021157	-0.046683791	-0.087283413	-0.040599621
A0A3Q1LIJ0	YBX3	A0A3Q1LIJ0	ENSBTAG00000009663	-0.160227861	-0.190537695	-0.030309834
Q08DA0	RABL6	bta:508218; Q08DA0		-0.020738666	-0.139701498	-0.118962832
A1A4P5	PFDN2	A1A4P5; bta:404138		0.134796778	-0.039197888	-0.173994666
A0AAF6YJR2	FKBP3	A0AAF6YJR2	ENSBTAG00000002610	0.148808985	0.227930605	0.07912162
F6QMQ4	RPS14	F6QMQ4	ENSBTAG00000008570	-0.081280688	-0.108461667	-0.027180978
A7MAZ9	DCP1A	A7MAZ9; bta:783258		0.034800362	0.034800362	0
A0AAA9SAW2	LPCAT4	A0AAA9SAW2	ENSBTAG00000020040	-0.15436851	-0.16134818	-0.00697967
A0A3Q1MTL1	SCAF4	A0A3Q1MTL1	ENSBTAG00000018855	-0.031535573	0.107657713	0.139193286
A0A452DIZ8	STRIP1	A0A452DIZ8	ENSBTAG00000020964	-0.123714049	0.066984334	0.190698383
G5E6N7	CBX1	G5E6N7	ENSBTAG00000002949	0.163875957	0.047500424	-0.116375533
E1BEV7	BMP1	E1BEV7	ENSBTAG00000000917	-0.158856764	-0.459944516	-0.301087752
A0A452DI40	IVD	A0A452DI40	ENSBTAG00000004409	-0.07339541	-0.052041532	0.021353878
A0AAA9TAZ9	PTPN2	A0AAA9TAZ9	ENSBTAG00000010563	-0.124328135	-0.023724194	0.100603941
A0A3Q1M0U3	SORBS3	A0A3Q1M0U3	ENSBTAG00000014401	-0.115224401	-0.024478976	0.090745425
A0AAF6DM45	ABCF3	A0AAF6DM45	ENSBTAG00000006920	-0.299857837	-0.200216359	0.099641478
Q5E9C0	RSU1	bta:617534; Q5E9C0		0.14360813	0.12717124	-0.01643689
A0AAA9TQT8	ATE1	A0AAA9TQT8	ENSBTAG00000003178	0.117229732	0.213057578	0.095827846
F1MEG3	LAMA1	F1MEG3	ENSBTAG00000018160	0.023458973	0.087926507	0.064467534
F1N455	CTSC	F1N455	ENSBTAG00000011100	-0.026073512	0.043828502	0.069902013

Accession	Gene_Symbol	Gene_ID	Ensembl_Gene ID	log2(ratio(CM12H/CM0H))	log2(ratio(CM12R/CM0H))	log2(ratio(CM12H/CM12R))
Q08DL0	SLC3A2	bta:507107; Q08DL0	ENSBTAG00000013240	-0.337923746	-0.267456264	0.070467483
E1B725	GLA	bta:532742; E1B725	ENSBTAG00000019256	-0.067648692	0.011178924	0.078827616
Q17QZ7	TTC27	bta:524908; Q17QZ7		-0.24003048	-0.303519306	-0.063488826
Q5E9N5	CLPB	bta:520639; Q5E9N5		0.258193143	0.063815098	-0.194378045
A0AAA9TTF1	NOL10	A0AAA9TTF1	ENSBTAG00000002716	0.14076238	-0.213091603	-0.353853983
F1MLG1	SRPRB	F1MLG1	ENSBTAG00000044075	0.223018727	0.085139452	-0.137879275
Q3MHI4	PHAX	bta:507478; Q3MHI4	ENSBTAG00000009647	0.171428592	0.148538877	-0.022889716
A0AAA9SCC4	SEPTIN10	A0AAA9SCC4	ENSBTAG00000022461	-0.280275626	-0.189127738	0.091147888
A0AAA9T4C9	SLC44A1	A0AAA9T4C9	ENSBTAG00000007163	0.208359724	0.237243473	0.028883748
A0AAA9TAH2	PUM3	A0AAA9TAH2	ENSBTAG00000013357	-0.345579363	-0.270089163	0.0754902
A0A3Q1M182	VAV2	A0A3Q1M182	ENSBTAG00000004511	-0.399251532	-0.167674557	0.231576974
A0A3Q1MQ45	AKT2	A0A3Q1MQ45	ENSBTAG00000001400	-0.100008456	-0.152003093	-0.051994638
A6QQX0	PBK	A6QQX0; bta:534781	ENSBTAG00000021069	-0.354642075	-0.015385163	0.339256911
A0A3Q1MGZ4	TKFC	A0A3Q1MGZ4	ENSBTAG00000018201	-0.094557997	-0.104530583	-0.009972586
A5PJ81	LACTB	A5PJ81; bta:614196	ENSBTAG00000017373	-0.1153734	-0.188734851	-0.073361451
A0AAA9TAG5	SELENBP1	A0AAA9TAG5	ENSBTAG00000008091	0.207782726	0.334231062	0.126448335
F6RN75	SYNPO2	F6RN75	ENSBTAG00000006434	0.089392377	0.12566113	0.036268754
Q2HJD0	FAF2	bta:767948; Q2HJD0	ENSBTAG00000017744	-0.081933271	-0.192094606	-0.110161335
E1BB26	HEATR5B	bta:540503; E1BB26	ENSBTAG00000014310	0.076725791	0.022554316	-0.054171474
A0AAA9T3F8	TACC3	A0AAA9T3F8	ENSBTAG00000011044	-0.049105472	0.237727506	0.286832978
A0AAA9SP28	PHF2	A0AAA9SP28	ENSBTAG00000012922	0.18223147	0.107152353	-0.075079117
E1BDD9	MRTFA	bta:509011; E1BDD9	ENSBTAG00000002630	0.15284407	0.073790261	-0.079053809
G1K192	ARL3	G1K192	ENSBTAG00000004318	0.100643077	0.066427362	-0.034215715
A0AAA9SCV4	UAP1L1	A0AAA9SCV4	ENSBTAG00000048054	-0.332575339	-0.262186011	0.070389328
F1MFX7	UHRF2	F1MFX7	ENSBTAG00000020815	-0.500102983	-0.27190494	0.228198043
F1MNM6	TANGO6	bta:534015; F1MNM6	ENSBTAG00000013941	0.069214495	0.122573617	0.053359122
A0AAA9U1B4	SRSF10	A0AAA9U1B4	ENSBTAG00000008072	0.085575732	-0.061400545	-0.146976276
A0A3Q1M100	ITSN2	A0A3Q1M100	ENSBTAG00000023172	-0.173390521	-0.124272754	0.049117767
G3MX91	TARDBP	bta:540632; G3MX91	ENSBTAG00000003697	0.054392297	0.071601587	0.01720929
A0A3Q1N6R4	CCDC22	A0A3Q1N6R4	ENSBTAG00000013277	0.038360474	0.101454151	0.063093678
A0AAA9TQ21	ESF1	A0AAA9TQ21	ENSBTAG00000007130	0.103513957	-0.009789317	-0.113303274
F6QY62	INTS4	F6QY62	ENSBTAG00000010505	-0.326173995	-0.205333159	0.120840836
A0AAF7ABD1	ERLIN2	A0AAF7ABD1	ENSBTAG00000017831	-0.098180394	0.03129613	0.129476524
A0A452DI32	BCCIP	A0A452DI32	ENSBTAG00000005792	0.065053387	-0.032936404	-0.097989791
A0AAA9S584	CPSF3	A0AAA9S584	ENSBTAG00000019735	-0.078917057	-0.061639066	0.017277991
Q2KIR1	SNRPA	bta:509802; Q2KIR1	ENSBTAG00000009077	-0.048715285	-0.019860422	0.028854863
A0AAA9TI02	CERT1	A0AAA9TI02	ENSBTAG00000000081	0.103667596	0.205643267	0.101975671
E1BLF7	NUDCD1	E1BLF7	ENSBTAG00000007386	0.095624604	0.060942764	-0.034681839
A0AAF6ZJM9	QDPR	A0AAF6ZJM9	ENSBTAG00000040333	0.103148523	0.161388319	0.058239796
Q3T133	TMED9	bta:618580; Q3T133; Q5E957	ENSBTAG00000001394	0.207920124	0.230917156	0.022997032
A0A3Q1NKB1	RANBP1	A0A3Q1NKB1	ENSBTAG00000006656	0.039615203	-0.044042726	-0.083657929
A0A3Q1MRI8	IDH3G	A0A3Q1MRI8	ENSBTAG00000001059	-0.138913094	-0.12041675	0.018496344
A4FUC6	SRSF9	A4FUC6; bta:509905; F1MWF4	ENSBTAG00000012792	0.004496715	-0.010547165	-0.01504388
A4FV64	SUGP1	A4FV64; bta:533971; E1BMW4	ENSBTAG00000006116	-0.103190559	-0.033136391	0.070054168
F1N081	FNTA	bta:281169; F1N081	ENSBTAG00000003201	-0.027244836	-0.100324534	-0.073079698
A0A3Q1MXQ4	NHSL3	A0A3Q1MXQ4	ENSBTAG00000018062	0.04051516	0.02713613	-0.01337903
A5D9H9	POLDIP2	A5D9H9; bta:539673; F1MFH2	ENSBTAG00000008116	-0.26117106	-0.256987312	0.004183749
A8E644	DCLK1	A8E644; bta:613449; F1ME50		0.152003093	0.017073513	-0.13492958
A0AAF6YXA0	MAT2B	A0AAF6YXA0	ENSBTAG00000012350	0.032090925	0.021473067	-0.010617859
Q0VCQ6	PDCD10	bta:506411; Q0VCQ6	ENSBTAG00000031709	0.009429413	-0.030273011	-0.039702424
A6H704	CHMP7	A6H704; bta:541288	ENSBTAG00000013528	0.1123287	0.036051227	-0.076277473
F1MIZ7	RO60	F1MIZ7	ENSBTAG00000013812	-0.024401343	-0.009102207	0.015299136
Q3SZG7	RPL13	Q3SZG7		-0.099060869	-0.063888599	0.03517227
Q32KN6	PGK2	bta:538592; Q32KN6		0.137381059	0.20321432	0.065833261
P30274	CCNA2	bta:281667; P30274; Q17QS7		0.175419758	0.17154328	-0.003876478
E1BAT8	KIF20B	E1BAT8	ENSBTAG00000005708	-0.06333457	0.098906911	0.162241482
A0AAA9SBU8	GTSE1	A0AAA9SBU8	ENSBTAG00000007102	-0.071532867	-0.146798416	-0.075265549
A0A3Q1LQN8	GPKOW	A0A3Q1LQN8	ENSBTAG00000002568	-0.229097445	-0.305576175	-0.07647873
A0AAA9U021	HACD3	A0AAA9U021	ENSBTAG00000015155	0.254369545	0.178538132	-0.075831413
A0AAA9SEF0	NBEAL1	A0AAA9SEF0	ENSBTAG00000014469	-0.153805336	0.010278787	0.164084123
A0AAA9SWJ0	ARAP1	A0AAA9SWJ0	ENSBTAG00000002051	-0.071317405	-0.02181222	0.049505185
A0AAF6YNA5	ZWILCH	A0AAF6YNA5	ENSBTAG00000005213	-0.497299048	-0.303851805	0.193447244
A0A3Q1LTK2	VPS41	A0A3Q1LTK2	ENSBTAG00000007305	-0.043068722	-0.001516232	0.041552489
Q0P565	HDDC2	bta:509282; Q0P565		-0.027456963	-0.292256442	-0.264799479
A0AAA9SHG0	FAM120C	A0AAA9SHG0	ENSBTAG00000003855	-0.031842171	-0.033376137	-0.001533966
A0A3Q1M5Q9	PTPRF	A0A3Q1M5Q9	ENSBTAG00000000253	0.043197689	-0.173767068	-0.216964757
A0A3Q1LWF1	MARK3	A0A3Q1LWF1	ENSBTAG00000013410	-0.031498153	-0.170941342	-0.139443189
A0A3Q1MDQ6	KLHDC4	A0A3Q1MDQ6	ENSBTAG00000006905	-0.016378928	-0.117287943	-0.100909015
A0A3Q1N4D8	POLD2	A0A3Q1N4D8	ENSBTAG00000012241	-0.166675164	-0.12688362	0.039791544
F1MBJ4	PAXBP1	bta:514918; F1MBJ4	ENSBTAG00000003064	0.046589559	0.094495945	0.047906385
E1BMM8	CHPF	E1BMM8	ENSBTAG00000020539	-0.224264836	-0.296885508	-0.072620672
Q08DP5	KAT7	bta:508246; F1MQP0; Q08DP5	ENSBTAG00000018699	-0.216200588	-0.185633871	0.030566716
Q0VC82	SEPHS1	bta:507470; Q0VC82		0.171015062	0.125258238	-0.045756824
Q3SZZ0	BRIX1	bta:518501; Q3SZZ0	ENSBTAG00000019211	-0.220538653	-0.124898648	0.095640005
A0AAF6YL13	CSTF2	A0AAF6YL13	ENSBTAG00000003547	0.185735516	0.169925001	-0.015810515
A0A3Q1LTT9	MAPK14	A0A3Q1LTT9	ENSBTAG00000020783	-0.285287213	-0.260093131	0.025194082
A0A3Q1MAC0	FNBP1L	A0A3Q1MAC0	ENSBTAG00000004383	0.047305715	0.093109404	0.04580369
Q2TA25	PLK1	bta:538238; F1N225; Q2TA25	ENSBTAG00000014453	-0.261508552	-0.100081013	0.161427539
A0AAF6YQG5	EPB41	A0AAF6YQG5	ENSBTAG00000006667	0.059533323	0.057997724	-0.001535599
E1B8R6	ARIH2	E1B8R6	ENSBTAG00000005488	-0.025943267	0.066984334	0.092927602
E1BFI8	SPAG1	E1BFI8	ENSBTAG00000050156	-0.091392932	-0.029277793	0.062115139
P05689	CTSZ	bta:404187; P05689; Q0IIK1	ENSBTAG00000018784	0.052984456	0.205748665	0.152764209
F6QN89	SNTB2	F6QN89	ENSBTAG00000001654	0.044761451	0.08246216	0.037700709
A6QLG5	RPS9	A6QLG5; bta:533892	ENSBTAG00000006487	-0.28580338	-0.162882766	0.122920614
A4IFP7	ARF5	A4IFP7; bta:511918		0.141331407	0.104294028	-0.037037379
E1B731	TMEM165	bta:532600; E1B731	ENSBTAG00000001269	0.154674882	0.145796731	-0.008878151
Q3SZK4	TBRG4	bta:767855; Q3SZK4		-0.245910228	-0.18497523	0.060934998
F6RSR1	KLHL3	F6RSR1	ENSBTAG00000002796	-0.184489629	-0.119298928	0.065190701
A0AAA9SZ85	ADPRHL1	A0AAA9SZ85	ENSBTAG00000020928	-0.021859434	-0.138805791	-0.116946357
Q27U16	TRIM5	bta:505265; Q27U16		0.280174793	-0.039181027	-0.31935582
A6QPQ5	MRPL1	A6QPQ5; bta:504835	ENSBTAG00000018360	-0.078599098	-0.146793459	-0.068194361

Accession	Gene_Symbol	Gene_ID	Ensembl_Gene ID	log2(ratio(CM12H/CM0H))	log2(ratio(CM12R/CM0H))	log2(ratio(CM12H/CM12R))
A0AAA9SLW2	VPS53	A0AAA9SLW2	ENSBTAG00000018126	-0.070389328	-0.059211779	0.011177549
A0A3Q1M8V6	DAZAP1	A0A3Q1M8V6	ENSBTAG00000019714	0.012990839	-0.099017929	-0.112008768
A0AAA9RZX1	PIGS	A0AAA9RZX1	ENSBTAG00000013098	0.058835101	0	-0.058835101
A0AAA9SE16	STON1	A0AAA9SE16	ENSBTAG00000004168	-0.024153862	-0.065974037	-0.041820176
A0AAF6ZIV2	GFM2	A0AAF6ZIV2	ENSBTAG00000015519	-0.204944343	-0.081885946	0.123058396
F1MCX5	MAPKAPK2	F1MCX5	ENSBTAG00000038107	-0.260740165	-0.271477976	-0.010737812
E1BQ16	OGA	bta:538561; E1BQ16	ENSBTAG00000016336	-0.05074096	-0.037474705	0.013266255
Q2KIY3	WDFY1	bta:614729; Q2KIY3	ENSBTAG00000016951	-0.113472639	-0.008046284	0.105426354
A0AAF6Z3X9	PTGIS	A0AAF6Z3X9	ENSBTAG00000017537	0.029559798	-0.063399568	-0.092959366
Q5BIS9	PRKAB1	A6QQW7; bta:534107; Q5BIS9	ENSBTAG00000005940	-0.034203424	0.180301088	0.214504512
Q3T057	RPL23	A6QLM2; bta:504876; Q3T057	ENSBTAG00000003229	-0.245066633	-0.245066633	0
Q3SZD7	CBR1	bta:515946; Q3SZD7	ENSBTAG00000023384	-0.085693748	0.01759406	0.103287808
A6H7F3	BCL2L14	A6H7F3		0.034119378	0.050881169	0.016761791
A0AAA9TIA3	CFH	A0AAA9TIA3	ENSBTAG00000023177	-0.065821123	0.106097929	0.171919052
E1BEI2	SPCS2	E1BEI2	ENSBTAG00000006644	0.007462232	-0.01378182	-0.021244053
Q1RMI2	RHOG	bta:538559; Q1RMI2	ENSBTAG00000000347	0.08639558	0.299663328	0.213267748
A6QLU9	LOC508963	A6QLU9; bta:508963		0.074870286	0.017295253	-0.057575034
A0AAF6ZBL8	ZNF512	A0AAF6ZBL8	ENSBTAG00000003902	-0.01637297	-0.139257865	-0.122884895
E1BIE7	PGAM5	bta:508608; E1BIE7	ENSBTAG00000007513	-0.153886791	-0.057157071	0.09672972
A0AAA9SDC5	LSM14A	A0AAA9SDC5	ENSBTAG00000000630	0.029389249	-0.112219427	-0.141608676
Q2KIC7	PPP6C	bta:511058; F1N6Z5; Q2KIC7	ENSBTAG00000008033	-0.133311671	0.031830534	0.165142205
A0AAA9T3G0	RPL19	A0AAA9T3G0	ENSBTAG00000002060	-0.057100407	0.046610942	0.10371135
E1BM72	PES1	E1BM72	ENSBTAG00000007728	-0.40636131	-0.423005066	-0.016643756
E1B8G1	NUMB	E1B8G1	ENSBTAG00000011683	0.220055729	0.089297053	-0.130758675
F1MW73	CRACD	F1MW73	ENSBTAG00000040398	0.367793755	0.294904921	-0.072888833
Q3ZCF3	SKP1	bta:615427; Q3ZCF3	ENSBTAG00000025191	-0.029087579	0.155454519	0.184542098
E1BN70	INTS3	bta:511052; E1BN70	ENSBTAG00000006235	-0.185915729	-0.052078867	0.133836861
A0AAF7AEM3	SGTA	A0AAF7AEM3	ENSBTAG00000015090	0.06511729	0.110694863	0.045577572
A0A3Q1MU57	DDX54	A0A3Q1MU57	ENSBTAG00000011930	-0.39115255	-0.377463195	0.013689355
A0A3Q1MPG8	GBE1	A0A3Q1MPG8	ENSBTAG00000036262	-0.150092781	-0.055109636	0.094983145
Q58DG1	MYG1	Q58DG1		-0.058020953	-0.050251979	0.007768974
A0AAF6ZVE8	EEF1B2	A0AAF6ZVE8	ENSBTAG00000021979	-0.091231451	-0.12157606	-0.030344609
Q3B7M7	URI1	A7E3X6; bta:515119; Q3B7M7		-0.023945696	0.013296823	0.037242518
Q7YRD0	PNO1	A6QLI9; bta:360193; Q7YRD0	ENSBTAG00000010604	-0.330398874	-0.529125105	-0.198726231
H7BWW2				0.071053297	0.104588901	0.033535604
A0A452DIE1	BTF3L4	A0A452DIE1	ENSBTAG00000011025	0.02691409	-0.034730545	-0.061644635
Q2HJ57	COTL1	bta:617165; Q2HJ57	ENSBTAG00000016315	0.10433666	0.227068909	0.122732249
Q24K16	PTGR3	bta:532505; Q24K16	ENSBTAG00000001724	-0.043516722	-0.041994089	0.001522633
A0AAA9SNR2	LOC515358	A0AAA9SNR2	ENSBTAG00000062846	0.003286323	0.093931687	0.090645364
P12624	MARCKS	bta:613548; P12624; Q1LZI0	ENSBTAG00000002082	-0.033166864	-0.067114196	-0.033947332
Q08E32	CHMP4B	bta:616164; F1MAW4; Q08E32	ENSBTAG00000013387	0.185920165	0.121594838	-0.064325327
Q2KID9	MRPS5	bta:512469; P82674; Q2KID9		-0.351637931	-0.211630452	0.140007479
A0AAA9SRD9	TIA1	A0AAA9SRD9	ENSBTAG00000019182	0.062451508	-0.01491942	-0.077370928
E1BAJ4	STBD1	bta:513376; E1BAJ4	ENSBTAG00000010389	0.025039746	0.022116351	-0.002923395
A0A3Q1MYN1	CNOT10	A0A3Q1MYN1	ENSBTAG00000004834	0.048590738	0.091115354	0.042524617
Q3MHP2	RAB11B	bta:532723; Q3MHP2	ENSBTAG00000002475	0.060858348	0.058083934	-0.002774414
A5PJS3	DBR1	A5PJS3	ENSBTAG00000019949	-0.151117733	-0.048634775	0.102482958
A0AAF6YGY8	GRWD1	A0AAF6YGY8	ENSBTAG00000000080	0.104307408	-0.046006575	-0.150313983
A0AAA9TJF9	PDPK1	A0AAA9TJF9	ENSBTAG00000034436	0.098672387	-0.066224045	-0.164896432
Q1LZB5	TOMM40	bta:510219; Q1LZB5	ENSBTAG00000001781	-0.060120992	-0.029747343	0.030373649
Q3T094	ETHE1	bta:509150; Q3T094	ENSBTAG00000004379	-0.013016886	-0.061776198	-0.048759312
A0AAF6YUZ1	NUDT21	A0AAF6YUZ1	ENSBTAG00000010207	0.037719666	0.00816622	-0.029553446
Q08DB4	CPNE1	bta:615677; Q08DB4		0	0.076739443	0.076739443
A3KMX6	UBR7	A3KMX6; bta:505130	ENSBTAG00000004310	0.112414465	-0.027990744	-0.140405209
F1MI95	ERMP1	F1MI95	ENSBTAG00000015607	0	-0.054819168	-0.054819168
A0AAF6YNF5	CSNK1A1	A0AAF6YNF5	ENSBTAG00000005326	-0.139193286	-0.255334881	-0.116141595
A0AAA9RYF3	CENPC	A0AAA9RYF3	ENSBTAG00000001323	-0.040252167	-0.347585026	-0.307332859
P01131	LDLR	bta:281276; F1MZ58; P01131	ENSBTAG00000012314	-0.391637067	-0.279019503	0.112617563
A0A3Q1MCW7	CRYBG1	A0A3Q1MCW7; bta:526200	ENSBTAG00000017527	0.231579725	0.153512261	-0.078067465
F1MM83	PGLS	F1MM83	ENSBTAG00000016783	0.066551193	0.290631964	0.224080771
G3MZH3	MYO5A	G3MZH3	ENSBTAG00000006489	-0.156358872	0.089435084	0.245793956
Q2NL21	DNAJC11	bta:508137; Q2NL21	ENSBTAG00000011217	0.058893689	0.05600541	-0.002888279
A0AAF6YPQ0	FECH	A0AAF6YPQ0	ENSBTAG00000006393	-0.093109404	-0.039170597	0.053938807
A0AAF6YKI7	RMDN3	A0AAF6YKI7	ENSBTAG00000002921	-0.071502948	-0.076189563	-0.004686615
A5PKG4	NFS1	A5PKG4; bta:517656; F1MZY8		0.059747463	0.062670858	0.002923395
A0AAA9TXM1	ALDH5A1	A0AAA9TXM1; bta:532724	ENSBTAG00000021902	0.005957455	0.046984724	0.041027268
A0AAA9T8I6	RDH11	A0AAA9T8I6	ENSBTAG00000001950	-0.029670867	0.270357747	0.300028613
A0A3Q1LP37	UBE2S	A0A3Q1LP37	ENSBTAG00000009211	-0.031933465	0.112948391	0.144881856
A0AAA9RWG1	ELMO2	A0AAA9RWG1	ENSBTAG00000003639	0.094996514	0.072186631	-0.022809883
F1MIM5	VWA8	F1MIM5	ENSBTAG00000001133	-0.11417102	-0.195091015	-0.080919995
F1N0D3	AXL	F1N0D3	ENSBTAG00000003166	-0.0768936	-0.293811722	-0.216918122
Q3T0I4	ALYREF	bta:537706; Q3T0I4	ENSBTAG00000008498	-0.081019351	-0.17558676	-0.094567409
G3N0V2	KRT1	G3N0V2	ENSBTAG00000046089	-0.390033493	-0.159871337	0.230162156
A0AAF7AEY9	AK1	A0AAF7AEY9	ENSBTAG00000006305	0.223671974	0.311495895	0.087823921
F1MM32	QSOX1	F1MM32	ENSBTAG00000014191	-0.084309967	-0.262761452	-0.178451485
A0A3Q1NBY0	NTAN1	A0A3Q1NBY0; bta:505869	ENSBTAG00000004803	-0.041157325	0.01346226	0.054619585
A8E4P3	STOM	A8E4P3; bta:100125834; F1N2R1	ENSBTAG00000038375	-0.100103552	0.053318819	0.153422371
A5PJF7	CCL2	A5PJF7		-0.942212888	-1.57508277	-0.632869882
A0AAA9T6K1				0.02120609	0.024010163	0.002804073
Q5E9J8	RIC8A	bta:521037; Q17QK2; Q5E9J8		-0.099001836	-0.129750287	-0.03074845
C3V9V7	TMED2	bta:100140040; C3V9V7	ENSBTAG00000039630	0.145245727	0.127527001	-0.017718726
B0JYN8	PSMB3	B0JYN8; bta:533874		0.029831223	0.125873362	0.096042138
A0AAA9TKZ5	CFL2	A0AAA9TKZ5	ENSBTAG00000015053	0.02520534	0.099314554	0.074109214
Q5EA75	PCYT2	bta:510274; Q5EA75		-0.116133734	-0.121836093	-0.005702359
Q2T9L9	GTF2F2	bta:509259; Q2T9L9	ENSBTAG00000034495; ENSBTAG00000049772	0.040197089	-0.011693641	-0.05189073
Q0VC21	MRPL15	bta:510347; Q0VC21	ENSBTAG00000001174	-0.151692469	-0.005602706	0.146089763
A0A3Q1LVW7	TBL3	A0A3Q1LVW7	ENSBTAG00000020366	-0.159366248	-0.240224523	-0.080858274
A0AAA9S0B0	DDI2	A0AAA9S0B0	ENSBTAG00000039662	0.004806318	-0.035744273	-0.040550591
F1MB04	CASP3	F1MB04	ENSBTAG00000015874	0.005353234	0.104710787	0.099357552
Q17R06	RAB21	bta:507488; Q17R06	ENSBTAG00000053598	0.139772805	0.242328644	0.10255584

Accession	Gene_Symbol	Gene_ID	Ensembl_Gene ID	log2(ratio(CM12H/CM0H))	log2(ratio(CM12R/CM0H))	log2(ratio(CM12H/CM12R))
A0A3Q1MK97	AHNAK2	A0A3Q1MK97	ENSBTAG00000048906	0.183878768	0.348288681	0.164409914
A0AAA9SEF4	NSRP1	A0AAA9SEF4	ENSBTAG00000019341	0.09261115	-0.094704424	-0.187315574
A0A3Q1LRS4	SMARCA2	A0A3Q1LRS4	ENSBTAG00000007494	0.142527766	0.22842784	0.085900073
P51122	ANP32A	bta:538427; P51122; P55930	ENSBTAG00000012365	0.118644496	0.195416827	0.076772331
A8E658	PPIG	A8E658; bta:783720; F1MD69	ENSBTAG00000027789	-0.030171528	-0.0723586	-0.042187071
A0AAF6YW73	NAXD	A0AAF6YW73	ENSBTAG00000011466	-0.016961992	0.013056153	0.030018145
A0AAA9TPL0	SUN1	A0AAA9TPL0	ENSBTAG00000046498	0.255887512	0.066495412	-0.1893921
A0A3Q1M273	PBXIP1	A0A3Q1M273	ENSBTAG00000004552	0.127111918	0.051789302	-0.075322616
F6RSB3	ZRANB2	F6RSB3	ENSBTAG00000002627	0.247092862	0.05375368	-0.193339182
Q08DH9	CTCF	bta:517097; F1MK31; Q08DH9	ENSBTAG00000013757	-0.005581032	-0.016808288	-0.011227255
F1N318	VTA1	bta:506430; F1N318	ENSBTAG00000005851	-0.093714435	0	0.093714435
Q75WB5	OPLAH	bta:408006; F1ME97; Q75WB5		-0.11995151	-0.226603136	-0.106651626
Q0VCL3	ATG3	bta:508571; Q0VCL3	ENSBTAG00000009084	0.081427599	-0.065047297	-0.146474897
F1MZ61	SMG8	F1MZ61	ENSBTAG00000003365	-0.092193698	-0.165808893	-0.073615195
E1BN60	SLC30A1	bta:522265; E1BN60	ENSBTAG00000010909	-0.053737871	-0.030654258	0.023083613
A4IFT6	TMED7	A4IFT6; bta:100125926	ENSBTAG00000035030	0.090197809	0.117623553	0.027425744
A4FUC0	MRPL37	A4FUC0; bta:510127	ENSBTAG00000010715	-0.279763601	-0.297429475	-0.017665874
A0AAF6ZJH4	VPS28	A0AAF6ZJH4	ENSBTAG00000026320	0.041349259	0.025245951	-0.016103308
G5E6P3	GNA13	G5E6P3	ENSBTAG00000046174	0.08503054	0.088143151	0.003112612
A0A452DJ27	AGFG1	A0A452DJ27	ENSBTAG00000021323	0.044870272	0.05700658	0.012136307
A0A3Q1NI16	HS1BP3	A0A3Q1NI16	ENSBTAG00000015490	0.287281952	0.250756076	-0.036525876
E1BAB5	GOLIM4	E1BAB5	ENSBTAG00000018727	0.043799463	-0.073794074	-0.117593537
A0AAA9TFK3	COG5	A0AAA9TFK3	ENSBTAG00000005526	0.011972642	-0.022889716	-0.034862357
A0A3S5ZPR2	MELK	A0A3S5ZPR2	ENSBTAG00000021686	-0.197065832	-0.337160085	-0.140094253
A0AAA9SUW0	OSBPL11	A0AAA9SUW0	ENSBTAG00000006471	-0.005012263	0.058826973	0.063839237
A0AAF6Z126	PHF6	A0AAF6Z126	ENSBTAG00000015196	0.205708396	0.27438907	0.068680674
F6R277	TRMT1	F6R277	ENSBTAG00000019325	-0.278002307	-0.172913223	0.105089084
A0A3S5ZP65	MEPCE	A0A3S5ZP65; bta:519969	ENSBTAG00000016763	0.228421803	0.092521868	-0.135899935
E1B777	WIPF2	bta:616849; E1B777	ENSBTAG00000038126	0.174909596	0.118929924	-0.055979672
Q0P5H2	DNTTIP2	bta:511258; Q0P5H2	ENSBTAG00000047679	0.079560378	-0.146841388	-0.226401766
A7YWC9	UTP25	A7YWC9; bta:510385; F1MDA2	ENSBTAG00000020886	0.064390587	0.039851911	-0.024538676
Q58DS5	RAB13	bta:514541; Q32KZ4; Q58DS5		-0.147142382	0.094583369	0.241725751
A5PJV9	DTYMK	A5PJV9; bta:506946		-0.141627341	-0.1291499	0.01247744
Q58D84	FSTL1	bta:534482; Q58D84	ENSBTAG00000022155	-0.29668065	-0.743224585	-0.446543935
A0A3Q1MRE3	AKAP13	A0A3Q1MRE3	ENSBTAG00000037383	-0.014797002	0.060284857	0.075081859
A0A3Q1M1V1	TOPBP1	A0A3Q1M1V1	ENSBTAG00000000526	0.040077439	0.034419805	-0.005657635
Q5E988	RPS5	bta:506229; Q3T0I8; Q5E988	ENSBTAG00000015989	0.001378591	-0.023639869	-0.02501846
F1N735	KDM2A	F1N735	ENSBTAG00000044032	0.073063462	-0.025654589	-0.098718051
Q9N285	MTCH2	A6QR21; bta:337927; F1MR38; Q5E9I9; Q9N285	ENSBTAG00000018742	-0.055948247	-0.002891173	0.053057074
Q0V8M0	KRI1	bta:511427; Q0V8M0	ENSBTAG00000010729	-0.091630475	0.097183666	0.188814141
F1MMU3	BRD3	F1MMU3	ENSBTAG00000010582	-0.022839976	-0.096840558	-0.074000581
A0A3Q1NDF8	NPC1	A0A3Q1NDF8	ENSBTAG00000015195	0.056853848	0.11804321	0.061189362
F1N6W2	PPIL4	F1N6W2	ENSBTAG00000016980	-0.145961287	0.023165985	0.169127271
A0A3Q1MFA8	GNL3L	A0A3Q1MFA8	ENSBTAG00000031564	-0.024595666	-0.288936269	-0.264340603
A0A3Q1MT88	BAX	A0A3Q1MT88	ENSBTAG00000013340	-0.039142256	-0.074488445	-0.03534619
A0AAA9S4Z4	AKT1	A0AAA9S4Z4	ENSBTAG00000017636	-0.126633439	-0.079754746	0.046878692
A0A140T872	DHFR	A0A140T872; bta:508809	ENSBTAG00000007681	-0.082724922	0.132215195	0.214940118
Q2HJD4	CSTF2	G3MZ78; Q2HJD4		0.125293733	-0.037474705	-0.162768439
F1MMV6	CAB39L	F1MMV6	ENSBTAG00000034222	0.011172912	0.013952672	0.00277976
P01045	KNG2	bta:280833; P01045; P01047		-0.267222203	-0.093574115	0.173648087
A0A452DIR5	TSFM	A0A452DIR5	ENSBTAG00000016912	0.07498116	0.215477153	0.140495993
A0A3Q1M9H9	NUP62	A0A3Q1M9H9; bta:100138517	ENSBTAG00000050948	0.039197888	-0.054506565	-0.093704453
Q2TBT5	RNASEH2A	bta:512830; Q2TBT5	ENSBTAG00000009661	-0.141972381	-0.137503524	0.004468858
A0A3Q1M647	CAMK2D	A0A3Q1M647	ENSBTAG00000014463	0.078168291	0.112080927	0.033912635
A0A3Q1MN47	ARHGEF12	A0A3Q1MN47	ENSBTAG00000021343	0.126416476	0.144389909	0.017973434
F2Z4J6	GTF2B	F2Z4J6	ENSBTAG00000000504	0.295192122	0.055871262	-0.23932086
F1MEM4	DHX8	F1MEM4	ENSBTAG00000008339	0.100075301	0.046208429	-0.053866872
Q76I81	RPS12	A1XED7; bta:326582; Q76I81; Q862U6	ENSBTAG00000001360	-0.00647821	-0.146841388	-0.140363178
A0A3Q1NAY5	DNAJC9	A0A3Q1NAY5; bta:510898	ENSBTAG00000000949	0.347675864	0.275107238	-0.072568626
A6QLS6	FRMD6	A6QLS6; bta:530910	ENSBTAG00000031707	-0.034692718	-0.002983858	0.03170886
E1BF95	PPM1F	E1BF95	ENSBTAG00000006938	-0.228950137	-0.26556028	-0.036610143
F6RT22	HABP4	F6RT22	ENSBTAG00000011586	0.132450296	0.064686969	-0.067763327
A0AAA9SG84	TBC1D23	A0AAA9SG84	ENSBTAG00000015010	0.324074431	0.311148256	-0.012926174
E1BCX3	ZSWIM8	E1BCX3	ENSBTAG00000012657	-0.133179219	0.038723432	0.171902651
Q2KIT5	CRELD2	bta:515222; Q2KIT5		0.35643877	0.203616196	-0.152822574
A0AAF6Z482	METTL13	A0AAF6Z482	ENSBTAG00000017721	-0.154498471	-0.075877507	0.078620964
F1MCX1	GPC4	bta:540179; F1MCX1	ENSBTAG00000020644	0.003901815	-0.175307285	-0.1792091
F1MNS0	HERC1	F1MNS0	ENSBTAG00000019474	-0.181756762	-0.054290156	0.127466606
A0A3Q1M6N5	BMP2K	A0A3Q1M6N5	ENSBTAG00000001126	0.148173266	0.056995366	-0.091177901
F1MSE5	ARMH3	F1MSE5	ENSBTAG00000013120	-0.096111893	0.058773709	0.154885602
A0AAA9SWH3	PATL1	A0AAA9SWH3	ENSBTAG00000005913	0.178786229	0.044812013	-0.133974216
A0A3Q1M011	STAU2	A0A3Q1M011	ENSBTAG00000002076	-0.060720381	-0.022074453	0.038645928
A7YWD2	IPO13	A7YWD2; bta:523399		0.0133378	0.045431429	0.03209363
A4FUF0	GLYR1	A4FUF0; bta:539636; Q58DC3	ENSBTAG00000001731	0.058469305	-0.094524505	-0.15299381
F1MJQ5	NDUFAF7	F1MJQ5	ENSBTAG00000001113	0.261651852	0.28633735	0.024685498
Q0VCY3	SURF6	bta:511610; Q0VCY3	ENSBTAG00000011554	-0.057240693	-0.139284037	-0.082043344
A0AAA9SDU1	CWC22	A0AAA9SDU1; bta:781016	ENSBTAG00000021726	-0.101753499	-0.028724289	0.07302921
A0AAF6Z059	RHOC	A0AAF6Z059	ENSBTAG00000014299	-0.13117429	0.102482958	0.233657247
A0AAA9U0Y4	OCRL	A0AAA9U0Y4	ENSBTAG00000018247	-0.117753531	-0.047565683	0.070187848
P55906	TGFBI	A7YWB6; bta:539596; P55906		-0.182034684	-0.49363564	-0.311600957
A0AAA9SZH9	TRIM56	A0AAA9SZH9; bta:514896	ENSBTAG00000012038	0.355828502	-0.001682443	-0.357510945
Q17QK8	SPR	A7YKV0; bta:533836; Q17QK8		0.093213162	0.079137977	-0.014075185
Q1LZB2	NCK1	bta:536154; F1MBQ7; Q1LZB2	ENSBTAG00000018427	0.076541555	0.058893689	-0.017647866
Q3ZBL4	LZTFL1	bta:512570; Q3ZBL4	ENSBTAG00000001484	-0.16083012	-0.147007786	0.013822334
Q3MI03	OGFOD1	bta:514141; Q3MI03		-0.153385647	-0.256581605	-0.103195957
A0AAA9T5H0	MPP1	A0AAA9T5H0	ENSBTAG00000013046	-0.057947349	-0.008545109	0.04940224
A5D7C2	SDAD1	A5D7C2; bta:507675		-0.454188627	-0.335360511	0.118828116
Q2NKU1	DUT	bta:507945; Q2NKU1	ENSBTAG00000011998	-0.093553516	-0.247927513	-0.154373998
A0A3Q1M3K3	SMAD5	A0A3Q1M3K3; bta:768050	ENSBTAG00000055062	-0.099445361	-0.321612026	-0.222166665
A0A452DIH1	MRPL44	A0A452DIH1	ENSBTAG00000011514	-0.051791209	-0.214803266	-0.163012057

Accession	Gene_Symbol	Gene_ID	Ensembl_Gene ID	log2(ratio(CM12H/CM0H))	log2(ratio(CM12R/CM0H))	log2(ratio(CM12H/CM12R))
P17697	CLU	A5D983; bta:280750; P17697; Q148K1	ENSBTAG00000005574	0.007054758	-0.199937571	-0.206992328
A0A3Q1LLL1	SH3GLB2	A0A3Q1LLL1	ENSBTAG00000010626	0.141868716	0.07952034	-0.062348377
P11456	M6PR	A1L5A0; bta:281291; P11456; Q0VCN7	ENSBTAG00000018207	0.085960816	0.192269718	0.106308902
A0AAA9SXY1	CHAF1A	A0AAA9SXY1	ENSBTAG00000008181	-0.246437895	-0.105082046	0.141355849
F6RDU5	SRSF11	F6RDU5	ENSBTAG00000015474	0.097327185	-0.05768735	-0.155014535
A0A3Q1MW87	JUND	A0A3Q1MW87	ENSBTAG00000052791	-0.019973024	-0.094255905	-0.074282882
A2VE70	VAC14	A2VE70; bta:533202		-0.103281209	-0.042863968	0.060417241
Q58DT8	WDR55	A1L559; bta:508709; Q58DT8	ENSBTAG00000015042	0	-0.056143078	-0.056143078
A0A3Q1M489	SNRPB	A0A3Q1M489	ENSBTAG00000001038	-0.224317298	0.070389328	0.294706626
A0A3Q1LXW0	TRIM33	A0A3Q1LXW0; bta:533296	ENSBTAG00000001499	-0.075442556	0.031364171	0.106806727
P61284	RPL12	A5PJB1; bta:404133; P61284; Q3SZV1	ENSBTAG00000006963	-0.187066134	-0.169925001	0.017141133
Q2YDJ9	GMPPB	bta:514161; Q2YDJ9		0.183678157	0.129283017	-0.05439514
F6RBQ9	RBM3	F6RBQ9	ENSBTAG00000025848	-0.159090386	-0.189865139	-0.030774753
A0AAA9U1E9	GALNS	A0AAA9U1E9	ENSBTAG00000004568	0.003122718	-0.03160466	-0.034727378
F1MWJ3	ADPRS	F1MWJ3	ENSBTAG00000021715	-0.330645312	-0.32752597	0.003119342
A4FV55	PPP2R5C	A4FV55; bta:505757	ENSBTAG00000020192	-0.265131855	-0.095051382	0.170080473
F1MD32	CREBBP	A0A8J8YPW6; F1MD32; M5FJS5	ENSBTAG00000026403	-0.160235945	-0.202865798	-0.042629853
G3N3E5	WDR5	G3N3E5	ENSBTAG00000047031	-0.065997559	0.084008937	0.150006496
E1B782	HERC2	bta:535440; E1B782	ENSBTAG00000013916	-0.126325974	-0.059501012	0.066824963
E1BA60	RAB32	E1BA60	ENSBTAG00000009784	0.047456317	0.125141753	0.077685436
Q3SZJ2	RIPK2	bta:534407; Q3SZJ2	ENSBTAG00000015271	0.09302599	0.089267338	-0.003758652
A0AAA9TCA1	PUDP	A0AAA9TCA1	ENSBTAG00000000207	-0.091358393	-0.089885504	0.001472889
A0AAA9TL09	CTNNBL1	A0AAA9TL09	ENSBTAG00000007658	0.046105081	-0.034866246	-0.080971327
P62992	RPS27A	bta:286839; O97577; P02248; P02249; P02250; P0CG52; P62990; P62992; P80169; Q01235; Q24K23; Q28169; Q28170; Q29120; Q3T0V5; Q3ZCE3; Q862C1; Q862F4; Q862M4; Q862T5; Q862X8; Q91887; Q91888	ENSBTAG00000015473	0.019844811	-0.185999764	-0.205844576
A0AAF6YM07	BPNT1	A0AAF6YM07	ENSBTAG00000004064	0.102978464	0.194728392	0.091749929
F1MBE0	CWC27	F1MBE0	ENSBTAG00000000037	-0.086926756	-0.192440831	-0.105514076
Q32KL5	EMC8	bta:510727; Q32KL5		0.013508479	0.084345919	0.070837439
A0AAA9RT28	STAG1	A0AAA9RT28; bta:539234	ENSBTAG00000035437	0.050675475	-0.024563253	-0.075238728
Q1LZ90	CLNS1A	bta:613968; F1N1B1; Q1LZ90	ENSBTAG00000019930	0.142695351	0.138487195	-0.004208156
Q58DU7	SH3BGRL	bta:614007; Q58DU7		-0.104697379	0.315501826	0.420199204
A0AAA9SW30	CADM1	A0AAA9SW30	ENSBTAG00000000977	0.053648463	0.050874048	-0.002774414
Q05B85	JPT2	A0A8J8XE70; bta:618512; M5FK16; Q05B85	ENSBTAG00000002209	0.086398575	-0.209236004	-0.295634579
A0AAA9S8T0	UBE2K	A0AAA9S8T0	ENSBTAG00000020175	0.166480992	0.220622243	0.054141252
A0AAA9SWA1	STK26	A0AAA9SWA1	ENSBTAG00000019652	-0.093574115	0.006952761	0.100526876
F1N444	LTBP1	F1N444	ENSBTAG00000019839	-0.026717338	-0.122268613	-0.095551274
F1MV85	PBDC1	bta:511011; F1MV85	ENSBTAG00000003609	0.024662054	0.053146593	0.028484539
A0AAA9S5H7	CELF2	A0AAA9S5H7	ENSBTAG00000008937	-0.020117588	-0.047876627	-0.027759039
Q32KP9	NUTF2	bta:614921; Q32KP9		0.026415187	0.308061981	0.281646794
E1BJC0	CARNMT1	bta:538441; E1BJC0	ENSBTAG00000008204	-0.016906776	-0.008428622	0.008478154
A0AAF7A0P8	CHID1	A0AAF7A0P8	ENSBTAG00000009899	-0.090286036	-0.05292332	0.037362717
A0A3Q1M902	HPF1	A0A3Q1M902	ENSBTAG00000020132	-0.073006858	-0.086827353	-0.013820495
F2Z4G3	RBM22	F2Z4G3	ENSBTAG00000030366	0.265473451	0.083926082	-0.181547369
A0AAA9S0Y0	IGF2BP3	A0AAA9S0Y0	ENSBTAG00000019406	-0.111413534	-0.057390559	0.054022975
E1BMW9	PURA	bta:782746; E1BMW9	ENSBTAG00000008881	0.096266288	0.078418286	-0.017848001
A0AAA9T3W1	NIF3L1	A0AAA9T3W1	ENSBTAG00000018282	0.122152643	0.118012913	-0.00413973
A0A3Q1MK61	UEVLD	A0A3Q1MK61	ENSBTAG00000013568	-0.014455983	-0.031996564	-0.017540581
P35705	PRDX3	bta:281998; P35705; Q3SZA8	ENSBTAG00000008731	0.184869877	0.499221806	0.314351929
E1BD67	SAAL1	E1BD67	ENSBTAG00000008804	0.133573026	0.130856085	-0.002716941
A0AAA9TSJ7	SEC13	A0AAA9TSJ7	ENSBTAG00000017825	0.034086831	0.166922513	0.132835683
F1MDT8	SETD2	F1MDT8	ENSBTAG00000002948	-0.080049	0.0377288	0.1177778
A6QQV4	METTL3	A6QQV4; bta:540339		0.016950069	-0.015824967	-0.032775036
A0AAA9TPZ1	NUDT5	A0AAA9TPZ1	ENSBTAG00000019509	0.133812978	0.051441385	-0.082371593
A0A3Q1LVP1	PTK2B	A0A3Q1LVP1	ENSBTAG00000005958	-0.127540281	-0.011672354	0.115867927
A4FUE3	RBBP5	A4FUE3; bta:510455		0.161362988	0.096307104	-0.065055884
F1MQB8	SIRT1	F1MQB8	ENSBTAG00000014023	-0.229285614	0.073502524	0.302788138
A0AAA9SNN6	SARNP	A0AAA9SNN6	ENSBTAG00000020662	0.16539753	0.14714421	-0.018253319
A0AAA9SGI8	COPS7A	A0AAA9SGI8	ENSBTAG00000011111	-0.153579731	-0.198735912	-0.045156181
G3MYD5	IVNS1ABP	bta:514940; G3MYD5	ENSBTAG00000014249	-0.309113749	-0.299644549	0.0094692
A0AAA9S4Z0	RALY	A0AAA9S4Z0	ENSBTAG00000016524	-0.005591848	-0.018253319	-0.012661471
A6QLS9	RAB10	A6QLS9; bta:783373	ENSBTAG00000000985	0.150678912	0.157476075	0.006797162
Q0P5G8	ACAD11	bta:526956; F1MNX2; Q0P5G8	ENSBTAG00000031010	-0.023699835	-0.049314937	-0.025615102
A0AAA9SYT0	UPF3B	A0AAA9SYT0	ENSBTAG00000035615	0.351558442	0.192047812	-0.15951063
A0AAF6YWB5	IMPA1	A0AAF6YWB5	ENSBTAG00000011709	-0.04580369	-0.039998068	0.005805622
A0A3Q1M792	SUZ12	A0A3Q1M792; bta:538774	ENSBTAG00000018752	-0.093627521	-0.177553603	-0.083926082
A0A3Q1LJH2	PRUNE1	A0A3Q1LJH2	ENSBTAG00000015336	-0.0439115	0.00308598	0.04699748
A0AAA9S140	MADD	A0AAA9S140	ENSBTAG00000021700	0.04324085	0.037549738	-0.005691112
A0AAA9U0N9	ABI1	A0AAA9U0N9	ENSBTAG00000005136	0.192230451	0.142019005	-0.050211446
F1MR28	OXR1	F1MR28	ENSBTAG00000008285	0.043670472	0.05684586	0.013175389
F1MUP3	SEC62	bta:538938; F1MUP3	ENSBTAG00000039442	0.173616907	-0.018262206	-0.191879113
A0AAA9U078	C15H11orf58	A0AAA9U078	ENSBTAG00000006755	0.049848549	0.130570563	0.080722013
F1MFW8	NOC4L	F1MFW8	ENSBTAG00000011889	-0.180887107	-0.333187633	-0.152300526
Q17QW1	CDC42EP1	bta:511099; Q17QW1	ENSBTAG00000030258	0.177698708	0.045040964	-0.132657744
F6RJG0	HMGCS1	F6RJG0	ENSBTAG00000011839	0.002378723	-0.159694656	-0.162073379
A0A3Q1M809	MED24	A0A3Q1M809	ENSBTAG00000021468	-0.140619643	-0.103795412	0.036824231
A5PKH0	TOR1AIP2	A5PKH0; bta:782450; F1N6U6	ENSBTAG00000035230	0.279154071	0.16089119	-0.118262882
F1MXY9	SRSF6	F1MXY9	ENSBTAG00000008527	-0.044565552	0.008204923	0.052770475
A0A452DJR2	JMJD6	A0A452DJR2	ENSBTAG00000019153	-0.008885963	-0.090075344	-0.081189381
E1BNH9	OBSL1	E1BNH9	ENSBTAG00000040338	-0.169236857	-0.28690868	-0.117671824
Q0P5F2	PSMG1	bta:614817; Q0P5F2	ENSBTAG00000013600	-0.162553013	-0.005924834	0.156628179
Q3T0P5	CASP6	bta:538409; Q3T0P5		-0.173211859	-0.061318979	0.11189288
A0AAA9T728	ANK2	A0AAA9T728	ENSBTAG00000002392	-0.118980437	0.010042001	0.129022438
A0AAA9TQU6	CORO2A	A0AAA9TQU6	ENSBTAG00000007659	-0.014025427	-0.025603068	-0.011577642
A0A3Q1MNS5	UBE2V2	A0A3Q1MNS5	ENSBTAG00000023218	0.011416044	0.053439259	0.042023215

Accession	Gene_Symbol	Gene_ID	Ensembl_Gene ID	log2(ratio(CM12H/CM0H))	log2(ratio(CM12R/CM0H))	log2(ratio(CM12H/CM12R))
A0AAA9RRZ7	WDR77	A0AAA9RRZ7	ENSBTAG00000014102	-0.040806216	0.00573637	0.046542586
A0A3Q1LJG6	AOX1	A0A3Q1LJG6	ENSBTAG00000009725	-0.276951186	-0.251538767	0.025412419
A6QLZ3	ARSB	A6QLZ3; bta:538401	ENSBTAG00000008341	-0.006886386	-0.019365325	-0.012478939
A0AAA9TJZ3	NFX1	A0AAA9TJZ3	ENSBTAG00000012385	-0.039241632	-0.178337241	-0.13909561
A0A3Q1LLQ5	RBM47	A0A3Q1LLQ5; bta:540831	ENSBTAG00000002356	-0.032180406	-0.010647244	0.021533162
E1BE36	ARMCX3	bta:516747; E1BE36	ENSBTAG00000037996	0.306207637	0.246355665	-0.059851972
Q0VCJ2	APIP	bta:508345; Q0VCJ2	ENSBTAG00000018257	-0.096985615	-0.022507399	0.074478216
A0AAA9T3B8	CD2BP2	A0AAA9T3B8	ENSBTAG00000015486	-0.14974712	-0.041222663	0.108524457
G1K1U4	POLD3	G1K1U4	ENSBTAG00000016869	-0.289251183	-0.136124713	0.153126469
A0JNF4	PRIM2	A0JNF4; bta:514682; F1MP18	ENSBTAG00000007937	-0.03656016	-0.118377108	-0.081816948
Q08DA3	USP16	bta:519992; F1N2B1; Q08DA3		-0.249081024	-0.275634443	-0.026553419
Q08DJ7	AAR2	Q08DJ7	ENSBTAG00000001645	0.185785242	-0.017866421	-0.203651662
F1MZA7	URB1	bta:530483; F1MZA7	ENSBTAG00000012412	-0.32223666	-0.294726811	0.027509849
Q0P5J0	QRICH1	A5D7N1; bta:511970; Q0P5J0		-0.051002903	-0.063351296	-0.012348392
Q2KI46	CDC42EP3	bta:538967; Q2KI46	ENSBTAG00000023736	0.185193803	0.091469046	-0.093724757
E1BCU7	METTL16	E1BCU7	ENSBTAG00000017440	-0.201897391	-0.249657808	-0.047760417
A8E4R4	EXOSC6	A8E4R4; bta:100126247	ENSBTAG00000053923	0.085004355	0.037232906	-0.047771449
A0A3Q1MXF1	THYN1	A0A3Q1MXF1	ENSBTAG00000012936	-0.116193018	-0.031836087	0.08435693
A7MB43	MTMR9	A7MB43; bta:539174		-0.016759473	0.099257938	0.116017411
Q29RM7	FAF1	bta:531770; Q29RM7	ENSBTAG00000014482	0.053538427	-0.02060047	-0.074138896
F1MHR4	PIP4K2A	bta:533289; F1MHR4	ENSBTAG00000018033	0.050282942	0.077478753	0.027195811
A5PK76	ASPSCR1	A5PK76; bta:515670		-0.16089119	-0.116202372	0.044688818
A8PUI7	SKA3	A8PUI7; bta:509921	ENSBTAG00000003314	-0.09064068	0.040603854	0.131244533
A0AAA9TVR1	MICU1	A0AAA9TVR1	ENSBTAG00000005807	0.00805977	0.018736563	0.010676793
A0AAA9TQM8	PUS1	A0AAA9TQM8	ENSBTAG00000012990	-0.209968933	-0.121729312	0.088239621
A0A3Q1N5S1	RFC1	A0A3Q1N5S1	ENSBTAG00000014515	0.16112099	-0.047906385	-0.209027375
G3MZ11	CRYBG3	G3MZ11	ENSBTAG00000047147	-0.020444769	-0.136150786	-0.115706017
A0A452DIE3	APRT	A0A452DIE3	ENSBTAG00000000639	0.004270437	0.099155467	0.094885029
F1MKC7	HELZ	F1MKC7	ENSBTAG00000004746	0.073284426	0.090197809	0.016913383
F1N270	ATP6V1D	bta:404152; F1N270	ENSBTAG00000016309	0.177888737	0.125823014	-0.052065723
A0A3Q1MKV8	PLEKHO2	A0A3Q1MKV8	ENSBTAG00000008411	-0.002971567	0.127755547	0.130727114
A0AAA9SZH8	GALK2	A0AAA9SZH8	ENSBTAG00000004011	0.003174248	0.060672128	0.05749788
F1MSA1	PPT1	F1MSA1	ENSBTAG00000013367	-0.024504964	-0.115350671	-0.090845707
Q3ZC35	CCN1	bta:508941; Q3ZC35	ENSBTAG00000009844	-0.84165538	-0.987344699	-0.14568932
A0AAA9SJP4	DHRS7	A0AAA9SJP4	ENSBTAG00000020729	0.061656591	0.057233404	-0.004423187
P00921	CA2	bta:280740; P00921; Q3ZBJ5; Q865Y7		0.376375879	0.467662548	0.091286669
A0A3Q1M8C6	VIPAS39	A0A3Q1M8C6	ENSBTAG00000020475	0.144958086	0.14974712	0.004789034
F6Q4T4	REPS1	F6Q4T4	ENSBTAG00000012299	0.114257179	0.115721101	0.001463922
F1MC10	P3H4	F1MC10	ENSBTAG00000025752	0.001309755	-0.147496712	-0.148806467
A0AAA9S957	NRBP1	A0AAA9S957	ENSBTAG00000018153	-0.084236537	0.004300137	0.088536675
Q3T0V3	EIF3K	bta:515326; Q3T0V3	ENSBTAG00000011212	0.114581968	0.134591928	0.020009961
A0AAA9S3E1	FBXO3	A0AAA9S3E1	ENSBTAG00000010071	-0.098180394	-0.105455725	-0.007275331
A0AAA9U053	EP400	A0AAA9U053	ENSBTAG00000020423	-0.038780599	-0.071168991	-0.032388392
A0AAA9S711	PSMA3	A0AAA9S711	ENSBTAG00000002808	0.095899918	0.147246798	0.05134688
F1MUZ0	EBNA1BP2	F1MUZ0	ENSBTAG00000021544	0.00135911	-0.078229155	-0.079588265
A0AAA9T3V8	PLPBP	A0AAA9T3V8	ENSBTAG00000011075	0.17055349	0.121990524	-0.048562965
E1BCT7	WDR33	bta:541270; E1BCT7	ENSBTAG00000005987	-0.155468869	-0.092789906	0.062678963
E1BFK4	SMG6	E1BFK4	ENSBTAG00000000267	-0.195550809	-0.183075296	0.012475514
F1MUX7	GTF2E2	F1MUX7	ENSBTAG00000012691	0.259143578	0.05119343	-0.207950148
Q3MHR5	SRSF2	bta:508312; Q3MHR5	ENSBTAG00000018258	-0.014383918	-0.00573637	0.008647549
Q0X0C4	HEXIM1	bta:539696; Q0X0C4	ENSBTAG00000006056	0.060282049	0.155471491	0.095189442
P02316	HMGN1	bta:614915; P02316; Q3T0D1		0.153111215	0.115599031	-0.037512184
A0AAA9SIT5	IKBKB	A0AAA9SIT5	ENSBTAG00000007599	-0.066295824	0.102089974	0.168385798
E1BGU0	HEATR5A	E1BGU0	ENSBTAG00000022847	-0.069881426	-0.060769638	0.009111788
Q3SX42	CHMP2B	bta:615954; Q3SX42		0.236479254	0.158872684	-0.07760657
A0A3Q1MJG1	TTC4	A0A3Q1MJG1	ENSBTAG00000015931	0.147557188	0.106915204	-0.040641984
F1MUZ3	SMAD3	bta:515125; F1MUZ3	ENSBTAG00000012599	0.168642036	0.172060746	0.00341871
A6QPZ6	TAP1	A6QPZ6	ENSBTAG00000008953	0.039183263	-0.132122969	-0.171306232
O18739	CCN2	bta:281103; O18739; Q2HJ71; Q9GL71	ENSBTAG00000006367	-0.683772655	-0.595158268	0.088614387
A0AAA9TKV8	PDAP1	A0AAA9TKV8	ENSBTAG00000044040	0.100852602	0.023800458	-0.077052144
Q5E9L7	VPS16	bta:505361; Q5E9L7		-0.180380665	-0.122920614	0.057460052
A0AAA9TVL3	RNF31	A0AAA9TVL3	ENSBTAG00000005815	-0.112080927	-0.054952311	0.057128616
A0AAF7AI76	ALDH3B1	A0AAF7AI76	ENSBTAG00000013093	-0.231882863	-0.130198723	0.10168414
A0AAA9TCR7	RAB18	A0AAA9TCR7	ENSBTAG00000009871	0.099025617	-0.112929636	-0.211955253
A0AAA9TDL0	C11H9orf78	A0AAA9TDL0	ENSBTAG00000020307	0.096171369	-0.012313261	-0.10848463
Q5E948	Ociad1	bta:533520; Q3ZBB3; Q5E948		0.2143786	0.040641984	-0.173736615
E1BJS0				-0.19175469	-0.177582719	0.014171971
A0A3Q1MIC9	DNM3	A0A3Q1MIC9	ENSBTAG00000014319	0.131762836	0.071443367	-0.060319469
A0A3Q1LLT2	EXOSC8	A0A3Q1LLT2	ENSBTAG00000011855	0.053222857	-0.095668235	-0.148891092
G3N315	STAM2	G3N315	ENSBTAG00000001887	-0.025116677	0.041862023	0.0669787
Q0V8R6	HEXA	bta:504468; Q0V8R6	ENSBTAG00000012981	0.02187048	0.034835375	0.012964895
A0JN63	CSTF1	A0JN63; bta:507051	ENSBTAG00000006639	0.051208941	0.14974712	0.098538179
A5PKK7	GORAB	A5PKK7		0.146289442	0.12683708	-0.019452363
A0AAA9TRT3	MORC2	A0AAA9TRT3	ENSBTAG00000009963	0.08165722	0.011541622	-0.070115598
E1BMI6	GNL3	E1BMI6	ENSBTAG00000014549	-0.506599315	-0.281658985	0.22494033
A0AAA9RYW7	SPART	A0AAA9RYW7	ENSBTAG00000008040	0.503726506	0.286228352	-0.217498154
A0AAA9SJA2	STK38	A0AAA9SJA2	ENSBTAG00000011126	0.066700401	0.20604628	0.139345878
P11116	LGALS1	bta:326598; P11116; P11945; Q54A27	ENSBTAG00000015089	0.107929559	0.234070589	0.12614103
P29105	HPCAL1	A5PKL3; bta:513870; P29105; P29106	ENSBTAG00000004259	0.029374602	0.218800783	0.189426181
A0A3Q1LT47	ANKRD44	A0A3Q1LT47	ENSBTAG00000031696	-0.198024415	-0.083312756	0.114711659
A0A140T8D6	BCAR3	A0A140T8D6	ENSBTAG00000003245	-0.024401343	-0.029022901	-0.004621558
A0A3Q1LQW5	SLC38A10	A0A3Q1LQW5	ENSBTAG00000018271	0.093936401	-0.156749613	-0.250686013
Q1JQE6	NCEH1	bta:534212; Q1JQE6	ENSBTAG00000020073	-0.023400433	0.05087233	0.074272762
A0A452DI54	CCDC25	A0A452DI54	ENSBTAG00000006547	-0.013533824	-0.10329307	-0.089759247
E1BGR7				-0.232875825	-0.343907138	-0.111031312
Q3ZBK3	SPC25	bta:505011; Q3ZBK3		-0.078002512	-0.131493919	-0.053491407
E1BHF6	SIPA1L3	E1BHF6	ENSBTAG00000018225	-0.219117289	-0.188902676	0.030214613
A4FV54	RAB8A	A4FV54; bta:100125881	ENSBTAG00000038696	0.138744196	0.16604293	0.027298734
Q05588	PLAUR	A5PJ83; bta:281983; Q05588	ENSBTAG00000013125	-0.347629266	-0.628472391	-0.280843125
Q8MJ50	OSTF1	bta:281961; Q8MJ50	ENSBTAG00000002746	0.073497813	-0.001676578	-0.07517439

Accession	Gene_Symbol	Gene_ID	Ensembl_Gene ID	log2(ratio(CM12H/CM0H))	log2(ratio(CM12R/CM0H))	log2(ratio(CM12H/CM12R))
O97764	CRYZ	bta:281093; O97764; Q0VCY2	ENSBTAG00000003162	0.05635485	0.137863792	0.081508942
A0AAA9TAV4	ANO6	A0AAA9TAV4	ENSBTAG00000002902	-0.0862782	-0.061331843	0.024946357
F6RBN9	MTX2	F6RBN9	ENSBTAG00000006693	-0.033434228	0.063255409	0.096689636
F1N063	CAMK1D	F1N063	ENSBTAG00000008650	0.099679004	0.13730805	0.037629046
Q0VCZ3	YTHDF2	bta:541050; Q0VCZ3	ENSBTAG00000015771	-0.00138388	0.002763784	0.004147665
A0AAA9T0P5	PNPLA6	A0AAA9T0P5	ENSBTAG00000032137	0.300946822	0.190081805	-0.110865017
A0AAA9TIR9	SOS1	A0AAA9TIR9; bta:537682	ENSBTAG00000011643	0.060360588	0.072972864	0.012612276
A0A3Q1MS93	CDC27	A0A3Q1MS93	ENSBTAG00000002726	-0.072595131	-0.129909323	-0.057314192
A4FV10	TMED4	A4FV10; bta:508729		0.077927256	0.181160878	0.103233622
A0AAA9SHQ6	GPALPP1	A0AAA9SHQ6	ENSBTAG00000009416	0.125530882	0.119121742	-0.00640914
A1A4J9	DNAJA3	A0A8J8Y340; A1A4J9; bta:513397; M5FJZ6	ENSBTAG00000010013	0.073080924	-0.102569734	-0.175650658
E1BP85	UCKL1	E1BP85	ENSBTAG00000015798	-0.01809482	-0.00138388	0.01671094
A0AAA9TMM9	NEK7	A0AAA9TMM9	ENSBTAG00000016943	0.035441579	0.01782962	-0.017611959
P21146	GRK2	bta:282682; P21146	ENSBTAG00000005832	0.105610188	-0.0061002	-0.111710387
A0A3Q1MDB6	HDAC7	A0A3Q1MDB6	ENSBTAG00000026819	-0.054261594	0.014451156	0.06871275
Q0VC53	DOHH	A5H2K6; A5H2K8; bta:526521; Q0VC53	ENSBTAG00000005272	0	0.216883001	0.216883001
A0AAF6Z2G3	GAA	A0AAF6Z2G3	ENSBTAG00000016021	0.13289427	0.152545358	0.019651088
A0AAA9S6V6	HSPE1	A0AAA9S6V6	ENSBTAG00000012589	0.255175051	0.424111083	0.168936032
F1MF02	SLAIN2	F1MF02	ENSBTAG00000021963	0.032330654	-0.143099869	-0.175430523
A7E3Q1	TARSL1	A7E3Q1; bta:511740		-0.111938596	0.050137306	0.162075902
F6Q0E0				-0.173780036	-0.207074476	-0.03329444
A0A3Q1LPQ8	ARVCF	A0A3Q1LPQ8	ENSBTAG00000001473	0.084888898	0.056507096	-0.028381801
Q2HJ47	DENR	bta:614238; Q2HJ47	ENSBTAG00000068852	0.017977741	0.034262825	0.016285084
E1BAZ9	EXOG	bta:524343; E1BAZ9	ENSBTAG00000019305	0.106808191	0.06874759	-0.038060601
A0A3Q1M9L7	MAP4K5	A0A3Q1M9L7	ENSBTAG00000014792	-0.041242982	-0.002806801	0.038436182
A0A3Q1M337	NDC1	A0A3Q1M337	ENSBTAG00000006625	0.010613674	0.021149835	0.010536161
A0AAF6DM24	GRSF1	A0AAF6DM24	ENSBTAG00000008577	-0.056894935	-0.204555941	-0.147661006
F1MPI4	POFUT2	F1MPI4	ENSBTAG00000007818	-0.276359034	-0.287006279	-0.010647244
Q3SZ21	RPP30	bta:615098; Q3SZ21	ENSBTAG00000002973	-0.305448161	-0.155048953	0.150399208
F6QVL1	POLR1G	F6QVL1	ENSBTAG00000023601	0.070313078	0.056521942	-0.013791136
Q5BIR6	MED17	bta:541303; Q3SYX5; Q5BIR6	ENSBTAG00000003552	-0.056855504	-0.072498482	-0.015642978
A0A3Q1NA18	AVEN	A0A3Q1NA18	ENSBTAG00000044029	0.075634438	-0.181208134	-0.256842572
A0A3Q1LN00	CBL	A0A3Q1LN00	ENSBTAG00000006817	0.183424191	0.118528389	-0.064895802
E1BK63	LOC101903301	E1BK63	ENSBTAG00000040367	-0.164475419	-0.158727623	0.005747797
F1MSM5	MTPAP	bta:511412; F1MSM5	ENSBTAG00000004387	0.130357917	0.240646174	0.110288257
G5E683	MBD2	G5E683	ENSBTAG00000037457	0.105808881	0.011216344	-0.094592537
A0A3Q1M7Q3	GIT1	A0A3Q1M7Q3	ENSBTAG00000012040	0.100164162	0.100164162	0
F6PRJ0	LRRC8E	bta:507824; F6PRJ0	ENSBTAG00000004300	0.02282277	0.193466424	0.170643654
E1BEM3	CDV3	E1BEM3	ENSBTAG00000035556	0.084256851	-0.011958464	-0.096215315
A0AAF6YXD6	CNBP	A0AAF6YXD6	ENSBTAG00000012159	-0.1260968	-0.068834197	0.057262603
A0AAA9S3N0	SERPINE2	A0AAA9S3N0	ENSBTAG00000008717	-0.235610805	-0.033921583	0.201689223
A0A3Q1M9B3	RPL18	A0A3Q1M9B3	ENSBTAG00000015388	-0.090154437	0.016044967	0.106199404
A0AAA9SVT1	RPL32	A0AAA9SVT1	ENSBTAG00000015283	-0.047488926	-0.026344995	0.021143931
F1MKA6	AARS2	F1MKA6	ENSBTAG00000013722	-0.440572591	-0.259801614	0.180770978
A0AAF6DLE4	HAUS5	A0AAF6DLE4	ENSBTAG00000013436	-0.005894574	0.036305264	0.042199838
Q2KJ61	ELP3	bta:784720; Q2KJ61		-0.018262206	-0.057067532	-0.038805326
P62248	MYDGF	bta:407769; P62248; Q3T039	ENSBTAG00000018655	0.097736802	0.058138436	-0.039598366
A0AAF6Z8G6	CTSK	A0AAF6Z8G6; bta:513038	ENSBTAG00000021035	-0.557446229	-0.290029883	0.267416346
Q0VCH0	ANP32E	bta:507203; F1MPJ7; Q0VCH0	ENSBTAG00000016730	-0.25464426	-0.219454832	0.035189428
Q3T083	SDF2L1	bta:517962; Q3T083		0.170133982	0.051728283	-0.118405699
Q2KHT8	DIMT1	bta:509725; Q2KHT8		-0.074663568	-0.281908163	-0.207244595
A0A3Q1MH41	SRPK2	A0A3Q1MH41	ENSBTAG00000002175	0.083543641	0.14059833	0.057054689
A0AAA9SH20	GMPR2	A0AAA9SH20	ENSBTAG00000002715	0.04940762	0.045464032	-0.003943588
A0A3Q1N340	TRMT1L	A0A3Q1N340	ENSBTAG00000011285	0.128448365	0.059501012	-0.068947354
A5D7R9	NRAS	A5D7R9; bta:506322	ENSBTAG00000046797	0.214399631	-0.231186932	-0.445586563
A5PK21	CKAP2L	A5PK21; bta:507498; Q0V8I3		0.102759574	0.307749843	0.204990268
A0AAA9S5X5	HM13	A0AAA9S5X5	ENSBTAG00000008840	0.078002512	-0.042892094	-0.120894606
A2VDK6	WASF2	A2VDK6; bta:504482	ENSBTAG00000018374	-0.053589657	0.068490295	0.122079952
A0AAF6YYF2	COG4	A0AAF6YYF2	ENSBTAG00000012988	-0.022346159	-0.04647443	-0.024128271
A0AAA9S0R6	GLIPR2	A0AAA9S0R6	ENSBTAG00000000078	-0.045037616	-0.04994753	-0.004909914
Q08DV2	GNPNAT1	A0A3Q1MLH0; bta:512299; F1N5F5; Q08DV2	ENSBTAG00000005344	0.002831591	0.021102693	0.018271102
A0AAA9SJN2	PARVA	A0AAA9SJN2	ENSBTAG00000000700	-0.065821123	-0.139506671	-0.073685548
A0AAA9S2K0	CCNY	A0AAA9S2K0	ENSBTAG00000005492	0.901281949	0.025722565	-0.875559384
Q32PB8	RPS21	bta:615178; Q32PB8		-0.105645039	-0.083596291	0.022048749
A0AAA9STD7	POLR3A	A0AAA9STD7	ENSBTAG00000013259	-0.116675171	0.094545637	0.211220807
A0A3Q1M4E6	TJAP1	A0A3Q1M4E6	ENSBTAG00000011189	0.105825473	0.077199632	-0.028625841
F1MNU7	YTHDC2	bta:541024; F1MNU7	ENSBTAG00000014099	-0.261927195	-0.189089207	0.072837988
F1MT65	HDGFL2	F1MT65	ENSBTAG00000013251	-0.059786722	-0.042030251	0.017756471
F1MM80	ASPM	F1MM80	ENSBTAG00000007860	0.004312993	0.085259088	0.080946095
Q58DR5	SCAMP3	bta:539670; Q3ZCL2; Q58DR5		-0.061230566	0.010388488	0.071619054
A0AAA9SM15	ADAM17	A0AAA9SM15; bta:517541	ENSBTAG00000001141	0.153638212	0.097749791	-0.055888421
A0A3Q1N363	NIPSNAP1	A0A3Q1N363	ENSBTAG00000013152	0.082554709	0.130520034	0.047965325
A0AAA9SD78	EXOSC7	A0AAA9SD78; bta:100847343	ENSBTAG00000018329	0.071191048	0.041499363	-0.029691685
A0AAF6ZJK8	MRPL38	A0AAF6ZJK8	ENSBTAG00000022009	-0.09064068	-0.063132364	0.027508315
A0AAA9SUN6	SVIL	A0AAA9SUN6	ENSBTAG00000027444	0.029179097	0.098115752	0.068936655
A0AAF6DMH4	LANCL2	A0AAF6DMH4	ENSBTAG00000018026	0.074152756	0.016643756	-0.057509
E1BP14	ARHGEF17	E1BP14	ENSBTAG00000012505	-0.148205372	-0.056372931	0.091832441
Q32LC3	EIF1AX	Q32LC3	ENSBTAG00000017633	0.203091865	0.011140558	-0.191951308
A3KN48	EEFSEC	A3KN48; bta:532824	ENSBTAG00000030962	-0.039528364	-0.188050889	-0.148522525
A0AAA9RUC6	U2AF1	A0AAA9RUC6	ENSBTAG00000011645	-0.00141649	0.001415101	0.002831591
A0AAA9S7F8	GNA13	A0AAA9S7F8	ENSBTAG00000046174	-0.358761987	-0.053011245	0.305750741
A0AAA9RSE0	MAN1A1	A0AAA9RSE0	ENSBTAG00000024381	-0.21739174	-0.331621491	-0.114229751
F1N3H6	DPY19L1	F1N3H6	ENSBTAG00000005785	0.155343219	0.098473697	-0.056869522
E1BNM2	ITGA8	bta:511976; E1BNM2	ENSBTAG00000007602	0.030696797	-0.217230716	-0.247927513
Q0VD18	LHPP	bta:534183; Q0VD18	ENSBTAG00000010957	-0.038573705	-0.086005535	-0.04743183
F1N1J5	CEP43	F1N1J5	ENSBTAG00000014495	-0.117569596	-0.016401607	0.101167989
Q2KJ44	PTPA	bta:514460; Q2KJ44		-0.026497802	0.00413973	0.030637533
A0A3Q1LSG0				0.220342265	0.247172531	0.026830267

Accession	Gene_Symbol	Gene_ID	Ensembl_Gene ID	log2(ratio(CM12H/CM0H))	log2(ratio(CM12R/CM0H))	log2(ratio(CM12H/CM12R))
A0A3Q1M127	KIDINS220	A0A3Q1M127	ENSBTAG00000007963	0.214018729	0.066135102	-0.147883627
A0AAA9T6L5	AGO1	A0AAA9T6L5	ENSBTAG00000012253	0.143662317	0.104731324	-0.038930993
Q3ZBA3	PLEKHA2	bta:530879; Q3ZBA3	ENSBTAG00000003509	-0.100324534	-0.043589269	0.056735266
F1N7Q7	COL4A2	F1N7Q7	ENSBTAG00000025210	-0.194470119	-0.304479124	-0.110009005
A0AAA9SCI1	PDXK	A0AAA9SCI1	ENSBTAG00000018186	-0.042435266	0.072149786	0.114585052
Q9N2J2	GPX4	bta:286809; Q9N2J2	ENSBTAG00000053003	-0.106041637	-0.132259158	-0.026217521
E1BER5	EPHA4	E1BER5	ENSBTAG00000010030	0.292505984	0.076025838	-0.216480146
B0JYR3	WASL	B0JYR3		0.151324018	0.178574053	0.027250034
A0AAF6Z7G4	RNASEH2B	A0AAF6Z7G4	ENSBTAG00000020149	-0.134149429	-0.255096586	-0.120947158
A7MAZ3	UBA5	A7MAZ3; bta:509292		-0.110583166	-0.116644919	-0.006061753
A0A3Q1LSY0	PAK3	A0A3Q1LSY0; bta:534526	ENSBTAG00000015670	-0.025535092	0.118566134	0.144101226
A0AAA9RUU3	CD47	A0AAA9RUU3	ENSBTAG00000003585	-1.572290853	-1.526278456	0.046012398
A7E3V0	SQSTM1	A7E3V0		0.044253238	0.148589898	0.10433666
A0AAA9SXN7	TLK2	A0AAA9SXN7	ENSBTAG00000015713	0.164182761	0.026284645	-0.137898116
A0AAA9T1A8	LUM	A0AAA9T1A8	ENSBTAG00000001745	0.304044003	-0.596828788	-0.900872791
Q3SX39	MNAT1	bta:534176; F1MPA0; Q3SX39	ENSBTAG00000030801	0.044313901	-0.092932939	-0.137246839
A3KN01	ZNF830	A3KN01; bta:539497	ENSBTAG00000020340	0.248679113	0.095191001	-0.153488112
G3X7E1	SAP18	bta:615692; G3X7E1	ENSBTAG00000018631	0.016000479	-0.014824647	-0.030825126
E1BPL3	ABCB7	E1BPL3	ENSBTAG00000011028	0.120671771	-0.012641439	-0.13331321
F1MYF5	HS2ST1	F1MYF5	ENSBTAG00000017595	0.131117552	0.10555865	-0.025558902
A0AAA9TWT4	DYNC2H1	A0AAA9TWT4	ENSBTAG00000019309	-0.040197089	-0.066642341	-0.026445252
G3N0U8	ERLIN1	bta:617074; G3N0U8	ENSBTAG00000007588	-0.100435951	-0.109922942	-0.009486991
A0A3Q1MEL1	RHOT1	A0A3Q1MEL1	ENSBTAG00000010001	-0.022745663	0.028727178	0.051472841
Q0VD11	KIAA1715	bta:533298; Q0VD11		0.216875416	0.042056148	-0.174819268
A0AAF6DM91	TRIM3	A0AAF6DM91	ENSBTAG00000003565	0.077041036	0.037552746	-0.03948829
Q5J801				-0.309918077	0.177109669	0.487027747
A2VDQ5	NLN	A2VDQ5; bta:538650		-0.478047297	-0.282399731	0.195647566
A0A3Q1MUB3	ARHGAP31	A0A3Q1MUB3; bta:515490	ENSBTAG00000000210	-0.050915839	-0.060754031	-0.009838192
Q2KIN5	HMBS	bta:515614; Q2KIN5	ENSBTAG00000005364	0.086628962	0.091957481	0.00532852
A0A3Q1LXT2	CFAP36	A0A3Q1LXT2	ENSBTAG00000000542	-0.365478373	-0.178370692	0.187107682
A0AAA9TAG9	TXLNG	A0AAA9TAG9; bta:515927	ENSBTAG00000001768	0.046018565	-0.092284457	-0.138303022
A0AAF6YUX3	GGCT	A0AAF6YUX3	ENSBTAG00000010597	0.089925679	0.065821123	-0.024104557
A0A3Q1N3F3	PPIH	A0A3Q1N3F3	ENSBTAG00000004590	-0.160912575	-0.068867498	0.092045077
F1MCK7	WDR43	bta:538915; F1MCK7	ENSBTAG00000018196	-0.491889078	-0.222392421	0.269496656
A7MB77	RBM17	A7MB77; bta:512694	ENSBTAG00000021951	-0.01093992	-0.08703718	-0.07609726
F6QCN2	PSME3IP1	F6QCN2	ENSBTAG00000002242	0.080895594	-0.009425013	-0.090320607
P82925	MRPS31	bta:534185; P82925; Q148G3	ENSBTAG00000015522	-0.138249938	-0.195490631	-0.057240693
Q5E9K3	PNPO	bta:512573; Q5E9K3	ENSBTAG00000012259	-0.169643253	-0.107608974	0.062034278
F6RBM0	HSD17B12	F6RBM0	ENSBTAG00000000087	-0.110558375	0.002953317	0.113511693
Q2YDN6	RPF2	bta:511294; Q2YDN6		-0.275844213	-0.26622621	0.009618003
A0AAA9SRY0	ATAD1	A0AAA9SRY0	ENSBTAG00000000806	0.00557564	-0.054071823	-0.059647463
E1BMX2	TRMT2A	E1BMX2	ENSBTAG00000019871	-0.179447776	-0.192243381	-0.012795605
F1MG80	NMI	F1MG80	ENSBTAG00000032369	-0.250283704	-0.077374844	0.17290886
A6QLS4	NDE1	A6QLS4; bta:508088	ENSBTAG00000015986	-0.068654348	-0.318938052	-0.250283704
A0AAF6DLS1	ARHGAP18	A0AAF6DLS1	ENSBTAG00000015381	-0.023678996	0.049468676	0.073147673
A0AAA9U0G2	ARPC4	A0AAA9U0G2	ENSBTAG00000007964	-0.310642287	0.060645009	0.371287296
A0AAA9TBR3	PAOX	A0AAA9TBR3	ENSBTAG00000018321	-0.054383637	-0.141846478	-0.087462841
F1MWB8	DMXL2	F1MWB8	ENSBTAG00000000737	-0.156401557	-0.109007031	0.047394526
A7Z062	ZC3H7A	A0A8J8XWW7; A7Z062; bta:540922; M5FKF8	ENSBTAG00000014826	-0.425382668	-0.231384006	0.193998662
A5PJZ4	HECTD3	A5PJZ4; bta:513646		-0.04143409	0.260844633	0.302278723
A0A3Q1M861	EHBP1	A0A3Q1M861	ENSBTAG00000044173	-0.026808797	-0.051225323	-0.024416527
Q3T0V7	EDF1	bta:515380; Q3T0V7	ENSBTAG00000030302	0.409587824	0.068634903	-0.340952921
A4FUE7	ZC2HC1A	A4FUE7; bta:516900		0.087462841	0.11401626	0.026553419
F6QEG6	RABGAP1L	F6QEG6	ENSBTAG00000049490	-0.109815094	-0.172099373	-0.062284278
Q2KIM0	FUCA1	bta:509522; Q2KIM0	ENSBTAG00000030434	0.027515679	0.206697787	0.179182108
Q0V8A9	TIMM50	Q0V8A9		-0.097749791	-0.202607105	-0.104857314
G5E628	UBQLN2	G5E628	ENSBTAG00000021843	0.129767875	0.250248251	0.120480376
O97681	ATP6V0A2	bta:338038; O97681	ENSBTAG00000016523	0.131829954	0.124127346	-0.007702608
E1BEM1	EEF2K	bta:521730; E1BEM1	ENSBTAG00000017662	0.134749412	0.068947354	-0.065802058
A0AAA9T257	ALG11	A0AAA9T257; bta:540845	ENSBTAG00000010337	0.0393425	-0.058950995	-0.098293495
P55052	FABP5	bta:281760; O62808; P55052; Q5E9D9	ENSBTAG00000047330	0.195902953	0.329950918	0.134047965
A0AAF6YH77	YKT6	A0AAF6YH77	ENSBTAG00000000274	0.022281447	-0.055797838	-0.078079286
A0AAA9SNV6	ZNF281	A0AAA9SNV6	ENSBTAG00000047428	-0.0094692	-0.260878716	-0.251409516
A0AAA9S2F0	TXN	A0AAA9S2F0	ENSBTAG00000002953	0.074406688	0.181432102	0.107025413
F1N1L4	DPP8	bta:536604; F1N1L4	ENSBTAG00000014807	-0.237404992	-0.278737014	-0.041332022
A0A3Q1N960	SYTL4	A0A3Q1N960; bta:515978	ENSBTAG00000005724	-0.069353281	-0.080999722	-0.011646441
A0AAA9RS96	UBXN7	A0AAA9RS96	ENSBTAG00000007577	0.157491378	0.008656196	-0.148835182
F1MXY8	SLC25A1	F1MXY8	ENSBTAG00000008528	-0.025995209	0.064550316	0.090545524
F6K7I9	CD46	F6K7I9		0.202842205	0.088371168	-0.114471037
A0AAA9SD62	STAM	A0AAA9SD62	ENSBTAG00000002658	0.182864057	0.179874015	-0.002990043
A4FV68	PPP2R5E	A4FV68; bta:533617		0.147677168	0.059239203	-0.088437965
A0AAA9TSI6	PACSIN3	A0AAA9TSI6	ENSBTAG00000009576	-0.067337991	-0.014167332	0.053170659
A0AAA9S0T2	SERPINF2	A0AAA9S0T2	ENSBTAG00000020859	-0.515380049	0.266089719	0.781469769
A0A3Q1N325	PPP3CB	A0A3Q1N325	ENSBTAG00000019525	-0.169533018	-0.264289469	-0.094756451
A5PK18	AGK	A5PK18; bta:514693		-0.21528384	-0.174241608	0.041042233
A0A3Q1MRT1	PRKAG1	A0A3Q1MRT1	ENSBTAG00000014426	-0.484033592	-0.095062197	0.388971395
A0AAA9SLG2	CLTA	A0AAA9SLG2	ENSBTAG00000001137	0.004295869	0.043785589	0.03948972
Q9XT56	F11R	bta:281258; Q5E9V8; Q9XT56	ENSBTAG00000017846	0.185159515	0.133739424	-0.051420091
F1MM52	NTPCR	F1MM52	ENSBTAG00000013935	0.102218649	0.075566257	-0.026652392
A0AAA9T259	PLEK2	A0AAA9T259	ENSBTAG00000016255	-0.181864615	-0.335644974	-0.153780359
A0A3Q1MIS5	NBEA	A0A3Q1MIS5	ENSBTAG00000022991	-0.194349384	0.014827418	0.209176802
A0AAA9S2L1	XRCC1	A0AAA9S2L1; bta:616905	ENSBTAG00000016268	-0.285402219	-0.09557766	0.189824559
A0A3Q1MPI8	ZNF800	A0A3Q1MPI8	ENSBTAG00000020004	0.112474729	0.030012569	-0.08246216
A0A3Q1MWX6	RPRD1B	A0A3Q1MWX6	ENSBTAG00000005086	0.233718839	0.134485332	-0.099233507
Q3T112	PSMB8	bta:282013; Q32S32; Q3T112		-0.168631684	0.044590151	0.213221835
Q3SZ19	PSMD9	bta:513315; Q3SZ19	ENSBTAG00000004179	0.06667104	-0.004183749	-0.070854788
Q1W668	SMAD2	bta:516010; Q1W668		-0.091081723	-0.026105891	0.064975832
A0A3Q1M6S1	RELCH	A0A3Q1M6S1; bta:504761	ENSBTAG00000016666	-0.039933627	-0.020568302	0.019365325
F1MKD1	NTMT1	F1MKD1	ENSBTAG00000015437	0.168162393	0.12882998	-0.039332412

Accession	Gene_Symbol	Gene_ID	Ensembl_Gene ID	log2(ratio(CM12H/CM0H))	log2(ratio(CM12R/CM0H))	log2(ratio(CM12H/CM12R))
Q2KI45	TRMT10C	bta:532418; Q2KI45	ENSBTAG00000024027	-0.101610002	0.021942553	0.123552555
A0AAF6YUI9	CPSF2	A0AAF6YUI9	ENSBTAG00000010227	-0.074382196	-0.299746184	-0.225363988
Q862I1	RPL24	bta:282048; P38663; Q3T157; Q862I1; Q862I5; Q862V7	ENSBTAG00000013461	0.010216396	0.037587368	0.027370972
Q9TU47	EIF6	bta:286811; Q2TBM1; Q9TU47	ENSBTAG00000011263	-0.080389387	0.045276573	0.12566596
Q3ZBT6	TRA2B	bta:615156; Q3ZBT6	ENSBTAG00000001697	0.075387122	0.237747008	0.162359886
A0A3Q1MK32	UBE2L3	A0A3Q1MK32	ENSBTAG00000013038	0.052631502	-0.043532895	-0.096164397
F1MQQ0	CDC45	F1MQQ0	ENSBTAG00000004286	-1.423024187	-1.316582489	0.106441698
Q0III9	ACTN3	bta:539375; Q0III9	ENSBTAG00000022244	-0.073425942	0.097349956	0.170775898
A0A3Q1MG73	FBLN1	A0A3Q1MG73	ENSBTAG00000012088	-0.216811389	-0.013483229	0.20332816
A0AAA9TJZ6	NUP35	A0AAA9TJZ6	ENSBTAG00000009777	-0.299381009	-0.215803876	0.083577133
A0AAF6ZHP5	C4BPA	A0AAF6ZHP5	ENSBTAG00000009876	-0.446203073	-0.438978747	0.007224327
Q0VBZ5	JUNB	bta:514246; Q0VBZ5	ENSBTAG00000012046	-0.292180751	-0.249359469	0.042821283
A0AAF6Z7M5	CSNK1D	A0AAF6Z7M5	ENSBTAG00000019986	-0.063621201	-0.28536332	-0.221742119
A0AAF6Z8P4	SNAP29	A0AAF6Z8P4	ENSBTAG00000020760	0.274556276	0.080348994	-0.194207282
F1MD13				-0.235311838	-0.232235728	0.00307611
F6QIZ6	TUBGCP4	F6QIZ6	ENSBTAG00000014479	-0.040077439	-0.059168201	-0.019090761
A0AAA9S8Y0	TPD52L2	A0AAA9S8Y0	ENSBTAG00000021232	0.038792483	-0.018262206	-0.057054689
A0AAA9T383	CWF19L2	A0AAA9T383	ENSBTAG00000018629	0.170231469	0.006879818	-0.163351651
A0AAA9SUC5	KYAT3	A0AAA9SUC5	ENSBTAG00000000505	-0.213173891	-0.147313305	0.065860587
A7Z085	WDR82	A7Z085; bta:783336	ENSBTAG00000002960	-0.014158063	0.044394119	0.058552182
A0AAA9T2E9	PHF3	A0AAA9T2E9	ENSBTAG00000019139	-0.13383817	-0.197716067	-0.063877897
A0A3Q1LRL0	NACC1	A0A3Q1LRL0	ENSBTAG00000016350	-0.055417114	-0.107323264	-0.051906149
F1MYE0	IRS1	bta:538598; F1MYE0	ENSBTAG00000021308	-0.023248946	0.00256024	0.025809186
A0AAA9RVY9	DBT	A0AAA9RVY9	ENSBTAG00000006320	0.195550809	0.264326564	0.068775755
F1ML90	PARP14	F1ML90	ENSBTAG00000016656	-0.183339645	0.002511219	0.185850864
A0AAF6Z8C2	ACP1	A0AAF6Z8C2	ENSBTAG00000020498	0.101311572	0.250205407	0.148893836
Q32PJ6	CIAO1	bta:540069; Q32PJ6	ENSBTAG00000015659	-0.06832219	-0.148892399	-0.080570209
F1MM82	SLC39A10	A0A3Q1N2V0; F1MM82	ENSBTAG00000016782	0.126671803	0.04051516	-0.086156644
Q0P592	FN3KRP	bta:615868; F1MII4; Q0P592	ENSBTAG00000015390	0.071178764	0.180572246	0.109393481
Q3SZA5	SMS	A1L596; bta:615950; Q3SZA5	ENSBTAG00000018382	0.012504477	0.001569	-0.010935478
A0A3Q1MEN6	CHTOP	A0A3Q1MEN6	ENSBTAG00000005166	0.209650082	0.044438234	-0.165211848
G3MX46	CIC	bta:538483; G3MX46	ENSBTAG00000019785	0.110162635	-0.056252546	-0.166415181
F1MPG0	MLH1	bta:533652; F1MPG0	ENSBTAG00000016758	-0.355686288	-0.094550701	0.261135587
F1N099	BLTP3B	F1N099	ENSBTAG00000006040	-0.110349472	-0.268816758	-0.158467286
Q3ZBY2	TAGLN3	bta:515562; Q3ZBY2		-0.045179983	-0.133640884	-0.088460901
E1BK69	UFSP2	bta:617788; E1BK69	ENSBTAG00000018454	-0.059054426	-0.087462841	-0.028408415
A0AAA9RZX6	SMARCD2	A0AAA9RZX6	ENSBTAG00000021062	0.032355373	-0.028539406	-0.06089478
F1N3H0	DCAF8	bta:510316; F1N3H0	ENSBTAG00000005796	-0.213554068	-0.15027428	0.063279788
E1BML6	SLC2A13	bta:613556; E1BML6	ENSBTAG00000043962	0.057898384	0.168542447	0.110644064
A0AAA9S547	CD58	A0AAA9S547	ENSBTAG00000040131	0.070519189	0.175670991	0.105151801
A0A3Q1N5D7	GOLM2	A0A3Q1N5D7	ENSBTAG00000012545	0.126953604	0.052889693	-0.074063912
F1MM35	VPS13A	F1MM35	ENSBTAG00000017734	-0.206251114	-0.148976308	0.057274805
Q2KI74	TRADD	bta:504707; Q2KI74	ENSBTAG00000012642	0.017124178	-0.03487046	-0.051994638
Q5BIN2	SMARCB1	A2VE50; bta:537412; Q5BIN2	ENSBTAG00000009988	0.123735368	0.096661778	-0.02707359
A0A3Q1M2B6	EGLN1	A0A3Q1M2B6; bta:534075	ENSBTAG00000053296	0.32220685	0.228948783	-0.093258067
A0A3Q1MIB8	CASP2	A0A3Q1MIB8; bta:531419	ENSBTAG00000018159	-0.114702497	-0.070787917	0.04391458
Q08DC2	RAB31	bta:616979; Q08DC2	ENSBTAG00000046533	-0.031138512	-0.054052904	-0.022914392
Q17QI5	VAMP7	bta:613984; Q17QI5		-0.050874048	0.096663162	0.147537211
A7YY77	ENDOD1	A7YY77; bta:538618; F1MN88	ENSBTAG00000024803	0.034240324	0.051060489	0.016820165
Q1JQA2	SMAD1	bta:540488; Q06AL6; Q1JQA2	ENSBTAG00000002835	0.333797651	0.071642669	-0.262154982
Q3SZV7	HPX	bta:534509; Q3SZV7		-0.224123309	0.205560966	0.429684275
Q3T0Q3	POLR2C	A7E3Q9; bta:504452; Q3T0Q3	ENSBTAG00000001856	-0.15104735	0.144526483	0.295573833
A0AAA9RS89	RRP8	A0AAA9RS89	ENSBTAG00000000697	-0.017198769	-0.059058089	-0.04185932
A0AAA9T454	CHD2	A0AAA9T454	ENSBTAG00000017776	-0.073715154	0.013495842	0.087210996
E1BGX4	WBP2	bta:507060; E1BGX4	ENSBTAG00000008634	0.197483635	0.352301744	0.154818109
A0A3Q1N178	TPP1	A0A3Q1N178	ENSBTAG00000015403	0.04535675	0.187471983	0.142115233
F1N532	EMG1	F1N532	ENSBTAG00000013126	-0.22881869	-0.366181333	-0.137362642
A0A3Q1MSA9	MTCL1	A0A3Q1MSA9	ENSBTAG00000009459	0.023369559	-0.052210799	-0.075580358
A0A3Q1LMP2	KRAS	A0A3Q1LMP2; bta:541140	ENSBTAG00000009778	0.141043881	0.032768029	-0.108275852
A0AAA9SVU4	SIRT5	A0AAA9SVU4	ENSBTAG00000014904	0.019206669	0.039608757	0.020402088
A0AAF6ZPG9	GUK1	A0AAF6ZPG9	ENSBTAG00000014775	0.011614629	-0.052590827	-0.064205456
A0A452DIR7	BPNT2	A0A452DIR7	ENSBTAG00000015637	-0.039031812	-0.172161605	-0.133129793
A0AAA9TSN5	NHLRC2	A0AAA9TSN5	ENSBTAG00000003690	-0.017540581	-0.030837404	-0.013296823
Q0VD30	CCZ1	bta:511088; Q0VD30	ENSBTAG00000003288	-0.172918146	-0.005992511	0.166925634
F1MPL1	MFN1	bta:515180; F1MPL1	ENSBTAG00000010485	-0.140230614	-0.24549696	-0.105266347
Q0P5N1	CNPY3	bta:510220; Q0P5N1	ENSBTAG00000015900	0.280375605	0.17009355	-0.110282055
A0AAA9S8X1	RCOR1	A0AAA9S8X1	ENSBTAG00000002336	0.079108958	-0.124384218	-0.203493176
A0A3Q1MAB7	DCUN1D1	A0A3Q1MAB7	ENSBTAG00000012797	0.071701465	0.133041289	0.061339824
A0AAA9TL37	NUF2	A0AAA9TL37	ENSBTAG00000007247	0.064305376	0.016015281	-0.048290095
F1MSS2	DDHD2	bta:513116; F1MSS2	ENSBTAG00000013145	0.192454434	0.143590854	-0.048863581
A0AAA9SQY1	MIPEP	A0AAA9SQY1	ENSBTAG00000008888	-0.062284278	0.005793964	0.068078242
F1MM20	CCDC86	F1MM20	ENSBTAG00000014215	0.090447367	-0.100909015	-0.191356382
A6QLC7	TRAPPC11	A6QLC7; bta:537211		-0.066696447	-0.031681006	0.035015441
P79132	CAV1	A4D7R5; bta:281040; P79132; Q5PX19		0.196397213	0.186272996	-0.010124217
F1MXV4	SIPA1L1	bta:787248; F1MXV4	ENSBTAG00000015805	-0.251979217	-0.004631449	0.247347769
Q3ZCK2	RALA	bta:538477; Q3ZCK2	ENSBTAG00000006661	0.020483473	0.040680187	0.020196714
F1N388	AKAP11	F1N388	ENSBTAG00000014752	0.264837292	-0.158374139	-0.423211431
A0AAA9SB14	NUP58	A0AAA9SB14	ENSBTAG00000015225	0.204863536	0.078159063	-0.126704473
A0A3Q1N213	NLE1	A0A3Q1N213	ENSBTAG00000019094	-0.105277037	-0.202887833	-0.097610797
A0AAF6YR37	MAPRE2	A0AAF6YR37	ENSBTAG00000007520	0.113747574	0.034051849	-0.079695726
A5D7A3	MKLN1	A5D7A3; bta:508844; F1MMV9		-0.002537723	-0.11611123	-0.113573507
E1BAI0	RAB23	bta:618588; E1BAI0	ENSBTAG00000005362	-0.194417794	-0.299030405	-0.104612611
A0AAF6ZBG6				0.156250065	0.274894561	0.118644496
A0A3Q1MJQ1				-0.472068444	-0.09190551	0.380162934
F1MJI8	PHKA2	F1MJI8	ENSBTAG00000015355	-0.028804029	0.016678741	0.04548277
A0AAA9T1Y2	IGSF23	A0AAA9T1Y2	ENSBTAG00000048075	0.063867422	0.151398317	0.087530895
F1MEP0	MRPL45	F1MEP0	ENSBTAG00000025001	-0.116253068	-0.148314278	-0.032061209
Q2KIE4	MCTS1	bta:508412; Q2KIE4		-0.095383156	-0.115240574	-0.019857418
G1K130	GPATCH1	G1K130	ENSBTAG00000000721	0.160633399	0.050404953	-0.110228445

Accession	Gene_Symbol	Gene_ID	Ensembl_Gene ID	log2(ratio(CM12H/CM0H))	log2(ratio(CM12R/CM0H))	log2(ratio(CM12H/CM12R))
F6R3N4	KRT10	F6R3N4	ENSBTAG00000020824	-0.549522831	-0.566201573	-0.016678741
B0JYL0	SMAD4	B0JYL0	ENSBTAG00000006919	0.135636302	-0.020673568	-0.156309871
A2VE85	PRRX1	A2VE85; bta:540901	ENSBTAG00000004570	0.072931522	-0.065588342	-0.138519864
A0AAA9S9F0	BROX	A0AAA9S9F0	ENSBTAG00000007595	0.072544213	0.086320716	0.013776503
A0AAF6YMX3	CDK9	A0AAF6YMX3	ENSBTAG00000004695	-0.2504386	-0.37899828	-0.12855968
A0AAA9SH73	SNX4	A0AAA9SH73	ENSBTAG00000011383	0.188947438	0.119637568	-0.06930987
Q3ZBG5	BAG2	bta:506107; Q3ZBG5		0.390214486	0.155901584	-0.234312902
A0AAA9S9E2	PHACTR4	A0AAA9S9E2	ENSBTAG00000002727	0.182826017	0.212084959	0.029258942
F6QYT8	AP1G2	F6QYT8	ENSBTAG00000007099	-0.210158714	-0.13720268	0.072956033
Q08D87	FARS2	bta:505390; Q08D87	ENSBTAG00000018072	-0.150714397	-0.331954712	-0.181240315
F1MNQ8	STX2	F1MNQ8	ENSBTAG00000007988	0.084428781	0.092002001	0.00757322
P21839	BCKDHB	bta:282150; P21839; Q148F4; Q28047	ENSBTAG00000012096	-0.132787729	-0.156659133	-0.023871404
A0AAA9RYD0	QRSL1	A0AAA9RYD0	ENSBTAG00000017557	-0.11302791	-0.027111846	0.085916064
Q5EA68	SEC61A1	A9JSM4; bta:505064; Q5EA68	ENSBTAG00000004937	0.206648358	0.073630232	-0.133018126
F6RN30	GGA1	F6RN30	ENSBTAG00000006464	-0.055200167	-0.078838518	-0.02363835
A0AAA9U2M1	ATE1	A0AAA9U2M1	ENSBTAG00000003178	0.002971567	0.092179533	0.089207967
A0A3Q1MC43	ARID4B	A0A3Q1MC43; bta:534612	ENSBTAG00000000222	0.202579168	-0.030774753	-0.233353921
F1N4M3	PDPR	F1N4M3	ENSBTAG00000016471	-0.076298206	-0.056002044	0.020296162
E1BEY9	LSM14B	E1BEY9	ENSBTAG00000021558	0.301407654	0.22192287	-0.079484784
A0AAA9S307	STIM2	A0AAA9S307	ENSBTAG00000019353	0.021716843	-0.10120377	-0.122920614
H7BWW0	CORO1B	H7BWW0	ENSBTAG00000004783	-0.058370563	0.091888038	0.150258601
A0AAA9T947	ATP11A	A0AAA9T947	ENSBTAG00000000446	-0.021082134	0.016646666	0.0377288
A0AAA9TM73	ALG9	A0AAA9TM73	ENSBTAG00000020279	0.102403855	0.006139137	-0.096264718
F6RD06	FKBP7	F6RD06	ENSBTAG00000001097	0.141624858	-0.028014376	-0.169639234
E1BA21	TFAM	E1BA21	ENSBTAG00000003546	0.161074084	0.199041934	0.03796785
E1BC89	OSBPL5	E1BC89	ENSBTAG00000001077	-0.313598215	-0.189120999	0.124477216
Q3SYZ2	MAPKAPK3	bta:615215; Q0V8M1; Q3SYZ2	ENSBTAG00000016532	-0.301853655	-0.169925001	0.131928653
F1MWW9	CDCA5	F1MWW9	ENSBTAG00000026971	0.844829627	0.324456921	-0.520372706
A6QLY8	IGFBP7	A6QLY8; bta:616368		-0.2410081	-0.808499464	-0.567491364
A0AAF6YT43	MX1	A0AAF6YT43	ENSBTAG00000030913	-0.204849218	0.04276232	0.247611537
A0AAA9SEM5	MRPL40	A0AAA9SEM5	ENSBTAG00000019891	-0.09169569	0.010569242	0.102264932
Q3SZF8	SNRPD2	bta:514932; Q3SZF8	ENSBTAG00000012177	-0.056918429	0.020778492	0.077696921
A0A3Q1MJR9	VPS52	A0A3Q1MJR9	ENSBTAG00000002643	0.010171805	-0.31006794	-0.320239745
Q09430	PFN2	A4FUW7; bta:539034; Q09430		0.251225104	0.093599367	-0.157625736
E1BPP2	BMS1	E1BPP2	ENSBTAG00000012878	-0.22674564	-0.286217447	-0.059471808
Q2KID0	EXOSC2	bta:615712; Q2KID0	ENSBTAG00000048071	-0.037067327	0.064680996	0.101748323
F1MBS9	CDC40	F1MBS9	ENSBTAG00000000409	-0.104588901	-0.006319305	0.098269596
Q861S4	RPL21	bta:326584; Q861S4	ENSBTAG00000001648	-0.12195188	-0.093635072	0.028316808
A0AAA9T6Q2	UBE2I	A0AAA9T6Q2	ENSBTAG00000038866	0.003066303	-0.015430041	-0.018496344
A0AAA9SQL2	TBC1D10B	A0AAA9SQL2	ENSBTAG00000031852	0.177633693	0.149278456	-0.028355237
A0AAA9TZ45	PLAT	A0AAA9TZ45	ENSBTAG00000001244	-0.221926058	-0.283610458	-0.0616844
E1B8G9	H2BC26	bta:520120; E1B8G9	ENSBTAG00000058723	-0.282933963	-0.116575577	0.166358386
F2Z4I1	RHEB	F2Z4I1	ENSBTAG00000031861	-0.194816177	-0.228387327	-0.03357115
A0AAF6Z191	GLE1	A0AAF6Z191	ENSBTAG00000030566	-0.231920227	-0.384220752	-0.152300526
E1BH45	RB1CC1	bta:539858; E1BH45	ENSBTAG00000000878	0.01647101	-0.045900851	-0.06237186
Q7SIB2	COL4A1	G1K238; Q7SIB2	ENSBTAG00000012849	-0.172467196	-0.249429178	-0.076961982
F1N1E3	MMP14	F1N1E3	ENSBTAG00000014824	-0.224680598	-0.200833856	0.023846742
E1BK11	ANAPC5	bta:540537; E1BK11	ENSBTAG00000003092	-0.242743002	-0.12367437	0.119068631
A0AAA9TT40	ZC3H13	A0AAA9TT40	ENSBTAG00000007067	0.169281085	0.030102032	-0.139179053
A3KMX8	ARMT1	A3KMX8; bta:540698		0.313435865	0.296299953	-0.017135912
F1N0R7	FLRT3	bta:529416; F1N0R7	ENSBTAG00000003319	0.098768758	-0.030999116	-0.129767875
A0A3Q1MR39	FLOT2	A0A3Q1MR39	ENSBTAG00000010073	0.003079393	0.073615195	0.070535802
A0AAA9TWP9	NT5C3A	A0AAA9TWP9	ENSBTAG00000016709	0.036566129	0.139551352	0.102985223
E1BAL6	EIF2AK4	bta:513829; E1BAL6	ENSBTAG00000014351	0.031457432	0.051124465	0.019667033
G3MWN1	ARG2	G3MWN1	ENSBTAG00000001945	0.084282596	0.209661863	0.125379267
A0AAA9TZ73	DBI	A0AAA9TZ73	ENSBTAG00000009517	-0.253620375	-0.51524796	-0.261627585
A0AAA9SJL3	SPG7	A0AAA9SJL3	ENSBTAG00000012041	0.041903953	-0.011777168	-0.053681121
A0AAA9S2Z7	FABP3	A0AAA9S2Z7	ENSBTAG00000016819	0.180110235	0.180110235	0
E1BGD1	TOMM34	E1BGD1	ENSBTAG00000003256	0.054232312	-0.007290036	-0.061522348
F6RU89	RPA2	F6RU89	ENSBTAG00000006225	0.138345809	0.233383599	0.09503779
F1N795	RASA2	F1N795	ENSBTAG00000015413	0.028727178	0.084520405	0.055793227
F1MY63	TYMS	F1MY63	ENSBTAG00000007003	-0.577175463	-0.392392503	0.18478296
A0A3Q1M488	MRPL39	A0A3Q1M488	ENSBTAG00000019542	-0.246982001	-0.235058833	0.011923167
Q3B7M6	NEDD1	bta:519463; Q3B7M6	ENSBTAG00000001894	0.197574677	0.026615856	-0.170958821
A5D7E0	RAB35	A5D7E0; bta:614521; F1MSJ9		0.003223901	0.119196464	0.115972563
G3X687	TUBGCP6	bta:618908; G3X687	ENSBTAG00000000650	-0.075232426	-0.033130593	0.042101833
F6QYF9	DDX41	F6QYF9	ENSBTAG00000015187	-0.001429119	-0.047924826	-0.046495707
Q2YDM1	ARL1	bta:517345; Q2YDM1	ENSBTAG00000011883	-0.441896959	-0.273909762	0.167987197
A0AAA9TQT5	WDR47	A0AAA9TQT5	ENSBTAG00000020384	-0.025090981	0.034140141	0.059231122
F1N3R6	EPHB2	F1N3R6	ENSBTAG00000045902	0.02743323	0.042179815	0.014746585
A0A3Q1LM49	PPM1A	A0A3Q1LM49	ENSBTAG00000018127	-0.075442556	-0.042413755	0.0330288
A5D7C1	DDX52	A5D7C1; bta:510826	ENSBTAG00000010313	0.085442936	0	-0.085442936
A0A3Q1MXM3	ARID1B	A0A3Q1MXM3	ENSBTAG00000002728	-0.158761899	-0.202530652	-0.043768753
A0A3Q1M1E6	DHRS7B	A0A3Q1M1E6	ENSBTAG00000010559	0.031677212	0.05708737	0.025410159
A0AAA9SJQ0	RPS6KA4	A0AAA9SJQ0; bta:523746	ENSBTAG00000039153	-0.178460813	-0.314901969	-0.136441156
A0A3Q1M3Q2	PARN	A0A3Q1M3Q2	ENSBTAG00000018097	-0.192082587	-0.186096292	0.005986295
A0A173FDG1	BoLA	A0A173FDG1		0.072408496	0.138605238	0.066196743
A0A3Q1MEJ9	MBNL1	A0A3Q1MEJ9	ENSBTAG00000004564	-0.048959903	0.148205372	0.197165275
Q3SZ32	TCEB2	A0A8J8XQC3; bta:617277; F1N668; M5FK31; Q3SZ32		-0.030743523	-0.02778718	0.002956343
E1BDC9	VPS52	E1BDC9	ENSBTAG00000002643	-0.056955885	0.012357207	0.069313091
F1MM23	CDK5RAP3	F1MM23	ENSBTAG00000014202	0.00292636	0.030434675	0.027508315
A5PK88	PITHD1	A5PK88; bta:506494	ENSBTAG00000021620	0.186224875	0.238497297	0.052272422
F6Q3P6	MINK1	F6Q3P6	ENSBTAG00000004907	0.03446341	0.164630702	0.130167292
F1MXJ5	IST1	F1MXJ5	ENSBTAG00000019883	0.218409985	0.143297786	-0.0751122
E1BJ09	MIB1	bta:533735; E1BJ09	ENSBTAG00000007467	0.102803387	-0.122856748	-0.225660135
Q1LZE1	KCMF1	bta:613522; Q1LZE1		0.080528437	0.053650054	-0.026878384
E1BEN4	GMPPA	bta:504889; E1BEN4	ENSBTAG00000002995	-0.171456688	-0.286244445	-0.114787757
Q1RMT1	ERCC3	bta:507984; Q1RMT1	ENSBTAG00000020777	-0.05402946	0.037579298	0.091608758
Q3SZ29	POLR1E	bta:511587; F1MXX3; Q3SZ29	ENSBTAG00000012243	0.04535675	0.069783917	0.024427166

Accession	Gene_Symbol	Gene_ID	Ensembl_Gene ID	log2(ratio(CM12H/CM0H))	log2(ratio(CM12R/CM0H))	log2(ratio(CM12H/CM12R))
A0A3Q1NE17	DLGAP4	A0A3Q1NE17	ENSBTAG00000001741	-0.059647463	-0.178996391	-0.119348927
A0A3Q1MSC8	SRRM1	A0A3Q1MSC8	ENSBTAG00000013772	0.052911531	0.139512149	0.086600618
F6RWP3	PAIP1	F6RWP3	ENSBTAG00000020376	-0.038219322	0.058516465	0.096735787
A0AAA9SJI3	TBC1D13	A0AAA9SJI3	ENSBTAG00000012480	-0.026883294	-0.028391601	-0.001508307
Q3ZCC8	TPPP3	bta:614988; Q3ZCC8	ENSBTAG00000019822	-0.026152288	-0.008664861	0.017487427
A7MB47	CNOT9	A7MB47; bta:536537	ENSBTAG00000003807	0.027425744	0.002911595	-0.024514149
A0AAA9SZ09	ILKAP	A0AAA9SZ09	ENSBTAG00000010176	0.084762062	0.081968847	-0.002793215
Q32L30	TEX264	bta:535455; Q32L30	ENSBTAG00000034796	0.041138695	-0.121201711	-0.162340406
A8E4N5	RING1	A8E4N5; bta:100125778; F1MUV0	ENSBTAG00000018884	0.162112704	0.079807401	-0.082305303
A0A3Q1M5N1	ZMYM2	A0A3Q1M5N1	ENSBTAG00000020403	-0.211054877	-0.123204579	0.087850298
A0AAA9SGY7	PSMG2	A0AAA9SGY7	ENSBTAG00000010552	0.035282977	0.062306151	0.027023174
Q32PA4	PHPT1	bta:618691; Q32PA4	ENSBTAG00000060065	0.041199117	-0.008382912	-0.049582029
A0AAF6YM21	NDUFA8	A0AAF6YM21	ENSBTAG00000004295	-0.007514054	-0.039250816	-0.031736762
A0AAA9TF84	PACS1	A0AAA9TF84	ENSBTAG00000009026	-0.05910352	-0.331084085	-0.271980565
A0AAA9TG44	NMD3	A0AAA9TG44	ENSBTAG00000016228	-0.204672791	-0.248650488	-0.043977697
E1BIV7	ZFC3H1	bta:781886; E1BIV7	ENSBTAG00000012267	0.22157757	-0.002853997	-0.224431567
E1BJH5	MAP4K3	E1BJH5	ENSBTAG00000016442	0.016083723	-0.006484034	-0.022567757
A0AAA9RTS3	BOP1	A0AAA9RTS3	ENSBTAG00000009811	-0.281670123	-0.123428339	0.158241784
A0AAA9T7W8	FMN1	A0AAA9T7W8	ENSBTAG00000025634	-0.235664778	-0.217117479	0.018547299
A0AAA9T9D3	CHAMP1	A0AAA9T9D3	ENSBTAG00000020861	-0.04026387	0.059952159	0.10021603
A0A3Q1LQK8	OSBPL6	A0A3Q1LQK8	ENSBTAG00000014405	-0.091483061	-0.186235938	-0.094752877
G1K122	RBP4	bta:281444; G1K122	ENSBTAG00000000442	-0.055455605	-0.364874705	-0.3094191
A5PJM4	AIFM2	A5PJM4; bta:534217	ENSBTAG00000001165	0.082035769	0.226770862	0.144735093
F1MK36	DDRGK1	F1MK36	ENSBTAG00000013753	0.099437628	-0.064665214	-0.164102841
F1MRE2	TUFT1	F1MRE2	ENSBTAG00000017566	-0.067516947	0.041675198	0.109192145
Q645M6	FADD	bta:493720; Q645M6	ENSBTAG00000018274	-0.38039511	-0.293695487	0.086699623
Q3MHN8	TRMT5	bta:528221; Q3MHN8	ENSBTAG00000011796	-0.20469042	-0.137376308	0.067314112
Q3SWY6	STK25	bta:373543; Q3SWY6; Q6V9V8	ENSBTAG00000009676	-0.399440644	-0.375509135	0.023931509
Q56JX8	RPS13	bta:535668; Q56JX8	ENSBTAG00000040308	-0.108831603	-0.111660417	-0.002828815
A0A3Q1MI24	TIPRL	A0A3Q1MI24	ENSBTAG00000005393	-0.048412205	0.12035194	0.168764146
F6RNG7	FAM20B	F6RNG7	ENSBTAG00000013999	0.110918935	0.19416131	0.083242375
Q2KI55	RBPMS	bta:614417; Q2KI55		-0.013309089	0.138827705	0.152136794
E1B9D9	TRAF2	E1B9D9	ENSBTAG00000010497	-0.533010421	-0.488850608	0.044159813
A0AAA9SEL1	MED23	A0AAA9SEL1	ENSBTAG00000012418	-0.043532895	-0.0451122	-0.001579305
Q0VFX8	CRIP2	bta:780821; Q0VFX8		0.184790095	0.108127044	-0.076663051
A0A452DJ34	RPL28	A0A452DJ34	ENSBTAG00000023343	-0.10219215	-0.142637675	-0.040445526
A0AAA9RXH4	KRR1	A0AAA9RXH4	ENSBTAG00000011591	-0.343558111	-0.150731436	0.192826675
A0AAA9S719	NUP43	A0AAA9S719	ENSBTAG00000010196	0.141693282	0.126771057	-0.014922226
F6QYN9	SRSF4	F6QYN9	ENSBTAG00000013309	0.283513217	0.2988748	0.015361583
A0AAA9SLP6	AAMP	A0AAA9SLP6	ENSBTAG00000003927	-0.240779028	-0.061784073	0.178994955
A0AAF6YK23	PTMA	A0AAF6YK23	ENSBTAG00000002549	-0.122943088	-0.086734555	0.036208533
A0AAA9SLC8	MTMR1	A0AAA9SLC8	ENSBTAG00000009657	-0.143419247	-0.341118915	-0.197699668
Q2TBN3	CETN2	bta:508601; Q2TBN3	ENSBTAG00000007844	0.374765983	-0.042774024	-0.417540006
A0AAF6Z1W8	RPL18A	A0AAF6Z1W8	ENSBTAG00000015831	-0.347457467	-0.221111733	0.126345734
F2Z4H3	POLR2H	bta:505599; F2Z4H3	ENSBTAG00000040199	-0.046776771	-0.058298641	-0.01152187
Q17R09	DHX38	bta:520046; Q17R09	ENSBTAG00000013265	-0.299250036	-0.154545025	0.144705011
E1B9A3	MB21D2	bta:519376; E1B9A3	ENSBTAG00000005430	0.149949435	0.159167965	0.00921853
Q0VC98	GABPA	bta:510204; Q0VC98	ENSBTAG00000019043	-0.075874867	-0.080209457	-0.00433459
A0AAA9S345	UBE2N	A0AAA9S345	ENSBTAG00000021767	0.098159511	0.263298902	0.165139392
A0AAA9T3D4	ERGIC1	A0AAA9T3D4	ENSBTAG00000015955	0.128007612	0.058298641	-0.069708972
A0A3Q1MGT8	SGPL1	A0A3Q1MGT8	ENSBTAG00000009984	0.080782655	-0.038270017	-0.119052672
A0A3Q1MY43	DCAF5	A0A3Q1MY43	ENSBTAG00000048519	0.199418307	0.09363971	-0.105778597
F1MBN7	RIOK2	bta:540772; F1MBN7	ENSBTAG00000011763	-0.180691432	-0.343541908	-0.162850476
A0A452DJ05	MARF1	A0A452DJ05	ENSBTAG00000020387	0.261927195	0.179302139	-0.082625057
Q32L22	POLR1C	bta:516337; Q32L22	ENSBTAG00000018969	-0.122067887	-0.09934781	0.022720077
A0AAF7ATF7	ASS1	A0AAF7ATF7	ENSBTAG00000020747	-0.202105407	-0.169925001	0.032180406
A0AAA9TLZ5	MACROH2A1	A0AAA9TLZ5	ENSBTAG00000016105	-0.465909829	-0.336218752	0.129691077
Q0VC02	AAAS	bta:506561; Q0VC02		-0.024070549	-0.031675706	-0.007605158
A0AAA9SBD1	SSBP1	A0AAA9SBD1	ENSBTAG00000010931	0.176816448	0.068490295	-0.108326153
Q3ZBT5	STX7	bta:507031; Q3ZBT5	ENSBTAG00000017139	0.043579863	-0.006334566	-0.049914429
A0AAA9SWW0	TSC1	A0AAA9SWW0	ENSBTAG00000005190; ENSBTAG00000066551	-0.06933665	-0.001505159	0.067831491
A0A3Q1MM07	GAB1	A0A3Q1MM07	ENSBTAG00000002813	0.046975616	0.063388706	0.01641309
A0A3Q1NLW9	VPS37B	A0A3Q1NLW9; bta:535566	ENSBTAG00000048809	0.137206092	-0.001636637	-0.138842728
A0AAA9SKW6	BRCA1	A0AAA9SKW6	ENSBTAG00000022520	0.130086052	0.043721377	-0.086364675
E1B8Q4	CHTF18	A0A8J8Y4N9; E1B8Q4; M5FKI2	ENSBTAG00000019743	0.002856823	-0.039131245	-0.041988068
A4FV37	CAVIN3	A4FV37; bta:510203		-0.001694299	-0.034269902	-0.032575604
E1BBC2	TMEM209	E1BBC2	ENSBTAG00000015588	0.050262903	0.207052502	0.156789599
F6QQY9	SRI	F6QQY9	ENSBTAG00000010390	-0.139748527	0.040096645	0.179845172
Q8HXY9	CFDP1	bta:281682; Q2KIY4; Q8HXY9; Q8I031; Q8I033		0.121900053	0.076592425	-0.045307628
A0A3Q1M2S8	TUT4	A0A3Q1M2S8	ENSBTAG00000002721	-0.284344912	-0.137503524	0.146841388
A6QQW4	GTSE1	A6QQW4; bta:507901		0.127032907	0.172836597	0.04580369
A6QLY4	ISOC1	A6QLY4; bta:540524		-0.024708987	-0.11321061	-0.088501623
A0AAF6ZXX8	CDC42	A0AAF6ZXX8	ENSBTAG00000001700	-0.040479069	-0.028797264	0.011681805
Q2KHU5	MACROD1	bta:613568; Q2KHU5	ENSBTAG00000017181	-0.048963152	-0.124825166	-0.075862015
A0A3Q1LTG9	TBC1D9B	A0A3Q1LTG9	ENSBTAG00000015611	-0.048440355	-0.019181413	0.029258942
E1BP11	RFX1	E1BP11; F1MGS1	ENSBTAG00000008854	0.04385429	0.001483491	-0.042370798
A0AAA9SER2	HAUS8	A0AAA9SER2	ENSBTAG00000007366	0.032487853	-0.021061616	-0.053549468
Q0Z7W6	TMX1	A3KN45; A5D9C0; bta:509037; Q0Z7W6	ENSBTAG00000011225	0.155248089	0.141926711	-0.013321378
A0A3Q1M407	CEP131	A0A3Q1M407	ENSBTAG00000000875	-0.015397105	-0.067405944	-0.052008839
A0AAA9TEH8	NACA	A0AAA9TEH8	ENSBTAG00000010701	-0.181592456	-0.409556426	-0.22796397
A0AAF6Z0Y9	RAD23A	A0AAF6Z0Y9	ENSBTAG00000015116	-0.147282677	-0.033326488	0.113956189
F1MCU6	QTRT1	F1MCU6	ENSBTAG00000037941	-0.370606425	-0.380097874	-0.009491449
A0AAF6DLY7	PAK4	A0AAF6DLY7	ENSBTAG00000013958	0.11059934	0.116758133	0.006158793
A0AAA9T7Y9	CYB5R1	A0AAA9T7Y9	ENSBTAG00000019927	0.00737953	0.014721506	0.007341975
P79251	ATP6V1G1	bta:281641; P79251; Q32KN3	ENSBTAG00000000203	0.147889919	0.057044212	-0.090845707
F1MBQ2	UBE2J1	bta:539754; F1MBQ2	ENSBTAG00000018436	-0.013734111	-0.238637752	-0.22490364
A0A452DIQ1	NAPG	A0A452DIQ1	ENSBTAG00000005158	-0.09046301	-0.125904589	-0.035441579
A0AAA9SXE3	MAP3K6	A0AAA9SXE3	ENSBTAG00000010254	-0.542849323	-0.593534689	-0.050685366

Accession	Gene_Symbol	Gene_ID	Ensembl_Gene ID	log2(ratio(CM12H/CM0H))	log2(ratio(CM12R/CM0H))	log2(ratio(CM12H/CM12R))
Q3ZCL8	SH3BGRL3	bta:614672; Q3ZCL8	ENSBTAG00000009580	0.050076872	0.148176314	0.098099441
F1N0K0	COL11A1	bta:287013; F1N0K0	ENSBTAG00000021217	-0.15664217	-0.83222296	-0.67558079
A0A3Q1MAA7	USP28	A0A3Q1MAA7	ENSBTAG00000002323	0.135808657	0.056122759	-0.079685898
A0AAA9S5W8	RAB12	A0AAA9S5W8	ENSBTAG00000010356	0.007494537	-0.006023788	-0.013518324
A0AAA9TVP9	FAM91A1	A0AAA9TVP9; bta:507013	ENSBTAG00000005025	-0.31358694	-0.266384418	0.047202521
A0AAA9SXX9	WAC	A0AAA9SXX9	ENSBTAG00000018263	-0.092503539	-0.114828086	-0.022324547
Q58DJ9	CHEK2	bta:518897; Q58DJ9		-0.047549352	0.079617168	0.12716652
Q5EA77	SNX17	bta:529972; Q17QP3; Q5EA77		-0.246970444	-0.155721382	0.091249062
E1BF38	DENND10	bta:534370; E1BF38	ENSBTAG00000008729	-0.140007479	-0.102218649	0.03778883
A0A3Q1MQW8	RPS18	A0A3Q1MQW8	ENSBTAG00000002648	-0.06573486	-0.144323595	-0.078588735
A0AAA9TAN7	ZFYVE1	A0AAA9TAN7	ENSBTAG00000011041	0.222891365	0.459749148	0.236857783
A5PK75	TOR1A	A5PK75; bta:533699	ENSBTAG00000020305	-0.015282929	0	0.015282929
F6PXF3	STAMBP	F6PXF3	ENSBTAG00000009600	0.022672468	0.081446178	0.058773709
A0AAA9ST58	LAMC2	A0AAA9ST58; bta:511043	ENSBTAG00000000793	-0.378640211	-0.186413124	0.192227087
A5PJG7	HCCS	A5PJG7; bta:506250	ENSBTAG00000012113	-0.108436232	0.011249141	0.119685373
Q66LN0	PTGES2	bta:493639; F1N1J6; Q66LN0		-0.061516924	-0.121400216	-0.059883292
A0AAA9TVC8	NDUFA7	A0AAA9TVC8	ENSBTAG00000052280	0.040458143	0.079812582	0.039354439
Q3T114	RIDA	bta:504390; Q3T114	ENSBTAG00000012595	0.074311809	0.150601738	0.076289929
Q3ZBW5	RHOB	bta:515118; Q3ZBW5	ENSBTAG00000061681	-0.131717858	-0.149980065	-0.018262206
A1L504	LMF2	A1L504; bta:510646; Q32PH4		-0.020218279	0.019938847	0.040157126
A0A0K1L4Y6	TRIM26	A0A0K1L4Y6		0.088641032	-0.036782789	-0.125423821
A0AAA9SKE5	SET	A0AAA9SKE5	ENSBTAG00000020959	0.119992699	0.108852141	-0.011140558
E1B8C2	NOM1	E1B8C2	ENSBTAG00000016435	-0.017552894	0.022636196	0.04018909
O18756	GLCE	bta:281195; O18756	ENSBTAG00000001405	-0.052841127	0.002879632	0.055720758
F1MKY0	GGA2	F1MKY0	ENSBTAG00000002352	-0.157367525	0.043024429	0.200391953
Q1RMQ1	FRG1	bta:512533; Q1RMQ1	ENSBTAG00000007089	0.151736397	0.061344987	-0.09039141
A0A3Q1NM68	STRN4	A0A3Q1NM68	ENSBTAG00000005830	-0.301687837	-0.294890675	0.006797162
A6QLC8	TRAPPC12	A6QLC8; bta:615707; F1MZZ3	ENSBTAG00000008685	0.046910149	0.053488797	0.006578648
P48734	CDK1	bta:281061; P48734; Q2YDM6	ENSBTAG00000010109	-0.643273165	-0.275450304	0.367822861
G3MYQ9	ABCB8	G3MYQ9	ENSBTAG00000007759	-0.387118796	-0.435075253	-0.047956457
A0AAA9T614	APEH	A0AAA9T614	ENSBTAG00000011583	0.304022076	0.10418885	-0.199833225
A0AAA9TH62	MRPS7	A0AAA9TH62	ENSBTAG00000016133	-0.280022967	-0.272820281	0.007202686
F1N0E6	VWA5A	F1N0E6	ENSBTAG00000021215	0.56578553	0.65080895	0.085023421
F1MP72	NCOA7	F1MP72	ENSBTAG00000046503	0.357426776	0.319872973	-0.037553803
Q3SZI7	COG6	bta:509305; Q3SZI7		-0.275966639	-0.13901896	0.136947679
A0A3Q1MGD3	LRBA	A0A3Q1MGD3	ENSBTAG00000018546	0.020005692	0.016945892	-0.0030598
F1N2G5	ARHGEF5	F1N2G5	ENSBTAG00000001815	0.057030945	0.013510286	-0.043520659
Q0VCT3	TERF2IP	bta:509625; Q0VCT3	ENSBTAG00000015686	0.160828631	-0.049243519	-0.21007215
A0AAF6YTE4	MRPL50	A0AAF6YTE4	ENSBTAG00000008962	-0.227788908	-0.001293317	0.226495591
A0AAA9T1I0	NCDN	A0AAA9T1I0	ENSBTAG00000016696	-0.129407794	-0.097730583	0.031677212
Q32L87	DCAF7	bta:516678; F1MVU0; Q32L87	ENSBTAG00000003500	0.076981357	-0.070314733	-0.147296091
Q148H9	RRP1	A0A3Q1N7Y1; bta:512925; Q148H9	ENSBTAG00000003981	-0.03796785	-0.136032137	-0.098064286
E1BHL1				0.282292023	0.591946329	0.309654307
P22439	PDHX	bta:517402; P22439; Q0P576	ENSBTAG00000018261	0.011194586	-0.018378529	-0.029573115
P61356	RPL27	bta:404137; P61356; Q3T0P1	ENSBTAG00000017441	-0.160151842	-0.12782942	0.032322422
F1MHT2	PIP4K2B	bta:539211; F1MHT2	ENSBTAG00000006499	0.16748408	0.116858007	-0.050626073
A0AAA9SZ52	TIGAR	A0AAA9SZ52	ENSBTAG00000016650	0.233686891	0.116554257	-0.117132634
A0A3Q1MRS1	EIF4E2	A0A3Q1MRS1	ENSBTAG00000054466	0.10359389	-0.108479101	-0.212072991
A0A452DHW2	NAXE	A0A452DHW2	ENSBTAG00000000404	0.027804405	0.043022849	0.015218443
F1N0Y0	UPP1	F1N0Y0	ENSBTAG00000008428	-0.083009776	-0.015596855	0.067412921
Q2KII4	ELOC	bta:540859; Q2KII4	ENSBTAG00000050514	0.131450854	0.333273068	0.201822215
A0A452DJI7	FOXO1	A0A452DJI7	ENSBTAG00000044105	0.046182101	0.054952311	0.00877021
Q08DN3	CDK5RAP1	bta:514086; Q08DN3	ENSBTAG00000018535	-0.84362841	-0.297794488	0.545833921
A0AAA9T2Y0	MVB12B	A0AAA9T2Y0	ENSBTAG00000050426	-0.139976009	-0.195471122	-0.055495113
A0A3Q1NEF8	RAC1	A0A3Q1NEF8	ENSBTAG00000053390	-0.031154523	-0.02815827	0.002996252
A0AAA9T5D9	UBE2V1	A0AAA9T5D9	ENSBTAG00000027316	0.141260713	-0.02446524	-0.165725953
A0A3Q1M8I7	BABAM2	A0A3Q1M8I7	ENSBTAG00000031335	-0.103792486	0.002899891	0.106692377
E1BP38	RALGAPA1	E1BP38	ENSBTAG00000001282	-0.373079825	-0.222947838	0.150131987
A0AAF6Z3Q7	METTL1	A0AAF6Z3Q7	ENSBTAG00000016908	-0.09723729	-0.094484054	0.002753236
A0A452DIF2	CLTB	A0A452DIF2	ENSBTAG00000010740	0.075483734	0.072350844	-0.003132889
A0AAA9RW61	TRIM36	A0AAA9RW61	ENSBTAG00000014027	0.10842246	0.082071133	-0.026351327
P00423	COX4I1	bta:281090; P00423; Q3SZS0; Q5E991	ENSBTAG00000016079	0.146401448	-0.164480863	-0.310882311
Q2KHX8	RCL1	bta:768075; Q2KHX8		-0.262099412	-0.296146893	-0.034047481
Q3T0F9	GRB2	bta:535298; Q3T0F9		0.199106232	0.058454063	-0.140652169
Q2KHW4	SLC25A15	bta:532560; Q2KHW4	ENSBTAG00000011647	0.145033539	0.127460909	-0.017572629
G3N166	PRPF38B	bta:100299693; G3N166	ENSBTAG00000047874	0.321325591	0.08905649	-0.232269101
A0A3Q1N3W6	MTA3	A0A3Q1N3W6	ENSBTAG00000004073	-0.071576933	0.045367184	0.116944116
A0A3Q1NJD5	BICC1	A0A3Q1NJD5	ENSBTAG00000010694	-0.070071238	-0.168533877	-0.098462639
A0AAA9SBV1	IWS1	A0AAA9SBV1	ENSBTAG00000021172	0.059307094	-0.00443679	-0.063743884
Q5E9Q4	NECAP2	bta:509439; Q5E9Q4		0.059501012	-0.016662688	-0.0761637
A0AAA9U1G3				-0.106409474	-0.083932758	0.022476716
A0A3Q1LX25	PLEKHG3	A0A3Q1LX25	ENSBTAG00000001551	-0.166679342	-0.107915339	0.058764003
A0AAA9RZH3	NCOR1	A0AAA9RZH3	ENSBTAG00000013271	-0.075027045	-0.220768679	-0.145741633
A0AAA9SQ70	GOSR1	A0AAA9SQ70	ENSBTAG00000004961	0.208586622	0.09461143	-0.113975192
G1K1P8				0.152324371	0.12900758	-0.023316791
Q1RML0	PRCC	bta:507189; Q1RML0	ENSBTAG00000000608	0.018935189	0.100512778	0.081577589
Q5BIN5	PIN1	bta:535470; Q5BIN5	ENSBTAG00000016988	-0.133743706	-0.101650076	0.03209363
F1MDM4	REEP5	F1MDM4	ENSBTAG00000012804	0.177207358	0.192287931	0.015080573
A4FUI1	MIX23	A4FUI1; bta:508149	ENSBTAG00000063883	0.080005643	0.180213745	0.100208102
A6QQV3	LSM2	A6QQV3; bta:538662		0.085094161	0.197128564	0.112034403
F1N6Q4	UPRT	F1N6Q4	ENSBTAG00000004549	0.010033687	-0.014455983	-0.02448967
F6QAZ2	PCDHGB4	F6QAZ2	ENSBTAG00000017349	-0.119064839	-0.188287211	-0.069222372
E1BEP7	ELP2	bta:511041; E1BEP7	ENSBTAG00000018954	-0.035306869	0.13317327	0.168480139
A0AAA9SSY0	CRELD1	A0AAA9SSY0	ENSBTAG00000010835	0.19885878	0.432368865	0.233510085
Q3T045	SMNDC1	bta:520500; Q3T045		0.307790984	-0.110342429	-0.418133413
Q3SZE9	TBCC	bta:515354; Q3SZE9	ENSBTAG00000000940	0.064192379	0.092574181	0.028381801
P79345	NPC2	bta:280815; P79345; Q3T091; Q58DR4		0.118799661	0.223515141	0.10471548
G5E5P6	AGPAT3	G5E5P6	ENSBTAG00000020456	0.049366077	-0.046392905	-0.095758983
F6RIB6	TMCC1	F6RIB6	ENSBTAG00000006614	-0.082062854	-0.067760927	0.014301927
A0A3Q1MFB9	PLD2	A0A3Q1MFB9	ENSBTAG00000013392	-0.25276607	-0.200846891	0.051919179

Accession	Gene_Symbol	Gene_ID	Ensembl_Gene ID	log2(ratio(CM12H/CM0H))	log2(ratio(CM12R/CM0H))	log2(ratio(CM12H/CM12R))
Q3ZCH9	HDHD2	bta:505403; Q3ZCH9	ENSBTAG00000011893	0.007581179	-0.012213364	-0.019794543
F1MCN4	STARD13	F1MCN4	ENSBTAG00000021815	-0.178507196	-0.196155062	-0.017647866
Q3T0D5	RPL30	bta:513031; Q3T0D5	ENSBTAG00000016278	-0.248636977	-0.184360092	0.064276886
F1N431	FDPS	F1N431	ENSBTAG00000003948	0.061938603	0.050759679	-0.011178924
F6R6Q1	FLAD1	bta:506682; F6R6Q1	ENSBTAG00000000186	-0.131951131	-0.156543119	-0.024591988
Q0VC37	DFFA	bta:507981; F1N6C7; Q0VC37	ENSBTAG00000013535	0.125412195	0.087043904	-0.038368292
A0AAA9SES4	AURKA	A0AAA9SES4	ENSBTAG00000013009	0.314556106	0.308631272	-0.005924834
G3MYZ3	AFM	bta:508264; G3MYZ3		-0.226770862	0.209847168	0.43661803
F1MW60	PDXP	F1MW60	ENSBTAG00000030255	0.077265126	0.112241265	0.034976138
E1BGZ1	DUS3L	E1BGZ1	ENSBTAG00000011842	-0.336705748	-0.110480866	0.226224882
P23934	NDUFS6	bta:327691; P23934	ENSBTAG00000009914	-0.066597749	-0.056535319	0.01006243
A6QLV2	URB2	A6QLV2; bta:514623		-0.387111063	-0.424979248	-0.037868186
A0AAA9TP48	HARS2	A0AAA9TP48	ENSBTAG00000025212	-0.096470718	0.154371079	0.250841798
A0AAA9STE1	ACAP2	A0AAA9STE1	ENSBTAG00000054986	0.090280842	0.096701391	0.006420549
Q2TBU2	TMX2	bta:509244; Q2TBU2; Q5E937	ENSBTAG00000002404	0.283895296	0.118229718	-0.165665578
A0A3Q1M5L7	CNOT2	A0A3Q1M5L7	ENSBTAG00000003748	0.001462438	0.037549738	0.0360873
A0AAA9SE78	ACOX3	A0AAA9SE78	ENSBTAG00000004893	-0.007819504	-0.175589914	-0.167770409
A0A3Q1MRP0	SPAST	A0A3Q1MRP0	ENSBTAG00000021694	0.13681669	0.034333732	-0.102482958
G3N1U2	HRAS	G3N1U2	ENSBTAG00000046644	-0.082800305	-0.051190699	0.031609607
G3N0D7	CBLB	G3N0D7	ENSBTAG00000013057	-0.105333518	-0.264578221	-0.159244703
Q5E9Q1	POGLUT1	bta:511862; Q5E9Q1	ENSBTAG00000008562	-0.020659471	-0.064316824	-0.043657354
Q95KV7	NDUFA13	bta:338084; Q3T0G4; Q95KV7		-0.030696797	-0.060740423	-0.030043626
A0A3S5ZPK5	WDR81	A0A3S5ZPK5	ENSBTAG00000011491	-0.209572117	-0.055519949	0.154052169
A0AAF6YSG6	EIF2B2	A0AAF6YSG6	ENSBTAG00000008664	0.117895239	-0.042569433	-0.160464672
F1MXE2	SIK3	bta:506539; F1MXE2	ENSBTAG00000015119	0.205722749	0.15268538	-0.053037369
A0AAA9SVY3	PPP3R1	A0AAA9SVY3	ENSBTAG00000010619	0.246664445	0.545592777	0.298928333
F1MCQ6	INPP5F	F1MCQ6	ENSBTAG00000021801	0.093794933	0.059113094	-0.034681839
Q3ZBZ1	SDF4	bta:528783; Q3ZBZ1		0.030072152	-0.045570316	-0.075642467
A0AAA9SKY4	SPAG5	A0AAA9SKY4	ENSBTAG00000013100	0.014631921	0.196316994	0.181685073
A0AAA9TJA1	CROT	A0AAA9TJA1	ENSBTAG00000021535	-0.205886427	-0.193843828	0.012042599
F1MD78	FNBP4	F1MD78	ENSBTAG00000020911	-0.11983977	-0.077550853	0.042288917
A0AAF6YNC4	COPS7B	A0AAF6YNC4	ENSBTAG00000005207	0.145940361	0.173712057	0.027771696
Q17QN2	TOE1	bta:513052; Q17QN2	ENSBTAG00000011243	-0.110580541	-0.217183754	-0.106603214
A0AAA9SV38	RNF114	A0AAA9SV38	ENSBTAG00000027317	0.158637512	-0.008699695	-0.167337207
A0A3Q1LX70	RAB34	A0A3Q1LX70	ENSBTAG00000046129	-0.143029774	-0.173808436	-0.030778662
F1MNI0	SAMD4A	F1MNI0	ENSBTAG00000001468	-0.058837824	-0.108566357	-0.049728534
F1MSF6	UTP3	bta:521106; F1MSF6	ENSBTAG00000009310	0.227887101	-0.363774835	-0.591661936
A0AAA9SUW9	CARMIL1	A0AAA9SUW9	ENSBTAG00000016549	0.408084739	0.093976148	-0.31410859
F1MZZ9	CCDC50	F1MZZ9	ENSBTAG00000006945	-0.066658094	-0.144995067	-0.078336972
Q08DB5	STX5	bta:510312; Q08DB5	ENSBTAG00000009490	0.137367041	-0.004510775	-0.141877816
Q29460	PAFAH1B3	bta:282515; Q0VCF0; Q29460		0.030038826	0.071409261	0.041370434
P21327	INPP1	bta:281869; P21327; Q1RMJ2	ENSBTAG00000007584	0.068608443	0.061462836	-0.007145607
A0AAF6YTE2	NSDHL	A0AAF6YTE2	ENSBTAG00000009231	-0.163116612	-0.09739243	0.065724182
P81187	CFB	bta:514076; P81187; Q2KIU6	ENSBTAG00000046158	-0.285547659	0.269483657	0.555031316
A6QLU7	TCAF2	A6QLU7; bta:510320		-0.038083502	0.042728544	0.080812046
F6RA71	VAT1L	F6RA71	ENSBTAG00000018146	0.145535319	0.068356592	-0.077178727
A0A3S5ZP61	PSMD10	A0A3S5ZP61	ENSBTAG00000013721	0.113520864	0.153049229	0.039528364
E1BGX5	ZNF787	E1BGX5	ENSBTAG00000002904	-0.050486193	-0.042396294	0.0080899
F1MNP7	SPC24	F1MNP7	ENSBTAG00000006185	0.134130701	-0.069367408	-0.20349811
A0AAA9S006	MRPS27	A0AAA9S006	ENSBTAG00000001962	-0.195942271	-0.231114541	-0.03517227
A7YY28	ABHD14B	A7YY28; bta:615289	ENSBTAG00000037377	0.063167471	0.104937193	0.041769722
A0A3Q1LSX9	FMNL2	A0A3Q1LSX9		0.045023925	-0.112362365	-0.15738629
A0AAA9S6N7	ERO1B	A0AAA9S6N7	ENSBTAG00000034633	-0.320442567	-0.201732951	0.118709616
E1B716	SHC1	E1B716	ENSBTAG00000019838	-0.135397912	-0.195256291	-0.05985838
A0AAF6YMI2	ABHD10	A0AAF6YMI2	ENSBTAG00000004601	0.125664311	0.243961165	0.118296854
A0AAA9S5X7	AURKB	A0AAA9S5X7	ENSBTAG00000001717	0.044311474	0.130295626	0.085984152
A0AAA9TP92	CAMK1	A0AAA9TP92	ENSBTAG00000007780	0.2410081	0.330221302	0.089213202
E1BFH7	SMC6	E1BFH7	ENSBTAG00000018256	-0.035222473	-0.01614974	0.019072733
A0AAF7APX7	WARS2	A0AAF7APX7	ENSBTAG00000005064	-0.072684454	-0.150768174	-0.07808372
F1MZ33	DNASE2	F1MZ33	ENSBTAG00000006082	-0.111520444	-0.085341792	0.026178652
F1N579	EPN1	F1N579	ENSBTAG00000046996	0.118148876	0.007224327	-0.110924549
A0AAA9RXY8	PRKRA	A0AAA9RXY8	ENSBTAG00000001096	0.11213831	0.098239407	-0.013898904
G3X6L8	NIPSNAP3A	G3X6L8	ENSBTAG00000006901	-0.025090981	0.082856932	0.107947913
F1MYQ1	TXNDC9	F1MYQ1	ENSBTAG00000004544	0.049944431	-0.225690011	-0.275634443
A0AAA9TRE9	SERPINF1	A0AAA9TRE9	ENSBTAG00000009705	-0.281201118	0.198370823	0.479571942
A0A3Q1MWG1	CARM1	A0A3Q1MWG1	ENSBTAG00000012048	-0.377157616	-0.466050665	-0.08889305
A0A3Q1MR53	ZNF217	A0A3Q1MR53	ENSBTAG00000030556	-0.057387482	-0.194751622	-0.13736414
A0AAF6DLT9	GTF2H1	A0AAF6DLT9	ENSBTAG00000005160	-0.134904073	0.012950671	0.147854743
A0AAA9TCG7	POLR2E	A0AAA9TCG7	ENSBTAG00000020776	-0.172445874	-0.135087623	0.037358251
A0A3Q1LSC1	MLLT1	A0A3Q1LSC1	ENSBTAG00000002277	0.133538258	0.077099112	-0.056439146
P00129	UQCRB	bta:616871; P00129; Q3SZ83	ENSBTAG00000001521	-0.145273893	-0.107213292	0.038060601
A0AAA9T797	YY1	A0AAA9T797	ENSBTAG00000020819	-0.017842746	-0.039809866	-0.02196712
F6PR59	ARMC6	F6PR59	ENSBTAG00000013291	-0.077214628	-0.146182913	-0.068968285
F1MVT2	DCAF11	F1MVT2	ENSBTAG00000021392	-0.046465045	-0.084411184	-0.037946139
P61420	ATP6V0D1	bta:282148; P12953; P61420; Q02547; Q3T0P9		0.3845873	0.490813374	0.106226073
Q58CT1	MVK	Q58CT1		0.057104451	0.049270793	-0.007833657
A0AAA9SPV1	CISD1	A0AAA9SPV1	ENSBTAG00000003634	-0.099067342	-0.053439259	0.045628083
A0AAF6DM76	INPP5K	A0AAF6DM76	ENSBTAG00000011479	-0.068468129	0.096970088	0.165438217
E1BLD4	RBM28	E1BLD4	ENSBTAG00000017001	-0.004833154	0.100928909	0.105762063
O77689	CCNB2	bta:281668; O77689; Q17QG1; Q5BIS1; Q5E9U8; Q5EAA0		-0.190697045	-0.076418306	0.114278739
F1MMK3	DDX50	bta:534331; F1MMK3	ENSBTAG00000033413	0.076062206	0.010278787	-0.065783419
Q08D91	KRT75	bta:539683; Q08D91		-0.120645354	0.073462162	0.194107516
Q1LZ72	CNPY2	bta:506534; F1MSP1; Q1LZ72	ENSBTAG00000004374	0.177231523	0.17994081	0.002709287
A0A3Q1NA56	NOC3L	A0A3Q1NA56	ENSBTAG00000011368	-0.066919592	-0.132607495	-0.065687903
A0AAA9TY37	PTRH2	A0AAA9TY37	ENSBTAG00000055796	-0.005894574	0.030557467	0.036452041
F6R1P1	TOR3A	F6R1P1	ENSBTAG00000014006	-0.13983442	-0.055393634	0.084440786
E1BMD5	AVEN	E1BMD5	ENSBTAG00000044029	0.057278971	-0.035206604	-0.092485575
Q5EA76	INTS14	bta:534159; Q5EA76	ENSBTAG00000015158	-0.017904798	-0.128769815	-0.110865017
A0AAA9TME5	CDKN2A	A0AAA9TME5	ENSBTAG00000034220	-0.619041056	0.015833874	0.63487493

Accession	Gene_Symbol	Gene_ID	Ensembl_Gene ID	log2(ratio(CM12H/CM0H))	log2(ratio(CM12R/CM0H))	log2(ratio(CM12H/CM12R))
A7MBI4	DHX32	A7MBI4; bta:532978; F1N3H3	ENSBTAG00000005795	0.040206322	-0.189065188	-0.22927151
F1MDA7	FBXO7	F1MDA7	ENSBTAG00000020634	0.028074952	0.017221741	-0.010853211
E1BLP4	MFF	E1BLP4	ENSBTAG00000021319	0.136107592	-0.111645356	-0.247752948
A6QLB8	GSK3A	A6QLB8; bta:536561; F1MHW8		-0.022694547	0.13355033	0.156244878
F6QFI8	NOP53	F6QFI8	ENSBTAG00000021192	-0.256374094	-0.502088319	-0.245714225
Q56JY1	RPL35A	bta:506768; Q56JY1	ENSBTAG00000014208	-0.340327933	-0.154220919	0.186107015
A0A3Q1MC59	DOCK5	A0A3Q1MC59	ENSBTAG00000015513	-0.204735765	-0.176446721	0.028289044
P60984	GMFB	A7E313; bta:615255; P17774; P60984	ENSBTAG00000054095	0.024271543	0.036957756	0.012686213
E1B828	LEMD2	bta:518023; E1B828	ENSBTAG00000015538	0.1023208	0.033947332	-0.068373468
F1MPB1	GNPTAB	F1MPB1	ENSBTAG00000019409	0.012744739	-0.008988783	-0.021733522
Q58CX1	GALT	bta:506997; F1MQ04; Q58CX1		-0.026472211	0.038819249	0.06529146
G3N2J7	DMXL1	G3N2J7	ENSBTAG00000006748	-0.168315749	-0.138076135	0.030239613
A0A3Q1LX85	FAM169A	A0A3Q1LX85	ENSBTAG00000016002	0.102743728	-0.099716792	-0.202460519
P82908	KGD4	bta:613835; P82908; Q3SYR1		-0.007195501	0.110499541	0.117695043
Q7YRA3	EXOSC4	bta:618292; Q32L11; Q7YRA3	ENSBTAG00000014607	-0.454946221	-0.335144699	0.119801522
Q2TBK2	MRPL9	bta:505981; Q2TBK2	ENSBTAG00000000797	-0.255416034	-0.258360311	-0.002944277
A0AAA9TGL5	FAM162A	A0AAA9TGL5	ENSBTAG00000011140	0.204452685	0.191396532	-0.013056153
A0AAA9TNC5	RPUSD2	A0AAA9TNC5	ENSBTAG00000057394	-0.294743266	-0.246721751	0.048021515
F1MH28	TLE1	F1MH28	ENSBTAG00000000256	0.035441579	0.004478105	-0.030963473
A0AAA9T0I3	RMDN1	A0AAA9T0I3	ENSBTAG00000015734	-0.343098795	-0.29074339	0.052355405
A0A3Q1M2R1	RC3H1	A0A3Q1M2R1	ENSBTAG00000052708	0.06168595	-0.064441753	-0.126127703
E1BG99	EIF4ENIF1	E1BG99	ENSBTAG00000008991	0.207232614	0.065914117	-0.141318497
A7E3S4	RAF1	A7E3S4; A7YY51; bta:521196	ENSBTAG00000045748	-0.120941037	-0.079490594	0.041450443
A0AAA9SZG2	CEP41	A0AAA9SZG2	ENSBTAG00000007413	0	-0.125251263	-0.125251263
A0A0A8J2N9	ASCT2	A0A0A8J2N9; bta:282355		-0.279737285	-0.370748223	-0.091010939
F6RJ01				0.124498542	-0.030031163	-0.154529705
P00435	GPX1	A6QPG3; bta:281209; P00435	ENSBTAG00000054195	-0.244575041	0.079071571	0.323646612
Q6EWQ6	DHPS	bta:444988; Q3MHI1; Q6EWQ6	ENSBTAG00000006247	-0.234538859	-0.339869554	-0.105330695
A0AAA9SLY8	RICTOR	A0AAA9SLY8	ENSBTAG00000006697	-0.222848321	-0.107862234	0.114986087
E1BM03	WDR3	bta:514870; E1BM03	ENSBTAG00000019937	-0.189126308	-0.255654875	-0.066528568
Q2TBN7	MED27	bta:525389; Q2TBN7	ENSBTAG00000000382	-0.039840265	-0.024057267	0.015782997
A0AAA9TLZ1	FBLN1	A0AAA9TLZ1	ENSBTAG00000012088	-0.155037154	-0.073043781	0.081993372
Q2TA12	MRPL2	bta:514403; Q2TA12	ENSBTAG00000012752	-0.161589142	-0.075653771	0.085935372
A0AAA9SEU1	SNX30	A0AAA9SEU1	ENSBTAG00000044070	-0.059364971	-0.098612792	-0.039247821
A0A3Q1MCZ1	PURB	A0A3Q1MCZ1; bta:504276	ENSBTAG00000052945	-0.074613061	-0.100886755	-0.026273694
A7Z063	WASHC1	A7Z063; bta:533602	ENSBTAG00000011902	-0.039312924	-0.034538419	0.004774505
Q0VCP0	SLC33A1	bta:517797; F1N5E5; Q0VCP0	ENSBTAG00000014318	0.004732738	-0.104850306	-0.109583045
A0A3Q1M4W7	EHMT2	A0A3Q1M4W7	ENSBTAG00000005676	-0.254284144	-0.162402043	0.091882101
Q3SZ40	DDX56	bta:508728; Q3SZ40		-0.001289848	-0.319030146	-0.317740298
A0AAA9SWX2	NF2	A0AAA9SWX2	ENSBTAG00000013153	-0.026258066	0.028625841	0.054883907
A0A3Q1MG93	OSBPL1A	A0A3Q1MG93	ENSBTAG00000023259	-0.184232838	-0.193850841	-0.009618003
A0A3Q1LKT6	STXBP5	A0A3Q1LKT6	ENSBTAG00000021107	-0.069149722	-0.117660595	-0.048510873
A0A3Q1NMC5	RPS24	A0A3Q1NMC5	ENSBTAG00000013264	-0.078698961	0.050626073	0.129325035
A0AAF6YTR5	POLR2G	A0AAF6YTR5	ENSBTAG00000009483	0.126300642	-0.288333814	-0.414634456
Q7M2Y2				-0.208470045	-0.179960323	0.028509722
A0AAA9THU3	EZH2	A0AAA9THU3	ENSBTAG00000009426	-0.222392421	-0.208760556	0.013631866
E1BAD4	RUNX1	bta:529631; E1BAD4	ENSBTAG00000004742	-0.120093845	-0.28299158	-0.162897735
F1N322	NISCH	F1N322	ENSBTAG00000014779	0.074663568	0.084425679	0.009762111
F1N498	MRPS11	F1N498	ENSBTAG00000019831	-0.029588185	-0.177265353	-0.147677168
F1MJA6	TEX2	F1MJA6	ENSBTAG00000004723	-0.202769295	-0.141671158	0.061098137
Q58D62	FETUB	bta:504615; Q58D62	ENSBTAG00000017531	-0.486082002	0.299150832	0.785232834
F1MMK9	AMBP	F1MMK9	ENSBTAG00000060375	-0.442897304	0.116386575	0.559283878
A0AAF7AAT7	ARMC8	A0AAF7AAT7	ENSBTAG00000019952	-0.048498518	0.049803556	0.098302074
A4IFE9	TM9SF1	A4IFE9; bta:539478		0.097297201	0.106811004	0.009513803
A0A3Q1LSN6	GSTM3	A0A3Q1LSN6	ENSBTAG00000001842	0.302028537	0.632746272	0.330717735
A0AAA9SVD5	LRIF1	A0AAA9SVD5	ENSBTAG00000012659	0.205378568	0.079617168	-0.1257614
A0AAA9TD00	CEBPB	A0AAA9TD00	ENSBTAG00000051972	0.626813007	0.576444719	-0.050368288
C7FFR6	DPM1	C7FFR6; F1MLE6	ENSBTAG00000007721	0.060720381	0.28757659	0.226856209
A0AAA9TS07	WDR45B	A0AAA9TS07	ENSBTAG00000003695	0.026299857	0.0539562	0.027656343
A0AAA9T7J6	TNFAIP2	A0AAA9T7J6	ENSBTAG00000035995	0.056583528	0.113700499	0.057116971
Q2TA37	ARL2	bta:511349; Q2TA37	ENSBTAG00000002238	0.03562391	-0.161280907	-0.196904817
Q2HJH8	CFAP300	bta:617430; Q2HJH8	ENSBTAG00000015309	-0.791333426	-0.419201124	0.372132302
A2VE31	SLC38A2	A2VE31; bta:338044; Q95M41	ENSBTAG00000011105	-0.147741853	-0.063803035	0.083938818
A0A3Q1LTM3	EPG5	A0A3Q1LTM3; bta:529859	ENSBTAG00000012067	-0.232923089	-0.134621015	0.098302074
A0AAA9S7H9	LYPLA1	A0AAA9S7H9	ENSBTAG00000004243	0.184553666	0.245717528	0.061163863
F1N5B5	REXO4	bta:100140713; F1N5B5	ENSBTAG00000019721	-0.305915777	-0.354495957	-0.04858018
A0AAA9TB64				0.046542586	0.070670857	0.024128271
Q2HJI6	GRN	Q2HJI6		0.02486038	-0.123860129	-0.148720509
A6QLI8	GTF2E1	A6QLI8; bta:540525	ENSBTAG00000016848	0.178677475	0.081079606	-0.097597869
A0A3Q1M3Z1	CD46	A0A3Q1M3Z1	ENSBTAG00000005397	0.096111893	0.025422597	-0.070689296
A0A3Q1MS48				-0.32828559	-1.097465809	-0.769180219
A4IFU2	RAP2B	A4IFU2; bta:784133	ENSBTAG00000055257	0.040466592	-0.007913871	-0.048380462
A0AAF6DME4	NPEPL1	A0AAF6DME4	ENSBTAG00000016724	-0.041552489	0.134604466	0.176156955
A6QLD9	GALNT16	A6QLD9; bta:526331	ENSBTAG00000009998	-0.136107592	-0.111757731	0.024349861
A0A3Q1LUF0	ARID5B	A0A3Q1LUF0	ENSBTAG00000051333	-0.098895756	0.059968589	0.158864345
E1BFH5	TSC22D4	E1BFH5	ENSBTAG00000015272	-0.041391631	-0.04589771	-0.004506079
A0AAF6YME1	RHOT2	A0AAF6YME1	ENSBTAG00000004551	-0.210845257	-0.233565333	-0.022720077
Q3SZ86	MRPS26	bta:516004; Q3SZ86		-0.084192056	-0.233939175	-0.14974712
A5PJN6	UTP6	A5PJN6; bta:614984		-0.117283522	-0.197811959	-0.080528437
A0AAA9TW47	GSTK1	A0AAA9TW47	ENSBTAG00000009839	-0.178337241	-0.191048293	-0.012711052
E1B7Z0	TAB2	bta:540203; E1B7Z0	ENSBTAG00000013454	-0.098603399	-0.132550731	-0.033947332
A2I7M9	SERPINA3-2	A2I7M9		-0.439601897	0.227481298	0.667083195
F1N4W0	PIGT	F1N4W0	ENSBTAG00000016122	-0.006286263	-0.014182807	-0.007896544
A0AAA9T5Y4	RUFY3	A0AAA9T5Y4	ENSBTAG00000016795	-0.046211265	-0.012688693	0.033522573
F1MPS0	NCAPD3	F1MPS0	ENSBTAG00000002260	-0.334211524	-0.140250719	0.193960805
F1MQH3	FERMT1	F1MQH3	ENSBTAG00000020465	-0.138414029	-0.141757472	-0.003343443
F1MS24	CHM	bta:519552; F1MS24	ENSBTAG00000009598	-0.119036291	-0.149128356	-0.030092065
A0AAA9TJA9	TMEM87A	A0AAA9TJA9	ENSBTAG00000008863	-0.037544369	0.060904119	0.098448488
Q2YDI0	MRPL11	bta:515990; Q2YDI0; Q58DQ9		-0.258093665	-0.145692922	0.112400743
A6QLY2	GPX7	A6QLY2; bta:523311		-0.019647902	-0.074367446	-0.054719545
E1BKU3				0.070243461	0.127444017	0.057200556

Accession	Gene_Symbol	Gene_ID	Ensembl_Gene ID	log2(ratio(CM12H/CM0H))	log2(ratio(CM12R/CM0H))	log2(ratio(CM12H/CM12R))
G5E5D4	MARK1	G5E5D4	ENSBTAG00000010460	-0.064384781	-0.007013602	0.057371179
A0A3Q1MRI3	CALCOCO1	A0A3Q1MRI3	ENSBTAG00000015016	-0.065365428	-0.090661723	-0.025296295
A8E4L9	SYNC	A8E4L9; bta:618122	ENSBTAG00000026684	-0.152562711	-0.323187541	-0.170624829
F1MDE6	APBB2	F1MDE6	ENSBTAG00000027569	-0.035333073	-0.121306296	-0.085973223
Q28036	SLC9A1	bta:317654; F1MHK2; Q28036	ENSBTAG00000008766	0.089210705	0.055548538	-0.033662167
A0AAA9TPX3	IL18	A0AAA9TPX3	ENSBTAG00000000277	-0.086217958	0.167857045	0.254075003
A0AAA9S0B2	GRB10	A0AAA9S0B2	ENSBTAG00000017086	-0.048044233	-0.089864409	-0.041820176
A4FUZ1	GLO1	A4FUZ1; bta:540335; F1MUW8	ENSBTAG00000012703	0.286881148	0.340562269	0.053681121
F1MYV2	KDM5C	F1MYV2	ENSBTAG00000014943	0.04822828	-0.116272122	-0.164500403
A0A3Q1N546	CNOT4	A0A3Q1N546	ENSBTAG00000017919	0.270010442	0.00965017	-0.260360272
Q3T0U5	CHMP2A	bta:523847; Q3T0U5	ENSBTAG00000013882	0.298919999	0.08246216	-0.216457839
A0A3Q1M2E0	WDR36	A0A3Q1M2E0	ENSBTAG00000053476	-0.179517644	-0.110978564	0.06853908
A0AAA9TT41	ARF6	A0AAA9TT41; bta:100294979	ENSBTAG00000068075	-0.267746533	-0.238159737	0.029586796
F1MYI1	ARNT	F1MYI1	ENSBTAG00000021037	0.316051529	0.061705961	-0.254345568
A0AAF6YSV5	WDR89	A0AAF6YSV5	ENSBTAG00000008815	-0.005013715	-0.09035927	-0.085345555
A0A3Q1LXI8	TMED3	A0A3Q1LXI8	ENSBTAG00000000807	0.130469932	0.146649859	0.016179927
Q0P5F7	PIP4K2C	bta:540005; Q0P5F7		0.129128627	0.247643098	0.118514471
A0A3Q1NM09	ARB2A	A0A3Q1NM09	ENSBTAG00000050195	-0.226275856	-0.273474827	-0.047198971
A0AAA9TRW7	ERGIC3	A0AAA9TRW7	ENSBTAG00000006670	0.093832741	0.081577589	-0.012255152
A0JND3	EOGT	A0JND3; bta:508782	ENSBTAG00000022681	-0.121990524	-0.070689296	0.051301229
F6RRD7	RPAP2	A4FV88; bta:529184; F6RRD7		0.077469295	-0.086468347	-0.163937642
Q5E938	EIF1	bta:509764; Q5E938	ENSBTAG00000002282	0.278301162	0.411802756	0.133501593
F1N2J9	RAB8B	F1N2J9	ENSBTAG00000011146	0.169925001	0.192848146	0.022923144
Q2KJD3	CWC15	bta:535258; Q2KJD3	ENSBTAG00000007913	0.192816981	-0.115935725	-0.308752706
A0AAA9SKM0	ADARB1	A0AAA9SKM0	ENSBTAG00000017486	0.033349417	0.05825092	0.024901503
G3MXZ7	NAGLU	G3MXZ7	ENSBTAG00000047956	0.06453066	0.100762977	0.036232317
P56602	PPOX	bta:515770; E1BPX0; P56602	ENSBTAG00000021894	-0.089864409	-0.029839637	0.060024771
A0AAA9SXG0	YTHDC1	A0AAA9SXG0	ENSBTAG00000015572	0.257217079	-0.072931522	-0.330148602
A0AAA9T1I5	PDCL	A0AAA9T1I5	ENSBTAG00000030675	0.290179736	-0.035994259	-0.326173995
F1MFY1	MORC3	F1MFY1	ENSBTAG00000011876	-0.476438044	-0.262452791	0.213985253
A0A3Q1MQ73	POFUT1	A0A3Q1MQ73	ENSBTAG00000016231	0.072183849	-0.011216344	-0.083400193
A0AAA9RZJ0	TOM1L2	A0AAA9RZJ0	ENSBTAG00000003983	0.075206344	0.038093171	-0.037113173
A0AAF6Z0J2	OXA1L	A0AAF6Z0J2	ENSBTAG00000014820	-0.030102032	-0.066776478	-0.036674447
E1BDJ1	XPC	E1BDJ1	ENSBTAG00000007362	-0.072149786	-0.049429709	0.022720077
A0AAA9S5M1	BAG1	A0AAA9S5M1	ENSBTAG00000017845	-0.160854011	-0.213595473	-0.052741462
A0AAA9SNG8	PYM1	A0AAA9SNG8	ENSBTAG00000031146	0.041539195	0.052417249	0.010878054
A0AAA9TDU0	BLM	A0AAA9TDU0	ENSBTAG00000020301	0.165757229	-0.020814717	-0.186571946
F1MQV1	MPHOSPH10	F1MQV1	ENSBTAG00000009854	0.075101455	-0.068921683	-0.144023138
A0AAA9TPN6	SLU7	A0AAA9TPN6	ENSBTAG00000008457	0.228570996	0.039695701	-0.188875295
Q6TUI4	DICER1	bta:337871; Q6TUI4		-0.01282404	-0.076017867	-0.063193826
A0AAA9SVY0	COMMD4	A0AAA9SVY0	ENSBTAG00000007346	-0.010426026	-0.031506876	-0.021080851
F1MPW4	SMC5	bta:508938; F1MPW4	ENSBTAG00000018437	-0.323553667	-0.073316678	0.250236989
A6H7C1	MORF4L2	A6H7C1; bta:538442	ENSBTAG00000001311	-0.110627818	-0.876525704	-0.765897886
A0AAA9T4J2	MOCS3	A0AAA9T4J2	ENSBTAG00000066776	-0.367266051	-0.216279298	0.150986753
A0A452DHY5	MAGOH	A0A452DHY5	ENSBTAG00000003485	-0.206545168	-0.253992331	-0.047447162
A6QQC2	TNFRSF10A	A6QQC2		0.033065505	-0.315254089	-0.348319594
Q0VCY8	PEA15	bta:510586; Q0VCY8	ENSBTAG00000005793	-0.105082046	0.220688115	0.325770161
A5D9H4	CHAF1B	A5D9H4; bta:511654	ENSBTAG00000011880	-0.078569052	0.011424833	0.089993886
A0AAA9SQC4	ZNF330	A0AAA9SQC4	ENSBTAG00000000297	-0.037011784	-0.001354008	0.035657776
A0A3Q1LJ78	LPGAT1	A0A3Q1LJ78	ENSBTAG00000005137	0.158092259	0.105046024	-0.053046234
A0A3Q1MBW0	CRYL1	A0A3Q1MBW0	ENSBTAG00000011726	0.167218618	0.126399714	-0.040818905
G3MXQ8	MAN1B1	G3MXQ8	ENSBTAG00000046775	-0.382087077	-0.309483329	0.072603749
A0A3Q1M9P9	VCAN	A0A3Q1M9P9	ENSBTAG00000014906	-0.13762507	-0.055757284	0.081867786
E1BNX1	RAB3D	E1BNX1	ENSBTAG00000065529	-0.241344863	-0.18551934	0.055825523
Q02373	NDUFB10	bta:327701; Q02373; Q3ZC99	ENSBTAG00000009534	-0.113435764	-0.199490411	-0.086054647
A0AAA9TUM5	APOOL	A0AAA9TUM5	ENSBTAG00000021790	0.111084111	-0.028797264	-0.139881375
Q2KJJ8	PLD3	bta:613932; Q2KJJ8	ENSBTAG00000019150	0.232258574	0.359241253	0.126982679
A0A3Q1MQS2	CXADR	A0A3Q1MQS2	ENSBTAG00000004869	0.314773376	0.251946538	-0.062826837
E1BPX6	POLR3B	E1B9R0; E1BPX6	ENSBTAG00000004781	-0.044477073	-0.088956744	-0.044479671
A0AAA9RXM2	TMEM263	A0AAA9RXM2	ENSBTAG00000053105	-0.001379909	-0.034899985	-0.033520076
F6PWS3	RALB	F6PWS3	ENSBTAG00000032021	0.101096485	0.214694872	0.113598386
A7MB64	ITPRIP	A7MB64; bta:538685	ENSBTAG00000003990	-0.272390081	-0.207611745	0.064778335
F1N0W0	HDAC3	bta:404125; F1N0W0	ENSBTAG00000017360	-0.213874793	-0.163998887	0.049875907
Q2HJH3				0.057796165	0.099352811	0.041556646
A0A3Q1N8U1	UBAP1	A0A3Q1N8U1	ENSBTAG00000000320	0.22841101	0.198275292	-0.030135718
Q1RMH8	SNX3	bta:528265; Q1RMH8	ENSBTAG00000018801	-0.064273214	0.123433941	0.187707155
Q2NL29	ISYNA1	bta:509394; Q2NL29		-0.133457798	-0.218281005	-0.084823207
G3MWH1	TPD52L2	G3MWH1	ENSBTAG00000021232	0.178337241	-0.009140654	-0.187477895
F1MHR3	ALDH1A3	F1MHR3	ENSBTAG00000009125	-0.079269102	-0.128242566	-0.048973463
A0AAF6YJA9	CYP51A1	A0AAF6YJA9	ENSBTAG00000001992	-0.425376479	-0.517932597	-0.092556118
E1BEL9	COASY	E1BEL9	ENSBTAG00000046746	-0.038881527	-0.027665182	0.011216344
P82924	MRPS30	bta:516084; P82924; Q05B75	ENSBTAG00000000084	-0.002766434	-0.100128034	-0.0973616
E1B887	RIOK1	bta:516289; E1B887	ENSBTAG00000013821	0.059156212	-0.2362424	-0.295398612
Q3ZBF7	PTGES3	bta:493638; Q3ZBF7; Q66LN1	ENSBTAG00000017967	0.243354247	0.207628544	-0.035725703
A0AAA9SHC7	FAM118B	A0AAA9SHC7	ENSBTAG00000012169	0.15803768	0.106812356	-0.051225323
Q08E26	CRTC2	bta:540959; Q08E26		-0.071384633	-0.066911157	0.004473477
F1N7R5	CC2D1B	F1N7R5	ENSBTAG00000015896	0.048328178	0.004748315	-0.043579863
Q58CQ5	ANKZF1	bta:507867; Q58CQ5		-0.19229039	-0.105578756	0.086711633
F1MF92	ZMYM4	F1MF92	ENSBTAG00000021023	-0.142566886	-0.066495412	0.076071474
Q2KIC5	ITPA	bta:613653; Q2KIC5		-0.102569734	-0.084460353	0.018109381
A0AAA9TJE2	TATDN1	A0AAA9TJE2	ENSBTAG00000033446	-0.021102693	-0.099735203	-0.07863251
F1MU45	PCF11	F1MU45	ENSBTAG00000015277	0.06909794	-0.018185472	-0.087283413
A0A3Q1N203	ARPIN	A0A3Q1N203	ENSBTAG00000027059	-0.004445905	0.001478929	0.005924834
A0AAF6Z667	S100A4	A0AAF6Z667	ENSBTAG00000019203	0.218527712	0.005393259	-0.213134453
A6QQS5	NELFA	A6QQS5; bta:789389; F1MW50	ENSBTAG00000017921	-0.269676895	-0.092090192	0.177586703
A0A3Q1M360	FBXO30	A0A3Q1M360	ENSBTAG00000005376	0.043439261	-0.044787922	-0.088227183
Q0VD41	CHN2	E1BEJ8; Q0VD41	ENSBTAG00000020931	-0.072679966	-0.044994137	0.027685829
Q3SZ15	EEF1E1	bta:617105; F1ML35; Q3SZ15	ENSBTAG00000002534	0.025535092	0.098032083	0.072496991
A0AAA9SHE6	KIRREL1	A0AAA9SHE6	ENSBTAG00000005799	0.123861913	0.007678012	-0.116183901
Q08DM8	TSN	A1L5B5; bta:509943; Q08DM8	ENSBTAG00000006059	-0.012407392	-0.017956226	-0.005548834
A0AAA9S4U6	RNF2	A0AAA9S4U6	ENSBTAG00000000023	0.06611114	-0.09105198	-0.15716312

Accession	Gene_Symbol	Gene_ID	Ensembl_Gene ID	log2(ratio(CM12H/CM0H))	log2(ratio(CM12R/CM0H))	log2(ratio(CM12H/CM12R))
A0A3Q1N6L4	ACOT8	A0A3Q1N6L4	ENSBTAG00000020371	0.049488183	0.032614364	-0.016873819
A7MB19	NLRX1	A7MB19; bta:539974		-0.005573845	0.036618591	0.042192436
A0AAF7A0B6	MBOAT7	A0AAF7A0B6	ENSBTAG00000015908	0.108252891	0.049582029	-0.058670862
A0A3Q1MIH5	SYNJ1	A0A3Q1MIH5	ENSBTAG00000003063	-0.037082257	0.032180406	0.069262662
A0A3Q1M1S3	CTIF	A0A3Q1M1S3; bta:615119	ENSBTAG00000018901	-0.133573026	-0.10555865	0.028014376
A3KN22	UBE2M	A3KN22; bta:613343	ENSBTAG00000012744	0.127129709	0.052203707	-0.074926002
G5E534	RPIA	bta:613376; G5E534	ENSBTAG00000002866	-0.033094401	-0.034689421	-0.00159502
A0A3Q1MJU3	IBTK	A0A3Q1MJU3	ENSBTAG00000026716	-0.055141554	-0.064540252	-0.009398698
A0AAA9S920	FAHD2A	A0AAA9S920	ENSBTAG00000011553	0.176077228	0.234725331	0.058648103
E1BI49	MAN1A2	bta:532645; E1BI49	ENSBTAG00000003586	-0.346429057	-0.147856224	0.198572833
A0AAA9TV39	BCL2L13	A0AAA9TV39	ENSBTAG00000013956	0.116275389	0.507984721	0.391709332
A0AAA9SZK0	NCBP2	A0AAA9SZK0	ENSBTAG00000008873	0.06020853	0.017056692	-0.043151838
P80747	ITGB5	A3KMX2; P80747		-0.002630256	-0.129118785	-0.126488529
A0AAA9T3R5	BCL9L	A0AAA9T3R5	ENSBTAG00000005138	-0.153070373	-0.193674227	-0.040603854
A0AAA9SNZ0	VPS37C	A0AAA9SNZ0	ENSBTAG00000008064	0.090093466	0.035707887	-0.054385579
A0AAF6YW51	PFN2	A0AAF6YW51	ENSBTAG00000011150	-0.094327383	-0.010181376	0.084146006
A0AAF7AT19	PKN1	A0AAF7AT19	ENSBTAG00000017037	-0.149692933	-0.140106884	0.009586049
A0AAA9TFC0	PDCD5	A0AAA9TFC0	ENSBTAG00000009304	-0.11645322	-0.320627784	-0.204174563
A6QLP7	SSR1	A6QLP7; bta:529312	ENSBTAG00000022731	0.148234624	-0.025311089	-0.173545712
A0AAA9SKF2	EIF2D	A0AAA9SKF2	ENSBTAG00000010432	-0.172069537	-0.105293059	0.066776478
A0AAF6ZEA8	AK4	A0AAF6ZEA8	ENSBTAG00000030674	0.097434151	0.056825815	-0.040608336
A0AAA9T9L5	GYG2	A0AAA9T9L5	ENSBTAG00000013341	-0.315096061	-0.211778747	0.103317314
Q3ZBX6	MRPL3	bta:614906; Q3ZBX6	ENSBTAG00000030942	-0.190365338	-0.120699347	0.069665991
E1BIZ3	UNC119B	bta:617896; E1BIZ3	ENSBTAG00000007483	0.0085115	-0.005702359	-0.014213859
A0A3Q1M1W2	PON2	A0A3Q1M1W2	ENSBTAG00000008361	0.15589701	0.058026945	-0.097870064
A2VDP0				0.178145508	0.121678557	-0.056466952
A0AAF6YT25	FAM136A	A0AAF6YT25	ENSBTAG00000008674	0.363513634	0.160613696	-0.202899939
A0AAA9T341	LOC532875	A0AAA9T341; bta:532875	ENSBTAG00000038051	-0.513012659	-0.381903755	0.131108903
A0AAA9T1Y1	NIP7	A0AAA9T1Y1	ENSBTAG00000001666	-0.021303513	-0.103485157	-0.082181644
A0AAA9TUB9	SSR4	A0AAA9TUB9	ENSBTAG00000021421	0.10443741	0.193006068	0.088568657
Q2T9M8	SNAP23	bta:522423; F1N473; Q2T9M8	ENSBTAG00000005661	0.187004402	0.19358305	0.006578648
A0A3Q1NA87	CRIM1	A0A3Q1NA87	ENSBTAG00000009020	-0.12294123	-0.257343294	-0.134402064
A0A3Q1LUZ8	TOP3B	A0A3Q1LUZ8; bta:537418	ENSBTAG00000053372	-0.214840824	-0.066196743	0.148644081
F1MG95	PARG	F1MG95	ENSBTAG00000023018	-0.216565958	-0.088273936	0.128292022
E1BL67	OSBPL2	E1BL67	ENSBTAG00000017402	0.139263982	0.163058282	0.0237943
A0AAA9S740	PEX14	A0AAA9S740	ENSBTAG00000013538	0.032945943	-0.004171651	-0.037117594
G3MWG5	XIAP	G3MWG5	ENSBTAG00000040350	-0.003370784	-0.042711676	-0.039340892
Q1LZG6	CCNB1	bta:327679; Q1LZG6	ENSBTAG00000014239	-0.091711404	0.21620855	0.307919954
A0A3Q1N402	BIRC2	A0A3Q1N402	ENSBTAG00000035735	-0.226370539	-0.079955305	0.146415235
A0AAF6YPV7	CHMP4A	A0AAF6YPV7	ENSBTAG00000039415	0.28484524	-0.020308982	-0.305154222
A0AAA9TX02	INTS12	A0AAA9TX02	ENSBTAG00000033648	-0.253012973	-0.119565699	0.133447274
F1N3F2	IRF2BPL	F1N3F2	ENSBTAG00000025329	-0.093689416	-0.062211184	0.031478231
A0A3Q1LVE2	SASS6	A0A3Q1LVE2	ENSBTAG00000015778	0.218680829	-0.08276746	-0.301448289
A0A3Q1MIH6	MAVS	A0A3Q1MIH6	ENSBTAG00000013545	0.003126101	-0.060720381	-0.063846482
A0AAA9SU87	SRFBP1	A0AAA9SU87	ENSBTAG00000014320	0.394311473	-0.007502331	-0.401813804
F6S1J8	YES1	F6S1J8	ENSBTAG00000001523	-0.159969921	-0.016923303	0.143046618
A0AAF6YUU2	EMC2	A0AAF6YUU2	ENSBTAG00000010006	0.065712841	0.012551317	-0.053161524
F1MSC2	CFAP97	F1MSC2	ENSBTAG00000013211	0.880900649	-0.228434373	-1.109335022
A0AAA9TEN0	ACADSB	A0AAA9TEN0	ENSBTAG00000018041	-0.090063698	-0.174349252	-0.084285554
E1BPZ7	FBXO38	E1BPZ7	ENSBTAG00000020303	-0.033271171	0.13273878	0.166009951
A0AAA9TRI4	CDC123	A0AAA9TRI4	ENSBTAG00000019511	0.096111893	-0.074000581	-0.170112474
Q95108	TXN2	bta:281557; Q2KHU7; Q95108	ENSBTAG00000000014	0.131865448	0.645569265	0.513703818
E1BNN3	RALGPS2	E1BNN3	ENSBTAG00000010718	0	-0.230698424	-0.230698424
A0A3Q1M9B2	ARHGAP21	A0A3Q1M9B2	ENSBTAG00000021857	0.11441485	0.109624491	-0.004790359
A0AAF6DLX2	NTN4	A0AAF6DLX2	ENSBTAG00000003183	0.278875242	-0.214124805	-0.493000047
A0A3Q1M9D2	MPST	A0A3Q1M9D2	ENSBTAG00000030648	0.064055394	0.142665229	0.078609835
F1ML97	NOA1A	F1ML97	ENSBTAG00000019362	-0.136136688	-0.192840446	-0.056703758
G5E6J3	MED16	G5E6J3	ENSBTAG00000047217	-0.180572246	-0.068057442	0.112514804
A0AAA9TXS8	MECP2	A0AAA9TXS8	ENSBTAG00000047855	0.127482448	0.097679237	-0.029803211
A0A3Q1LWP8	WDR35	A0A3Q1LWP8	ENSBTAG00000051164	-0.069708972	-0.076071474	-0.006362502
Q2NL17	CLPTM1	bta:618037; Q2NL17	ENSBTAG00000020560	-0.261410402	-0.306630795	-0.045220393
A0AAA9SCK0	FKBP1A	A0AAA9SCK0	ENSBTAG00000008303	0.054138173	0.192227087	0.138088914
A0A452DHW6	MYD88	A0A452DHW6	ENSBTAG00000000563	0.087462841	-0.016086984	-0.103549825
G3MZN5	NCKAP5L	G3MZN5	ENSBTAG00000004851	-0.096806593	-0.064729887	0.032076707
A0AAA9S5V1	USP25	A0AAA9S5V1	ENSBTAG00000019314	-0.20744208	-0.205790454	0.001651626
A0A452DHY9	PHF10	A0A452DHY9	ENSBTAG00000003929	0.466108876	-0.261227326	-0.727336202
Q08DZ5	SDC1	bta:529759; Q08DZ5		0.514573173	0.222607316	-0.291965857
A0AAA9SW70	VAMP3	A0AAA9SW70	ENSBTAG00000064040	0.067283417	0.23641855	0.169135133
A0AAA9U1C9	DNAJB12	A0AAA9U1C9	ENSBTAG00000013651	0.029927332	-0.171027919	-0.200955251
A0A8E6GNW9	COX2	A0A8E6GNW9		-0.178776413	-0.112058507	0.066717906
A0A3Q1LQ88	DAB2IP	A0A3Q1LQ88	ENSBTAG00000008232	0.001618278	0.02249236	0.020874082
Q08DT6	MRPL47	bta:534317; Q08DT6		-0.087628907	-0.080587921	0.007040985
Q0P5A5	CKS1B	bta:615827; Q0P5A5	ENSBTAG00000024476	-1.063094601	-0.600239361	0.462855239
A0AAF6ZCD9	TTC5	A0AAF6ZCD9	ENSBTAG00000009372	-0.383128251	-0.381626226	0.001502025
A0AAA9TGD7	YJU2	A0AAA9TGD7	ENSBTAG00000012830	-0.011950602	-0.160698049	-0.148747446
A0A452DJF8	NAGK	A0A452DJF8	ENSBTAG00000014668	-0.111703271	-0.065275909	0.046427362
Q0VCS9	ANKMY2	bta:509032; Q0VCS9	ENSBTAG00000010980	0.179428838	0.016101674	-0.163327163
E1BNI6	TMED8	E1BNI6	ENSBTAG00000020469	0.160214602	0.109740363	-0.05047424
A0A3Q1M7G4	INTS13	A0A3Q1M7G4	ENSBTAG00000013515	-0.043311171	-0.126743537	-0.083432365
G3N0S9				-0.553275406	-0.501332774	0.051942632
A0AAA9T7D0	BCAS3	A0AAA9T7D0	ENSBTAG00000002663	-0.064409713	-0.131829954	-0.067420241
F1MES6	UBXN6	F1MES6	ENSBTAG00000008186	0.142957954	0.088950923	-0.054007031
A0AAF6YLJ6	PAAF1	A0AAF6YLJ6	ENSBTAG00000003679	-0.091024174	0.035138002	0.126162176
F1MEK3	SCAF1	F1MEK3	ENSBTAG00000006612	0.124738969	-0.138046503	-0.262785472
A0A3Q1LL49	MORF4L1	A0A3Q1LL49	ENSBTAG00000010988	-0.18706836	-0.579385783	-0.392317423
F1MWY0	NSMAF	F1MWY0	ENSBTAG00000008958	-0.035985945	-0.156984477	-0.120998531
G3X7D8	MRRF	G3X7D8	ENSBTAG00000018426	0.172594188	0.064652958	-0.10794123
A0AAA9T4F5	PDCD6	A0AAA9T4F5	ENSBTAG00000046493	0.134333345	0.360310572	0.225977227
F1MX69	BPGM	F1MX69	ENSBTAG00000008895	0.195347598	-0.098891757	-0.294239355
A0AAF6Z8F0	CASP4	A0AAF6Z8F0	ENSBTAG00000020884	0.164945894	0.016678741	-0.148267153
A0A3Q1MF31	TRAPPC9	A0A3Q1MF31	ENSBTAG00000013955	-0.146841388	-0.227214805	-0.080373416

Accession	Gene_Symbol	Gene_ID	Ensembl_Gene ID	log2(ratio(CM12H/CM0H))	log2(ratio(CM12R/CM0H))	log2(ratio(CM12H/CM12R))
E1BDS1	MAGED1	bta:512562; E1BDS1	ENSBTAG00000008733	-0.17478454	-0.392903051	-0.218118511
A0AAF6YI61	ABHD12	A0AAF6YI61	ENSBTAG00000001420	-0.254572827	-0.326302742	-0.071729915
E1BMM4	VPS26B	bta:614321; E1BMM4	ENSBTAG00000014838	-0.051726275	0.088096694	0.13982297
E1BKB2	NGLY1	bta:533736; E1BKB2	ENSBTAG00000003793	-0.218894977	-0.298483242	-0.079588265
Q1LZ99	DMAP1	bta:508127; Q1LZ99	ENSBTAG00000005912	-0.066994306	-0.216947492	-0.149953186
A0A3Q1M4Y7	SBF1	A0A3Q1M4Y7	ENSBTAG00000021337	-0.091489844	-0.12228493	-0.030795087
A0A3Q1M7F4	CTU2	A0A3Q1M7F4	ENSBTAG00000020943	-0.105046024	-0.187251819	-0.082205795
A0AAA9TG97	ATG4B	A0AAA9TG97	ENSBTAG00000015401	0.065588342	0.144855821	0.079267479
Q5E992	PPIL1	bta:508179; Q5E992	ENSBTAG00000002376	-0.086177742	0.047217236	0.133394978
Q3SZ22	MRPL46	bta:540720; Q3SZ22	ENSBTAG00000019830	-0.039216396	-0.061183516	-0.02196712
Q2TBU5	NT5DC1	bta:100125914; Q2TBU5	ENSBTAG00000003076	-0.167007518	-0.12659325	0.040414268
A0A3Q1NJG8				-0.10436978	-0.11007778	-0.005708
Q0VCI1	OSGEP	bta:507512; Q0VCI1	ENSBTAG00000002743	0.070893855	0.20826635	0.137372495
A0AAA9S900	RBKS	A0AAA9S900	ENSBTAG00000031332	-0.080132602	-0.002644721	0.077487881
A0AAA9T820	AUP1	A0AAA9T820	ENSBTAG00000020123	-0.019533887	-0.029400916	-0.009867029
F1MFA7	CCDC186	F1MFA7	ENSBTAG00000020233	0.27992342	0.113062664	-0.166860755
A0AAA9T7X2	CDC16	A0AAA9T7X2	ENSBTAG00000019971	-0.148487831	0.010043666	0.158531497
A0AAF6YYP8	GLTPD2	A0AAF6YYP8	ENSBTAG00000048034	0.050323314	0.155218503	0.104895188
A0AAA9RY43	LACTB2	A0AAA9RY43	ENSBTAG00000001808	0.285945505	0.370337692	0.084392187
Q2TA08	BAG5	bta:522854; Q2TA08	ENSBTAG00000058752	0.162611193	0.01412687	-0.148484323
A4FUB8	TMEM115	A4FUB8; bta:532459		0.052614626	0.189033824	0.136419198
F1MJX8	DNAJC1	F1MJX8	ENSBTAG00000033315	0.068425141	-0.124781625	-0.193206766
Q3MHG6	GTPBP10	bta:613957; Q3MHG6		-0.221567789	-0.185943879	0.03562391
A0AAA9TT54	ST3GAL4	A0AAA9TT54	ENSBTAG00000016506	-0.186768512	-0.023898268	0.162870244
A0AAA9RT25	PTPRK	A0AAA9RT25	ENSBTAG00000020829	-0.082350058	-0.076309456	0.006040602
A0AAF6Z0X4	RABEPK	A0AAF6Z0X4	ENSBTAG00000015098	-0.153363485	-0.056855504	0.096507981
Q3SZ45	SDF2	bta:508463; Q3SZ45		0.154774843	0.158922507	0.004147665
A0AAA9SHM7	MED15	A0AAA9SHM7	ENSBTAG00000018048	-0.224560258	-0.031535573	0.193024685
A0AAA9U0U6	ACADL	A0AAA9U0U6	ENSBTAG00000010579	-0.106795234	-0.029942315	0.076852919
A0A3Q1LUC4	INPP4A	A0A3Q1LUC4	ENSBTAG00000004814	-0.13185143	0.054185576	0.186037006
A7YY65	MTCH1	A7YY65; bta:781257	ENSBTAG00000033453	-0.055432391	-0.062096121	-0.00666373
F1MB46	TBC1D10A	F1MB46	ENSBTAG00000018086	-0.130087519	-0.047973122	0.082114397
Q2HJ98	FAHD1	bta:509273; Q2HJ98	ENSBTAG00000033015	0.006286263	0.041910173	0.03562391
Q3SYZ9	MED4	bta:515299; Q3SYZ9	ENSBTAG00000019502	0	-0.09657326	-0.09657326
A0AAA9TZ37	SERPING1	A0AAA9TZ37	ENSBTAG00000016267	-0.167083372	0.250081683	0.417165055
A0A3Q1NE41	ARPP19	A0A3Q1NE41	ENSBTAG00000011022	0.119182916	-0.265667863	-0.384850779
Q0P584	ZMAT2	bta:782132; Q0P584	ENSBTAG00000005441	0.278615219	-0.016505271	-0.29512049
A0A3Q1MPE1	ZNF451	A0A3Q1MPE1	ENSBTAG00000018879	-0.091528095	-0.173677136	-0.082149041
A4FUE6	MOBKL3	A4FUE6; bta:781884	ENSBTAG00000019116	0.023526484	-0.032453188	-0.055979672
A0A3Q1MCI2	LRCH1	A0A3Q1MCI2	ENSBTAG00000002959	0.071000769	0.06009187	-0.010908899
A0AAF6Z7J4	VPS25	A0AAF6Z7J4	ENSBTAG00000019906	-0.022093771	-0.026553419	-0.004459648
Q3ZBG7	SRP19	bta:514960; Q3ZBG7	ENSBTAG00000012803	-0.12516355	-0.134005427	-0.008841877
A5D7H7	NOP9	A5D7H7; bta:528833; F1MK13	ENSBTAG00000010235	0.230452191	-0.156725504	-0.387177695
A0A3Q1M7I4	NR3C1	A0A3Q1M7I4	ENSBTAG00000019472	0.115863843	0.276654015	0.160790172
A0A3Q1MY50	ACBD5	A0A3Q1MY50	ENSBTAG00000017278	0.15425806	0.030306483	-0.123951577
A0AAA9T7H1	PTAR1	A0AAA9T7H1	ENSBTAG00000005826	-0.022720077	-0.09028336	-0.067563284
A6QQA9	ACOT2	A6QQA9; bta:511033	ENSBTAG00000046814	0.039683701	0.122974045	0.083290343
A0AAF6ZBD7	SRSF7	A0AAF6ZBD7	ENSBTAG00000014891	-0.062046138	-0.039678325	0.022367813
A0AAA9SNW6	RBM5	A0AAA9SNW6	ENSBTAG00000006330	0.41717641	-0.017226414	-0.434402824
A0AAF6YIL8	RPLP2	A0AAF6YIL8	ENSBTAG00000001777	-0.21169965	-0.145824519	0.065875131
A0A3Q1N184	NHSL1	A0A3Q1N184	ENSBTAG00000000245	-0.002704209	-0.030028795	-0.027324587
A0A3Q1ME54	PARP4	A0A3Q1ME54	ENSBTAG00000025400	-0.112879545	-0.102123148	0.010756397
F6QDZ3	USE1	F6QDZ3	ENSBTAG00000011134	-0.082961449	0.056855504	0.139816953
A0AAA9SJM0	NSA2	A0AAA9SJM0	ENSBTAG00000003066	-0.438749088	-0.233177835	0.205571252
Q3ZBE1	ARMC1	bta:514000; Q3ZBE1; Q5EAC8	ENSBTAG00000015901	0.067114196	0.006093758	-0.061020438
A0A3Q1MMP5	TSC2	A0A3Q1MMP5	ENSBTAG00000020617	-0.153606979	-0.192645078	-0.039038099
E1BBZ8	FOSL2	bta:509889; E1BBZ8	ENSBTAG00000023929	-0.158204433	-0.081838292	0.076366141
Q08DT8	RINT1	bta:510686; Q08DT8		-0.176020325	-0.095127344	0.080892981
A0AAA9SLU6	TEAD3	A0AAA9SLU6	ENSBTAG00000019495	-0.114761062	-0.021651658	0.093109404
F1N1U1	SYNJ2	F1N1U1	ENSBTAG00000000817	-0.236467607	-0.33146412	-0.094996514
A6QQR1	NSUN5	A6QQR1; bta:616714; E1BGI7	ENSBTAG00000017941	0.063043518	-0.174535307	-0.237578825
Q2KHT7	RPS27	A1XEE0; bta:615638; Q2KHT7		-0.087712176	-0.145370001	-0.057657825
E1BPU3	PEX1	E1BPU3	ENSBTAG00000024431	0.038261558	0.170463421	0.132201862
Q08DI5	RAP2C	bta:515181; Q08DI5	ENSBTAG00000004131	0.088832445	0.127729226	0.03889678
A0AAA9SJ23	TRMT10A	A0AAA9SJ23	ENSBTAG00000000197	0.121875951	0.043151838	-0.078724112
A0A3Q1ML70	ATP7A	A0A3Q1ML70	ENSBTAG00000010018	-0.029805877	0.084567772	0.114373649
A0AAA9S661	KIFAP3	A0AAA9S661	ENSBTAG00000017727	0.148741711	0.074078144	-0.074663568
A6H7J3	GAN	A6H7J3; bta:521175	ENSBTAG00000009428	-0.1718738	-0.180676115	-0.008802316
A0A3Q1MKU4	IL1RAP	A0A3Q1MKU4	ENSBTAG00000013205	-0.249435294	-0.14687727	0.102558025
A0AAA9T4Z5				-0.101910781	-0.341548149	-0.239637368
A0A3Q1LYV0	NHLRC3	A0A3Q1LYV0	ENSBTAG00000010951	0.43422315	0.393771106	-0.040452044
A0AAA9SX05	ANO10	A0AAA9SX05	ENSBTAG00000008893	0.011252275	0.194459409	0.183207134
A0AAA9RV38	PREB	A0AAA9RV38	ENSBTAG00000007080	-0.004626498	0.007678012	0.01230451
F1MDX7	FHIP2B	F1MDX7	ENSBTAG00000014361	0.006049045	0.032960199	0.026911154
A0A3Q1NIX3	CASC3	A0A3Q1NIX3	ENSBTAG00000006250	-0.045759661	-0.085757729	-0.039998068
A0A3Q1M9K0	TTC39B	A0A3Q1M9K0	ENSBTAG00000003943	0.276868372	0.14024338	-0.136624992
A7Z069	KRIT1	A7Z069	ENSBTAG00000002750	-0.054690642	-0.062911148	-0.008220507
A0A3Q1N041	GPCPD1	A0A3Q1N041; bta:518469	ENSBTAG00000008293	-0.396458703	0.300189436	0.696648139
Q3ZBV4	HMGN3	bta:515652; Q3ZBV4	ENSBTAG00000055765	0.049549589	0.12852838	0.07897879
F1MC22	CFAP298	F1MC22	ENSBTAG00000003059	-0.329084798	-0.305877407	0.023207391
A0AAA9SD49	IRF2BP1	A0AAA9SD49	ENSBTAG00000050077	0.268096032	0.17613855	-0.091957481
F1N293	CNNM3	F1N293	ENSBTAG00000010846	0.681359833	0.329806569	-0.351553264
A0AAA9THQ4	SOAT1	A0AAA9THQ4	ENSBTAG00000004059	-0.004829109	-0.062816108	-0.057986999
A6QLZ7	CRISPLD2	A6QLZ7; bta:505329		-0.197036847	-0.530514717	-0.33347787
A5PJT3	PRMT9	A5PJT3; bta:532021	ENSBTAG00000010381	0.062764795	-0.122432176	-0.185196971
F6QBF9	RBM8A	F6QBF9	ENSBTAG00000008369	-0.067469583	-0.30032584	-0.232856257
A0A3Q1LXL8	MTX1	A0A3Q1LXL8	ENSBTAG00000014840	0.018025257	0.016646666	-0.001378591
F1MFG7	BPHL	F1MFG7	ENSBTAG00000006379	-0.260764232	-0.182940815	0.077823418
A6H7J2	RPS19BP1	A6H7J2; bta:509108	ENSBTAG00000017463	0.049915887	0.044602087	-0.0053138
A0AAA9S7V3	NDUFB9	A0AAA9S7V3	ENSBTAG00000020405	-0.10103632	-0.173016271	-0.071979951
F1MX05	SCCPDH	F1MX05	ENSBTAG00000003804	-0.055522497	-0.052622607	0.002899891

Accession	Gene_Symbol	Gene_ID	Ensembl_Gene ID	log2(ratio(CM12H/CM0H))	log2(ratio(CM12R/CM0H))	log2(ratio(CM12H/CM12R))
Q3SZI0	MPI	bta:513586; Q3SZI0	ENSBTAG00000005845	0.097057998	0.186788159	0.089730161
E1B9G4	PPIL3	bta:615703; E1B9G4	ENSBTAG00000005416	-0.168229508	-0.053925882	0.114303627
A0AAA9S0E0	LYPLA2	A0AAA9S0E0	ENSBTAG00000011625	0.068849408	0.039131245	-0.029718163
A0AAF7AII2	DGKA	A0AAF7AII2	ENSBTAG00000004018	-0.157183333	0.014552756	0.171736088
A0AAA9TQR4	PEG10	A0AAA9TQR4	ENSBTAG00000059156	0.152122814	-0.052423078	-0.204545892
A0AAA9SIA3	XPNPEP3	A0AAA9SIA3; bta:539202	ENSBTAG00000006204	0.312717972	0.263034406	-0.049683566
Q5E9M1	ARL6IP5	bta:509977; Q5E9M1	ENSBTAG00000021506	0.187202993	0.29455432	0.107351327
A0A3Q1LWZ0	ORC4	A0A3Q1LWZ0	ENSBTAG00000015291	-0.072709322	-0.057019948	0.015689374
A0AAA9TCD6	MICOS13	A0AAA9TCD6	ENSBTAG00000001792	0.049421599	0.039943512	-0.009478087
A0A3Q1MIF4	CFI	A0A3Q1MIF4	ENSBTAG00000034501	-0.176943583	0.259750672	0.436694255
A0A452DK61	CISD2	A0A452DK61	ENSBTAG00000027630	0.281094377	0.194321656	-0.086772722
A0A3Q1MET1	SLC44A2	A0A3Q1MET1	ENSBTAG00000002628	0.051689477	0.156351838	0.104662361
F1MY58	TOM1L1	F1MY58	ENSBTAG00000007013	0.031445558	-0.154865418	-0.186310976
A0AAA9TGJ7	CHUK	A0AAA9TGJ7	ENSBTAG00000007591	0.023408221	-0.007887909	-0.03129613
F1MXI3	PI4KB	F1MXI3	ENSBTAG00000007320	0.246944703	-0.144319734	-0.391264438
Q5E9I4	MGAT1	bta:534248; Q5E9I4	ENSBTAG00000001546	0.093022367	0.159364861	0.066342495
G3X782	CHKB	bta:536213; G3X782	ENSBTAG00000016050	-0.145556313	-0.173390521	-0.027834208
F1MYH8	PSMD8	F1MYH8	ENSBTAG00000031641	0.019705409	0.046025108	0.026319699
Q17QB3	ASAH1	bta:510620; Q17QB3	ENSBTAG00000011257	-0.099127209	0.014227877	0.113355087
A7YWA1	POLR1F	A7YWA1; bta:512375; F1MNK1	ENSBTAG00000006721	-0.026782602	-0.084846577	-0.058063975
A0A3Q1LTB1	NFAT5	A0A3Q1LTB1	ENSBTAG00000013412	0.249978253	0.121105034	-0.128873219
Q08DX5	CDK7	bta:515462; F1N2N7; Q08DX5	ENSBTAG00000011046	0.059757776	-0.053079559	-0.112837336
A0AAA9TXA0	C17H12orf43	A0AAA9TXA0	ENSBTAG00000021796	0.195510412	0.119663096	-0.075847316
A0A3Q1MS44	DDX51	A0A3Q1MS44	ENSBTAG00000017219	-0.397394538	-0.449330677	-0.051936138
F6PY02	RDH13	F6PY02	ENSBTAG00000030393	-0.001444862	-0.074076591	-0.072631728
E1BPF3	BCL9	E1BPF3	ENSBTAG00000002422	-0.074962058	-0.110918935	-0.035956878
F1MB54	EXOSC9	F1MB54	ENSBTAG00000004942	0.072408496	0.060832144	-0.011576351
Q5E9H5	BCS1L	bta:539713; Q5E9H5	ENSBTAG00000003813	-0.122245906	-0.063604238	0.058641668
A0AAA9T9U8	RFTN1	A0AAA9T9U8	ENSBTAG00000030593	-0.333841416	-0.633217669	-0.299376253
A0A3Q1M9C8	AKAP8	A0A3Q1M9C8	ENSBTAG00000001807	-0.060958881	-0.036004953	0.024953927
A0A3Q1NEI6	EIF4E	A0A3Q1NEI6	ENSBTAG00000009522	0.077092868	-0.015925546	-0.093018414
E1BH93	MAST4	E1BH93	ENSBTAG00000025964	-0.056232611	-0.214475808	-0.158243196
A0AAA9RZE5	GYS1	A0AAA9RZE5	ENSBTAG00000039958	-0.135577546	-0.093843787	0.041733758
Q1ZYR0	CLN5	bta:529186; Q1ZYR0	ENSBTAG00000018846	0.006572654	0.173580172	0.167007518
E1BIJ3	CASKIN2	E1BIJ3	ENSBTAG00000007220	0	0.117426883	0.117426883
Q5E936	TXNDC12	bta:506991; Q5E936	ENSBTAG00000011024	-0.058370563	0.129362744	0.187733306
A0AAA9TNM4	CPNE8	A0AAA9TNM4	ENSBTAG00000020914	-0.788068049	-0.635861774	0.152206276
A0A3Q1MT43	ARHGAP42	A0A3Q1MT43; bta:508009	ENSBTAG00000004423	-0.116266308	-0.168505724	-0.052239416
A0AAF6Z1E4	TOX4	A0AAF6Z1E4	ENSBTAG00000015146	-0.047627268	0.050103314	0.097730583
O46375	TTR	bta:280948; O46375; Q3SZ91	ENSBTAG00000010991	-0.508114422	0.100217638	0.60833206
A0A3Q1LJP7	TMEM245	A0A3Q1LJP7; bta:521172	ENSBTAG00000038794	0.155014535	0.022787365	-0.13222717
A0A3Q1MCK1	HPS5	A0A3Q1MCK1; bta:510165	ENSBTAG00000005147	-0.170463421	-0.097998438	0.072464983
Q3SYS0	MRPL24	bta:532203; Q3SYS0	ENSBTAG00000001520	-0.203267077	-0.203267077	0
A0AAA9SFF8	RPL22	A0AAA9SFF8	ENSBTAG00000014423	0.057773287	-0.004427712	-0.062200999
A0AAA9SY74	COMMD9	A0AAA9SY74	ENSBTAG00000019045	0.141814295	0.532151353	0.390337059
Q08DW1	RAB9A	bta:511776; Q08DW1	ENSBTAG00000010923	0.110873217	0.130502024	0.019628807
A4IFA7	CBR4	A4IFA7; bta:533020	ENSBTAG00000022013	-0.254062065	0.073537256	0.327599321
A0A3Q1MSQ6	ANKS1A	A0A3Q1MSQ6	ENSBTAG00000016890	0.099535674	0.155820847	0.056285173
B0JYN1	CTSL2	B0JYN1		-0.121663741	-0.172937618	-0.051273877
A0AAF6DM48	MFN2	A0AAF6DM48	ENSBTAG00000005314	-0.192113535	-0.245526055	-0.05341252
F6RJ80	EML3	F6RJ80	ENSBTAG00000011872	-0.258918297	-0.290979507	-0.032061209
A0AAF6Z7A4	EIF1AD	A0AAF6Z7A4	ENSBTAG00000020186	0.212364961	-0.08246216	-0.294827121
A0AAA9SCT8	THOC7	A0AAA9SCT8	ENSBTAG00000032872	-0.020221394	-0.115734681	-0.095513287
A0A3S5ZP75	PPCS	A0A3S5ZP75	ENSBTAG00000012982	0.083503817	0.213208244	0.129704426
A0A452DIN1	GADD45GIP1	A0A452DIN1	ENSBTAG00000015117	-0.077852145	-0.196904817	-0.119052672
Q3MHZ7	PIGK	bta:508700; Q3MHZ7	ENSBTAG00000031012	0.103298625	0.002971567	-0.100327059
F1N1Z3	CRTC3	F1N1Z3	ENSBTAG00000010915	0.758558125	0.730079209	-0.028478916
Q2T9Y6	GCLM	bta:525659; Q2T9Y6	ENSBTAG00000007842	0.193603998	0.177408935	-0.016195063
A0A3Q1MGX6	AKTIP	A0A3Q1MGX6; bta:540462	ENSBTAG00000016250	-0.153323034	0.014766711	0.168089745
P01096	ATP5IF1	bta:327699; P01096; Q2M2T4	ENSBTAG00000006342	0.095890807	0	-0.095890807
Q6IE76	FDFT1	bta:281767; F1MW61; Q6IE76	ENSBTAG00000012432	-0.123902558	-0.325292329	-0.201389771
A0AAA9SST7	ATG2B	A0AAA9SST7	ENSBTAG00000012833	0.089786022	-0.129128627	-0.218914649
Q0IIM2	ARL6	bta:519014; Q0IIM2	ENSBTAG00000010091	-0.007370106	0.099413146	0.106783252
F1MYH0	ZNHIT6	F1MYH0	ENSBTAG00000021051	-0.389042291	-0.352048083	0.036994207
F6Q3R4	QTRT2	F6Q3R4	ENSBTAG00000004977	-0.26846318	-0.264389685	0.004073495
A0AAA9TCY6	ADPRH	A0AAA9TCY6	ENSBTAG00000009391	0.027985774	0.185376139	0.157390365
A1A4M0	TIMM13	A1A4M0; bta:516325; F6RJY8		-0.031118158	-0.004204068	0.02691409
F1MNG1	CYP1B1	F1MNG1	ENSBTAG00000010531	-0.132857159	-0.201739634	-0.068882476
A0A452DIV1	MRPL13	A0A452DIV1	ENSBTAG00000006767	-0.139162748	-0.170403428	-0.03124068
E1BM96	MAGI3	E1BM96	ENSBTAG00000019611	-0.087232838	-0.241119629	-0.153886791
F1MH25	AMZ2	F1MH25	ENSBTAG00000009345	-0.046350316	-0.192086651	-0.145736335
Q5E982	DPH5	bta:508904; Q2HJ50; Q5E982	ENSBTAG00000019029	-0.159526052	-0.121629444	0.037896608
A0A3Q1LYZ4	SFSWAP	A0A3Q1LYZ4	ENSBTAG00000000439	0.175824913	-0.022985537	-0.198810451
F1N4A6	EMSY	F1N4A6	ENSBTAG00000010826	0.205132746	0.259482842	0.054350097
A0A3Q1M442	TUBB1	A0A3Q1M442; bta:541271	ENSBTAG00000018785	0.154655107	-0.003987184	-0.158642291
Q1RMS1	TCIRG1	bta:513684; F1MGS2; Q1RMS1	ENSBTAG00000000292	0.071216799	0.061255688	-0.00996111
G3MWU7	TPRN	G3MWU7	ENSBTAG00000047715	-0.202126573	-0.152785254	0.049341319
E1BEZ8	PTPN14	E1BEZ8	ENSBTAG00000021553	0.045867009	-0.129219698	-0.175086707
E1B789	EDEM3	E1B789	ENSBTAG00000019264	-0.247092862	-0.263034406	-0.015941544
E1B9Q8	ZNF609	bta:510374; E1B9Q8	ENSBTAG00000015808	0.058950995	0.007145607	-0.051805388
Q0P5A1	DCTN3	bta:514327; Q0P5A1		-0.052669069	-0.144461544	-0.091792476
A0A3Q1NJJ1	ARK2N	A0A3Q1NJJ1	ENSBTAG00000032964	0.234320688	-0.108027622	-0.34234831
Q1RMT8	IRAK4	bta:533692; Q1RMT8	ENSBTAG00000021105	-0.089068674	-0.221614478	-0.132545804
A0AAF7AH20	CRYAB	A0AAF7AH20	ENSBTAG00000000434	0.863052321	0.966251209	0.103198888
F1N0D6	JAK1	F1N0D6	ENSBTAG00000003147	0.117580929	-0.094239328	-0.211820257
A0A3Q1MRR5	LIMCH1	A0A3Q1MRR5	ENSBTAG00000010677	0.101243832	0.041353963	-0.059889869
A2VDY0	CTHRC1	A2VDY0; bta:538634	ENSBTAG00000032591	-0.167741532	-0.522084542	-0.35434301
Q0VD51	RNF13	bta:539035; Q0VD51	ENSBTAG00000011147	0.449483645	0.484404907	0.034921262
E1BL26	RNF123	bta:514160; E1BL26	ENSBTAG00000011588	-0.04491845	-0.105977018	-0.061058568
F6PZI3	SLC29A1	bta:510932; F6PZI3	ENSBTAG00000015131	-0.147898695	-0.158869813	-0.010971118
A0AAA9TSF3	MCRIP1	A0AAA9TSF3	ENSBTAG00000019812	-0.265474827	-0.366639744	-0.101164917

Accession	Gene_Symbol	Gene_ID	Ensembl_Gene ID	log2(ratio(CM12H/CM0H))	log2(ratio(CM12R/CM0H))	log2(ratio(CM12H/CM12R))
A0AAF6YRV5	SLC1A4	A0AAF6YRV5	ENSBTAG00000007763	0.052151282	0.243728883	0.191577601
E1BC57	ROR2	E1BC57	ENSBTAG00000005092	0.007541549	0.120354723	0.112813174
Q6TJY3	RPS6KB1	bta:404181; Q6TJY3		-0.130681017	-0.165657155	-0.034976138
A0AAA9T0P8	CEP97	A0AAA9T0P8	ENSBTAG00000013464	0.216146512	-0.11585336	-0.331999872
A0A3Q1NJQ0	FAR1	A0A3Q1NJQ0	ENSBTAG00000009061	-0.258669213	-0.210098654	0.048570559
A0AAA9SNI5	BSDC1	A0AAA9SNI5	ENSBTAG00000020655	0.285531406	0.427162815	0.141631409
Q3ZBH8	RPS20	bta:513222; Q3ZBH8	ENSBTAG00000019147	-0.050009425	-0.036321514	0.013687911
P82917	MRPS18C	bta:613561; P82917; Q2KI03	ENSBTAG00000018155	-0.076855923	-0.128114043	-0.05125812
A0A3Q1M1E9	GPD1L	A0A3Q1M1E9	ENSBTAG00000009826	0.135919015	0.097951165	-0.03796785
Q5E9K0	PSMB2	bta:516919; Q5E9K0	ENSBTAG00000002377	0.11627827	0.119877511	0.003599241
Q08DC8	NUMBL	bta:510973; Q08DC8		0.0935928	0.067992977	-0.025599823
A0A3Q1LWZ9	ERCC2	A0A3Q1LWZ9	ENSBTAG00000002072	0.073924728	-0.024478976	-0.098403704
A0AAF6YQ96	GABARAPL2	A0AAF6YQ96	ENSBTAG00000006550	0.322716956	-0.173051816	-0.495768771
A0AAA9TWW3	MRPL48	A0AAA9TWW3	ENSBTAG00000008347	-0.134143308	-0.148413405	-0.014270097
A0A3Q1LN51	ATF1	A0A3Q1LN51	ENSBTAG00000018131	0.25956863	-0.003198882	-0.262767512
F1MZ00	SNRPD3	F1MZ00	ENSBTAG00000021262	-0.048812249	-0.00141233	0.047399919
A0A3Q1LXK3	TTC17	A0A3Q1LXK3	ENSBTAG00000031299	0.485642171	-0.172754371	-0.658396542
A0A3Q1M4U3	TRAPPC10	A0A3Q1M4U3	ENSBTAG00000007100	-0.063143983	-0.059659211	0.003484772
A6QLV3	SHOC2	A6QLV3; bta:511417	ENSBTAG00000007709	0.033637597	0.030743523	-0.002894073
Q2NKS3	PSMG3	bta:510741; Q2NKS3	ENSBTAG00000000050	0.110372971	0.214291923	0.103918951
A0AAA9U299	TMEM30A	A0AAA9U299	ENSBTAG00000005100	0.112800602	0.008664861	-0.104135741
E1BHL0	CNOT11	bta:506268; E1BHL0	ENSBTAG00000008267	0.049501961	0.150372846	0.100870884
A0A3Q1LWG3	RPS6KC1	A0A3Q1LWG3	ENSBTAG00000001181	-0.051609311	0.106087258	0.15769657
A0AAA9TXN0	MAPK8	A0AAA9TXN0	ENSBTAG00000007876	-0.296208267	-0.107725479	0.188482788
A0A3Q1MCA2	DMWD	A0A3Q1MCA2; bta:790091	ENSBTAG00000021417	-0.294037698	-0.032945943	0.261091756
A6H716	BRAP	A6H716; bta:518844	ENSBTAG00000001168	-0.083462722	-0.204841961	-0.121379239
A2VDW3	XRCC4	A2VDW3; bta:613590; F1N695	ENSBTAG00000024275	-0.032573991	-0.095706355	-0.063132364
F1MCG4	RALGAPB	F1MCG4	ENSBTAG00000003040	0.110817991	0.002876761	-0.10794123
F1MIX0	ABRAXAS2	F1MIX0	ENSBTAG00000023840	-0.082382328	-0.287396973	-0.205014645
A0AAA9TRK4	PIN4	A0AAA9TRK4	ENSBTAG00000047376	0.368672995	0.361926163	-0.006746832
Q8HXK9	PYCARD	bta:282846; Q8HXK9		0.096605932	0.070893855	-0.025712077
F1MZE4	SPAG7	F1MZE4	ENSBTAG00000008590	-0.154498471	-0.103973722	0.05052475
P57709	ATP2C1	bta:327663; P57709	ENSBTAG00000011626	0.051747775	0.088647357	0.036899583
Q2TBQ3	GAMT	bta:515270; Q2TBQ3	ENSBTAG00000004112	-0.056955885	-0.091249062	-0.034293178
A0AAA9RV48	UTP4	A0AAA9RV48	ENSBTAG00000006347	-0.381288708	-0.395254887	-0.013966179
Q2NL01	GPX8	bta:511575; Q2NL01	ENSBTAG00000021960	0.019279062	-0.331665106	-0.350944169
E1BG08	AIDA	bta:508353; E1BG08	ENSBTAG00000007593	0.027982413	0.04580369	0.017821277
F1N3I6	WWC1	F1N3I6	ENSBTAG00000013880	0.078905197	-0.240441058	-0.319346255
A0AAA9SRE4	PPIC	A0AAA9SRE4	ENSBTAG00000001568	0.050208741	-0.059005739	-0.109214479
A0AAA9TKA1	SNX12	A0AAA9TKA1	ENSBTAG00000007871	0.087823921	0.105050335	0.017226414
E1B9S8	CBFB	E1B9S8	ENSBTAG00000016103	0.17519386	-0.056083547	-0.231277407
A0A3Q1ND53	HAGH	A0A3Q1ND53	ENSBTAG00000019998	0.072669068	0.082757885	0.010088818
E1BIR9	SARM1	E1BIR9	ENSBTAG00000002816	-0.135303734	-0.09399026	0.041313474
A2VDZ0	PPP2R5A	A2VDZ0; bta:533788	ENSBTAG00000000754	0.252257688	0.180043398	-0.07221429
Q2KID4	DNAL1	bta:538164; Q2KID4	ENSBTAG00000015930	0.165134301	0.075735216	-0.089399085
A0AAA9S3M6	RARS2	A0AAA9S3M6	ENSBTAG00000016967	-0.197977163	-0.105236216	0.092740947
A0AAA9SL62	GDAP2	A0AAA9SL62	ENSBTAG00000016387	0.00433459	0.180657363	0.176322773
A0AAA9TIV3	BUD31	A0AAA9TIV3	ENSBTAG00000020439	0.150714397	-0.136502	-0.287216397
A5PJC3	NARS2	A5PJC3; bta:504824; F6QRZ3		-0.161776253	-0.104745308	0.057030945
Q3MQ24	ATG5	bta:532686; Q3MQ24; Q3SZN7	ENSBTAG00000005400	-0.042749647	-0.024273044	0.018476603
A0AAA9SFQ4	LRCH4	A0AAA9SFQ4	ENSBTAG00000003160	-0.134776679	-0.07468481	0.06009187
A0AAA9S5S6	EIPR1	A0AAA9S5S6	ENSBTAG00000018387	-0.033390768	-0.121909626	-0.088518858
Q5EA98	MFAP1	bta:510905; Q5EA98	ENSBTAG00000017213	0.063741373	0.006375154	-0.057366219
Q3T093	NECAP1	A5PKA6; bta:504449; Q3T093	ENSBTAG00000020237	0.193250786	0.171898225	-0.021352561
Q58D63	MOB3A	bta:505007; Q08E56; Q58D63		-0.077591124	-0.264219858	-0.186628733
E1BA19				0.327956677	-0.149926928	-0.477883605
E1BDK3	NAA30	E1BDK3	ENSBTAG00000012998	0	-0.119208233	-0.119208233
A0A3Q1LL79	NPTN	A0A3Q1LL79	ENSBTAG00000008218	0.147643554	0.160219184	0.01257563
F6QEM8	GSTM5	F6QEM8	ENSBTAG00000037673	0.033503858	0.16591784	0.132413982
F1MMX8	MED8	F1MMX8	ENSBTAG00000016899	-0.057631137	-0.020818472	0.036812666
F1MYW0	NT5DC3	bta:520927; F1MYW0	ENSBTAG00000006013	-0.257674891	-0.126944596	0.130730295
P61285	DYNLL1	bta:101902172; bta:404151; bta:784058; P61285; Q6B859	ENSBTAG00000024605	-0.002923395	0.152295107	0.155218503
A0AAF7A6E5	PQBP1	A0AAF7A6E5	ENSBTAG00000018498	0.092729298	0.152763007	0.060033709
A0AAF7ADA8	BCAT2	A0AAF7ADA8	ENSBTAG00000009172	-0.193514957	-0.060606765	0.132908193
A0A3Q1MMC0	EFNB1	A0A3Q1MMC0	ENSBTAG00000015801	-0.218946149	-0.092160859	0.126785291
A0AAA9U136	PRKD2	A0AAA9U136	ENSBTAG00000015599	-0.14014401	-0.021595148	0.118548862
A0AAA9SV20	H1-0	A0AAA9SV20	ENSBTAG00000067495	-0.224947221	-0.110296368	0.114650853
Q3SWX1	CNPY4	bta:505637; Q3SWX1		-0.00138388	-0.140827069	-0.139443189
A0A3Q1M294	KMO	A0A3Q1M294; bta:515996	ENSBTAG00000014439	-0.173718575	-0.004046833	0.169671742
A4IFA8				-0.316497818	-0.097982079	0.218515739
F1MHW3	TRIR	F1MHW3	ENSBTAG00000011825	0.212668536	-0.063171326	-0.275839862
P82669	MRPS25	bta:533011; P82669; Q32P88	ENSBTAG00000013723	-0.072921393	-0.145181828	-0.072260435
A0A452DJ65	TK1	A0A452DJ65	ENSBTAG00000007121	0.056989979	-0.154647815	-0.211637793
Q2KHY9	PTDSS1	bta:509819; F1MKI4; Q2KHY9	ENSBTAG00000013901	0.085260618	0.054267052	-0.030993567
A0AAA9TH89	CACNA2D1	A0AAA9TH89	ENSBTAG00000020569	-0.269569842	-0.299351302	-0.02978146
A0AAF6ZE24	MTFR1L	A0AAF6ZE24	ENSBTAG00000021781	0.149199021	0.078739521	-0.0704595
F1MWH8	ABL2	F1MWH8	ENSBTAG00000015026	0.043887644	-0.025286442	-0.069174086
Q59A28	CRYZL1	bta:506426; Q59A28		-0.61356942	-0.248006197	0.365563224
E1BD42	NUP37	E1BD42	ENSBTAG00000000022	-0.084359819	-0.039491051	0.044868768
A2VDV0	NUDT4	A2VDV0; bta:614183	ENSBTAG00000007447	-0.147891435	-0.156411312	-0.008519877
A0AAA9RZF6				0.135504869	0.09356704	-0.04193783
A5D7R0	FAS	A5D7R0	ENSBTAG00000010785	-0.308330491	-0.312396333	-0.004065841
A7Z088	YEATS2	A7Z088; bta:514192; F1N223	ENSBTAG00000014451	-0.034302497	-0.079434467	-0.045131971
F1MDA8	DCLK1	F1MDA8	ENSBTAG00000038495	0.609571968	0.58185659	-0.027715378
Q08DK4	SLC25A22	bta:504371; Q08DK4	ENSBTAG00000001229	-0.194516521	-0.127198049	0.067318471
Q3SZD1	PEX19	bta:521522; Q3SZD1	ENSBTAG00000007537	-0.526473551	-0.561878888	-0.035405336
Q17QG8	H2AFX	bta:531733; Q17QG8	ENSBTAG00000067452	-0.321928095	0.016950069	0.338878164
Q3ZCD7	TECR	bta:614105; Q3ZCD7	ENSBTAG00000012632	-0.013776503	-0.038597675	-0.024821172
A6QQ09	LOC100138230	A6QQ09; bta:100138230		-0.146929694	-0.407229132	-0.260299438
F1MJN1	ZNF318	F1MJN1	ENSBTAG00000010217	0.148257312	0.020990897	-0.127266415

Accession	Gene_Symbol	Gene_ID	Ensembl_Gene ID	log2(ratio(CM12H/CM0H))	log2(ratio(CM12R/CM0H))	log2(ratio(CM12H/CM12R))
A0AAF6Z211	NOL11	A0AAF6Z211	ENSBTAG00000015951	-0.386373741	-0.22259348	0.163780261
A0AAA9TJ50	FOXK2	A0AAA9TJ50	ENSBTAG00000003687	-0.153385647	-0.08056128	0.072824368
A0AAA9TY59	GLT8D1	A0AAA9TY59	ENSBTAG00000014550	-0.073857592	0.084423061	0.158280653
A0A3Q1MC91	SAMD4B	A0A3Q1MC91	ENSBTAG00000009785	-0.021123292	-0.005602706	0.015520585
G3N0L9				-0.357552005	-0.152282388	0.205269617
G3X6X2	NT5C	G3X6X2	ENSBTAG00000011527	-0.341416524	-0.438378254	-0.09696173
E1BFG7	PHC2	E1BFG7	ENSBTAG00000004603	0.202188851	0.092045077	-0.110143773
F1MKY4	ZFYVE9	F1MKY4; G3MXR1	ENSBTAG00000014289	0.171153499	0.103638276	-0.067515223
A0AAA9TKC2	PLA2G15	A0AAA9TKC2	ENSBTAG00000007512	0.141191028	0.166585655	0.025394628
E1BG57				-0.00874742	-0.032761295	-0.024013875
A0A3Q1M7U1	KLHL11	A0A3Q1M7U1; bta:538695	ENSBTAG00000050549	-0.174961158	-0.318041173	-0.143080014
A0AAF6ZB27	IAH1	A0AAF6ZB27	ENSBTAG00000001140	-0.214319121	-0.120241435	0.094077686
A6QNX2	DPP7	A6QNX2; bta:100125760; H9GW42		0.088024748	0.086525844	-0.001498904
A0AAA9S8H4	ADI1	A0AAA9S8H4	ENSBTAG00000005403	-0.152808843	0.099439007	0.252247851
Q3SZ11	RPA3	bta:512754; Q3SZ11	ENSBTAG00000013168	0.083466823	0.345552399	0.262085576
F6PU96	CHSY1	bta:281690; F6PU96	ENSBTAG00000007357	-0.241560666	-0.103273565	0.1382871
A0AAF6DMC7	RXRB	A0AAF6DMC7	ENSBTAG00000000602	0.163611228	0.01098305	-0.152628178
A0AAA9RVB5	ARFIP2	A0AAA9RVB5	ENSBTAG00000003566	0.095647127	0.016164819	-0.079482308
A0AAF7APV1	AMDHD2	A0AAF7APV1	ENSBTAG00000001022	-0.211372286	-0.267549887	-0.056177601
E1BKM6	TAOK2	bta:533897; E1BKM6	ENSBTAG00000017422	-0.271225898	-0.419828674	-0.148602776
A4FUB5	COMMD10	A4FUB5; bta:538706	ENSBTAG00000019071	0.142929026	0.064463593	-0.078465434
A0AAA9TSS4	DDX28	A0AAA9TSS4	ENSBTAG00000004290	-0.898750207	-0.739937161	0.158813046
A0A3Q1LYP9	TMCC3	A0A3Q1LYP9	ENSBTAG00000014375	0.533218926	0.666664233	0.133445307
A0A3Q1M7M3	MRPS23	A0A3Q1M7M3	ENSBTAG00000012366	-0.138565587	-0.202282807	-0.06371722
E1BKZ0	PCNT	E1BKZ0	ENSBTAG00000030670	0.051555188	-0.146182913	-0.1977381
F1MBJ5	SMG9	F1MBJ5	ENSBTAG00000011815	-0.461808513	-0.233199176	0.228609336
F1N5R4	COG8	F1N5R4	ENSBTAG00000001665	-0.096898205	-0.023956741	0.072941464
A4IFF2	WDR46	A4IFF2		-0.171428592	-0.118262882	0.05316571
A0A3Q1LR40	LIPG	A0A3Q1LR40	ENSBTAG00000048916	-0.034281135	-0.293015403	-0.258734268
F1MSD6	TRAPPC5	F1MSD6	ENSBTAG00000004268	0.005624549	-0.099133192	-0.104757741
Q05B63	TRMT11	bta:514867; Q05B63		-0.295251926	-0.365778922	-0.070526996
E1B9H1	TINAGL1	E1B9H1	ENSBTAG00000013235	0.154747096	0.03991901	-0.114828086
A0AAA9S4A3	MXRA5	A0AAA9S4A3	ENSBTAG00000022150	-0.22446675	-0.312189092	-0.087722342
Q3ZBI6	FHL3	bta:504795; Q3ZBI6	ENSBTAG00000007300	0.022764891	0.084929871	0.06216498
Q02368	NDUFB7	bta:338065; Q02368; Q3T0A1	ENSBTAG00000012634	0.015689374	0.045176139	0.029486765
A0A452DJJ7	MRPL28	A0A452DJJ7	ENSBTAG00000016581	-0.185555653	-0.068361555	0.117194099
A0AAA9SGW9	ATXN3	A0AAA9SGW9	ENSBTAG00000005786	-0.130703692	0.003781641	0.134485332
A0A3Q1MCA3	CBX8	A0A3Q1MCA3	ENSBTAG00000009107	0.316944687	0.195944886	-0.120999801
A0AAA9T1E3	IGFBP2	A0AAA9T1E3	ENSBTAG00000005596	0.036095799	-0.030419396	-0.066515194
P35604	COPZ1	bta:281707; P35604; Q5EA44		0.415037499	0.564263508	0.149226009
E1BNH5	MTMR12	E1BNH5	ENSBTAG00000012278	-0.177451006	-0.078246643	0.099204362
Q3SX43	RRAGA	bta:618207; Q3SX43	ENSBTAG00000033543	-0.111661823	-0.229333647	-0.117671824
A4FUD7	RAB33B	A4FUD7; bta:519982	ENSBTAG00000003208	0.029486765	-0.041736732	-0.071223497
F1N7I6	GCA	bta:507139; F1N7I6	ENSBTAG00000018446	0.178394592	0.394546973	0.216152381
A0AAA9SHJ5	AP2S1	A0AAA9SHJ5	ENSBTAG00000010584	-0.123475648	0.042863968	0.166339616
A0AAA9SL48				0.001540518	-0.071083098	-0.072623616
Q32KX0	ISOC2	bta:516212; Q32KX0		0.036525876	0.078002512	0.041476636
A2VDP5	ENPP4	A2VDP5; bta:538583	ENSBTAG00000003499	0.222392421	0.076512894	-0.145879527
A5PJS8	INTS8	A5PJS8; bta:100124522; F1MI91	ENSBTAG00000004902	-0.240088474	-0.078725486	0.161362988
A0A452DID6	DHRS11	A0A452DID6	ENSBTAG00000010297	-0.185014812	-0.157603695	0.027411117
A0A452DIU4	CLEC3B	A0A452DIU4	ENSBTAG00000018331	0.043529868	0.162938571	0.119408703
A0AAA9T613	LXN	A0AAA9T613	ENSBTAG00000009336	0.009358055	0.038597675	0.02923962
A0AAA9TPF0	IKBKG	A0AAA9TPF0	ENSBTAG00000006268	0.034204899	0.013779427	-0.020425472
F1MC26	TBC1D2B	F1MC26	ENSBTAG00000012022	0.066996564	0.090276225	0.023279661
A0AAA9SD33	MGA	A0AAA9SD33	ENSBTAG00000015138	-0.06276361	-0.32946833	-0.26670472
E1BCW0	HGFAC	E1BCW0	ENSBTAG00000017335	0.084236537	0.38038343	0.296146893
A0AAA9TXU3	GSTZ1	A0AAA9TXU3	ENSBTAG00000002706	-0.032191286	-0.13331321	-0.101121924
Q3T0D2	SCAMP1	bta:535352; Q3T0D2	ENSBTAG00000007371	0.228912521	0.292063454	0.063150933
B2XBK5	CDYL	B2XBK5		-0.18781516	-0.152755869	0.035059291
G3N258	TNRC6A	G3N258	ENSBTAG00000017999	0.103445649	-0.042759247	-0.146204896
A0AAA9SK06				-0.001389211	-0.0083554	-0.006966189
A0AAA9SAM7	DENND5A	A0AAA9SAM7	ENSBTAG00000006322	-0.379206507	-0.350873601	0.028332906
A0AAA9T4U1	MBD3	A0AAA9T4U1	ENSBTAG00000038409	0.015154499	-0.125669383	-0.140823882
G3MYW2	NKRF	G3MYW2	ENSBTAG00000045744	-0.187869656	0.00290866	0.190778317
F1N420	NPM3	bta:504950; F1N420	ENSBTAG00000016335	-0.511121096	-0.574179502	-0.063058407
Q2KJ63	KLKB1	bta:533547; Q2KJ63	ENSBTAG00000009501	0	0.241685807	0.241685807
P48305	NDUFB4	bta:327706; P48305; Q54A31	ENSBTAG00000015892	-0.048867118	0.007373245	0.056240362
Q9BE40	MYH1	bta:281337; Q9BE40	ENSBTAG00000018204	-0.121660607	1.913903762	2.035564369
F1N059	MRPS18B	F1N059	ENSBTAG00000006936	-0.202076862	-0.204991398	-0.002914536
A0AAA9TYA9	CERS2	A0AAA9TYA9	ENSBTAG00000000099	-0.003742402	-0.023867506	-0.020125105
A0A452DKN5	RGN	A0A452DKN5	ENSBTAG00000046155	-0.234266522	-0.088237329	0.146029192
A0AAA9TDI5	SIPA1	A0AAA9TDI5	ENSBTAG00000013884	-0.045005146	-0.047696742	-0.002691596
Q29RR1	LRRC41	bta:337889; Q29RR1		-0.373936934	-0.354806996	0.019129938
A0A3Q1MZU1	CTDSPL2	A0A3Q1MZU1; bta:540232	ENSBTAG00000015385	-0.208977331	-0.32017121	-0.111193879
A6QLB1	RAB27A	A6QLB1; bta:618035	ENSBTAG00000012408	-0.023104151	0.092472594	0.115576745
A1L552	INTS5	A1L552; bta:514150	ENSBTAG00000010455	-0.543361596	-0.37183981	0.171521786
Q3ZBK6	LSM4	bta:613634; Q3ZBK6	ENSBTAG00000008578	0.148981741	0.16791147	0.018929729
A0A3Q1MTG5	SNX16	A0A3Q1MTG5	ENSBTAG00000019639	0.069262662	-0.398489654	-0.467752316
A0AAA9TBE0	LRSAM1	A0AAA9TBE0	ENSBTAG00000012844	-0.28286742	-0.221062135	0.061805284
A0A3Q1M4E5	FCSK	A0A3Q1M4E5	ENSBTAG00000012985	-0.181017076	0.016582884	0.19759996
E1BEB4	FBLN2	bta:511854; E1BEB4	ENSBTAG00000004014	0.439942841	0.139025239	-0.300917602
A0A3Q1LPP6	MLKL	A0A3Q1LPP6	ENSBTAG00000012216	-0.085220133	-0.078609835	0.006610298
Q2YDF6	MRPS35	bta:513438; P82672; Q2YDF6	ENSBTAG00000006369	-0.104684899	-0.292346569	-0.18766167
A0A452DIK3	NDUFS8	A0A452DIK3	ENSBTAG00000012072	-0.106915204	-0.072967872	0.033947332
Q08DS9	DHX40	bta:533484; Q08DS9		0.72312494	0.123263563	-0.599861377
Q1JQE2	SLC7A6OS	bta:512543; Q1JQE2	ENSBTAG00000009636	0.39864711	-0.166563106	-0.565210216
Q32LE2	GKAP1	bta:613960; Q32LE2	ENSBTAG00000023523	0.110975825	-0.096008692	-0.206984517
A0A3Q1MKH2	AKAP17A	A0A3Q1MKH2	ENSBTAG00000054242	0.150188279	0.260283795	0.110095516
A0A3Q1ML38	LENG8	A0A3Q1ML38	ENSBTAG00000011689	-0.066709342	-0.014241923	0.05246742
Q1RMS6	INTS7	bta:534964; Q1RMS6	ENSBTAG00000018548	-0.225401171	-0.142019005	0.083382166
E1BIS5	RCC1L	bta:513273; E1BIS5	ENSBTAG00000011753	-0.135070044	0.001431955	0.136502

Accession	Gene_Symbol	Gene_ID	Ensembl_Gene ID	log2(ratio(CM12H/CM0H))	log2(ratio(CM12R/CM0H))	log2(ratio(CM12H/CM12R))
Q32PI6	MRPL4	bta:507154; Q32PI6	ENSBTAG00000001419	-0.007131478	-0.031645627	-0.024514149
A0AAA9SFS1				0.054619585	0.30981628	0.255196695
F6QH10	SMOC1	F6QH10	ENSBTAG00000030599	-0.160121826	-0.447458977	-0.287337151
Q3MHX0	SUOX	bta:509837; F6RUR3; Q3MHX0	ENSBTAG00000006160	0.039929533	0.169175043	0.12924551
A0A3Q1N5C0	USP3	A0A3Q1N5C0	ENSBTAG00000008805	-0.231221181	-0.255373213	-0.024152032
A0A3Q1LZG8	PKD2	A0A3Q1LZG8	ENSBTAG00000020031	0.135011104	0.003040454	-0.13197065
A0AAA9TP18	MRPS16	A0AAA9TP18	ENSBTAG00000000950	-0.177351044	-0.118402657	0.058948387
A0AAA9SCL2	POLR3E	A0AAA9SCL2	ENSBTAG00000018044	0.076424441	0.250853645	0.174429204
Q05B45	TMEM120A	bta:520173; Q05B45		-0.040237131	0.086628962	0.126866093
A4FV59	PHF5A	A4FV59; bta:507180	ENSBTAG00000006423	0.174961158	0.161928594	-0.013032564
Q02380	NDUFB5	bta:338061; Q02380; Q3SZV4; Q56K08	ENSBTAG00000002463	-0.040641984	-0.144198176	-0.103556192
Q3MHR2	DCK	bta:530642; Q3MHR2	ENSBTAG00000012397	0.505978701	-0.049124511	-0.555103212
A3KMY2	DKK2	A3KMY2; bta:541161	ENSBTAG00000012969	-0.626541604	-0.775735164	-0.14919356
F1MTJ9	LSS	F1MTJ9	ENSBTAG00000018936	-0.377773231	-0.234754922	0.143018309
A0AAF6YJP2				0.288441013	0.016022018	-0.272418995
F1MRW6	PLEKHM2	F1MRW6	ENSBTAG00000018722	0.079652249	0.078002512	-0.001649737
Q2TBQ1	F13B	bta:511483; Q2TBQ1	ENSBTAG00000000070	-0.201897391	-0.223271042	-0.021373651
Q3T0N1	MVB12A	bta:507199; Q3T0N1	ENSBTAG00000003914	-0.067337991	-0.065697633	0.001640358
P32871	PIK3CA	bta:282306; P32871	ENSBTAG00000009232	0.085590869	0.109906529	0.024315661
Q2YDL5	FKBP11	bta:506043; Q2YDL5		0.02642603	-0.14412257	-0.1705486
F1MW69	NELFCD	F1MW69	ENSBTAG00000018783	-0.273607426	-0.073797752	0.199809674
A4FUG9	BXDC5	A4FUG9; bta:513081		-0.405225777	-0.152003093	0.253222683
Q17QZ4	TFDP1	bta:534579; Q17QZ4	ENSBTAG00000019645	0.046589559	-0.147898695	-0.194488254
Q3T132	LAMTOR2	bta:616375; Q3T132	ENSBTAG00000011754	0.017260765	0.217215157	0.199954392
A0A140T8A2	DPH2	A0A140T8A2; bta:768224	ENSBTAG00000018888	-0.182612519	-0.16461609	0.017996429
A0AAA9TH24	MRPL10	A0AAA9TH24	ENSBTAG00000001832	-0.089435084	-0.17140393	-0.081968847
E1BN13	PDCD2L	bta:507032; E1BN13	ENSBTAG00000002571	-0.04148196	-0.264312824	-0.222830864
A0AAA9SDT0	STX18	A0AAA9SDT0	ENSBTAG00000005712	0.097955215	-0.027911138	-0.125866354
Q58CS7	CHMP3	bta:507722; Q29RQ4; Q58CS7	ENSBTAG00000013589	0.184906393	0.006011238	-0.178895155
Q32L99	PTGR2	bta:506263; Q32L99	ENSBTAG00000003747	0.276209795	0.216234134	-0.059975661
A0AAF6ZG02	GCDH	A0AAF6ZG02	ENSBTAG00000016211	-0.052993088	-0.064488726	-0.011495639
A0AAA9SAB7	TMEM131	A0AAA9SAB7; bta:540861	ENSBTAG00000006744	0.137077174	-0.054147229	-0.191224403
A6QNS7	PDE4DIP	A6QNS7		-0.004283116	-0.028797264	-0.024514149
A6QQ47	VPS51	A6QQ47; bta:525567		-0.255570029	-0.107874974	0.147695054
F1MS32	APOD	F1MS32	ENSBTAG00000023600	-0.11318249	0.520623371	0.63380586
E1B9A2	CARD6	E1B9A2	ENSBTAG00000014374	0.059530245	0.007217099	-0.052313146
A0A452DJH7	MRPS10	A0A452DJH7	ENSBTAG00000015301	-0.106308902	-0.172884377	-0.066575475
A0A3Q1MTZ4	ADGRG6	A0A3Q1MTZ4	ENSBTAG00000020707	0.100194288	0.029343962	-0.070850326
A0AAF6DLX3	MICU2	A0AAF6DLX3	ENSBTAG00000015943	-0.046764467	-0.02181811	0.024946357
Q2KJ16	PHKG2	A7E3T5; bta:512670; Q2KJ16	ENSBTAG00000003417	-0.039528364	-0.033376137	0.006152227
A7E3T6	CEP27	A7E3T6		0.005581032	0.005581032	0
F1MU23	FBXL8	F1MU23	ENSBTAG00000023632	0.039034713	0.018147347	-0.020887366
E1BB36	MGAT2	bta:101906103; E1BB36	ENSBTAG00000033891	-0.063948863	-0.076524492	-0.01257563
Q3ZCE1	STAP2	bta:505456; Q3ZCE1		-0.086156644	0	0.086156644
Q32KU8	NUDCD2	bta:532693; Q32KU8	ENSBTAG00000061380	0.009226953	0.15411732	0.144890367
G3MYT4	TPBG	G3MYT4	ENSBTAG00000039477	0.162686175	0.125936989	-0.036749186
Q5E954	DNAJA1	bta:528862; Q3SZU2; Q5E954	ENSBTAG00000016265	0.815321232	0.05332306	-0.761998172
E1BEL6	NRP2	E1BEL6	ENSBTAG00000011971	-0.017431082	-0.135541223	-0.11811014
Q2TA14	PRCP	bta:534927; Q2TA14		0.086501132	0.186503169	0.100002037
A0AAA9S954	CAMKK1	A0AAA9S954; bta:510260	ENSBTAG00000007166	0.176380024	0.217763944	0.041383921
F1N6Q0	DNAJA4	F1N6Q0	ENSBTAG00000015942	1.009823403	0.505764715	-0.504058687
E1BF57	INTS6	E1BF57	ENSBTAG00000002970	-0.203700266	0.063292153	0.266992419
A0AAA9S1Y2	CTDP1	A0AAA9S1Y2	ENSBTAG00000053484	-0.186931427	-0.114642566	0.072288861
E1B986	PRKAB2	bta:512665; E1B986	ENSBTAG00000014387	-0.12116569	-0.010726407	0.110439283
A0AAA9SG44	TBC1D22A	A0AAA9SG44	ENSBTAG00000012291	-0.214287372	-0.28845339	-0.074166019
E1BMI4	UXS1	E1BMI4	ENSBTAG00000005614	-0.215612639	-0.351306967	-0.135694327
A0AAF6ZL46	REPIN1	A0AAF6ZL46	ENSBTAG00000038241	0.047005987	0.022388232	-0.024617756
F1N429	SESTD1	bta:789054; F1N429	ENSBTAG00000001861	0.273626763	-0.142533894	-0.416160656
A0AAA9S356	TDP1	A0AAA9S356	ENSBTAG00000013138	-0.046518379	-0.058770639	-0.012252261
Q3T035	ARPC3	bta:506596; Q3T035	ENSBTAG00000005345	0.142066611	0.1155944	-0.026472211
Q05204	LAMP1	A2VE82; A5D7M2; bta:281897; Q05204; Q17QC6		-0.145631405	-0.2645088	-0.118877396
A0AAA9TH54	SMARCD1	A0AAA9TH54	ENSBTAG00000037935	0.057407376	-0.097496528	-0.154903905
A0A3Q1M9U6	MTMR10	A0A3Q1M9U6	ENSBTAG00000014422	-0.072706445	-0.036637191	0.036069255
Q3T087	RPL11	bta:512745; Q3T087		-0.107745174	-0.041027268	0.066717906
G3MZR3	SECISBP2L	G3MZR3	ENSBTAG00000046553	0.234256757	-0.011162106	-0.245418863
A0A3Q1MLH8	PTPRE	A0A3Q1MLH8	ENSBTAG00000002435	-0.257361722	-0.123860129	0.133501593
A0AAA9U2I0	NME7	A0AAA9U2I0	ENSBTAG00000002689	0.062550271	0.004352025	-0.058198247
Q2LGB5	TOLLIP	bta:539480; Q2LGB5; Q58D12		0.018881466	0.24710886	0.228227394
A0AAA9TUZ6	GMDS	A0AAA9TUZ6	ENSBTAG00000012058	-0.203973084	-0.16008544	0.043887644
F1MB07	LIG4	bta:781252; F1MB07	ENSBTAG00000015868	-0.052317194	0.014797002	0.067114196
A0A3Q1LR92	RASSF4	A0A3Q1LR92	ENSBTAG00000002669	-0.02270126	0.261650715	0.284351975
F1MWS9	PPAN	bta:522370; F1MWS9	ENSBTAG00000012745	0.224391999	-0.106556101	-0.3309481
Q3MI00	DNAJB1	bta:538426; Q3MI00	ENSBTAG00000016874	0.92189692	0.586771523	-0.335125397
A4IFV0	LDLRAP1	A4IFV0; bta:511199; F1MJ16	ENSBTAG00000001050	-0.227410496	-0.011495639	0.215914857
A0JNG7	FHIP2A	A0JNG7; bta:522276		-0.054049717	-0.088029032	-0.033979315
E1BIX7	GPSM2	bta:513654; E1BIX7	ENSBTAG00000017124	0.069393335	-0.122113522	-0.191506857
Q4ZJ65	NFIC	bta:553098; F6RWF1; Q4ZJ65	ENSBTAG00000008520	-0.019985705	-0.206355458	-0.186369753
A0AAA9TQ25	SURF4	A0AAA9TQ25	ENSBTAG00000051352	-0.010427371	0.017702002	0.028129373
E1BBA0	ARAP3	E1BBA0	ENSBTAG00000015938	0.236515533	0.048837608	-0.187677925
F6RR50	RBCK1	F6RR50	ENSBTAG00000017002	-0.16726985	0.082630887	0.249900737
A0AAA9SCN9	MBD1	A0AAA9SCN9	ENSBTAG00000003801	-0.03058832	-0.080005643	-0.049417324
A6QR44	DGCR8	A6QR44; bta:540254	ENSBTAG00000019869	0.177625965	-0.063940821	-0.241566786
Q148D1	CCDC12	bta:617519; Q148D1	ENSBTAG00000011311	-0.026327282	-0.167155756	-0.140828473
F1MRQ7	SH3BGR	bta:617797; F1MRQ7	ENSBTAG00000007068	-0.430586326	-0.356585745	0.074000581
A7YWH0	TTYH2	A7YWH0; bta:533184; F1N342	ENSBTAG00000011007	-0.095005266	0.014967576	0.109972842
P21809	BGN	bta:280733; P21809; P79259; Q17QB0; Q5BIM3		-0.194911685	-0.69045563	-0.495543945
A0A3Q1MAE3				0.073720419	0.083006764	0.009286345
A0A3Q1LVM4	DDHD1	A0A3Q1LVM4	ENSBTAG00000019924	-0.073857592	-0.133704814	-0.059847222

Accession	Gene_Symbol	Gene_ID	Ensembl_Gene ID	log2(ratio(CM12H/CM0H))	log2(ratio(CM12R/CM0H))	log2(ratio(CM12H/CM12R))
A5PJW3	TCF25	A5PJW3; bta:100848970		-0.44530129	-0.766877894	-0.321576604
A0AAA9T2R9	ZBTB7A	A0AAA9T2R9	ENSBTAG00000020117	-0.306345577	0	0.306345577
A0AAF7ACE3	PIH1D1	A0AAF7ACE3	ENSBTAG00000016526	-0.14415701	-0.0133378	0.13081921
A0AAF6DMA0	CCDC43	A0AAF6DMA0	ENSBTAG00000005693	0.302698623	0.143432103	-0.15926652
A0AAF7ADH5	VPS29	A0AAF7ADH5	ENSBTAG00000008417	0.02753595	0.062984786	0.035448836
E1BD36	IL6ST	bta:522155; E1BD36	ENSBTAG00000017745	-0.193111793	-0.140555464	0.05255633
Q2TBH6	ELP4	bta:614139; Q2TBH6	ENSBTAG00000023831	-0.028758993	-0.083738882	-0.054979889
Q3SZ96	LZIC	Q3SZ96		0.013163367	-0.051145665	-0.064309033
A0AAA9T0C4	ZNF384	A0AAA9T0C4	ENSBTAG00000017072	0.187932951	0.1502771	-0.037655851
F6Q722	PLGRKT	F6Q722	ENSBTAG00000017530	-0.007913871	0.026587003	0.034500873
F1MPV5	NFXL1	F1MPV5	ENSBTAG00000002201	0.051632768	0.045986205	-0.005646563
F1MRT5	DOK1	F1MRT5	ENSBTAG00000018579	0.12893659	0.115010854	-0.013925736
A0AAA9SH80	MRPL22	A0AAA9SH80	ENSBTAG00000019457	-0.186413124	-0.201119623	-0.014706499
E1BM89	NCBP3	bta:510317; E1BM89	ENSBTAG00000016621	-0.093659107	-0.123284276	-0.029625169
A0AAA9S0N4	GET4	A0AAA9S0N4	ENSBTAG00000026191	-0.127755547	-0.286649512	-0.158893964
A6QQU1	KATNB1	A6QQU1; bta:508813; F1MVM3	ENSBTAG00000001848	0.039528364	0.046012398	0.006484034
A0AAF6YR26	ZGPAT	A0AAF6YR26	ENSBTAG00000007498	-0.145213248	-0.136591539	0.008621709
A0AAA9TKT2	EED	A0AAA9TKT2	ENSBTAG00000007847	-0.127358698	-0.130288029	-0.002929331
A0A3Q1MEX1	RUFY2	A0A3Q1MEX1	ENSBTAG00000002614	-0.07923723	-0.112757306	-0.033520076
F6PSX0	IFI35	F6PSX0	ENSBTAG00000007389	-0.353323291	-0.189824559	0.163498732
Q5E996	NOP16	A4IF95; bta:506421; Q5E996	ENSBTAG00000010734	-0.378921829	-0.502756706	-0.123834876
A0AAF6ZJL6	AHSA2	A0AAF6ZJL6	ENSBTAG00000006025	0.325400287	0.431026795	0.105626508
Q148I5	KTI12	bta:538901; Q148I5		-0.086983302	-0.115796999	-0.028813698
A0AAF7AST6	ACSF3	A0AAF7AST6	ENSBTAG00000015968	0.158021082	0.10437262	-0.053648463
F1MDD0	PSMG4	bta:789063; F1MDD0	ENSBTAG00000031249	0.087000038	0.094387126	0.007387088
P32120	ARRB2	O77565; O77566; P32120		-0.123430979	-0.160944664	-0.037513685
A0AAA9S9N2	AKAP10	A0AAA9S9N2	ENSBTAG00000003439	0.023246344	0.014572801	-0.008673543
E1B805				-0.222205894	-0.219597042	0.002608853
Q02375	NDUFS4	bta:327680; Q02375	ENSBTAG00000003728	-0.048824709	0.012257466	0.061082174
A0AAF6DLV9	FDXR	A0AAF6DLV9	ENSBTAG00000006525	-0.01603011	-0.028983688	-0.012953578
A0A3Q1LI98	ABL1	A0A3Q1LI98; bta:540876	ENSBTAG00000017976	-0.115477217	-0.10106456	0.014412658
Q0VCF9	LSM12	bta:538005; Q0VCF9	ENSBTAG00000016252	0.095252459	0.058475889	-0.036776571
A0A3Q1MGA6	WBP4	A0A3Q1MGA6	ENSBTAG00000016637	-0.048868179	-0.206266082	-0.157397904
A0A452DHW5	WDR18	A0A452DHW5	ENSBTAG00000002434	-0.07760657	-0.115714835	-0.038108265
Q3SZ13	UQCC2	bta:100607974; Q3SZ13; Q58DH5	ENSBTAG00000054726	0.112747425	0.164764382	0.052016958
A0AAF7AP05	MTRF1	A0AAF7AP05	ENSBTAG00000012260	-0.094823546	-0.196320736	-0.10149719
Q0P564	CRBN	bta:511585; Q0P564	ENSBTAG00000019536	-0.116204272	0.272597349	0.388801621
G3MY77	MAGT1	bta:100301169; G3MY77	ENSBTAG00000045931	-0.070812468	-0.096215315	-0.025402848
A0AAA9S9R0	RPL35	A0AAA9S9R0	ENSBTAG00000003205	-0.074000581	-0.022367813	0.051632768
P21793	DCN	bta:280760; P21793; Q3MHN1; Q5U7W0; Q861V7; Q862D9; Q862E8; Q862L4; Q862R5	ENSBTAG00000003505	0.293546294	-0.198008263	-0.491554556
Q32PB9	RPL38	A6QLN0; bta:615300; Q32PB9	ENSBTAG00000030164	-0.151571084	-0.193941883	-0.042370798
Q2NL00	GSTT1	bta:517724; Q2NL00	ENSBTAG00000040298	-0.116688621	-0.061490801	0.05519782
E1BIE4	EPHB4	E1BIE4	ENSBTAG00000003446	0.257829247	-0.047536657	-0.305365904
A0AAA9T5T6	SH3PXD2A	A0AAA9T5T6	ENSBTAG00000044153	-0.2509265	-0.161909604	0.089016897
Q29RZ9	DNAAF10	bta:540032; Q29RZ9	ENSBTAG00000010595	-0.030373649	0.091699834	0.122073483
P00429	COX6B1	bta:100270792; P00429; Q54A32		0.0366634	0.221409726	0.184746326
A0A3Q1M4P7	EXOC6	A0A3Q1M4P7	ENSBTAG00000021116	-0.01994541	0.064560886	0.084506296
E1BKA4	HAUS4	E1BKA4	ENSBTAG00000012723	-0.073421069	-0.043319038	0.030102032
A0A3Q1M7S6	TPST2	A0A3Q1M7S6	ENSBTAG00000010368	0.074061867	0.144506642	0.070444775
A0A3Q1LJH6	CEP192	A0A3Q1LJH6	ENSBTAG00000013380	-0.023722911	-0.282232394	-0.258509483
A0A3Q1MKE9	IL1RN	A0A3Q1MKE9	ENSBTAG00000019665	-0.095157233	0.020319984	0.115477217
A0A343QZ23	ND5	A0A343QZ23		-0.010786555	-0.00673214	0.004054415
Q02367	NDUFB6	bta:327665; Q02367; Q3T079		0.009567885	0.220824274	0.21125639
A0A3Q1MI06	LTV1	A0A3Q1MI06	ENSBTAG00000032163	-0.286485265	-0.440837752	-0.154352487
A0AAA9TUV1	ATRN	A0AAA9TUV1	ENSBTAG00000003848	0.071099025	0.003308935	-0.06779009
A0AAA9SMX1	TRAPPC3	A0AAA9SMX1	ENSBTAG00000005732	0.202095492	0.202095492	0
A0A3Q1MTM4	COX15	A0A3Q1MTM4	ENSBTAG00000045703	0.185195516	0.117206327	-0.067989189
A0A3Q1LJU5	EML2	A0A3Q1LJU5	ENSBTAG00000015764	0.001437663	0.16816591	0.166728246
A0AAA9SPV0	H6PD	A0AAA9SPV0	ENSBTAG00000004246	-0.037847631	-0.057820654	-0.019973024
A4IFD1	PDCD4	A4IFD1; bta:506724	ENSBTAG00000019434	0.368208367	-0.127843821	-0.496052188
Q3T0Z3	UBL5	bta:614551; Q3T0Z3	ENSBTAG00000049452	-0.271553085	-0.411401979	-0.139848893
F1MLI8	PODXL	F1MLI8	ENSBTAG00000010452	0	-0.047425346	-0.047425346
Q3T0C6	ATP1B3	bta:532844; Q3T0C6	ENSBTAG00000014140	0.169058258	0.148098639	-0.020959619
A0AAF6YJZ6	RPS28	A0AAF6YJZ6	ENSBTAG00000002468	-0.097047773	-0.124556088	-0.027508315
A0AAA9RTG5	TOR1B	A0AAA9RTG5	ENSBTAG00000002837	0.15461701	-0.026402999	-0.181020009
A0AAA9TXF1				-0.514785007	-0.371707143	0.143077864
A0A140T853	IDI1	A0A140T853; bta:514293	ENSBTAG00000004075	0.081079606	0.136705205	0.055625599
A0AAA9TVG8	DNAJB2	A0AAA9TVG8	ENSBTAG00000013782	0.604485059	0.127822174	-0.476662885
G5E513				-0.504344041	-0.271683284	0.232660757
A0AAA9THB3	UTP18	A0AAA9THB3	ENSBTAG00000020629	-0.231917929	-0.107963432	0.123954497
F1N353	BNIP1	bta:614520; F1N353	ENSBTAG00000002021	0.234249695	0.024223597	-0.210026099
Q2TBL9	TRAPPC4	bta:785345; Q2TBL9	ENSBTAG00000008075	-0.253956744	-0.035558866	0.218397878
E1BLT8	ZDHHC5	bta:533250; E1BLT8	ENSBTAG00000002381	-0.002865334	0.167378319	0.170243653
A6H754	CDC37L1	A6H754; bta:510572	ENSBTAG00000017137	0.072603749	0.100362788	0.027759039
A0A0F7RQ40	DDT	A0A0F7RQ40; bta:615999		-0.259846128	-0.308426309	-0.04858018
F1MKU4	USP1	F1MKU4	ENSBTAG00000020451	-0.078280114	-0.29716019	-0.218880076
A0AAA9SFW5	REL	A0AAA9SFW5	ENSBTAG00000015386	0.008037319	0.119068631	0.111031312
E1BH05	ARHGEF10	E1BH05	ENSBTAG00000008585	-0.367939252	-0.466684733	-0.09874548
A0AAA9RTD6				-0.042828972	-0.116686564	-0.073857592
Q0IIJ1	ERGIC2	bta:512481; Q0IIJ1		0.053170659	0.098331558	0.045160899
A0A3Q1LM29	TRIM24	A0A3Q1LM29; bta:537246	ENSBTAG00000002431	0.064576943	-0.025168228	-0.08974517
A0AAA9T6M9	AGPAT1	A0AAA9T6M9	ENSBTAG00000004442	0.072886715	0.098368745	0.025482029
F1MRB9	NOL8	F1MRB9	ENSBTAG00000018613	-0.358366796	-0.325332017	0.033034779
A4IFM5	CDCA8	A4IFM5; bta:508022	ENSBTAG00000014326	0.075031692	0.192816981	0.117785289
Q00361	ATP5ME	bta:338040; Q00361; Q3T0S4		0.008461579	-0.152003093	-0.160464672
Q32PJ3	ORC3	bta:523714; Q32PJ3		-0.11808607	-0.16699567	-0.0489096
A0AAF6YL90	ANGEL2	A0AAF6YL90	ENSBTAG00000003702	-0.275503758	-0.153107126	0.122396631
A0A452DHL8	HDHD3	A0A452DHL8	ENSBTAG00000000250	0.169512862	0.129388977	-0.040123885
F1ML72	RPL34	bta:505603; F1ML72	ENSBTAG00000014226	-0.17884808	-0.167178087	0.011669993

Accession	Gene_Symbol	Gene_ID	Ensembl_Gene ID	log2(ratio(CM12H/CM0H))	log2(ratio(CM12R/CM0H))	log2(ratio(CM12H/CM12R))
A5D7A6	UTP11L	A5D7A6; bta:537675		-0.261516578	-0.339434813	-0.077918235
A0AAA9SP21	SDCBP	A0AAA9SP21	ENSBTAG00000019910	0.025385318	-0.050676887	-0.076062206
Q2KIU0	VTI1B	bta:780809; Q2KIU0		0.096021189	0.01496585	-0.081055339
E1BKH5	SETDB1	bta:100138100; E1BKH5	ENSBTAG00000000098	-0.051174844	-0.102731455	-0.051556611
F1MDG4	FUT4	F1MDG4	ENSBTAG00000038062	0.028970753	0.011658205	-0.017312548
A0A059NZ16				0.06898661	-0.16514847	-0.23413508
A0AAF6ZKW8	NDUFS7	A0AAF6ZKW8	ENSBTAG00000019419	-0.192476843	-0.112633888	0.079842955
Q2TA42	ARGLU1	bta:540543; Q2TA42	ENSBTAG00000015124	0.244637849	0.247483402	0.002845553
A0AAA9TU04	SCO1	A0AAA9TU04	ENSBTAG00000021780	0.032926005	0.176470131	0.143544126
F1MWS4	RNF25	F1MWS4	ENSBTAG00000003817	0.200331271	0.033086792	-0.167244479
A0AAA9RUC1	MECR	A0AAA9RUC1	ENSBTAG00000017253	-0.291648891	-0.341036918	-0.049388027
A0AAA9SND8	EHHADH	A0AAA9SND8; bta:518852	ENSBTAG00000019625	-0.026756458	0.076091949	0.102848408
Q29448	HMGCL	bta:317658; Q148J2; Q29448	ENSBTAG00000021832	0.080752198	0.080752198	0
A4FUH5	NDUFAF4	A4FUH5; bta:615440	ENSBTAG00000003174	0.050173289	-0.029473855	-0.079647145
A0A3Q1NNF6	GTF3C4	A0A3Q1NNF6; bta:540249	ENSBTAG00000004091	-0.292386249	-0.029058284	0.263327965
Q17QR4	CCDC137	bta:511225; Q17QR4	ENSBTAG00000000356	-0.054883907	-0.329743748	-0.274859841
A0AAA9RZ33	PMM1	A0AAA9RZ33	ENSBTAG00000004009	-0.145197916	-0.060066569	0.085131347
A0A3Q1LXD0	DDX49	A0A3Q1LXD0	ENSBTAG00000000811	-0.012887684	-0.02154388	-0.008656196
P23727	PIK3R1	bta:282307; P23727		0.046235466	0.087137654	0.040902187
F1N1D8	MINPP1	bta:541067; F1N1D8	ENSBTAG00000005928	-0.244370019	-0.469891253	-0.225521234
Q07112	GGFBPP5	bta:281361; Q07112		0.518764011	-0.041511611	-0.560275622
A0AAA9SU98	DYM	A0AAA9SU98	ENSBTAG00000000024	-0.06165047	0.022809883	0.084460353
A0AAF6DMC6	SCYL3	A0AAF6DMC6	ENSBTAG00000014794	0.050843677	-0.006324846	-0.057168523
E1B860	IFT140	bta:100139697; E1B860	ENSBTAG00000007245	-0.227410496	-0.254472392	-0.027061896
A0AAA9RTY1	CD9	A0AAA9RTY1	ENSBTAG00000014764	0.348486306	0.5600467	0.211560393
E1BM28	DNPH1	E1BM28	ENSBTAG00000019913	-0.861932078	-0.07106219	0.790869888
E1BG95	UBP1	E1BG95	ENSBTAG00000003968	-0.105810283	-0.091960304	0.013849979
A0A3Q1MAS5	CDK5RAP2	A0A3Q1MAS5	ENSBTAG00000006532	-0.045059723	-0.034116345	0.010943378
A0AAA9SNQ8	OS9	A0AAA9SNQ8	ENSBTAG00000007152	0.220545574	0.046610942	-0.173934632
Q3SX30	PSMF1	bta:617807; Q1JPI8; Q3SX30	ENSBTAG00000018417	0.074216107	0.022809883	-0.051406225
A8YXY3	SELENOF	A8YXY3; bta:614360	ENSBTAG00000052268	0.124148328	0.210064392	0.085916064
F1MYT8	TRAF3IP2	F1MYT8	ENSBTAG00000008600	-0.299979122	0.270951999	0.570931121
A0A3Q1ML04	SBF2	A0A3Q1ML04	ENSBTAG00000043981	-0.078855421	-0.090692985	-0.011837564
A0A140T863				0.168137376	-0.084268917	-0.252406293
A6QQI8	MMAA	A6QQI8	ENSBTAG00000012800	0.137241715	0	-0.137241715
A0AAF6ZP66	MARCKSL1	A0AAF6ZP66	ENSBTAG00000046862	0.044534685	-0.202619962	-0.247154647
F1MSX4	MAP3K2	bta:504592; F1MSX4	ENSBTAG00000004216	0.159314983	0.029333494	-0.12998149
A0A3Q1LR42	NADK2	A0A3Q1LR42	ENSBTAG00000011334	0.015266757	-0.027894006	-0.043160763
A0A3Q1LT08	STX4	A0A3Q1LT08	ENSBTAG00000007523	-0.037103995	-0.031333207	0.005770788
F6QK68	B4GALT7	F6QK68	ENSBTAG00000001767	-0.100088324	-0.107014382	-0.006926058
A0AAA9SAU0	KHNYN	A0AAA9SAU0	ENSBTAG00000010666	0.073691951	0.07075666	-0.002935291
A0A452DIX0	PCYOX1L	A0A452DIX0	ENSBTAG00000019951	-0.212253975	0.107787011	0.320040985
A6QQ16	NSL1	A6QQ16; bta:506867; E1BPS9		0.172052026	0.020484858	-0.151567168
A6QLC4	CDH1	A6QLC4		-0.059533323	-0.027894006	0.031639317
F1MKN3	MRPS34	F1MKN3	ENSBTAG00000026415	-0.22557929	-0.170084178	0.055495113
A0A3Q1LYZ0	RIC1	A0A3Q1LYZ0	ENSBTAG00000001283	-0.246621316	-0.338251791	-0.091630475
A0A1D8QLT6				-0.092767169	-0.006431999	0.086335169
A6QQ81	DSN1	A6QQ81; bta:508271	ENSBTAG00000013915	0.084482065	0.018859027	-0.065623038
E1BHG2	BEND3	E1BHG2	ENSBTAG00000012534	-0.252187024	-0.193423021	0.058764003
Q3T0E0	ATOX1	bta:613998; Q3T0E0	ENSBTAG00000008340	0.070389328	0.28656992	0.216180592
Q27955	KCNAB2	bta:541597; Q27955; Q58HC4		-0.177352522	0.035433693	0.212786216
A0A3Q1MQA4	ZFAND2B	A0A3Q1MQA4	ENSBTAG00000020606	0.143527311	-0.001509885	-0.145037196
E1BIL7	ZKSCAN1	bta:100140434; E1BIL7	ENSBTAG00000016757	-0.032389829	0.106019707	0.138409536
Q3ZCD0	CD81	bta:511435; Q3ZCD0		0.24914888	0.11624237	-0.13290651
Q2KIS5	HSD17B11	bta:527592; Q2KIS5	ENSBTAG00000006307	0.149235996	0.072444742	-0.076791254
Q8MJJ1	SPG21	bta:404069; Q17QJ9; Q8MJJ1	ENSBTAG00000011540	-0.087368229	0.120475884	0.207844113
F1MG31	CDCA2	F1MG31	ENSBTAG00000002756	0.163314374	-0.024728802	-0.188043176
A0A452DJ48	ALKBH5	A0A452DJ48	ENSBTAG00000025046	-0.145412055	-0.228268988	-0.082856932
G3MZK0	ADO	bta:100297937; G3MZK0	ENSBTAG00000045717	0.083591812	0.208014775	0.124422963
E1BMF7	USP13	bta:100141289; E1BMF7	ENSBTAG00000005946	-0.201198631	-0.339131166	-0.137932536
A0AAA9S5N1	BDH2	A0AAA9S5N1	ENSBTAG00000002526	-0.029682916	0.085551444	0.11523436
A0A3Q1M3C1	TEAD1	A0A3Q1M3C1	ENSBTAG00000032657	0.135106024	0.130599945	-0.004506079
A0A3Q1LN92	TGM3	A0A3Q1LN92	ENSBTAG00000001785	-0.323976247	-0.437651695	-0.113675449
A0A3Q1N1V2	NIT2	A0A3Q1N1V2	ENSBTAG00000004833	0.090386236	0.103468699	0.013082463
E1BNF0	RBM33	E1BNF0	ENSBTAG00000010243	0.121745272	0.056350854	-0.065394418
Q0II22	RNF126	bta:507447; Q0II22	ENSBTAG00000014349	-0.158922507	-0.067837533	0.091084975
Q2KJ71	TRA2A	bta:534586; Q2KJ71	ENSBTAG00000012622	-0.058364163	-0.115053581	-0.056689418
F1MYS0	BBX	bta:533187; F1MYS0	ENSBTAG00000006893	0.163848519	-0.036848831	-0.20069735
A0AAF6Z8E2	FHIP1B	A0AAF6Z8E2	ENSBTAG00000020515	-0.548327499	-0.388366884	0.159960615
F1MW70	DUSP12	bta:505302; F1MW70	ENSBTAG00000021754	0.025090981	0.00297463	-0.022116351
A0A0X9PX45	RAD51	A0A0X9PX45		0.459290737	0.066621051	-0.392669686
A0AAA9SCB7	LEO1	A0AAA9SCB7	ENSBTAG00000013830	0.067545559	-0.032914622	-0.100460182
E1BFW1	CCDC134	bta:516012; E1BFW1	ENSBTAG00000021173	0.188887514	0.191810909	0.002923395
Q2TBL4	POLR3C	bta:507314; Q2TBL4	ENSBTAG00000021637	-0.003798234	-0.137882897	-0.134084663
F1MIB3	ZNF462	F1MIB3	ENSBTAG00000019187	-0.082035769	-0.365161593	-0.283125824
A0AAA9TM42	CDK14	A0AAA9TM42	ENSBTAG00000048664	0.025639821	-0.081877007	-0.107516828
Q58DW3	RPL29	bta:507270; Q58DW3	ENSBTAG00000018628	-0.089737105	0.064751252	0.154488357
Q3T0D3	NTAQ1	bta:510463; Q3T0D3	ENSBTAG00000009916	-0.102233658	0.052726576	0.154960234
F1MZC3	RPP40	F1MZC3	ENSBTAG00000003332	-0.325906378	-0.3604636	-0.034557222
A0AAF6YKL8	CCDC115	A0AAF6YKL8	ENSBTAG00000015841	0.017629894	-0.051154088	-0.068783982
A0AAA9RYP5	RBMS1	A0AAA9RYP5	ENSBTAG00000005180	-0.05292332	-0.047260133	0.005663187
Q3ZCB8	EMC3	bta:508371; Q3ZCB8	ENSBTAG00000003882	0.181169759	0.008682243	-0.172487516
A0AAA9SK35	PRKG2	A0AAA9SK35	ENSBTAG00000002978	0.487938046	0.300659478	-0.187278568
E1BPW0	ENTPD5	E1BPW0		0.163190167	0.117469432	-0.045720736
A6H7A8	DNTTIP1	A6H7A8; bta:505524		-0.662536926	-0.506959989	0.155576937
A0A3Q1MNH6				0.306552869	0.357842098	0.051289229
A0A3Q1MH99	ZNF207	A0A3Q1MH99	ENSBTAG00000008883	0.369382487	0.139039123	-0.230343365
F1MMM8	MRPL19	F1MMM8	ENSBTAG00000002557	-0.319416876	-0.17906499	0.140351886
Q92176	CORO1A	bta:282196; Q3SZF9; Q92176	ENSBTAG00000008631	0.034125784	0.063796651	0.029670867
Q1RMU6	XXYLT1	bta:533703; F1N283; Q1RMU6	ENSBTAG00000014564	0.069953534	0.081529885	0.011576351
Q1LZD3	TSSC4	bta:509559; Q1LZD3	ENSBTAG00000047793	-0.192834694	-0.248508292	-0.055673598

Accession	Gene_Symbol	Gene_ID	Ensembl_Gene ID	log2(ratio(CM12H/CM0H))	log2(ratio(CM12R/CM0H))	log2(ratio(CM12H/CM12R))
F1MRP0	GHDC	F1MRP0	ENSBTAG00000000666	-0.220080039	-0.117098093	0.102981946
A0A3Q1ME72	DOP1B	A0A3Q1ME72	ENSBTAG00000014306	-1.2304	-1.327261539	-0.096861539
A0AAA9SK55	RAB4B	A0AAA9SK55	ENSBTAG00000009079	-0.169925001	-0.074224585	0.095700416
Q0III8	RNASET2	bta:508245; Q0III8		0.005222431	0.133574257	0.128351825
G3MZ52	CGGBP1	bta:613824; G3MZ52	ENSBTAG00000046959	0.105749732	-0.098159511	-0.203909243
A0AAA9TPV0	LIN7C	A0AAA9TPV0	ENSBTAG00000001460	0.072557165	0.012098143	-0.060459022
A5PJP3	RAB22A	A5PJP3; bta:617385		0.099636805	0.072482754	-0.027154052
Q1LZ74	OARD1	bta:768056; Q1LZ74	ENSBTAG00000005975	0.233700912	-0.021556143	-0.255257055
Q1LZC4	MGME1	bta:506205; F6Q192; Q1LZC4	ENSBTAG00000003425	-0.149768089	0.059162072	0.208930162
E1BLQ9	LPIN3	E1BLQ9	ENSBTAG00000009165	-0.238189213	-0.174530576	0.063658637
F1MS53	RAPGEF6	F1MS53	ENSBTAG00000000682	-0.044854236	-0.061656591	-0.016802356
A2VE79	NUDT3	A2VE79; bta:618855	ENSBTAG00000019684	0.068028607	0.076861462	0.008832854
Q32P59	SLIRP	bta:613614; Q32P59		-0.1177778	-0.224923264	-0.107145464
A0AAA9THI0	HTRA2	A0AAA9THI0	ENSBTAG00000020124	0.132479624	0.158175239	0.025695615
A0A3Q1NNN2	SLC7A5	A0A3Q1NNN2	ENSBTAG00000006731	0.041941763	0.011304231	-0.030637533
A0A3Q1MAN2	MRTFB	A0A3Q1MAN2	ENSBTAG00000008728	0.009143412	0.03878446	0.029641048
E1BCC2	TSC22D2	bta:541277; E1BCC2	ENSBTAG00000018402	0.034835375	-0.028187226	-0.063022602
A0AAF6YMV1	KRT19	A0AAF6YMV1	ENSBTAG00000004905	0.181867304	0.110215308	-0.071651996
A0AAF7ALD7	FAM110B	A0AAF7ALD7	ENSBTAG00000050550	-0.014781841	-0.058526077	-0.043744236
H7BWV5	CUTC	H7BWV5	ENSBTAG00000002971	0.010226742	0.231162631	0.220935889
A0AAA9SMC0	GMIP	A0AAA9SMC0	ENSBTAG00000015144	-0.124098927	0.140495993	0.26459492
A0A3Q1M238	SPPL2A	A0A3Q1M238; bta:530325	ENSBTAG00000011831	0.017487427	0.127606439	0.110119012
A0AAA9SX30	EVI5	A0AAA9SX30; bta:536706	ENSBTAG00000011363	-0.077527863	-0.140497408	-0.062969544
A0AAA9SY66	FAM177A1	A0AAA9SY66	ENSBTAG00000009680	-0.142468877	-0.006536917	0.13593196
A0A3Q1LN49	CDK17	A0A3Q1LN49	ENSBTAG00000001510	0.108354062	-0.081178404	-0.189532466
E1BGY1	FAM111B	E1BGY1	ENSBTAG00000036016	0.273429577	0.0669787	-0.206450877
A0AAA9TLP0	HEBP2	A0AAA9TLP0	ENSBTAG00000000243	0.031060296	0.172887236	0.14182694
A5PJ71	MRPL41	A5PJ71; bta:506521	ENSBTAG00000039138	-0.117975015	-0.135031707	-0.017056692
B7TCI5	TBL1X	B7TCI5; bta:515972	ENSBTAG00000018112	0.038723432	-0.030085726	-0.068809158
A5D7S7	LOX	A5D7S7; bta:280841	ENSBTAG00000012994	0.110017157	-0.103451349	-0.213468506
F1N3C5	TBC1D5	F1N3C5	ENSBTAG00000030581	-0.159087022	-0.189301635	-0.030214613
P61602	NCALD	A1L533; bta:281940; P29554; P61602; Q0IIL3; Q9H0W2	ENSBTAG00000026963	0.161812356	0.493110973	0.331298618
E1BCP5	C19H17orf75	E1BCP5	ENSBTAG00000020994	-0.496691653	-0.425763906	0.070927747
G5E5Y0	SNRPB2	G5E5Y0	ENSBTAG00000027506	-0.074998148	-0.121353784	-0.046355636
P68301	MT2A	bta:404070; P09579; P68301; Q546G1	ENSBTAG00000023659	-0.283206285	-0.299724215	-0.01651793
Q3ZBN8	DNAJC19	bta:513918; Q3ZBN8	ENSBTAG00000023513	0.013740057	0.065780075	0.052040018
A0A3Q1M0Z5	GTF3C3	A0A3Q1M0Z5	ENSBTAG00000007779	-0.08709857	-0.14232054	-0.05522197
Q32LA2	THEX1	bta:540728; Q32LA2	ENSBTAG00000009685	-0.227721925	-0.06128953	0.166432395
Q17QM9	CNRIP1	bta:539715; Q17QM9	ENSBTAG00000001408	0.062374053	-0.036580357	-0.09895441
Q29RQ1	C7	bta:507339; Q29RQ1		-0.206016592	0.015445058	0.22146165
A0A3Q1MTJ1	MACO1	A0A3Q1MTJ1	ENSBTAG00000002988	-0.219028187	-0.374811397	-0.15578321
A0AAF6YVI0	APOO	A0AAF6YVI0	ENSBTAG00000010937	0.064316824	0.104768868	0.040452044
A0A3Q1M2M3	ZNF148	A0A3Q1M2M3	ENSBTAG00000050482	-0.095559033	-0.033008762	0.062550271
A0A3Q1MEG2	BECN1	A0A3Q1MEG2	ENSBTAG00000019914	0.08228463	0.088308418	0.006023788
E1BGS8	ATG16L1	E1BGS8	ENSBTAG00000003928	0.08121835	0.116050922	0.034832572
A0AAA9THL1	AATF	A0AAA9THL1; bta:786013	ENSBTAG00000021927	-0.212360268	-0.337401852	-0.125041584
A0AAA9S240	COX5A	A0AAA9S240	ENSBTAG00000017267	-0.379269806	0.394361326	0.773631132
A0A3Q1N285	SECISBP2	A0A3Q1N285	ENSBTAG00000001939	0.053515509	-0.007006789	-0.060522298
E1BN44	RREB1	E1BN44	ENSBTAG00000005980	0.124287072	-0.075021736	-0.199308808
F1N406	EML6	bta:516921; F1N406	ENSBTAG00000011101	-1.000779624	-0.710705966	0.290073659
Q17QB7	RAB30	bta:540583; Q17QB7	ENSBTAG00000002417	0.140743976	0.08736288	-0.053381096
A0AAA9SCB5	AKT3	A0AAA9SCB5	ENSBTAG00000017788	0.040546792	0.138749106	0.098202314
Q3ZC01	CNOT7	bta:508055; Q3ZC01	ENSBTAG00000018036	0.073674733	0.003904454	-0.069770278
A0A3Q1M0R0	ADGRE5	A0A3Q1M0R0	ENSBTAG00000021818	0.065555222	0	-0.065555222
A6QLA2	PI4K2B	A6QLA2; bta:521790		0.091974215	-0.038209949	-0.130184164
Q0P5N2	TSPYL1	bta:538541; Q0P5N2	ENSBTAG00000010885	0.096215315	0.022779869	-0.073435447
A0AAF6Z5M1	RABGGTB	A0AAF6Z5M1	ENSBTAG00000018447	0.077478753	0.182660989	0.105182237
A0A3Q1MQS6	SURF1	A0A3Q1MQS6	ENSBTAG00000030384	-0.032061209	-0.128022471	-0.095961262
Q3SZA4	SLC25A20	bta:518758; F1MCM8; Q3SZA4	ENSBTAG00000000191	0.227382458	0.188939379	-0.038443079
A7MAY9	ZNF592	A7MAY9; bta:516451		0.115880712	0.169341977	0.053461265
E1BHW7	RMC1	bta:100140797; E1BHW7	ENSBTAG00000015190	-0.006126103	0.106041637	0.11216774
E1BEC1	WDR7	E1BEC1	ENSBTAG00000004005	0.00453916	0.055008947	0.050469788
A0AAF6DM62	KHDC4	A0AAF6DM62	ENSBTAG00000000108	-0.089393663	-0.574315257	-0.484921593
F1MN39	IFRD1	F1MN39	ENSBTAG00000010549	-0.443435082	-0.362161615	0.081273467
A6QQT6	REEP3	A0A3Q1MR20; A6QQT6; bta:512704; F1MV24	ENSBTAG00000019755	-0.061054035	-0.123358693	-0.062304659
F1MQH0	ELMO1	F1MQH0	ENSBTAG00000003490	0.162045708	0.211745177	0.049699469
E1B8Z4	STAT2	bta:511023; E1B8Z4	ENSBTAG00000004380	-0.00904514	0.001502025	0.010547165
A0AAA9RTE2	UQCC1	A0AAA9RTE2	ENSBTAG00000030990	0.119208233	0.273707907	0.154499675
F1N2I5	CMBL	bta:516405; F1N2I5	ENSBTAG00000010801	0.215117091	0.280705432	0.065588342
F1MMY4	NSD2	F1MMY4	ENSBTAG00000007986	-0.22704253	-0.348873243	-0.121830713
F1MM07	MYH7	F1MM07	ENSBTAG00000009703	0.002107663	1.862338277	1.860230614
G3N3L6	TIMM29	G3N3L6	ENSBTAG00000045893	0.045548473	-0.079742659	-0.125291132
A0AAA9TWV2	RAB4A	A0AAA9TWV2	ENSBTAG00000018857	-0.006821266	0.101802281	0.108623546
F1MQX7	PRMT7	F1MQX7	ENSBTAG00000009638	0.203451833	0.071053297	-0.132398536
A0A3Q1LHS7	CDC42EP4	A0A3Q1LHS7	ENSBTAG00000053044	0.146934649	0.017243572	-0.129691077
Q56K03	RPL27A	bta:404190; Q2TBT4; Q56K03		-0.091423028	0.076669768	0.168092796
Q3T171	RPL36	A5PJ75; bta:768327; Q3T171		-0.149685976	-0.174497731	-0.024811755
A0AAA9T2L5	SRCAP	A0AAA9T2L5	ENSBTAG00000013917	0.0538227	0.055176707	0.001354008
A0A3Q1LVY4	MSI2	A0A3Q1LVY4	ENSBTAG00000004406	-0.059623983	0.021738659	0.081362642
Q9N2I8	TXNRD2	bta:282389; Q9N2I8	ENSBTAG00000043581	0.020057652	-0.02180437	-0.041862023
E1BLX2	SETD1A	bta:782887; E1BLX2	ENSBTAG00000002345	0.027480736	0.135112096	0.107631359
A0A452DIQ8	NOL9	A0A452DIQ8	ENSBTAG00000016733	0.016973079	-0.061029433	-0.078002512
F1MVI8	DCAF13	F1MVI8	ENSBTAG00000021449	-0.121875951	-0.43640469	-0.314528739
A0A3Q1NEN5	NOB1	A0A3Q1NEN5	ENSBTAG00000020633	-0.403598827	-0.413602696	-0.010003869
A0AAA9TXU2	RBM19	A0AAA9TXU2	ENSBTAG00000031824	-0.403571333	-0.340145953	0.06342538
E1BJT5	NKTR	bta:100335533; E1BJT5	ENSBTAG00000002795	-0.176701411	-0.082064537	0.094636874
Q29455	DNAJC5	bta:282216; Q0VCC7; Q29455; Q29456	ENSBTAG00000015796	-0.015989731	-0.044032768	-0.028043037

Accession	Gene_Symbol	Gene_ID	Ensembl_Gene ID	log2(ratio(CM12H/CM0H))	log2(ratio(CM12R/CM0H))	log2(ratio(CM12H/CM12R))
E1BLS8	EMILIN1	E1BLS8	ENSBTAG00000011324	-0.082214763	-0.083328384	-0.00111362
A0A3Q1LZC0	GK	A0A3Q1LZC0	ENSBTAG00000008124	0.02392088	-0.052188277	-0.076109157
A0AAF6DM30	IRAK3	A0AAF6DM30	ENSBTAG00000007636	0.095715228	0.134248099	0.038532872
A0A3Q1MA47	VPS8	A0A3Q1MA47	ENSBTAG00000005209	-0.208185929	-0.153673846	0.054512082
Q02379	NDUFS5	bta:338057; Q02379; Q3ZC47	ENSBTAG00000010232	-0.132450296	-0.159118246	-0.02666795
A0A3Q1NLK1	VEZF1	A0A3Q1NLK1	ENSBTAG00000004193	-0.050774567	0.023369559	0.074144126
A0A3Q1MLQ1	MED6	A0A3Q1MLQ1	ENSBTAG00000006786	-0.054902678	-0.064488726	-0.009586049
A0AAA9T1A9	SRP14	A0AAA9T1A9	ENSBTAG00000014353	0.136372442	0.162998944	0.026626502
Q58D05	TRAFD1	bta:512642; Q08E03; Q58D05	ENSBTAG00000008761	-0.125873362	-0.254771095	-0.128897733
A0AAA9SMS2	CCNK	A0AAA9SMS2; bta:530744	ENSBTAG00000018636	0.018200179	-0.039998068	-0.058198247
A0AAA9TWM3	S100A11	A0AAA9TWM3	ENSBTAG00000015145	0.003758652	-0.318795205	-0.322553857
Q0VD19	SMPD1	bta:505097; Q0VD19	ENSBTAG00000015628	0.101570983	0.148264219	0.046693235
Q17QQ4	TAF9	bta:532936; Q17QQ4	ENSBTAG00000057772	0.124194435	0.142792971	0.018598536
A0A452DJK1	PPIF	A0A452DJK1	ENSBTAG00000016711	0.100791745	0.035800721	-0.064991024
A0JN35	ENC1	A0JN35; bta:617091		-0.86941589	-0.827490026	0.041925864
Q32T06				-0.115477217	0.106915204	0.222392421
A4FV41	UBAC2	A4FV41; bta:100125312	ENSBTAG00000005682	-0.006879818	-0.190489608	-0.18360979
A0AAA9U0I9	SPATA13	A0AAA9U0I9	ENSBTAG00000019545	0.039989731	-0.052151282	-0.092141013
F1MV20	ZFP36L2	F1MV20	ENSBTAG00000003553	0.068358793	0.034584237	-0.033774556
A0A3Q1MBH0	DLG3	A0A3Q1MBH0	ENSBTAG00000011291	0.295723025	0.264536431	-0.031186594
F1N545	ANKRD13A	bta:511883; F1N545	ENSBTAG00000014376	0.007061664	0.050088947	0.043027284
A0AAA9SZB4	TLE3	A0AAA9SZB4	ENSBTAG00000015580	0.205002284	0.129017058	-0.075985227
Q3T0D8	LAMTOR1	bta:614849; Q3T0D8		0.048560358	0.343319352	0.294758994
A6H7G8	GTF2H4	A6H7G8; bta:100137723	ENSBTAG00000046757	-0.105895559	-0.1303608	-0.02446524
A7MB61	NAB1	A7MB61; bta:516781; F1MH19	ENSBTAG00000000266	-0.005624549	-0.172734535	-0.167109986
G1FM83				-0.31413499	-0.303246674	0.010888316
A0AAA9T0E2	MRPS6	A0AAA9T0E2	ENSBTAG00000012594	0.109010622	0.046764467	-0.062246155
A0AAA9S2J4	UBAC1	A0AAA9S2J4	ENSBTAG00000039851	-0.077527863	-0.109437111	-0.031909248
E1BHI2	RMND5A	bta:516731; E1BHI2	ENSBTAG00000021140	0.063488826	0.263526038	0.200037212
A0AAA9SKZ0	SENP3	A0AAA9SKZ0	ENSBTAG00000000131	-0.213481367	-0.079940516	0.133540851
F1N6L1	VARS2	F1N6L1	ENSBTAG00000010698	-0.22216057	-0.201216556	0.020944014
A0AAA9SGC0	LIMS2	A0AAA9SGC0	ENSBTAG00000006262	0.21141713	0.026541787	-0.184875343
A0A3Q1LZK2	SAP30BP	A0A3Q1LZK2	ENSBTAG00000011717	0.181796185	0.15759649	-0.024199695
P56966	GGPS1	bta:780882; P56966; Q0VC78	ENSBTAG00000013068	0.102098188	0.124328135	0.022229947
A0A3Q1MBQ1	PRAG1	A0A3Q1MBQ1	ENSBTAG00000000357	-0.342908449	-0.281687623	0.061220826
Q1RMX9	DCUN1D5	bta:508759; Q1RMX9	ENSBTAG00000003087	-0.022271892	0.053037369	0.075309262
A0A3Q1M4M7	BCR	A0A3Q1M4M7	ENSBTAG00000020566	0.074000581	-0.049988136	-0.123988717
Q2NKZ9	SCPEP1	bta:505054; F1MLZ3; Q2NKZ9	ENSBTAG00000007893	0.054171474	0.143323852	0.089152378
F1MUW4	APAF1	F1MUW4	ENSBTAG00000021661	0.003234744	-0.003242013	-0.006476756
A0A140T891	VNN1	A0A140T891	ENSBTAG00000015094	-0.037073433	0.31152032	0.348593753
A0A3Q1M1E8	PDE3A	A0A3Q1M1E8	ENSBTAG00000017260	-0.181250323	-0.253658819	-0.072408496
A4FUI2	SNRPE	A4FUI2; bta:531493	ENSBTAG00000050130	0.10466881	-0.056812092	-0.161480902
G3N186	PARP2	G3N186	ENSBTAG00000009374	-0.267573565	-0.316901278	-0.049327712
Q2KHX4	SEC61A2	bta:614128; Q2KHX4	ENSBTAG00000019508	0.187131317	0.112347726	-0.074783591
A0AAF6YK92				-0.059256586	0.221562809	0.280819395
A0A3Q1M7D4	VLDLR	A0A3Q1M7D4	ENSBTAG00000018517	-0.324470289	-0.090132263	0.234338026
O18778	PHYH	bta:281400; O18778; Q3T0C0		-0.187501775	-0.234465254	-0.046963479
A0AAA9S627				-0.448878653	-0.456507941	-0.007629288
O77635	ADAM-9	O77635		0.050254771	-0.075985227	-0.126239997
A0AAA9T0A2	POGZ	A0AAA9T0A2	ENSBTAG00000021289	-0.206775407	-0.069124361	0.137651046
F6RTG2	PRPF39	F6RTG2	ENSBTAG00000002603	0.469662008	0.283565716	-0.186096292
A2I7N2	SERPINA3-6	A2I7N2		-0.171576627	0.295944891	0.467521519
A0JN51	ELF1	A0JN51; bta:505251	ENSBTAG00000007356	-0.283044367	0.019736218	0.302780585
A0AAA9S481	TAF7	A0AAA9S481	ENSBTAG00000070085	0.548135844	-0.079567257	-0.627703101
E1BNA3	AP4E1	E1BNA3	ENSBTAG00000013616	-0.267656254	-0.104099171	0.163557083
A0AAA9TSC9	DECR1	A0AAA9TSC9	ENSBTAG00000019663	0.038392235	0.16455441	0.126162176
Q2T9V8	DTD1	bta:514493; Q2T9V8		0.003156883	-0.051472841	-0.054629724
A0A3Q1MNC2	WLS	A0A3Q1MNC2	ENSBTAG00000012976	-0.137273814	-0.430434604	-0.293160791
Q2T9T7	DUSP3	bta:615432; Q2T9T7	ENSBTAG00000003966	0.091017892	0.178040954	0.087023062
Q0VCJ8	METTL9	bta:787067; Q0VCJ8		-0.295602268	0.027116128	0.322718396
A7YVD7	NDUFAF6	A7YVD7; bta:523017	ENSBTAG00000007570	0.022542569	-0.122951416	-0.145493985
E1BHR3	HDGFL3	bta:784070; E1BHR3	ENSBTAG00000018527	0.120345989	0.155621463	0.035275475
Q5EA01	B4GAT1	bta:618055; Q3MHK2; Q58DG2; Q5EA01	ENSBTAG00000022238	0.093822022	-0.169212383	-0.263034406
F1MZK8	PREPL	F1MZK8	ENSBTAG00000017429	-0.078933884	-0.090783602	-0.011849718
A0A3Q1MQY2	INTS9	A0A3Q1MQY2	ENSBTAG00000008845	0.00827552	-0.067970727	-0.076246247
A0A3Q1N6H0	C19H17orf49	A0A3Q1N6H0	ENSBTAG00000021931	-0.202324678	-0.278464446	-0.076139768
Q2KI32	RAB3IP	bta:533284; Q2KI32	ENSBTAG00000031950	0.121823741	0.13086888	0.00904514
A5PK43	ERAL1	A5PK43; bta:523344		-0.691185349	-0.489709497	0.201475853
A0AAA9T355	TXNDC17	A0AAA9T355; bta:404159	ENSBTAG00000056053	0.099924226	0.237304131	0.137379905
A0A3Q1NB22	PPP2R3A	A0A3Q1NB22	ENSBTAG00000023416	-0.076189563	-0.150025444	-0.073835881
A0AAA9S5X2	MYADM	A0AAA9S5X2	ENSBTAG00000018152	-0.248206988	-0.255305133	-0.007098145
Q1LZ96	ATPAF2	bta:513521; Q1LZ96	ENSBTAG00000008801	0.058404557	0.073006858	0.014602301
A6QL75	PTP4A2	A6QL75; bta:614435; F1MEE0		-0.145540843	-0.110506791	0.035034051
F1N5A3	PNISR	F1N5A3	ENSBTAG00000019730	0.231044292	-0.044726357	-0.275770649
F1MXU4	SYDE1	F1MXU4	ENSBTAG00000015184	-0.079533455	-0.366885877	-0.287352422
Q08DI1	ETAA1	bta:282224; Q08DI1		-0.118686774	-0.065072264	0.05361451
A0A3Q1MBG8	SMG7	A0A3Q1MBG8	ENSBTAG00000008003	-0.173445612	0.044840567	0.218286178
F1MI38	ICAM1	F1MI38	ENSBTAG00000010303	0.010615905	0.153494031	0.142878126
E1BBI6	CCNYL1	E1BBI6	ENSBTAG00000016297	-0.032864634	0.033720618	0.066585252
A0AAA9T3I7	B4GALT4	A0AAA9T3I7	ENSBTAG00000001390	-0.343541908	-0.241739627	0.101802281
E1BBE2	EREG	E1BBE2	ENSBTAG00000010273	1.046313874	0.756807256	-0.289506617
A0AAA9SHX1	NUBP1	A0AAA9SHX1	ENSBTAG00000009560	0.062329133	0.114742086	0.052412953
Q17QT7	CYRIA	bta:504763; Q17QT7	ENSBTAG00000012779	-0.111361109	0.197446064	0.308807173
A0AAF6DLL4	CRABP2	A0AAF6DLL4	ENSBTAG00000005716	0.258048349	0.466099199	0.20805085
A0AAF6ZJN1	RSRC2	A0AAF6ZJN1	ENSBTAG00000006118	0.005304032	0.031535573	0.026231542
Q17QQ1	LAMTOR3	bta:533155; Q17QQ1	ENSBTAG00000007923	0.15521265	0.193648832	0.038436182
A0AAA9SI39	PIP5K1A	A0AAA9SI39	ENSBTAG00000006099	-0.167996616	-0.047071834	0.120924782
A0AAA9TIG1	PHRF1	A0AAA9TIG1	ENSBTAG00000046359	-0.096215315	-0.226275856	-0.130060541
Q3SZB7	FBP1	bta:513483; Q3SZB7	ENSBTAG00000009733	0.206450877	0.428843299	0.222392421
F1MP14	FOXK1	F1MP14	ENSBTAG00000019310	-0.005912693	0.021959253	0.027871945

Accession	Gene_Symbol	Gene_ID	Ensembl_Gene ID	log2(ratio(CM12H/CM0H))	log2(ratio(CM12R/CM0H))	log2(ratio(CM12H/CM12R))
F1N4L1	SLC4A2	bta:404084; F1N4L1	ENSBTAG00000011226	-0.063807969	-0.034454056	0.029353913
A0A3Q1LSG1	TMEM123	A0A3Q1LSG1	ENSBTAG00000049233	0.174313255	-0.705392511	-0.879705766
Q0VCH8	TMEM65	bta:614243; Q0VCH8	ENSBTAG00000022114	0.075401224	0.120294234	0.04489301
F1MTC4	ZNF629	F1MTC4	ENSBTAG00000026307	0.012443063	-0.004171651	-0.016614714
Q2TBK5	TMED1	bta:538144; Q2TBK5	ENSBTAG00000010968	0.014329369	0.032363292	0.018033923
F1N3Q4	PALM	F1N3Q4	ENSBTAG00000019771	0	0.151458577	0.151458577
Q8HYI9	PFDN5	bta:282848; Q3T0V1; Q8HYI9	ENSBTAG00000008935	-0.435479297	-0.760974987	-0.32549569
Q17R02	NSD3	F6QQ19; Q17R02	ENSBTAG00000001529	0.059338668	-0.038841726	-0.098180394
F1N026	ITM2B	F1N026	ENSBTAG00000003109	-0.042600151	-0.444048586	-0.401448435
A0AAA9TBL4	NUDT1	A0AAA9TBL4; bta:525496	ENSBTAG00000002480	-0.117543623	0.02997208	0.147515702
A0AAF6Z1J5	MTIF2	A0AAF6Z1J5	ENSBTAG00000015481	0.027127178	0.078533403	0.051406225
F1MYY8	GTF3C5	F1MYY8	ENSBTAG00000021373	-0.185950425	0.094785982	0.280736408
A6H791	TRMT61A	A6H791; bta:510283		-0.198151554	-0.17625064	0.021900915
A0JNC1	CDS2	A0JNC1; bta:614834	ENSBTAG00000006066	0.107570726	0.096165963	-0.011404763
A0AAA9U1A1	ACSL5	A0AAA9U1A1	ENSBTAG00000006707	-0.026689878	0.088326782	0.11501666
A0A3S5ZPA0	EXT2	A0A3S5ZPA0	ENSBTAG00000019644	0.173148902	-0.281689605	-0.454838507
F1MN55	TAPT1	F1MN55	ENSBTAG00000019451	-0.139485054	-0.222863967	-0.083378914
A0AAA9SV06	ORC1	A0AAA9SV06	ENSBTAG00000002719	-0.190964986	-0.562775737	-0.371810752
A3KN49	HIF1AN	A3KN49; bta:520864		0.431511139	0.177269852	-0.254241287
A0AAA9SGV5	TPD52	A0AAA9SGV5	ENSBTAG00000007335	0.402098444	0.378166934	-0.023931509
A6QLE1	TDRD7	A6QLE1; bta:506702	ENSBTAG00000003719	-0.170566912	0.150975899	0.321542811
F1N3R0	SCRN2	F1N3R0	ENSBTAG00000001828	-0.065509953	-0.121247161	-0.055737207
P67810	SEC11A	A6QQV7; bta:282078; O75957; P21378; P67810	ENSBTAG00000011239	-0.030502088	-0.06166302	-0.031160931
A0AAA9SS58	DYNC2I2	A0AAA9SS58	ENSBTAG00000015343	-0.357635188	-0.244521108	0.11311408
A0AAA9S1I4	MAX	A0AAA9S1I4	ENSBTAG00000017994	0.139540749	0.020779917	-0.118760832
A0AAA9T6R0	PRKD1	A0AAA9T6R0	ENSBTAG00000012789	0.057333175	0.06738684	0.010053665
Q3SZC0	ERH	bta:508901; Q3SZC0	ENSBTAG00000017798	-0.067434361	0.294509413	0.361943774
A0A3Q1MHW6	TBC1D12	A0A3Q1MHW6	ENSBTAG00000009693	0.133441498	-0.010448949	-0.143890448
A0A3Q1LP41	MON1B	A0A3Q1LP41	ENSBTAG00000052957	-0.252660591	-0.199033406	0.053627185
E1BGD5	MUL1	E1BGD5	ENSBTAG00000020850	0.053335268	-0.039131245	-0.092466513
A3KMZ9	SNRNP27	A3KMZ9; bta:790677		0.16649706	-0.065914117	-0.232411177
G3MZF6	WDR76	G3MZF6	ENSBTAG00000017215	0.287481949	0.220520979	-0.06696097
A0A3Q1LZZ0	ATP6AP1	A0A3Q1LZZ0	ENSBTAG00000012117	0.027852121	-0.140177658	-0.168029779
A0AAA9S1C6	LRRN4CL	A0AAA9S1C6	ENSBTAG00000010484	-0.043832081	0.021426558	0.065258639
G3MYZ7	USP34	G3MYZ7	ENSBTAG00000006027	-0.123106506	-0.014116502	0.108990003
A0A3Q1MUA3	COL15A1	A0A3Q1MUA3	ENSBTAG00000010082	-0.175535221	-0.050295116	0.125240104
A0AAA9RZF9	TNS2	A0AAA9RZF9	ENSBTAG00000006904	-0.163498732	-0.128065039	0.035433693
A0AAF6Z9X3	USP21	A0AAF6Z9X3	ENSBTAG00000021893	-0.114288542	-0.108123169	0.006165373
F1MLY7	ATR	bta:504869; F1MLY7	ENSBTAG00000011529	-0.199122642	-0.181913352	0.01720929
A0AAF6ZVD1				0.269320669	0.26670472	-0.002615948
E1BIJ5	N4BP2	E1BIJ5	ENSBTAG00000000175	-0.066753044	-0.282276734	-0.21552369
Q2TBW5	ZNHIT2	bta:539138; Q2TBW5	ENSBTAG00000063864	-0.076594156	0.210622241	0.287216397
A0AAA9SAS6	HDAC8	A0AAA9SAS6	ENSBTAG00000046092	-0.569202075	-0.199543595	0.36965848
Q2YDD3	ZFPL1	bta:613442; Q1JP84; Q2YDD3	ENSBTAG00000015479	-0.184667021	-0.078076589	0.106590431
G8JL05	LPCAT3	G8JL05	ENSBTAG00000013127	0.30565404	-0.089754653	-0.395408693
A0A3Q1M4C0	CEP85	A0A3Q1M4C0; bta:517520	ENSBTAG00000009579	0.130715662	0.14178145	0.011065788
G5E690	GGA3	G5E690	ENSBTAG00000016128	0.230596965	0.117313051	-0.113283914
A0A3Q1MGP4	CHD9	A0A3Q1MGP4	ENSBTAG00000002287	-0.333301269	-0.243571108	0.089730161
G3X6H0	CEP170B	G3X6H0	ENSBTAG00000004802	0.00892523	-0.128324097	-0.137249327
A0AAA9SGX1	EMB	A0AAA9SGX1	ENSBTAG00000044010	0.212937483	0.046565349	-0.166372134
A0A0N4STN0	ATP5MG	A0A0N4STN0; bta:515696	ENSBTAG00000007332	-0.049105472	0.029515492	0.078620964
F1MS34	RTKN	F1MS34	ENSBTAG00000000490	-0.293594466	-0.201351285	0.092243181
A0A3Q1LNU2	DYRK1A	A0A3Q1LNU2	ENSBTAG00000001771	-0.250479519	-0.286213731	-0.035734212
F6RFU1	SHF	F6RFU1	ENSBTAG00000020934	-0.101283336	-0.118356849	-0.017073513
A0AAA9SRA7	PWWP3A	A0AAA9SRA7	ENSBTAG00000030839	-0.352353508	-0.049496966	0.302856542
A0A3Q1NIL1	WWP2	A0A3Q1NIL1	ENSBTAG00000018421	-0.062931256	-0.047696742	0.015234514
A0AAA9SU76	TMEM126A	A0AAA9SU76	ENSBTAG00000000451	0.084120621	0.113407848	0.029287227
A0AAA9THQ6	SNX8	A0AAA9THQ6	ENSBTAG00000013708	-0.252980741	-0.341517416	-0.088536675
Q0VCG0	LYRM4	bta:614011; Q0VCG0	ENSBTAG00000031432	0.057353171	0.069142369	0.011789198
A0A3Q1MPJ7	CPPED1	A0A3Q1MPJ7	ENSBTAG00000007142	-0.224815084	0.016845671	0.241660755
A0AAF6YT67	CDC23	A0AAF6YT67	ENSBTAG00000008759	-0.187511629	-0.025139562	0.162372067
F1N332	ULK3	F1N332	ENSBTAG00000005838	0.052579915	0.023433569	-0.029146346
Q02827	NDUFC2	bta:338046; Q02827; Q3SZR4		-0.033166864	-0.166933001	-0.133766137
A0A3Q1LZP9	STARD7	A0A3Q1LZP9	ENSBTAG00000018372	0.148370858	-0.055467755	-0.203838613
Q2YDK1	ISG20L2	bta:515980; Q2YDK1	ENSBTAG00000001518	-0.06317286	-0.018378529	0.044794331
A0AAA9TUG8	DNMBP	A0AAA9TUG8	ENSBTAG00000008939	0.058893689	-0.114642566	-0.173536255
F1N582	ODR4	F1N582	ENSBTAG00000011946	-0.12702204	-0.084392187	0.042629853
E1B945	ANAPC4	E1B945	ENSBTAG00000000646	0.0135944	0.063462096	0.049867696
Q1RMV0	PEX5	A1L572; bta:514832; Q1RMV0	ENSBTAG00000010490	0.288042101	0.168493046	-0.119549055
A0AAA9SUV0	B3GLCT	A0AAA9SUV0	ENSBTAG00000033412	-0.064739198	-0.266691429	-0.201952231
P08037	B4GALT1	bta:281781; P08037; Q0VC05; Q8MIG0	ENSBTAG00000015249	-0.03970971	0.014290658	0.054000367
A0A3Q1N6J6	OSCP1	A0A3Q1N6J6	ENSBTAG00000007994	-0.164067955	-0.001423478	0.162644477
Q0VCR1	RTF2	bta:513536; Q0VCR1		0.326183466	-0.544790628	-0.870974094
Q3T0F7	MTPN	bta:541099; Q3T0F7	ENSBTAG00000007806	-0.325287107	0.462234981	0.787522088
A0AAA9S3D4	ASCC1	A0AAA9S3D4	ENSBTAG00000000392	-0.31732252	-0.245838791	0.07148373
A0A3Q1MXN1	SLC4A7	A0A3Q1MXN1	ENSBTAG00000018227	-0.067771131	0.004131826	0.071902957
A0AAA9SH66	SGO2	A0AAA9SH66	ENSBTAG00000031579	0.027585832	0.238220282	0.21063445
A0AAA9S4B2	KIF16B	A0AAA9S4B2	ENSBTAG00000000939	-0.161086198	-0.035133537	0.125952661
G3N0C3				0.015767316	-0.20069735	-0.216464666
A0A3Q1MEK8	PHKB	A0A3Q1MEK8	ENSBTAG00000004806	-0.029659974	-0.08789977	-0.058239796
A0A3Q1M5C4	TMEM33	A0A3Q1M5C4	ENSBTAG00000043958	0.27064759	0.092841021	-0.177806569
A0AAA9S542	SPIRE1	A0AAA9S542	ENSBTAG00000010542	0.103516369	0.028839683	-0.074676686
A0A3Q1MIG2	AP1S1	A0A3Q1MIG2	ENSBTAG00000014468	-0.059321091	-0.031101217	0.028219874
A1XEA0	EEF1A1	A1XEA0		0.07121071	-0.203493176	-0.274703886
Q58D31	SORD	bta:508954; Q0II66; Q58D31		-0.117809446	-0.057041196	0.06076825
A0AAA9TEF0	MED14	A0AAA9TEF0	ENSBTAG00000011790	-0.130113295	-0.111870333	0.018242962
A0A3Q1LZW1	PALM3	A0A3Q1LZW1; bta:527118	ENSBTAG00000046744	0.022660597	-0.008909482	-0.031570079
A5PJJ5	LRRC14	A5PJJ5; bta:539952	ENSBTAG00000004969	-0.44710359	-0.090071694	0.357031896
A0AAA9T881	CD200	A0AAA9T881	ENSBTAG00000033319	-0.076350886	0.069680909	0.146031795

Accession	Gene_Symbol	Gene_ID	Ensembl_Gene ID	log2(ratio(CM12H/CM0H))	log2(ratio(CM12R/CM0H))	log2(ratio(CM12H/CM12R))
A6QLI5	MAGED4	A6QLI5; bta:100125231		0.464790534	-0.200621799	-0.665412333
A0A3Q1LMF5	CYLD	A0A3Q1LMF5	ENSBTAG00000006291	0.046229843	0.167228374	0.120998531
E1BIE5	UBE2Q1	E1BIE5	ENSBTAG00000007515	-0.085469342	-0.033582601	0.051886742
A0AAA9TER0	VPS26C	A0AAA9TER0	ENSBTAG00000004791	-0.04761802	-0.072029686	-0.024411666
Q3ZBB0	SLC35B2	bta:767848; Q3ZBB0	ENSBTAG00000008076	0.114303627	0.187106161	0.072802535
A6H7E4	LARP6	A6H7E4; bta:787569		0.054966459	0.199805736	0.144839277
A0A3Q1MZF8	PTPRS	A0A3Q1MZF8	ENSBTAG00000018052	-0.150967604	-0.206361238	-0.055393634
Q5E963	ARPC5L	bta:613421; Q5E963	ENSBTAG00000015278	0.084932748	0.110747066	0.025814318
E1BGF8	GPAT3	bta:519739; E1BGF8	ENSBTAG00000017592	0.235628248	0.258693409	0.02306516
A0AAA9U040	SNU13	A0AAA9U040	ENSBTAG00000006104	-0.310089892	-0.065268289	0.244821603
F1MKN2	MPG	F1MKN2	ENSBTAG00000026422	0.159002234	0.067114196	-0.091888038
A0AAA9TFD6	PFDN1	A0AAA9TFD6	ENSBTAG00000051779	-0.040939632	-0.401215166	-0.360275534
A0A3Q1N459	C2CD5	A0A3Q1N459	ENSBTAG00000001673	-0.107035444	-0.080348994	0.02668645
A0AAA9SNV4	MRPS17	A0AAA9SNV4	ENSBTAG00000007934	-0.128033389	-0.195147585	-0.067114196
A0A3Q1LNP3	TRABD	A0A3Q1LNP3	ENSBTAG00000001931	0.03613876	0.001406821	-0.034731938
A0A3Q1M1V0	NAGPA	A0A3Q1M1V0	ENSBTAG00000007896	0.064441753	0.019919183	-0.04452257
A0AAA9TGQ0	CCDC90B	A0AAA9TGQ0	ENSBTAG00000007479	0.004171651	-0.160382759	-0.16455441
A0A452DIW5	WDR91	A0A452DIW5	ENSBTAG00000017905	-0.131644006	0.046764467	0.178408473
G3MY50	ASMTL	G3MY50	ENSBTAG00000046514	-0.054148687	-0.079948156	-0.025799469
A0A3Q1M266	RCOR3	A0A3Q1M266	ENSBTAG00000007110	-0.320508818	-0.346271914	-0.025763096
Q1LZ79	GEMIN8	bta:515968; Q1LZ79	ENSBTAG00000010289	-0.048775515	-0.16458797	-0.115812455
A0AAA9RVP2	PEX6	A0AAA9RVP2	ENSBTAG00000005532	-0.007645461	0.042081589	0.049727049
A5D7V3	ESM1	A5D7V3; bta:539571	ENSBTAG00000004221	-0.658025963	-0.637869767	0.020156196
A0A3Q1M6S6	DHTKD1	A0A3Q1M6S6	ENSBTAG00000000291	-0.186151316	0.018109381	0.204260696
P0CG53	UBB	bta:281370; O97577; P02248; P02249; P02250; P0CG53; P62990; P80169; Q01235; Q24K23; Q28169; Q28170; Q29120; Q3T0V5; Q3ZCE3; Q862C1; Q862F4; Q862M4; Q862T5; Q862X8; Q91887; Q91888	ENSBTAG00000017246	0.070328042	-0.102664566	-0.172992608
F1MBX3	ESRRA	bta:507834; F1MBX3	ENSBTAG00000008645	-0.652791256	-0.414204294	0.238586962
Q1RML7	MAK16	bta:504683; Q1RML7	ENSBTAG00000033504	-0.255807099	-0.188692903	0.067114196
E1BF52	GFER	E1BF52	ENSBTAG00000017631	0.069656166	0.004191853	-0.065464314
A4FUD2	HSD17B7	A4FUD2; bta:505212	ENSBTAG00000005976	-0.026967048	-0.022675438	0.00429161
A0A3Q1M4V2	SNRPG	A0A3Q1M4V2	ENSBTAG00000053210	0.153290639	0.053387213	-0.099903427
A0A3Q1MYX0	RNPS1	A0A3Q1MYX0	ENSBTAG00000009969	0.024615299	-0.044825199	-0.069440498
E1BAZ2	NDUFAF1	E1BAZ2	ENSBTAG00000010778	0.109502518	-0.173837079	-0.283339597
Q5E9Y2	STX17	bta:534304; Q5E9Y2	ENSBTAG00000008016	0.162528273	0.191890333	0.02936206
A0A3Q1LM43	DNAJB6	A0A3Q1LM43	ENSBTAG00000000771	0.345292088	0.002941275	-0.342350813
Q3T0E1	EXOSC3	bta:533245; Q3T0E1	ENSBTAG00000018925	0.039491051	0.036801963	-0.002689088
A2VDZ7	CDC20	A2VDZ7; bta:515376		-0.105460508	-0.266823495	-0.161362988
E1BGK1	ARID4A	E1BGK1	ENSBTAG00000018898	-0.185323167	-0.027936877	0.15738629
A0A3Q1MFU3	TRAF5	A0A3Q1MFU3	ENSBTAG00000012020	-0.32223275	-0.449565182	-0.127332432
A7YY24	IGBP1	A7YY24; bta:100296883	ENSBTAG00000007797	0.245318818	0.11268105	-0.132637769
E1BPS3	WDFY3	E1BPS3	ENSBTAG00000013495	0.005943139	-0.079574932	-0.085518071
Q02370	NDUFA2	bta:327698; Q02370; Q148D8; Q2VYC4	ENSBTAG00000015041	-0.154439754	-0.212050477	-0.057610723
A0AAA9T860	PLXNA2	A0AAA9T860; bta:407770	ENSBTAG00000001173	0.107915339	-0.116644919	-0.224560258
A0AAA9SU88	GBGT1	A0AAA9SU88	ENSBTAG00000030319	-0.045747822	0.046020164	0.091767986
Q3T904	ATG9A	A5D7D0; bta:540482; Q3T904		0.320081248	0.019857418	-0.30022383
A7YY74	GALNT4	A7YY74; bta:100125265		-0.337459247	-0.248599316	0.088859931
A0A3Q1MEP0				-0.440954874	-0.521623434	-0.08066856
Q1RML9	PLA2G7	bta:282311; Q1RML9	ENSBTAG00000019315	-0.071502948	0.051035464	0.122538413
A0AAA9S3A1	RMDN2	A0AAA9S3A1	ENSBTAG00000032905	-0.066652312	0.002769089	0.069421401
F1MLQ1	LOC524236	F1MLQ1	ENSBTAG00000034949	-0.480391237	-0.063156182	0.417235055
A6QQL0	SLC15A4	A6QQL0; bta:510499	ENSBTAG00000009167	0.158637512	0.188120534	0.029483022
E1BNB3	SNIP1	E1BNB3	ENSBTAG00000016852	0.185678543	0.051805388	-0.133873156
Q9XT97	PSEN1	bta:282705; Q9XT97		0.135192194	0.066627908	-0.068564286
A0A3Q1MD82	CDK16	A0A3Q1MD82	ENSBTAG00000016769	-0.105765105	-0.059687095	0.04607801
F1N555	GIMAP8	F1N555	ENSBTAG00000014402	0.34567571	0.279467559	-0.066208151
A0AAA9TQ78	SSH3	A0AAA9TQ78	ENSBTAG00000031590	-0.092458222	-0.014729856	0.077728366
Q2KIG1	ASF1A	bta:618099; Q2KIG1	ENSBTAG00000002058	0.249562623	0.067862029	-0.181700593
A0AAA9T6J5	ARHGAP32	A0AAA9T6J5	ENSBTAG00000015905	0.149813162	0.10546307	-0.044350092
A0AAA9SNB5	MRPL52	A0AAA9SNB5	ENSBTAG00000014822	-0.08316369	-0.168435383	-0.085271694
A3KMX1	CHST14	A3KMX1; bta:511245		-0.170579137	-0.288634898	-0.118055762
A0A3Q1MHL9	NSMCE2	A0A3Q1MHL9	ENSBTAG00000009394	-0.182968039	-0.196130072	-0.013162033
F1MTK2	MEAK7	F1MTK2	ENSBTAG00000000946	0.05117026	-0.041634489	-0.092804749
E1BGJ4	MAN2A2	E1BGJ4	ENSBTAG00000018905	-0.249978253	0.023651268	0.273629521
A0A3Q1MLJ3	PFKFB2	A0A3Q1MLJ3	ENSBTAG00000002126	-0.19163613	-0.014199869	0.177436261
A0AAA9TPX1	CCS	A0AAA9TPX1	ENSBTAG00000004343	-0.092476187	-0.144032798	-0.051556611
F1MPR9	NFRKB	bta:510304; F1MPR9	ENSBTAG00000017610	0.100710507	0.106180447	0.00546994
A0AAA9TP59	KCTD10	A0AAA9TP59	ENSBTAG00000002697	0.066733989	-0.257191838	-0.323925827
A0A3Q1MQE4	MAP2	A0A3Q1MQE4	ENSBTAG00000018130	-0.055569688	-0.037241192	0.018328496
A2VE18	STX6	A2VE18; bta:510909	ENSBTAG00000020458	0.115363157	0.01328322	-0.102079937
F1MLH9	PAX8	bta:534661; F1MLH9	ENSBTAG00000019354	0.035697369	0.029808809	-0.005888559
A6H7F6	GOLPH3L	A6H7F6; bta:532555		0.06574736	-0.064390587	-0.130137947
A6QPM9	PTMS	A6QPM9; bta:613777; F1N7I3	ENSBTAG00000018451	0.010259208	-0.277261007	-0.287520215
A0A3Q1MLQ5	SLC2A1	A0A3Q1MLQ5	ENSBTAG00000009617	-0.036594509	-0.089723004	-0.053128495
A0AAA9S613	PLCG2	A0AAA9S613	ENSBTAG00000002103	-0.070721173	-0.014673862	0.05604731
A0A3Q1LXC0	POLB	A0A3Q1LXC0	ENSBTAG00000000225	-0.146152104	-0.051085779	0.095066325
Q32PC9	ARL2BP	A4IFR7; bta:613462; Q32PC9	ENSBTAG00000004844	0.146175534	0.407513649	0.261338115
A0AAA9TN82	KIN	A0AAA9TN82	ENSBTAG00000013925	0.058364163	-0.100008456	-0.158372618
A0A3Q1M5N9	OLFML2A	A0A3Q1M5N9; bta:516730	ENSBTAG00000004848	0.113607715	0.019300556	-0.094307159
F1N6E1	SAV1	bta:516557; F1N6E1	ENSBTAG00000001539	-0.031848866	0.034240324	0.06608919
A0A3Q1MSK9	EPN2	A0A3Q1MSK9	ENSBTAG00000017771	-0.578637655	-0.632648589	-0.054010935
A2VE40	GINS4	A2VE40; bta:616799	ENSBTAG00000017133	-0.090720389	-0.153675187	-0.062954798
Q2KHX3	PRAF2	bta:508703; Q2KHX3		0.106065059	0.059726345	-0.046338715
Q32L78	TRAPPC6B	bta:521470; Q32L78	ENSBTAG00000015023	0.024023229	0.098817736	0.074794507
Q3ZBV0	TSPAN13	bta:526460; Q3ZBV0		0.078085314	0.372326296	0.294240982
F6RVL5	RGL2	F6RVL5	ENSBTAG00000016827	-0.166078279	-0.124258103	0.041820176
F1MBD5	SURF2	bta:526046; F1MBD5	ENSBTAG00000000046	-0.045715718	-0.109014586	-0.063298868

Accession	Gene_Symbol	Gene_ID	Ensembl_Gene ID	log2(ratio(CM12H/CM0H))	log2(ratio(CM12R/CM0H))	log2(ratio(CM12H/CM12R))
E1BF40	GPT2	E1BF40	ENSBTAG00000002980	-0.228869178	-0.066114434	0.162754744
A0AAA9TA89	EXD2	A0AAA9TA89; bta:539532	ENSBTAG00000010052	0.047346573	-0.128412469	-0.175759043
A0AAA9T770				-0.054774223	-0.025686644	0.029087579
A0AAA9SNY2	MOB1B	A0AAA9SNY2	ENSBTAG00000016290	0.065430091	-0.097197843	-0.162627934
A0A3Q1LX29	DOP1A	A0A3Q1LX29	ENSBTAG00000000676	0.075232426	0.109143486	0.03391106
A6QNS6	NID1	A6QNS6; bta:534319; F1MWN3	ENSBTAG00000007244	-0.23878686	0.037544369	0.276331228
A0AAA9SKB6	HAUS7	A0AAA9SKB6	ENSBTAG00000014095	-0.134129455	-0.093015574	0.041113881
A0AAA9TLK0	COMMD2	A0AAA9TLK0	ENSBTAG00000005068	0.005308911	0.023737748	0.018428837
Q3SX24	FBXO6	bta:513023; Q3SX24	ENSBTAG00000014777	-0.082168542	0.119695066	0.201863608
A0A3Q1N9Q2	TACC2	A0A3Q1N9Q2	ENSBTAG00000010786	0.059747463	-0.063922391	-0.123669854
Q3MHX1	COMMD7	bta:514295; Q3MHX1	ENSBTAG00000016427	0.305565911	0.36607065	0.060504739
F1N652	GOSR2	F1N652	ENSBTAG00000015958	0.081592424	0.061281975	-0.020310449
Q7YR75	MRPL12	A5D9H8; bta:399560; Q7YR75	ENSBTAG00000000417	-0.167324861	-0.293731203	-0.126406342
A0AAA9RWD4	JPH2	A0AAA9RWD4	ENSBTAG00000068398	-0.003954397	-0.055004799	-0.051050402
Q3SZN5	POLE3	bta:510678; Q3SZN5	ENSBTAG00000000252	0.078002512	-0.108311811	-0.186314323
Q32LE7	PDZD11	bta:515371; Q32LE7	ENSBTAG00000012860	0.072034789	-0.064273214	-0.136308003
A6H732	TMX4	A6H732; bta:618015; F1MD03	ENSBTAG00000035323	-0.143317366	-0.092273302	0.051044064
A0AAA9SKI8	PAPOLG	A0AAA9SKI8	ENSBTAG00000007414	-0.030434675	-0.10713585	-0.076701175
F1MGU7	FGG	F1MGU7	ENSBTAG00000006745	-0.246437895	-0.30579003	-0.059352134
A0A3Q1MY63	MGRN1	A0A3Q1MY63	ENSBTAG00000018999	-0.150491627	-0.255432683	-0.104941057
A0AAA9T2R6	ZNF143	A0AAA9T2R6	ENSBTAG00000016074	0.108987149	0.144873629	0.03588648
A0A140T8D4	LGMN	A0A140T8D4	ENSBTAG00000046979	0.183887154	0.005363185	-0.17852397
F6R1D7	LRATD2	F6R1D7	ENSBTAG00000026172	0.098429736	0.281083043	0.182653306
F1MQ25	MRPL49	F1MQ25	ENSBTAG00000005075	-0.163173959	-0.063638286	0.099535674
E1BBB8	TRPS1	bta:537652; E1BBB8	ENSBTAG00000017694	0.233710796	0.025823775	-0.20788702
Q28203	CD40	A7YWS9; bta:286849; Q28203		0.113501193	0.066880807	-0.046620387
Q0VCK9	VMP1	bta:508631; Q0VCK9	ENSBTAG00000011623	0.071674198	0.004492048	-0.06718215
E1BEW5	PPTC7	bta:525355; E1BEW5	ENSBTAG00000018332	0.01875396	0.1202403	0.10148634
A0AAA9TXJ2	PRUNE2	A0AAA9TXJ2	ENSBTAG00000012991	-0.213481367	-0.119594858	0.093886509
A0AAA9T3N7	ZNF385B	A0AAA9T3N7	ENSBTAG00000003120	-0.254866785	-0.277251951	-0.022385167
Q3T116	MRPL58	A7YWR4; bta:515465; Q3T116	ENSBTAG00000003303	-0.117400384	-0.146887148	-0.029486765
Q32KU6	TSPAN6	bta:514741; Q32KU6	ENSBTAG00000045550	0.18276172	0.211504105	0.028742385
O77627	JUN	O77627	ENSBTAG00000068635	0.100792924	-0.341875694	-0.442668618
A0AAA9RT10	FAM185A	A0AAA9RT10	ENSBTAG00000014879	-0.093208054	-0.011886329	0.081321724
Q2KII6	NGDN	bta:539602; Q2KII6	ENSBTAG00000009595	0.037509505	-0.172811921	-0.210321426
A0A3Q1NN25	OPA3	A0A3Q1NN25	ENSBTAG00000019605	0.06994412	0.123842076	0.053897956
A0A3Q1M7C4	FAM117B	A0A3Q1M7C4	ENSBTAG00000048876	-0.994315295	-1.412598454	-0.418283159
Q58D49	MMAB	bta:617636; Q2NL20; Q58D49		-0.113725736	-0.169399334	-0.055673598
A0AAA9T971	MTM1	A0AAA9T971	ENSBTAG00000014138	-0.020299562	0.035553673	0.055853235
F1MNB3	MIGA1	F1MNB3	ENSBTAG00000031044	-0.160899494	-0.057293225	0.103606269
A0AAA9TYF6	PTCD1	A0AAA9TYF6	ENSBTAG00000020440	-0.03058832	-0.115477217	-0.084888898
Q2KIQ7	SF3B6	bta:508876; Q2KIQ7	ENSBTAG00000003407	0.241123455	0.060212807	-0.180910648
Q95KE5	MRPL43	bta:282277; Q28183; Q2KID5; Q95KE5	ENSBTAG00000003294	-0.076379766	-0.205225138	-0.128845372
F1N6T6	TARBP1	F1N6T6	ENSBTAG00000004517	-0.11185155	-0.175541418	-0.063689868
F1N196	TBC1D17	F1N196	ENSBTAG00000047035	0.09010389	0.134542123	0.044438234
A0AAA9SFN1	SEC14L2	A0AAA9SFN1; bta:282469	ENSBTAG00000017404	-0.215776229	-0.192013655	0.023762573
A0AAA9S2W2	ADIPOQ	A0AAA9S2W2	ENSBTAG00000059085	-0.065626599	0.34250804	0.408134639
A0AAA9SGT8	DIPK1A	A0AAA9SGT8	ENSBTAG00000002028	-0.148787815	-0.148787815	0
A0AAA9T6S9	EXOSC1	A0AAA9T6S9	ENSBTAG00000012699	0.087885832	0.034114576	-0.053771256
A0A3Q1LS50	CYTH1	A0A3Q1LS50	ENSBTAG00000006663	0.023157721	-0.133617997	-0.156775718
Q2KJG8	BCKDK	bta:505005; Q2KJG8	ENSBTAG00000010524	-0.384225756	-0.328312384	0.055913372
F1MY08	ATP23	bta:514014; F1MY08	ENSBTAG00000017543	-0.053046234	-0.032924638	0.020121596
F1MYX2	APOM	F1MYX2	ENSBTAG00000008833	-0.147557188	-0.10922907	0.038328119
A0AAA9STJ0	TMEM201	A0AAA9STJ0	ENSBTAG00000003180	-0.281938364	-0.275035503	0.00690286
A0A452DIB4	NDUFA6	A0A452DIB4	ENSBTAG00000009509	-0.068505081	-0.149842157	-0.081337076
Q1RMW0	PLSCR3	bta:510355; Q1RMW0	ENSBTAG00000017747	-0.021788689	-0.067997118	-0.046208429
F1MEA6	DIS3L	F1MEA6	ENSBTAG00000000371	-0.273429577	-0.254183488	0.019246089
F6RVI7	MTO1	F6RVI7	ENSBTAG00000016839	-0.216299329	-0.19445093	0.021848399
A7MB98	ZFP36L1	A7MB98; bta:614773	ENSBTAG00000025434	0.2591042	-0.333126301	-0.592230501
A5D9D4	MSTO1	A5D9D4; bta:535567; Q29S12	ENSBTAG00000026613	-0.172161605	-0.195595174	-0.023433569
A0AAF6DLX7	MAPRE3	A0AAF6DLX7	ENSBTAG00000008884	-0.060260525	-0.107167445	-0.046906921
A0AAF6DLL6	PRKAR1B	A0AAF6DLL6	ENSBTAG00000046142	0.051753423	-0.089963377	-0.1417168
F1MK24	CNNM4	F1MK24	ENSBTAG00000019213	0.326383153	0.112614158	-0.213768994
Q0II29				0.292615396	0.164662665	-0.127952731
A5PJE5	NFATC2IP	A5PJE5; bta:505998		0.032014807	-0.072149786	-0.104164593
F1MET2	ERF	bta:789798; F1MET2	ENSBTAG00000000332	-0.124098927	-0.005742077	0.118356849
A0AAA9SKI3	ANKRD28	A0AAA9SKI3	ENSBTAG00000024815	-0.175310467	-0.159730455	0.015580011
A0A3Q1LZC5	EDIL3	A0A3Q1LZC5; bta:538062	ENSBTAG00000044033	-0.103093493	-0.026028709	0.077064784
Q2M2U4	RNASEH2C	bta:505618; Q2M2U4	ENSBTAG00000005577	0.028071757	0.038277828	0.010206071
Q5E994	CHMP1B	bta:512033; Q2KJ31; Q5E994		0.035189428	-0.037591888	-0.072781317
A6QL68	CLYBL	A6QL68	ENSBTAG00000004165	-0.006011238	0.084460353	0.090471591
E1BKP6	CEP250	E1BKP6	ENSBTAG00000006021	0.237730044	0.10408916	-0.133640884
A0AAA9TBD1	MGMT	A0AAA9TBD1	ENSBTAG00000050386	0.21998117	-0.261502069	-0.481483239
F6QAN5	NOCT	F6QAN5	ENSBTAG00000005998	-0.055941284	0.199654136	0.25559542
Q0VCM0	S100A16	bta:505679; Q0VCM0	ENSBTAG00000014204	-0.03082799	-0.102698164	-0.071870174
A0AAA9SQR3	FAM3A	A0AAA9SQR3	ENSBTAG00000012747	-0.84195799	-0.312277925	0.529680065
A0A3Q1N8K4	FAM151B	A0A3Q1N8K4	ENSBTAG00000007632	-0.043652945	-0.068315	-0.024662054
A7MB60	TTC21B	A0A3Q1M8G2; A7MB60; bta:527412; F6PR93	ENSBTAG00000016512	-0.205544306	-0.248664522	-0.043120216
A0A3Q1LXB2	TAF6L	A0A3Q1LXB2	ENSBTAG00000009484	-0.137227964	0.019163214	0.156391178
A0A3Q1MYE6	INTS2	A0A3Q1MYE6	ENSBTAG00000012070	-0.114787757	-0.182044196	-0.067256438
F1N2J0	SMG8	F1N2J0	ENSBTAG00000003365	-0.432480413	-0.438032807	-0.005552393
Q6T8E9	CCNT1	bta:407194; Q6T8E9	ENSBTAG00000005861	-0.317915032	-0.255818911	0.062096121
E1BKS1	SDC4	bta:508133; E1BKS1	ENSBTAG00000015127	0.39538054	-0.109552383	-0.504932923
A0AAA9T9X0	YAF2	A0AAA9T9X0	ENSBTAG00000015025	-0.042004675	-0.245577209	-0.203572533
A0AAA9U2N6	PIK3R2	A0AAA9U2N6	ENSBTAG00000002350	-0.032575604	-0.001696291	0.030879313
Q2TBI6	MRPL32	bta:532842; Q2TBI6	ENSBTAG00000000991	-0.070954647	-0.140898768	-0.06994412
F1MBB0	RELB	F1MBB0	ENSBTAG00000038428	0.122591814	0.277487616	0.154895801
P25417	CSTB	bta:512805; P25417; Q32PC6		0.033063208	0.300794938	0.267731729
E1BBR9	SPTY2D1	bta:539967; E1BBR9	ENSBTAG00000001660	0.492906323	0.244000839	-0.248905484
A0AAF7A7H1	MAN2B1	A0AAF7A7H1	ENSBTAG00000006241	-0.038279688	-0.092869575	-0.054589886

Accession	Gene_Symbol	Gene_ID	Ensembl_Gene ID	log2(ratio(CM12H/CM0H))	log2(ratio(CM12R/CM0H))	log2(ratio(CM12H/CM12R))
E1BCH5	LATS1	bta:535935; E1BCH5	ENSBTAG00000015076	0.005373172	-0.133874438	-0.139247611
Q2T9Y0	RSRC1	bta:509437; Q2T9Y0		0.043957746	-0.159702618	-0.203660364
A0AAA9TCS9	AP5Z1	A0AAA9TCS9	ENSBTAG00000012197	-0.148667381	-0.077167861	0.07149952
Q24K21	DPCD	bta:516908; Q24K21	ENSBTAG00000003578	-0.148010225	-0.117523303	0.030486922
A0AAA9THN5	HOMER3	A0AAA9THN5; bta:508124	ENSBTAG00000000813	-0.074254555	-0.168719239	-0.094464684
A0AAA9SV91	ZMIZ1	A0AAA9SV91	ENSBTAG00000019313	0.10704321	0.18478296	0.07773975
A0AAA9RXP7	UQCRQ	A0AAA9RXP7	ENSBTAG00000009479	0.052159776	-0.021695071	-0.073854847
A0A3Q1LFT9	HYCC1	A0A3Q1LFT9	ENSBTAG00000004575	-0.021613813	-0.092751903	-0.07113809
G3N1P6	ANAPC2	G3N1P6	ENSBTAG00000046223	0.024736678	0.018954326	-0.005782353
F6Q1G9	RAB24	F6Q1G9	ENSBTAG00000025425	0.058270605	-0.021394782	-0.079665387
F1MQK6	UBR3	F1MQK6	ENSBTAG00000020296	-0.046064872	-0.046064872	0
A7YSY2	AFG2B	A7YSY2; bta:533070	ENSBTAG00000018279	0.190698383	0.165942284	-0.024756099
A0AAA9TRS1	RIOK3	A0AAA9TRS1	ENSBTAG00000010042	0.158531497	0	-0.158531497
A0AAA9SXL2	LAMC3	A0AAA9SXL2	ENSBTAG00000017473	-0.04875463	-0.176682368	-0.127927737
Q2KIJ2	METTL18	bta:783955; Q2KIJ2		-0.223477889	-0.221958463	0.001519426
A7MBG2	ABHD17B	A7MBG2; bta:781153	ENSBTAG00000006816	-0.064396665	-0.014721506	0.049675159
A0A3Q1M7N0	NUBP2	A0A3Q1M7N0	ENSBTAG00000016561	-0.032580654	-0.051326581	-0.018745926
A0A3Q1N0F9	RAPGEF2	A0A3Q1N0F9	ENSBTAG00000012450	-0.040250957	-0.051555188	-0.011304231
A0A3S5ZPS3	ACOT4	A0A3S5ZPS3; bta:511431	ENSBTAG00000002843	0.057214736	-0.016509087	-0.073723823
Q0P5J1	FAR2	bta:534380; Q0P5J1	ENSBTAG00000011095	-0.726610852	-0.957627056	-0.231016204
A0AAA9SB62	CHST12	A0AAA9SB62	ENSBTAG00000020898	-0.024023229	-0.038903479	-0.01488025
E1B770	LPCAT2	E1B770	ENSBTAG00000019272	-0.176506022	-0.276534841	-0.100028818
A0AAA9SLH3	ISCU	A0AAA9SLH3	ENSBTAG00000011787	0.212934911	0.029686131	-0.18324878
F1N1Q7	PREX1	F1N1Q7	ENSBTAG00000015609	0.0890433	0.294791965	0.205748665
A0AAA9T4H0	TIMM17A	A0AAA9T4H0	ENSBTAG00000047059	-0.216778815	-0.549250404	-0.332471589
A0A3Q1LUT0	SLC39A14	A0A3Q1LUT0	ENSBTAG00000019225	0.280107919	0.019011969	-0.261095951
A0AAA9T180	CYB5B	A0AAA9T180	ENSBTAG00000002412	0.1023208	0.152898899	0.050578099
A0AAF6DLG6				-0.272578179	-0.115477217	0.157100961
A7YWK4	MED19	A7YWK4; bta:509245; F1MQL1	ENSBTAG00000002389	0.152449679	0.045431429	-0.10701825
A7Z060	OBI1	A7Z060; bta:782050	ENSBTAG00000000869	0.228676796	-0.066897037	-0.295573833
A0A3S5ZPC1	SYPL1	A0A3S5ZPC1	ENSBTAG00000019794	-0.441199986	-0.344050906	0.097149081
A0AAA9TQC8	RPL22L1	A0AAA9TQC8	ENSBTAG00000007394	-0.393467189	-0.696579034	-0.303111845
Q2HJE9	TIMM17B	bta:540765; Q2HJE9		0.035522695	-0.030756292	-0.066278987
F1MI02	FCHSD2	F1MI02	ENSBTAG00000019062	-0.018736563	0.067537212	0.086273774
A0AAA9TGR7	CMIP	A0AAA9TGR7	ENSBTAG00000013524	-0.044925871	-0.109534952	-0.064609081
E1BKC3	TNFAIP3	bta:508105; E1BKC3	ENSBTAG00000000436	0.144161905	0.303942349	0.159780444
Q01321	NDUFA4	bta:327704; Q01321; Q3ZC70	ENSBTAG00000011145	0.175048993	0.033916635	-0.141132358
Q3ZC25	TMEM106B	bta:508903; Q3ZC25	ENSBTAG00000019750	-0.043118234	0.067039928	0.110158161
A0A3Q1LUU0	INPP5B	A0A3Q1LUU0	ENSBTAG00000024107	-0.128007612	0.01504388	0.143051493
A0A3Q1ME94	BANF1	A0A3Q1ME94	ENSBTAG00000031875	-0.289838195	0.095252459	0.385090654
Q32LE1	YEATS4	bta:615330; Q32LE1	ENSBTAG00000002256	0.277755912	0.08894177	-0.188814141
A0AAA9TH35	COPS8	A0AAA9TH35	ENSBTAG00000005854	-0.007275331	0.045712974	0.052988305
A0AAA9SG08	DYNLRB1	A0AAA9SG08	ENSBTAG00000006134	0.249335861	0.31177874	0.062442879
A0A3Q1MDC6	NCSTN	A0A3Q1MDC6	ENSBTAG00000004334	0	0.08590905	0.08590905
A5PKM1	ZNRF2	A5PKM1; bta:615645	ENSBTAG00000000260	0.09073655	-0.069204363	-0.159940913
Q2YDG6	NUP42	bta:515127; F6QMH3; Q2YDG6	ENSBTAG00000016014	0.289643515	0.081960091	-0.207683423
Q3T0J1	FBXO4	bta:534852; Q3T0J1	ENSBTAG00000016981	-0.64291397	-0.458929025	0.183984945
Q0VFX9	GGACT	bta:512596; Q0VFX9		0.09169569	0.090369074	-0.001326616
E1BB53	TEP1	E1BB53	ENSBTAG00000001179	0.168361782	0.029230711	-0.139131071
A7MB12	UTP15	A7MB12; bta:526343	ENSBTAG00000006843	-0.188120534	-0.162331852	0.025788681
Q08DC9	CEND1	bta:504370; Q08DC9	ENSBTAG00000001228	0.129283017	0.246408087	0.11712507
A0AAF6DLK5	PIGG	A0AAF6DLK5	ENSBTAG00000005964	0.466393635	0.238562508	-0.227831127
A0AAF6Z202	GAPDHS	A0AAF6Z202	ENSBTAG00000015917	0.249139964	0.165308373	-0.083831591
A6QPI6	TOMM22	A6QPI6; bta:510780	ENSBTAG00000010157	0.109316694	0.236146717	0.126830022
A0AAA9SYS8	GATB	A0AAA9SYS8	ENSBTAG00000013914	-0.138279375	-0.08246216	0.055817215
F1N230	ORC2	F1N230	ENSBTAG00000031473	-0.215686727	-0.173996044	0.041690683
A0A3Q1MR59	ELL	A0A3Q1MR59	ENSBTAG00000003587	-0.129079949	0.003893916	0.132973865
A0AAA9T2P8	C1GALT1	A0AAA9T2P8	ENSBTAG00000004995	0.257076951	-0.05246742	-0.30954437
Q0NXR6	ACAD8	A1A4N5; bta:512070; Q0NXR6	ENSBTAG00000012937	-0.485926981	-0.259538172	0.226388809
A0A3Q1LKV3	RBM23	A0A3Q1LKV3	ENSBTAG00000038333	0.483049084	-0.277240032	-0.760289117
Q8HXG5	NDUFB11	bta:404161; Q3T137; Q8HXG5	ENSBTAG00000068665	-0.128662725	0.014486334	0.143149059
Q08DH2	TPST1	bta:513004; Q08DH2	ENSBTAG00000000390	0.304365532	0.154055292	-0.150310241
A7Z024	RP2	A7Z024	ENSBTAG00000005496	0.15700497	0.153902399	-0.003102571
F1N783	CHSY3	F1N783	ENSBTAG00000014661	-0.355250777	-0.362410569	-0.007159792
P68401	PAFAH1B2P68402	bta:282514; O00687; P68401; Q29459; Q3ZBB8	ENSBTAG00000005627	0.091303005	0.163661605	0.0723586
Q56JX5	RPS25	bta:282052; Q56JX5	ENSBTAG00000013866; ENSBTAG00000027772	-0.068461879	-0.186413124	-0.117951245
Q1KLV9	TRAF6	Q1KLV9		0.03391408	-0.102193513	-0.136107592
Q2HJ74	GATM	bta:414732; Q2HJ74	ENSBTAG00000005586	-0.130331868	-0.131644006	-0.001312137
A0A3Q1MBF5	MRPS2	A0A3Q1MBF5	ENSBTAG00000005688	-0.280364284	-0.273118187	0.007246098
A0A3Q1M319	AMOTL1	A0A3Q1M319	ENSBTAG00000018945	-0.113398327	-0.389855905	-0.276457578
A0A3Q1M4H2	ADAM9	A0A3Q1M4H2	ENSBTAG00000017455	0.148188554	-0.177625965	-0.325814518
F1MFD9	OSBPL10	F1MFD9	ENSBTAG00000009194	0.029877041	0.071296969	0.041419927
F6RC12	FEZ1	F6RC12	ENSBTAG00000009124	0.648246804	-0.670221589	-1.318468393
A0A3Q1LPS2	R3HDM1	A0A3Q1LPS2	ENSBTAG00000013302	0.044947406	-0.150650989	-0.195598395
F2Z4I9	PAM16	F2Z4I9	ENSBTAG00000009200	0.15065415	0.038434723	-0.112219427
A0AAA9SNL2	MPHOSPH8	A0AAA9SNL2	ENSBTAG00000003922	-0.187693519	-0.11717433	0.070519189
A0AAF7A0S6	KIAA1191	A0AAF7A0S6	ENSBTAG00000011608	-0.074676686	0.064192379	0.138869066
A0AAA9TPR6	RWDD1	A0AAA9TPR6	ENSBTAG00000009689	0.073704005	0.033705937	-0.039998068
F1MPW9	C2CD3	F1MPW9	ENSBTAG00000002907	-0.039131245	-0.091489844	-0.052358599
A0A452DIG5	MGST3	A0A452DIG5	ENSBTAG00000010265	0.231325546	0.26176666	0.030441114
F1MC83	HAX1	F1MC83	ENSBTAG00000013025	0.291005521	0.168257724	-0.122747797
A0AAA9RSU6	WDTC1	A0AAA9RSU6	ENSBTAG00000053588	-0.305584469	-0.069893215	0.235691253
A0A3Q1MWL7	DENND3	A0A3Q1MWL7	ENSBTAG00000006132	-0.003476375	-0.161486266	-0.158009891
F6QND5	FGA	F6QND5	ENSBTAG00000001638	-0.602904671	-0.401056745	0.201847927
A0AAF7AMQ9	S100A2	A0AAF7AMQ9	ENSBTAG00000037651	-0.027201728	0.115477217	0.142678945
A0AAF6Z0W9	FBXO9	A0AAF6Z0W9	ENSBTAG00000015083	-0.008259727	-0.145763251	-0.137503524
A4FUX6	MCC	A4FUX6; bta:616704; E1BC92	ENSBTAG00000035858	-0.01841462	-0.097526349	-0.079111729
A0A452DJ92	ZFYVE26	A0A452DJ92	ENSBTAG00000014334	-0.217401177	-0.048533482	0.168867695
P0C2B6	MRPL51	A7MB58; bta:513622; P0C2B6	ENSBTAG00000014729	-0.137503524	-0.130235523	0.007268

Accession	Gene_Symbol	Gene_ID	Ensembl_Gene ID	log2(ratio(CM12H/CM0H))	log2(ratio(CM12R/CM0H))	log2(ratio(CM12H/CM12R))
E1BFT9	ANKS3	A0A8J8XSB4; bta:528545; E1BFT9; M5FMW8	ENSBTAG00000006593	-0.032838613	-0.022764891	0.010073722
Q3SYW5	AZI2	bta:767932; Q3SYW5	ENSBTAG00000004117	-0.054649025	-0.161773239	-0.107124214
A0AAA9RZM4	ANTXR2	A0AAA9RZM4	ENSBTAG00000014324	0.08191913	0.141828119	0.059908989
F1MCE2	TBCEL	bta:540781; F1MCE2	ENSBTAG00000018213	0.016713874	0.084583209	0.067869336
Q32LN1	UBE2H	bta:539313; Q32LN1	ENSBTAG00000000911	0.459772238	0.352332971	-0.107439268
A0AAA9RXC3	RRM2B	A0AAA9RXC3	ENSBTAG00000021208	0.267969111	0.252047116	-0.015921995
A0A3Q1M534	RBSN	A0A3Q1M534	ENSBTAG00000013725	-0.114863957	-0.160753356	-0.045889399
E1BIU5	FBXO42	bta:100848477; E1BIU5	ENSBTAG00000008510	-0.131789873	0.078002512	0.209792385
E1BNQ3	RAP2A	E1BNQ3	ENSBTAG00000020412	0.138560831	0.10514815	-0.03341268
E1BBM0	MRM3	bta:538817; E1BBM0	ENSBTAG00000019162	-0.176298546	-0.126522312	0.049776235
A0A452DIY2	MTIF3	A0A452DIY2	ENSBTAG00000020530	0.003939998	-0.007912424	-0.011852422
F1MIQ3	LIN9	F1MIQ3	ENSBTAG00000033267	0.159995884	-0.191797182	-0.351793066
A0A3Q1MKP5	PMS1	A0A3Q1MKP5	ENSBTAG00000018795	-0.089157519	-0.05256301	0.036594509
A0AAA9TUI5	NME6	A0AAA9TUI5	ENSBTAG00000003025	-0.178198307	-0.08285043	0.095347877
A0A3Q1MWF7				0.028569152	-0.007231569	-0.035800721
A0A452DIT0				0.00329007	0.148540426	0.145250356
P23935	NDUFA5	bta:327714; P23935; Q32P63	ENSBTAG00000009334	-0.193603998	-0.458174003	-0.264570005
A0AAA9RU55	FGB	A0AAA9RU55	ENSBTAG00000022120	-0.837479035	-0.716905371	0.120573663
Q3ZBM4	ITGB1BP1	bta:513531; Q3ZBM4	ENSBTAG00000013063	-0.052210799	-0.043614776	0.008596024
Q2TBR9	LLPH	bta:510518; Q2TBR9	ENSBTAG00000002004	-0.272262246	-0.316793406	-0.04453116
A0AAF6Z072	TNFAIP8	A0AAF6Z072	ENSBTAG00000014551	0.239575433	0.160170715	-0.079404718
F6QQE8	CYP27A1	F6QQE8	ENSBTAG00000013489	-0.031267061	0.069020996	0.100288057
A0AAA9T2U9	THNSL1	A0AAA9T2U9; bta:788561	ENSBTAG00000009117	-0.033623652	-0.006055392	0.02756826
A0A3Q1MDV1	SUFU	A0A3Q1MDV1	ENSBTAG00000021068	0.162396457	0.036268754	-0.126127703
Q32LH7	CYB5R4	bta:533608; Q32LH7	ENSBTAG00000013591	0.197897859	0.187982391	-0.009915469
F1MD46	RB1	F1MD46	ENSBTAG00000006640	-0.00737953	0.261807102	0.269186633
Q95142	PDE6D	bta:281976; Q95142	ENSBTAG00000019480	0.078597596	0.116346872	0.037749277
A7E303	COL8A1	A7E303; bta:538564	ENSBTAG00000013662	-0.158103293	-0.137868809	0.020234484
A0A3Q1LWA8	MYO9A	A0A3Q1LWA8	ENSBTAG00000007433	-0.030557467	-0.107579887	-0.07702242
G3MZ59	TTC9	G3MZ59	ENSBTAG00000045604	0.045160899	0.009460329	-0.035700569
E1BL00	PWP2	E1BL00	ENSBTAG00000007105	-0.157995757	-0.243994676	-0.085998919
E1BIZ2	SSH1	E1BIZ2	ENSBTAG00000018825	-0.118153478	-0.143244459	-0.025090981
A0AAA9SS15	BRAF	A0AAA9SS15	ENSBTAG00000021761	-0.057353171	0.011789198	0.069142369
Q17QJ6	BCL10	bta:540824; Q17QJ6	ENSBTAG00000013851	-0.116899292	-0.17990909	-0.063009798
F1MUX1	MBD4	F1MUX1	ENSBTAG00000021645	-0.06997953	-0.012348392	0.057631137
F1N5L6	DSC3	F1N5L6	ENSBTAG00000015238	-0.378400308	-0.44049308	-0.062092771
A0AAF6YIA2	HACE1	A0AAF6YIA2	ENSBTAG00000001533	-0.299979122	-0.035828079	0.264151043
F1MG06	RPTOR	F1MG06	ENSBTAG00000002883	-0.006061753	0.012047628	0.018109381
F1MRF7	GAR1	F1MRF7	ENSBTAG00000000748	-0.21717963	-0.119228465	0.097951165
F6QB70	TREX1	F6QB70	ENSBTAG00000008406	-0.048318024	0.126300642	0.174618666
Q3SZB0	B3GAT3	bta:404162; F1N6Z1; Q3SZB0		0.008647549	0.011518584	0.002871036
A0AAA9S5U9	POGLUT3	A0AAA9S5U9	ENSBTAG00000008709	-0.035721922	-0.207657649	-0.171935727
A0A3Q1LYQ7	SRP9	A0A3Q1LYQ7	ENSBTAG00000033322	0.101131134	-0.06583199	-0.166963124
A0A3Q1LHP2	SCHIP1	A0A3Q1LHP2	ENSBTAG00000014960	-0.109377018	-0.349463091	-0.240086073
A0A3Q1MPM1	NEU1	A0A3Q1MPM1	ENSBTAG00000005674	0.08685839	0.010237109	-0.076621282
A0A3Q1M8A8	TDRD3	A0A3Q1M8A8	ENSBTAG00000009308	-0.087724476	-0.018253319	0.069471156
Q3ZCE0	LSM8	bta:613630; Q3ZCE0		0.055730431	-0.032232507	-0.087962938
A3KN19	FAM83D	A3KN19; bta:508561		0.177633693	0.268317627	0.090683933
Q32LC1	RPP38	bta:511723; G5E591; Q32LC1	ENSBTAG00000063884	-0.119862132	0.418583077	0.538445209
O77480	MTFMT	bta:286855; O77480	ENSBTAG00000021630	-0.147153694	-0.068641666	0.078512028
E1BDJ7	DNAJC25	bta:535430; E1BDJ7	ENSBTAG00000030960	0.231904508	0.08419835	-0.147706158
A0AAA9SDA7	GRK6	A0AAA9SDA7	ENSBTAG00000043994	0.011091615	-0.003184759	-0.014276373
A0AAF6Z2U9	C3H1orf52	A0AAF6Z2U9	ENSBTAG00000016326	0.353636955	0.103093493	-0.250543462
A0AAA9T279	APTX	A0AAA9T279	ENSBTAG00000013947	-0.125030381	-0.040484665	0.084545715
A0A3Q1M591	RAB43	A0A3Q1M591	ENSBTAG00000053386	-0.142681107	-0.041950475	0.100730632
A0AAA9SL03	POU2F1	A0AAA9SL03	ENSBTAG00000024534	0.076519021	-0.040599621	-0.117118642
A0AAA9SMD9	TRIM59	A0AAA9SMD9	ENSBTAG00000058033	-0.297583125	-0.434655629	-0.137072504
F1MP23	FZD6	F1MP23	ENSBTAG00000010529	-0.237549434	-0.038602839	0.198946595
A0A3Q1MDF2	REEP4	A0A3Q1MDF2	ENSBTAG00000009504	-0.029274922	-0.010115344	0.019159578
F1MW97	HGH1	F1MW97	ENSBTAG00000039705	-0.140372656	-0.078533403	0.061839254
A0AAA9TS87	DHDH	A0AAA9TS87	ENSBTAG00000013337	0.162877137	0.279549493	0.116672356
Q32KX8	METTL26	bta:514636; Q32KX8		0.010847382	-0.00545443	-0.016301812
E1B7A6	FLVCR1	bta:533317; E1B7A6	ENSBTAG00000015974	-0.757169165	-0.851100852	-0.093931687
A0A3Q1LYV4	TPD52L1	A0A3Q1LYV4	ENSBTAG00000003336	-0.205087057	-0.260671268	-0.055584212
F1N6M9	DVL1	bta:523325; F1N6M9	ENSBTAG00000010696	0.099209604	0.099209604	0
E1BNB5	FOSL1	E1BNB5	ENSBTAG00000006194	-0.614767461	-0.727776423	-0.113008962
Q3ZBY7	DEGS1	bta:507290; Q3ZBY7	ENSBTAG00000012705	0.060871756	-0.054301384	-0.11517314
A0AAF6YK00	CIAO3	A0AAF6YK00	ENSBTAG00000002484	0.140147706	0.17351469	0.033366983
F1N7S0	TULP3	F1N7S0	ENSBTAG00000015879	0.250643261	0.297622188	0.046978928
A0AAA9SAG7	CMSS1	A0AAA9SAG7	ENSBTAG00000013663	0.025195315	0.183843503	0.158648188
A0A3Q1N6C7	CPSF4	A0A3Q1N6C7	ENSBTAG00000002090	-0.06593427	-0.0402171	0.02571717
Q2HJ95	ZDHHC6	bta:514158; Q2HJ95	ENSBTAG00000006713	0.228564652	0.525443258	0.296878606
F1MXA4	ADD2	F1MXA4	ENSBTAG00000009076	-0.214018729	-0.043068722	0.170950007
A0AAF6DMH1	RPP14	A0AAF6DMH1	ENSBTAG00000019987	0.091120851	-0.144808324	-0.235929176
Q2KI83	DNAJC17	bta:524795; Q2KI83	ENSBTAG00000013480	-0.258828295	-0.123617493	0.135210802
A0AAF6YPG3	DRAP1	A0AAF6YPG3	ENSBTAG00000006199	0.148176314	0.105891169	-0.042285145
F6R223	RFK	F6R223	ENSBTAG00000019345	0.187009543	-0.124764103	-0.311773646
A0A4Y5NDR9	AAMDC	A0A4Y5NDR9		0.01036317	0.118331666	0.107968497
A0JN40	KIF3C	A0JN40; bta:777770	ENSBTAG00000019138	0.060942764	-0.012873081	-0.073815846
E1B7F6	PLXNB1	E1B7F6	ENSBTAG00000013407	0.132384553	0.137503524	0.005118971
A2VE20	UBE2G1	A2VE20; bta:613676	ENSBTAG00000014433	0.1782305	0.081782045	-0.096448455
A0AAF7A1V8	FOXP1	A0AAF7A1V8	ENSBTAG00000016533	-0.007702608	-0.165897535	-0.158194927
A5PK70	NME3	A0A8J8YPU5; A5PK70; bta:515663; M5FKE7	ENSBTAG00000016552	0.12171113	-0.060309514	-0.182020644
A0AAF6DLM2	SLX9	A0AAF6DLM2	ENSBTAG00000017010	-0.434707479	-0.415037499	0.01966998
A7MBI8	NUDT9	A7MBI8; bta:517589	ENSBTAG00000011354	0.242127384	0.353918209	0.111790825
F6RW79	USP11	F6RW79	ENSBTAG00000016772	-0.277406523	-0.065979661	0.211426863
Q2KI48	PTTG1IP	bta:617245; Q2KI48	ENSBTAG00000021771	-0.012558904	-0.007836494	0.00472241
A0AAF6Z117	S100A10	A0AAF6Z117	ENSBTAG00000015147	-0.052907266	0.324300949	0.377208215
A0AAF6ZUW3	NFYC	A0AAF6ZUW3	ENSBTAG00000021943	0.02387305	0.10817555	0.0843025

Accession	Gene_Symbol	Gene_ID	Ensembl_Gene ID	log2(ratio(CM12H/CM0H))	log2(ratio(CM12R/CM0H))	log2(ratio(CM12H/CM12R))
A0AAA9S8D8	PLSCR2	A0AAA9S8D8	ENSBTAG00000022227	0.038567656	0.392655806	0.354088149
A2VE26	EXTL3	A2VE26; bta:783970; F1MYW6	ENSBTAG00000008844	-0.146238071	0.006484034	0.152722104
F1MWT0	MAN2C1	F1MWT0	ENSBTAG00000007368	-0.256410684	-0.206942008	0.049468676
A0A3Q1MB37	ACSS2	A0A3Q1MB37	ENSBTAG00000013303	-0.034023582	0.07183732	0.105860902
A6QR70	WRNIP1	A6QR70; bta:788824; F1MGL0	ENSBTAG00000006166	-0.288464154	-0.252880185	0.035583969
Q4JIJ3	MTR	bta:280869; Q4JIJ3		-0.216118188	0.120113131	0.336231318
A0AAF6DME0	AP4B1	A0AAF6DME0	ENSBTAG00000003614	-0.368960349	-0.247107288	0.121853062
A0A3Q1NCP3	SHROOM3	A0A3Q1NCP3; bta:100139141	ENSBTAG00000019633	-0.398611951	-0.366311459	0.032300492
A6H784	SCO2	A6H784; bta:100125923	ENSBTAG00000057697	-0.091029876	-0.058158731	0.032871146
A0A3Q1LF83	RYBP	A0A3Q1LF83	ENSBTAG00000022689	0.244202731	0.07427773	-0.169925001
A0AAA9RS78	PARL	A0AAA9RS78	ENSBTAG00000014460	0.031122517	-0.012035065	-0.043157582
Q0V7P2	PJA1	bta:786612; Q0V7P2	ENSBTAG00000021026	0.045514659	-0.201479735	-0.246994395
A0A3Q1MUV9	INHCA	A0A3Q1MUV9	ENSBTAG00000023411	-0.281801952	0.216737891	0.498539843
A0A3Q1N1I0	IQSEC2	A0A3Q1N1I0	ENSBTAG00000014945	-0.074078144	-0.049468676	0.024609467
A0AAF7AEL2	GPR108	A0AAF7AEL2	ENSBTAG00000017291	0.070510061	-0.02171428	-0.09222434
A0AAA9TVV3	CASP9	A0AAA9TVV3	ENSBTAG00000002472	0.152785254	0.049222516	-0.103562738
A7MB76	TRAPPC13	A7MB76; bta:511108		-0.04026387	0.014486334	0.054750204
A0A024QYS0	PTEN	A0A024QYS0; bta:540786		0.011019001	0.009450001	-0.001569
A0A3Q1LST8	SP3	A0A3Q1LST8	ENSBTAG00000000176	0.034181936	-0.002885391	-0.037067327
A4IFL0	TIMM23	A4IFL0; bta:509841	ENSBTAG00000011694	-0.211888295	-0.602709556	-0.390821262
F6Q6K1	CHEK1	F6Q6K1	ENSBTAG00000017582	0.112162273	-0.341499839	-0.453662113
A4IFF3	TTC9C	A4IFF3; bta:786577	ENSBTAG00000000773	0.171355537	0.056831223	-0.114524314
A1A4Q4	TMA7	A1A4Q4; bta:615251	ENSBTAG00000053755	0.332342432	0.087555993	-0.244786439
E1B9U5	FPGT	bta:100138313; E1B9U5	ENSBTAG00000038480	-0.611857728	-0.404502218	0.20735551
A0AAA9RYE8	SUMF2	A0AAA9RYE8	ENSBTAG00000008190	-0.187627003	-0.283792966	-0.096165963
A0AAA9RVN6	CIRBP	A0AAA9RVN6	ENSBTAG00000066789	-0.451485546	-0.39908166	0.052403886
A7E3T1	CD151	A7E3T1	ENSBTAG00000019569	-0.059802187	-0.100119642	-0.040317455
A0AAA9TRT2	GOLGA1	A0AAA9TRT2	ENSBTAG00000015286	0.212213918	0.067953028	-0.14426089
E1BH71	DTX3L	bta:515051; E1BH71	ENSBTAG00000009933	-0.173663187	-0.044840567	0.12882262
Q58CY6	PRXL2B	A4FUH8; bta:617001; Q58CY6		-0.084888898	-0.303497389	-0.218608492
A5D791	EIF2AK3	A5D791; bta:535820; F1MCN0	ENSBTAG00000000184	0.146116654	0.100111485	-0.046005169
F1MLY3	DCP1B	F1MLY3	ENSBTAG00000030285	0.118012913	0.201242586	0.083229674
A0AAA9SRY3	TENT4B	A0AAA9SRY3	ENSBTAG00000007942	-0.195256291	-0.16764406	0.027612231
Q2HJF6	SRA1	bta:780787; Q2HJF6	ENSBTAG00000001449	0.092675837	0.009677914	-0.082997923
A0AAA9SI99	VRK2	A0AAA9SI99	ENSBTAG00000033801	-0.196955207	-0.253692222	-0.056737015
A0AAA9TII1	SHROOM4	A0AAA9TII1; bta:519045	ENSBTAG00000002996	-0.18324878	-0.25864185	-0.07539307
F1MEQ5	DARS2	F1MEQ5	ENSBTAG00000004358	-0.058187414	0.00921853	0.067405944
F1N2J7				0.099960245	0.146738651	0.046778405
A0A3Q1LWS9	HMGN5	A0A3Q1LWS9	ENSBTAG00000001877	0.133531984	-0.0932097	-0.226741684
F1MC02	MGLL	F1MC02	ENSBTAG00000018248	-0.125835216	-0.047640486	0.07819473
F1MUR7	RETREG2	bta:507475; F1MUR7	ENSBTAG00000012620	0.052863275	0.128694688	0.075831413
F1N588	SMPD2	F1N588	ENSBTAG00000008145	0.0832003	0.156573487	0.073373186
A0A3Q1MDD9				0.167265655	0.025674922	-0.141590733
A0A3Q1M222	RBM42	A0A3Q1M222	ENSBTAG00000013440	0.166344532	0.129722735	-0.036621797
Q3MHJ2	MAEA	A7E3P9; bta:511956; Q3MHJ2	ENSBTAG00000012575	-0.1581227	-0.153530563	0.004592137
A0A3Q1LM54	KMT2D	A0A3Q1LM54	ENSBTAG00000014429	0.046006575	-0.218286178	-0.264292753
Q9TTA5	SMARCAL1	bta:338072; F1MDP3; Q9TTA5		-0.086226884	-0.081388327	0.004838557
A0AAA9TKN6	UTP23	A0AAA9TKN6	ENSBTAG00000040422	0.225465255	0.155014535	-0.07045072
A0A452DI94	PTGS1	A0A452DI94	ENSBTAG00000006716	0.128474558	0.400377193	0.271902635
A0AAA9TFU8	EDRF1	A0AAA9TFU8	ENSBTAG00000009439	0.13737753	-0.059413486	-0.196791015
Q32KN2	BNIP3	bta:615342; Q32KN2		0.532565665	0.092275373	-0.440290291
Q32LJ6	RABL3	bta:505510; Q32LJ6	ENSBTAG00000015898	0.09682384	-0.136923079	-0.233746919
A0A3Q1N5M8				-0.045156181	-0.189779744	-0.144623563
Q3SYY7	OCIAD2	bta:505877; Q3SYY7	ENSBTAG00000001839	0.165364732	0.335149727	0.169784995
F1N599	ELF2	F1N599	ENSBTAG00000020594	-0.140942608	-0.113656782	0.027285826
A0AAA9TCX8	PARD6B	A0AAA9TCX8	ENSBTAG00000021601	0.201047519	0.220464973	0.019417454
Q2TBK4	HAUS1	bta:528378; Q2TBK4	ENSBTAG00000002512	0.046105081	-0.092466513	-0.138571593
A0AAA9SKU5				0.150467495	0.120976198	-0.029491297
A0A3Q1MBS8	TRMU	A0A3Q1MBS8; bta:616451	ENSBTAG00000005595	-0.462575888	-0.40599236	0.056583528
E1B955	SLC30A5	bta:508169; E1B955	ENSBTAG00000001013	-0.01509109	-0.048857834	-0.033766744
F1MSI0	RANBP10	F1MSI0	ENSBTAG00000032763	-0.007020428	-0.038322731	-0.031302304
Q0VD50	NMNAT1	bta:522863; Q0VD50	ENSBTAG00000051897	-0.01165036	-0.080870409	-0.069220049
E1BH22	H2AC18	bta:614974; E1BH22; F2Z4I6	ENSBTAG00000032456; ENSBTAG00000038476	-0.154277396	0.178812805	0.333090201
A0A3Q1NHF7	MBNL2	A0A3Q1NHF7	ENSBTAG00000018313	-0.02181222	0.033623652	0.055435872
G3X6Y2	CXHXorf38	G3X6Y2	ENSBTAG00000011788	0.155059652	0.091865825	-0.063193826
A2VE19	PLEKHF1	A2VE19; bta:782309	ENSBTAG00000008625	0.170374789	0.198873396	0.028498606
A0AAA9S828	ABHD2	A0AAA9S828	ENSBTAG00000019954	0.12350206	-0.2504386	-0.37394066
A0A3Q1LKH0	PHF8	A0A3Q1LKH0	ENSBTAG00000013289	-0.074546953	0.029353913	0.103900866
A0AAA9TYI9				-0.038509563	0.149248731	0.187758295
F6RDN5	CHMP4C	F6RDN5	ENSBTAG00000004792	0.132486095	0.092854308	-0.039631787
A0AAA9SQ47	SFXN2	A0AAA9SQ47	ENSBTAG00000004321	-0.179969634	-0.153869917	0.026099718
A0AAA9SFP0	POLE	A0AAA9SFP0; bta:785457	ENSBTAG00000000590	-0.320650808	-0.14506262	0.175588188
C4T8B4	CRP	bta:527553; C4T8B4	ENSBTAG00000062195	-0.327459201	-0.095860015	0.231599185
F1N5S7	PDF	F1N5S7	ENSBTAG00000001663	0.149353515	0.187304144	0.037950629
E1BHF2	KATNAL1	bta:537739; E1BHF2; W0GI54	ENSBTAG00000009340	-0.238191545	-0.249944727	-0.011753181
F1N1T6	FASTKD2	F1N1T6	ENSBTAG00000016193	-0.21793152	-0.222189354	-0.004257834
E1BPM4	CHD7	E1BPM4	ENSBTAG00000021841	-0.389864428	-0.343817061	0.046047368
A0A3Q1MB94	LOC518495	A0A3Q1MB94; bta:518495	ENSBTAG00000048740	-0.060055123	-0.021835801	0.038219322
E1BCE1	ATAD5	E1BCE1	ENSBTAG00000018383	0.115908968	-0.092735213	-0.208644181
A0AAA9T9T8	GATD1	A0AAA9T9T8	ENSBTAG00000010348	-0.210158714	-0.053637964	0.15652075
F1N4K5	FOXO3	bta:535530; F1N4K5	ENSBTAG00000011234	0.050465067	0.117989061	0.067523994
F1N1K9	TGS1	F1N1K9	ENSBTAG00000005898	0.066045335	0.014617096	-0.051428239
F1MRD5	PUS10	F1MRD5	ENSBTAG00000009694	-0.023978863	0.087151949	0.111130812
A6H713	LIPA	A6H713; bta:100125267; F1N110	ENSBTAG00000003636	0.120519344	0.174711444	0.0541921
A0AAF6YJE9	MEN1	A0AAF6YJE9	ENSBTAG00000002095	-0.034093199	-0.049860515	-0.015767316
Q24JY6	TMEM160	bta:508953; Q24JY6	ENSBTAG00000015579	0.052812191	-0.08961953	-0.142431721
O97725	NDUFA12	bta:281742; O97725; Q3T092	ENSBTAG00000067029	0.082223125	0.153579731	0.071356606
F1N3M4	PIK3AP1	F1N3M4	ENSBTAG00000019872	0.278394042	0.312899604	0.034505562
Q1RMX2	UBE2D2	bta:541003; Q1RMX2	ENSBTAG00000004161	-0.046840254	-0.021102693	0.025737561
A0AAA9SMR5	RFFL	A0AAA9SMR5	ENSBTAG00000013645	-0.076216403	0.115286851	0.191503254

Accession	Gene_Symbol	Gene_ID	Ensembl_Gene ID	log2(ratio(CM12H/CM0H))	log2(ratio(CM12R/CM0H))	log2(ratio(CM12H/CM12R))
A0AAA9S8T7				0.081577589	-0.010164979	-0.091742569
F6QQ46	C10H15orf48	F6QQ46	ENSBTAG00000004558	0.184341774	0.197036847	0.012695073
Q28029	ATP6V1F	bta:282405; Q28029; Q2NKR2	ENSBTAG00000007572	0.081711343	0.276482722	0.194771379
Q0D253	NDNL2	bta:617494; Q0D253		-0.036680663	-0.093511886	-0.056831223
A0AAA9T6A1	SEC11C	A0AAA9T6A1	ENSBTAG00000011954	0	-0.059501012	-0.059501012
A0AAA9TU86	TP53RK	A0AAA9TU86	ENSBTAG00000027326	0.180309484	0.122674428	-0.057635055
Q5EA49	MIS12	bta:767858; Q5EA49	ENSBTAG00000020432	0.174515794	0.100581759	-0.073934035
A1L4Z7	OLR1	A1L4Z7		0.292987628	0.599018096	0.306030468
Q3ZBN4	CUEDC2	bta:516091; Q3ZBN4	ENSBTAG00000002501	0.470330335	-0.688901541	-1.159231875
F6RM59	FIBP	F6RM59	ENSBTAG00000020775	0.010237109	0.110161335	0.099924226
A0AAF6YZH3	ACYP1	A0AAF6YZH3	ENSBTAG00000013992	0.290121511	0.432270187	0.142148676
Q148K5	PEX11B	bta:506527; Q148K5		0.056048746	0.143948516	0.08789977
G5E526	PTP4A1	bta:613326; G5E526	ENSBTAG00000002275	-0.014541326	0.314468006	0.329009332
A0AAA9SF51	PLEKHG1	A0AAA9SF51	ENSBTAG00000009679	-0.125992766	-0.120459893	0.005532874
A0AAA9T663	ATG7	A0AAA9T663	ENSBTAG00000035827	-0.218149565	0.042848163	0.260997728
Q1RMW1	UBE2F	bta:617083; Q1RMW1		0.078900828	0.04344529	-0.035455538
A0AAA9TLV8	DCTN6	A0AAA9TLV8	ENSBTAG00000000744	0.175044167	0.054061734	-0.120982433
G3X686	SELENOO	G3X686	ENSBTAG00000000647	-0.305300133	-0.087147326	0.218152807
A0AAA9SKL6	DTD2	A0AAA9SKL6; bta:514035	ENSBTAG00000068336	0.004232849	0.014061466	0.009828617
E1BE06	AFTPH	E1BE06	ENSBTAG00000009299	0.172005312	0.146841388	-0.025163924
A0A3Q1MDF9	ACTR8	A0A3Q1MDF9	ENSBTAG00000011180	-0.388026547	-0.195187571	0.192838976
A0AAA9S1J3	LTF	A0AAA9S1J3	ENSBTAG00000001292	-0.425209304	-0.434311511	-0.009102207
F6QT33	RBM34	F6QT33	ENSBTAG00000000221	-0.279087144	-0.124340048	0.154747096
A0AAF6ZVL1	HSDL1	A0AAF6ZVL1	ENSBTAG00000018770	0.044142555	0.090097189	0.045954633
A0A3Q1M883	AGFG2	A0A3Q1M883	ENSBTAG00000000713	0.191130437	0.372794275	0.181663838
Q0P5L7	GLMP	bta:100125876; Q0P5L7	ENSBTAG00000006364	0.1443169	0.229166163	0.084849263
A0AAA9S448	B2M	A0AAA9S448	ENSBTAG00000048782	0.055655698	0.05292332	-0.002732378
Q3MHR3	DYNLL2	bta:540369; Q3MHR3	ENSBTAG00000025313	0.133447274	0.221733807	0.088286532
Q3SZ34	UBL4A	bta:504533; Q3SZ34		-0.051874439	0.031677212	0.083551651
A7E3R8	TMEM109	A7E3R8		-0.078284757	-0.250390197	-0.17210544
Q3ZCI1	TMEM14C	bta:613802; Q3ZCI1		0.292368297	0.149525203	-0.142843094
G3MZM8	VKORC1L1	G3MZM8	ENSBTAG00000014448	0.194596864	0.149918274	-0.04467859
Q0VCU0	PCBP4	bta:506889; Q0VCU0		-0.241225258	0.125729916	0.366955174
F1N430	TIMP2	F1N430	ENSBTAG00000010899	0.169769815	-0.162437701	-0.332207516
Q0VD44	TWSG1	bta:537290; F1N2H5; Q0VD44	ENSBTAG00000001805	0.317311467	0.024375575	-0.292935892
A0A452DI42	DCAKD	A0A452DI42	ENSBTAG00000006048	0.179024912	0.067412921	-0.111611991
A0A3Q1N2S6	PEF1	A0A3Q1N2S6	ENSBTAG00000010378	-0.040560363	0.099345206	0.13990557
F1MN80	WTAP	F1MN80	ENSBTAG00000007974	0.06157147	-0.128642151	-0.190213621
F6RE68	UBL7	F6RE68	ENSBTAG00000000010	0.106359873	0.143427199	0.037067327
Q9XSN4	GAPDL17	Q9XSN4		-0.059413486	0.012419259	0.071832745
Q3T0K8	EMC4	bta:523162; Q3T0K8	ENSBTAG00000006416	0.078777113	-0.041820176	-0.120597289
A0A3Q1LRB4	FAM118A	A0A3Q1LRB4	ENSBTAG00000010590	-0.0662663	0.098563834	0.164830134
A0AAA9SFN2	IFT122	A0AAA9SFN2	ENSBTAG00000019121	-0.160289705	-0.192220194	-0.031930489
Q2HJG7	CHURC1	bta:616539; Q2HJG7		0.230612928	0.389284401	0.158671473
A0AAA9SDR2	DIP2C	A0AAA9SDR2	ENSBTAG00000006531	-0.066017173	0.08935952	0.155376693
A0A3Q1LLS6	USP40	A0A3Q1LLS6; bta:531139	ENSBTAG00000000149	-0.41654109	-0.302337367	0.114203723
F1MWN2	HCK	F1MWN2	ENSBTAG00000007932	0.053274793	0.248641424	0.195366631
A0A3Q1NH47	SP1	A0A3Q1NH47	ENSBTAG00000003021	-0.090636919	-0.155306346	-0.064669428
G3N260	EARS2	bta:100300732; G3N260	ENSBTAG00000045593	0.023108263	0.187589126	0.164480863
A0A385KL35				0.20559417	-0.087382551	-0.292976721
A0A3Q1LS59	EYA3	A0A3Q1LS59	ENSBTAG00000043989	-0.198269627	0.041499363	0.23976899
F1N173	PLXNA3	F1N173	ENSBTAG00000008552	0.107862234	0.133613228	0.025750994
A5PK72				-0.423895828	0.021515321	0.445411148
A0AAA9RUL0	STEEP1	A0AAA9RUL0	ENSBTAG00000047169	0.344325195	-0.141315857	-0.485641052
A1A4Q9	NUDT16	A1A4Q9		-0.451874267	-0.736067837	-0.28419357
A0AAA9T584	CSNK1G3	A0AAA9T584	ENSBTAG00000017550	0.008630305	-0.030619235	-0.03924954
A0A3Q1NL63	PIMREG	A0A3Q1NL63	ENSBTAG00000002981	-0.019031261	-0.001455063	0.017576198
A0A3Q1M6X0	GYG1	A0A3Q1M6X0	ENSBTAG00000001721	0.17872195	-0.129748411	-0.30847036
A0A3Q1LPH5				0.023571707	0.008363473	-0.015208234
Q0P5E8	CHRAC1	bta:510942; Q0P5E8	ENSBTAG00000020226	0.371968777	0.242764577	-0.129204201
A0AAA9S2T2	PPM1H	A0AAA9S2T2	ENSBTAG00000011857	0.130512139	0.116772082	-0.013740057
A0AAA9S0G1	CCNH	A0AAA9S0G1	ENSBTAG00000009566	-0.029039673	-0.026375415	0.002664258
A0JN42	TTI1	A0JN42; F6Q3H3	ENSBTAG00000004100	-0.011790918	0.580482078	0.592272996
A0AAA9U0X9	TPRKB	A0AAA9U0X9	ENSBTAG00000011176	-0.098905538	-0.013296823	0.085608716
A0AAF6YM64	H2AZ1	A0AAF6YM64	ENSBTAG00000004428	-0.346853927	-0.433194776	-0.086340849
Q1JQD9	L3MBTL2	bta:513297; Q0V8P8; Q1JQD9		-0.39317494	-0.340122794	0.053052146
A0AAA9TNQ6				-0.126465775	-0.256879262	-0.130413488
A0AAA9T8K2	SH3BP5L	A0AAA9T8K2	ENSBTAG00000002645	0.041955316	-0.041588324	-0.083543641
A0A3Q1NGV7	FGD1	A0A3Q1NGV7	ENSBTAG00000015860	0.192843455	0.140530309	-0.052313146
F6QF30	DUS2	F6QF30	ENSBTAG00000014644	0.017811276	-0.060120992	-0.077932269
A0A3Q1LGY5	WRAP53	A0A3Q1LGY5	ENSBTAG00000001071	-0.25569658	-0.167372913	0.088323666
A0A452DIN8	MRPL20	A0A452DIN8	ENSBTAG00000004872	-0.246283042	-0.210811467	0.035471574
F1N5P6	SLC7A1	bta:539465; F1N5P6	ENSBTAG00000018577	-0.121947534	0.0360873	0.158034834
A0AAA9TJW3	MIER1	A0AAA9TJW3	ENSBTAG00000005443	-0.039840265	0.008395921	0.048236186
F1MXZ2	SLC16A3	F1MXZ2	ENSBTAG00000017461	0.405279161	0.215389034	-0.189890128
A0AAF6YN19	HIRIP3	A0AAF6YN19	ENSBTAG00000005028	-0.297237586	-0.393927222	-0.096689636
A0A3Q1M7P7	CADM3	A0A3Q1M7P7	ENSBTAG00000003217	0.003072834	0.258412848	0.255340014
F1MHZ3	NPAT	bta:526583; F1MHZ3	ENSBTAG00000010286	0.111084111	-0.100350525	-0.211434636
A0AAF7AEI7	SNUPN	A0AAF7AEI7	ENSBTAG00000021293	-0.16797673	-0.16651725	0.001459479
Q2KHV2	PACC1	bta:616392; Q2KHV2	ENSBTAG00000000758	0.157362101	0.114810938	-0.042551163
A4FUY6	TFCP2	A4FUY6; bta:509448; F6R2A8	ENSBTAG00000019312	0.022195746	0.011140558	-0.011055189
A5PKH8	PGM2L1	A5PKH8; bta:515366	ENSBTAG00000000770	-0.011736108	0.009984089	0.021720197
A6QR35	SCAMP2	A6QR35; bta:534312	ENSBTAG00000005844	0.028854863	0.027555723	-0.00129914
A0AAA9S2Q2	VTI1A	A0AAA9S2Q2	ENSBTAG00000050055	0.034976138	-0.105554171	-0.140530309
E1B9G8	ARHGEF18	bta:522521; E1B9G8	ENSBTAG00000040507	0.130776354	0.04094102	-0.089835334
A3FPG8	GPAT4	A3FPG8; bta:511614		0.049412194	0.186032255	0.136620061
Q29RR7	VPS37A	bta:513985; Q29RR7	ENSBTAG00000010355	-0.080740847	0.081500635	0.162241482
A5PKE0	C16H1ORF55	A5PKE0; bta:534664		0.273600488	-0.248820547	-0.522421035
A6QNJ7	PGM5	A6QNJ7; bta:785045; F1MTN7	ENSBTAG00000033190	0.113858079	0.182784969	0.06892689
Q3SZK3	GHITM	bta:404143; Q3SZK3	ENSBTAG00000032829	-0.219189989	-0.187550672	0.031639317
A5PJA8	EMC7	A5PJA8; bta:615423		0.087628907	-0.042983171	-0.130612077

Accession	Gene_Symbol	Gene_ID	Ensembl_Gene ID	log2(ratio(CM12H/CM0H))	log2(ratio(CM12R/CM0H))	log2(ratio(CM12H/CM12R))
A0A3Q1M210	TPPP	A0A3Q1M210	ENSBTAG00000052247	0.117569596	0.098628033	-0.018941564
B0JYP6	IGK	B0JYP6		-0.259801614	-0.164644381	0.095157233
F1N463	ARID2	F1N463	ENSBTAG00000011087	-0.097825199	-0.084392187	0.013433012
A1L522	SMYD5	A1L522; bta:509313; F1MGV1		0.007502331	-0.058329659	-0.06583199
A0AAA9SM52	DMAC2	A0AAA9SM52	ENSBTAG00000009449	-0.087634274	-0.030490367	0.057143907
A0AAA9SWU6	PGGT1B	A0AAA9SWU6	ENSBTAG00000001895	-0.038298044	-0.112439507	-0.074141463
A0A3Q1LX15	BCLAF3	A0A3Q1LX15; bta:511003	ENSBTAG00000021962	0.022791865	-0.077066884	-0.099858749
Q08DJ3	FUBP1	bta:513562; F1MX51; Q08DJ3		0.593051321	0.293136438	-0.299914883
A0AAF6DME7	FTSJ1	A0AAF6DME7	ENSBTAG00000031829	-0.248565451	-0.248565451	0
A2VDT8	HPS6	A2VDT8; bta:511792	ENSBTAG00000021942	-0.093383447	-0.185421338	-0.092037891
A0A3Q1M2V9	TMEM11	A0A3Q1M2V9	ENSBTAG00000010566	-0.172717262	-0.228531558	-0.055814296
A0AAA9TDE1	GPN1	A0AAA9TDE1	ENSBTAG00000003904	-0.009407453	-0.027042873	-0.01763542
A0AAA9S464	KCTD5	A0AAA9S464	ENSBTAG00000040575	0.011997533	-0.186205423	-0.198202956
A0A3Q1LT12	GSTA4	A0A3Q1LT12	ENSBTAG00000004288	-0.106301073	-0.010686679	0.095614395
A0AAF6YUT0	THG1L	A0AAF6YUT0	ENSBTAG00000010359	-0.082132627	0.002798633	0.084931261
A0AAA9TU88	TGFBRAP1	A0AAA9TU88	ENSBTAG00000021731	0.108666685	0.011404763	-0.097261922
A0AAA9SIJ7	TIMP1	A0AAA9SIJ7	ENSBTAG00000066152	-0.23878686	-0.480341538	-0.241554678
F6REB9	NAA40	F6REB9	ENSBTAG00000003073	-0.463143756	-0.415743837	0.047399919
Q32KR9	CHMP1A	bta:789414; Q32KR9	ENSBTAG00000033331	0.154328146	-0.010216396	-0.164544542
A0A3Q1MLK8	OGFOD3	A0A3Q1MLK8; bta:506185	ENSBTAG00000018172	0.188617865	0.259643817	0.071025951
A0A3Q1MN55	TXNL4A	A0A3Q1MN55	ENSBTAG00000013587	0.191035825	0.188215306	-0.002820519
A0AAA9SW13	ENY2	A0AAA9SW13	ENSBTAG00000007387	-0.219445137	-0.434983888	-0.21553875
F2Z4F6	SNX7	F2Z4F6	ENSBTAG00000031461	0.06555663	0.053231681	-0.01232495
E1BGE6	FBXW8	bta:787118; E1BGE6	ENSBTAG00000036183	-0.248259053	-0.204819792	0.043439261
A0A452DKI2	PYROXD1	A0A452DKI2	ENSBTAG00000021078	-0.002953317	0.056413309	0.059366626
A0A3Q1M072	GORASP1	A0A3Q1M072	ENSBTAG00000047135	-0.053111336	0.301238236	0.354349573
Q5E9Q3	CCDC51	A6QQF9; bta:517515; Q5E9Q3; Q5E9W7; Q5EA69	ENSBTAG00000013413	0.171655059	0.125271194	-0.046383865
F1MXD0	SPOUT1	F1MXD0	ENSBTAG00000012485	0.218305464	-0.02739833	-0.245703794
Q3MHJ0				0.051241285	-0.004123952	-0.055365237
Q2KHV9				0.123936812	0.100809663	-0.023127148
F6Q3F4	ZNF277	F6Q3F4	ENSBTAG00000013050	-0.074723918	-0.301463992	-0.226740074
A0A3Q1N043	TMA16	A0A3Q1N043	ENSBTAG00000012658	-0.442027137	-0.352174382	0.089852755
F6R6Y8	PARP9	F6R6Y8	ENSBTAG00000021791	-0.041375928	0.129930304	0.171306232
P82928	MRPS28	bta:535290; P82928; Q2KI96	ENSBTAG00000001302	0.093642847	0.02398668	-0.069656166
A0A3Q1N8M5	INO80	A0A3Q1N8M5	ENSBTAG00000010380	0.079591821	-0.013914747	-0.093506568
F1MRA9	PAK1IP1	F1MRA9	ENSBTAG00000018674	0.042091333	-0.076333692	-0.118425025
Q2LGB7	TICAM2	bta:539350; Q2LGB7	ENSBTAG00000002357	-0.054774223	0.110245317	0.165019539
E1BDA1	BRMS1	E1BDA1	ENSBTAG00000017689	-0.145299553	0.005747797	0.15104735
A4FUC9	RHPN2	A4FUC9; bta:533687	ENSBTAG00000003089	0.228371274	0.192120748	-0.036250526
A0AAF6YTM4	C4H7orf25	A0AAF6YTM4	ENSBTAG00000009472	-0.218409985	-0.02986464	0.188545345
Q0P5G9	SPRYD4	bta:539560; Q0P5G9	ENSBTAG00000009281	-0.155111117	0.020443389	0.175554507
A0AAA9SZU0	LOC531462	A0AAA9SZU0	ENSBTAG00000010813	-0.073421069	-0.205286517	-0.131865448
A0A0A0MP99	CA1	A0A0A0MP99	ENSBTAG00000036116	-0.021100121	0.125312672	0.146412793
B2Z4B0	ANGPTL2	B2Z4B0; bta:512019	ENSBTAG00000011810	-0.145661567	-0.281197842	-0.135536276
F1MNN7	LBP	F1MNN7	ENSBTAG00000016864	-0.444813736	-0.565846835	-0.121033099
A0A3Q1N2Q7	GALNT12	A0A3Q1N2Q7	ENSBTAG00000009037	-0.955377009	-1.113904455	-0.158527446
A0AAA9TXB2	IQSEC1	A0AAA9TXB2	ENSBTAG00000003237	-0.443183805	-0.326500825	0.11668298
F1N1Q2	SLC6A17	bta:407174; F1N1Q2	ENSBTAG00000001981	0.329052529	0.303238212	-0.025814318
A0A3Q1MH47	FIS1	A0A3Q1MH47	ENSBTAG00000007900	0.047257287	0.005992511	-0.041264775
Q2TBR6	PFDN4	bta:514621; Q2TBR6	ENSBTAG00000008812	-0.29074339	-0.500338537	-0.209595147
A0A3Q1MBE1	LOC404103	A0A3Q1MBE1	ENSBTAG00000049782	-0.260062839	-0.133213223	0.126849616
F1MU27	MARCHF11	F1MU27	ENSBTAG00000037576	-0.372040759	-0.436819094	-0.064778335
A0AAA9SGI1	DVL3	A0AAA9SGI1	ENSBTAG00000019336	-0.010669743	-0.138566954	-0.127897211
A0AAA9S7E2	DHX33	A0AAA9S7E2	ENSBTAG00000032083	0.163439302	0.550724598	0.387285296
Q3T0N3	TMCO1	bta:614715; Q3T0N3	ENSBTAG00000010009	0.001492701	0.036861868	0.035369168
A0A3Q1MEE5	DIP2A	A0A3Q1MEE5	ENSBTAG00000011495	0.127933372	-0.418539188	-0.54647256
F1MB60	RPS26	F1MB60	ENSBTAG00000038896	-0.229388688	0.008082346	0.237471034
Q0IIB2	NFKBIB	bta:525408; F1N2I4; Q0IIB2		-0.160199771	-0.113976754	0.046223017
A0AAA9SB18	SIL1	A0AAA9SB18	ENSBTAG00000033747	-0.032994464	-0.163083628	-0.130089164
A0AAA9TR33	PDGFC	A0AAA9TR33; bta:613787	ENSBTAG00000043959	-0.129315499	-0.576154495	-0.446838996
A0AAA9TJG9	PECR	A0AAA9TJG9	ENSBTAG00000006821	-0.028170673	-0.051385386	-0.023214714
A0AAA9SST1	PRKCQ	A0AAA9SST1	ENSBTAG00000010664	-0.318753847	-0.282227971	0.036525876
A0AAA9TJ16	ALKBH8	A0AAA9TJ16	ENSBTAG00000010642	-0.582399531	-0.607194041	-0.02479451
A6QPR9	TEFM	A6QPR9		0.021194222	0.071405668	0.050211446
A0A3Q1NNB2	NRDE2	A0A3Q1NNB2	ENSBTAG00000005427	-0.046338715	-0.057800106	-0.011461391
A0A3Q1N5T1	NEDD9	A0A3Q1N5T1	ENSBTAG00000006287	-0.199208937	-0.168577318	0.030631619
A0AAF6YTK9	USP22	A0AAF6YTK9	ENSBTAG00000008978	-0.143801984	-0.277914919	-0.134112935
A0A3Q1MHV8	SEC14L1	A0A3Q1MHV8	ENSBTAG00000013677	-0.067498761	-0.471764587	-0.404265826
A0A3Q1M0E5	PARP16	A0A3Q1M0E5	ENSBTAG00000012352	-0.022414149	0.019147842	0.041561991
G3N348	WFS1	bta:100298456; G3N348	ENSBTAG00000046671	-0.00307611	0.144609381	0.147685491
E1BPQ4	HMGA2	bta:100297155; E1BPQ4	ENSBTAG00000044118	0.035068792	-0.094991748	-0.130060541
A7MB67	ZYG11B	A7MB67; bta:540744	ENSBTAG00000019680	0.058957033	0.068049679	0.009092646
A5D9D8	CYP3A4	A5D9D8; bta:507988		-0.37995504	0.087201696	0.467156736
A0AAA9S7W7	RGP1	A0AAA9S7W7	ENSBTAG00000011433	-0.158964755	-0.160389639	-0.001424884
A0AAA9S5L7	DSCC1	A0AAA9S5L7	ENSBTAG00000015338	-0.095774646	-0.10433666	-0.008562014
E1BPN1	WWTR1	E1BPN1	ENSBTAG00000007814	-0.081529885	-0.085262604	-0.003732719
A0A3Q1M5C7				-0.169145882	-0.086060729	0.083085153
Q0VCQ2	SLC27A4	bta:514427; Q0VCQ2	ENSBTAG00000015436	-0.056995366	0.095313675	0.152309041
A5PJN1	CCDC59	A5PJN1; bta:615189; Q32KL6	ENSBTAG00000036282	0.433307087	-0.127523752	-0.560830838
A0A3Q1NK96	TXNDC15	A0A3Q1NK96	ENSBTAG00000014744	0.175136428	0.208653554	0.033517126
A0AAA9SQX1				0.13901896	-0.11872392	-0.25774288
A5PKD2	GLTP	A5PKD2		0.183864192	0.254547079	0.070682887
F1MTZ3	UBASH3B	bta:523809; F1MTZ3	ENSBTAG00000000842	-0.269432177	-0.023598512	0.245833665
A5PK03	FRMD8	A5PK03; bta:515469		0.018450852	0.051906149	0.033455297
A0AAA9SGT2	ELP6	A0AAA9SGT2	ENSBTAG00000011478	-0.060787718	-0.068980217	-0.008192498
F1ME21	ZBTB38	bta:539843; F1ME21	ENSBTAG00000040061	-0.419713986	-0.527405459	-0.107691472
A0A3Q1LM07	KIF7	A0A3Q1LM07	ENSBTAG00000002440	-0.02549212	0.019124655	0.044616775
A0AAA9TNK4	SLC39A6	A0AAA9TNK4	ENSBTAG00000000998	0.180120769	-0.006152227	-0.186272996
A0AAA9SP61	NXT2	A0AAA9SP61	ENSBTAG00000020739	0.040445526	-0.001415101	-0.041860626
F1MV65	LOC100848991	bta:100848991; F1MV65	ENSBTAG00000030470	0.165216939	-0.211734451	-0.376951391

Accession	Gene_Symbol	Gene_ID	Ensembl_Gene ID	log2(ratio(CM12H/CM0H))	log2(ratio(CM12R/CM0H))	log2(ratio(CM12H/CM12R))
E1BDI3	POLG	E1BDI3	ENSBTAG00000009098	-0.482663925	-0.291428417	0.191235508
A0A3Q1LL04	YIPF1	A0A3Q1LL04; bta:511748	ENSBTAG00000050962	-0.042629853	-0.048609944	-0.005980092
A0AAF6DLN3	HID1	A0AAF6DLN3	ENSBTAG00000010809	-0.031364171	0.033947332	0.065311503
A6QLV5	ZFAND3	A6QLV5; bta:532641; F1MWI6	ENSBTAG00000000527	0.160124438	-0.485685398	-0.645809836
Q6B857	CFAP20	bta:445424; Q6B857	ENSBTAG00000013874	-0.077914727	0.003156883	0.081071611
E1BKM9	ZMYM3	E1BKM9	ENSBTAG00000008492	-0.373900432	0.059525565	0.433425997
A6QR43	PJA2	A0A3Q1M5S9; A6QR43; bta:511508	ENSBTAG00000021675	-0.136893376	-0.555962629	-0.419069253
A0AAA9S3N3	CCM2	A0AAA9S3N3	ENSBTAG00000008090	0.021848399	0.027619187	0.005770788
A0A3Q1LRP0	GXYLT1	A0A3Q1LRP0; bta:617596	ENSBTAG00000015024	-0.028014376	-0.091348947	-0.06333457
A0AAA9SDW3	COA1	A0AAA9SDW3	ENSBTAG00000012968	0.038034585	-0.031166541	-0.069201127
E1BAK9	SYVN1	E1BAK9	ENSBTAG00000005076	0.034765418	-0.022697735	-0.057463153
F1N6V1	ARHGAP6	F1N6V1	ENSBTAG00000002626	-0.280107919	-0.112400743	0.167707176
G9MD91	LGALS9	bta:510813; G9MD91		-0.138679314	-0.135448193	0.003231121
F1MDC7	MSANTD2	F1MDC7	ENSBTAG00000011248	-0.368800278	-0.520667646	-0.151867368
Q3MHY8	RBM7	bta:515307; Q3MHY8	ENSBTAG00000008219	-0.124179411	-0.291573812	-0.167394401
A5PJP6	BRCC3	A5PJP6; bta:519513; Q58D60	ENSBTAG00000007817	-0.011393505	-0.04612405	-0.034730545
Q2KJA0	ARAF	F1MQC5; Q2KJA0		-0.150702782	-0.255508864	-0.104806081
A0AAA9U2H6	IGHMBP2	A0AAA9U2H6	ENSBTAG00000022185	-0.066883678	-0.234750062	-0.167866384
A3KMW9	ZNF644	A3KMW9; bta:539923	ENSBTAG00000011200	-0.068778278	-0.025750994	0.043027284
A0A3Q1M007	ZC3HAV1L	A0A3Q1M007	ENSBTAG00000014250	0.021720197	0.005041455	-0.016678741
A0AAA9T2R3	DEPDC1B	A0AAA9T2R3	ENSBTAG00000017026	0.075288127	0.182951682	0.107663554
Q1JQA5	NENF	bta:616334; Q1JQA5	ENSBTAG00000000759	-0.115908968	0.114480675	0.230389643
A0AAA9TXZ0	PAN2	A0AAA9TXZ0	ENSBTAG00000004376	-0.047252283	0.023846742	0.071099025
F1N193	ZBTB40	F1N193	ENSBTAG00000016448	-0.253578614	-0.415423195	-0.161844581
Q3SX05	ECSIT	bta:507245; Q3SX05	ENSBTAG00000015049	-0.222200688	-0.352742418	-0.13054173
Q3ZC10	SNRPD1	bta:508788; Q3ZC10	ENSBTAG00000008292	-0.091749929	-0.090197809	0.00155212
Q3ZBW6	DECR2	bta:768256; Q3ZBW6	ENSBTAG00000026429	-0.024631977	-0.323237058	-0.298605081
Q2NL38	DCI	bta:535174; Q2NL38		0.053843583	0.042370798	-0.011472784
A0A3Q1M3P1	CARD11	A0A3Q1M3P1	ENSBTAG00000014698	0.015891373	-0.099209604	-0.115100977
A0AAA9S6A6	RBPJ	A0AAA9S6A6	ENSBTAG00000003602	-0.04324085	-0.062388692	-0.019147842
A5PJS5	ORC5	A5PJS5; bta:519409; F1N4D2	ENSBTAG00000040058	-0.125530882	-0.130008987	-0.004478105
Q0P5F0	ACP2	bta:535407; Q0P5F0		0.004024257	0.10239852	0.098374263
A0A3Q1LPN8	HERC5	A0A3Q1LPN8	ENSBTAG00000020538	-0.231019599	-0.028428408	0.20259119
Q0IIG6	TARBP2	bta:514674; Q0IIG6		0.33248874	0.053466025	-0.279022715
A0A3Q1N7N8	RRP7	A0A3Q1N7N8	ENSBTAG00000016510	-0.180400965	-0.337102604	-0.156701638
F1MYV8	MLF2	F1MYV8	ENSBTAG00000014931	0.141391537	-0.222392421	-0.363783958
A0AAA9SXH6	JAK2	A0AAA9SXH6; bta:525246	ENSBTAG00000012047	-0.104391391	-0.15224636	-0.047854969
E1BBI5	ZBTB11	E1BBI5	ENSBTAG00000013460	0.116193018	-0.182612519	-0.298805537
A0A3Q1MMY4	QSER1	A0A3Q1MMY4	ENSBTAG00000014399	-0.057820654	-0.130692587	-0.072871933
F1MNL4	NEO1	F1MNL4	ENSBTAG00000004990	0.161306548	-0.106504998	-0.267811546
P82931	MRPS6	bta:615431; P82931; Q3ZC18	ENSBTAG00000012594	0.026694867	-0.355066478	-0.381761344
A0AAA9SD03	C2CD2	A0AAA9SD03	ENSBTAG00000021259	0.263286427	0.306725688	0.043439261
F1MDV2	SLC30A9	bta:522345; F1MDV2	ENSBTAG00000020367	-0.002923395	0.091922489	0.094845885
F1N378	ETNK1	F1N378	ENSBTAG00000005833	0.233066369	-0.238583498	-0.471649867
O46728				0.113231777	0.13280452	0.019572743
E1BEN8	TAF5	bta:516001; E1BEN8	ENSBTAG00000009709	-0.111660417	-0.170908099	-0.059247681
Q3MHY0	TERF1	bta:519474; G3X7V3; Q3MHY0	ENSBTAG00000032982	-0.03145415	-0.130153236	-0.098699086
A6QP88	ZFAND1	A6QP88		-0.350428418	-0.308909148	0.041519269
A0A3Q1NF54	PISD	A0A3Q1NF54	ENSBTAG00000067410	0.092401631	0.014483396	-0.077918235
A0AAA9SBR4	ERBB2	A0AAA9SBR4; bta:505709	ENSBTAG00000021798	-0.117084677	-0.804947086	-0.687862408
A3KN00	DUSP23	A3KN00; bta:513978	ENSBTAG00000004776	-0.032604665	-0.254997087	-0.222392421
Q3SZV6	TIMM21	bta:614633; Q3SZV6		0.216409123	0.07169273	-0.144716392
E1BL71				0.035156445	-0.285102829	-0.320259275
E1BLQ5	RPS6KB2	E1BLQ5	ENSBTAG00000009175	-0.11783649	-0.082724922	0.035111568
A0AAF6Z8G4	HSF1	A0AAF6Z8G4	ENSBTAG00000020751	-0.192463823	-0.218801787	-0.026337963
A0A3Q1LPC6	YIPF3	A0A3Q1LPC6	ENSBTAG00000018967	0.139128485	0.058416686	-0.080711799
A0AAA9SXT9	NDUFAF2	A0AAA9SXT9	ENSBTAG00000021607	0.047488926	0.059802187	0.012313261
A0AAA9TVB1	TAX1BP3	A0AAA9TVB1	ENSBTAG00000000833	-0.276383959	-0.020712709	0.255671251
A0AAF6YUJ1	RRP15	A0AAF6YUJ1	ENSBTAG00000009838	0.267419501	-0.255937231	-0.523356731
F1N551	GATA4	bta:327716; F1N551	ENSBTAG00000005425	0.059998411	-0.255605846	-0.315604257
F1MYL8	MED13	F1MYL8	ENSBTAG00000011721	0.104099171	0.188445089	0.084345919
F6RLZ6	TRIM32	F6RLZ6	ENSBTAG00000017155	0.393011193	0.260062839	-0.132948354
A0A3Q1MU04	APBB1	A0A3Q1MU04	ENSBTAG00000015655	0.156504486	0	-0.156504486
A5PJX0	FBXO22	A5PJX0; bta:536605	ENSBTAG00000002833	0.112445782	0.074463652	-0.037982131
F1MJ19	PCIF1	F1MJ19	ENSBTAG00000010126	0.188072348	-0.038333136	-0.226405484
F6QJC0	POMGNT1	F6QJC0	ENSBTAG00000013322	-0.055200167	-0.070915955	-0.015715788
F1MER3	CWC25	F1MER3	ENSBTAG00000030591	0.348754948	0.013043037	-0.33571191
A0AAF6DMB2	AAGAB	A0AAF6DMB2	ENSBTAG00000040368	0.249945949	-0.037437301	-0.28738325
Q3SZG8	ISCA1	bta:514652; Q3SZG8	ENSBTAG00000053156	-0.054398897	-0.281197842	-0.226798946
A0AAF6ZH32	BABAM1	A0AAF6ZH32	ENSBTAG00000012587	-0.038872892	0.173526421	0.212399312
A0AAA9SAD6	PDE4D	A0AAA9SAD6	ENSBTAG00000000494	0.108479101	-0.041268018	-0.14974712
E1BFV2	MAP3K21	bta:616014; E1BFV2	ENSBTAG00000021183	-0.040806216	-0.086864237	-0.046058021
A0AAA9T7R0	ADISSP	A0AAA9T7R0	ENSBTAG00000018735	-0.119298928	-0.088536675	0.030762254
Q2YDI8	MKRN2	bta:508004; Q2YDI8	ENSBTAG00000004512	0.221926058	0.043404684	-0.178521374
A0AAA9SBZ8	UBR2	A0AAA9SBZ8	ENSBTAG00000005967	-0.134066079	-0.131047887	0.003018192
E1B8D0	GEN1	bta:785690; E1B8D0	ENSBTAG00000020059	0.541153139	0.199258495	-0.341894644
A0A452DIR9	BUD23	A0A452DIR9	ENSBTAG00000017067	0.05270705	-0.229343021	-0.282050071
A5D7F6	MAML2	A5D7F6; bta:521194		-0.460794323	-0.468312292	-0.007517969
Q2KHW8	GEMIN6	bta:525263; Q2KHW8		-0.101724062	-0.154698527	-0.052974465
Q3T0H4	ZCCHC17	bta:538816; Q3T0H4	ENSBTAG00000016818	0.191019506	0.315601291	0.124581785
Q3T103	CDIPT	bta:515135; Q3T103	ENSBTAG00000007954	-0.027672418	0.034468757	0.062141175
E1BK34	DVL2	E1BK34	ENSBTAG00000003075	-0.164507571	0.03869529	0.203202861
A0AAA9TK64	GNB1L	A0AAA9TK64	ENSBTAG00000021052	-0.186691852	-0.179046391	0.007645461
F1MYR3	GAS8	F1MYR3	ENSBTAG00000007096	-0.822183391	-0.570284894	0.251898496
A0AAA9SJ57	KIAA0319L	A0AAA9SJ57	ENSBTAG00000016694	0.06871275	-0.02319276	-0.09190551
A6QP29	TBC1D2	A6QP29; bta:513828		-0.275157965	-0.273332924	0.001825041
E1BP59	KAT2A	E1BP59	ENSBTAG00000044019	-0.120528086	-0.145593424	-0.025065337
M5FMW2	MAPK8IP3	A0A3Q1MTK3; A0A8J8XE10; M5FMW2	ENSBTAG00000002211	-0.158290301	0.057257318	0.215547619
A0A3Q1MW97	ANO1	A0A3Q1MW97	ENSBTAG00000009997	0.148425423	0.320438477	0.172013054
A0AAA9SME8	ANKIB1	A0AAA9SME8; bta:505204	ENSBTAG00000034936	-0.012060217	0.058833638	0.070893855
A0AAA9SVS9	CASP7	A0AAA9SVS9; bta:526279	ENSBTAG00000006615	0.227550026	0.230978214	0.003428188

Accession	Gene_Symbol	Gene_ID	Ensembl_Gene ID	log2(ratio(CM12H/CM0H))	log2(ratio(CM12R/CM0H))	log2(ratio(CM12H/CM12R))
F6QWY1	ELP5	F6QWY1	ENSBTAG00000019446	-0.011002494	-0.067309935	-0.056307441
A0AAF6Z9K7	IFNAR1	A0AAF6Z9K7	ENSBTAG00000021819	0.161087436	-0.313373084	-0.474460521
A0AAA9TCS3	ZNF687	A0AAA9TCS3	ENSBTAG00000007318	-0.012683115	0.0327722	0.045455315
F1MPD0	NRG1	F1MPD0	ENSBTAG00000004150	0.443531415	0.245232398	-0.198299017
E1BBV0	ZNF703	E1BBV0	ENSBTAG00000013943	0.230973197	-0.207670916	-0.438644112
A0AAA9SWL7	C23H6orf47	A0AAA9SWL7	ENSBTAG00000023628	0.130396637	0.180219336	0.049822699
Q3SX23	LONP2	bta:541085; Q3SX23	ENSBTAG00000010867	-0.123250577	-0.11044077	0.012809807
E1BAQ2	YTHDF1	E1BAQ2	ENSBTAG00000010383	0.219009782	0.189685702	-0.029324079
A0AAA9S999	MPHOSPH6	A0AAA9S999	ENSBTAG00000009746	0.075443867	-0.003943588	-0.079387455
A0AAA9T4I5	MAP3K3	A0AAA9T4I5	ENSBTAG00000008151	0.300674189	0.03522523	-0.265448959
A0AAF6YS40	RILPL2	A0AAF6YS40	ENSBTAG00000007804	0.26174686	-0.149003281	-0.410750141
A0A3Q1LY17	TIMM9	A0A3Q1LY17	ENSBTAG00000010503	-0.312998223	-0.464710421	-0.151712198
A0AAA9SIN1	PHKA1	A0AAA9SIN1	ENSBTAG00000015848	-0.190605933	-0.194687109	-0.004081177
A0AAA9TA72	WDR73	A0AAA9TA72	ENSBTAG00000001387	-0.02867239	-0.041896085	-0.013223695
A0AAA9U2N4	WDR4	A0AAA9U2N4	ENSBTAG00000021490	0.063086702	0.058773709	-0.004312993
A0AAA9TN08	ARL6IP4	A0AAA9TN08; bta:526095	ENSBTAG00000001594	0.143748379	0.072952601	-0.070795778
A0A3Q1MCN9	TBC1D24	A0A3Q1MCN9	ENSBTAG00000005458	-0.020255145	-0.108181652	-0.087926507
A0A3Q1LGL5	GOLT1B	A0A3Q1LGL5	ENSBTAG00000000379	0.06333457	0.005619073	-0.057715498
A8E640	GGCX	A8E640		-0.595296647	-0.660618433	-0.065321786
F6QPX6	BRI3BP	F6QPX6	ENSBTAG00000001543	0.196969824	0.128022471	-0.068947354
F1N264	ZEB1	bta:535183; F1N264	ENSBTAG00000020053	-0.186878135	-0.600779206	-0.413901071
A0A3Q1M1G7	ATF2	A0A3Q1M1G7	ENSBTAG00000002295	0.212716664	0.059960794	-0.152755869
Q0II95	IP6K1	bta:534029; F1N7H3; Q0II95		0.152151664	0.090692985	-0.061458679
A0AAF6YSM1				0.028332906	0.238225212	0.209892305
A0AAA9TQT4				0.284143432	0.140177658	-0.143965774
A5PJT2	INTS10	A5PJT2; bta:534094	ENSBTAG00000025003	-0.315970157	0	0.315970157
E1B843	NAA20	bta:540212; E1B843	ENSBTAG00000001144	0.116253068	0.190120677	0.073867608
A2VDM1	LAMB3	A2VDM1; bta:529939		0.045017538	0.06263846	0.017620922
A0AAA9T7M7	CNOT6L	A0AAA9T7M7	ENSBTAG00000049571	-0.058594654	-0.130629443	-0.072034789
A0A3Q1NBH6	GLS	A0A3Q1NBH6	ENSBTAG00000007863	0.634905921	0.624534237	-0.010371684
F6QTX4	RIPK3	F6QTX4	ENSBTAG00000018422	-0.208694572	-0.125530882	0.08316369
Q32KQ2	WDR53	bta:518441; Q32KQ2	ENSBTAG00000014660	-0.048255169	-0.314236858	-0.26598169
P20821	GCSH	bta:317723; P20821; Q0P5G3	ENSBTAG00000006795	0.062887706	0.105000884	0.042113178
A0AAF6YIZ9	MRPS12	A0AAF6YIZ9	ENSBTAG00000001782	0.037670418	-0.066387397	-0.104057815
G3MWS8	KLF16	G3MWS8	ENSBTAG00000038055	-0.386740528	-0.404462298	-0.01772177
A0AAA9T330	PPP1R3D	A0AAA9T330; bta:782160	ENSBTAG00000061854	0.157140489	0.093213162	-0.063927327
G3MWQ7				0.108860061	0.235758876	0.126898814
A0A3Q1MJ38	CDKAL1	A0A3Q1MJ38	ENSBTAG00000025589	-0.234905033	-0.329422631	-0.094517599
Q3T0Y8	VAMP8	bta:507309; Q3T0Y8	ENSBTAG00000023997	-0.001424884	-0.034591748	-0.033166864
A0A3Q1N3E7	NEURL4	A0A3Q1N3E7	ENSBTAG00000010519	0.077103153	0.229253452	0.1521503
A0AAA9TJJ3	PITPNM1	A0AAA9TJJ3	ENSBTAG00000010482	-0.184691712	-0.31204533	-0.127353618
A0A0M3T9B6	OPN	A0A0M3T9B6		-0.200241277	-1.122836773	-0.922595496
A7Z054	TXNIP	A7Z054; bta:506790	ENSBTAG00000020060	0.370547859	-0.157750287	-0.528298147
G3N060	FAM98C	G3N060	ENSBTAG00000039593	-0.051017737	-0.277201738	-0.226184
F6R592	FCGRT	F6R592	ENSBTAG00000013926	0.074229079	-0.049592682	-0.123821761
Q3ZC24	MARCHF5	bta:540937; Q3ZC24	ENSBTAG00000020981	-0.028682755	-0.04324085	-0.014558096
A6QPN6	IFI30	A6QPN6; bta:615930		-0.005417562	0.255081669	0.260499231
Q3ZC34	RBMS2	bta:509798; Q3ZC34	ENSBTAG00000017830	0.10622032	0.267081239	0.160860919
A5PKL7	LZTS2	A5PKL7; bta:504411		-0.465803307	-0.64691599	-0.181112682
A2VDQ6	CDH11	A2VDQ6; bta:785475; E1BHE3	ENSBTAG00000032092	0.015329148	0.061499329	0.046170181
F1MG92	PDP1	F1MG92	ENSBTAG00000000199	-0.055586762	0.080296666	0.135883428
F1N6K5	HDHD5	F1N6K5	ENSBTAG00000016995	-0.076692757	-0.054750204	0.021942553
Q2KHU4	CLPP	bta:535981; Q2KHU4	ENSBTAG00000014712	0.043426378	0.182538258	0.13911188
A0AAA9T506	C18H19orf47	A0AAA9T506	ENSBTAG00000018859	0.043193517	-0.019938847	-0.063132364
Q5EA92	ARL14EP	bta:523894; Q2KIN9; Q5EA92	ENSBTAG00000012417	0.251474503	0.224227328	-0.027247175
A6QLZ6	GLRX5	A6QLZ6; bta:505935	ENSBTAG00000013423	0.042169454	0.432959407	0.390789953
F1N1J9	SOS2	F1N1J9	ENSBTAG00000005550	0.173788541	-0.101916538	-0.275705078
E1BC48	CEP350	bta:534896; E1BC48	ENSBTAG00000008082	0.067114196	-0.040641984	-0.10775618
E1B9B6	NUAK1	bta:519892; E1B9B6	ENSBTAG00000010457	-0.181446871	-0.242200902	-0.060754031
A0A3Q1LRR1	MTMR3	A0A3Q1LRR1	ENSBTAG00000001030	-0.106053508	-0.124258103	-0.018204595
E1BPC2	PDZD8	E1BPC2	ENSBTAG00000011401	-0.140692389	-0.195871948	-0.055179559
A0AAA9SU74	WASHC3	A0AAA9SU74	ENSBTAG00000000021	-0.132669417	-0.09567521	0.036994207
E1BD64	MPC2	bta:616718; E1BD64	ENSBTAG00000020968	0.087575839	0.105542385	0.017966547
A0AAA9T0G1	HUS1	A0AAA9T0G1	ENSBTAG00000013226	0.066837765	0.056641997	-0.010195767
F1MM71	IFIT1	F1MM71	ENSBTAG00000007881	0.031659155	1.437440487	1.405781331
E1BGL4	CPEB4	bta:538794; E1BGL4	ENSBTAG00000009995	-0.154920577	-0.150546561	0.004374016
A0A140T876	RER1	A0A140T876	ENSBTAG00000009307	0.208998459	0.100453321	-0.108545138
A0A3Q1MIR4	CEP44	A0A3Q1MIR4	ENSBTAG00000020055	-0.209083533	-0.291992888	-0.082909355
A0AAA9SEB2	CARS2	A0AAA9SEB2	ENSBTAG00000011471	-0.089066352	-0.225995783	-0.136929431
F1MY22	TMEM63B	bta:534891; F1MY22	ENSBTAG00000032680	-0.218088379	-0.139191216	0.078897164
E1BJ55	ZNF428	bta:540173; E1BJ55	ENSBTAG00000012796	-0.150546561	-0.176991814	-0.026445252
Q5E9Z1	RPUSD4	A7YWI2; bta:516934; Q0V8H4; Q1JP71; Q1JPL3; Q58D36; Q58DF5; Q5E9Z1; Q5E9Z6		-0.07058806	0.057143907	0.127731967
A0A3Q1N449	TGFB1	A0A3Q1N449	ENSBTAG00000020457	-0.15102797	0.104404713	0.255432683
F1MAV4	BARD1	F1MAV4	ENSBTAG00000033690	-0.001330286	0.014552756	0.015883042
A0AAA9STM1	AP1S2	A0AAA9STM1	ENSBTAG00000020420	0.110476536	0.124282336	0.0138058
G3MXK1	ITPRIPL2	bta:783291; G3MXK1	ENSBTAG00000057103	0.004592137	-0.003069565	-0.007661702
Q0VCT5	OSTM1	bta:518522; Q0VCT5	ENSBTAG00000010134	0.041634489	-0.039528364	-0.081162853
E1BFR4	PLXNA1	E1BFR4	ENSBTAG00000009159	0.045663379	-0.207256949	-0.252920328
F1MI39	CHKA	F1MI39	ENSBTAG00000010304	0.11880743	0.441875545	0.323068115
Q2KHZ3	MTF2	bta:615211; F1MPR4; Q2KHZ3	ENSBTAG00000001393	0.206753298	0.181639328	-0.02511397
Q17QJ0	ASF1B	bta:510538; Q17QJ0	ENSBTAG00000004085	-0.112657801	-0.07364414	0.03901366
A6H757	ACP6	A6H757; bta:515738	ENSBTAG00000017051	-0.146401448	-0.237875453	-0.091474005
Q3SZ46	NOL7	bta:510511; Q3SZ46	ENSBTAG00000021554	0.283739125	-0.064747181	-0.348486306
F1MBS7	MALT1	bta:504994; F1MBS7	ENSBTAG00000027916	0.123529918	-0.251681601	-0.375211519
F6QB59	GMNN	F6QB59	ENSBTAG00000017329	0.810029056	-0.675397771	-1.485426827
F1MC78	CCNDBP1	F1MC78	ENSBTAG00000013032	0.316283986	-0.130946517	-0.447230504
Q3T0J3	MRPL16	bta:538054; Q3T0J3	ENSBTAG00000021188	-0.049105472	0.062524254	0.111629727
A0AAA9SJP0	CHML	A0AAA9SJP0	ENSBTAG00000061790	-0.044441279	-0.219628396	-0.175187117
A0A3Q1LH29	SPIN1	A0A3Q1LH29	ENSBTAG00000003044	0.195995372	0.142660104	-0.053335268

Accession	Gene_Symbol	Gene_ID	Ensembl_Gene ID	log2(ratio(CM12H/CM0H))	log2(ratio(CM12R/CM0H))	log2(ratio(CM12H/CM12R))
Q58CQ7	RASSF8	bta:515639; F1MC74; Q58CQ7	ENSBTAG00000021980	-0.194647431	-0.18665464	0.007992791
A0A3Q1MLB6	UBE2R2	A0A3Q1MLB6; bta:537885	ENSBTAG00000054773	0.178405711	-0.070904668	-0.249310379
A0AAA9S3Q1	UQCRH	A0AAA9S3Q1	ENSBTAG00000009603	-0.364778399	-0.548359395	-0.183580996
A4IFI9	JAM2	A4IFI9; bta:538846	ENSBTAG00000000603	0.313250971	-0.105545773	-0.418796745
A0AAA9SYM8	ISCA2	A0AAA9SYM8	ENSBTAG00000021956	0.257629601	0.187627003	-0.070002598
A0AAA9SLK1	SYNJ2BP	A0AAA9SLK1	ENSBTAG00000007586	-0.017903374	0.155853047	0.173756421
F1MEI8	GTF3C2	bta:782752; F1MEI8	ENSBTAG00000017283	-0.042855479	-0.015566257	0.027289222
A0AAA9TJZ0	IGF2BP1	A0AAA9TJZ0	ENSBTAG00000011736	0.007777351	-0.018311878	-0.026089229
Q0VC13	MAPDA	bta:512667; Q0VC13		0.018082359	0.043527376	0.025445017
F6RQE7	PIP4P1	F6RQE7	ENSBTAG00000002748	0.189033824	0.537807648	0.348773824
A0JNA1	SGPP1	A0JNA1; bta:516881	ENSBTAG00000004430	0.110381371	-0.057825671	-0.168207042
A4IFE5	LRRC8D	A4IFE5; bta:539204	ENSBTAG00000020958	-0.138624064	-0.236839192	-0.098215129
A0A3S5ZP82	LAD1	A0A3S5ZP82	ENSBTAG00000024663	-0.093109404	0.079484784	0.172594188
A0AAA9S6E2	C3H1orf50	A0AAA9S6E2	ENSBTAG00000017386	-0.345376621	-0.021830248	0.323546373
A0A3Q1NCS8	NOSTRIN	A0A3Q1NCS8	ENSBTAG00000010362	-0.276221156	-0.045284469	0.230936687
A0AAA9SUM5	SZRD1	A0AAA9SUM5	ENSBTAG00000009251	-0.047684798	0.036490341	0.084175139
A0AAA9T2L7	DDX11	A0AAA9T2L7	ENSBTAG00000007093	0.014964439	-0.040212268	-0.055176707
F1N3U6	UVRAG	bta:614285; F1N3U6	ENSBTAG00000016355	0.100101992	0.15607563	0.055973638
F1MPJ6	PLEKHG5	bta:615910; F1MPJ6	ENSBTAG00000016728	0.375098754	-0.084491064	-0.459589818
A7MBG4	KEAP1	A7MBG4; bta:532791; F1MV29	ENSBTAG00000003543	-0.066919592	-0.061222862	0.00569673
F1MF33	IFT56	bta:508011; F1MF33	ENSBTAG00000016373	-0.091350462	0.050358297	0.141708759
F6Q6Z7	PPHLN1	F6Q6Z7	ENSBTAG00000008595	-0.020391789	-0.164714145	-0.144322356
A0AAA9TX07	FADS3	A0AAA9TX07	ENSBTAG00000015511	-0.411943957	-0.342984831	0.068959126
F1MPV3	RALBP1	bta:523324; F1MPV3	ENSBTAG00000021250	0.115477217	0.139930261	0.024453044
A0A3Q1LLN9	RPL7L1	A0A3Q1LLN9	ENSBTAG00000018478	-0.469919869	-0.399679572	0.070240297
A0AAA9SKU2	LYSMD1	A0AAA9SKU2	ENSBTAG00000020395	-0.070317838	-0.187547571	-0.117229732
A2VDS1	SCLY	A2VDS1; bta:790815		0.028890979	0.083272549	0.05438157
A0AAA9T930	CLEC16A	A0AAA9T930	ENSBTAG00000005208	0.102154544	0.145181828	0.043027284
E1BGT6	PUS7L	E1BGT6	ENSBTAG00000004718	-0.363081583	-0.222790242	0.140291341
F6QGE6	ACOT13	F6QGE6	ENSBTAG00000016337	0.283190602	0.418312631	0.135122029
A5PJV0	MAFG	A5PJV0; bta:515219	ENSBTAG00000000040	-0.191365526	-0.058787016	0.132578509
Q3T0Q2	TMEM59	bta:509775; F1MBR3; Q3T0Q2		0.029039673	-0.400004684	-0.429044357
F6QED7	C18H19orf48	bta:516719; F6QED7	ENSBTAG00000011079	-0.294871796	-0.576951958	-0.282080162
A0AAA9S4Y4				-0.158540238	-0.1005036	0.058036637
Q0VCI8	PPP1R14C	bta:617148; F1MRD7; Q0VCI8	ENSBTAG00000026586	0.495307313	-0.127444017	-0.622751331
A0AAA9T4I3	ARL6IP1	A0AAA9T4I3	ENSBTAG00000049516	-0.055557976	0.056583528	0.112141505
A0AAF6ZNZ9				-0.002839952	0.086711633	0.089551585
Q0P5I6	PRPF38A	bta:507240; Q0P5I6	ENSBTAG00000002720	0.174395705	0.059216043	-0.115179662
A0A3Q1M7A3	PLEKHA1	A0A3Q1M7A3	ENSBTAG00000004636	-0.167109986	-0.050465067	0.116644919
Q29RH8	PDK2	Q29RH8		0.078563669	0.20685707	0.128293401
E1BB20				0.173360809	-0.037637657	-0.210998467
A0AAA9TML5	PTBP2	A0AAA9TML5	ENSBTAG00000008809	-0.10371135	0.315480552	0.419191901
A6H7E1	MARS2	A6H7E1; bta:514726; Q0V8Q7	ENSBTAG00000002056	-0.05294888	-0.125530882	-0.072582002
A7YWI9	TDP2	A7YWI9; bta:507579	ENSBTAG00000000365	0.148808985	-0.087202763	-0.236011748
Q148F9	C25H7ORF26	bta:517505; Q148F9		-0.276935223	-0.212544635	0.064390587
Q32LJ4	TPRG1L	A5D9I0; bta:616333; Q32LJ4		-0.25761127	-0.271351327	-0.013740057
A0A3Q1MLW5	FBXO21	A0A3Q1MLW5	ENSBTAG00000032097	0.007752276	-0.221356375	-0.22910865
A0AAA9TGJ1	TBC1D25	A0AAA9TGJ1	ENSBTAG00000009288	0.09046301	-0.025014207	-0.115477217
A0AAF6ZC43	SNRPC	A0AAF6ZC43	ENSBTAG00000024549	0.050358297	-0.114700949	-0.165059246
A0AAF6ZAY0	QPCTL	A0AAF6ZAY0	ENSBTAG00000012176	-0.038665109	0.159050958	0.197716067
A0A3Q1LTD9	ARHGAP10	A0A3Q1LTD9	ENSBTAG00000002531	-0.117898253	0.056918429	0.174816682
A0A3Q1M3R2	PDE8A	A0A3Q1M3R2	ENSBTAG00000015909	-0.066910051	0.029703594	0.096613645
Q0VCF3	SIKE1	bta:506848; Q0VCF3		-0.034382994	-0.337034987	-0.302651993
A0A3Q1MH84	S100A13	A0A3Q1MH84	ENSBTAG00000021378	0.066342495	-0.212397691	-0.278740186
Q17QU5	MLST8	bta:535236; Q17QU5; Q58DN5		-0.081844133	0.007486758	0.089330892
Q05KI7	BAK1	bta:514090; Q05KI7		-0.084752852	-0.279894139	-0.195141287
A0A3Q1MSM3	TASOR2	A0A3Q1MSM3	ENSBTAG00000019197	-0.141073533	-0.206028975	-0.064955442
Q2NKZ7	PTPMT1	bta:614890; Q2NKZ7	ENSBTAG00000018479	-0.463567393	-0.396798381	0.066769012
A0A3Q1LWE9	HDAC4	A0A3Q1LWE9	ENSBTAG00000017764	-0.402182294	-0.391363963	0.010818332
Q3T0Z5	LENG1	bta:617891; Q3T0Z5		0.213109181	-0.324875379	-0.537984559
F1MX50	CREG1	F1MX50	ENSBTAG00000008931	0.236590738	0.389849167	0.15325843
A0AAA9SRY9	SLC25A46	A0AAA9SRY9	ENSBTAG00000009667	-0.067626426	-0.080565481	-0.012939056
A0AAF7AC02	FSD1	A0AAF7AC02	ENSBTAG00000005999	0.207864591	0.040233925	-0.167630667
A0AAF6ZDK8	NUDT12	A0AAF6ZDK8	ENSBTAG00000027728	0.071727683	0.420973414	0.349245731
A0AAA9TY08	FBXL18	A0AAA9TY08	ENSBTAG00000010264	-0.40801305	-0.10433666	0.30367639
E1BC94	IBA57	bta:506009; E1BC94	ENSBTAG00000014918	0.195802164	0.245577209	0.049775044
A0AAA9SRA4	TRIM16	A0AAA9SRA4	ENSBTAG00000002605	-0.088056177	-0.11042399	-0.022367813
A0AAA9SX17	KDM5A	A0AAA9SX17; bta:507962	ENSBTAG00000020472	-0.065783419	-0.005602706	0.060180713
E1BCV8	FYTTD1	E1BCV8	ENSBTAG00000014874	0.284404242	0.117989061	-0.166415181
E1BPL5	KDM4A	bta:512622; E1BPL5	ENSBTAG00000002078	0.221562809	0.069421401	-0.152141408
Q1RMT3	MTHFS	bta:513372; Q1RMT3	ENSBTAG00000020023	-0.232263531	-0.186334845	0.045928687
A0A3Q1LLL8	RAB20	A0A3Q1LLL8	ENSBTAG00000049416	-0.159340251	-0.054120939	0.105219312
F1ML71	NDFIP2	F1ML71	ENSBTAG00000014227	-0.012028097	-0.161796186	-0.149768089
A0AAA9TLR9	COQ9	A0AAA9TLR9	ENSBTAG00000001855	-0.397139245	-0.311904367	0.085234878
A0AAA9T1H8	STX8	A0AAA9T1H8	ENSBTAG00000017815	-0.211305172	-0.163952402	0.04735277
Q2T9W9	CAAP1	bta:515858; Q2T9W9	ENSBTAG00000001223	0.250823942	-0.189033824	-0.439857767
A0A3Q1M8E6	NHSL2	A0A3Q1M8E6	ENSBTAG00000045565	0.451048035	0.335184192	-0.115863843
A0A3Q1LJR3	GPAA1	A0A3Q1LJR3	ENSBTAG00000014610	-0.014088931	-0.024033692	-0.009944761
A0A452DIV3	CCND1	A0A452DIV3	ENSBTAG00000017514	-0.374658756	-0.751591651	-0.376932894
A7MB66	ABHD13	A7MB66; bta:513848	ENSBTAG00000067980	-0.028014376	0.089637212	0.117651589
A0AAF6YP33	FNTB	A0AAF6YP33	ENSBTAG00000005897	0.073797752	-0.028677325	-0.102475076
Q2HJE8	CHCHD1	bta:540610; F1MUI6; Q2HJE8	ENSBTAG00000012654	-0.064074481	-0.383435502	-0.319361021
A0A3Q1N2T8	CDC34	A0A3Q1N2T8; bta:616156	ENSBTAG00000002098	-0.291354302	-0.410543657	-0.119189355
E1BNX4	CREB3L1	E1BNX4	ENSBTAG00000006143	0.008777833	-0.537940833	-0.546718666
A0AAA9SB42	DGUOK	A0AAA9SB42	ENSBTAG00000012037	0.039075379	0.116999851	0.077924472
A0A3Q1MVP2	CERS5	A0A3Q1MVP2	ENSBTAG00000017395	0.170757054	0.123406975	-0.047350079
Q2NL16	FBXO28	bta:537745; Q2NL16	ENSBTAG00000019538	-0.128539381	-0.115587418	0.012951963
F1N3Y6	ABCC3	F1N3Y6	ENSBTAG00000020070	-0.125831037	-0.216900618	-0.091069581
A0AAA9S0Z5	POLR3F	A0AAA9S0Z5	ENSBTAG00000008825	-0.076621282	-0.226325259	-0.149703977
F1MQ15	IFFO2	F1MQ15	ENSBTAG00000030340	-0.069168439	-0.325885122	-0.256716682
A0AAA9S938	SCAMP4	A0AAA9S938	ENSBTAG00000045762	0.25569243	0.239655467	-0.016036963

Accession	Gene_Symbol	Gene_ID	Ensembl_Gene ID	log2(ratio(CM12H/CM0H))	log2(ratio(CM12R/CM0H))	log2(ratio(CM12H/CM12R))
A6QLM8	HSCB	A6QLM8; bta:786040		-0.069386063	-0.188707687	-0.119321624
A0A3Q1LK65	THTPA	A0A3Q1LK65	ENSBTAG00000007097	0.111483108	0.280466709	0.168983602
A0AAA9TVQ8	CMC1	A0AAA9TVQ8	ENSBTAG00000035286	0.071384633	0.213403638	0.142019005
A2VDU2	MAPKAP1	A2VDU2; bta:533861	ENSBTAG00000010271	-0.082132627	-0.051326581	0.030806047
F1MGX8	SPDL1	F1MGX8	ENSBTAG00000008180	0.028485581	-0.700839338	-0.729324919
E1BEK4	RAD54L2	E1BEK4	ENSBTAG00000032253	-0.263829498	-0.055365237	0.208464261
A0AAA9TBA2	GSE1	A0AAA9TBA2	ENSBTAG00000009918	0.018469022	-0.064325327	-0.082794349
A0AAF6ZA81	POLR2K	A0AAF6ZA81	ENSBTAG00000022539	0.002649578	-0.144948336	-0.147597914
A0A3Q1LFP3	UBE2G2	A0A3Q1LFP3	ENSBTAG00000048090	-0.078082186	0.157442371	0.235524557
Q08DD7	SNX11	bta:534567; Q08DD7		0.074468407	0.026273694	-0.048194712
A0A3Q1M1Z7	PIAS1	A0A3Q1M1Z7	ENSBTAG00000005564	0.059527701	-0.22702387	-0.286551571
A0A3Q1LL74	PLD1	A0A3Q1LL74	ENSBTAG00000017490	0.105810283	-0.099204362	-0.205014645
A0AAA9S5G5	KIF21A	A0AAA9S5G5	ENSBTAG00000004832	-0.175849835	-0.093635072	0.082214763
E1BGA2	MITD1	E1BGA2	ENSBTAG00000003970	0.176404437	0.02591281	-0.150491627
G3MZT8	LTBP4	G3MZT8	ENSBTAG00000004757	0.019224567	0.090357744	0.071133177
A0AAA9TMD6	CLPTM1L	A0AAA9TMD6	ENSBTAG00000001329	0.053737871	-0.180572246	-0.234310117
F1MSR5	TBCK	F1MSR5	ENSBTAG00000004227	-0.169212383	-0.014326781	0.154885602
Q3MHM8	ARL6IP6	bta:507137; Q3MHM8	ENSBTAG00000001435	0.162241482	0.011216344	-0.151025137
F1MW45	DDX55	F1MW45	ENSBTAG00000007266	-0.26579819	-0.223543355	0.042254835
E1BJH0	CNOT8	bta:533443; E1BJH0	ENSBTAG00000012086	-0.566546434	-0.260960078	0.305586357
P82920	MRPS21	bta:614343; P82920; Q2HJ36	ENSBTAG00000024378	-0.096297682	-0.383536356	-0.287238674
F1MVR0	CDK19	bta:511802; F1MVR0	ENSBTAG00000007288	-0.194256128	-0.056020086	0.138236042
A0AAA9SYE7	SERGEF	A0AAA9SYE7	ENSBTAG00000005340	-0.014964439	-0.148098639	-0.1331342
Q3SZT2	NQO2	bta:508566; Q3SZT2		-0.210781131	-0.336049203	-0.125268072
Q08D83-2	RTN3	Q08D83-2		0.151679945	0.174763558	0.023083613
A0AAA9TND5	CHMP6	A0AAA9TND5	ENSBTAG00000038745	0.009056496	-0.007286354	-0.01634285
A0AAA9SNR3	TTC7A	A0AAA9SNR3	ENSBTAG00000032558	-0.04580369	0.06237186	0.10817555
A0AAA9RXV8				-0.068361555	-0.045212577	0.023148978
A0A3Q1MKD4	BRD7	A0A3Q1MKD4	ENSBTAG00000021575	-0.106190049	-0.073220919	0.032969131
Q3SZ07	OVCA2	bta:513845; Q3SZ07	ENSBTAG00000047926	-0.332868958	-0.2677984	0.065070557
Q1RMU0	NAB2	bta:504755; Q1RMU0	ENSBTAG00000006324	-0.096999735	-0.034522765	0.06247697
A0AAA9TSM8	KCTD3	A0AAA9TSM8	ENSBTAG00000006186	-0.17412499	-0.351429522	-0.177304532
Q2KI76	SELENOS	bta:512276; Q2KI76	ENSBTAG00000000640	-0.009211173	-0.099781636	-0.090570464
Q6XBT4	USF1	bta:407239; Q6XBT4	ENSBTAG00000017873	0.345171095	0.147812864	-0.197358232
Q0IIH8	MINDY3	bta:538509; Q0IIH8	ENSBTAG00000010387	-0.092918962	0.113938108	0.20685707
F1N0P9	GID4	F1N0P9	ENSBTAG00000008802	0.176243141	0.077370928	-0.098872213
A0A452DI09	UBE2T	A0A452DI09	ENSBTAG00000004790	-0.172164168	-0.197939378	-0.025775209
F6QEF9	HMGA1	F6QEF9	ENSBTAG00000034529	0.239811449	0.2057596	-0.034051849
A0A3Q1LVW6	ZDHHC13	A0A3Q1LVW6; bta:514176	ENSBTAG00000020642	0.241230036	-0.023269779	-0.264499815
A1L530	SLC5A6	A1L530; bta:516021		0.065848703	-0.019167411	-0.085016114
A6QLE9	MTMR14	A6QLE9; bta:514596	ENSBTAG00000009765	0.054198308	0.061961708	0.0077634
A0A3Q1MA68	GRAMD4	A0A3Q1MA68	ENSBTAG00000021803	0.011391898	-0.131633599	-0.143025497
A0A452DJ79	POP7	A0A452DJ79	ENSBTAG00000026248	0.036776571	0.170084178	0.133307607
A0AAA9TSB6	NEK1	A0AAA9TSB6	ENSBTAG00000026915	-0.074000581	-0.152003093	-0.078002512
A5PK74	RIOX1	A5PK74; bta:511031	ENSBTAG00000025372	0.111271982	-0.06377591	-0.175047893
A0A140T852	MRPL30	A0A140T852	ENSBTAG00000038949	-0.509484296	-0.570253934	-0.060769638
F1MS84	NOVA2	F1MS84	ENSBTAG00000013157	-0.152919381	-0.069235633	0.083683748
A0AAA9T844	ZNG1	A0AAA9T844	ENSBTAG00000000269	0.025324156	0.147035286	0.12171113
G3X7E2	PPP6R2	G3X7E2	ENSBTAG00000018660	-0.452270405	-0.566700294	-0.114429889
F1MSK1	PRR12	F1MSK1	ENSBTAG00000014454	-0.055814296	-0.194548788	-0.138734492
A0A3Q1M1T9	PRORP	A0A3Q1M1T9	ENSBTAG00000009682	-0.164480863	-0.11490916	0.049571703
Q3ZBP4	RCAN1	bta:539640; Q3ZBP4	ENSBTAG00000020035	0.14513544	0.129115219	-0.016020221
Q2T9X3	SPRYD7	bta:615298; Q2T9X3		-0.19727996	-0.124851487	0.072428473
F6QMY2	THY1	F6QMY2	ENSBTAG00000019627	0.102046786	0.117385685	0.015338899
Q0V8R7	NSUN4	bta:618779; Q0V8R7		0.116141595	-0.070821337	-0.186962932
A0AAA9SHW9	WDR19	A0AAA9SHW9	ENSBTAG00000014512	-0.019998073	-0.146182913	-0.126184839
Q0IIB7	C13H20ORF7	bta:522230; Q0IIB7		-0.153489642	-0.115972563	0.037517078
A0A3Q1MMH9	ZNF579	A0A3Q1MMH9; bta:618680	ENSBTAG00000051917	-0.024104557	-0.20515349	-0.181048933
A0AAA9RVZ8	SMAP2	A0AAA9RVZ8	ENSBTAG00000021922	0.160387031	-0.005912693	-0.166299724
A0A452DIP3	DNAJC21	A0A452DIP3	ENSBTAG00000015648	0.087007976	0.023009978	-0.063997998
Q5EA55	GOLGA7	bta:616809; Q0VCT8; Q5EA55	ENSBTAG00000027626	0.152003093	0.121306296	-0.030696797
A0AAA9TFH8	MLLT11	A0AAA9TFH8	ENSBTAG00000015369	-0.410695952	0.449197164	0.859893116
A0AAA9TBX6	CARHSP1	A0AAA9TBX6	ENSBTAG00000069945	-0.157208253	-0.024453044	0.132755209
A0A452DIX7	WDR6	A0A452DIX7	ENSBTAG00000018910	0.141567641	-0.169925001	-0.311492642
E1BMA5	UCK2	E1BMA5	ENSBTAG00000044150	-0.060803279	-0.201175935	-0.140372656
A0AAA9TBI6	BRD1	A0AAA9TBI6	ENSBTAG00000046760	-0.150084616	-0.145299553	0.004785063
A0AAA9T762	MED25	A0AAA9T762	ENSBTAG00000008518	-0.29428534	-0.042114874	0.252170466
Q2KJ57	ABCD1	bta:515178; Q2KJ57	ENSBTAG00000020551	-0.081511079	0.105990696	0.187501775
E1B7N1	APPBP2	bta:505734; E1B7N1	ENSBTAG00000004489	-0.099750825	-0.243885953	-0.144135129
Q0VCQ5	KLHL7	bta:534697; Q0VCQ5	ENSBTAG00000016010	-0.277575337	0.167344558	0.444919895
A0AAA9SIC6	RENBP	A0AAA9SIC6	ENSBTAG00000047827	-0.124247427	0.136586071	0.260833498
F6QF86	ALG6	bta:510138; F6QF86	ENSBTAG00000030706	-0.090677668	-0.239165498	-0.148487831
A5PJP1	BLOC1S3	A5PJP1; bta:538539	ENSBTAG00000007070	0.061331843	-0.033358597	-0.09469044
A0A3Q1LJI0	CARD19	A0A3Q1LJI0	ENSBTAG00000011202	0.055853235	0.093702496	0.037849261
F1MWR2	SF3B4	bta:506123; F1MWR2	ENSBTAG00000012495	0.276373786	0.180026391	-0.096347396
A0AAA9T2J5	PKP4	A0AAA9T2J5	ENSBTAG00000013773	0.003174248	-0.046829132	-0.05000338
A5D9A8	SQLE	A5D9A8; bta:526535	ENSBTAG00000005498	0.077626565	-0.237549434	-0.315176
A5PKJ7	RND3	A5PKJ7; bta:783333	ENSBTAG00000039731	0.072862873	0.289063461	0.216200588
A0A3Q8TMT3	NCR3LG1	A0A3Q8TMT3		0.162587844	0.06440745	-0.098180394
A0AAA9T6T6	MED13L	A0AAA9T6T6; bta:517336	ENSBTAG00000001888	0.364354048	0.28614352	-0.078210528
A0AAA9S0R0	PCOLCE	A0AAA9S0R0	ENSBTAG00000020528	-0.248428073	-0.129783576	0.118644496
A5D7P0	CDC25C	A5D7P0; bta:507731		-0.422691072	-0.104451164	0.318239908
E1BEL5	PSMC3IP	E1BEL5	ENSBTAG00000020907	0.275341301	0.118999272	-0.156342029
A0AAA9SQU5				-0.137503524	-0.181606806	-0.044103283
A0AAA9SUY7	DTNBP1	A0AAA9SUY7	ENSBTAG00000012939	0.137840641	-0.080064389	-0.21790503
A0A3Q1M9P7	JADE1	A0A3Q1M9P7	ENSBTAG00000017493	-0.042346291	-0.040812325	0.001533966
A0A0F6QMJ3	C5	A0A0F6QMJ3		-0.175748544	-0.057185347	0.118563197
A0A3Q1MD51	GSDMD	A0A3Q1MD51	ENSBTAG00000021474	-0.285033479	-0.247190128	0.037843351
A0A3Q1MIL2	CDCA3	A0A3Q1MIL2	ENSBTAG00000019777	0.190991348	0.267435106	0.076443757
F1N5P3	PUS3	F1N5P3	ENSBTAG00000014339	-0.156158723	-0.035001596	0.121157126
A5PJK1	TMPPE	A5PJK1; bta:527730		0.063362061	0.040641984	-0.022720077

Accession	Gene_Symbol	Gene_ID	Ensembl_Gene ID	log2(ratio(CM12H/CM0H))	log2(ratio(CM12R/CM0H))	log2(ratio(CM12H/CM12R))
F1N6R4	BNIP2	F1N6R4	ENSBTAG00000013493	-0.214968588	-0.229439071	-0.014470483
Q08DN6	CDC42EP2	bta:507966; Q08DN6	ENSBTAG00000032089	-0.014631921	0.101184634	0.115816555
A0AAA9T8E2	ABCA1	A0AAA9T8E2	ENSBTAG00000020661	-0.219736339	0.231981824	0.451718163
Q2KJF3	NFU1	bta:615964; Q2KJF3	ENSBTAG00000000141	0.013445531	0.344445531	0.331
A0AAF6DMI3	WDR54	A0AAF6DMI3	ENSBTAG00000000489	0.065144025	0.204672791	0.139528765
A0A5H2Q5V2				0.108524457	0.792790294	0.684265838
A0AAF6ZAV2	PROS1	A0AAF6ZAV2	ENSBTAG00000023652	-0.103935596	-0.106812356	-0.002876761
Q5E9Z2	HABP2	bta:507993; Q3MHK6; Q5E9Z2	ENSBTAG00000019322	-0.772316705	-0.407056767	0.365259938
A0AAA9S256	CLCN5	A0AAA9S256	ENSBTAG00000001507	0.240895429	0.088065813	-0.152829616
A0A3Q1MGN4	DSE	A0A3Q1MGN4	ENSBTAG00000005205	-0.239916472	-0.143884211	0.096032261
A0AAA9T6F6	EMC10	A0AAA9T6F6	ENSBTAG00000045510	0.164281423	-0.058722439	-0.223003862
A0A3Q1LV95	LPIN2	A0A3Q1LV95	ENSBTAG00000012060	0.077852145	0.236606281	0.158754135
Q2HJI2	NDUFAF3	bta:511968; Q2HJI2		0.03924954	0.002944277	-0.036305264
A0AAA9S9Y9	MEAF6	A0AAA9S9Y9	ENSBTAG00000010506	0.101879614	-0.028129373	-0.130008987
A0A3Q1M8L1	DOCK4	A0A3Q1M8L1	ENSBTAG00000021182	0.02664684	0.087575839	0.060928999
Q2YDM9	LOC780876	bta:780876; Q2YDM9		-0.004312993	0.072779875	0.077092868
F1MMS1	TAF4	F1MMS1	ENSBTAG00000047991	-0.107163495	-0.037602113	0.069561382
Q05B67	FAM210A	bta:511554; Q05B67	ENSBTAG00000009141	0.0371275	0.153957456	0.116829955
A0A3Q1LNF9	KIF18A	A0A3Q1LNF9; bta:537566	ENSBTAG00000002117	-0.067179316	0.320012347	0.387191662
A0AAA9TXR6	NCS1	A0AAA9TXR6	ENSBTAG00000008726	-0.01449957	-0.00965017	0.0048494
A0A3Q1LY14	MAGI1	A0A3Q1LY14	ENSBTAG00000010581	0.006773229	0.043468029	0.0366948
A0AAA9TTA4	FAM234B	A0AAA9TTA4; bta:512120	ENSBTAG00000014322	0.039485087	0	-0.039485087
A0A3Q1MTI0	KANSL3	A0A3Q1MTI0	ENSBTAG00000004497	-0.036603106	-0.0568245	-0.020221394
A0A3Q1LXK7	LOC132344161	A0A3Q1LXK7	ENSBTAG00000049681	-0.093877681	-0.110365804	-0.016488123
P50227	SULT1A1	bta:282485; P50227; Q3T0S9	ENSBTAG00000008635	0.102403855	0.225903698	0.123499842
A0A3Q1NAS1	LOC524576	A0A3Q1NAS1; bta:524576	ENSBTAG00000050417	-0.156749613	-0.319723824	-0.162974211
A0AAA9S8I5	SMYD2	A0AAA9S8I5	ENSBTAG00000013166	-0.069067573	-0.204214771	-0.135147198
A0AAA9TCX1	GPN3	A0AAA9TCX1	ENSBTAG00000005347	0.418156841	0.447458977	0.029302136
F1CYZ1	MEF2D	bta:100336730; F1CYZ1		-0.064795123	-0.060976783	0.003818339
A7YY73	MAFF	A7YY73; bta:617914	ENSBTAG00000021435	-0.329339216	0.001231494	0.33057071
A0A3Q1LX97	CKM	A0A3Q1LX97	ENSBTAG00000013921	-0.247823636	1.499368301	1.747191938
A0AAA9SR01	ACTR5	A0AAA9SR01; bta:100297865	ENSBTAG00000048262	-0.170730302	-0.127874823	0.042855479
A0A140T8A1	HSPB6	A0A140T8A1	ENSBTAG00000018598	0.051183541	0.297650097	0.246466556
A0AAA9TSC2	STXBP4	A0AAA9TSC2	ENSBTAG00000016078	-0.068125833	-0.248104499	-0.179978666
A0A3Q1LT19				-0.264009529	-0.182929923	0.081079606
F1MZF2	DSC2	F1MZF2	ENSBTAG00000040584	-0.158188893	-0.372463987	-0.214275094
F1MUK6	COMMD3	F1MUK6	ENSBTAG00000003589	0.392472493	0.480300139	0.087827646
A2VDR2	ACBD6	A2VDR2; bta:618245		-0.231325546	-0.265636201	-0.034310655
A0AAA9SFB8	AFG1L	A0AAA9SFB8	ENSBTAG00000014592	0.103093493	0.020939338	-0.082154155
A0A3Q1LTF3	HAL	A0A3Q1LTF3	ENSBTAG00000016276	-0.678071905	-0.600522786	0.077549119
E1B7I8	TELO2	E1B7I8	ENSBTAG00000019086	-0.100481393	0.031996564	0.132477957
A0A3Q1MW68	PATJ	A0A3Q1MW68	ENSBTAG00000021975	0.099079774	0.121251155	0.022171381
A3KN05	MAIP1	A3KN05; bta:510566	ENSBTAG00000019231	0.007956059	-0.043189606	-0.051145665
A0A3Q1MDP7	WDR75	A0A3Q1MDP7	ENSBTAG00000003002	-0.200220147	-0.249081024	-0.048860876
A0AAA9SCA8	DGKZ	A0AAA9SCA8	ENSBTAG00000013636	0.017576198	-0.075630263	-0.093206461
Q5EAE2	TADA3	bta:510184; Q5EAE2	ENSBTAG00000007961	0.085841404	-0.086647478	-0.172488882
A0AAA9TL75	DUS4L	A0AAA9TL75	ENSBTAG00000021849	0.091358393	0.095502087	0.004143694
Q71SS4	DERL1	bta:404121; Q1RMX8; Q71SS4	ENSBTAG00000020693	-0.03796785	-0.053161524	-0.015193674
Q2TBI1	NSMCE4A	bta:527312; Q2TBI1	ENSBTAG00000019166	-0.161815932	-0.223030359	-0.061214426
Q17QV2	MON1A	bta:520286; Q17QV2; Q5E9N7	ENSBTAG00000020047	0.133394978	0.225923604	0.092528626
E1BKK9	SELENOM	E1BKK9	ENSBTAG00000031279	-0.047500424	0.028912692	0.076413116
A0A3Q1LVN6	PRDM2	A0A3Q1LVN6	ENSBTAG00000020552	-0.080283802	-0.33689567	-0.256611868
P07995	INHBA	A0JN81; bta:281867; P07995	ENSBTAG00000056438	-0.543420238	-0.760572207	-0.217151969
A0AAA9S209	FAM120B	A0AAA9S209	ENSBTAG00000003884	-0.151676655	-0.094089163	0.057587492
A0A452DIT2	ZDHHC20	A0A452DIT2	ENSBTAG00000006177	0.244880803	0.127629887	-0.117250916
A0AAA9TEE5	DENND1A	A0AAA9TEE5	ENSBTAG00000003610	0.360680498	0.512683592	0.152003093
A0AAA9T2G8	ADGRG1	A0AAA9T2G8	ENSBTAG00000038141	0.024910461	0.008351369	-0.016559092
F1MSS9	BAG4	bta:516353; F1MSS9	ENSBTAG00000013143	0.392722913	0.626928666	0.234205753
B0JYN4	CIAO2A	B0JYN4	ENSBTAG00000002012	0.038360474	-0.010114078	-0.048474551
A0A452DK75	SLC35A4	A0A452DK75	ENSBTAG00000030584	-0.002734968	-0.130008987	-0.127274019
A0AAA9TD16	NDRG2	A0AAA9TD16	ENSBTAG00000000843	0.069577394	0.087189353	0.017611959
G3MWJ1	SFR1	G3MWJ1	ENSBTAG00000047450	-0.161622784	-0.18552597	-0.023903187
A0A452DJ07	PRR14	A0A452DJ07	ENSBTAG00000009099	-0.044438234	-0.032838613	0.011599621
F1N1U0	ATF7	F1N1U0	ENSBTAG00000005863	0.166625354	-0.290255875	-0.456881229
Q1L836				-0.320886062	-0.434628228	-0.113742166
Q1LZ86	ABHD6	bta:505283; Q1LZ86	ENSBTAG00000016615	0.254012557	0.217623177	-0.03638938
Q08DX0	SNX29	bta:518366; Q08DX0		-0.108235749	-0.166299724	-0.058063975
A0AAA9SKQ5	SKA2	A0AAA9SKQ5	ENSBTAG00000021680	-0.122137102	-0.041710252	0.080426851
A0A3Q1LXS8	GGTA1	A0A3Q1LXS8	ENSBTAG00000012090	0.244163632	0.157171813	-0.086991819
A0AAA9RZP1	TMEM259	A0AAA9RZP1	ENSBTAG00000011351	0.034580232	0.073287483	0.038707251
E1BNI4	MBOAT1	bta:541284; E1BNI4	ENSBTAG00000016519	0.117774792	0.035739023	-0.082035769
F1MZ14	GCH1	F1MZ14	ENSBTAG00000040151	0.288070957	0.698252527	0.410181571
A0AAA9SZ35	BRD8	A0AAA9SZ35	ENSBTAG00000008753	-0.235478683	-0.163975735	0.071502948
A0A452DIL7	ERI3	A0A452DIL7	ENSBTAG00000015559	-0.130894067	-0.029747343	0.101146723
G1K200	LOXL4	G1K200	ENSBTAG00000020895	0.029653274	-0.105143504	-0.134796778
E1B8Z0	BRPF3	bta:507996; E1B8Z0	ENSBTAG00000013316	0.157995757	0.287241266	0.12924551
A6QNK8	TAPBPL	A6QNK8; bta:529141		0.070883486	0.135750143	0.064866657
A0AAF6YIN8	SH3RF1	A0AAF6YIN8	ENSBTAG00000001508	0.146418249	0.009803175	-0.136615074
A0AAF6YWK3	SLC43A2	A0AAF6YWK3	ENSBTAG00000011482	-0.526930379	0.147690101	0.67462048
A4FV30	CCDC8	A4FV30; bta:616838		0.082227098	-0.146841388	-0.229068486
Q0P5B2	METTL2	bta:506221; Q0P5B2		0.053925882	0.122827995	0.068902113
Q32PH9	CDC7	bta:516094; F1N271; Q32PH9	ENSBTAG00000016307	-0.01938391	0.081544999	0.100928909
A0AAA9SQ21	PLPP2	A0AAA9SQ21	ENSBTAG00000000717	-0.07617168	-0.142872082	-0.066700401
A0AAA9TIE7	TNFAIP8L1	A0AAA9TIE7	ENSBTAG00000037765	-0.166072676	-0.148863386	0.01720929
A0A3Q1LRV8	RNF14	A0A3Q1LRV8	ENSBTAG00000005249	-0.289231923	-0.29074339	-0.001511467
A0AAA9TKX2	IFT74	A0AAA9TKX2	ENSBTAG00000019568	-0.155892238	-0.046829132	0.109063106
F6RP53	GEM	bta:538437; F6RP53	ENSBTAG00000007596	0.037730389	-0.015036911	-0.0527673
A0AAA9TIC4	SMIM12	A0AAA9TIC4	ENSBTAG00000014235	0.185633871	0.218498505	0.032864634
A0A3Q1MXK6	HINT2	A0A3Q1MXK6	ENSBTAG00000011444	-0.690286094	-0.361276762	0.329009332
E1B8E9	MSL1	bta:787839; E1B8E9	ENSBTAG00000010375	0.246540973	-0.101913416	-0.348454389
A0A3Q1LRX5	LSM6	A0A3Q1LRX5	ENSBTAG00000014060	0.105693961	0.037949354	-0.067744607

Accession	Gene_Symbol	Gene_ID	Ensembl_Gene ID	log2(ratio(CM12H/CM0H))	log2(ratio(CM12R/CM0H))	log2(ratio(CM12H/CM12R))
A6QP01	SELENOT	A6QP01; bta:783831		-0.034440645	0.056583528	0.091024174
A0A3Q1MS65	TAF6	A0A3Q1MS65	ENSBTAG00000030966	-0.144248253	-0.135351853	0.0088964
A0AAA9TQ82	KIAA0586	A0AAA9TQ82	ENSBTAG00000004631	-0.415037499	-0.236130222	0.178907277
A0AAA9SLG7	YPEL5	A0AAA9SLG7	ENSBTAG00000023744	0.094250326	0.077041036	-0.01720929
Q08DA5	PANK3	bta:510749; Q08DA5	ENSBTAG00000011895	0.035662198	-0.209110001	-0.244772199
A0A3Q1LLF3	BOLA3	A0A3Q1LLF3	ENSBTAG00000050675	0.040406462	-0.032871146	-0.073277607
A0AAA9TU07	GPX3	A0AAA9TU07	ENSBTAG00000043553	-0.248970655	-0.64201719	-0.393046535
Q148D6	MPDU1	bta:504961; Q148D6		-0.083532623	-0.130245368	-0.046712745
A0A3Q1NJR3	PEX16	A0A3Q1NJR3	ENSBTAG00000003126	-0.202003514	-0.292658553	-0.090655038
A0AAA9U0M9				-0.346175641	-0.171368418	0.174807223
E1BLR4	RHBDF2	E1BLR4	ENSBTAG00000003436	-0.258734268	0.073462162	0.33219643
F1MYF2	MMGT1	F1MYF2	ENSBTAG00000025297	0.075704083	-0.091474005	-0.167178087
A0AAA9TJA6	HMG20B	A0AAA9TJA6	ENSBTAG00000008789	-0.691800425	-0.571960655	0.11983977
A0AAA9RX88	CENPN	A0AAA9RX88	ENSBTAG00000011635	0.089916637	0.029084492	-0.060832144
A0AAF6YS06	TONSL	A0AAF6YS06	ENSBTAG00000007749	0.013044348	-0.137372495	-0.150416843
G3N340	DUS1L	G3N340	ENSBTAG00000047760	-0.074723918	-0.127780992	-0.053057074
E1BGZ3	ARID3A	bta:511283; E1BGZ3	ENSBTAG00000008607	-0.108856302	-0.079108958	0.029747343
A0AAA9THC7	WWC3	A0AAA9THC7	ENSBTAG00000013824	0.044394119	-0.100886755	-0.145280874
E1BI78	YBEY	E1BI78	ENSBTAG00000003161	-0.333950623	-0.274815499	0.059135124
A0AAA9SQR2				-0.21326141	0.059600142	0.272861552
F1MSQ6	NEFH	bta:528842; F1MSQ6	ENSBTAG00000013147	-0.525948341	-0.649640558	-0.123692217
Q76LV2	HSP90AA1	bta:281832; Q76LV2		0.617912923	0.560957038	-0.056955885
Q3ZC04	MRPL57	bta:615666; Q3ZC04		0.038665109	-0.027843633	-0.066508742
A0AAF6Z9T9	FSTL3	A0AAF6Z9T9	ENSBTAG00000003018	0.06058998	-0.055876498	-0.116466478
A0A3Q1MVF2	SLC6A6	A0A3Q1MVF2	ENSBTAG00000011088	-0.086348705	-0.144296054	-0.057947349
F1ME22	ADAM19	bta:510907; F1ME22	ENSBTAG00000003836	-0.58162899	-0.725568339	-0.143939349
A0A3Q1MFX1	SENP1	A0A3Q1MFX1	ENSBTAG00000012423	0.348172194	0.257797757	-0.090374437
E1BF46	ZNF407	bta:530360; E1BF46	ENSBTAG00000022887	0.032543373	0.023127148	-0.009416225
A4IFA0	GAS2L1	A4IFA0; bta:518935		-0.067434361	0.065707852	0.133142212
P10575	GLRX	bta:515416; P10575; Q32KQ3		0.253820081	0.179298138	-0.074521943
F1MTX9	SLC38A1	F1MTX9	ENSBTAG00000000859	-0.249132269	-0.259185934	-0.010053665
P0CB32	HSPA1L	bta:540190; P0CB32	ENSBTAG00000025442	0.903410027	0.733485025	-0.169925001
P80221	CXCL6	A5PJH0; bta:281735; P80221; Q9GKP5	ENSBTAG00000009812	-0.13489161	-0.520116676	-0.385225067
Q0P5L5	SUMF1	bta:536435; Q0P5L5	ENSBTAG00000039855	-0.057030945	-0.209608703	-0.152577758
E1BA23	AMPD3	E1BA23	ENSBTAG00000015821	0.030098131	0.231619374	0.201521243
A0AAA9S7Y7	THBS2	A0AAA9S7Y7	ENSBTAG00000018810	0.57797607	0.168220329	-0.409755741
F1MN32	ITPKC	F1MN32	ENSBTAG00000019465	-0.224458827	-0.21164902	0.012809807
F1MKZ2	ASAP2	F1MKZ2	ENSBTAG00000002329	-0.11783649	-0.161835683	-0.043999193
E1BJZ4	GCC1	E1BJZ4	ENSBTAG00000003102	-0.1812974	-0.258691116	-0.077393716
A2VDL2	SLC2A3	A2VDL2; bta:282358	ENSBTAG00000004556	0.113753714	-0.14102659	-0.254780305
A0AAA9TAP8	CBLL1	A0AAA9TAP8	ENSBTAG00000018348	0.202308175	0.078818747	-0.123489428
F1MWU9	HSPA6	F1MWU9	ENSBTAG00000039035	2.242437501	1.001486548	-1.240950953
Q5E975	TMEM230	bta:515498; Q5E975	ENSBTAG00000006063	0.195550809	0.085819368	-0.109731441
A0A3Q1M2Z5	TGFB2	A0A3Q1M2Z5	ENSBTAG00000005359	0.007533673	0.056777192	0.049243519
Q1JPA6	NAT1	bta:512603; Q1JPA6	ENSBTAG00000016473	0.040733791	0.094348301	0.05361451
A0A3Q1LKR3	AADAT	A0A3Q1LKR3	ENSBTAG00000050967	-0.159036685	-0.111031312	0.048005373
E1BNC4	DIPK2A	bta:509304; E1BNC4	ENSBTAG00000007933	0.177035241	0.069283989	-0.107751253
A0AAA9SI10	LDAH	A0AAA9SI10	ENSBTAG00000031014	0.153717711	0.123970368	-0.029747343
G3N1U1	CENPV	G3N1U1	ENSBTAG00000013274	0.230761285	0.122989967	-0.107771318
Q3T149	HSPB1	Q3T149	ENSBTAG00000011969	0.847996907	0.789493188	-0.058503719
A6H720	NCK2	A6H720; bta:526430	ENSBTAG00000015205	0.14172811	-0.030911277	-0.172639386
A0AAA9TA99				0.282884182	-0.068845707	-0.351729889
Q5EAC9	HSPB8	bta:539524; Q3T0Q5; Q5EAC9	ENSBTAG00000001303	1.419956342	1.204259331	-0.215697011
A0A3Q1M2G9	LGALS8	A0A3Q1M2G9	ENSBTAG00000013544	0.073309764	-0.195278535	-0.268588299
O18824	SCARB1	bta:282346; O18824	ENSBTAG00000014269	0.087137654	0.174217827	0.087080174
E1BQ22	TRIM68	bta:538657; E1BQ22	ENSBTAG00000011332	-0.20571368	0.070161071	0.275874752
A0A3Q1M2J3	CANT1	A0A3Q1M2J3	ENSBTAG00000009991	0.105493129	0.196371369	0.090878241
A0AAA9T599	UBXN2B	A0AAA9T599	ENSBTAG00000009138	0.342524999	0.215099929	-0.127425071
P82927	MRPL42	bta:614148; P82927; Q32LA9	ENSBTAG00000002708	0	-0.102044206	-0.102044206
A0AAA9TU40	AKT1S1	A0AAA9TU40	ENSBTAG00000005763	0.155410844	-0.032864634	-0.188275478
Q05B70	GRAMD3	bta:505627; Q05B70		0.196118942	0.091630475	-0.104488467
Q0IIM3	HSPH1	bta:507165; Q0IIM3	ENSBTAG00000005012	0.881139959	0.716452997	-0.164686962
A0AAA9T723	C20H5orf22	A0AAA9T723	ENSBTAG00000017569	0.186607846	0.204843418	0.018235571
Q0VBY5	WDR41	bta:514919; Q0VBY5	ENSBTAG00000020212	-0.25115502	-0.213286835	0.037868186
Q3SZU5	SPCS3	bta:767917; Q3SZU5		0.035813418	0.118065012	0.082251594
A0AAA9SG01	ERN1	A0AAA9SG01	ENSBTAG00000016799	-0.177482962	0.131799077	0.309282038
Q08DX7	SPNS1	bta:505997; Q08DX7	ENSBTAG00000014653	0.212699025	0.247927513	0.035228488
A0AAA9RXW3	ALG5	A0AAA9RXW3	ENSBTAG00000011850	0.111692872	0.00297463	-0.108718242
F6QLT0				0.302903269	-0.286534691	-0.58943796
A0AAA9SLB4	YIPF6	A0AAA9SLB4	ENSBTAG00000001977	-0.12683708	-0.118308808	0.008528271
A0AAA9TN79	IMP3	A0AAA9TN79	ENSBTAG00000069591	-1.460722291	-1.323403243	0.137319048
A0A3Q1LUT5	PTPRA	A0A3Q1LUT5	ENSBTAG00000011425	-0.076102979	-0.041945968	0.034157011
Q58D98	POLR3D	bta:508049; Q58D98		0.045928687	-0.073860235	-0.119788921
Q17QM4	SELENOI	bta:506381; Q17QM4	ENSBTAG00000007837	0.007217099	-0.041096839	-0.048313938
E1BGW4	USP36	bta:504581; E1BGW4	ENSBTAG00000021505	-0.087056734	-0.209453366	-0.122396631
A0AAA9TK62	EXTL2	A0AAA9TK62	ENSBTAG00000019026	0.023207391	0.077797278	0.054589886
Q1JPF1	NKIRAS2	bta:509053; Q1JPF1	ENSBTAG00000002657	-0.080022083	-0.063658637	0.016363446
A0AAA9SI71	LIAS	A0AAA9SI71	ENSBTAG00000014520	-0.044981382	-0.031201955	0.013779427
A0A3Q1N4J1	LARP1B	A0A3Q1N4J1	ENSBTAG00000012135	-0.059545942	0.044825199	0.104371141
A0AAA9SW74	SNTA1	A0AAA9SW74	ENSBTAG00000000512	-0.027638681	-0.134768556	-0.107129875
A0AAA9RU66	CD82	A0AAA9RU66	ENSBTAG00000031252	0.115923554	0.190031236	0.074107682
A0A3Q1LM90	ATP11B	A0A3Q1LM90; bta:614392	ENSBTAG00000000290	0.072900547	0.097775216	0.024874669
F1MZJ7	CCDC85C	F1MZJ7	ENSBTAG00000025181	0.03500645	-0.159050958	-0.194057408
A0AAA9T585	HACL1	A0AAA9T585	ENSBTAG00000003718	-0.184424571	-0.109624491	0.07480008
A0AAA9S463	COX10	A0AAA9S463	ENSBTAG00000015294	-0.033280217	-0.35614381	-0.322863594
Q3T061	COX7A2L	bta:540225; Q3T061	ENSBTAG00000000705	-0.146699062	-0.174915304	-0.028216242
A0AAA9SXK1	GLIPR1	A0AAA9SXK1	ENSBTAG00000011590	-0.108993184	-0.061270143	0.047723041
A0AAF6YGN5	TUSC2	A0AAF6YGN5	ENSBTAG00000058889	0.031767046	0.062849649	0.031082604
A0A3S5ZPJ2	TNIP1	A0A3S5ZPJ2	ENSBTAG00000012671	-0.069132481	0.086060729	0.15519321
F6RWD2	DHDDS	F6RWD2	ENSBTAG00000003235	-0.290862279	-0.448346175	-0.157483896

Accession	Gene_Symbol	Gene_ID	Ensembl_Gene ID	log2(ratio(CM12H/CM0H))	log2(ratio(CM12R/CM0H))	log2(ratio(CM12H/CM12R))
A0AAA9TD35	TMEM181	A0AAA9TD35	ENSBTAG00000063575	-0.159940913	0.019460314	0.179401227
A0A3Q1LL22	ASXL2	A0A3Q1LL22; bta:100140419	ENSBTAG00000019983	-0.116090217	-0.181307877	-0.065217659
A0AAA9TZT6	DPH1	A0AAA9TZT6	ENSBTAG00000009376	-0.336496568	-0.375785699	-0.039289131
E1BKA3	AJUBA	bta:519937; E1BKA3	ENSBTAG00000012724	0.422764861	-0.386903927	-0.809668788
G3MXX2	SERPINA7	G3MXX2	ENSBTAG00000047655	-0.366538982	0.238159737	0.604698719
A0A452DI74	CALCOCO2	A0A452DI74	ENSBTAG00000007935	0.561326942	0.06322098	-0.498105961
A0A3Q1MAU4	FASTKD5	A0A3Q1MAU4	ENSBTAG00000051021	-0.285754482	-0.113287286	0.172467196
F1MKI1	ATP7B	bta:518075; F1MKI1	ENSBTAG00000010353	-0.291733244	-0.485045464	-0.19331222
A0A3Q1LGR3	KDM1B	A0A3Q1LGR3	ENSBTAG00000014608	0.129368004	0.185376139	0.056008135
Q9GJW7	GABARAP	bta:327715; Q3SZ82; Q9GJW7	ENSBTAG00000014883	0.55356302	0.020735609	-0.532827411
E1BJ69	KNSTRN	E1BJ69	ENSBTAG00000032340	0.123477395	0.124806456	0.00132906
A0A452DJ15	MRPL27	A0A452DJ15	ENSBTAG00000021720	0.029943802	-0.098161024	-0.128104826
A0AAA9RSH3	IMPACT	A0AAA9RSH3	ENSBTAG00000003035	-1.120433786	-0.879962554	0.240471233
Q32PI9	MPZL1	A2SZV4; bta:539387; Q32PI9	ENSBTAG00000002823	0.12566765	-0.086232398	-0.211900048
A0AAA9SJD1	AGL	A0AAA9SJD1	ENSBTAG00000006491	-0.021835801	0.104480649	0.12631645
Q0VCF8	SOX4	bta:768313; Q0VCF8	ENSBTAG00000051245	0.237394585	-1.031621979	-1.269016564
A0AAA9S9S8	SLC35F6	A0AAA9S9S8	ENSBTAG00000018761	0.13498451	0.028931002	-0.106053508
Q148I0				0.174460763	-0.624186685	-0.798647448
E1BL88	ABCB10	E1BL88	ENSBTAG00000017053	0.034351505	-0.014292088	-0.048643592
A0A3Q1MET8	FKRP	A0A3Q1MET8	ENSBTAG00000050243	-0.108441812	-0.076020334	0.032421478
F1MVT1	FLRT2	bta:539905; F1MVT1	ENSBTAG00000064499	0	-0.055801792	-0.055801792
A0A0S2I887	L3MBTL3	A0A0S2I887		0.001368132	-0.047305715	-0.048673847
F1N7N4	ADAMTS16	F1N7N4	ENSBTAG00000018460	-0.058767408	0.149576356	0.208343764
G3MYQ0	STYX	bta:616825; G3MYQ0	ENSBTAG00000046956	-0.046866605	-0.321928095	-0.27506149
A0AAA9T6H1	MIS18BP1	A0AAA9T6H1	ENSBTAG00000002613	-0.240481049	-0.1553573	0.085123749
G5E5D9	NKAP	G5E5D9	ENSBTAG00000010884	0.111611991	0.04781148	-0.063800511
F1N180	MOB2	F1N180	ENSBTAG00000011249	0.191107843	0.159817615	-0.031290228
A0A3Q1M2U6	TMEM87B	A0A3Q1M2U6	ENSBTAG00000012099	-0.209415765	-0.161526753	0.047889012
Q9GKZ4	TRAM1	bta:281546; Q3SX16; Q9GKZ4	ENSBTAG00000007562	0.170579137	0.153474481	-0.017104656
A0AAF6YKL2	IMP4	A0AAF6YKL2	ENSBTAG00000003225	-0.138125956	-0.446139554	-0.308013597
A0AAA9S2W8	AMFR	A0AAA9S2W8	ENSBTAG00000003798	0.186272996	-0.072703194	-0.258976189
A7MB15	FZD1	A7MB15; bta:445417	ENSBTAG00000002107	0.091512619	0.193283297	0.101770678
Q32L67	GLRX2	bta:513762; Q32L67	ENSBTAG00000015972	-0.396453794	-0.138626195	0.257827599
E1BBA1	GGNBP2	E1BBA1	ENSBTAG00000010290	-0.274089594	-0.367297648	-0.093208054
A0AAA9TKC4	KYAT1	A0AAA9TKC4	ENSBTAG00000036101	-0.066776478	-0.053466025	0.013310454
E1BAX6	CYP7B1	E1BAX6	ENSBTAG00000001299	-0.511804503	-0.698016842	-0.186212339
A0AAF6Z257	IRAK1	A0AAF6Z257	ENSBTAG00000016085	0.955261685	0.686308055	-0.26895363
Q5EAE6	DAP	bta:616066; Q3SYY4; Q5EAE6	ENSBTAG00000006346	0.026607835	-0.248487095	-0.27509493
A0AAA9STX7				0.020199614	0.202586	0.182386386
A4IFD8	METTL14	A4IFD8; bta:531382		0.017996429	-0.020671079	-0.038667508
A0A3Q1NBI1	VAV3	A0A3Q1NBI1	ENSBTAG00000031575	-0.068961624	-0.014355293	0.054606331
A0A3Q1MUW9	FBXL20	A0A3Q1MUW9	ENSBTAG00000008371	0.197669992	0.215874587	0.018204595
Q5EA45	FOXRED1	bta:510097; Q58CP5; Q5EA45; Q5EAA3		0.136745208	0.174181015	0.037435807
F1MCM1	MTRF1L	F1MCM1	ENSBTAG00000009275	-0.245420207	-0.189089207	0.056331
Q1LZH1	MTARC2	bta:615506; Q1LZH1	ENSBTAG00000016277	-0.094035495	0.953748276	1.047783771
Q08D81	WDR13	bta:523881; Q08D81	ENSBTAG00000018060	-0.17154328	-0.092160859	0.079382421
E1B899	MIOS	bta:512321; E1B899	ENSBTAG00000013781	-0.176308215	-0.196184925	-0.01987671
A0A3Q1LNT8	EML1	A0A3Q1LNT8	ENSBTAG00000013491	-0.021373651	-0.018983103	0.002390547
Q3MHV9	SERINC1	bta:539993; Q3MHV9	ENSBTAG00000000019	-0.015320799	0.011384763	0.026705563
A0AAF7AF97	DYNC2LI1	A0AAF7AF97	ENSBTAG00000013676	-0.279931475	-0.270961322	0.008970154
A0A3Q1NM28	CEMIP	A0A3Q1NM28	ENSBTAG00000007431	-0.296091271	0.090621245	0.386712515
G3N2E9	KDM6A	bta:280952; G3N2E9	ENSBTAG00000003740	0.00823615	0.055365237	0.047129087
Q3SX20	BORCS6	bta:539395; Q3SX20		0	0.016713874	0.016713874
F1MXW5	OXSM	F1MXW5	ENSBTAG00000007031	-0.114306328	0	0.114306328
Q0VCU3	CTSF	bta:509715; Q0VCU3	ENSBTAG00000010994	0.382015245	0.215267987	-0.166747258
A0AAF7ABH3	BMI1	A0AAF7ABH3	ENSBTAG00000015584	0.072116992	0.041090096	-0.031026896
F6RWX3	SEC61B	F6RWX3	ENSBTAG00000002457	0.070009721	-0.051385386	-0.121395108
F1N3J6	PIGW	F1N3J6	ENSBTAG00000034396	0.007371989	0.020547377	0.013175389
Q2KIR4	RPP25L	bta:512203; Q2KIR4	ENSBTAG00000016295	0.077459489	-0.075630263	-0.153089752
A0A3Q1MDR6	CBX4	A0A3Q1MDR6	ENSBTAG00000049156	0.480475396	0.022390957	-0.458084439
A0A3Q1M0N7	ECE1	A0A3Q1M0N7	ENSBTAG00000002977	0.253968597	0.357632157	0.103663559
A0A3Q1MJW8	C28H1orf131	A0A3Q1MJW8	ENSBTAG00000010765	0.342045141	-0.072992358	-0.415037499
A4IFE2	RCHY1	A4IFE2; bta:540733	ENSBTAG00000007189	0.50701078	-0.635906966	-1.142917746
Q5E9J5	DHCR7	A6QR24; bta:514745; Q5E9J5	ENSBTAG00000016465	-0.089386318	-0.194243632	-0.104857314
A5D7I4	EXT1	A5D7I4; bta:538602	ENSBTAG00000006209	-0.273699814	-0.209283086	0.064416728
A0AAA9TUJ0	RETREG1	A0AAA9TUJ0	ENSBTAG00000016444	0.028826035	-0.091654341	-0.120480376
F1MH50	PLEKHH2	bta:100299044; F1MH50	ENSBTAG00000032521	0.098092812	0.003324184	-0.094768628
A0AAA9SJN1	TMEM63A	A0AAA9SJN1	ENSBTAG00000002854	-0.032256721	0.049875907	0.082132627
A0A3Q1M5S1	GTF2H3	A0A3Q1M5S1	ENSBTAG00000007270	0.108307095	0.146744631	0.038437536
E1BC04	CNST	bta:541076; E1BC04	ENSBTAG00000000511	-0.210406517	-0.236638059	-0.026231542
A1A4L1	THEM4	A1A4L1; bta:787270		-0.239007135	-0.182368064	0.056639071
A0A3Q1MBH1	MND1	A0A3Q1MBH1	ENSBTAG00000000281	-0.039979438	-0.122161082	-0.082181644
A0AAF6YSH2	LIMD2	A0AAF6YSH2	ENSBTAG00000008154	-0.286145928	-0.296734563	-0.010588635
F1MEZ4	CCDC82	F1MEZ4	ENSBTAG00000006581	0.495868574	-0.066726114	-0.562594688
A0AAA9T7B5	WIPI2	A0AAA9T7B5	ENSBTAG00000012049	0.095952955	0.139424458	0.043471503
J9JH84	RNF169	J9JH84	ENSBTAG00000000727	0.302856542	-0.009347949	-0.312204491
Q1JPG0	MIGA2	bta:535315; Q1JPG0	ENSBTAG00000010653	0.203032746	-0.038553241	-0.241585987
E1BCF2	CRLF3	E1BCF2	ENSBTAG00000018381	0.313826296	0.206643224	-0.107183072
A0AAA9TN88	ATG13	A0AAA9TN88	ENSBTAG00000017325	0.362386238	0.240244297	-0.122141941
A0AAA9TV41	PHF19	A0AAA9TV41	ENSBTAG00000016958	-0.20461772	-0.102946302	0.101671418
F1MD95	CLSTN1	F1MD95	ENSBTAG00000011823	-0.042770772	-0.161870123	-0.119099351
A0A3S5ZPL0	GNB5	A0A3S5ZPL0	ENSBTAG00000003762	-0.042125476	-0.113316524	-0.071191048
A0AAA9S597	GNPTG	A0AAA9S597	ENSBTAG00000014118	-0.022185707	0	0.022185707
F6PX95	ESS2	F6PX95	ENSBTAG00000018534	0.034992453	-0.122190879	-0.157183333
Q2KJ19	DR1	bta:613828; Q2KJ19	ENSBTAG00000008737	0.007312205	-0.076781234	-0.08409344
A0A3Q1MIF8	NFIX	A0A3Q1MIF8	ENSBTAG00000018229	-0.016993071	-0.125223762	-0.108230691
A5D7I7	RASA3	A5D7I7; bta:282532; F1N4G2		-0.094797755	-0.181838323	-0.087040568
E1BB75	EAF1	E1BB75	ENSBTAG00000019202	0.056207105	0.004187797	-0.052019308
Q3SWW7	IFRD2	bta:767939; Q3SWW7		-0.197639988	-0.174476955	0.023163033
F1MRH9	PTPN21	F1MRH9	ENSBTAG00000018596	0.13117429	-0.031026896	-0.162201185

Accession	Gene_Symbol	Gene_ID	Ensembl_Gene ID	log2(ratio(CM12H/CM0H))	log2(ratio(CM12R/CM0H))	log2(ratio(CM12H/CM12R))
P00257	FDX1	A5D9I2; bta:281157; P00257; P08498; P12713; Q32KZ0	ENSBTAG00000011793	0.109794142	0.162952078	0.053157936
A0AAA9TNA0	SH2B1	A0AAA9TNA0	ENSBTAG00000019219	0.040684436	0.199505992	0.158821556
A0AAA9TGF0	SENP6	A0AAA9TGF0	ENSBTAG00000005869	-0.129166021	-0.151171329	-0.022005308
A0AAF6DLT7	TUBGCP5	A0AAF6DLT7	ENSBTAG00000014714	-0.039528364	-0.022454851	0.017073513
F1MDC9	PRKCE	bta:507041; F1MDC9	ENSBTAG00000020614	-0.119713555	-0.051676035	0.068037521
Q2KIA4	SCD5	bta:617419; Q2KIA4	ENSBTAG00000022449	-0.098762657	-0.245039603	-0.146276946
A0AAA9S414	GABARAPL1	A0AAA9S414	ENSBTAG00000011765	0.57392845	0.084064265	-0.489864186
A0A3Q1LGW7	KRT76	A0A3Q1LGW7; bta:100336907	ENSBTAG00000038033	-0.564174028	-0.468358241	0.095815787
A0AAA9RRX3	RPE	A0AAA9RRX3	ENSBTAG00000054647	0.481314385	1.004484601	0.523170216
A0AAA9TVC3	CPTP	A0AAA9TVC3	ENSBTAG00000007623	0.005846796	0.010216396	0.0043696
Q32KP1	TSPAN31	bta:510619; Q32KP1	ENSBTAG00000007158	0.167727446	0.014746585	-0.15298086
Q2T9N4	PMF1	bta:616311; Q2T9N4	ENSBTAG00000009432	-0.042204703	-0.225961978	-0.183757275
A0A3Q1LLU2	RIN2	A0A3Q1LLU2	ENSBTAG00000020382	-0.076058078	-0.183628804	-0.107570726
A0AAA9TRF8				0.284511131	-0.225137798	-0.509648929
E1BNL6	GMEB2	E1BNL6	ENSBTAG00000014769	0.095596572	0.107968497	0.012371925
Q2HJB0	MBLAC1	bta:505636; Q2HJB0		-0.016103308	-0.03387801	-0.017774702
A0A3Q1MJ94	SLC38A9	A0A3Q1MJ94	ENSBTAG00000033313	0	0.115254802	0.115254802
A0AAA9TSJ0	STK19	A0AAA9TSJ0	ENSBTAG00000005589	0.191955947	0.060711414	-0.131244533
Q3B7N1	CHD1L	bta:524787; Q3B7N1		-0.215728691	-0.165568596	0.050160095
A0A3Q1LT06	TCP11L1	A0A3Q1LT06; bta:508910	ENSBTAG00000008613	0.141399867	0.398435532	0.257035665
A0A3Q1N6M3	MTNAP1	A0A3Q1N6M3	ENSBTAG00000006089	0.047628863	0.139930261	0.092301398
A0AAF6Z521	CA4	A0AAF6Z521	ENSBTAG00000017969	-0.019691848	0.222392421	0.242084269
A0AAA9SRI3	MZT2B	A0AAA9SRI3	ENSBTAG00000014646	-0.289357231	-0.643268072	-0.353910841
A0A3Q1MMK5	SNF8	A0A3Q1MMK5	ENSBTAG00000018803	0.042855479	0.111568229	0.06871275
A0AAA9SW54	MAP1LC3A	A0AAA9SW54	ENSBTAG00000006135	0.147313305	-0.023602899	-0.170916204
A0AAA9TT39	PPP1R11	A0AAA9TT39	ENSBTAG00000032247	0.385481178	0.406717183	0.021236005
Q56K13	SF3B5	bta:574400; Q56K13	ENSBTAG00000060547	-0.26960706	0.34684947	0.61645653
A5D962	PHF23	A5D962; bta:539774; Q58CU8	ENSBTAG00000014881	-0.023124725	-0.071855708	-0.048730983
A0AAA9U0T8				0.480068622	-0.053111336	-0.533179959
Q3SZ87	SSR3	bta:767980; Q3SZ87	ENSBTAG00000018471	0.128642151	0.099635338	-0.029006813
Q3T0E3	COA3	bta:539832; Q3T0E3	ENSBTAG00000019911	0.181776151	0.043439261	-0.13833689
A0AAA9TF96	POLE2	A0AAA9TF96	ENSBTAG00000004931	-0.291655084	-0.150572558	0.141082526
F6RM69	FZR1	F6RM69	ENSBTAG00000031387	0.24564451	0.125917819	-0.11972669
E1BDF7	ATXN1	E1BDF7	ENSBTAG00000019675	0.007246098	-0.111728856	-0.118974954
Q2HJF1	MRPL53	bta:505728; Q2HJF1	ENSBTAG00000016599	-0.227898898	-0.255751018	-0.027852121
A0A3Q1M9J6	RIC8B	A0A3Q1M9J6; bta:522873	ENSBTAG00000000147	0.096612649	-0.012371925	-0.108984573
Q9XS50	VEGFC	bta:282122; Q9XS50	ENSBTAG00000014737	0.097658608	-0.213255358	-0.310913966
A1A4R3	DNAJC15	A1A4R3; bta:618047		-0.215663421	-0.517756334	-0.302092913
A0A3Q1MBI9	THBD	A0A3Q1MBI9	ENSBTAG00000048591	0.137910356	-0.262736051	-0.400646407
A1A4J3	ZCCHC3	A1A4J3; bta:507116	ENSBTAG00000002116	-0.570619618	-0.249808881	0.320810737
F1MMD7	ITIH4	F1MMD7	ENSBTAG00000007850	-0.754043232	-0.525902516	0.228140716
F1MKU2	CBX2	F1MKU2	ENSBTAG00000038306	-0.072979445	-0.133788358	-0.060808912
A0AAF6Z1X8	TFPI2	A0AAF6Z1X8	ENSBTAG00000015844	-0.158448503	-0.314977911	-0.156529408
A0A3Q1MJ30	CERS6	A0A3Q1MJ30	ENSBTAG00000044179	0.035189428	-0.004116108	-0.039305537
A0A3Q1M496	ENTPD4	A0A3Q1M496	ENSBTAG00000006768	0.011227255	0.018200179	0.006972923
A0AAA9T9Y0	JMJD1C	A0AAA9T9Y0	ENSBTAG00000001573	-0.925905557	-0.765722487	0.16018307
A0AAA9TLF7	C8orf33	A0AAA9TLF7	ENSBTAG00000000879	-0.107579887	-0.218797366	-0.111217479
A0AAA9S3C7	KIAA0930	A0AAA9S3C7	ENSBTAG00000006776	-0.11897749	0.006975171	0.125952661
E1BKM5	RECQL5	bta:512590; E1BKM5	ENSBTAG00000011715	0.025560226	-0.059968589	-0.085528816
A0AAF6Z1T1	COQ5	A0AAF6Z1T1	ENSBTAG00000015761	-0.373891517	-0.155030786	0.21886073
F1MPI8	CDK8	F1MPI8	ENSBTAG00000016737	-0.192141256	0.365702119	0.557843374
A0AAA9SJD5	NOPCHAP1	A0AAA9SJD5	ENSBTAG00000021196	0.069891933	0.118801533	0.0489096
A0A3Q1MT65	PAM	A0A3Q1MT65	ENSBTAG00000012387	-0.419728021	-0.958457738	-0.538729717
A0AAA9TCI2	HMGXB4	A0AAA9TCI2	ENSBTAG00000010533	-0.047123912	-0.161427539	-0.114303627
A0A3Q1LKV4	KSR1	A0A3Q1LKV4	ENSBTAG00000006601	-0.077100968	-0.083536793	-0.006435825
E1BB99	EPC1	E1BB99	ENSBTAG00000001552	-0.043319038	-0.073421069	-0.030102032
Q1JPJ0	NDOR1	bta:513873; G3MXY0; Q1JPJ0		0.126386754	0.082706747	-0.043680007
A0A3Q1M5L8	ZFYVE19	A0A3Q1M5L8	ENSBTAG00000013485	0.3293839	0.227721925	-0.101661975
A0AAA9SAS8	FAM76B	A0AAA9SAS8; bta:519943	ENSBTAG00000020748	0	0.051768062	0.051768062
Q3ZC02	DCTN5	bta:506441; Q3ZC02	ENSBTAG00000006410	0.019628807	0.029343962	0.009715155
A0AAA9S2M3	TTC39C	A0AAA9S2M3; bta:532895	ENSBTAG00000005635	-0.131840054	-0.001924877	0.129915177
A0A3Q1LWI1	FKBP14	A0A3Q1LWI1	ENSBTAG00000007870	0.14597268	0.318439876	0.172467196
G3N184	NDEL1	bta:510769; G3N184	ENSBTAG00000011994	-0.111628207	-0.101988784	0.009639424
A0AAA9S208	DENND5B	A0AAA9S208	ENSBTAG00000020315	0.12799293	0.271568792	0.143575862
A0FDH2	APOBEC3F	A0FDH2		-0.078923105	-0.05057229	0.028350815
A0AAA9TE30	DALRD3	A0AAA9TE30	ENSBTAG00000018913	-0.147421027	-0.233250937	-0.08582991
A0AAA9SB40	LOC784488	A0AAA9SB40	ENSBTAG00000060244	0.258042376	0.085614868	-0.172427509
Q0VCU4	CD247	Q0VCU4		0.082086703	0.421779077	0.339692374
F1MUQ6	PRR11	F1MUQ6	ENSBTAG00000021681	-0.178160832	0.125754331	0.303915162
A0AAA9S1F6	ANKRD52	A0AAA9S1F6; bta:521057	ENSBTAG00000011762	0.082806479	0.20614092	0.123334442
F6RUM1	BUD13	F6RUM1	ENSBTAG00000016855	0.181592456	0.094276701	-0.087315755
F1N535	NCAPG2	F1N535	ENSBTAG00000016131	-0.178059277	0.031347944	0.209407221
A0AAA9THX1	ATP5MF	A0AAA9THX1	ENSBTAG00000002094	0.065687903	-0.040898031	-0.106585934
A0AAA9TA05	GEMIN2	A0AAA9TA05	ENSBTAG00000020930	0.034215715	0.182692298	0.148476582
A0AAA9SQK2	MARVELD2	A0AAA9SQK2	ENSBTAG00000040001	0.093396536	0.182462888	0.089066352
A0AAA9TWY3	NFATC3	A0AAA9TWY3	ENSBTAG00000004368	0.14650654	0.411325907	0.264819367
F1N6L6	FAM76A	F1N6L6	ENSBTAG00000019614	-0.149612546	-0.105701046	0.0439115
Q2KJ84	TMED5	A5D9A9; bta:534351; Q2KJ84	ENSBTAG00000020180	0.343907138	0.273394917	-0.07051222
F6RG44	TTI2	F6RG44	ENSBTAG00000015463	0.14939895	-0.106615028	-0.256013978
P15103	GLUL	bta:281199; O02850; P15103; Q3ZBU6; Q866Q7	ENSBTAG00000013631	-0.202354668	-0.325174566	-0.122819898
F1N1F1	CDAN1	bta:538940; F1N1F1	ENSBTAG00000005751	-0.108269212	-0.196240133	-0.087970922
A2VDR1	RAB40C	A0A8J8XK94; A2VDR1; bta:618031; M5FMU8		0.182228462	-0.029963591	-0.212192053
A0AAA9TQD4	UBE2J2	A0AAA9TQD4	ENSBTAG00000015882	0.288538365	-0.221062135	-0.509600501
A0AAA9SAF0	CDCA7L	A0AAA9SAF0	ENSBTAG00000004976	-0.096144045	-0.451316075	-0.355172031
A0AAA9TNL9	SGF29	A0AAA9TNL9	ENSBTAG00000016883	-0.339016053	-0.160250564	0.178765489
E1BNM5	ISG20	bta:506604; E1BNM5	ENSBTAG00000014762	0.146220741	-0.022367813	-0.168588554
Q148M8	ABT1	bta:539270; Q148M8	ENSBTAG00000010784	-0.08721492	-0.190568578	-0.103353658
Q3T131	COQ3	bta:540298; Q3T131		-0.519617262	-0.194904041	0.324713221

Accession	Gene_Symbol	Gene_ID	Ensembl_Gene ID	log2(ratio(CM12H/CM0H))	log2(ratio(CM12R/CM0H))	log2(ratio(CM12H/CM12R))
A0A3Q1N4U1	KLHL13	A0A3Q1N4U1	ENSBTAG00000000501	0.149599102	-0.131959588	-0.28155869
A0AAA9T6V6	CYB5A	A0AAA9T6V6	ENSBTAG00000012012	-0.006853671	0.396543913	0.403397584
E1BJ93	LEF1	bta:535399; E1BJ93	ENSBTAG00000006844	0.022056921	0.296652768	0.274595848
A0A3Q1M3B6	RBMX2	A0A3Q1M3B6; bta:613705	ENSBTAG00000014771	-0.010746381	-0.137503524	-0.126757142
A0AAA9RRT9	NIT1	A0AAA9RRT9	ENSBTAG00000020153	0	0.193082722	0.193082722
A0A3Q1M2H1	MMRN1	A0A3Q1M2H1	ENSBTAG00000010285	-0.030290465	-0.006530998	0.023759467
A0A3Q1N888	KATNIP	A0A3Q1N888	ENSBTAG00000006129	0.586423458	0.696047949	0.109624491
Q5E9A7	KLHDC2	bta:535436; Q5E9A7	ENSBTAG00000004933	-0.780950712	-0.416596664	0.364354048
E1B883	RRN3	E1B883	ENSBTAG00000004804	-0.163344304	-0.37401424	-0.210669936
Q2KIJ5	KDSR	bta:505558; F1MLE5; Q2KIJ5	ENSBTAG00000007723	0.067488019	0.148379471	0.080891452
Q5E999	DUSP11	bta:508944; Q2KJ27; Q5E999		0.283135612	-0.454838507	-0.737974119
A0AA95Z4A0	FABP4	A0AA95Z4A0		0.19401871	0.167071811	-0.026946899
A0AAA9SYI5	SNX15	A0AAA9SYI5	ENSBTAG00000002243	0.044688818	0.078855421	0.034166603
E1BA17	COL14A1	E1BA17	ENSBTAG00000013369	-0.016544849	0.016357262	0.032902111
A0AAA9TEN1	OTUD6B	A0AAA9TEN1	ENSBTAG00000003481	0.062735755	0.170637268	0.107901512
F6PVT9	STEAP2	F6PVT9	ENSBTAG00000003506	-0.041222663	0.183727729	0.224950392
F1MQI4	TIMELESS	bta:522884; F1MQI4	ENSBTAG00000020459	-0.045339308	0.106812356	0.152151664
A0AAA9T2J3	CYP4F8	A0AAA9T2J3	ENSBTAG00000048257	-0.012881291	0.259467791	0.272349082
Q5E9C3	SSNA1	bta:507840; Q2NL12; Q5E9C3	ENSBTAG00000046415	0.006018762	-0.490986353	-0.497005114
Q0VCV8	BTBD10	bta:505888; Q0VCV8	ENSBTAG00000013033	-0.039774459	-0.246425488	-0.206651029
P63171	DYNLT1	bta:282380; P63171; Q15763; Q32PD4	ENSBTAG00000004472	-0.075909666	0.179719085	0.255628751
F1MSV8	MRPL21	F1MSV8	ENSBTAG00000004348	0.007876426	-0.236945177	-0.244821603
A0AAA9TGR5	CENPK	A0AAA9TGR5	ENSBTAG00000044175	-0.085143095	-0.214028948	-0.128885854
A7MBI3	L2HGDH	A7MBI3; bta:514230	ENSBTAG00000005550	-0.124545098	-0.111302521	0.013242577
A0AAA9T184	LMBRD2	A0AAA9T184	ENSBTAG00000020142	-0.077184054	0.081643238	0.158827293
F1N6W1	PTPN9	F1N6W1	ENSBTAG00000016984	-0.222787951	-0.285031508	-0.062243557
A0A3Q1NNI1	COA4	A0A3Q1NNI1	ENSBTAG00000054199	0.24906263	0.038322731	-0.210739898
A0AAA9SK75	FAM107B	A0AAA9SK75	ENSBTAG00000010023	0.271302022	0.189142917	-0.082159105
F6RWY3	ALG2	F6RWY3	ENSBTAG00000002454	-0.014006858	-0.046748259	-0.032741401
Q0VC80	YRDC	bta:512062; Q0VC80	ENSBTAG00000006510	-0.074861437	-0.096215315	-0.021353878
A0A3Q1M2Y0	EVI5L	A0A3Q1M2Y0	ENSBTAG00000004294	-0.025445017	0.131683109	0.157128126
Q0VCV5	LNX2	bta:526424; Q0VCV5	ENSBTAG00000015614	0.297522782	-0.157183333	-0.454706115
A0AAA9T2M2	FOXJ2	A0AAA9T2M2	ENSBTAG00000019742	-0.140719301	0.184592154	0.325311455
A0AAA9TSC6	GPATCH11	A0AAA9TSC6	ENSBTAG00000003861	0.16457687	0.049915887	-0.114660983
E1BBK3	FLYWCH2	A0A8J8XTP4; E1BBK3; M5FI44	ENSBTAG00000038115	-0.107054453	-0.278686354	-0.1716319
A0A3Q1N1U9	IFT43	A0A3Q1N1U9	ENSBTAG00000012005	-0.258918297	-0.219389933	0.039528364
E1BDF2	SHARPIN	bta:512499; E1BDF2		-0.1944541	0.265142069	0.459596169
A0A3Q1LPS7	C7H1orf35	A0A3Q1LPS7	ENSBTAG00000020720	0.23786383	-0.080861833	-0.318725663
A0AAF7A363	ALKBH3	A0AAF7A363	ENSBTAG00000009518	-0.056472769	0.038255519	0.094728288
E1BM39	CUL9	bta:540077; E1BM39	ENSBTAG00000019908	0.037512184	-0.087371488	-0.124883672
F1MEK1	METTL17	F1MEK1	ENSBTAG00000006613	-0.260314904	-0.283595728	-0.023280824
Q2HJ90	LRRC42	bta:534728; Q2HJ90	ENSBTAG00000019198	-0.135726649	-0.14412257	-0.008395921
Q0VCH6	SLC25A40	bta:613948; Q0VCH6	ENSBTAG00000054987	0.048461489	-0.078251446	-0.126712935
F1MGY9	AGA	F1MGY9	ENSBTAG00000017085	-0.281876446	-0.07848074	0.203395706
A0A3Q1M6H8	NCAPH2	A0A3Q1M6H8	ENSBTAG00000012607	-0.334060357	-0.102569734	0.231490624
A0AAA9S6S8	TMOD2	A0AAA9S6S8	ENSBTAG00000014011	-0.118082667	-0.182270079	-0.064187412
Q66WT7	CAV2	A4D7R4; A7YWJ5; bta:493642; Q66WT7	ENSBTAG00000062162	0.204323101	0.508557235	0.304234134
A0AAF6ZG78	TMBIM6	A0AAF6ZG78	ENSBTAG00000018588	0.452153841	-0.013370763	-0.465524604
P01035	CST3	bta:281102; P01035; Q54A26	ENSBTAG00000000598	-0.323221412	-0.286493379	0.036728033
F1MWY9	ECI2	bta:505355; F1MWY9	ENSBTAG00000015178	0.150791508	-0.266975784	-0.417767292
A4IFB2	KLF5	A4IFB2; bta:535702	ENSBTAG00000002129	-0.13259361	-0.024045474	0.108548136
A5PJR6	TMEM41B	A5PJR6; bta:616372; F1N5X2	ENSBTAG00000001607	-0.016378928	-0.079511292	-0.063132364
Q3SZC2	PSMB9	bta:510593; Q3SZC2	ENSBTAG00000008954	-0.001220038	0.030170381	0.031390419
Q5E9H3	FES	bta:507304; Q5E9H3	ENSBTAG00000002941	-0.078406345	-0.034322316	0.044084029
A0AAA9SB48	NCKIPSD	A0AAA9SB48	ENSBTAG00000021850	-0.142019005	-0.027020214	0.114998791
F6RC73	LYPLAL1	F6RC73	ENSBTAG00000015534	-0.168858314	-0.081960091	0.086898222
Q3MHX9	NUDT16L1	A0A8J8Y2A7; bta:518085; F1MEX5; M5FK06; Q3MHX9		-0.397678377	-0.330258136	0.067420241
A2VE45	IFT70A	A2VE45; bta:510160		0.30964694	0.11585853	-0.19378841
E1BFW9	OTUD5	E1BFW9	ENSBTAG00000004600	0.048066659	-0.20619161	-0.254258268
A0AAA9SVP3	PRKX	A0AAA9SVP3	ENSBTAG00000014367	-0.450914536	-0.05100578	0.399908756
A0AAA9RYC9	MAP2K7	A0AAA9RYC9	ENSBTAG00000010639	0.293046975	0.262221849	-0.030825126
A1A4M6	STARD5	A1A4M6; bta:512369	ENSBTAG00000010964	-0.281938364	-0.03081482	0.251123543
E1BL80	THAP12	E1BL80	ENSBTAG00000031609	-0.058200633	0.013013328	0.071213961
A0A3Q1LRW7	JADE2	A0A3Q1LRW7	ENSBTAG00000021687	0.026658564	-0.080353397	-0.107011961
Q29RJ1	NUDT2	bta:768044; Q29RJ1		-0.16620802	-0.207804885	-0.041596866
Q08D88	NFIL3	bta:506097; Q08D88	ENSBTAG00000065834	-0.043762826	0.041165744	0.08492857
Q2YDK4	HMGN4	bta:614918; Q2YDK4		0.106128351	0.145064013	0.038935663
Q17QE0	FAM241A	bta:767842; Q17QE0		0.270363684	-0.193449331	-0.463813014
A0AAA9TFG3	SOD3	A0AAA9TFG3	ENSBTAG00000013980	0.029577927	-0.030197045	-0.059774973
A1L539	GPC6	A1L539; bta:536153	ENSBTAG00000040347	0.00996111	0.1857567	0.17579559
Q08DL6	PEX7	bta:533077; Q08DL6	ENSBTAG00000016791	-0.026323587	-0.158931082	-0.132607495
A0AAA9RZ65	SLC31A1	A0AAA9RZ65	ENSBTAG00000021678	-0.03357115	0.099219605	0.132790755
F1MEL4	COIL	F1MEL4	ENSBTAG00000017275	-0.231661917	0.001288696	0.232950613
F1MIU1	LOC510536	F1MIU1	ENSBTAG00000023846	0.20471791	-0.192397681	-0.397115591
A0AAA9TF18	CLMP	A0AAA9TF18	ENSBTAG00000020046	-0.215772972	-1.041779803	-0.826006831
A0AAA9TLH2	SZT2	A0AAA9TLH2; bta:533805	ENSBTAG00000005580	-0.18267085	-0.007166905	0.175503945
Q08DZ3	ELMOD2	bta:515278; Q08DZ3	ENSBTAG00000014007	-0.202469964	-0.170133982	0.032335982
G3MZR4	LOC523702	G3MZR4	ENSBTAG00000045999	-0.34468856	-0.151437774	0.193250786
A0A3Q1MBA7	PHC3	A0A3Q1MBA7	ENSBTAG00000013938	0.199357034	0.077136714	-0.12222032
A0AAA9TZF9	PIP5K1C	A0AAA9TZF9	ENSBTAG00000000710	0.353258582	-0.01750865	-0.370767232
A0A3Q1LMJ2	MFSD10	A0A3Q1LMJ2	ENSBTAG00000021130	-0.461379515	-0.596128927	-0.134749412
A0A3Q1N4A9	CFAP410	A0A3Q1N4A9	ENSBTAG00000019589	-0.139450043	-0.041105203	0.09834484
F1MM05	MAU2	F1MM05	ENSBTAG00000006124	-0.16018307	0.179821038	0.340004108
A0AAA9RX20	BBS1	A0AAA9RX20	ENSBTAG00000020147	-0.160577829	0.245292823	0.405870653
A0AAA9RWF0	TPCN2	A0AAA9RWF0	ENSBTAG00000015183	-0.398790267	-0.221509499	0.177280768
A0A3Q1NEK5	SKA1	A0A3Q1NEK5	ENSBTAG00000018216	-0.067050527	0.057663063	0.12471359
F1N0F2	SIAE	F1N0F2	ENSBTAG00000009412	0.204866369	0.112672671	-0.092193698
A6QP68	PUSL1	A6QP68		-0.211745177	-0.249359469	-0.037614292

Accession	Gene_Symbol	Gene_ID	Ensembl_Gene ID	log2(ratio(CM12H/CM0H))	log2(ratio(CM12R/CM0H))	log2(ratio(CM12H/CM12R))
A0AAA9SW16	TUSC3	A0AAA9SW16	ENSBTAG00000009191	0.057116971	-0.063815098	-0.120932068
F1MGL9	LIMK2	F1MGL9	ENSBTAG00000010662	-0.251119927	-0.074000581	0.177119345
A0A3Q1LQC9	GCFC2	A0A3Q1LQC9	ENSBTAG00000008048	-0.2784134	-0.078588735	0.199824664
Q0IIE8	DNAJB14	bta:538849; Q0IIE8	ENSBTAG00000004426	0.297332181	-0.053025709	-0.35035789
F1MLY5	MLLT6	F1MLY5	ENSBTAG00000011532	-0.007417472	-0.049675159	-0.042257687
Q32KY6	DCTPP1	bta:614103; Q32KY6		-0.190368391	0.034385621	0.224754011
Q0VCL5	HMG20A	bta:513719; Q0VCL5	ENSBTAG00000020441	0.399026727	0.063648676	-0.33537805
Q0P5J9	RPRD1A	bta:504355; Q0P5J9	ENSBTAG00000005497	0.362142551	-0.206450877	-0.568593428
E1BB33	HJURP	bta:617456; E1BB33	ENSBTAG00000024726	-0.011151322	0.065156286	0.076307608
A0AAA9T0M0	CDKN2A	A0AAA9T0M0	ENSBTAG00000034220	-0.025200554	-0.083564717	-0.058364163
Q3SX29	SFRP2	bta:510821; Q3SX29	ENSBTAG00000018563	-0.031864499	-0.73085062	-0.698986121
E1BII1	CTU1	bta:100297056; E1BII1	ENSBTAG00000023365	0.035133537	-0.063216906	-0.098350443
E1BF06	TNFSF15	bta:514239; E1BF06	ENSBTAG00000018069	0.299108616	0.553307162	0.254198546
A0AAA9TQY3	SPINDOC	A0AAA9TQY3	ENSBTAG00000018022	0.297879798	0.499038385	0.201158587
F1MSM3	NOTCH1	F1MSM3	ENSBTAG00000022799	-0.012811387	-0.19386032	-0.181048933
E1BF91	ATAT1	E1BF91	ENSBTAG00000006941	0.190368391	0.099094415	-0.091273976
A0A3Q1MR63	CEP78	A0A3Q1MR63	ENSBTAG00000020752	-0.091902633	0.050417907	0.14232054
A0AAA9SN40	HOOK2	A0AAA9SN40	ENSBTAG00000008654	0.068404505	0.128525498	0.060120992
A0AAA9TEI3	TRIM2	A0AAA9TEI3	ENSBTAG00000008816	-0.27533236	-0.46038863	-0.18505627
A0AAF6YTR8	WDR74	A0AAF6YTR8	ENSBTAG00000009491	-0.519017189	-0.503750432	0.015266757
Q2HJF5	DPH6	bta:767944; Q2HJF5	ENSBTAG00000001364	-0.477159211	-0.271349674	0.205809537
A0AAA9RX26	LBHD1	A0AAA9RX26	ENSBTAG00000010465	-0.449120111	-0.287343858	0.161776253
A0AAA9SG13	CCDC167	A0AAA9SG13	ENSBTAG00000012069	-0.105550265	-0.227728057	-0.122177793
F1N6T3	LSM7	F1N6T3	ENSBTAG00000004521	0.164801852	0.475644264	0.310842412
E1BPY4	NCOA2	E1BPY4	ENSBTAG00000020312	0.10496956	0.011165191	-0.093804368
A0A3Q1M0N2	RNASEH1	A0A3Q1M0N2	ENSBTAG00000016235	-0.200776155	0.005270125	0.20604628
A0AAA9TS89	ATRIP	A0AAA9TS89	ENSBTAG00000008399	-0.383797987	-0.386930876	-0.003132889
A0A3Q1M0Y3	HERPUD1	A0A3Q1M0Y3	ENSBTAG00000016896	-0.011475319	-0.119079983	-0.107604664
A0AAA9SV16	TOP3A	A0AAA9SV16	ENSBTAG00000017087	-0.192082587	-0.033197393	0.158885194
A0A452DJ80	MYCBP	A0A452DJ80	ENSBTAG00000030337	-0.097122467	-0.269058193	-0.171935727
F1MPF7	PHIP	F1MPF7	ENSBTAG00000024688	-0.187701081	-0.134040947	0.053660133
F1MLD7	EFR3A	F1MLD7	ENSBTAG00000047834	0.310678954	0.485926981	0.175248028
A0AAA9TY81	CACUL1	A0AAA9TY81	ENSBTAG00000022808	-0.033487655	-0.152616224	-0.119128568
Q3SYX6	SUPT4H1	bta:616425; Q3SYX6	ENSBTAG00000063664	0.12718008	-0.102569734	-0.229749814
E1BCP9	GLB1L	bta:532551; E1BCP9	ENSBTAG00000020611	-0.110676231	-0.120294234	-0.009618003
A0A3Q1MRT6	FXYD5	A0A3Q1MRT6; bta:505584	ENSBTAG00000031441	0.017059093	-0.288009269	-0.305068362
Q29S14	TVP23B	bta:513550; Q29S14	ENSBTAG00000060680	0.23932086	0.122101608	-0.117219252
Q0VCN2	WDR83	bta:780845; Q0VCN2	ENSBTAG00000006245	0.033462759	-0.057104451	-0.09056721
A0AAA9S8U8	COMMD8	A0AAA9S8U8; bta:507292	ENSBTAG00000001348	0.062121712	0.116364757	0.054243045
F6Q952	KBTBD2	F6Q952	ENSBTAG00000003115	-0.217300279	-0.160219184	0.057081095
A0A3Q1MUL6	IREB2	A0A3Q1MUL6	ENSBTAG00000002504	-0.142222073	-0.01926586	0.122956213
E1BNR7	VANGL1	E1BNR7	ENSBTAG00000005788	-0.09329192	0.07468481	0.16797673
F1MEL7	RFLNB	F1MEL7	ENSBTAG00000006606	0.459243975	0.187475975	-0.271768
A5D7V4	TRMO	A5D7V4; bta:507596; F1MKS2	ENSBTAG00000019238	0.283440616	-0.409294606	-0.692735222
A0AAA9TQW6	KMT2C	A0AAA9TQW6	ENSBTAG00000024199	-0.161625678	-0.210725533	-0.049099854
F1MX94	PTS	A0A3Q1LXL4; A0A8J8XH99; bta:506860; F1MX94	ENSBTAG00000062028	0.207674278	0.22086171	0.013187432
G5E6N6	ZCCHC9	G5E6N6	ENSBTAG00000005265	0.172077131	-0.084091956	-0.256169087
Q08E13	RNF10	A6H708; bta:520757; Q08E13		-0.015830229	0.008562014	0.024392243
Q2T9Q1	TBC1D20	bta:504543; Q2T9Q1	ENSBTAG00000013330	0.183635381	0.178664851	-0.00497053
P00516	PRKG1	bta:282004; P00516; P21136		0.005482065	-0.052196211	-0.057678276
Q5E9T1	GDPGP1	bta:522909; Q0V8C7; Q58D74; Q5E9T1	ENSBTAG00000005174	-0.179344541	-0.009954564	0.169389976
A0A3Q1LXJ7	TRAF1	A0A3Q1LXJ7	ENSBTAG00000003012	0.108557803	0.275634443	0.167076639
G3MZ71	KRT2	G3MZ71	ENSBTAG00000039261	-0.819603468	-0.543739838	0.27586363
A0AAA9RSX8	ATP11C	A0AAA9RSX8	ENSBTAG00000016484	-0.048378923	-0.058543902	-0.010164979
A0AAF6Z3H7	CTSS	A0AAF6Z3H7	ENSBTAG00000017135	-0.080597008	-0.100469155	-0.019872147
A7E309	MDFIC	A7E309; bta:520530	ENSBTAG00000021543	0.053874241	0.086054647	0.032180406
A0AAA9TQG4	ABCD4	A0AAA9TQG4	ENSBTAG00000014633	-0.216072009	-0.022985537	0.193086472
Q5EA32	TRPV2	bta:507664; Q5EA32	ENSBTAG00000003014	-0.044706804	-0.217731044	-0.17302424
A0A3Q1LVR3	PRPF18	A0A3Q1LVR3	ENSBTAG00000017244	-0.258714956	-0.505026005	-0.246311048
P0DW91	ZFTRAF1	P0DW91; Q3SX19; Q7YR89; Q7YR91; Q7YRA6; Q8MK42	ENSBTAG00000035254	0.053066421	0.170339152	0.117272731
Q32LD4	TFB2M	bta:516874; Q32LD4	ENSBTAG00000000072	-0.087062859	-0.237404992	-0.150342133
A0A3Q1M5Y6	UBE2Q2	A0A3Q1M5Y6	ENSBTAG00000004024	-0.08295037	-0.166964411	-0.084014041
A0JNG0	TMEM168	A0JNG0; bta:541075	ENSBTAG00000021967	-0.005967723	0.068331831	0.074299555
Q5DPW9	CST6	bta:503685; G3N3P6; Q5DPW9	ENSBTAG00000046587	0.020922773	0.081929859	0.061007086
E1BCG5	NDST1	bta:514172; E1BCG5	ENSBTAG00000001692	-0.200621799	-0.114528388	0.086093411
A0AAA9T296	ANKRD46	A0AAA9T296	ENSBTAG00000001254	0.252173413	-0.131313235	-0.383486648
A0AAA9SJA1	SIRT6	A0AAA9SJA1	ENSBTAG00000019909	-0.330926545	-0.187352073	0.143574471
A0AAA9S992	MED18	A0AAA9S992	ENSBTAG00000002678	0.424244263	0.390821262	-0.033423002
E1B9D0	CHPF2	E1B9D0	ENSBTAG00000014371	-0.501839398	-0.210728895	0.291110503
Q08DQ8	SLC26A6	bta:514514; E1BFJ3; Q08DQ8	ENSBTAG00000038381	-0.200941634	-0.206741421	-0.005799787
A0AAA9SD21	CSKMT	A0AAA9SD21	ENSBTAG00000022293	0.025474971	0.276677014	0.251202043
A0A3Q1LGF4	SCEL	A0A3Q1LGF4	ENSBTAG00000032821	0.158139218	0.069262662	-0.088876555
Q3ZC66	CRIPT	bta:617723; Q3ZC66	ENSBTAG00000015723	0.360967651	-0.223484793	-0.584452444
A0AAA9TPD8	MPV17	A0AAA9TPD8	ENSBTAG00000018269	-0.060906725	-0.179720512	-0.118813787
F1MFW9	KRT24	bta:788424; F1MFW9	ENSBTAG00000040279	-0.604576882	-0.861539465	-0.256962583
A0AAA9RUZ5	PROCR	A0AAA9RUZ5	ENSBTAG00000008291	-0.005318697	-0.108856302	-0.103537604
G3X7J5	TOR4A	bta:618444; G3X7J5	ENSBTAG00000023787	-0.055205502	0.034061836	0.089267338
A0AAF6ZED3	NFYB	A0AAF6ZED3	ENSBTAG00000030744	0.129837438	0.347576682	0.217739245
A7YWK3	KRT73	A7YWK3; bta:531981	ENSBTAG00000037638	-0.242770421	-0.162517465	0.080252956
Q0II32	TIMMDC1	bta:507206; F6PYZ8; Q0II32	ENSBTAG00000012581	0.577677229	0.27368165	-0.303995579
A0A3Q1M6Z9	MMADHC	A0A3Q1M6Z9	ENSBTAG00000015962	-0.037198618	-0.045296086	-0.008097468
Q17QW3	RDH14	bta:505949; Q17QW3	ENSBTAG00000023928	0.066222267	0.275169157	0.20894689
P82926	MRPS33	bta:523435; P82926; Q32LL3	ENSBTAG00000005285	-0.05336693	-0.019296253	0.034070676
Q5E9C2	DAD1	bta:614538; Q3T175; Q53ZY7; Q5E9C2		0.188103354	-0.018253319	-0.206356673
A0AAA9SAX7	TIMM22	A0AAA9SAX7	ENSBTAG00000008423	0.064784947	-0.120883637	-0.185668584
A0AAF6YLK6	LMAN2L	A0AAF6YLK6	ENSBTAG00000003975	-0.019325416	0.1094444	0.128769815
F6RMD1	TMEM9B	F6RMD1	ENSBTAG00000008310	0.22056738	-0.008536682	-0.229104062

Accession	Gene_Symbol	Gene_ID	Ensembl_Gene ID	log2(ratio(CM12H/CM0H))	log2(ratio(CM12R/CM0H))	log2(ratio(CM12H/CM12R))
A0AAA9TZ13	MYRF	A0AAA9TZ13	ENSBTAG00000006990	0.019279062	0.120941037	0.101661975
A0AAA9S2K4	ABRACL	A0AAA9S2K4	ENSBTAG00000001446	-0.116752246	0.329723717	0.446475964
A7YWG4	GGH	A7YWG4; bta:525303	ENSBTAG00000007534	0.108546535	0.166705266	0.058158731
Q5EA56	TRAF7	A0A8J8XK14; bta:511692; M5FHP8; Q5EA56	ENSBTAG00000019073	0.038672492	-0.010591411	-0.049263903
A0A3Q1M7N7	GULP1	A0A3Q1M7N7	ENSBTAG00000007141	0.056279034	0.123220193	0.066941159
A0AAA9RWT3	PTTG1	A0AAA9RWT3	ENSBTAG00000012184	0	0.217855641	0.217855641
A0AAA9RWZ2	PROSER2	A0AAA9RWZ2	ENSBTAG00000039571	0.217445885	0.143610004	-0.073835881
A0A3Q1LXQ0				-0.274081336	0.451479495	0.725560831
Q08E12	GINS3	bta:538943; Q08E12	ENSBTAG00000009363	0.043614776	0.223208425	0.17959365
A0A452DJ63	KNOP1	A0A452DJ63	ENSBTAG00000019593	0.388989405	0.008699695	-0.38028971
A0AAA9T8Z6	ATXN7	A0AAA9T8Z6	ENSBTAG00000011287	0.164363528	-0.061861354	-0.226224882
A7MBJ3	EDEM2	A7MBJ3; bta:513253		0.499695489	0.313738697	-0.185956792
A0AAA9TJH2	RAD51AP1	A0AAA9TJH2	ENSBTAG00000040065	0.342524999	-0.318228444	-0.660753443
A5D7S5	SLC25A35	A5D7S5; G1K1W1		0.401136682	0.366298259	-0.034838424
E1BHC5	OSGEPL1	bta:514738; E1BHC5	ENSBTAG00000012557	-0.256908303	0.05450192	0.311410223
A0AAA9S9Y1	POLR1H	A0AAA9S9Y1	ENSBTAG00000031792	0.104995734	-0.076594156	-0.181589889
E1BC22	CIAO2B	bta:614585; E1BC22	ENSBTAG00000013931	-0.411554129	0.10749635	0.519050479
A5PJA1	AK6	A5PJA1; bta:102216273		0.139704059	-0.104639496	-0.244343555
F1N5T0	CUTA	F1N5T0	ENSBTAG00000001635	-0.13946874	0.412391392	0.551860132
A0A3Q1MIG0	SH3BP2	A0A3Q1MIG0	ENSBTAG00000013996	-0.208683288	-0.133593975	0.075089313
A0AAA9SWX9	ZNF333	A0AAA9SWX9; bta:789635	ENSBTAG00000062579	0.296116761	-0.185439006	-0.481555767
Q6J1J1	BIRC5	bta:414925; Q32LI0; Q6J1J1	ENSBTAG00000013573	-0.269717296	-0.260048647	0.009668648
A4D7R9	ST7	A4D7R9; Q9BDI1	ENSBTAG00000010756	0.05932428	-0.255517556	-0.314841836
A4IFN9	SMG5	A4IFN9; bta:513598	ENSBTAG00000009435	-0.196647008	-0.072388906	0.124258103
A0A3Q1MQB3	LIN54	A0A3Q1MQB3	ENSBTAG00000010546	-0.609281131	-0.607682577	0.001598554
E1BN06	RASSF5	E1BN06	ENSBTAG00000010427	0.207119566	-0.016142211	-0.223261777
G3MYU1	BAP1	G3MYU1	ENSBTAG00000045817	0.013358383	-0.014838509	-0.028196892
B0JYL2	NPR3	B0JYL2; E1BJP0	ENSBTAG00000007665	0.08718457	0.255129208	0.167944637
E1BIA1	PPFIA3	E1BIA1	ENSBTAG00000002844	-0.54121974	0	0.54121974
F6QT14	ELOVL1	F6QT14	ENSBTAG00000055871	-0.06515268	-0.041820176	0.023332504
A0A3Q1MUK5	AGAP3	A0A3Q1MUK5	ENSBTAG00000048379	-0.340059979	-0.280980746	0.059079233
A0AAF6YL67	TMEM128	A0AAF6YL67	ENSBTAG00000003652	0.044299008	-0.708307553	-0.752606561
A0AAA9TXN7	PARP8	A0AAA9TXN7	ENSBTAG00000004066	-0.191721162	-0.054385579	0.137335583
A0A3Q1LNW3	ICE2	A0A3Q1LNW3	ENSBTAG00000005328	0.041301343	-0.021102693	-0.062404035
G3XCF6	NFATC1	G3XCF6	ENSBTAG00000000656	0.035567246	0.152003093	0.116435848
A0A452DKP2	LOC100299281	A0A452DKP2	ENSBTAG00000046172	-0.853295095	-0.422106083	0.431189012
A1A4J6	ATP9B	A1A4J6; bta:510301	ENSBTAG00000001224	-0.225837665	-0.149424549	0.076413116
A7MBC3	WEE1	A7MBC3	ENSBTAG00000005278	0.52334931	-0.193343889	-0.716693199
Q8HZT6	RDH10	bta:282852; Q8HZT6	ENSBTAG00000020143	0.016089703	-0.02997208	-0.046061782
Q2M2S2	SMDT1	bta:506149; Q2M2S2		-0.173822424	-0.136581232	0.037241192
Q32PA1	CD59	bta:505574; Q32PA1	ENSBTAG00000002302	-0.243501448	-0.135087623	0.108413825
Q1RMN9	LOC510860	bta:510860; Q1RMN9	ENSBTAG00000039196	-0.652211761	-0.535249526	0.116962236
Q08DP7	PPP2R5B	bta:505915; Q08DP7	ENSBTAG00000007061	-0.183640995	0.07694332	0.260584315
A0AAF6DM01	CCDC32	A0AAF6DM01	ENSBTAG00000025593	-0.305610249	0.449930322	0.755540571
F1MGT3	MTSS2	F1MGT3	ENSBTAG00000009358	0.025557711	-0.056531958	-0.082089669
A0AAA9T2X7	BCL7B	A0AAA9T2X7	ENSBTAG00000020544	-0.10782888	-0.048295557	0.059533323
E1B7S1	PARP12	E1B7S1	ENSBTAG00000016546	0.191481145	-0.204662043	-0.396143188
E1B806	SAP30	bta:781150; E1B806	ENSBTAG00000015578	-0.082077513	0.048866001	0.130943514
A0AAF6YRH9	ARFRP1	A0AAF6YRH9	ENSBTAG00000007497	-0.211115563	-0.038913221	0.172202342
A0AAA9U271	POLL	A0AAA9U271	ENSBTAG00000003576	0.138772784	0.081464101	-0.057308683
A0AAF6YYX7	GET1	A0AAF6YYX7	ENSBTAG00000013629	0.183825818	-0.012022528	-0.195848347
F1N1M2	GATA6	F1N1M2	ENSBTAG00000005734	-0.053305831	-0.082961449	-0.029655618
E1BDS4	ABHD14A	E1BDS4	ENSBTAG00000053854	-0.340107537	-0.195182801	0.144924736
E1BLW3	LZTR1	E1BLW3	ENSBTAG00000011292	-1.122484007	-0.434742909	0.687741098
E1BPN5	SSH2	E1BPN5	ENSBTAG00000011011	0.068125833	-0.186720244	-0.254846077
Q58DB8	MDS018	bta:614957; Q58DB8		0.411398922	0.025216286	-0.386182637
Q3SZA2	FMC1	bta:767923; Q3SZA2		-0.013617569	-0.157367525	-0.143749956
F1MEB4	SPG11	F1MEB4	ENSBTAG00000000362	-0.141463549	-0.020652429	0.12081112
F1N148	MRPL54	F1N148	ENSBTAG00000008396	-0.055699189	-0.200658518	-0.144959329
A0AAA9TRL1	PRXL2A	A0AAA9TRL1	ENSBTAG00000021416	-0.032625147	-0.007186541	0.025438607
A0AAA9SS13	BAZ2A	A0AAA9SS13	ENSBTAG00000017840	-0.196579541	-0.115789794	0.080789747
Q3SWY9	RAB28	bta:536021; Q3SWY9	ENSBTAG00000003218	-0.169925001	0.061400545	0.231325546
A0A3Q1MR14	LGALSL	A0A3Q1MR14	ENSBTAG00000055296	0.112124895	0.226827393	0.114702497
F1N5Q7	RFXAP	F1N5Q7	ENSBTAG00000005351	0.113327684	-0.134868747	-0.24819643
A0AAA9RYL0	ROR1	A0AAA9RYL0	ENSBTAG00000012312	0.205708396	0.081931855	-0.123776541
A0AAA9TP99	MACF1	A0AAA9TP99	ENSBTAG00000021024	-1.010527175	-1.035719266	-0.025192091
A6QLM0	PEDS1	A6QLM0; bta:507694	ENSBTAG00000010135	0.066042277	0.050951187	-0.01509109
Q05716	IGFBP4	A5D7U8; bta:282262; Q05716	ENSBTAG00000008611	0.413820549	-0.42235465	-0.836175198
Q1RMJ7	TRNAU1AP	bta:532582; Q1RMJ7	ENSBTAG00000010393	0.245112498	0.157488633	-0.087623865
A0A3Q1NG02	MCRS1	A0A3Q1NG02	ENSBTAG00000019281	0.012443063	-0.264423616	-0.276866679
A0AAA9U068	PEX3	A0AAA9U068	ENSBTAG00000001747	-0.231099117	-0.258873783	-0.027774666
A6QQQ3	PRELP	A6QQQ3; F1MX63	ENSBTAG00000017834	0.274783005	-0.093310211	-0.368093216
Q32LM8	NRM	bta:511167; Q32LM8	ENSBTAG00000006966	-0.044147104	-0.173387669	-0.129240565
E1BGW5	SIN3B	E1BGW5	ENSBTAG00000009330	-0.053826991	0.06317286	0.116999851
F1MER1	CDYL2	F1MER1	ENSBTAG00000017112	0.018615678	-0.220571986	-0.239187664
A0AAA9SA86	TAF8	A0AAA9SA86	ENSBTAG00000011339	-0.013533824	-0.151853429	-0.138319605
Q2YDE7	ZBTB8OS	bta:613795; Q2YDE7	ENSBTAG00000027159	-0.278610212	0.050737362	0.329347574
A0A3Q1M5I0	SCAI	A0A3Q1M5I0	ENSBTAG00000015553	-0.051208941	-0.168565892	-0.117356951
A0AAA9TWI8	CENPH	A0AAA9TWI8	ENSBTAG00000014246	-0.522901533	-0.586548752	-0.063647219
F1N3D2	ZNF280C	F1N3D2	ENSBTAG00000019885	-0.131108903	0.005685505	0.136794408
A0AAA9T0W6	NECTIN3	A0AAA9T0W6	ENSBTAG00000013769	0.017505109	-0.117680366	-0.135185475
A0AAA9RVN1	APLF	A0AAA9RVN1	ENSBTAG00000018401	0.021635914	0.175199943	0.15356403
A0AAA9TTS0	PINX1	A0AAA9TTS0	ENSBTAG00000000500	0.152295107	-0.180182907	-0.332478014
A0AAF6Z712	GINS1	A0AAF6Z712	ENSBTAG00000019984	0.011809302	-0.006602735	-0.018412037
Q3MHP8	BET1L	bta:509758; Q3MHP8	ENSBTAG00000001744	0.035650587	0.095227047	0.05957646
E1BGA4	ASH1L	E1BGA4	ENSBTAG00000003954	-0.357117523	-0.623718544	-0.266601021
Q58CQ1	IRF9	bta:509855; Q58CQ1	ENSBTAG00000005816	-0.004204068	0.396376388	0.400580456
A0A3Q1MS71	LDB1	A0A3Q1MS71	ENSBTAG00000005780	0.12853337	-0.067114196	-0.195647566
A7YWM8	NANP	A7YWM8; bta:516539	ENSBTAG00000000686	0.10744629	-0.125801328	-0.233247618
A0AAA9TMB1	MANEA	A0AAA9TMB1	ENSBTAG00000005026	-0.971493572	-0.798319779	0.173173793

Accession	Gene_Symbol	Gene_ID	Ensembl_Gene ID	log2(ratio(CM12H/CM0H))	log2(ratio(CM12R/CM0H))	log2(ratio(CM12H/CM12R))
E9LZ03				0.303804012	0.244538062	-0.059265949
A0A3Q1N9J7	CLDND1	A0A3Q1N9J7	ENSBTAG00000020996	0.048005373	0.029525952	-0.018479421
A0AAA9RUN2	PHACTR2	A0AAA9RUN2	ENSBTAG00000016498	0.09654394	0.128584206	0.032040266
A0AAA9ST48	KDM3A	A0AAA9ST48	ENSBTAG00000013580	-0.078855421	-0.231158732	-0.152303311
A0AAA9RU64	UBE2B	A0AAA9RU64	ENSBTAG00000010982	0.030457291	-0.170259617	-0.200716908
A0AAA9SV46	INO80C	A0AAA9SV46	ENSBTAG00000012426	-0.456443762	0.064680996	0.521124758
A5PK28	LHFPL2	A5PK28; bta:616131	ENSBTAG00000007374	0.529240026	0.277865211	-0.251374815
F1MEA1	TMEM132A	F1MEA1	ENSBTAG00000018364	-0.043562035	-0.001480447	0.042081589
A0AAA9TR91	TMEM222	A0AAA9TR91	ENSBTAG00000010249	-0.119482852	-0.068257528	0.051225323
E1B7K6	DGKE	bta:538147; E1B7K6	ENSBTAG00000004449	0.159154917	-0.146162215	-0.305317132
A0A3Q1MXI5	DAPK1	A0A3Q1MXI5	ENSBTAG00000000738	-0.048344392	-0.116023796	-0.067679405
E1BE31	JMY	E1BE31	ENSBTAG00000025856	0.199840579	0.02181811	-0.178022469
A6QPS5	CENPI	A6QPS5; bta:512877; F1MZ56	ENSBTAG00000021254	-0.061597191	-0.096631242	-0.035034051
A0AAF6YZ47	CENPS	A0AAF6YZ47	ENSBTAG00000013531	0.394434965	-0.143257051	-0.537692016
A5PJW2	TMEM223	A5PJW2; bta:616632		-0.035310011	-0.113153662	-0.077843651
A0A3Q1MFB7	ZNF706	A0A3Q1MFB7	ENSBTAG00000008054	0.487013078	-0.241296696	-0.728309774
A2VDQ0	IFT81	A2VDQ0; bta:519602	ENSBTAG00000020584	-0.295696553	-0.169759564	0.125936989
A0A3Q1LTR5	NR2C2	A0A3Q1LTR5	ENSBTAG00000013716	-0.167766893	-0.034976138	0.132790755
A5PJJ2	ALG12	A5PJJ2; bta:100125314; G3MWZ3	ENSBTAG00000046173	-0.435215381	-0.404149183	0.031066198
Q3T0H0	LCMT1	bta:618021; Q3T0H0	ENSBTAG00000013169	-0.05246742	0.082856932	0.135324352
Q3SZN3	OMA1	bta:506223; F1N0Z4; Q3SZN3	ENSBTAG00000017326	0.292574877	0.302197461	0.009622585
F6R642	MRM1	F6R642	ENSBTAG00000010311	-0.641712463	-0.627180438	0.014532026
A0A3Q1M937	KCTD9	A0A3Q1M937	ENSBTAG00000012382	-0.326288213	-0.190159485	0.136128728
F1MBU9	PIK3C2B	F1MBU9	ENSBTAG00000024633	0.083370087	0.053488797	-0.02988129
Q08DQ3	CDK2AP1	bta:615387; Q08DQ3	ENSBTAG00000020504	-0.336790856	-0.436849719	-0.100058863
A0A3Q1LF69	CLSPN	A0A3Q1LF69	ENSBTAG00000002826	0.415688388	-0.363872739	-0.779561127
Q5E9L5	PRMT6	bta:540228; Q5E9L5	ENSBTAG00000039951	0.100111485	0.046150746	-0.053960739
E1BHM1	MRGBP	bta:616297; E1BHM1	ENSBTAG00000031716	-0.178217197	-0.342025664	-0.163808468
E1B7T3	NUDT15	E1B7T3	ENSBTAG00000019501	0.381831982	0.376720568	-0.005111414
F1MK42	RHOQ	bta:100139988; F1MK42	ENSBTAG00000013749	0.144778357	0.128700149	-0.016078208
E1BK04	NCOA1	E1BK04	ENSBTAG00000015007	0.205134249	0.024466867	-0.180667382
Q3T0L3	MRPL17	bta:506988; Q3T0L3	ENSBTAG00000024781	-1.303246674	-1.415493399	-0.112246725
A0AAA9TY18	IFT80	A0AAA9TY18	ENSBTAG00000031572	0.27702487	-0.11972526	-0.396750131
A0A3Q1LVT3	TRIM4	A0A3Q1LVT3	ENSBTAG00000050135	0.172202342	-0.053987647	-0.226189989
F1MFA9	MCPH1	F1MFA9	ENSBTAG00000011032	-0.050747504	-0.130381322	-0.079633819
Q2TBV5	GTF2H2	bta:511829; Q2TBV5		0	0.156827013	0.156827013
A3KN46	LETMD1	A3KN46; bta:514595		-0.041945968	0.101814813	0.143760781
A0AAA9TAP6	CDA	A0AAA9TAP6; bta:616377	ENSBTAG00000068005	-0.325799619	-0.182267063	0.143532556
A0AAA9U0Q6	SHB	A0AAA9U0Q6	ENSBTAG00000014922	0.074325111	0.035267635	-0.039057476
Q3ZBP7	TAF11	bta:507047; Q3ZBP7	ENSBTAG00000016887	0.277498229	-0.197350746	-0.474848975
F1MYW5	MPND	F1MYW5	ENSBTAG00000006005	0.051138849	-0.056881022	-0.108019871
Q05B92	TFE3	bta:520800; Q05B92		-0.008137049	0.046310735	0.054447784
A0A3Q1N625	GPR89A	A0A3Q1N625	ENSBTAG00000005809	-0.065357201	-0.1291499	-0.063792699
F1MFT2	R3HCC1	bta:614496; F1MFT2	ENSBTAG00000025830	0.239187664	0.065279149	-0.173908515
A0AAA9TZE6	TMEM126B	A0AAA9TZE6; bta:504229	ENSBTAG00000020596	0.198313828	0.024139354	-0.174174474
Q5EA28	CXXC1	bta:511446; Q5EA28	ENSBTAG00000021884	-0.048543852	-0.002654453	0.045889399
F1N1C8	BRAT1	F1N1C8	ENSBTAG00000014502	-0.085699552	-0.046810539	0.038889013
F1MV64	INSR	bta:408017; F1MV64	ENSBTAG00000012687	0.016893578	-0.082162317	-0.099055895
Q2HJ67	HOXA5	bta:768039; Q2HJ67		0.259749104	-0.228371274	-0.488120377
G3MZ88	DNAJB9	bta:614588; G3MZ88	ENSBTAG00000046588	0.196422218	-0.167520223	-0.363942441
A0AAA9TXA5	RCSD1	A0AAA9TXA5	ENSBTAG00000014151	0.259690963	0.150198597	-0.109492366
A0A3Q1M501	PLEKHA6	A0A3Q1M501	ENSBTAG00000004641	0.183352006	0.190278063	0.006926058
F1N4Z9	EEIG2	bta:514601; F1N4Z9	ENSBTAG00000005439	0.05367598	-0.089987813	-0.143663793
A0AAF6YW66	MED22	A0AAF6YW66	ENSBTAG00000011556	-0.254109783	-0.437787939	-0.183678157
A0JNI5	CLASRP	A0JNI5; bta:509208		-0.054076673	-0.198862112	-0.144785439
E1BDI0	LARGE1	bta:506466; E1BDI0	ENSBTAG00000021953	0.245312636	0.025394628	-0.219918008
A0AAA9S3U5	PPP1R37	A0AAA9S3U5	ENSBTAG00000018834	-0.143460093	-0.02103799	0.122422103
Q32L52	GID8	bta:505570; Q32L52	ENSBTAG00000001827	-0.343839046	-0.718422552	-0.374583506
A0AAA9TSQ0	MRPS14	A0AAA9TSQ0	ENSBTAG00000001109	0.154722595	0.045131971	-0.109590624
Q5EA10	PIGM	bta:509680; Q5EA10		-0.034966046	0.107737487	0.142703533
Q5EAC3	RAD9A	F1N0C5; Q5EAC3		-0.22059914	0.147535826	0.368134966
A0AAA9T5P5	UBE2E1	A0AAA9T5P5	ENSBTAG00000055252	-0.140233317	-0.699871661	-0.559638344
A4FV21	ALG3	A4FV21; bta:614624		0.094734007	0.142640393	0.047906385
A0A3Q1LSC0	FGD4	A0A3Q1LSC0	ENSBTAG00000005453	0.181819712	0.265389827	0.083570115
A0AAF6Z380	FUNDC2	A0AAF6Z380	ENSBTAG00000016977	0.169732552	-0.100401897	-0.270134449
F6RZG1	MDK	F6RZG1	ENSBTAG00000007740	0.043647885	0.00491549	-0.038732394
F6RA94	DAGLB	F6RA94	ENSBTAG00000009236	-0.010134377	-0.002888279	0.007246098
F1MZC5				-0.067479278	-0.083635046	-0.016155768
E1BPH4	HPS3	bta:783104; E1BPH4	ENSBTAG00000002395	-0.207472496	-0.221163655	-0.013691159
A0A3Q1LUE0	FGFR1	A0A3Q1LUE0	ENSBTAG00000015457	-0.331941884	-0.83121592	-0.499274036
A0AAA9S476				0.073291117	0.159643156	0.086352038
A0A3Q1LW44	PIAS2	A0A3Q1LW44	ENSBTAG00000007833	0.018838505	0.049699469	0.030860964
A0A3Q1LM42	ANKS6	A0A3Q1LM42	ENSBTAG00000005133	-0.050022607	-0.064409713	-0.014387106
F1MND2	GBA2	F1MND2	ENSBTAG00000011431	-0.062569823	-0.039362432	0.023207391
G1K1B6	POP5	G1K1B6	ENSBTAG00000005385	-0.1686594	-0.221669085	-0.053009685
Q8WNW7	NFKBIA	bta:282291; Q8WNW7	ENSBTAG00000016683	0.367137798	-0.006348503	-0.373486301
E1BJL2	PCGF6	bta:507739; E1BJL2	ENSBTAG00000009879	0.137503524	-0.027307346	-0.16481087
A0AAA9TR18	F3	A0AAA9TR18	ENSBTAG00000007101	-0.155425432	-0.078182433	0.077242999
A5H027	RNASEL	A5H027; bta:100048947; E1BC88	ENSBTAG00000009091	0.222392421	0.401616984	0.179224562
Q08DR4	UGCG	bta:514357; Q08DR4	ENSBTAG00000016073	-0.493301048	-0.552608142	-0.059307094
Q2YDC9	PDCD2	bta:512079; Q2YDC9	ENSBTAG00000011179	-0.557310267	-0.269020161	0.288290106
A0A3Q1LT05	AMMECR1L	A0A3Q1LT05; bta:539958	ENSBTAG00000008089	-0.214965966	-0.190769255	0.02419671
Q08DU8	BLOC1S6	bta:614408; F1MY65; Q08DU8	ENSBTAG00000012250	0.323294931	-0.001368132	-0.324663063
A6QP39	MSLN	A0A8J8Y870; A6QP39; bta:516237; M5FJX0		-0.672386644	-1.016189238	-0.343802595
A0AAA9S1B7	CTNNAL1	A0AAA9S1B7	ENSBTAG00000004997	0.28366202	-0.154653421	-0.438315441
A0AAA9RZ54	CMTM7	A0AAA9RZ54	ENSBTAG00000001712	-0.047474018	-0.099265227	-0.051791209
Q58DU8	NR2C2AP	bta:508684; Q58DU8	ENSBTAG00000001763	-0.051147205	-0.017942171	0.033205034
E1BE33	ZEB2	E1BE33	ENSBTAG00000012615	-0.068786111	-0.205448658	-0.136662547
Q2NKY9	JAGN1	bta:539101; Q2NKY9	ENSBTAG00000010832	0.113587017	0.256625266	0.143038249
A0A3Q1MPI7	DAAM1	A0A3Q1MPI7	ENSBTAG00000005984	0.161328251	-0.056911824	-0.218240075

Accession	Gene_Symbol	Gene_ID	Ensembl_Gene ID	log2(ratio(CM12H/CM0H))	log2(ratio(CM12R/CM0H))	log2(ratio(CM12H/CM12R))
Q08DG5	HOXB4	bta:768240; Q08DG5	ENSBTAG00000039599	-0.058506336	-0.177888737	-0.119382401
A0AAA9SBV3	VAMP5	A0AAA9SBV3	ENSBTAG00000018285	0.142417045	0.135185475	-0.007231569
A0AAF6DMA2	PDSS1	A0AAF6DMA2; bta:509696		0.034866246	0.187695862	0.152829616
F1N4X3	TSC22D1	F1N4X3	ENSBTAG00000046664	0.287281952	0.150779952	-0.136502
E1BQ28	SLC39A8	bta:508193; E1BQ28	ENSBTAG00000005668	-0.427882783	-0.63673615	-0.208853368
E1BJM5	PASK	E1BJM5	ENSBTAG00000000785	-0.104195087	-0.172759374	-0.068564286
A0AAA9SYD9	ROBO1	A0AAA9SYD9; bta:536815	ENSBTAG00000009851	0.028401937	0.011581772	-0.016820165
A0A3Q1LY39	CCSER2	A0A3Q1LY39; bta:540046	ENSBTAG00000017611	-0.071242081	-0.331798339	-0.260556257
A0AAF6Z4P3	TGFBR1	A0AAF6Z4P3	ENSBTAG00000018035	-0.192645078	-0.041157325	0.151487753
A0A3Q1NL90	NIN	A0A3Q1NL90; bta:510366	ENSBTAG00000020281	-0.033390768	-0.192448619	-0.159057851
A0AAA9S473	ZNF280D	A0AAA9S473	ENSBTAG00000056475	-0.172984801	-0.049753035	0.123231766
A0AAF6ZZH8	AMOTL2	A0AAF6ZZH8	ENSBTAG00000000742	-0.10600398	-0.26066642	-0.15466244
A0AAA9T8A6	CRPPA	A0AAA9T8A6	ENSBTAG00000015073	-0.147300275	-0.217262834	-0.069962558
E1BEH7	TC2N	E1BEH7	ENSBTAG00000003375	-0.097135389	-0.086013916	0.011121473
A0AAA9U255	LDAF1	A0AAA9U255	ENSBTAG00000018562	0.433230616	0.396148359	-0.037082257
F1N0S8	TUSC1	F1N0S8	ENSBTAG00000008551	-0.066810918	0.143859019	0.210669936
A0AAA9SRY7	DCP2	A0AAA9SRY7	ENSBTAG00000000767	-0.079511292	-0.150044036	-0.070532744
A2VE68	ATG4C	A2VE68; bta:531455		0.027539024	0.059017255	0.031478231
G3N0S0	UIMC1	G3N0S0	ENSBTAG00000001707	-0.017903374	0.053843583	0.071746957
A0A3Q1ME40	N4BP1	A0A3Q1ME40	ENSBTAG00000019938	-0.165228215	-0.110028047	0.055200167
A0A3Q1M2Y5	TTYH3	A0A3Q1M2Y5	ENSBTAG00000005934	-0.036419479	-0.115131455	-0.078711976
Q17QH5	BID	bta:510373; Q17QH5		-0.345097259	0.591548013	0.936645272
F6RDW7	NUDT7	F6RDW7	ENSBTAG00000007184	-0.009993969	0.238159737	0.248153706
F1MSN2	TACSTD2	bta:539853; F1MSN2	ENSBTAG00000004381	0.274174963	0.188445089	-0.085729874
P05630	ATP5F1D	A6H7D8; bta:338081; P05630	ENSBTAG00000000550	-0.082205795	0.182621893	0.264827688
A0AAA9THD8	PBX2	A0AAA9THD8	ENSBTAG00000014421	-0.006004983	0.01934535	0.025350333
A0A3Q1LNE2	ARHGEF11	A0A3Q1LNE2	ENSBTAG00000012361	0.012504477	-0.056018492	-0.068522969
A0AAA9S123	POLK	A0AAA9S123	ENSBTAG00000004028	-0.131371761	-0.255041615	-0.123669854
A0AAA9SX79	ARHGAP11A	A0AAA9SX79	ENSBTAG00000020100	0.185629902	0.038247469	-0.147382433
A0AAA9SB29	NHEJ1	A0AAA9SB29	ENSBTAG00000031041	-0.231998109	-0.245030673	-0.013032564
F1N0Q2	AKAP6	bta:537810; F1N0Q2	ENSBTAG00000017719	-0.117598391	-0.36111736	-0.243518969
E1BAD1	C2H2orf69	bta:535735; E1BAD1	ENSBTAG00000009952	-0.112373979	-0.238637752	-0.126263773
A0AAA9SG42	HDAC5	A0AAA9SG42	ENSBTAG00000016254	0.10375394	-0.004427712	-0.108181652
A0A3Q1MIR9	KAT5	A0A3Q1MIR9	ENSBTAG00000005578	-0.145794129	-0.159131928	-0.0133378
Q2KID7	OSTC	bta:768033; Q2KID7	ENSBTAG00000011611	-0.184929584	-0.199205957	-0.014276373
A0A140T8A3	MTG1	A0A140T8A3	ENSBTAG00000020304	-0.612262816	-0.424265464	0.187997352
A0A3Q1MH43	USP32	A0A3Q1MH43	ENSBTAG00000020089	-0.099988213	-0.046672398	0.053315815
F1MDF1	PTPN18	F1MDF1	ENSBTAG00000011658	0.243719263	0.280872261	0.037152998
A0A452DIZ1	SUDS3	A0A452DIZ1	ENSBTAG00000019757	-0.094063711	-0.04895446	0.045109252
A0A3Q1NID4	METAP1D	A0A3Q1NID4	ENSBTAG00000019075	-0.195592362	-0.172180975	0.023411387
E1BE60	PPP1R14A	bta:615348; E1BE60	ENSBTAG00000023472	0.045716596	-0.282299305	-0.3280159
A0AAA9T1N8	DMD	A0AAA9T1N8	ENSBTAG00000046838	0.034454056	0.02448967	-0.009964386
A0A3Q1LXN7	HCFC2	A0A3Q1LXN7	ENSBTAG00000020933	-0.126474745	-0.026183932	0.100290814
A0AAA9T877	PARS2	A0AAA9T877; bta:508907	ENSBTAG00000055903	-0.222176594	-0.060837586	0.161339009
A0AAA9SVU5	SLC25A21	A0AAA9SVU5	ENSBTAG00000019350	-0.063066795	-0.165542934	-0.102476139
A0AAF6ZDS6				-0.069239083	-0.024662054	0.044577028
A0AAA9S0W0	NDUFB3	A0AAA9S0W0	ENSBTAG00000012760	0.019645513	-0.144194726	-0.163840239
A0A3Q1LX72	RGS10	A0A3Q1LX72	ENSBTAG00000002647	0.140588996	0.075110925	-0.065478071
A0AAA9S566	ARHGEF28	A0AAA9S566	ENSBTAG00000005633	0.26634714	0.143821248	-0.122525892
A0A3Q1MSW4	TBC1D1	A0A3Q1MSW4	ENSBTAG00000013699	-0.347336341	-0.227568014	0.119768326
A0A3Q1M4P0	NPR2	A0A3Q1M4P0	ENSBTAG00000011434	-0.204744556	-0.102709921	0.102034634
A1A4H5	CRTC1	A1A4H5; bta:510465		0.257943639	0.226741684	-0.031201955
A0A3Q1LP80	DERPC	A0A3Q1LP80	ENSBTAG00000001698	-0.480576719	-0.34696349	0.133613228
A0A3Q1N1A7	CPB2	A0A3Q1N1A7	ENSBTAG00000007073	-0.311054699	-0.588439474	-0.277384775
A0AAA9S7Q8	TRAPPC2L	A0AAA9S7Q8	ENSBTAG00000019184	0.036749186	0.188171601	0.151422415
Q24JZ5	TMEM199	bta:511376; Q24JZ5		0.262509885	0.12788887	-0.134621015
E1BE01	SOX6	E1BE01	ENSBTAG00000044185	-0.355012728	-0.559985835	-0.204973107
F1MZ35	POLR2I	F1MZ35	ENSBTAG00000015002	-0.23520197	0.030286695	0.265488665
E1BDK9	RETSAT	bta:614455; E1BDK9	ENSBTAG00000021934	-0.19466168	-0.109185844	0.085475836
A4FV53	GTPBP2	A4FV53; bta:513605	ENSBTAG00000006016	0.022593002	0.214236973	0.191643971
Q5EA03	TMEM186	bta:513552; Q5EA03	ENSBTAG00000022530	-0.400402153	-0.091074095	0.309328058
A0A452DII7	MXRA8	A0A452DII7	ENSBTAG00000012247	-0.646616432	-0.850663318	-0.204046886
E1BJE9	SMCR8	E1BJE9	ENSBTAG00000017090	-0.107313024	-0.19190656	-0.084593536
Q2TBW9	ING5	bta:614733; Q2TBW9	ENSBTAG00000009123	-0.094530655	-0.116104067	-0.021573412
Q2TBQ0	TFB1M	bta:533438; Q2TBQ0	ENSBTAG00000031895	-0.075536103	0.035657776	0.111193879
A0A452DIL0	FAM193B	A0A452DIL0	ENSBTAG00000015192	-0.073275095	-0.530236204	-0.456961109
A0AAA9SGD2	DXO	A0AAA9SGD2	ENSBTAG00000005588	-0.159002234	-0.117223822	0.041778412
A2VE77	COP1	A2VE77; bta:519896	ENSBTAG00000031385	0.441304274	0.202389397	-0.238914877
A0AAA9SUV1	MYBPC1	A0AAA9SUV1	ENSBTAG00000011392	-0.810461889	-0.789981647	0.020480242
A0AAA9SA39	NEPRO	A0AAA9SA39; bta:516064	ENSBTAG00000005723	-0.184700131	-0.173718575	0.010981557
A0AAA9TZD2				-0.119463667	0.014260693	0.13372436
Q2KJ48	CDR2	bta:534647; Q2KJ48	ENSBTAG00000018050	0.027591385	-0.254264196	-0.281855581
F6PSM0	DKK3	F6PSM0	ENSBTAG00000004275	0.169394696	0.096882197	-0.0725125
A0A3Q1M282	WDFY2	A0A3Q1M282	ENSBTAG00000008053	-0.348648094	-0.171491523	0.177156571
A0AAF6Z3W7	PAXIP1	A0AAF6Z3W7	ENSBTAG00000017505	-0.279230753	-0.064192379	0.215038373
A0AAA9TDE5	DTWD1	A0AAA9TDE5	ENSBTAG00000006201	-0.184368379	-0.315144733	-0.130776354
A0AAA9T8R5	FXYD6	A0AAA9T8R5	ENSBTAG00000014354	-0.113568371	0.447369087	0.560937457
A3KN50	MYST1	A3KN50; bta:100125883		-0.977339403	-1.074200942	-0.096861539
G3N1G1	RUSF1	G3N1G1	ENSBTAG00000047734	-0.01603821	-0.201996393	-0.185958183
A0AAA9TCZ9	DPH7	A0AAA9TCZ9	ENSBTAG00000038691	0.072270286	-0.009915469	-0.082185755
F6RGV5	MED20	F6RGV5	ENSBTAG00000010100	0.171342884	0.089714121	-0.081628762
F1MNH5	DESI2	F1MNH5	ENSBTAG00000001478	-0.041496302	-0.061058568	-0.019562266
A7MBC7	NEMP1	A7MBC7; bta:533988	ENSBTAG00000014659	-0.874134502	-0.426798209	0.447336294
A0A3Q1M0J3	CHCHD2	A0A3Q1M0J3	ENSBTAG00000040295	-0.241621099	-0.533639193	-0.292018094
Q17QY0	MYBL2	bta:510420; Q17QY0	ENSBTAG00000007783	0.033469157	-0.539139916	-0.572609073
A0AAF6ZA20	UXT	A0AAF6ZA20	ENSBTAG00000015820	0.071239131	0.112353012	0.041113881
A0AAA9S0S8	FIRRM	A0AAA9S0S8	ENSBTAG00000014793	0.193006068	-0.00869096	-0.201697028
E1B7S6	NR4A3	bta:528877; E1B7S6	ENSBTAG00000001864	-1.75116391	-1.599160817	0.152003093
E1B6Z5	CCNT2	E1B6Z5	ENSBTAG00000014158	0.151007788	0.231904508	0.08089672
Q3MHN2	C9	bta:526766; Q3MHN2	ENSBTAG00000016149	-0.443877182	-0.341788517	0.102088665
A0AAA9SGM8	MKNK1	A0AAA9SGM8	ENSBTAG00000019933	-0.242767007	-0.247860375	-0.005093368

Accession	Gene_Symbol	Gene_ID	Ensembl_Gene ID	log2(ratio(CM12H/CM0H))	log2(ratio(CM12R/CM0H))	log2(ratio(CM12H/CM12R))
A0A452DJ03	VWA1	A0A452DJ03	ENSBTAG00000021294	-0.491730256	0.056533129	0.548263386
A0A3Q1N0H8	FRA10AC1	A0A3Q1N0H8	ENSBTAG00000000447	-0.126478154	-0.095541987	0.030936167
A0AAA9T2G4	PGS1	A0AAA9T2G4	ENSBTAG00000000675	-0.063495178	-0.053790914	0.009704263
A5PJR3	DCXR	A5PJR3		-0.029673918	-0.368793315	-0.339119396
A0AAF6YVJ7				0.082913637	-0.034269902	-0.117183539
A0A3Q1N7G2	PALD1	A0A3Q1N7G2	ENSBTAG00000008583	0.058181071	0.017702002	-0.040479069
Q3MHM0	APBB1IP	bta:533670; Q3MHM0	ENSBTAG00000012526	0.146301397	0.157348889	0.011047493
A0A3Q1M7E0	DHCR24	A0A3Q1M7E0	ENSBTAG00000004688	0.139724764	-0.076644681	-0.216369445
F1N1V2	CEPT1	F1N1V2	ENSBTAG00000020169	0.126742044	0.041404686	-0.085337357
F1MNS5	ENPP1	bta:615535; F1MNS5	ENSBTAG00000021830	-0.216583575	-0.123124384	0.093459191
A0A3Q1M1G0				-0.143745201	-0.123582986	0.020162215
E1BDL0	PCDHB15	E1BDL0	ENSBTAG00000062518	-0.105717721	-0.035836295	0.069881426
A0AAF6ZX18	UBE2E3	A0AAF6ZX18	ENSBTAG00000043956	0.107132821	-0.134003866	-0.241136688
G3X6H5	JADE3	G3X6H5	ENSBTAG00000005124	0.00981429	-0.018168123	-0.027982413
A0AAA9SSM2	MRPL18	A0AAA9SSM2	ENSBTAG00000001289	-0.123853469	-0.231210878	-0.107357409
A2VE81	OCLN	A2VE81; bta:512405	ENSBTAG00000000561	0.681603596	0.686299109	0.004695513
E1BGF9	ZHX3	bta:512451; E1BGF9	ENSBTAG00000017594	-0.007075517	0.078299669	0.085375186
A0A3Q1MUT2	DPY19L3	A0A3Q1MUT2; bta:521976	ENSBTAG00000015165	0.024821172	0.062769047	0.037947875
A0AAA9TS92	GTPBP8	A0AAA9TS92	ENSBTAG00000006534	-0.064254542	-0.271254913	-0.207000371
A0AAA9T8L6	SNX14	A0AAA9T8L6	ENSBTAG00000018408	-0.118877396	-0.135195971	-0.016318575
O02659	MBL	A4IFQ1; bta:281297; O02659	ENSBTAG00000007049	0.132155959	-0.176756205	-0.308912164
Q6B4J2	VKORC1	A2VDX1; bta:445422; Q6B4J2		0.093010964	0.220140673	0.127129709
A6QLK2	NABP2	A6QLK2; bta:533842	ENSBTAG00000002798	-0.144032798	-0.065241195	0.078791603
A0AAA9RXL2	SLC17A5	A0AAA9RXL2	ENSBTAG00000044053	0.095551274	0.37961938	0.284068106
E1BC46	KIF18B	E1BC46	ENSBTAG00000008088	0.077505215	0.042552616	-0.034952599
A0AAA9SVM7	CETN4	A0AAA9SVM7	ENSBTAG00000016147	0.055371264	-0.254918325	-0.310289589
A0A3Q1M4X6	SMUG1	A0A3Q1M4X6	ENSBTAG00000021974	-0.150690956	-0.003943588	0.146747368
A0A3Q1MCW8	MGAT5	A0A3Q1MCW8; bta:537595	ENSBTAG00000018744	-0.034433497	0.236420413	0.27085391
A0A3Q1LTS5	COX16	A0A3Q1LTS5	ENSBTAG00000030595	0.021413307	-0.241621099	-0.263034406
A0A452DIV4	RABAC1	A0A452DIV4	ENSBTAG00000018633	0.082617031	-0.268900731	-0.351517762
A0A3Q1MCG3	NT5E	A0A3Q1MCG3	ENSBTAG00000048655	0.028108485	-0.017726169	-0.045834654
F1MB88	ZBTB14	bta:101904037; F1MB88	ENSBTAG00000038970	-0.164011326	-0.282122729	-0.118111403
F1MUR2	KDM5B	F1MUR2	ENSBTAG00000006175	-0.104301231	-0.152318058	-0.048016827
Q6Y1E2	PLP2	bta:399683; Q3ZBT4; Q6Y1E2	ENSBTAG00000016093	-0.0133378	0.02486206	0.03819986
A2VDP2	PCMTD1	A2VDP2; bta:521261	ENSBTAG00000017492	-1.445052479	-1.385109939	0.05994254
E1BEQ6	C8H9orf40	bta:533577; E1BEQ6	ENSBTAG00000013231	0.205860697	-0.280845682	-0.486706379
A0AAF6YWS3	BLOC1S1	A0AAF6YWS3	ENSBTAG00000011918	0.018395268	-0.199651683	-0.218046951
A0A3Q1LVM5	ITIH1	A0A3Q1LVM5	ENSBTAG00000007843	-0.389042291	0.107383535	0.496425826
A0A452DJC9	TMEM70	A0A452DJC9	ENSBTAG00000012920	0.122216546	0.069393335	-0.05282321
Q2KJJ1	SLC25A17	bta:506631; Q2KJJ1	ENSBTAG00000006200	0.080489918	-0.10418885	-0.184678768
A0A3Q1LHC4	HOMER2	A0A3Q1LHC4	ENSBTAG00000017715	0.200957152	0.26710407	0.066146918
A0AAA9SB84	SLC7A2	A0AAA9SB84	ENSBTAG00000010085	-0.008562014	-0.48713517	-0.478573156
A5PJR2	MCL1	A5PJR2; bta:788087	ENSBTAG00000015154	0.021772109	-0.385237486	-0.407009596
F6RR15	LSR	F6RR15	ENSBTAG00000017033	0.0971152	0.219599206	0.122484007
A0A3S5ZPU2	AGPAT5	A0A3S5ZPU2	ENSBTAG00000004922	0.023171891	0.024266085	0.001094194
Q5E9E8	YIPF5	A1L5B9; bta:521829; Q5E9E8	ENSBTAG00000012758	-0.070727711	-0.019072733	0.051654977
A2VE72	FAM8A1	A2VE72; bta:511911	ENSBTAG00000017118	0.032773741	0.039801008	0.007027267
A0A3Q1LR05	INTS11	A0A3Q1LR05	ENSBTAG00000007620	-0.145452277	-0.092244911	0.053207366
A0AAA9T2W9	GSTCD	A0AAA9T2W9	ENSBTAG00000013927	-0.314970544	-0.292018094	0.022952451
A0AAA9TNB8	POLR3H	A0AAA9TNB8	ENSBTAG00000005311	0.030936167	-0.015720458	-0.046656625
Q3SZ68	LAMTOR5	bta:516090; Q3SZ68	ENSBTAG00000014970	0.263034406	0.355890506	0.0928561
P62866	FAU	P62866; Q8HYI8		-0.264482168	0.132627991	0.397110159
F1MGP5	MTRR	F1MGP5	ENSBTAG00000009401	-0.021041137	-0.081643238	-0.060602101
A0A3Q1MAZ0	MEX3D	A0A3Q1MAZ0	ENSBTAG00000040334	-0.11647218	-0.113241058	0.003231121
F1N2C1	C3H1orf43	F1N2C1	ENSBTAG00000020026	0.403839293	0.401338954	-0.002500339
Q3SZT5	RABL2B	bta:515671; Q3SZT5	ENSBTAG00000004876	-0.160142438	0.137503524	0.297645962
A0A3Q1MEI1	VPS13B	A0A3Q1MEI1	ENSBTAG00000012357	-0.092179533	-0.114663547	-0.022484014
A0AAA9T9X9	DIABLO	A0AAA9T9X9	ENSBTAG00000004199	0.076364825	0.215616011	0.139251186
G5E5C4	PDCD11	G5E5C4	ENSBTAG00000009715	-0.613186779	-0.295996604	0.317190176
A0A3Q1NMJ3	NCBP2AS2	A0A3Q1NMJ3; bta:783161	ENSBTAG00000048390	0.413801786	0.192439199	-0.221362587
A0AAA9S7S7	DNAJC16	A0AAA9S7S7	ENSBTAG00000002476	-0.045156181	0.103736218	0.148892399
A0AAF7AQ98	IGF2	A0AAF7AQ98	ENSBTAG00000013066	0.404066381	0.013187432	-0.390878949
A0AAA9SAY2	TRIT1	A0AAA9SAY2	ENSBTAG00000013832	-0.423499078	-0.192973822	0.230525256
A0AAA9S8W1	NAPEPLD	A0AAA9S8W1	ENSBTAG00000014171	0.017372564	-0.019193565	-0.036566129
A0AAA9SDZ4	SYNE3	A0AAA9SDZ4	ENSBTAG00000003526	0.155694317	0.085362193	-0.070332125
E1BG09	RTTN	E1BG09	ENSBTAG00000013221	0.086749519	0.056466952	-0.030282567
E1BN04	ZMYND11	E1BN04	ENSBTAG00000002578	-0.05294888	-0.296164999	-0.243216119
A0AAA9STE8	PLPP1	A0AAA9STE8	ENSBTAG00000010526	-0.060353907	0.248733842	0.309087749
Q2TBX9	ITFG1	bta:512545; Q2TBX9	ENSBTAG00000004805	-0.072956033	0.074166019	0.147122052
A0A3Q1LT78	DCAF6	A0A3Q1LT78	ENSBTAG00000020969	-0.153772186	-0.324250474	-0.170478288
Q0P572	MBIP	bta:774027; Q0P572		-0.091810264	-0.310096443	-0.218286178
A0AAA9SAV7	AGPAT2	A0AAA9SAV7	ENSBTAG00000025161	0.023999319	0.054792473	0.030793154
A0AAA9SUE0	TPK1	A0AAA9SUE0	ENSBTAG00000017676	0.062797828	0.179456306	0.116658478
A0A3Q1M864	GAB2	A0A3Q1M864	ENSBTAG00000002922	-0.081136763	-0.12963528	-0.048498518
A2VE11	IGSF8	A2VE11; bta:519266		0.293980627	0.082883385	-0.211097242
Q32L97	VAMP4	A5PK89; bta:616923; Q32L97	ENSBTAG00000020591	0.050923935	-0.079923544	-0.130847479
E1BJ67	CCNL1	bta:514613; E1BJ67	ENSBTAG00000003851	0.029503994	-0.050555104	-0.080059098
A0A3Q1LHL4	USP12	A0A3Q1LHL4	ENSBTAG00000017179	0.051289229	-0.0138058	-0.065095028
A7MB73	CERCAM	A7MB73; bta:516122	ENSBTAG00000038139	-0.331543184	-0.188337039	0.143206145
A0AAF7AMH2	ETV6	A0AAF7AMH2	ENSBTAG00000014605	0.294447358	0.226275856	-0.068171503
A0AAA9TUT1	PGAP1	A0AAA9TUT1; bta:504677	ENSBTAG00000020416	-0.682552328	-0.595485487	0.087066841
A0AAF6Z0C8	PTCD2	A0AAF6Z0C8	ENSBTAG00000014675	-0.096488083	-0.091423028	0.005065055
F1MML3	BCORL1	bta:540023; F1MML3	ENSBTAG00000004962	-0.073704005	-0.04580369	0.027900316
A6H728	MUTED	A6H728; bta:505520		0.168447587	0.030681253	-0.137766334
A0A3Q1MFU2	RADX	A0A3Q1MFU2; bta:511907	ENSBTAG00000003668	-0.024060898	-0.069563594	-0.045502695
Q28024	GNG12	A5PJE1; bta:286850; Q28024	ENSBTAG00000061772	0.003680346	-0.085391491	-0.089071838
F6RMS7	CENPQ	F6RMS7	ENSBTAG00000020710	0.364261795	0.153069106	-0.21119269
F1N5Q1	ZNF691	F1N5Q1	ENSBTAG00000016028	0.23786383	0.190942783	-0.046921047
A0AAA9T3J5	RRAD	A0AAA9T3J5	ENSBTAG00000013929	0.315840765	0.390146324	0.074305559
A7E301	SHROOM1	A7E301; bta:615958	ENSBTAG00000014676	0.104785025	0.190232727	0.085447703
A0A3Q1LLM3	HOMER1	A0A3Q1LLM3	ENSBTAG00000025853	0.307783913	0.263278744	-0.044505169

Accession	Gene_Symbol	Gene_ID	Ensembl_Gene ID	log2(ratio(CM12H/CM0H))	log2(ratio(CM12R/CM0H))	log2(ratio(CM12H/CM12R))
F1N7E7	TSHZ3	F1N7E7	ENSBTAG00000002687	0.143590854	-0.018417634	-0.162008487
Q5EA18	CENPL	A0JNC6; bta:615527; Q5EA18	ENSBTAG00000006925	0.13317327	0.066609669	-0.066563601
A0AAA9TEA9				-0.133775998	0.102499363	0.236275361
E1B9E6	TRAF3IP1	E1B9E6	ENSBTAG00000015973	0.020102904	-0.055018871	-0.075121774
Q2KJ51	ANGPTL4	bta:509963; Q2KJ51	ENSBTAG00000002473	-0.200040184	-0.335277649	-0.135237464
Q3SZV5	POMP	bta:510314; Q3SZV5	ENSBTAG00000014024	-0.065033885	0.418015199	0.483049084
A2VDV9	SMIM8	A2VDV9; bta:533179	ENSBTAG00000021663	0.074169703	0.741356335	0.667186632
A0AAA9SZM8	HTATIP2	A0AAA9SZM8	ENSBTAG00000013419	-0.104264814	-0.076725791	0.027539024
Q05B49				-0.409678583	-0.254774679	0.154903905
Q3T0U4	ADIPOR1	bta:407235; F1MRN0; Q3T0U4	ENSBTAG00000009727	0.214870302	-0.085919026	-0.300789328
A0AAF6Z555	POLG2	A0AAF6Z555	ENSBTAG00000018420	-0.04735277	0.052669069	0.100021839
A0AAA9RX60	ILRUN	A0AAA9RX60	ENSBTAG00000034493	0.030102032	0.073421069	0.043319038
A0AAA9RU38	WDSUB1	A0AAA9RU38	ENSBTAG00000004184	-0.089498151	-0.090941568	-0.001443417
A0AAA9TZZ2	CCDC97	A0AAA9TZZ2	ENSBTAG00000039635	-0.072541102	-0.121501005	-0.048959903
A0A3Q1LTZ7	PLXNC1	A0A3Q1LTZ7	ENSBTAG00000011245	-0.22304243	-0.289398311	-0.06635588
E1BMD3	MSRB3	E1BMD3	ENSBTAG00000044017	-0.378835134	-0.437495169	-0.058660035
A6QQR2	CLCN7	A6QQR2		0.150543245	0.371056545	0.2205133
E1BGZ9	PHF20L1	E1BGZ9	ENSBTAG00000006869	-0.116723606	-0.057959604	0.058764003
A0A3Q1NLB0				-0.029197641	-0.209257019	-0.180059379
A0AAA9S157	ADAT3	A0AAA9S157	ENSBTAG00000062956	0.08496956	0.019628807	-0.065340754
A0AAA9SA51	RPUSD3	A0AAA9SA51	ENSBTAG00000007968	-0.146423567	-0.165981413	-0.019557846
A0AAA9S206	PHF20	A0AAA9S206; bta:529591	ENSBTAG00000005571	-1.223152735	-1.12705191	0.096100825
A0A3Q1LJJ3	ETS1	A0A3Q1LJJ3	ENSBTAG00000002341	-0.353964468	-0.411603094	-0.057638626
Q2M2U3	LAMTOR4	bta:511903; Q2M2U3		0.079906867	-0.090512293	-0.17041916
A0AAA9S6C8	AXIN1	A0AAA9S6C8	ENSBTAG00000016577	0.060212807	-0.050358297	-0.110571104
A0A3Q1LPR8	SNX24	A0A3Q1LPR8	ENSBTAG00000031069	0.172872285	0.060662782	-0.112209504
A0AAA9TFR4	GM2A	A0AAA9TFR4	ENSBTAG00000021829	0.198246334	0.020801319	-0.177445015
F1MIH4	CLIC5	F1MIH4	ENSBTAG00000010244	-0.025261843	-0.149377624	-0.124115781
A0AAA9RXQ2	PKM	A0AAA9RXQ2	ENSBTAG00000001601	0.647199633	0.957539753	0.310340121
E1B9A0	SLC36A1	bta:518759; E1B9A0	ENSBTAG00000016094	-0.040971781	0.12035194	0.161323721
E1BFC5	FAM222B	bta:506074; E1BFC5	ENSBTAG00000047717	-0.071356606	-0.123773855	-0.052417249
A0AAA9S626	DMAC2L	A0AAA9S626	ENSBTAG00000015202	-0.022184369	0.037662853	0.059847222
A0AAF6YY86	UNC119	A0AAF6YY86	ENSBTAG00000013096	-0.118333269	0.130838483	0.249171752
A0AAA9S1W0	FCHSD1	A0AAA9S1W0	ENSBTAG00000049248	0.031288589	0.314528739	0.28324015
F1MBE6	E2F4	F1MBE6	ENSBTAG00000012063	-0.179431582	-0.272356392	-0.09292481
E1BM52	FOXP4	bta:517805; E1BM52	ENSBTAG00000014211	-0.180741288	-0.324148358	-0.14340707
A0A3Q1LTK4	DLG5	A0A3Q1LTK4	ENSBTAG00000013187	0.165337732	0.023846742	-0.14149099
F6RQN4	C1QTNF3	F6RQN4	ENSBTAG00000017071	0.270366752	0.202746909	-0.067619843
F1MS13	PHF21A	F1MS13	ENSBTAG00000009611	-0.288957751	-0.219718668	0.069239083
F1MQ33	SCAP	F1MQ33	ENSBTAG00000015782	-0.115147873	-0.297433533	-0.18228566
A0A3Q1MGD5	IRF5	A0A3Q1MGD5	ENSBTAG00000004989	-0.24610401	-0.301995211	-0.055891201
A0AAF6DLG7	USP38	A0AAF6DLG7	ENSBTAG00000001356	-0.298166277	-0.110306262	0.187860015
Q17QE3	POLR2M	bta:100125877; Q17QE3	ENSBTAG00000040384	-0.069086738	-0.088517268	-0.01943053
A0AAF6DM51	SELENON	A0AAF6DM51	ENSBTAG00000021778	0.060444898	0.798762914	0.738318015
A0AAA9T4M0	MYO19	A0AAA9T4M0	ENSBTAG00000012675	-0.009905743	-0.037140735	-0.027234992
F1N3J9	OGFRL1	F1N3J9	ENSBTAG00000016374	-0.230612928	-0.184153617	0.046459311
Q05589	PLAU	A7E3W6; bta:281408; Q05589; Q0IIG3; Q28209	ENSBTAG00000005947	-0.644833957	-0.12659757	0.518236387
Q0IIH0	WDR45	bta:539190; Q0IIH0		-0.081573801	-0.260016214	-0.178442413
F1MW52	ADAM12	F1MW52	ENSBTAG00000012444	-0.106839038	-0.554588852	-0.447749814
A0AAA9SUN1	MTERF3	A0AAA9SUN1	ENSBTAG00000001522	-0.173331603	-0.18957834	-0.016246737
A0AAA9SG79	GPR180	A0AAA9SG79	ENSBTAG00000005310	0.130245368	0.048194712	-0.082050655
Q58DL9	PLTP	bta:505640; Q58DL9	ENSBTAG00000000559	-0.87063471	-0.450341486	0.420293225
A0A3Q1LU95	STAMBPL1	A0A3Q1LU95	ENSBTAG00000001298	-0.567276	-0.276571345	0.290704656
Q2M2S4	PNKD	Q2M2S4	ENSBTAG00000047277	0.192806228	-0.159327528	-0.352133756
A0A3Q1N125	C7H19orf67	A0A3Q1N125	ENSBTAG00000016937	0.054792473	-0.179597482	-0.234389954
B0JYN5	MRFAP1	B0JYN5; bta:522338		-0.450696282	-1.150957283	-0.700261001
A0AAA9RVV3	SCAPER	A0AAA9RVV3	ENSBTAG00000007382	-0.157111939	-0.269250249	-0.11213831
A4FUY5	TOB2	A4FUY5; bta:507934	ENSBTAG00000015109	-0.238842942	-0.169399334	0.069443608
F6Q268	ACACB	F6Q268	ENSBTAG00000022058	-0.282792648	-0.135808657	0.146983992
A6QNP8	ESCO2	A6QNP8; bta:538949	ENSBTAG00000006551	-0.464898014	-0.117201211	0.347696803
A0AAA9S3B4	ZDHHC17	A0AAA9S3B4; bta:541172	ENSBTAG00000021756	-0.013406659	0.048118595	0.061525254
A6QQJ8	ZC3H12A	A6QQJ8; bta:535344	ENSBTAG00000011316	0.162803625	0.154086408	-0.008717217
A0AAF7A521	TIPIN	A0AAF7A521	ENSBTAG00000000372	0.053989276	0.0366634	-0.017325876
F6PT78	TDG	F6PT78	ENSBTAG00000021633	-0.309626499	-0.07609726	0.23352924
A0AAA9RWM0	SFT2D3	A0AAA9RWM0; bta:514114	ENSBTAG00000066017	-0.13131169	0.044436854	0.175748544
A0AAA9SJV5	GINS2	A0AAA9SJV5	ENSBTAG00000044006	0.149424549	0.312738923	0.163314374
F6QNS8	ZC3H8	F6QNS8	ENSBTAG00000015965	-0.454846064	-0.929353393	-0.474507328
A0A3Q1MJL6	GPRC5C	A0A3Q1MJL6	ENSBTAG00000018663	0.048498518	-0.099976124	-0.148474642
F1N547	CETN3	F1N547	ENSBTAG00000014373	0.233404058	-0.266533062	-0.49993712
G3MYM4	ZNF292	G3MYM4	ENSBTAG00000046612	-0.193250786	-0.123861913	0.069388873
A0AAA9S1Q8	FUT11	A0AAA9S1Q8; bta:539329	ENSBTAG00000002792	-0.077837434	-0.165104271	-0.087266836
A4IFN5	FITM2	A4IFN5; bta:518159	ENSBTAG00000020030	0.201415288	0.194842634	-0.006572654
E1BD50	TTC33	bta:531278; E1BD50	ENSBTAG00000000012	0.25287454	0.332217971	0.079343431
Q0P5D9	ALG8	bta:538731; Q0P5D9		-0.188144962	-0.127357244	0.060787718
A5PJN5	TCAF1	A5PJN5; bta:533126		-0.169058258	-0.072541102	0.096517155
A0AAA9SRG3	NARF	A0AAA9SRG3	ENSBTAG00000018178	-0.044616775	-0.222986245	-0.178369471
Q6L708	CLDN1	bta:414922; Q3ZC38; Q6L708	ENSBTAG00000013148	0.065210433	0.402557929	0.337347496
A0AAF6YMI8	KDELR1	A0AAF6YMI8	ENSBTAG00000004463	0.545618196	0.344470664	-0.201147532
A0A3Q1LLN6	MTSS1	A0A3Q1LLN6	ENSBTAG00000020407	0.0169446	0.039627074	0.022682474
A6QLD8	ADAMTSL4	A6QLD8; bta:507654		0.008158523	0.070519189	0.062360666
A0AAA9RZI0	AHI1	A0AAA9RZI0	ENSBTAG00000017958	-0.159588757	-0.630354404	-0.470765647
Q2YDH1	ANAPC10	bta:783986; Q2YDH1		-0.17351469	-0.079596826	0.093917863
Q1JQ94	RTL8A; RTL8B; RTL8C	bta:511516; bta:615809; bta:783730; Q1JQ94	ENSBTAG00000039890; ENSBTAG00000056216; ENSBTAG00000062559	0.030507464	0.146468369	0.115960905
A8E659	ANKH	A8E659; bta:511800		-0.075639077	-0.139142019	-0.063502942
A0AAF6YYH7	AP5M1	A0AAF6YYH7	ENSBTAG00000013043	0.116917752	0.109700653	-0.007217099
A0AAA9S3U7	ICOSLG	A0AAA9S3U7	ENSBTAG00000014880	0.004075412	0.098583085	0.094507673
G3N047	TTC7B	G3N047	ENSBTAG00000006392	-0.050098612	0.225905194	0.276003806
F1N5H2	FUCA2	bta:515729; F1N5H2	ENSBTAG00000001752	0.055531947	-0.003909745	-0.059441692

Accession	Gene_Symbol	Gene_ID	Ensembl_Gene ID	log2(ratio(CM12H/CM0H))	log2(ratio(CM12R/CM0H))	log2(ratio(CM12H/CM12R))
A0A3Q1M939	RNF121	A0A3Q1M939	ENSBTAG00000014167	0.162066777	0.099108145	-0.062958632
F1MHF3	NAV2	F1MHF3	ENSBTAG00000018431	0.176412051	0.16925226	-0.007159792
A0AAF6ZGE1	BOLA1	A0AAF6ZGE1	ENSBTAG00000006050	-0.145850866	-0.067617579	0.078233288
A0A452DIY8	MRPL55	A0A452DIY8; bta:506657	ENSBTAG00000020721	-0.137036708	-0.189313987	-0.052277279
A0AAF6DLW8	NRADD	A0AAF6DLW8	ENSBTAG00000022598	0.024453044	0.01227833	-0.012174714
Q95LI3	ZFY	bta:280962; Q95LI3		0.026600662	-0.126944596	-0.153545258
Q3T139	RNF146B	bta:767901; Q3T139	ENSBTAG00000034531	0.170595555	-0.124433776	-0.29502933
Q5E950	NHP2	bta:520891; Q3SZ42; Q5E950	ENSBTAG00000006374	-0.57985561	-0.309476354	0.270379256
Q148C8	MIEN1	bta:505710; Q148C8	ENSBTAG00000021802	-0.020576361	0.322556583	0.343132945
Q58DH7	SIGMAR1	bta:538903; Q58DH7	ENSBTAG00000015804	-0.134875285	-0.015310945	0.11956434
A1A4I1	SLC20A2	A1A4I1; bta:518905		-0.062163835	0.003505944	0.065669779
Q3ZC85	PLVAP	bta:524990; F1MHE4; Q3ZC85	ENSBTAG00000005434	-0.752268705	-0.771231016	-0.018962311
A3KMZ6	COX11	A3KMZ6; bta:510509	ENSBTAG00000007015	0.02822316	-0.101184634	-0.129407794
F1N3Q9	PNKP	F1N3Q9	ENSBTAG00000001834	-1.153756065	-0.740912779	0.412843286
A0AAA9RYE9	PNPLA8	A0AAA9RYE9	ENSBTAG00000024387	-0.592476554	-0.544320516	0.048156038
A0AAA9PWD1	FDX2	A0AAA9PWD1; bta:505159	ENSBTAG00000010318	0.140321288	0.30256277	0.162241482
E1BEH8	SLC35B4	bta:613784; E1BEH8	ENSBTAG00000003372	0.17872195	0.031097297	-0.147624653
Q3ZBZ7	CCDC109B	bta:504728; Q3ZBZ7		-0.443606651	-0.177369814	0.266236838
A0AAA9S3X9	SLC39A7	A0AAA9S3X9	ENSBTAG00000008191	0.137766334	-0.084845093	-0.222611426
F1N4N7	CACHD1	F1N4N7	ENSBTAG00000016455	-0.074742772	-0.15487777	-0.080134998
A0AAA9TUV8	MTURN	A0AAA9TUV8	ENSBTAG00000066764	-0.223954625	0.377069649	0.601024274
A0AAA9RXW4	C6H4orf3	A0AAA9RXW4	ENSBTAG00000048273	0.514879445	0.66731121	0.152431765
Q3ZC71	DPM3	bta:509745; Q3ZC71	ENSBTAG00000003535	0.162870244	0.110053545	-0.052816698
A0A3S7IDQ1	MDA-5	A0A3S7IDQ1		0.148214004	0.269589142	0.121375139
A0AAA9T6R4	NET1	A0AAA9T6R4	ENSBTAG00000007648	-0.117334741	0.0777956	0.195130341
Q08D99	TMEM41A	bta:505368; Q08D99	ENSBTAG00000017115	0.179158532	0.060870564	-0.118287967
A0A3Q1MLN4	PACRGL	A0A3Q1MLN4	ENSBTAG00000033351	-0.202710055	-0.551937195	-0.34922714
A0AAA9SBK8	ULK1	A0AAA9SBK8	ENSBTAG00000012987	0.140034322	0.303232001	0.16319768
Q2KJ54	SPINT2	bta:507484; Q2KJ54	ENSBTAG00000000182	-0.237539525	-0.240545141	-0.003005616
E1BHQ9	MCAM	E1BHQ9	ENSBTAG00000006835	0.224476557	-0.16578337	-0.390259926
A0AAA9T5S7	LCLAT1	A0AAA9T5S7	ENSBTAG00000054158	0.099428446	0.140862536	0.04143409
F1MHQ4	FABP4	F1MHQ4	ENSBTAG00000037526	0.353356149	0.276532653	-0.076823495
A6QR31	FAM32A	A6QR31; bta:508634	ENSBTAG00000046846	0.462526425	0.451146197	-0.011380228
F1N076	CP	F1N076	ENSBTAG00000012164	-0.101695397	0.097822558	0.199517955
A0AAA9SKP9	ZBTB21	A0AAA9SKP9	ENSBTAG00000030836	-0.187506904	-0.229133999	-0.041627096
E1BER4	CRACR2A	bta:525377; E1BER4; G3MY43	ENSBTAG00000018940	-0.5662078	-0.712539552	-0.146331752
A0A452DJ42	ATP5PF	A0A452DJ42	ENSBTAG00000000605	-0.410868405	-0.746867975	-0.33599957
A0A3Q1MVZ0	ANTXR1	A0A3Q1MVZ0; bta:616010	ENSBTAG00000007808	-0.05625488	-0.250644662	-0.194389781
A0AAA9T1V8	AVL9	A0AAA9T1V8	ENSBTAG00000003114	-0.088668062	-0.031478231	0.05718983
Q3SX46	C1GALT1C1	bta:531644; Q3SX46	ENSBTAG00000021410	-0.055358593	0.087790466	0.143149059
E1BCZ0	POLRMT	bta:504757; E1BCZ0	ENSBTAG00000015051	-0.793747992	-0.660481461	0.133266531
A0AAF7APJ1	COA6	A0AAF7APJ1	ENSBTAG00000051467	0.02774499	0.186601755	0.158856764
A0AAF6DLT4	ST3GAL1	A0AAF6DLT4	ENSBTAG00000001156	-0.153211128	-0.00135911	0.151852018
A0AAA9S8E2	PRSS23	A0AAA9S8E2	ENSBTAG00000015177	-0.684312845	-0.57397664	0.110336205
F6Q4D3	UBA7	F6Q4D3	ENSBTAG00000012335	-0.183259967	-0.072588	0.110671967
A0A3Q1MH81	TTC38	A0A3Q1MH81	ENSBTAG00000003017	-0.04193068	0.035257325	0.077188005
Q6QRN8	LAPTM4A	bta:404135; Q6QRN8	ENSBTAG00000020894	0.202546423	0.058773709	-0.143772714
A0A3Q1MCU3	CDC25B	A0A3Q1MCU3	ENSBTAG00000008436	0.246673977	0.365080839	0.118406862
Q1JQC2	IER3IP1	bta:101906131; bta:768079; Q1JQC2	ENSBTAG00000017330	-0.352148164	-0.532808168	-0.180660003
A0AAA9TTD4	WDR37	A0AAA9TTD4	ENSBTAG00000000683	0.051406225	0.064991024	0.013584799
A7E348	PYGO2	A7E348; bta:540401		0.433033929	0.548969654	0.115935725
A0AAF6DLW0	ADPRM	A0AAF6DLW0	ENSBTAG00000046919	-0.046194823	-0.05246742	-0.006272597
E1BAP3	SP110	E1BAP3	ENSBTAG00000015752	0.034369296	0.092258508	0.057889212
A7E380	SAMD9	A7E380		-0.120839187	0.202791203	0.32363039
F1N7F8	PIGU	F1N7F8	ENSBTAG00000011620	0.148584383	0.109555086	-0.039029297
C9EGS9	CD19	bta:517359; C9EGS9		-0.132818036	-0.058531612	0.074286424
A6QLJ8	PTER	A6QLJ8; bta:782020	ENSBTAG00000016676	-0.589473947	-0.581748654	0.007725293
F1ML88	PUS7	F1ML88	ENSBTAG00000007743	-0.727215084	-0.559783938	0.167431146
F1MS81	RMND1	F1MS81	ENSBTAG00000013224	-0.06030051	0.059231122	0.119531632
A0AAA9T095	SLC30A7	A0AAA9T095	ENSBTAG00000019027	0.095347877	-0.081408329	-0.176756205
A0AAA9RXB9	TANK	A0AAA9RXB9	ENSBTAG00000006654	-0.079853308	0.131184219	0.211037526
A0A452DJ75	CALML4	A0A452DJ75	ENSBTAG00000025822	-0.104997251	-0.273936064	-0.168938813
A0A3Q1M3Z8	QPRT	A0A3Q1M3Z8	ENSBTAG00000018082	-0.158327939	0.358272203	0.516600142
E1BPN4	CHST3	bta:506758; E1BPN4	ENSBTAG00000011014	-0.264794864	0.053252646	0.318047509
A0A3Q1NN05	CASTOR2	A0A3Q1NN05	ENSBTAG00000014417	-0.193569586	0.050849141	0.244418728
P0CG11	CHTF8	A8E4M1; bta:618186; P0C6S9; P0CG11		-0.014691522	0.027508315	0.042199838
A0A3Q1NCH3	CEP152	A0A3Q1NCH3	ENSBTAG00000011661	0.204963275	-0.19523609	-0.400199365
A0A3Q1M0G5	DNAJC24	A0A3Q1M0G5	ENSBTAG00000050888	-0.05708737	-0.028261335	0.028826035
A5PJQ7	PXK	A5PJQ7; bta:614093		-0.764631651	-0.71930766	0.045323991
F6PN06	TNK2	F6PN06	ENSBTAG00000021364	0.187707155	0.05639392	-0.131313235
A0A3Q1MPU3	TRAK1	A0A3Q1MPU3	ENSBTAG00000016076	-0.253257284	0	0.253257284
A6QLN7	FMO5	A6QLN7; bta:788719	ENSBTAG00000010841	0.557612013	0.215117091	-0.342494922
A0A3Q1MTU3	RASSF1	A0A3Q1MTU3	ENSBTAG00000020963	0.045562577	0.094555232	0.048992655
F1MYN0	BOLA2B	F1MYN0	ENSBTAG00000008632	0.152688297	0.447230504	0.294542207
Q1RMQ5	LIN37	bta:534550; Q1RMQ5	ENSBTAG00000018594	0.25894915	0.064756208	-0.194192942
Q3ZCC5	RBM18	bta:508615; Q3ZCC5	ENSBTAG00000016367	1.461839264	-0.634067973	-2.095907237
E1B842	HIVEP2	bta:540396; E1B842	ENSBTAG00000001146	0.18223147	0.277077354	0.094845885
A0AAA9TD53	F7	A0AAA9TD53	ENSBTAG00000007411	0.231325546	0.289506617	0.058181071
F1N2C5	IL17RA	F1N2C5	ENSBTAG00000011067	0.283426195	-0.116226141	-0.399652336
G5E6T2	ARHGAP23	G5E6T2	ENSBTAG00000016911	0.149848209	0.02983523	-0.120012979
A0A3Q1MAX3	NDFIP1	A0A3Q1MAX3	ENSBTAG00000047747	0.035590108	0.069002788	0.03341268
A0AAA9TZP0	RAD51B	A0AAA9TZP0	ENSBTAG00000018971	-0.408216233	-0.168045936	0.240170297
Q3ZBS1	MACIR	bta:538782; Q3ZBS1	ENSBTAG00000055286	0.468593465	0.308060572	-0.160532893
F1MX42	PANK2	F1MX42	ENSBTAG00000007920	0.135306805	-0.012123559	-0.147430364
A4Z945	FAM200C	A4Z945; A5D7P2; bta:539846	ENSBTAG00000030282	0.426145067	0.314705146	-0.111439921
A0AAF7A5Q1	RHBDF1	A0AAF7A5Q1	ENSBTAG00000019805	-0.07001793	0.069610851	0.139628781
A7MB44	SLC9A6	A0A3S5ZP41; A7MB44; bta:533137; F1MH53	ENSBTAG00000009523	0.033015057	-0.103835811	-0.136850868
A0AAA9SQM0	DHX35	A0AAA9SQM0	ENSBTAG00000004039	0.084622078	0.317251608	0.23262953
A7MB07	ADAMTS1	A7MB07; bta:512171	ENSBTAG00000000706	-0.117789303	-0.155580688	-0.037791385

Accession	Gene_Symbol	Gene_ID	Ensembl_Gene ID	log2(ratio(CM12H/CM0H))	log2(ratio(CM12R/CM0H))	log2(ratio(CM12H/CM12R))
A0A452DIB0	PPP1R35	A0A452DIB0	ENSBTAG00000026243	-0.099257938	-0.27005104	-0.170793101
E1BKV9	ERCC4	bta:516371; E1BKV9	ENSBTAG00000021773	-0.068243176	0.168937192	0.237180368
E1BE19	AHDC1	bta:522105; E1BE19	ENSBTAG00000037456	0.010716447	-0.100208102	-0.110924549
A0AAA9SRJ8	GMEB1	A0AAA9SRJ8	ENSBTAG00000001160	-0.040019932	-0.026094196	0.013925736
A0A3Q1MIB9	ATF6B	A0A3Q1MIB9	ENSBTAG00000008794	-0.025173071	0.0803811	0.105554171
A0A3Q1MZB9	AMD1	A0A3Q1MZB9	ENSBTAG00000003342	0.084352168	-0.465607675	-0.549959843
A0A3Q1LZ05	USP54	A0A3Q1LZ05	ENSBTAG00000011108	0.041863519	0.049212974	0.007349456
Q2KJ69	RLIG1	bta:767986; Q2KJ69		-0.647050181	-0.44161475	0.205435431
F1MMS0	BRIP1	F1MMS0	ENSBTAG00000012068	0.074734798	0.07937618	0.004641382
F6RXG8	RGCC	F6RXG8	ENSBTAG00000034885	0.045061808	0.351793066	0.306731258
A0AAA9TRQ1	FAM133B	A0AAA9TRQ1	ENSBTAG00000050705	0.512602278	0.253703646	-0.258898631
A0AAA9SNQ3	FOXJ3	A0AAA9SNQ3	ENSBTAG00000015887	0.205739013	0.078644494	-0.127094519
A0AAA9TQL1	DDR2	A0AAA9TQL1	ENSBTAG00000004885	0.08912589	0.024542184	-0.064583706
A0A3Q1M4A1	BIN3	A0A3Q1M4A1	ENSBTAG00000005470	0.031535573	0.251028553	0.219492979
A0AAA9S745	CTDSPL	A0AAA9S745	ENSBTAG00000053223	-0.291766124	-0.130553554	0.16121257
A0A452DJ67	ATP6V0C	A0A452DJ67	ENSBTAG00000026428	0.12269133	0.196691911	0.074000581
Q08DK7	SLC25A29	bta:507686; Q08DK7	ENSBTAG00000013666	0.027107802	0.015122728	-0.011985074
A0A3Q1NB49	IGF1R	A0A3Q1NB49	ENSBTAG00000021527	-0.008208813	-0.206450877	-0.198242064
F1MGR3	RERE	bta:535394; F1MGR3	ENSBTAG00000018272	-0.054352365	-0.304052132	-0.249699767
F6RXK0	PDGFD	F6RXK0	ENSBTAG00000034827	-0.062574312	-0.36005164	-0.297477328
E1B7S7	ZNF24	bta:538969; E1B7S7; G3N356	ENSBTAG00000010255	0.155175872	0.058960556	-0.096215315
Q08DP3	RELL1	bta:768210; Q08DP3	ENSBTAG00000004329	-0.155278225	-0.234937675	-0.079659449
A0AAA9THB8				-0.258940199	-0.106323976	0.152616224
O46419	LIPT1	bta:286864; O46419	ENSBTAG00000059194	-0.402245946	-0.111582064	0.290663881
A0AAF6YXB8	ZFAND6	A0AAF6YXB8	ENSBTAG00000012321	0.057610723	-0.168422976	-0.226033699
A0A3Q1MIQ1	RNGTT	A0A3Q1MIQ1	ENSBTAG00000002725	-0.706106444	-0.468457357	0.237649087
A0AAA9SGZ0	GLCCI1	A0AAA9SGZ0	ENSBTAG00000008533	0.251193583	-0.013872175	-0.265065758
A0AAF6DM43	CENPT	A0AAF6DM43	ENSBTAG00000006404	-0.026940543	0.052417249	0.079357792
Q17QE5	CIB1	bta:510141; Q17QE5	ENSBTAG00000021275	0.201289172	0.284995654	0.083706482
A2VDK9	VMA21	A2VDK9; bta:613674	ENSBTAG00000006296	0.206948616	0.237672653	0.030724037
A0AAA9TEG6	PDSS2	A0AAA9TEG6	ENSBTAG00000021859	-0.430304256	-0.234465254	0.195839002
A6QNW7	CD5L	A6QNW7; bta:528593; F1N514	ENSBTAG00000022514	-0.189214173	0.122396631	0.311610804
E1BHA0	LZTS1	E1BHA0	ENSBTAG00000000841	0.410181571	-0.139702853	-0.549884424
A0A3Q1M728				0.106520324	0.104807926	-0.001712398
A0AAF6YI58	RSL24D1	A0AAF6YI58	ENSBTAG00000001342	-0.332758028	-0.597968211	-0.265210182
F6QEW8	JTB	F6QEW8	ENSBTAG00000014694	0.119598092	0.205096961	0.085498868
A0AAA9TJF2	CHCHD4	A0AAA9TJF2	ENSBTAG00000015529	-0.235761476	-0.114559764	0.121201711
Q0VC75	TP53I3	bta:508875; F1N047; Q0VC75		-0.128324097	-0.17954942	-0.051225323
F6PVQ3	SNAPIN	F6PVQ3	ENSBTAG00000012470	-0.210715266	-0.702026286	-0.49131102
Q0VC50	RTN4IP1	bta:506626; Q0VC50	ENSBTAG00000017554	-0.634119011	-0.576040425	0.058078586
A0AAA9TF92	RHBDD1	A0AAA9TF92	ENSBTAG00000023963	-0.390909228	-0.330271285	0.060637943
A0AAA9U1A6	TRUB2	A0AAA9U1A6	ENSBTAG00000004663	-0.471232039	-0.382624027	0.088608012
A0A3Q1MUR7	GASK1B	A0A3Q1MUR7	ENSBTAG00000017069	-0.307258406	-0.314762689	-0.007504282
A0AAA9U073	ZNF106	A0AAA9U073; bta:788468	ENSBTAG00000004079	0.291134941	-0.182864057	-0.473998998
A0AAA9SK59	PWWP2A	A0AAA9SK59	ENSBTAG00000010341	0.269415199	0.092149847	-0.177265353
A0AAA9SM30	TSTD2	A0AAA9SM30	ENSBTAG00000014722	-0.538037166	-0.587107773	-0.049070607
A0AAA9SWD1	PIBF1	A0AAA9SWD1	ENSBTAG00000019895	0.067462968	-0.027894006	-0.095356974
F6QPT2	PWWP2B	bta:524314; F6QPT2	ENSBTAG00000019519	0.039335894	-0.140497408	-0.179833302
E1BIT2	MIEF1	bta:613306; E1BIT2	ENSBTAG00000017439	0.03901366	-0.107929559	-0.14694322
Q08DZ6	RWDD4	A0A3Q1MQ19; bta:509865; Q08DZ6	ENSBTAG00000003081	-0.758361887	-1.037810088	-0.2794482
A0A3Q1MRQ2				-0.248107862	-0.295128036	-0.047020174
E1BK15	CLCN6	bta:520210; E1BK15	ENSBTAG00000020700	0.074254555	0.091838867	0.017584312
A0AAA9TJW2	EIF2AK1	A0AAA9TJW2	ENSBTAG00000007370	-0.004308699	0.132802486	0.137111185
G5E5E4	HDAC10	G5E5E4	ENSBTAG00000011000	0.140900361	0.289358747	0.148458386
A0AAA9STC4	SDHD	A0AAA9STC4	ENSBTAG00000016266	0.069559564	0.261449897	0.191890333
Q08E24	SARAF	bta:515461; Q08E24	ENSBTAG00000013579	0.891241685	0.101131134	-0.790110551
Q2KIW5	CENPU	Q2KIW5		0.133546189	0.042828972	-0.090717217
A0AAA9SLL5	MRPS24	A0AAA9SLL5	ENSBTAG00000007398	-0.200378798	0.022425763	0.222804561
E1B8R5	TNKS	bta:535030; E1B8R5	ENSBTAG00000001463	0.138976413	0.094122177	-0.044854236
A0A3Q1MFC2	SYF2	A0A3Q1MFC2	ENSBTAG00000001651	0.228350533	-0.13116493	-0.359515462
Q0P5H8	HERPUD2	bta:512265; Q0P5H8	ENSBTAG00000015105	-0.113156439	0.157037758	0.270194197
F6RSI9	DONSON	F6RSI9	ENSBTAG00000035175	0.026189524	0.180937994	0.15474847
F1N7C7	RHOD	F1N7C7	ENSBTAG00000020600	0.367822861	0.311690986	-0.056131875
F1MEM9	TTC19	F1MEM9	ENSBTAG00000013270	-0.115361733	-0.095976391	0.019385341
F6R4P6	SERPIND1	F6R4P6	ENSBTAG00000013973	-0.605008443	-0.245806602	0.359201841
A0AAA9S2I6	AGPAT4	A0AAA9S2I6	ENSBTAG00000007668	0.067399285	0.054168975	-0.01323031
A0AAA9TQM2	SNX33	A0AAA9TQM2	ENSBTAG00000012206	0.008970154	0.041391631	0.032421478
F1MJA5	FAM171A1	F1MJA5	ENSBTAG00000008787	-0.329646008	-0.148098639	0.181547369
E1BFC4	SLC9B2	E1BFC4	ENSBTAG00000040088	0.310799814	-0.066435653	-0.377235467
A0A3Q1LWL2	PPP1R13B	A0A3Q1LWL2	ENSBTAG00000003559	0.227341637	0.318950395	0.091608758
A0AAA9SPJ1	C18H19orf33	A0AAA9SPJ1	ENSBTAG00000063903	-0.039644753	-0.068644625	-0.028999872
F1MW44	F13A1	F1MW44	ENSBTAG00000007268	0.172408126	0.187217786	0.01480966
E1BBP0	CHFR	E1BBP0	ENSBTAG00000013951	-0.195995372	0.056742512	0.252737884
A0JN69	MARCHF3	A0JN69; bta:520348	ENSBTAG00000006797	-0.432660867	-0.133805986	0.298854881
G3X8E4	BCOR	bta:784529; G3X8E4	ENSBTAG00000047339	0.022150904	-0.607458817	-0.629609721
A0AAA9S540	ABHD4	A0AAA9S540	ENSBTAG00000016658	0.313325237	0.244039942	-0.069285295
A0A452DJ18	TMEM134	A0A452DJ18	ENSBTAG00000004786	0.010391606	0.208419479	0.198027873
Q08DP2	BORCS5	bta:614947; Q08DP2	ENSBTAG00000008989	-0.03805419	-0.066251082	-0.028196892
F1N4V1	NOS1AP	F1N4V1	ENSBTAG00000010158	0.00921853	0.13582108	0.12660255
Q17R00	EMX2	bta:523601; Q17R00	ENSBTAG00000003027	-0.128117517	-0.124239302	0.003878215
F1MDF3	ALS2	bta:535750; F1MDF3	ENSBTAG00000007395	0.127647823	-0.114021487	-0.24166931
Q5E9A9	DDA1	bta:512898; Q5E9A9	ENSBTAG00000017068	-0.244594736	-0.164058123	0.080536613
A0AAA9S081	SH2D3C	A0AAA9S081	ENSBTAG00000015375	0.473110951	1.044161145	0.571050194
A0A3Q1MQ77	ADGRL2	A0A3Q1MQ77	ENSBTAG00000012305	0.004077332	-0.013674937	-0.017752269
A0AAA9SMX8	CENPO	A0AAA9SMX8	ENSBTAG00000015014	-0.151117733	0.164305065	0.315422798
A0AAA9T0Y5	POT1	A0AAA9T0Y5	ENSBTAG00000013180	0.118721115	0.080681754	-0.038039361
A0A3Q1LWX2	RUSC2	A0A3Q1LWX2	ENSBTAG00000011403	0.200266306	0.228865266	0.02859896
E1BMZ2	MECOM	E1BMZ2	ENSBTAG00000005871	-0.253408382	-0.108076514	0.145331868
A0AAA9TZ96	STK11IP	A0AAA9TZ96	ENSBTAG00000038992	-0.422995021	-0.494981134	-0.071986113
A7MB97	YIPF2	A7MB97; bta:515893	ENSBTAG00000025606	0.075340562	0.008226366	-0.067114196
A8YXY8	AKIRIN2	A8YXY8; bta:614292	ENSBTAG00000003764	-0.326660833	-0.374862255	-0.048201422

Accession	Gene_Symbol	Gene_ID	Ensembl_Gene ID	log2(ratio(CM12H/CM0H))	log2(ratio(CM12R/CM0H))	log2(ratio(CM12H/CM12R))
Q29RJ0	KCTD18	bta:613694; Q29RJ0	ENSBTAG00000002272	-0.47333614	-0.25307002	0.22026612
Q5E9V1	DCUN1D3	bta:504926; Q5E9V1	ENSBTAG00000032260	0.030012569	0.100401897	0.070389328
A0AAA9T6D8	UNC5B	A0AAA9T6D8	ENSBTAG00000014270	0.31937239	0.276096949	-0.043275441
A0A3Q1M7M7	FGFR1OP2	A0A3Q1M7M7	ENSBTAG00000004532	0.064379228	0.042310805	-0.022068423
A0A3Q1N2B6				0.104939247	0.069154672	-0.035784575
F6Q447	SERPINB9	F6Q447	ENSBTAG00000031267	0.073358017	0.134831247	0.06147323
A0AAA9S4A6	ELOVL5	A0AAA9S4A6	ENSBTAG00000003359	0.099359832	-0.077196844	-0.176556676
Q3SZM3	CYBC1	bta:618343; Q3SZM3	ENSBTAG00000018176	0.36377938	0.229375094	-0.134404285
Q05B57	YOD1	bta:539931; Q05B57		0.160146267	0.035845199	-0.124301068
A0AAA9SKL1	ITPKB	A0AAA9SKL1	ENSBTAG00000053007	-0.022848589	0.01449957	0.037348159
E1BIN3	ARSI	bta:540390; E1BIN3	ENSBTAG00000012834	-0.332460186	-0.003112612	0.329347574
Q56JY0	HIKESHI	bta:504867; Q56JY0	ENSBTAG00000019995	0.228859017	0.076569134	-0.152289883
A0AAA9TTE3	REEP6	A0AAA9TTE3	ENSBTAG00000002311	0.057333175	0.266280065	0.20894689
A0A3Q1M5N5	SPRYD3	A0A3Q1M5N5	ENSBTAG00000021464	0.053573357	-0.185497805	-0.239071162
Q0VC73	DNAAF2	bta:534465; Q0VC73	ENSBTAG00000004930	0.069285295	-0.029264595	-0.098549889
A2VE01	CLP1	A2VE01; bta:506507	ENSBTAG00000006493	-0.001453597	-0.138666284	-0.137212687
A0AAA9SS87	POC5	A0AAA9SS87	ENSBTAG00000007114	-0.162271429	0.159002234	0.321273663
A0AAA9S8B7	C1D	A0AAA9S8B7	ENSBTAG00000049002	-0.025671408	-0.033462759	-0.007791351
A0A3Q1MSE3	WDR20	A0A3Q1MSE3	ENSBTAG00000007007	0.221702961	0.165450415	-0.056252546
A0AAA9SRU6	CCDC102A	A0AAA9SRU6	ENSBTAG00000000462	0.025814318	-0.157318673	-0.183132991
A0A3Q1MAE0	CLOCK	A0A3Q1MAE0; bta:100300400	ENSBTAG00000044044	-0.131117552	-0.204503436	-0.073385884
F1MGL5	TMEM67	F1MGL5	ENSBTAG00000044190	0.318447526	0.343322195	0.024874669
A0A3Q1MED0	FERRY3	A0A3Q1MED0	ENSBTAG00000053303	-0.083879078	-0.109624491	-0.025745413
A0AAA9S753	RBX1	A0AAA9S753	ENSBTAG00000053583	-0.0686764	0.432075944	0.500752344
A0AAA9S0E6	TK2	A0AAA9S0E6	ENSBTAG00000005152	-0.301463992	0.008220507	0.309684499
F1MSU8	PLEKHG2	F1MSU8	ENSBTAG00000004017	-0.103516369	-0.230023223	-0.126506854
A0AAA9T6U6	GABPB1	A0AAA9T6U6	ENSBTAG00000022801	-0.104272994	-0.137503524	-0.03323053
A0A452DIJ9	SERPINE1	A0A452DIJ9	ENSBTAG00000014465	-0.80054439	-0.982808699	-0.182264309
A0AAA9S0Y3	RIOX2	A0AAA9S0Y3	ENSBTAG00000015546	-0.565158552	-0.701187681	-0.136029129
A0AAA9S3W1	TRAPPC1	A0AAA9S3W1	ENSBTAG00000047214	-0.202475139	-0.030070854	0.172404286
A0AAF6ZG22	FASTKD3	A0AAF6ZG22	ENSBTAG00000009400	-0.129532813	-0.156005024	-0.026472211
A0AAA9RWA7	DENND6A	A0AAA9RWA7	ENSBTAG00000018433	0.017440448	-0.088437965	-0.105878413
A6QLS5	OTULINL	A6QLS5; bta:534389; F1N7I7	ENSBTAG00000000672	0.002468256	-0.014898876	-0.017367132
A0A3S5ZPT8	MAD2L1BP	A0A3S5ZPT8	ENSBTAG00000009548	-0.066109796	-0.071642669	-0.005532874
A0AAF6Z5K6				0.180485911	0.23262953	0.052143619
Q3ZCG2	MPC1	bta:767977; Q3ZCG2	ENSBTAG00000027879	-0.07498704	-0.016961992	0.058025048
P00430	COX7C	bta:101902937; bta:101903567; bta:327718; P00430; Q3T0U0; Q56K07	ENSBTAG00000039555	0.014412658	0.088484942	0.074072284
A0AAA9RY04	ATF6	A0AAA9RY04	ENSBTAG00000005227	0.375509135	-0.134419974	-0.509929109
A0A3Q1NB78	ZDHHC3	A0A3Q1NB78	ENSBTAG00000011484	0.129937152	-0.031836087	-0.161773239
A0AAA9SWS7	LLGL2	A0AAA9SWS7	ENSBTAG00000020067	0.240441058	0.263648449	0.023207391
F1ML55	MIER3	F1ML55	ENSBTAG00000014248	0.043029793	-0.019991475	-0.063021268
E1BLB1	SHPRH	E1BLB1	ENSBTAG00000008112	-0.222392421	-0.176257963	0.046134458
A0AAA9T977	SOWAHC	A0AAA9T977	ENSBTAG00000068476	-0.059545942	-0.062514452	-0.002968509
A6H7B7	SFRS5	A6H7B7; bta:510474		-0.734390338	-0.241108527	0.49328181
F1MJ20	ARHGAP28	bta:530550; F1MJ20	ENSBTAG00000019036	0.023945696	0.017996429	-0.005949266
A6QLT2	MTMR2	A6QLT2; bta:536810	ENSBTAG00000016557	0.040641984	-0.044749507	-0.085391491
P00741	F9	F1MFL4; P00741		-0.252665432	-0.019045756	0.233619677
A2VDU6	AP1AR	A2VDU6; bta:533664	ENSBTAG00000003941	-0.160299415	-0.082379529	0.077919886
E1BMP7	DNA2	E1BMP7		-0.063096835	-0.102672347	-0.039575512
A0AAA9TZC1	STX16	A0AAA9TZC1; bta:519624	ENSBTAG00000003872	-0.090480746	0.356874474	0.44735522
F1N3Y8	FANCE	F1N3Y8	ENSBTAG00000019492	-0.266183459	0.086032306	0.352215764
A0AAA9S1M3	ENDOG	A0AAA9S1M3	ENSBTAG00000012484	-0.160302417	-0.220095502	-0.059793085
E1B702	TGFBR2	E1B702	ENSBTAG00000019832	0.261036674	-0.228839486	-0.48987616
A0A3Q1M7N8	SMIM26	A0A3Q1M7N8	ENSBTAG00000051249	0.465440719	0.081654154	-0.383786565
Q3SZX4	CA3	bta:513212; Q3SZX4	ENSBTAG00000015214	0.223747701	0.99524647	0.771498769
F1MUT4	F11	F1MUT4	ENSBTAG00000003572	0.0610687	0.151073736	0.090005036
Q7YS61	TRDMT1	bta:353353; Q58D29; Q7YS61		-0.149587113	-0.305381786	-0.155794673
A0AAA9TCW8	SNAP47	A0AAA9TCW8	ENSBTAG00000004188	-0.096707413	0.125927172	0.222634586
A0AAA9T8L1				0.28326889	0.086983302	-0.196285588
A0AAA9SS54	BTBD2	A0AAA9SS54	ENSBTAG00000016108	0.292091803	0.379469179	0.087377376
Q1RMU9	FIG4	bta:534118; Q1RMU9		0.129946173	0.167343161	0.037396989
Q0V8L2	GGT7	A6QR42; bta:615929; Q0V8L2	ENSBTAG00000013301	-0.079617168	0.021963431	0.1015806
A0AAA9TSY3	RBIS	A0AAA9TSY3	ENSBTAG00000032227	-0.556069475	-0.804604311	-0.248534836
E1BG64	FEZ2	bta:100299092; E1BG64	ENSBTAG00000009021	-0.294138066	-0.485679069	-0.191541003
A0A3Q1MDQ9	NKPD1	A0A3Q1MDQ9	ENSBTAG00000020074	0.104456623	0.366957783	0.262501161
F1MMI9	TRPT1	F1MMI9	ENSBTAG00000016555	-0.322275272	-0.161445153	0.16083012
Q1RMK0	RNF115	bta:614061; F6RQU6; Q1RMK0	ENSBTAG00000008131	0.186119438	-0.154562617	-0.340682055
F1MSV4	SGK3	F1MSV4	ENSBTAG00000013284	0.150992095	0.100040907	-0.050951187
A0A3Q1M1J0	GFOD1	A0A3Q1M1J0	ENSBTAG00000049619	-0.192086651	0.115176437	0.307263088
A0AAA9SBD6	ATPSCKMT	A0AAA9SBD6	ENSBTAG00000017885	0.060561246	0.135802618	0.075241372
F1MWF6	ACAP3	F1MWF6	ENSBTAG00000009856	-0.452666545	-0.481035058	-0.028368513
A0A452DI36	ALAS1	A0A452DI36	ENSBTAG00000004118	-0.041619089	-0.075440756	-0.033821667
Q2YDK5	POLR1D	Q2YDK5		-0.030405657	-0.208419479	-0.178013821
A0AAA9T6B4	FAIM	A0AAA9T6B4	ENSBTAG00000023426	-0.364672716	-0.086794463	0.277878253
A0A3Q1LRP7	NRF1	A0A3Q1LRP7	ENSBTAG00000014273	-0.213403638	-0.031250934	0.182152704
A0AAA9RU81				-0.134697729	-0.211348517	-0.076650788
A0AAA9TMX6	ZBTB33	A0AAA9TMX6	ENSBTAG00000017184	0.066267792	-0.227053799	-0.293321591
A0A3Q1LVU6	SLC12A6	A0A3Q1LVU6	ENSBTAG00000016236	1.214124805	0.913288367	-0.300836439
A0AAA9T713	STARD10	A0AAA9T713	ENSBTAG00000015182	0.156725504	0.371520777	0.214795273
A0AAA9SM03	KDM4C	A0AAA9SM03	ENSBTAG00000043987	-0.298752818	-0.512774301	-0.214021483
A0AAF6YZY8	CDC26	A0AAF6YZY8	ENSBTAG00000014430	0.119024103	-0.304006187	-0.42303029
Q5E9U1	P2RX4	bta:338036; Q5E9U1	ENSBTAG00000010812	0.177522149	0.142066611	-0.035455538
A0JNC4	ELOVL7	A0JNC4; bta:614096	ENSBTAG00000007704	-0.266280065	-0.08587166	0.180408405
F1MKY1	AGGF1	bta:511650; F1MKY1	ENSBTAG00000034823	-0.016766851	-0.050896712	-0.034129861
Q4TVR5	DSTYK	bta:534684; Q4TVR5	ENSBTAG00000016618	0.305249354	0.056902391	-0.248346963
A0AAA9T9D5	TICAM1	A0AAA9T9D5	ENSBTAG00000019966	0.103458093	0.010536161	-0.092921932
A0AAA9TSN9	DCBLD1	A0AAA9TSN9	ENSBTAG00000047338	-0.144457526	0.192163243	0.336620768
A0A0F7RPX0	MIF	A0A0F7RPX0; bta:280858		0.050108848	0.3332809	0.283172051
A0AAA9TBL0				-0.103904607	-0.140271727	-0.03636712

Accession	Gene_Symbol	Gene_ID	Ensembl_Gene ID	log2(ratio(CM12H/CM0H))	log2(ratio(CM12R/CM0H))	log2(ratio(CM12H/CM12R))
F1MLZ2	SLC20A1	bta:514255; F1MLZ2	ENSBTAG00000007895	-0.613766529	-0.957029961	-0.343263432
E1BNA6	DOCK8	E1BNA6	ENSBTAG00000002190	-0.298805537	-0.049925225	0.248880312
A0A3Q1LRZ5	PLEKHA7	A0A3Q1LRZ5	ENSBTAG00000006974	-0.118953127	-0.139201811	-0.020248684
E1BBH2	TRAF3	bta:506182; E1BBH2	ENSBTAG00000069778	-0.043106573	0.242064632	0.285171205
F6S165	RTN2	F6S165	ENSBTAG00000008185	-0.17421236	0.14255403	0.31676639
G5E6P9	TADA2B	bta:515581; G5E6P9	ENSBTAG00000010185; ENSBTAG00000066252	-0.255838904	-0.080796143	0.175042761
A0A452DJB5	SLC35C1	A0A452DJB5	ENSBTAG00000003199	-0.006440614	0.111269302	0.117709916
A0AAA9T467	COX14	A0AAA9T467	ENSBTAG00000000808	0.204682167	0.160710915	-0.043971252
A0AAA9T5D6	SMARCA1	A0AAA9T5D6	ENSBTAG00000002292	-0.585535567	-0.200030485	0.385505083
A0A3Q1M082	ATP8B2	A0A3Q1M082	ENSBTAG00000005161	0.073117929	0.091177901	0.018059972
O97827	ADGRL3	bta:282651; O97818; O97819; O97820; O97821; O97822; O97823; O97824; O97825; O97826; O97827; O97828; O97829	ENSBTAG00000013918	0.140572917	0.264219858	0.123646941
E1BI54	HOXC8	bta:100295249; E1BI54	ENSBTAG00000012149	0.320960601	-0.260779439	-0.58174004
E1BK01	PRSS12	E1BK01	ENSBTAG00000020705	0.128691223	-0.202209213	-0.330900436
A0AAA9S1N2	CAMKK2	A0AAA9S1N2	ENSBTAG00000010815	0.08944478	-0.122147109	-0.21159189
A0AAA9SZN1	IRF3	A0AAA9SZN1	ENSBTAG00000006633	0.124614012	-0.258276052	-0.382890064
A5D7G9	SGK196	A5D7G9; bta:514490		-0.575255037	-0.490707646	0.084547391
Q5EA04	TXNL4B	bta:510719; Q5EA04	ENSBTAG00000006353	0.271174794	-0.005949266	-0.277124061
A0AAF6DM00	NKAPD1	A0AAF6DM00	ENSBTAG00000001999	0.233374432	0.194321656	-0.039052777
A0A2H4GTG0				-0.043157582	0.39175878	0.434916362
E1BCU9	STK11	E1BCU9	ENSBTAG00000011639	0.047845049	-0.001521028	-0.049366077
A0AAA9TIB9	LRRC20	A0AAA9TIB9	ENSBTAG00000005666	-0.11712507	0.211711394	0.328836464
A0AAA9RW64	GDE1	A0AAA9RW64	ENSBTAG00000002101	-0.261516578	-0.255461186	0.006055392
A0A3Q1LLJ8	SPMIP6	A0A3Q1LLJ8	ENSBTAG00000008376	-0.585613096	-0.433805238	0.151807858
A5D7N7	TMCO3	A5D7N7; bta:509611	ENSBTAG00000015704	-0.057715498	-0.079968305	-0.022252807
G3N3X2	SRBD1	G3N3X2	ENSBTAG00000007508	-0.681935694	-0.474109839	0.207825855
E1B8K5	COQ8B	E1B8K5	ENSBTAG00000019462	-0.166176412	-0.095932951	0.070243461
A0AAA9RWM9	DHRS3	A0AAA9RWM9	ENSBTAG00000024493	0.156401557	0.120382335	-0.036019223
A0AAA9T6U9	USP53	A0AAA9T6U9	ENSBTAG00000010350	-0.028344626	0.124946014	0.153290639
Q2T9W7	MOSPD1	bta:523578; Q2T9W7	ENSBTAG00000013922	-0.005959096	-0.154251415	-0.14829232
A0AAF6DMI7	BCL7C	A0AAF6DMI7	ENSBTAG00000008412	0.226628759	0.086451101	-0.140177658
A0A172W434	ND4	A0A172W434		-0.031201955	0.02659421	0.057796165
F1N3E8	POLR3K	F1N3E8	ENSBTAG00000019803	0.054902678	-0.131374529	-0.186277207
A0AAF7AFF3	BCHE	A0AAF7AFF3	ENSBTAG00000011139	-0.059118216	-0.071804429	-0.012686213
Q2HJC0	NGRN	bta:508115; Q2HJC0		-0.293380276	-0.325741148	-0.032360872
A0AAA9STI8	FBXO5	A0AAA9STI8	ENSBTAG00000021193	0.281083043	-0.350941495	-0.632024538
Q0P563	FUOM	bta:613571; Q0P563		-0.224560258	-0.191201661	0.033358597
A0AAA9S762	ATP5MJ	A0AAA9S762	ENSBTAG00000026886	0.004069664	-0.117403001	-0.121472666
Q3MHJ7	EEPD1	bta:511767; Q3MHJ7; Q6Q139	ENSBTAG00000019065	0.158730415	0.310593025	0.15186261
A0A3Q1NDB9	BBS7	A0A3Q1NDB9	ENSBTAG00000004945	0.392891858	-0.171865412	-0.56475727
A0AAA9T8G7				0.23199559	0.182119683	-0.049875907
F1MZ30	COL10A1	F1MZ30	ENSBTAG00000003078	0.226645749	0.196939242	-0.029706507
E1BIN1	STIL	E1BIN1	ENSBTAG00000017844	0.391757479	0.254893873	-0.136863606
Q3SZR9	TSPAN3	bta:616881; Q3SZR9	ENSBTAG00000002415	0.1489416	0.241826128	0.092884528
F1MGJ6	NRBP2	bta:504664; F1MGJ6	ENSBTAG00000008079	-0.320239745	-0.256292828	0.063946917
F1MVD6	CCDC88B	F1MVD6	ENSBTAG00000040190	0.452014147	0.020203571	-0.431810576
A0AAA9S2H2	TSPAN18	A0AAA9S2H2	ENSBTAG00000021484	-0.09178484	-0.14979616	-0.058011321
Q5E985	HYAL1	bta:515397; Q3T0A8; Q5E985; Q5E9Z4		-0.073921571	0.126406342	0.200327913
Q2KJE0	TAX1BP1	bta:535589; Q2KJE0	ENSBTAG00000019020	0.637304398	0.01398988	-0.623314518
F1N1H5	MCM10	F1N1H5	ENSBTAG00000016406	0.335019092	0.72141078	0.386391687
A0AAA9SB31	ARL5B	A0AAA9SB31	ENSBTAG00000043964	-0.061737458	0.001548787	0.063286245
F6QUB2	RNFT1	F6QUB2	ENSBTAG00000009747	-0.05652518	-0.118519467	-0.061994287
E1BA57	DEPDC1	E1BA57	ENSBTAG00000001343	0.351804379	0.381748181	0.029943802
A0A452DJ16	FAM131B	A0A452DJ16	ENSBTAG00000009418	0.041388238	-0.013498999	-0.054887238
A0AAA9S0B6	CEP104	A0AAA9S0B6	ENSBTAG00000020014	0.411222526	0.104494168	-0.306728358
E1BND5	NCOA6	bta:508899; E1BND5	ENSBTAG00000007930	-0.225767148	-0.138582578	0.08718457
A0AAA9TS17	TNFRSF12A	A0AAA9TS17	ENSBTAG00000012082	-0.397978758	-0.758895318	-0.360916561
A0AAA9T627	NAA38	A0AAA9T627	ENSBTAG00000014695	-0.376401263	-0.085649064	0.290752199
F1MCL6	NADK	F1MCL6	ENSBTAG00000000212	0.181470002	-0.07440239	-0.255872393
A0AAA9TZN6	CSDC2	A0AAA9TZN6	ENSBTAG00000004004	0.037859544	0.160815757	0.122956213
A0AAA9SAI9	ARHGAP24	A0AAA9SAI9	ENSBTAG00000003959	-0.200007035	-0.066624732	0.133382303
A0AAA9T0W8	PIAS4	A0AAA9T0W8	ENSBTAG00000000753	-0.273306693	-0.020898893	0.2524078
A0AAA9RVU8	EEF1AKMT1	A0AAA9RVU8	ENSBTAG00000008296	-0.192038436	-0.153734548	0.038303888
F1MB67	SLC27A1	F1MB67	ENSBTAG00000016775	-0.195892686	-0.159930473	0.035962213
F1MFC9	TCTN1	F1MFC9	ENSBTAG00000009208	-0.827845448	-0.729936615	0.097908832
A0AAA9T921	TSEN2	A0AAA9T921	ENSBTAG00000037980	-0.469485283	-0.142740172	0.326745111
E1BPV8	SLCO4A1	E1BPV8	ENSBTAG00000016388	-0.276859661	-0.09513424	0.181725422
A0A0S1S2N1	ND2	A0A0S1S2N1		-0.035489085	0.01875396	0.054243045
A0AAA9TPS8	CCDC28B	A0AAA9TPS8	ENSBTAG00000015104	-0.0660034	-0.301145023	-0.235141623
A0AAA9SZH6	TEX30	A0AAA9SZH6	ENSBTAG00000025221	-0.526638239	-0.1613783	0.365259938
A0A3Q1MS15	FNIP1	A0A3Q1MS15	ENSBTAG00000025443	0.165503528	-0.142444265	-0.307947793
F1MC61	MAP3K11	F1MC61	ENSBTAG00000004114	0.005924834	0.169265182	0.163340348
A0AAF6Z6T7	PTOV1	A0AAF6Z6T7	ENSBTAG00000030578	0.277824812	-0.762177538	-1.04000235
A0A3Q1M8G4				0.288485033	0.090197809	-0.198287225
A5PJK0	SERPINB10	A5PJK0; bta:510205		-0.078923105	0.011997533	0.090920638
A0A3Q1MJP5	C25H7orf50	A0A3Q1MJP5; bta:522840	ENSBTAG00000051685	-0.249451434	-0.598428568	-0.348977135
F6PVN0	MTRES1	F6PVN0	ENSBTAG00000021398	0.184005035	-0.028748938	-0.212753973
A0A3Q1LP90	BMPR2	A0A3Q1LP90; bta:407127	ENSBTAG00000006420	0.005581032	-0.092364018	-0.09794505
A5D7L6	MRAS	A5D7L6; bta:540803	ENSBTAG00000001497	0.438203484	0.341856088	-0.096347396
A0AAA9S2L3	SLC35E1	A0AAA9S2L3; bta:533774	ENSBTAG00000005295	-0.037117594	-0.117287943	-0.080170349
Q32L09	SHFL	bta:539087; Q32L09	ENSBTAG00000015636	0.265653376	0.331902323	0.066248947
F1MWY6	EI24	F1MWY6	ENSBTAG00000007202	0.172408126	0.147382433	-0.025025693
Q5E9F6	VPS72	bta:534883; Q2TBU7; Q5E9F6	ENSBTAG00000018074	-0.093383447	-0.045936284	0.047447162
A0A3Q1LXS5	GRAMD1B	A0A3Q1LXS5	ENSBTAG00000001410	-0.114056343	0.218906807	0.33296315
A0AAA9RXT8	ADNP2	A0AAA9RXT8	ENSBTAG00000005916	-0.062561338	0.091243998	0.153805336
A0AAA9TSX0	RCCD1	A0AAA9TSX0	ENSBTAG00000018642	0.226037288	0.311944006	0.085906718
A0AAA9TRN0	ZNF668	A0AAA9TRN0	ENSBTAG00000006650	-0.103241106	-0.21290315	-0.109662044

Accession	Gene_Symbol	Gene_ID	Ensembl_Gene ID	log2(ratio(CM12H/CM0H))	log2(ratio(CM12R/CM0H))	log2(ratio(CM12H/CM12R))
Q32LB3	CXXC5	bta:538485; Q32LB3	ENSBTAG00000003986	0.119421165	-0.440939363	-0.560360527
A0AAA9SYW5	NEK3	A0AAA9SYW5	ENSBTAG00000020612	0.074000581	0.263343037	0.189342455
A0A3Q1M6J2	TSPAN15	A0A3Q1M6J2	ENSBTAG00000000224	0.210145361	0.250172255	0.040026894
A0AAA9T328	PHYHD1	A0AAA9T328	ENSBTAG00000038831	-0.45739994	-0.194737794	0.262662145
Q4U0T9	CSRP3	bta:540407; Q4U0T9	ENSBTAG00000011869	-0.147104294	-0.182167944	-0.03506365
A0AAA9TNQ2	RIT1	A0AAA9TNQ2	ENSBTAG00000000105	0.225645399	0.201775448	-0.023869952
A6QQS9	NBR1	A6QQS9; bta:515032	ENSBTAG00000001632	-0.210987658	-0.288280357	-0.077292699
A5PJW1	TAF10	A5PJW1; bta:514443	ENSBTAG00000000694	0.084846577	0.101509265	0.016662688
A0A3Q1M028	TSPO	A0A3Q1M028	ENSBTAG00000018073	0.022961148	0.045562577	0.022601429
A0A3Q1MQA6	PHAF1	A0A3Q1MQA6	ENSBTAG00000005058	0.185692317	0.196108933	0.010416616
G3X6S6	NHS	G3X6S6	ENSBTAG00000039195	-0.030711003	0.125200557	0.15591156
Q58CR1	MTFR2	bta:534254; Q58CR1	ENSBTAG00000007799	0.228094781	0.054659385	-0.173435396
F1MV04	ZFP91	F1MV04	ENSBTAG00000024657	0.0775366	0.002947284	-0.074589316
E1B9V4	TTF1	E1B9V4	ENSBTAG00000018710	0.023387666	0.004414165	-0.018973501
A0AAA9TUZ8	STX11	A0AAA9TUZ8	ENSBTAG00000011236	0.218894977	0.13044695	-0.088448027
Q2HJ44	TENT2	bta:533862; Q1JPF9; Q2HJ44	ENSBTAG00000006751	-1.098836015	0.007329542	1.106165557
A0AAA9TZI7	CDSN	A0AAA9TZI7	ENSBTAG00000021721	-0.384972626	-0.493451728	-0.108479101
F1MQB7	PSTK	F1MQB7	ENSBTAG00000014028	-0.092895212	-0.30079663	-0.207901419
A0AAF7AKA5	PPT2	A0AAF7AKA5	ENSBTAG00000004436	0.128924539	0.237989276	0.109064738
E1BCI6	RECK	bta:517232; E1BCI6	ENSBTAG00000000076	-0.104750811	0.099204362	0.203955173
A0A0B5H0C3	ND1	A0A0B5H0C3		-0.07018383	-0.047351385	0.022832446
F1MCM3	BLTP3A	bta:534225; F1MCM3	ENSBTAG00000009267	-0.25650214	-0.500942354	-0.244440214
A0AAA9SMJ0	FGD6	A0AAA9SMJ0	ENSBTAG00000014030	0.242876662	0.001429119	-0.241447544
A0AAA9SB64	MOCS1	A0AAA9SB64	ENSBTAG00000010449	-0.279667227	-0.170079276	0.109587951
F1N5V1	USP37	bta:407168; F1N5V1	ENSBTAG00000016572	0.543289652	0	-0.543289652
Q05KJ0	BCL2L1	bta:282152; Q05KJ0	ENSBTAG00000006526	0.004159623	-0.282887679	-0.287047303
A5PJU7	RMI2	A5PJU7; bta:615553		-0.171045541	-0.036296129	0.134749412
Q2KIT0				0.016210227	0.202460519	0.186250292
A0AAA9SY52	FAAH	A0AAA9SY52	ENSBTAG00000007507	-0.277984747	-0.108059746	0.169925001
A0A3Q1M6W9	ZNF385A	A0A3Q1M6W9	ENSBTAG00000015604	0.168151757	0.056862337	-0.11128942
Q3SYZ0	BAD	bta:615013; Q3SYZ0		0.232102546	0.345172692	0.113070146
G3N319	ATXN1L	bta:521025; G3N319	ENSBTAG00000046255	0.09168069	0.150206767	0.058526077
A0AAA9T3V1				-0.153445068	0.067572704	0.221017771
A5PJD1	PHLDB3	A5PJD1; bta:533145		-0.085676825	-0.175296355	-0.08961953
A0AAA9S7U1	DNAL4	A0AAA9S7U1	ENSBTAG00000049822	0.081321724	0.144089223	0.062767498
F1MNI8	ARL13B	F1MNI8	ENSBTAG00000005155	-0.06892689	-0.06892689	0
E1BCF7	DLX2	bta:528490; E1BCF7	ENSBTAG00000005741	0.199164271	0.249027548	0.049863277
F1MF11	PLCH1	F1MF11	ENSBTAG00000008307	0.476607736	0.526471013	0.049863277
E1BBW7	RETREG3	bta:789163; E1BBW7	ENSBTAG00000032493	0.141123272	-0.146752402	-0.287875673
A0A3Q1N8B0	FADS1	A0A3Q1N8B0; bta:533107	ENSBTAG00000022294	-0.222203846	-0.243478997	-0.021275151
F1N3J7	SLC26A2	F1N3J7	ENSBTAG00000014615	-0.169460291	0.038523072	0.207983362
A0AAA9S9F1	YIPF4	A0AAA9S9F1	ENSBTAG00000033041	0.151264575	0.235203394	0.083938818
F1MJQ8	RNPC3	F1MJQ8	ENSBTAG00000019091	0.023970562	0.260514414	0.236543851
E1B7B3	CSGALNACT2	E1B7B3	ENSBTAG00000005165	-0.096597624	0.052863275	0.149460899
P05632	ATP5F1E	bta:617230; P05632; Q32PE5	ENSBTAG00000039208	-0.297144994	-0.074752573	0.222392421
Q2NL14	KANSL2	bta:540194; Q2NL14; Q58D11		0.085702486	0.039335894	-0.046366591
A0AAA9SWH7	DGKH	A0AAA9SWH7	ENSBTAG00000013879	-0.154241566	-0.298631475	-0.144389909
E1BI93	CBARP	E1BI93	ENSBTAG00000025233	0.15680408	0.31406987	0.157265791
F6QTR5	COQ10A	F6QTR5	ENSBTAG00000004367	-0.357025511	-0.074478216	0.282547295
A5D7P9	LYVE-1	A5D7P9; bta:404179		0.225232373	0.08061846	-0.144613913
Q3B7N6	ERCC5	bta:509602; Q3B7N6	ENSBTAG00000014043	-0.290374926	-0.224998917	0.065376009
G3X746	CABIN1	G3X746	ENSBTAG00000024708	-0.247428972	-0.00666373	0.240765242
F1MX93	MYO5C	F1MX93	ENSBTAG00000003763	0.532772352	0.638663521	0.105891169
A0AAF6Z840	COQ6	A0AAF6Z840	ENSBTAG00000020331	-0.201180662	-0.187842862	0.0133378
A0AAA9TVB0	PRR5L	A0AAA9TVB0	ENSBTAG00000021029	-0.286074647	0.128552937	0.414627585
E1BAA5	TRMT44	E1BAA5	ENSBTAG00000004797	0.089667337	-0.088948181	-0.178615518
A0AAF6Z8G7	USP33	A0AAF6Z8G7	ENSBTAG00000020761	0.029443778	-0.128607123	-0.158050901
E1B8U2	THUMPD2	E1B8U2	ENSBTAG00000004722	-0.806089955	-0.575879396	0.230210559
A5PKB4	TIMP3	A5PKB4; F6RQF5	ENSBTAG00000020638	-0.924074664	-0.82976931	0.094305353
Q32LJ0	SVBP	bta:614073; Q32LJ0	ENSBTAG00000017388	-0.094122177	-0.547487795	-0.453365618
A0A3Q1ME34	REXO1	A0A3Q1ME34	ENSBTAG00000020232	-0.063586683	-0.010806755	0.052779928
A0A452DIR3	IFT57	A0A452DIR3	ENSBTAG00000005787	0.104857314	-0.055432391	-0.160289705
E1BB91	COL6A3	E1BB91	ENSBTAG00000030190	-0.070389328	-0.220338763	-0.149949435
A0AAA9S2N1	ZER1	A0AAA9S2N1	ENSBTAG00000010208	-0.121501005	-0.063815098	0.057685907
A0A3Q1MQ12	SDC2	A0A3Q1MQ12	ENSBTAG00000014357	0.188924014	0.249409143	0.060485129
A0A3Q1LXV0	TSPAN9	A0A3Q1LXV0	ENSBTAG00000049163	0	0.072986439	0.072986439
A0AAA9TEI4	MREG	A0AAA9TEI4; bta:613445	ENSBTAG00000013215	-0.148945063	-0.114642566	0.034302497
A0AAA9TJ11	NQO1	A0AAA9TJ11	ENSBTAG00000020632	0.266601021	0.230531766	-0.036069255
A0AAF6Z4S9	C16H1orf116	A0AAF6Z4S9	ENSBTAG00000018143	-0.057018421	0.14468568	0.201704101
E1BCU1	UBE3B	bta:512750; E1BCU1	ENSBTAG00000002698	-0.11501666	-0.069353281	0.045663379
A0AAA9T2M8	RFX7	A0AAA9T2M8; bta:541058	ENSBTAG00000000565	-0.247756101	-0.145617458	0.102138642
F1MVH6	B3GALT6	bta:522406; F1MVH6	ENSBTAG00000037523	0.193453537	-0.049355841	-0.242809378
A0A3Q1LF99	ANKRD11	A0A3Q1LF99; bta:532856	ENSBTAG00000016006	-0.259710769	-0.484001474	-0.224290705
E1BAS6	SETX	E1BAS6	ENSBTAG00000024822	-0.059515285	-0.109838599	-0.050323314
A0AAA9RXN7	CEP76	A0AAA9RXN7	ENSBTAG00000010547	-0.032936404	-0.169571096	-0.136634692
A0AAA9T1Y9	SAT2	A0AAA9T1Y9	ENSBTAG00000004222	0.218882437	0.227733355	0.008850918
G5E699	TMEM131L	G5E699	ENSBTAG00000016625	0.058459731	-0.447952122	-0.506411852
A0AAA9TWJ8	TMTC2	A0AAA9TWJ8	ENSBTAG00000002888	-0.673301975	-0.624447588	0.048854387
A6QNU8	TPGS2	A6QNU8; bta:540456	ENSBTAG00000021583	0.836501268	-0.490714659	-1.327215926
F1MVI6	TCF3	F1MVI6	ENSBTAG00000008695	0.344180902	-0.068895081	-0.413075983
A1A4K7	FAM210B	A1A4K7; bta:516545	ENSBTAG00000013007	0.068912921	0.272187141	0.20327422
E1BHM7	RNF216	E1BHM7	ENSBTAG00000017175	-0.086804828	-0.411531556	-0.324726728
A2VEA7	DAPL1	A2VEA7; bta:574085; Q4PP00	ENSBTAG00000032481	-0.051428239	0.03329444	0.084722679
E1BHJ3	FRMD4A	bta:510714; E1BHJ3	ENSBTAG00000008279	0.072802535	-0.037103995	-0.109906529
A0A3Q1MVB0	CDC6	A0A3Q1MVB0	ENSBTAG00000010384	0.233605398	-0.306962984	-0.540568381
E1BKB4	TTLL4	E1BKB4	ENSBTAG00000018415	-0.103970397	-0.222580284	-0.118609887
A0AAA9TK30	TTC28	A0AAA9TK30	ENSBTAG00000012193	-0.206530892	-0.351615847	-0.145084956
A0AAF7A190	HYAL2	A0AAF7A190	ENSBTAG00000000484	-0.467184701	-0.664961823	-0.197777122
F1MI51	SIMC1	bta:100336733; F1MI51	ENSBTAG00000034998	-0.129930304	-0.143209642	-0.013279338
A0AAA9TAS5	NOL12	A0AAA9TAS5	ENSBTAG00000060113	-0.134075754	0.265671875	0.399747628
E1BGL2	GARRE1	E1BGL2	ENSBTAG00000013175	0.019546975	-0.133120037	-0.152667012

Accession	Gene_Symbol	Gene_ID	Ensembl_Gene ID	log2(ratio(CM12H/CM0H))	log2(ratio(CM12R/CM0H))	log2(ratio(CM12H/CM12R))
A0A3Q1LPL5	PHF11	A0A3Q1LPL5	ENSBTAG00000013159	0	-0.791715798	-0.791715798
F1MFH5	KLHL42	bta:781458; F1MFH5	ENSBTAG00000006372	-0.03124068	-0.55920562	-0.52796494
A0AAA9RZ85	KDELR3	A0AAA9RZ85	ENSBTAG00000009886	-0.025872525	-0.002701677	0.023170848
A0AAA9S2A5	DCHS1	A0AAA9S2A5	ENSBTAG00000015405	-0.197695184	-0.057164875	0.140530309
Q2NKR1	TIMM10	bta:512818; Q2NKR1	ENSBTAG00000024701	-0.138222654	-0.042560462	0.095662192
A0A3Q1NMK6	HIRA	A0A3Q1NMK6	ENSBTAG00000019888	-0.008267616	0.149940627	0.158208243
A0AAA9TTT0	TPM1	A0AAA9TTT0	ENSBTAG00000005373	-0.941583314	-0.79105311	0.150530204
A0A3Q1MG28	RESTB	A0A3Q1MG28	ENSBTAG00000011789	-0.11044077	-0.137798886	-0.027358116
Q0VCP2	PEX11A	bta:515608; Q0VCP2	ENSBTAG00000006140	-0.146880661	-0.076781234	0.070099426
A0A3Q1MCX7	MPPE1	A0A3Q1MCX7; bta:506830	ENSBTAG00000002128	0.005053228	0.065963262	0.060910035
F1MTW9	TRIM35	bta:518310; F1MTW9	ENSBTAG00000009958	-0.783727185	-0.83522961	-0.051502425
A0AAA9T1M1	TRIM44	A0AAA9T1M1	ENSBTAG00000037389	0.613594654	0.166708289	-0.446886365
E1BBM5	RNF167	bta:616030; E1BBM5	ENSBTAG00000004913	-0.015415052	0.158262084	0.173677136
Q3T104	SEC61G	bta:615778; Q3T104	ENSBTAG00000014971	-0.052861814	-0.005333444	0.04752837
Q08D85	PXDC1	bta:613986; Q08D85	ENSBTAG00000026919	-0.072396696	-0.255671251	-0.183274555
Q3B7L5	FLCN	bta:526652; Q3B7L5	ENSBTAG00000008010	-0.080602652	-0.121909626	-0.041306974
Q3T0Z8	SNRPF	bta:615240; Q3T0Z8	ENSBTAG00000016271	0.045134913	0.210114092	0.164979179
G5E568	UBN2	bta:540792; G5E568	ENSBTAG00000014021	-0.222632891	-0.569179503	-0.346546613
A0A3Q1MFM1	PLEKHM1	A0A3Q1MFM1	ENSBTAG00000020359	-0.028870058	0.176762036	0.205632095
A0A3Q1MBZ8	KDM2B	A0A3Q1MBZ8	ENSBTAG00000002328	0.09292481	0.040765034	-0.052159776
A7Z056	USP20	A7Z056; bta:505839	ENSBTAG00000020311	0.179409251	0.186662635	0.007253384
Q58DI5	PLPP6	A6QLK7; bta:541021; Q58DI5	ENSBTAG00000011050	-0.184706045	-0.522556135	-0.33785009
Q3SZI3	C7H5orf15	bta:514781; Q3SZI3	ENSBTAG00000013112	0.126782678	0.066741551	-0.060041127
F1MJS7	WDR24	A0A8J8YNK6; F1MJS7; M5FJX7	ENSBTAG00000019006	0.121412424	0.134958955	0.013546532
A0AAF6DLL3	CCP110	A0AAF6DLL3; bta:538197	ENSBTAG00000003960	0.487363006	-0.013626501	-0.500989507
E1B949	FAT4	bta:781683; E1B949	ENSBTAG00000003345	-0.09360475	-0.170110736	-0.076505986
A0A3Q1LLQ6	TCF4	A0A3Q1LLQ6	ENSBTAG00000016462	0.407547632	-0.763504211	-1.171051843
A2VDS3	TLCD3A	A2VDS3; bta:508425	ENSBTAG00000046273	-0.253419317	-0.226234488	0.027184829
A0AAA9S548	PCMTD2	A0AAA9S548	ENSBTAG00000031715	-0.546608693	-0.179962678	0.366646015
A0A7U3JWD0	FGF2B	A0A7U3JWD0; bta:281160		-0.167619061	0.032597298	0.200216359
A0A3Q1LH17	DROSHA	A0A3Q1LH17	ENSBTAG00000017551	0.093911868	0.035241006	-0.058670862
F1N5D1	CA5B	bta:514494; F1N5D1	ENSBTAG00000020418	-0.410815161	-0.318760184	0.092054978
E1BD39	ORAI2	bta:511233; E1BD39	ENSBTAG00000012050	0.166778446	-0.10408916	-0.270867606
A0A3Q1ME24	RNF5	A0A3Q1ME24	ENSBTAG00000025413	-0.11284147	-0.025858168	0.086983302
E1BJZ9	TRAPPC14	E1BJZ9	ENSBTAG00000015011	0.011997533	0.041561991	0.029564458
A0AAA9SUI3	GTF2A2	A0AAA9SUI3	ENSBTAG00000010298	-0.120528086	-0.081592424	0.038935663
A0AAA9SGC7	ZBTB7B	A0AAA9SGC7	ENSBTAG00000021512	-0.04935253	-0.071674198	-0.022321668
E1BIS6	SYNM	E1BIS6	ENSBTAG00000011752	0.063669528	0.169756787	0.106087258
A0AAA9U0M1	SLC25A30	A0AAA9U0M1	ENSBTAG00000016529	-0.45205069	-0.235239301	0.216811389
A0AAA9U1L4	POLR2F	A0AAA9U1L4	ENSBTAG00000004820	0.171157547	0.332983283	0.161825736
A0AAF7A6Y1	ZDHHC21	A0AAF7A6Y1	ENSBTAG00000006197	0.139875403	0.024274746	-0.115600657
Q29S16	ABITRAM	bta:507081; Q29S16		-0.019011969	-0.00290866	0.016103308
A0AAA9SBK6	TMEM50A	A0AAA9SBK6	ENSBTAG00000001296	-0.058504845	0.136048216	0.194553061
G3N2G3	GPRIN3	bta:517995; G3N2G3	ENSBTAG00000045966	0.359149078	0.424366738	0.065217659
A0AAA9U0C4	IFT172	A0AAA9U0C4; bta:100848219	ENSBTAG00000018157	-0.079007874	-0.071067871	0.007940004
A0AAA9TUC8	PAFAH2	A0AAA9TUC8	ENSBTAG00000005105	0.099219605	0.201702563	0.102482958
A0AAA9SNV1	RAD54L	A0AAA9SNV1	ENSBTAG00000019901	-0.254409159	-0.437546677	-0.183137518
Q2TBH1	CENPM	bta:615953; Q2TBH1		-0.004427712	-0.131034028	-0.126606316
F1MVZ5	SIDT2	F1MVZ5	ENSBTAG00000007192	-0.058223221	0.255454769	0.31367799
F1MX90	RUNDC1	bta:539525; F1MX90	ENSBTAG00000015140	0.438121112	0.708768702	0.27064759
A0A3Q1LT07	GATC	A0A3Q1LT07	ENSBTAG00000012791	-0.303203453	0.343058262	0.646261715
F1MLZ4	LYST	F1MLZ4	ENSBTAG00000016804	0.454654478	0.027604461	-0.427050017
A0AAA9SE34	PRR5	A0AAA9SE34	ENSBTAG00000039784	-0.359241253	-0.391090119	-0.031848866
E1BQ25	ING3	bta:513000; E1BQ25	ENSBTAG00000016332	0.239928157	-0.094232561	-0.334160718
A0AAA9SLC2	NSUN6	A0AAA9SLC2	ENSBTAG00000022003	-0.011777168	-0.059871456	-0.048094288
F6R7S9	PFKFB3	F6R7S9	ENSBTAG00000008401	0.132247798	0.011495639	-0.120752159
A0A452DKA0	C16H1orf174	A0A452DKA0	ENSBTAG00000024520	0.129923989	-0.13552497	-0.265448959
E1BLX3	ORAI3	bta:515971; E1BLX3	ENSBTAG00000002344	0.130553554	0.439066559	0.308513005
E1BIA9	TAF3	bta:506674; E1BIA9	ENSBTAG00000002836	0.082019765	0.127917475	0.04589771
A0A3Q1MDW1	ROGDI	A0A3Q1MDW1	ENSBTAG00000016331	-0.161410547	0.417045426	0.578455973
A0AAA9TGS3	ASB6	A0AAA9TGS3	ENSBTAG00000008973	0.536263682	0.019732065	-0.516531617
A0AAA9TAB3	PCP4L1	A0AAA9TAB3	ENSBTAG00000040512	-0.034985358	-0.270265975	-0.235280617
F6R1G6	TRMT13	F6R1G6	ENSBTAG00000015781	-0.799902618	-0.770251812	0.029650807
A0AAA9TBU0	TSEN34	A0AAA9TBU0	ENSBTAG00000006486	-0.907929378	-0.9536772	-0.045747822
A0AAA9TYC2	DEF8	A0AAA9TYC2	ENSBTAG00000039014	0.034995421	0.274794119	0.239798698
G3N1R6	CCDC9B	G3N1R6	ENSBTAG00000013774	0.026553419	-0.631486781	-0.6580402
A0A3Q1M739	PTPN6	A0A3Q1M739	ENSBTAG00000020294	-0.017036854	0.160140489	0.177177344
A0AAA9U010	HAPSTR1	A0AAA9U010; bta:538487	ENSBTAG00000013858	0.211640434	0.239854036	0.028213602
E1BBP4	DDR1	E1BBP4; F1MCM5	ENSBTAG00000010682	0.171494001	-0.167964018	-0.339458019
Q08DX3	TM7SF3	bta:510190; Q08DX3	ENSBTAG00000006985	0.068149592	0.05450053	-0.013649062
O19131	TNFRSF1A	bta:282527; O19131; Q2HJ72		0.071338782	-0.365802904	-0.437141686
E1BLR1	SPATA2	bta:529745; E1BLR1	ENSBTAG00000018063	-0.160778728	-0.005348273	0.155430455
A0AAF7AGC6	TAF12	A0AAF7AGC6	ENSBTAG00000026660	-0.040894006	-0.06573486	-0.024840855
A6QLU2	LOC786832	A6QLU2; bta:786832		0.059307094	0.05504926	-0.004257834
Q0VCR6	TMEM68	bta:534013; Q0VCR6	ENSBTAG00000005893	0.086375693	0.139182052	0.052806359
E1BCR9	PANK1	bta:535887; E1BCR9	ENSBTAG00000010442	0.281236267	0.212138327	-0.06909794
E1BAS5	RLF	E1BAS5	ENSBTAG00000019695	0.257017796	1.181127138	0.924109342
A0AAA9T5M6	ACD	A0AAA9T5M6	ENSBTAG00000018903	-0.260046052	-0.046988475	0.213057578
Q08E55	MBTPS1	bta:511682; F1MJR7; Q08E55	ENSBTAG00000010170	-0.04476987	-0.432959407	-0.388189537
A0AAA9SFY1	DOCK11	A0AAA9SFY1	ENSBTAG00000006439	-0.225915488	-0.030480606	0.195434881
A0AAA9ST97	BCL3	A0AAA9ST97	ENSBTAG00000009493	0.212993723	0.001424884	-0.211568839
G3MYM8	PRG4	G3MYM8	ENSBTAG00000011932	-0.08703489	-0.073760312	0.013274578
Q32PA5	UBE2C	bta:506962; Q32PA5		-0.870548749	-0.27961729	0.590931459
F1MK03	HNF1B	F1MK03	ENSBTAG00000004710	-0.074478216	-0.041419927	0.033058289
A6QQP9	ANKRD40	A6QQP9; bta:522429	ENSBTAG00000002267	-0.0756377	-0.20105828	-0.12542058
A0AAA9TPY0	TRIM13	A0AAA9TPY0	ENSBTAG00000008173	-0.217980861	-0.143655751	0.074325111
E1BF94	KBTBD6	E1BF94	ENSBTAG00000008656	0.138188239	0.396079342	0.257891103
A0A3Q1MQS1	NSMCE1	A0A3Q1MQS1	ENSBTAG00000002176	-0.058693994	-0.227995128	-0.169301133
A0A3Q1NIP0	TMEM184B	A0A3Q1NIP0	ENSBTAG00000009668	0.065793651	-0.237652157	-0.303445808
A0AAA9TCT6	TXNRD3	A0AAA9TCT6	ENSBTAG00000011795	-0.555697906	-0.303000813	0.252697093
Q58DD1	ZBTB43	bta:509462; Q58DD1	ENSBTAG00000003438	0.169925001	0.17732725	0.007402248

Accession	Gene_Symbol	Gene_ID	Ensembl_Gene ID	log2(ratio(CM12H/CM0H))	log2(ratio(CM12R/CM0H))	log2(ratio(CM12H/CM12R))
E1B7X8	BMT2	bta:538658; E1B7X8	ENSBTAG00000007608	0.363265354	0.093891565	-0.269373789
E1BLP2	SLC12A9	bta:617865; E1BLP2	ENSBTAG00000001131	0.138102277	0.98260295	0.844500673
A0A0S1S4M0	COX1	A0A0S1S4M0		0.24855196	0.21535191	-0.03320005
E1BNE0	FER	E1BNE0	ENSBTAG00000003051	0.127444017	0.034801883	-0.092642135
A0AAA9SJM1	ZBTB17	A0AAA9SJM1	ENSBTAG00000018730	-0.35614381	-0.155248089	0.200895721
A0AAA9T5E2	IL15RA	A0AAA9T5E2	ENSBTAG00000006078	-0.179536039	-0.022385167	0.157150873
A0A3Q1LU39	NFE2L1	A0A3Q1LU39	ENSBTAG00000013653	0.066815272	-0.341124596	-0.407939868
A0A3Q1LPM5	R3HCC1L	A0A3Q1LPM5	ENSBTAG00000021856	-0.070098198	-0.096315719	-0.026217521
A0A3Q1MPW8	ATXN7L3	A0A3Q1MPW8	ENSBTAG00000019662	-0.098162332	-0.103663559	-0.005501227
A0A3Q1LPT9	STEAP3	A0A3Q1LPT9	ENSBTAG00000007111	-0.208878636	-0.21649182	-0.007613184
A0AAA9TRH9	BORA	A0AAA9TRH9	ENSBTAG00000019886	-0.006272597	-0.041268018	-0.034995421
E1BM95	SCLT1	E1BM95	ENSBTAG00000013611	-0.162644477	-0.024416527	0.13822795
A0AAA9TJ68	RIN3	A0AAA9TJ68	ENSBTAG00000010416	-0.038508382	0	0.038508382
A0AAA9T145	SATB2	A0AAA9T145	ENSBTAG00000016334	-0.064301866	-0.16083466	-0.096532795
E1BHU9	ADCY5	bta:505740; E1BHU9	ENSBTAG00000018777	-0.156078949	-0.250130573	-0.094051624
A4IF98	RMI1	A4IF98; bta:614063	ENSBTAG00000021135	-0.194516521	-0.066989519	0.127527001
A0A3Q1M6K1	PTPRJ	A0A3Q1M6K1	ENSBTAG00000024715	-0.062625335	-0.083534325	-0.02090899
F1MLQ7	THSD7A	F1MLQ7	ENSBTAG00000007680	-0.287878477	0.123574685	0.411453162
A0AAA9SP93	IGFBP5	A0AAA9SP93	ENSBTAG00000059978	-0.094178463	-0.223896012	-0.129717549
E1BN39	SGTB	bta:540864; E1BN39	ENSBTAG00000016902	-0.318075769	-0.109489148	0.208586622
F1MKH0	LSM11	F1MKH0	ENSBTAG00000010363	-0.073927643	0.231274217	0.305201859
A0AAA9TG18	CCHCR1	A0AAA9TG18	ENSBTAG00000014434	0.111333163	0.291283883	0.17995072
P19879	OGN	A5D9E8; bta:280884; O18818; P19879	ENSBTAG00000011824	0.35460377	0.114499119	-0.240104651
F6QBA1	APBA3	F6QBA1	ENSBTAG00000008395	-0.028129373	0.208502034	0.236631407
A0AAA9T6Z2	SMYD4	A0AAA9T6Z2	ENSBTAG00000009708	-0.246258693	-0.080974079	0.165284615
Q2KJD7	UBLCP1	bta:508163; Q2KJD7	ENSBTAG00000009103	-0.629944946	-0.403641454	0.226303491
A0AAF6ZGR2	SPATA2L	A0AAF6ZGR2	ENSBTAG00000033334	0	-0.243332491	-0.243332491
F1MD09	ING4	F1MD09	ENSBTAG00000017064	0.282911428	0.04534095	-0.237570479
F6S002	MANBAL	F6S002	ENSBTAG00000000528	0.279610581	-0.021289541	-0.300900122
A0A3Q1LZT7	LCOR	A0A3Q1LZT7	ENSBTAG00000046239	0.059426278	-0.109079446	-0.168505724
G3N022	SPEG	G3N022	ENSBTAG00000046176	0.267083912	0.080711799	-0.186372112
F1ME70	SLC2A10	F1ME70	ENSBTAG00000002942	0.282303931	0.262076123	-0.020227808
A0AAA9T630	MRPL35	A0AAA9T630	ENSBTAG00000004823	-0.298707597	-0.402362472	-0.103654875
A0A3Q1M0V4	PACS2	A0A3Q1M0V4	ENSBTAG00000046346	0.09503779	0.008395921	-0.086641869
E1B958	ALMS1	E1B958	ENSBTAG00000011172	0.034114576	0.075653771	0.041539195
A0AAA9TPA7				0.361956362	-0.064946359	-0.426902722
A0AAA9S8K0	ANKRD26	A0AAA9S8K0	ENSBTAG00000008288	0.1740294	0.217559268	0.043529868
A0AAA9T749	UHRF1	A0AAA9T749	ENSBTAG00000002224	-0.836769651	-0.611982715	0.224786936
A0AAA9T539	BLTP1	A0AAA9T539	ENSBTAG00000048798	-0.169756787	-0.208108195	-0.038351409
A0AAA9TRI3	PLEKHA3	A0AAA9TRI3	ENSBTAG00000001098	0.188105918	0.051927792	-0.136178126
F1N397	LRWD1	F1N397	ENSBTAG00000014742	-0.248227606	-0.118781674	0.129445932
A0AAA9TDV7	PTPN5	A0AAA9TDV7	ENSBTAG00000020257	0.222173848	0.245590512	0.023416664
Q28120	QPCT	bta:281437; Q28120		-0.008461579	-0.399188671	-0.390727092
F1N2V0	CRB2	bta:786516; F1N2V0	ENSBTAG00000014391	-0.067114196	0.071083098	0.138197294
A5PJ69	SERPINA10	A5PJ69; bta:517172	ENSBTAG00000009894	-0.392947558	-0.300044977	0.092902581
Q3ZBS7	VTN	bta:507525; Q3ZBS7	ENSBTAG00000016151	-0.781932325	-0.871266686	-0.089334361
A0A3Q1MLX1	DTL	A0A3Q1MLX1	ENSBTAG00000018142	0.36240945	0.337456555	-0.024952895
A0AAA9RX49	IPMK	A0AAA9RX49	ENSBTAG00000011197	-0.070908376	-0.398601797	-0.327693421
A0AAA9TDS9	GEMIN7	A0AAA9TDS9	ENSBTAG00000010668	0.063086702	0.218101237	0.155014535
Q95M17	CHIA	bta:282645; Q3SZE1; Q95M17		-0.110492088	-0.025695615	0.084796473
F1MME0	TAF2	F1MME0	ENSBTAG00000015333	0.002911595	-0.01759406	-0.020505655
A0AAA9S5S1	PABIR2	A0AAA9S5S1	ENSBTAG00000001886	-0.03320005	-0.027370972	0.005829079
A0AAA9TJY8	CEP70	A0AAA9TJY8	ENSBTAG00000018538	0.048866001	-0.274327061	-0.323193062
E1BM79	ADCY9	A0A8J8YQJ3; E1BM79; M5FKA2	ENSBTAG00000016629	0.19426882	0.132374287	-0.061894534
A0A3Q1LWV5	ARHGAP26	A0A3Q1LWV5; bta:538219	ENSBTAG00000027151	0.134716903	0.143984783	0.00926788
A0AAA9RW02	HOGA1	A0AAA9RW02	ENSBTAG00000012721	-0.300819068	-0.043167859	0.257651209
A0A3Q1MEI9	SIPA1L2	A0A3Q1MEI9	ENSBTAG00000000942	-0.411930251	-0.176472089	0.235458161
A0AAA9U128	DNAJC14	A0AAA9U128	ENSBTAG00000020664	0.01267383	0.17055349	0.15787966
A0A3Q1LZ94	SYT11	A0A3Q1LZ94	ENSBTAG00000011209	0.160753356	-0.01793905	-0.178692406
A5PKH9	WDR51B	A5PKH9; bta:525136		-1.288231986	-1.129156441	0.159075545
E1BMD4	PHLPP2	E1BMD4	ENSBTAG00000007545	0.070791025	0.185031894	0.114240869
A0AAA9TJZ8	MFAP5	A0AAA9TJZ8	ENSBTAG00000000310	-0.046817222	0.020788473	0.067605694
F1N064	ETV3	F1N064	ENSBTAG00000008649	0.154697203	0.095635279	-0.059061924
A0A3Q1NLU3	NCOA3	A0A3Q1NLU3; bta:523707	ENSBTAG00000007489	0.163602714	0.017364643	-0.146238071
F6RQT4	ALKBH1	F6RQT4	ENSBTAG00000008133	0.023497181	-0.268731136	-0.292228317
E1B7I9	FRS2	bta:538624; E1B7I9	ENSBTAG00000019155	-0.044032768	-0.010154758	0.03387801
A0A3Q1M881	TBX3	A0A3Q1M881	ENSBTAG00000021706	0.335076799	0.131754928	-0.203321871
F1N7G6	ST3GAL5	F1N7G6	ENSBTAG00000011601	-0.028381801	0.221053493	0.249435294
A0A3Q1LKQ6	USP6NL	A0A3Q1LKQ6	ENSBTAG00000019488	0.04580369	0.283571337	0.237767647
A0A3Q1MS84	RASSF7	A0A3Q1MS84	ENSBTAG00000045834	0.327834736	0.466811149	0.138976413
E1BH64	GTPBP3	E1BH64	ENSBTAG00000004200	0.209227962	0.275489404	0.066261442
E1BIJ1	DEPDC7	E1BIJ1	ENSBTAG00000021809	0.388758174	-0.121275839	-0.510034013
F1MSD7	PLCB4	F1MSD7	ENSBTAG00000013116	-0.132086246	-0.161430207	-0.029343962
A0AAF6DMH2	MGAT4B	A0AAF6DMH2	ENSBTAG00000009738	-0.165657155	-0.165657155	0
E1BDG5	WNT5A	bta:530005; E1BDG5	ENSBTAG00000020221	-0.699304542	-0.589967914	0.109336629
Q08DM5				0.064196712	0.232173442	0.16797673
A0AAA9SMF0	RPS29	A0AAA9SMF0	ENSBTAG00000052337	-0.10766651	-0.154446269	-0.04677976
A5PK34	DEF6	A5PK34; bta:516457	ENSBTAG00000025622	-0.137771708	-0.120177648	0.01759406
A0AAA9SS09	FANCG	A0AAA9SS09	ENSBTAG00000015005	-0.344840621	-0.347923303	-0.003082683
Q3ZBF2	NOP10	bta:616675; Q3ZBF2	ENSBTAG00000025632	-0.234323109	-0.16901907	0.065304039
A0AAF6Z1H8	SRD5A1	A0AAF6Z1H8	ENSBTAG00000015478	-0.214987231	-0.353158052	-0.13817082
A0A3Q1MH98	DUSP28	A0A3Q1MH98	ENSBTAG00000049766	-0.070307637	0.210909853	0.281217491
A0AAA9TJC7	CEP290	A0AAA9TJC7	ENSBTAG00000018745	-0.487084992	-0.686011502	-0.198926511
A0JNE3	NR2C1	A0JNE3; bta:511407		-0.123951577	0.047025335	0.170976912
Q29RM1	SLC25A19	bta:504418; Q29RM1	ENSBTAG00000000202	-0.214340149	-0.087378731	0.126961418
Q2KJ64	ARG1	bta:513608; Q2KJ64	ENSBTAG00000012403	0.032960199	0.167228374	0.134268176
A0A452DI99	MED30	A0A452DI99	ENSBTAG00000026254	-0.150989812	-0.306695635	-0.155705822
Q3SWZ8	LMO4	bta:614212; Q3SWZ8	ENSBTAG00000000305	0.171368418	0.203973084	0.032604665
A0A3Q1MH87	USP31	A0A3Q1MH87	ENSBTAG00000011744	0.133855747	-0.100762977	-0.234618724
A0A3Q1LNP8	JRK	A0A3Q1LNP8; bta:517625	ENSBTAG00000055204	0.324915042	0.087023062	-0.23789198

Accession	Gene_Symbol	Gene_ID	Ensembl_Gene ID	log2(ratio(CM12H/CM0H))	log2(ratio(CM12R/CM0H))	log2(ratio(CM12H/CM12R))
F6Q7Z4	JAM3	F6Q7Z4	ENSBTAG00000003176	-0.13290519	-0.342614496	-0.209709306
F1N079	RTEL1	F1N079	ENSBTAG00000021096	-0.263601392	-0.015673878	0.247927513
A0AAA9SEY1	NRBF2	A0AAA9SEY1	ENSBTAG00000012476	0.162938571	0.060033709	-0.102904862
F1MPF0	TRPM7	bta:514642; F1MPF0	ENSBTAG00000031165	-0.040798723	0.142647419	0.183446141
Q0VCQ3	MXI1	bta:614509; F1MDY4; Q0VCQ3	ENSBTAG00000017263	0.022554316	0.117806776	0.095252459
A0A3Q1M7Q5	C1S	A0A3Q1M7Q5	ENSBTAG00000004840	0.033501744	0.153623189	0.120121446
F1MNI1	CEP164	F1MNI1	ENSBTAG00000014137	0.008988783	-0.138706271	-0.147695054
F1MAV6	RFWD3	bta:540072; F1MAV6	ENSBTAG00000004460	0.260664799	0.211073557	-0.049591242
A0AAA9SJ60	SEMA4C	A0AAA9SJ60	ENSBTAG00000021920	-0.046402232	0.009954564	0.056356796
A0AAA9S953	RAD1	A0AAA9S953	ENSBTAG00000019209	0.325826794	0.278332294	-0.0474945
A0A3Q1LYR8	STN1	A0A3Q1LYR8	ENSBTAG00000015019	-0.087907089	-0.409835184	-0.321928095
A0AAA9SQ08	ABCC5	A0AAA9SQ08	ENSBTAG00000063100	-0.151109507	-0.101356472	0.049753035
A0A452DIJ0	TMUB1	A0A452DIJ0	ENSBTAG00000011229	-0.114951166	0.391565588	0.506516755
A0AAA9TB69	R3HDM4	A0AAA9TB69	ENSBTAG00000046406	-0.203394319	0.199703567	0.403097886
F1N4C5	PLXND1	F1N4C5	ENSBTAG00000001814	0.041080985	0.105272043	0.064191058
Q3SZ23	DNAJC30	bta:617118; F1MW37; Q3SZ23	ENSBTAG00000009022	-0.536302869	-0.482231046	0.054071823
E1U8D1	WWOX	E1U8D1		-0.87285472	-0.247062433	0.625792287
A6QQY8	FASTKD1	A6QQY8; bta:515744	ENSBTAG00000010743	-0.13865354	-0.095026607	0.043626932
F1MZB7	ZBTB10	F1MZB7	ENSBTAG00000003339	-0.841225826	-0.973644523	-0.132418697
E1BP61	SLC23A2	bta:783536; E1BP61	ENSBTAG00000032366	0.008732292	-0.035467512	-0.044199804
E1BDN7	JAG1	bta:783681; E1BDN7	ENSBTAG00000012817	-0.00308598	-0.156119202	-0.153033222
A0AAA9SCL3	TTPAL	A0AAA9SCL3	ENSBTAG00000018873	0.196823172	0.182146596	-0.014676577
E1BIU4	ZC3H7B	E1BIU4	ENSBTAG00000002782	-0.618731926	-0.586956551	0.031775376
E1BP84	ZNF512B	E1BP84	ENSBTAG00000011646	-0.048363022	0.27117848	0.319541502
A0AAA9S9D3	JCAD	A0AAA9S9D3	ENSBTAG00000001204	0.17872195	-0.048795504	-0.227517454
A0A3Q1NJ62	DPAGT1	A0A3Q1NJ62	ENSBTAG00000005371	-0.044781715	-0.108718242	-0.063936527
F6Q8D9	TROAP	F6Q8D9	ENSBTAG00000008499	-0.467974133	-0.385209933	0.0827642
F1MMG1	ZNF414	F1MMG1	ENSBTAG00000007660	-0.074767768	-0.087380044	-0.012612276
A0AAA9SQ88	AFF1	A0AAA9SQ88	ENSBTAG00000018488	-0.045900851	0.067661708	0.113562559
F1MUK3	IGFBP6	F1MUK3	ENSBTAG00000021467	-0.163435665	-0.353812156	-0.190376491
A0A3Q1LZS1	RUNX2	A0A3Q1LZS1	ENSBTAG00000004104	0.135106024	-0.013235827	-0.148341851
E1BAL1	TPGS1	bta:505683; E1BAL1	ENSBTAG00000010772	-0.233752284	-0.046820857	0.186931427
A0AAA9S1J0	KLHL20	A0AAA9S1J0	ENSBTAG00000024485	0.09356704	0.023246344	-0.070320695
Q29RS3	IQCB1	bta:534598; Q29RS3	ENSBTAG00000013069	-0.249008277	-0.16461609	0.084392187
P26892	IL6	A9QWQ9; bta:280826; P26892; Q08DT2	ENSBTAG00000014921	-0.50963525	0.314986485	0.824621735
A0A3Q1N198	CENPP	A0A3Q1N198	ENSBTAG00000011814	0.045638082	-0.223054968	-0.268693049
F1MDA3	SLC7A6	bta:530661; F1MDA3	ENSBTAG00000012112	-0.065251185	-0.12882998	-0.063578796
A0AAA9SLP7	ZFAND5	A0AAA9SLP7	ENSBTAG00000009417	-0.662637685	-0.948866037	-0.286228352
Q1JPI2	AP3S2	Q1JPI2		0.318894744	0.121034751	-0.197859993
Q1LZA9	TRIM27	bta:520111; F1MTX4; Q1LZA9	ENSBTAG00000000871	0.152800824	0.198021216	0.045220393
G3N0N0				0.380797987	0.462942813	0.082144826
Q05B90	CACFD1	bta:512024; Q05B90	ENSBTAG00000015097	-0.046934208	0.086525844	0.133460051
Q58D25	KLHDC3	Q58D25		-0.479217461	-0.62060688	-0.141389419
F1MWE5	MTERF4	bta:512153; F1MWE5	ENSBTAG00000000784	0.045176139	-0.102509352	-0.147685491
A0AAA9RVH2	MTX3	A0AAA9RVH2; bta:616872	ENSBTAG00000013132	0.017811276	-0.110135507	-0.127946783
A0AAA9TZV5	CCDC14	A0AAA9TZV5	ENSBTAG00000000566	-0.275471509	0.015397105	0.290868614
Q5E9Z8	LSM1	bta:535447; Q5E9Z8	ENSBTAG00000001612	0.276230573	0.431739885	0.155509312
A5D9H3	TMEM184C	A5D9H3	ENSBTAG00000010376	0.236802864	-0.049582029	-0.286384893
G3MZL1	CDH5	G3MZL1; G5E588	ENSBTAG00000007421	-0.197658397	0.312342046	0.510000443
F1MQD6	RSBN1L	bta:541150; F1MQD6	ENSBTAG00000015753	0.040206322	-0.044590151	-0.084796473
F1MPW8	ZFHX4	bta:539762; F1MPW8	ENSBTAG00000033268	-0.660772104	-0.537656786	0.123115318
E1BJA6	FBXO33	bta:539998; E1BJA6	ENSBTAG00000006836	0.335374269	0.349036256	0.013661987
E1BB03	ZRANB3	E1BB03	ENSBTAG00000026842	-0.649430035	-0.374807654	0.27462238
A0A3Q1NJF2	RLIM	A0A3Q1NJF2	ENSBTAG00000015630	0.113514437	-0.078599098	-0.192113535
A0AAA9TCA5	ACOX2	A0AAA9TCA5	ENSBTAG00000004178	-0.146841388	-0.242701404	-0.095860015
G3N179	SLC7A11	G3N179	ENSBTAG00000034075	0.102013075	0.304201926	0.202188851
A0A3Q1LP62	TNRC6C	A0A3Q1LP62	ENSBTAG00000018658	0.279751801	-0.065001904	-0.344753705
A0AAA9TDW4	ZDHHC7	A0AAA9TDW4	ENSBTAG00000002639	-0.166440008	-0.031186594	0.135253414
A0AAF6YSS0	SFXN4	A0AAF6YSS0	ENSBTAG00000008730	0.055495113	0.164455532	0.10896042
E1B8H8	SEC14L3	bta:521133; E1B8H8	ENSBTAG00000014464	0.228268988	0.282143229	0.053874241
A0A3Q1MSZ7	PEX26	A0A3Q1MSZ7	ENSBTAG00000011826	-0.044303227	0.087462841	0.131766068
A7E347	ZNF771	A7E347; bta:100135053		-0.104530583	-0.083009776	0.021520807
A0AAA9S2J8	CEP112	A0AAA9S2J8	ENSBTAG00000033680	-0.109201265	-0.038875491	0.070325774
