# Supplementary material for: Establishment and characterization of Hanwoo cumulus cell line for heat stress studies
Source: Anim Biosci. 2026 Jun 15;39(7):250896. doi: 10.5713/ab.250896 (PMC13353149; doi:10.5713/ab.250896)

Supplement 5. Uncropped electrophoresis image of SV40-T insertion genes

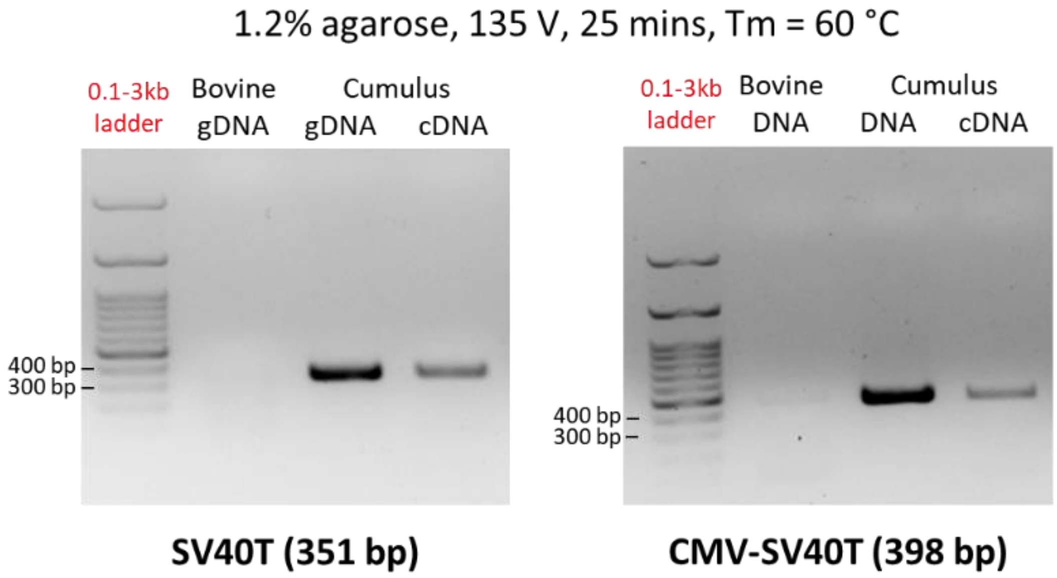

Supplement: Supplementary file 5 [file ab-250896-Supplementary-5.pdf]
