# Supplementary material for: Establishment and characterization of Hanwoo cumulus cell line for heat stress studies
Source: Anim Biosci. 2026 Jun 15;39(7):250896. doi: 10.5713/ab.250896 (PMC13353149; doi:10.5713/ab.250896)
Supplement: Supplementary file 7 [file ab-250896-Supplementary-7.pdf]

Supplement 7. STRING and enrichment analysis (www.string-db.org) of cumulus cell differentially expressed protein (DEP) under heat stress at fold change treshold ≥ |2|. (A) Protein STRING network of upregulated DEP (fc ≥ 2) from HS/CON and REC/CON group. (B) Protein STRING network of downregulated DEP (fc ≤ 2) from HS/CON and REC/CON group. (C) Summary of enrichment analysis on GO, KEGG, and Reactome database. Detail of protein networks statistics are on the parenthes and line thickness indicates the strength.

A

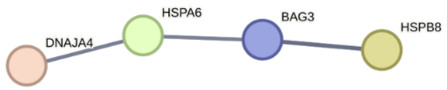

Network statistic
minimum interaction score: 0.700
number of nodes: 13
number of edges: 3
average node degree: 0.462
avg. local clustering coefficient: 0.154
expected number of edges: 0
PPI enrichment p-value: 0.000328

B

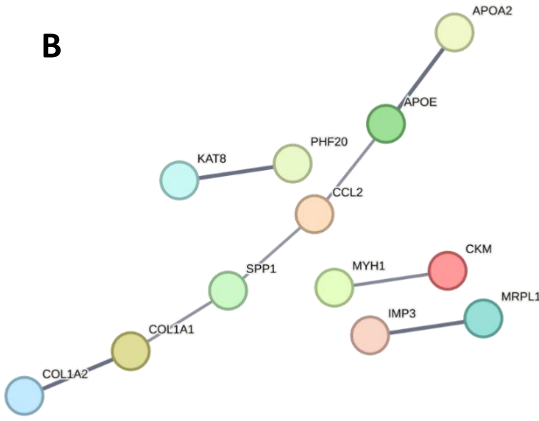

Network statistic
minimum interaction score: 0.700
number of nodes: 33
number of edges: 8
average node degree: 0.485
avg. local clustering coefficient: 0.242
expected number of edges: 3
PPI enrichment p-value: 0.0223

C

Category	Term ID	Term description	Gene count	Strength	Signal	False discovery rate	Protein list
**Upregulated DEP**							
(No enrichment in GO, KEGG, and Reactome database)							
**Downregulated DEP**							
GO Process	GO:0032375	Negative regulation of cholesterol transport	3	2.11	0.53	0.0447	APOE, ABCA2, APOA2
GO Component	GO:0005584	Collagen type I trimer	2	2.78	0.63	0.0263	COL1A1, COL1A2
GO Component	GO:0005788	Endoplasmic reticulum lumen	6	1.06	0.51	0.0263	COL1A1, APOE, COL1A2, APOA2, MSLN, SPP1
KEGG	©04512	ECM-receptor interaction	4	1.43	0.79	0.0055	COL1A1, COL1A2, CD47, SPP1
Reactome	©-3000480	Scavenging by Class A Receptors	3	2	0.74	0.0127	COL1A1, APOE, COL1A2
Reactome	©-216083	Integrin cell surface interactions	4	1.45	0.65	0.0163	COL1A1, COL1A2, CD47, SPP1
Reactome	©-8957275	Post-translational protein phosphorylation	4	1.35	0.57	0.026	APOE, APOA2, MSLN, SPP1
Reactome	©-381426	Regulation of Insulin-like Growth Factor (IGF) transport and uptake by IGFBPs	4	1.28	0.52	0.0341	APOE, APOA2, MSLN, SPP1
