# Supplementary material for: Establishment and characterization of Hanwoo cumulus cell line for heat stress studies
Source: Anim Biosci. 2026 Jun 15;39(7):250896. doi: 10.5713/ab.250896 (PMC13353149; doi:10.5713/ab.250896)
Supplement: Supplementary file 8 [file ab-250896-Supplementary-8.pdf]

Supplement 8. Venn diagram of concordant DEG-DEP and STRING analysis of upregulated DEG-DEP. (A) Venn diagram of genes with concordant expression in both DEG and DEP of the corresponding diagram intersections and (B-C) STRING analysis of upregulated DEG and DEP showing (B) protein-protein network (p-value < 0.01) with common enrichment (FDR < 0.05) to protein refolding, response to unfolded protein, and HSF-1 dependent transactivation, and (C) table of the STRING analysis.

A

B

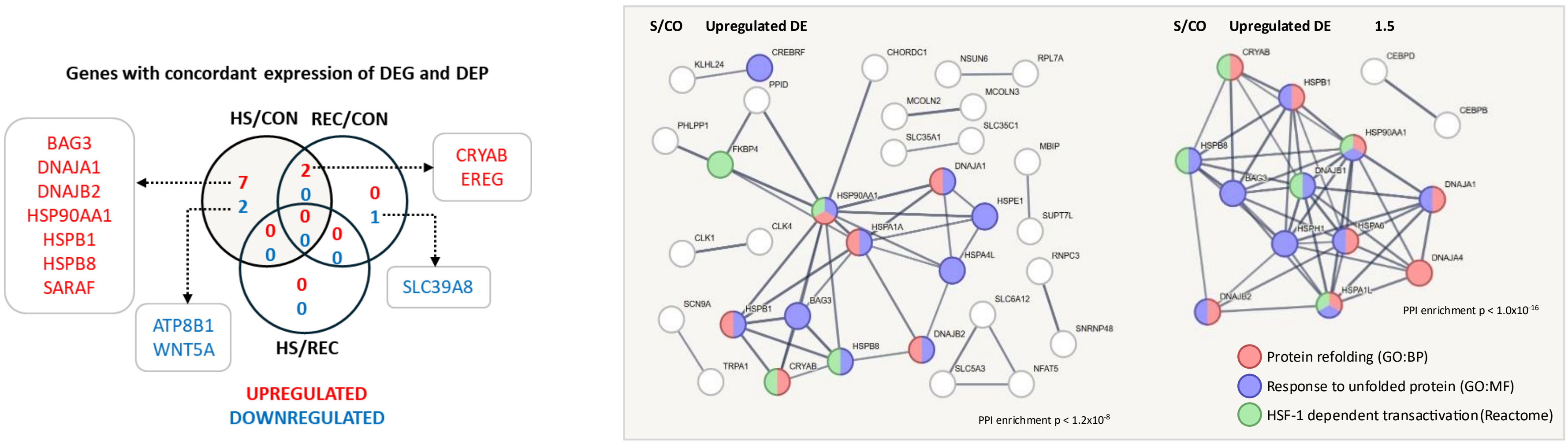

C

Category	Term ID	Term description	Gene count		Strength		Signal		False discovery rate		Protein list
			DEG	DEP	DEG	DEP	DEG	DEP	DEG	DEP	
GO Process	GO:0042026	Protein refolding	6	8	01.41	2.21	0.76	3.7	0.0014	2.27e-11	**DEG:** HSPB1, HSP90AA1, DNAJB2, HSPA1A, DNAJA1, CRYAB<br>**DEP:** HSPB1, HSPA6, HSP90AA1, DNAJB2, HSPA1L, DNAJA1, DNAJA4, CRYAB
GO Process	GO:0006986	Response to unfolded protein	11	10	0.98	1.62	0.81	2.65	0.0004	5.35e-10	**DEG:** EIF2AK2, HSPE1, HSPB1, HSPB8, HSPA4L, CREBRF, HSP90AA1, DNAJB2, BAG3, HSPA1A, DNAJA1<br>**DEP:** HSPB1, DNAJB1, HSPB8, HSPA6, HSP90AA1, DNAJB2, BAG3, HSPA1L, DNAJA1, HSPH1
Reactome	HSA-3371571	HSF1-dependent transactivation	5	5	1.36	2.04	0.52	1.99	0.0125	2.50e-6	**DEG:** DNAJB1, HSPB8, HSP90AA1, HSPA1L, CRYAB<br>**DEP:** FKBP4, HSPB8, HSP90AA1, HSBP1, CRYAB
