# Supplementary material for: Establishment and characterization of Hanwoo cumulus cell line for heat stress studies
Source: Anim Biosci. 2026 Jun 15;39(7):250896. doi: 10.5713/ab.250896 (PMC13353149; doi:10.5713/ab.250896)
Supplement: Supplementary file 9 [file ab-250896-Supplementary-9.pdf]

Supplement 9. Cumulus upregulated DEG - GO enrichment (BP, MF)

Cluster	ONTOLOGY	ID	Description	GeneRatio	BgRatio	pvalue	p.adjust	qvalue	geneID	Count
HS/REC	BP	GO:0009408	response to heat	8/66	103/18870	2.64436E-09	3.51436E-06	3.22891E-06	HSPA1A/HSPB1/BAG3/HSP90AA1/CHORDC1/HSBP1/DNAJB1/DNAJA4	8
HS/REC	BP	GO:0009266	response to temperature stimulus	9/66	171/18870	7.93053E-09	5.26983E-06	4.84179E-06	HSPA1A/HSPB1/SCN9A/BAG3/HSP90AA1/CHORDC1/HSBP1/DNAJB1/DNAJA4	9
HS/REC	BP	GO:0006457	protein folding	9/66	223/18870	7.89434E-08	3.49719E-05	3.21313E-05	HSPA1A/HSPB1/BAG3/HSP90AA1/CHORDC1/FKBP4/HSPA4L/DNAJB1/DNAJA4	9
HS/REC	BP	GO:0034605	cellular response to heat	6/66	66/18870	1.11858E-07	3.71648E-05	3.41461E-05	HSPA1A/BAG3/HSP90AA1/CHORDC1/HSBP1/DNAJB1	6
HS/CON	BP	GO:0035966	response to topologically incorrect protein	12/166	159/18870	1.67988E-08	3.79653E-05	3.65506E-05	CLU/HSPA1A/HSPB1/BAG3/HSP90AA1/HSPB8/DNAJB2/CREBRF/HSPA4L/EIF2AK2/DNAJA1/HSPE1	12
HS/CON	BP	GO:0006986	response to unfolded protein	11/166	137/18870	3.56511E-08	4.02858E-05	3.87847E-05	HSPA1A/HSPB1/BAG3/HSP90AA1/HSPB8/DNAJB2/CREBRF/HSPA4L/EIF2AK2/DNAJA1/HSPE1	11
HS/CON	BP	GO:0006457	protein folding	13/166	223/18870	9.06479E-08	6.07517E-05	5.8488E-05	CRYAB/CLU/HSPA1A/HSPB1/BAG3/HSP90AA1/CHORDC1/DNAJB2/PPID/FKBP4/HSPA4L/DNAJA1/HSPE1	13
HS/CON	BP	GO:0042026	protein refolding	6/166	27/18870	1.07525E-07	6.07517E-05	5.8488E-05	CRYAB/HSPA1A/HSPB1/HSP90AA1/DNAJB2/DNAJA1	6
HS/REC	BP	GO:0006986	response to unfolded protein	7/66	137/18870	4.96755E-07	0.000132038	0.000121313	HSPA1A/HSPB1/BAG3/HSP90AA1/HSPB8/HSPA4L/DNAJB1	7
HS/CON	BP	GO:0061077	chaperone-mediated protein folding	8/166	75/18870	3.09725E-07	0.000139996	0.000134779	CLU/HSPA1A/HSPB1/CHORDC1/DNAJB2/PPID/FKBP4/HSPE1	8
HS/REC	BP	GO:0035966	response to topologically incorrect protein	7/66	159/18870	1.35575E-06	0.0002612	0.000239984	HSPA1A/HSPB1/BAG3/HSP90AA1/HSPB8/HSPA4L/DNAJB1	7
HS/REC	BP	GO:0070841	inclusion body assembly	4/66	24/18870	1.37577E-06	0.0002612	0.000239984	HSPA1A/BAG3/DNAJB1/DNAJA4	4
HS/REC	BP	GO:0042026	protein refolding	4/66	27/18870	2.25439E-06	0.00037451	0.000344091	HSPA1A/HSPB1/HSP90AA1/DNAJA4	4
HS/REC	BP	GO:0061077	chaperone-mediated protein folding	5/66	75/18870	6.40961E-06	0.000946486	0.000869608	HSPA1A/HSPB1/CHORDC1/FKBP4/DNAJB1	5
HS/REC	MF	GO:0044183	protein folding chaperone	5/69	66/18496	4.67186E-06	0.00097902	0.000845135	HSPA1A/HSPB1/HSP90AA1/HSPA4L/DNAJB1	5
HS/REC	MF	GO:0031072	heat shock protein binding	6/69	128/18496	8.19264E-06	0.00097902	0.000845135	HSPA1A/CHORDC1/FKBP4/DNAJB1/CYP1A1/DNAJA4	6
HS/CON	BP	GO:0009266	response to temperature stimulus	10/166	171/18870	2.82573E-06	0.001064358	0.001024699	CRYAB/HSPA1A/HSPB1/SCN9A/BAG3/HSP90AA1/CHORDC1/HSBP1/DNAJA1/TRPA1	10
HS/CON	BP	GO:0009408	response to heat	8/166	103/18870	3.54384E-06	0.001144153	0.00110152	CRYAB/HSPA1A/HSPB1/BAG3/HSP90AA1/CHORDC1/HSBP1/DNAJA1	8
HS/REC	BP	GO:0090084	negative regulation of inclusion body assembly	3/66	12/18870	8.79057E-06	0.001168267	0.001073375	HSPA1A/DNAJB1/DNAJA4	3
HS/REC	BP	GO:0090083	regulation of inclusion body assembly	3/66	17/18870	2.68328E-05	0.00324189	0.002978569	HSPA1A/DNAJB1/DNAJA4	3
HS/CON	MF	GO:0051082	unfolded protein binding	8/173	122/18496	1.9391E-05	0.007116506	0.006572536	CRYAB/CLU/HSPA1A/HSPB1/HSP90AA1/DNAJB2/DNAJA1/HSPE1	8
HS/REC	MF	GO:0051082	unfolded protein binding	5/69	122/18496	9.22298E-05	0.007347643	0.006342823	HSPA1A/HSPB1/HSP90AA1/DNAJB1/DNAJA4	5
HS/CON	BP	GO:0070841	inclusion body assembly	4/166	24/18870	5.34948E-05	0.015112268	0.014549165	CLU/HSPA1A/BAG3/DNAJB2	4
HS/REC	MF	GO:0140662	ATP-dependent protein folding chaperone	3/69	40/18496	0.000444697	0.02657065	0.022937007	HSPA1A/HSP90AA1/HSPA4L	3
HS/REC	MF	GO:0060590	ATPase regulator activity	3/69	49/18496	0.000809608	0.036258457	0.031299967	BAG3/HSPA4L/DNAJB1	3
HS/REC	MF	GO:0030544	Hsp70 protein binding	3/69	51/18496	0.000910254	0.036258457	0.031299967	DNAJB1/CYP1A1/DNAJA4	3
HS/CON	BP	GO:0031647	regulation of protein stability	11/166	325/18870	0.000147152	0.036951535	0.035574673	CRYAB/CLU/HSPA1A/SERF2/TRIM21/FLOT1/BAG3/HSP90AA1/TMEM183A/WNT10B/DPM2	11
HS/REC	MF	GO:0000774	adenyl-nucleotide exchange factor activity	2/69	15/18496	0.001395675	0.045978426	0.039690691	BAG3/HSPA4L	2
HS/REC	MF	GO:0051087	protein-folding chaperone binding	4/69	133/18496	0.001539027	0.045978426	0.039690691	BAG3/DNAJB1/CYP1A1/DNAJA4	4
HS/REC	BP	GO:0043484	regulation of RNA splicing	5/66	181/18870	0.000427711	0.04736898	0.043521458	HSPA1A/CLK1/CLK4/DDX17/METTL4	5
