# Supplementary material for: Establishment and characterization of Hanwoo cumulus cell line for heat stress studies
Source: Anim Biosci. 2026 Jun 15;39(7):250896. doi: 10.5713/ab.250896 (PMC13353149; doi:10.5713/ab.250896)
Supplement: Supplementary file 10 [file ab-250896-Supplementary-10.pdf]

Supplement 10. Cumulus downregulated DEG - GO enrichment (BP, MF)

Cluster	ONTOLOGY	ID	Description	GeneRatio	BgRatio	pvalue	p.adjust	qvalue	geneID	Count
HS/CON	MF	GO:0005125	cytokine activity	10/118	237/18496	2.86997E-06	0.000636139	0.000589557	TGFB2/BMP7/WNT5A/GREM1/LIF/GDF5/TNFRSF11B/INHBA/IL36A/IL6	10
HS/CON	MF	GO:0008514	organic anion transmembrane transporter activity	10/118	242/18496	3.45728E-06	0.000636139	0.000589557	SFXN3/SLC17A9/SLC6A6/SLC39A8/SLC36A4/SLC43A3/MFSD2A/MFSD12/SLC7A11/SLC25A21	10
HS/CON	MF	GO:0046943	carboxylic acid transmembrane transporter activity	8/118	164/18496	1.0305E-05	0.000794489	0.000736311	SFXN3/SLC6A6/SLC36A4/SLC43A3/MFSD2A/MFSD12/SLC7A11/SLC25A21	8
HS/CON	MF	GO:0005342	organic acid transmembrane transporter activity	8/118	165/18496	1.0773E-05	0.000794489	0.000736311	SFXN3/SLC6A6/SLC36A4/SLC43A3/MFSD2A/MFSD12/SLC7A11/SLC25A21	8
HS/CON	MF	GO:0015175	neutral L-amino acid transmembrane transporter activity	5/118	46/18496	1.07947E-05	0.000794489	0.000736311	SFXN3/SLC6A6/SLC36A4/MFSD12/SLC7A11	5
HS/REC	BP	GO:0002237	response to molecule of bacterial origin	15/162	369/18870	7.08915E-07	0.001121428	0.000909823	ABCA1/TNFAIP3/NAGLU/VCAM1/MIR19A/CD274/CCR7/EDN1/FOS/CD36/MAPKAPK3/ANKRD1/MAPK3/THBD/IL6	15
HS/REC	BP	GO:0034614	cellular response to reactive oxygen species	10/162	154/18870	8.74801E-07	0.001121428	0.000909823	TNFAIP3/STAT6/EDN1/FOS/MDM2/ERCC6L2/CD36/MAPK9/MAPK3/IL6	10
HS/REC	BP	GO:0060537	muscle tissue development	16/162	433/18870	1.04937E-06	0.001121428	0.000909823	NAGLU/MIR19A/SORBS2/EDN1/FOS/TFAP2B/MAFF/GTF3C5/EGR1/NLN/ANKRD1/MYLK/PRICKLE1/BCL2/PRMT1/SEMA3C	16
HS/REC	BP	GO:0000302	response to reactive oxygen species	11/162	205/18870	1.62566E-06	0.00122537	0.000994152	TNFAIP3/STAT6/EDN1/FOS/MDM2/ERCC6L2/CD36/MAPK9/MAPK3/BCL2/IL6	11
HS/REC	BP	GO:0032496	response to lipopolysaccharide	14/162	348/18870	1.91106E-06	0.00122537	0.000994152	ABCA1/TNFAIP3/NAGLU/VCAM1/CD274/CCR7/EDN1/FOS/CD36/MAPKAPK3/ANKRD1/MAPK3/THBD/IL6	14
HS/CON	MF	GO:0015291	secondary active transmembrane transporter activity	9/118	286/18496	9.15001E-05	0.005612007	0.005201059	SLC17A9/SLC6A6/SLC39A8/SLC36A4/SLC35E3/MFSD2A/MFSD12/SLC7A11/SLC25A21	9
HS/REC	BP	GO:0035051	cardiocyte differentiation	9/162	165/18870	1.29482E-05	0.006004903	0.004871824	NAGLU/VCAM1/MIR19A/SORBS2/EDN1/MAPK3/PRICKLE1/INHBA/SEMA3C	9
HS/REC	BP	GO:0034599	cellular response to oxidative stress	11/162	255/18870	1.31111E-05	0.006004903	0.004871824	TNFAIP3/STAT6/NAGLU/EDN1/FOS/MDM2/ERCC6L2/CD36/MAPK9/MAPK3/IL6	11
HS/REC	BP	GO:0007052	mitotic spindle organization	8/162	130/18870	1.66033E-05	0.006653769	0.005398254	CENPE/WDR62/CENPH/TACC3/PLK1/TBCE/PLK2/PRICKLE1	8
HS/REC	BP	GO:0062197	cellular response to chemical stress	12/162	317/18870	1.94926E-05	0.006875018	0.005577755	TNFAIP3/STAT6/NAGLU/EDN1/FOS/MDM2/ERCC6L2/CD36/MAPK9/MYLK/MAPK3/IL6	12
HS/REC	BP	GO:1901653	cellular response to peptide	13/162	374/18870	2.14442E-05	0.006875018	0.005577755	OTOP1/ICAM1/STAT6/PLCB1/VCAM1/PTPRE/TYK2/EDN1/FOS/MDM2/CD36/MAPK3/INHBA	13
HS/REC	BP	GO:0033630	positive regulation of cell adhesion mediated by integrin	4/162	22/18870	3.39374E-05	0.009891217	0.008024821	IFT74/NCKAP1L/FERMT1/LIF	4
HS/CON	BP	GO:0035051	cardiocyte differentiation	8/121	165/18870	1.12256E-05	0.010228408	0.008397246	TGFB2/BMP7/MAPK3/GREM1/PRICKLE1/MYH11/INHBA/SEMA3C	8
HS/CON	BP	GO:0022612	gland morphogenesis	7/121	122/18870	1.36331E-05	0.010228408	0.008397246	TGFB2/BMP7/WNT5A/ELF3/BCL2/SEMA3C/IL6	7
HS/CON	BP	GO:0006582	melanin metabolic process	4/121	26/18870	2.156E-05	0.010228408	0.008397246	WNT5A/BCL2/MFSD12/SLC7A11	4
HS/CON	BP	GO:0048736	appendage development	8/121	182/18870	2.28425E-05	0.010228408	0.008397246	TGFB2/BMP7/WNT5A/GREM1/PRICKLE1/GDF5/SEMA3C/SLC7A11	8
HS/CON	BP	GO:0060173	limb development	8/121	182/18870	2.28425E-05	0.010228408	0.008397246	TGFB2/BMP7/WNT5A/GREM1/PRICKLE1/GDF5/SEMA3C/SLC7A11	8
HS/CON	BP	GO:0048771	tissue remodeling	8/121	184/18870	2.47084E-05	0.010228408	0.008397246	GREM1/ELF3/LIF/GDF5/TNFRSF11B/S1PR1/SEMA3C/IL6	8
HS/CON	BP	GO:0015711	organic anion transport	12/121	443/18870	2.80498E-05	0.010228408	0.008397246	ATP8B1/SFXN3/SLC17A9/SLC6A6/SLC39A8/SLC36A4/SLC43A3/MFSD2A/FABP4/MFSD12/SLC7A11/SLC25A21	12
HS/CON	BP	GO:0015804	neutral amino acid transport	5/121	58/18870	3.48412E-05	0.010228408	0.008397246	SFXN3/SLC6A6/SLC36A4/MFSD12/SLC7A11	5
HS/CON	BP	GO:0035107	appendage morphogenesis	7/121	146/18870	4.34802E-05	0.010228408	0.008397246	TGFB2/BMP7/WNT5A/GREM1/PRICKLE1/GDF5/SEMA3C	7
HS/CON	BP	GO:0035108	limb morphogenesis	7/121	146/18870	4.34802E-05	0.010228408	0.008397246	TGFB2/BMP7/WNT5A/GREM1/PRICKLE1/GDF5/SEMA3C	7
HS/CON	BP	GO:0072001	renal system development	10/121	324/18870	4.55555E-05	0.010228408	0.008397246	TGFB2/BMP7/WNT5A/GREM1/PRICKLE1/LIF/BCL2/ACTA2/HS3ST3A1/ANGPT2	10
HS/CON	BP	GO:0060485	mesenchyme development	10/121	327/18870	4.92211E-05	0.010228408	0.008397246	TGFB2/BMP7/MAPK3/WNT5A/GREM1/PRICKLE1/BCL2/ACTA2/SEMA3C/IL6	10
HS/CON	BP	GO:0014706	striated muscle tissue development	9/121	264/18870	5.17348E-05	0.010228408	0.008397246	TGFB2/BMP7/WNT5A/GREM1/PRICKLE1/MYH11/PRMT1/S1PR1/SEMA3C	9
HS/CON	BP	GO:0051798	positive regulation of hair follicle development	3/121	12/18870	5.42416E-05	0.010228408	0.008397246	TGFB2/WNT5A/FST	3
HS/REC	BP	GO:0035914	skeletal muscle cell differentiation	6/162	73/18870	3.8629E-05	0.010320388	0.008373011	FOS/MAFF/GTF3C5/EGR1/NLN/ANKRD1	6
HS/CON	MF	GO:0015171	amino acid transmembrane transporter activity	5/118	85/18496	0.000211942	0.011142118	0.01032622	SFXN3/SLC6A6/SLC36A4/MFSD12/SLC7A11	5
HS/CON	BP	GO:0072006	nephron development	7/121	156/18870	6.6212E-05	0.011539725	0.009473801	BMP7/GREM1/LIF/BCL2/ACTA2/HS3ST3A1/ANGPT2	7
HS/CON	BP	GO:0046942	carboxylic acid transport	10/121	351/18870	8.87285E-05	0.011539725	0.009473801	ATP8B1/SFXN3/SLC6A6/SLC36A4/SLC43A3/MFSD2A/FABP4/MFSD12/SLC7A11/SLC25A21	10
HS/CON	BP	GO:0071276	cellular response to cadmium ion	4/121	37/18870	9.01977E-05	0.011539725	0.009473801	MAPK3/SLC39A8/MT1A/MT2A	4
HS/CON	BP	GO:0015849	organic acid transport	10/121	352/18870	9.0835E-05	0.011539725	0.009473801	ATP8B1/SFXN3/SLC6A6/SLC36A4/SLC43A3/MFSD2A/FABP4/MFSD12/SLC7A11/SLC25A21	10
HS/CON	BP	GO:0030509	BMP signaling pathway	7/121	167/18870	0.000101598	0.011539725	0.009473801	BMP7/MAPK3/WNT5A/GREM1/GDF5/PRMT1/FST	7
HS/CON	BP	GO:0060537	muscle tissue development	11/121	433/18870	0.000108592	0.011539725	0.009473801	TGFB2/MYLK/BMP7/WNT5A/GREM1/PRICKLE1/MYH11/BCL2/PRMT1/S1PR1/SEMA3C	11
HS/CON	BP	GO:0033689	negative regulation of osteoblast proliferation	3/121	15/18870	0.000110614	0.011539725	0.009473801	GREM1/BCL2/MIR138-1	3
HS/CON	BP	GO:0061307	cardiac neural crest cell differentiation involved in heart development	3/121	15/18870	0.000110614	0.011539725	0.009473801	BMP7/MAPK3/SEMA3C	3
HS/CON	BP	GO:0061308	cardiac neural crest cell development involved in heart development	3/121	15/18870	0.000110614	0.011539725	0.009473801	BMP7/MAPK3/SEMA3C	3
HS/CON	BP	GO:0030326	embryonic limb morphogenesis	6/121	120/18870	0.000123592	0.011539725	0.009473801	TGFB2/BMP7/WNT5A/GREM1/PRICKLE1/GDF5	6
HS/CON	BP	GO:0035113	embryonic appendage morphogenesis	6/121	120/18870	0.000123592	0.011539725	0.009473801	TGFB2/BMP7/WNT5A/GREM1/PRICKLE1/GDF5	6
HS/CON	BP	GO:0050920	regulation of chemotaxis	8/121	232/18870	0.000126331	0.011539725	0.009473801	MAPK3/ITGA2/WNT5A/GREM1/ANGPT2/S1PR1/SEMA3C/IL6	8
HS/CON	BP	GO:0030278	regulation of ossification	6/121	121/18870	0.000129366	0.011539725	0.009473801	BMP7/MAPK3/WNT5A/GREM1/BCL2/S1PR1	6
HS/CON	BP	GO:0048732	gland development	11/121	443/18870	0.000132685	0.011539725	0.009473801	TGFB2/BMP7/MAPK3/ITGA2/WNT5A/HNRNPD/ELF3/BCL2/PCK2/SEMA3C/IL6	11
HS/CON	BP	GO:0043455	regulation of secondary metabolic process	3/121	16/18870	0.000135504	0.011539725	0.009473801	WNT5A/MFSD12/SLC7A11	3
HS/CON	BP	GO:0048021	regulation of melanin biosynthetic process	3/121	16/18870	0.000135504	0.011539725	0.009473801	WNT5A/MFSD12/SLC7A11	3
HS/CON	BP	GO:1900376	regulation of secondary metabolite biosynthetic process	3/121	16/18870	0.000135504	0.011539725	0.009473801	WNT5A/MFSD12/SLC7A11	3
HS/CON	BP	GO:0071772	response to BMP	7/121	179/18870	0.000156376	0.01229536	0.010094157	BMP7/MAPK3/WNT5A/GREM1/GDF5/PRMT1/FST	7
HS/CON	BP	GO:0071773	cellular response to BMP stimulus	7/121	179/18870	0.000156376	0.01229536	0.010094157	BMP7/MAPK3/WNT5A/GREM1/GDF5/PRMT1/FST	7
HS/CON	BP	GO:0003151	outflow tract morphogenesis	5/121	81/18870	0.00017325	0.01229536	0.010094157	TGFB2/BMP7/WNT5A/PRICKLE1/SEMA3C	5
HS/CON	BP	GO:0048644	muscle organ morphogenesis	5/121	81/18870	0.00017325	0.01229536	0.010094157	TGFB2/MYLK/WNT5A/LIF/S1PR1	5
HS/CON	BP	GO:0072028	nephron morphogenesis	5/121	81/18870	0.00017325	0.01229536	0.010094157	BMP7/GREM1/LIF/BCL2/HS3ST3A1	5
HS/CON	BP	GO:0048863	stem cell differentiation	8/121	246/18870	0.000188747	0.01229536	0.010094157	TGFB2/BMP7/MAPK3/GREM1/PRICKLE1/LIF/BCL2/SEMA3C	8
HS/CON	BP	GO:0001822	kidney development	9/121	314/18870	0.000192949	0.01229536	0.010094157	TGFB2/BMP7/WNT5A/GREM1/LIF/BCL2/ACTA2/HS3ST3A1/ANGPT2	9
HS/CON	BP	GO:0045926	negative regulation of growth	8/121	247/18870	0.000194034	0.01229536	0.010094157	TGFB2/WNT5A/GREM1/MT1A/BCL2/MT2A/INHBA/SEMA3C	8
HS/CON	BP	GO:0048738	cardiac muscle tissue development	8/121	247/18870	0.000194034	0.01229536	0.010094157	TGFB2/BMP7/WNT5A/GREM1/PRICKLE1/MYH11/PRMT1/S1PR1	8
HS/CON	BP	GO:0010002	cardioblast differentiation	3/121	18/18870	0.000195608	0.01229536	0.010094157	TGFB2/GREM1/PRICKLE1	3
HS/CON	BP	GO:0046851	negative regulation of bone remodeling	3/121	18/18870	0.000195608	0.01229536	0.010094157	GREM1/TNFRSF11B/IL6	3
HS/CON	BP	GO:0006935	chemotaxis	11/121	468/18870	0.000213663	0.012790006	0.010500249	TGFB2/MAPK3/ITGA2/WNT5A/GREM1/RAC3/ANGPT2/S1PR1/DEFB1/SEMA3C/IL6	11
HS/CON	BP	GO:0042330	taxis	11/121	470/18870	0.000221646	0.012790006	0.010500249	TGFB2/MAPK3/ITGA2/WNT5A/GREM1/RAC3/ANGPT2/S1PR1/DEFB1/SEMA3C/IL6	11
HS/CON	BP	GO:0030198	extracellular matrix organization	9/121	321/18870	0.000227255	0.012790006	0.010500249	TGFB2/GREM1/ELF3/PRICKLE1/SLC39A8/MYH11/TNFRSF11B/MMP1/IL6	9
HS/CON	BP	GO:0051797	regulation of hair follicle development	3/121	19/18870	0.000231199	0.012790006	0.010500249	TGFB2/WNT5A/FST	3
HS/CON	BP	GO:0061383	trabecula morphogenesis	4/121	47/18870	0.000231847	0.012790006	0.010500249	TGFB2/BMP7/GREM1/S1PR1	4
HS/CON	BP	GO:0043062	extracellular structure organization	9/121	322/18870	0.000232546	0.012790006	0.010500249	TGFB2/GREM1/ELF3/PRICKLE1/SLC39A8/MYH11/TNFRSF11B/MMP1/IL6	9
HS/CON	BP	GO:0045229	external encapsulating structure organization	9/121	323/18870	0.000237939	0.012819552	0.010524505	TGFB2/GREM1/ELF3/PRICKLE1/SLC39A8/MYH11/TNFRSF11B/MMP1/IL6	9
HS/CON	BP	GO:0046189	phenol-containing compound biosynthetic process	4/121	48/18870	0.000251678	0.013288615	0.010909593	TGFB2/WNT5A/MFSD12/SLC7A11	4
HS/CON	BP	GO:0046849	bone remodeling	5/121	89/18870	0.000269464	0.01384478	0.011366189	GREM1/GDF5/TNFRSF11B/S1PR1/IL6	5
HS/CON	BP	GO:0046850	regulation of bone remodeling	4/121	49/18870	0.0002727	0.01384478	0.011366189	GREM1/TNFRSF11B/S1PR1/IL6	4
HS/REC	BP	GO:0090023	positive regulation of neutrophil chemotaxis	4/162	25/18870	5.75224E-05	0.014185903	0.011509133	CCR7/EDN1/C3AR1/NCKAP1L	4
HS/CON	BP	GO:0048762	mesenchymal cell differentiation	8/121	262/18870	0.000289101	0.014400507	0.011822426	TGFB2/BMP7/MAPK3/WNT5A/GREM1/BCL2/SEMA3C/IL6	8
HS/REC	BP	GO:0007051	spindle organization	9/162	202/18870	6.36667E-05	0.014579674	0.011828603	CENPE/WDR62/CENPH/TACC3/PLK1/TBCE/MAPRE3/PLK2/PRICKLE1	9
HS/CON	BP	GO:0034104	negative regulation of tissue remodeling	3/121	21/18870	0.000314376	0.015090043	0.012388516	GREM1/TNFRSF11B/IL6	3
HS/CON	BP	GO:0097501	stress response to metal ion	3/121	21/18870	0.000314376	0.015090043	0.012388516	SLC39A8/MT1A/MT2A	3
HS/REC	BP	GO:0000079	regulation of cyclin-dependent protein serine/threonine kinase activity	6/162	82/18870	7.45693E-05	0.015398143	0.012492632	PLK1/GTPBP4/HHEX/CCNB3/MAPRE3/DIRAS3	6
HS/REC	BP	GO:1902850	microtubule cytoskeleton organization involved in mitosis	8/162	163/18870	8.35878E-05	0.015398143	0.012492632	CENPE/WDR62/CENPH/TACC3/PLK1/TBCE/PLK2/PRICKLE1	8
HS/REC	BP	GO:0071622	regulation of granulocyte chemotaxis	5/162	53/18870	9.01961E-05	0.015398143	0.012492632	CCR7/EDN1/C3AR1/NCKAP1L/MAPK3	5
HS/REC	BP	GO:1904029	regulation of cyclin-dependent protein kinase activity	6/162	85/18870	9.11876E-05	0.015398143	0.012492632	PLK1/GTPBP4/HHEX/CCNB3/MAPRE3/DIRAS3	6
HS/REC	BP	GO:0071624	positive regulation of granulocyte chemotaxis	4/162	28/18870	9.12554E-05	0.015398143	0.012492632	CCR7/EDN1/C3AR1/NCKAP1L	4
HS/CON	BP	GO:0001942	hair follicle development	5/121	93/18870	0.000330595	0.015585176	0.012795007	TGFB2/WNT5A/BCL2/INHBA/FST	5
HS/CON	BP	GO:0033630	positive regulation of cell adhesion mediated by integrin	3/121	22/18870	0.000362315	0.016491586	0.013539145	TGFB2/RAC3/LIF	3
HS/CON	BP	GO:0061687	detoxification of inorganic compound	3/121	22/18870	0.000362315	0.016491586	0.013539145	SLC39A8/MT1A/MT2A	3
HS/CON	BP	GO:0022404	molting cycle process	5/121	96/18870	0.000382928	0.016848828	0.013832431	TGFB2/WNT5A/BCL2/INHBA/FST	5
HS/CON	BP	GO:0022405	hair cycle process	5/121	96/18870	0.000382928	0.016848828	0.013832431	TGFB2/WNT5A/BCL2/INHBA/FST	5
HS/REC	BP	GO:0070663	regulation of leukocyte proliferation	10/162	267/18870	0.00010666	0.017097551	0.013871375	TNFAIP3/STAT6/VCAM1/CD274/TYK2/HHEX/NCKAP1L/MAPK3/BCL2/IL6	10
HS/CON	BP	GO:0060993	kidney morphogenesis	5/121	98/18870	0.00042114	0.018226408	0.014963387	BMP7/GREM1/LIF/BCL2/HS3ST3A1	5
HS/CON	BP	GO:0001894	tissue homeostasis	8/121	279/18870	0.000439782	0.018428965	0.015129681	RAC3/PRICKLE1/SLC39A8/MFSD2A/BCL2/TNFRSF11B/S1PR1/IL6	8
HS/CON	BP	GO:0060249	anatomical structure homeostasis	8/121	279/18870	0.000439782	0.018428965	0.015129681	RAC3/PRICKLE1/SLC39A8/MFSD2A/BCL2/TNFRSF11B/S1PR1/IL6	8
HS/CON	MF	GO:1901505	carbohydrate derivative transmembrane transporter activity	4/118	55/18496	0.000417664	0.019212529	0.017805662	SLC17A9/SLC50A1/SLC35E3/MFSD2A	4
HS/CON	BP	GO:0042438	melanin biosynthetic process	3/121	24/18870	0.000471753	0.019257661	0.015810017	WNT5A/MFSD12/SLC7A11	3
HS/CON	BP	GO:0003333	amino acid transmembrane transport	5/121	101/18870	0.000483768	0.019257661	0.015810017	SFXN3/SLC6A6/SLC36A4/MFSD12/SLC7A11	5
HS/CON	BP	GO:0019748	secondary metabolic process	4/121	57/18870	0.000488736	0.019257661	0.015810017	WNT5A/BCL2/MFSD12/SLC7A11	4
HS/CON	BP	GO:0072132	mesenchyme morphogenesis	4/121	57/18870	0.000488736	0.019257661	0.015810017	TGFB2/BMP7/WNT5A/ACTA2	4
HS/CON	BP	GO:1905039	carboxylic acid transmembrane transport	6/121	156/18870	0.000509792	0.019672153	0.016150304	SFXN3/SLC6A6/SLC36A4/MFSD12/SLC7A11/SLC25A21	6
HS/CON	BP	GO:0010038	response to metal ion	9/121	359/18870	0.000514159	0.019672153	0.016150304	MAPK3/WNT5A/HNRNPD/SLC39A8/MT1A/BCL2/MT2A/FABP4/TNFRSF11B	9
HS/REC	BP	GO:0045472	response to ether	3/162	12/18870	0.00012909	0.019707702	0.015989011	PLCB1/MDM2/HNRNPD	3
HS/CON	BP	GO:1903825	organic acid transmembrane transport	6/121	157/18870	0.000527309	0.019887079	0.016326753	SFXN3/SLC6A6/SLC36A4/MFSD12/SLC7A11/SLC25A21	6
HS/REC	BP	GO:0032436	positive regulation of proteasomal ubiquitin-dependent protein catabolic process	6/162	93/18870	0.000150215	0.020011156	0.016235206	MDM2/PLK1/USP5/MAPK9/SH3RF2/PRICKLE1	6
HS/REC	BP	GO:0055006	cardiac cell development	6/162	93/18870	0.000150215	0.020011156	0.016235206	NAGLU/MIR19A/SORBS2/EDN1/PRICKLE1/INHBA	6
HS/REC	BP	GO:0034694	response to prostaglandin	4/162	32/18870	0.000156045	0.020011156	0.016235206	A2M/CCR7/EDN1/PTGDR	4
HS/REC	BP	GO:0070498	interleukin-1-mediated signaling pathway	4/162	32/18870	0.000156045	0.020011156	0.016235206	PLCB1/EGR1/MAPK3/IL6	4
HS/CON	BP	GO:0061326	renal tubule development	5/121	104/18870	0.000553164	0.020445083	0.016784859	GREM1/PRICKLE1/LIF/BCL2/HS3ST3A1	5
HS/CON	BP	GO:0046686	response to cadmium ion	4/121	59/18870	0.000557593	0.020445083	0.016784859	MAPK3/SLC39A8/MT1A/MT2A	4
HS/CON	BP	GO:0001503	ossification	10/121	444/18870	0.000581379	0.021025226	0.01726114	BMP7/MAPK3/WNT5A/GREM1/PRICKLE1/GDF5/BCL2/S1PR1/MIR138-1/IL6	10
HS/CON	BP	GO:0044550	secondary metabolite biosynthetic process	3/121	26/18870	0.000600368	0.021132956	0.017349584	WNT5A/MFSD12/SLC7A11	3
HS/CON	BP	GO:0050926	regulation of positive chemotaxis	3/121	26/18870	0.000600368	0.021132956	0.017349584	ITGA2/ANGPT2/S1PR1	3
HS/REC	BP	GO:0002690	positive regulation of leukocyte chemotaxis	6/162	96/18870	0.000178873	0.021401075	0.017362858	CCR7/EDN1/C3AR1/NCKAP1L/MAPK3/IL6	6
HS/REC	BP	GO:0006979	response to oxidative stress	12/162	400/18870	0.000180234	0.021401075	0.017362858	TNFAIP3/STAT6/NAGLU/EDN1/FOS/MDM2/ERCC6L2/CD36/MAPK9/MAPK3/BCL2/IL6	12
HS/CON	BP	GO:0021915	neural tube development	6/121	162/18870	0.000622042	0.021607786	0.017739407	TGFB2/MARCKS/BMP7/WNT5A/PRICKLE1/SEMA3C	6
HS/REC	BP	GO:0090022	regulation of neutrophil chemotaxis	4/162	34/18870	0.000198575	0.021759695	0.017653809	CCR7/EDN1/C3AR1/NCKAP1L	4
HS/REC	BP	GO:1902624	positive regulation of neutrophil migration	4/162	34/18870	0.000198575	0.021759695	0.017653809	CCR7/EDN1/C3AR1/NCKAP1L	4
HS/REC	BP	GO:0030225	macrophage differentiation	5/162	64/18870	0.00022213	0.021759695	0.017653809	IL1RL1/NAGLU/IL31RA/LIF/INHBA	5
HS/REC	BP	GO:0097696	receptor signaling pathway via STAT	8/162	188/18870	0.000223751	0.021759695	0.017653809	STAT6/NAGLU/MIR19A/TYK2/IL31RA/DOT1L/LIF/IL6	8
HS/REC	BP	GO:0046942	carboxylic acid transport	11/162	351/18870	0.000231389	0.021759695	0.017653809	CRABP2/EDN1/SFXN2/CD36/SFXN4/SLC25A12/ATP8B1/SFXN3/SLC43A3/MFSD2A/MFSD12	11
HS/REC	BP	GO:0042542	response to hydrogen peroxide	6/162	101/18870	0.000236075	0.021759695	0.017653809	TNFAIP3/STAT6/EDN1/MDM2/BCL2/IL6	6
HS/REC	BP	GO:0015849	organic acid transport	11/162	352/18870	0.000237108	0.021759695	0.017653809	CRABP2/EDN1/SFXN2/CD36/SFXN4/SLC25A12/ATP8B1/SFXN3/SLC43A3/MFSD2A/MFSD12	11
HS/REC	BP	GO:0032434	regulation of proteasomal ubiquitin-dependent protein catabolic process	7/162	143/18870	0.000237551	0.021759695	0.017653809	MDM2/ARAF/PLK1/USP5/MAPK9/SH3RF2/PRICKLE1	7
HS/REC	BP	GO:0061307	cardiac neural crest cell differentiation involved in heart development	3/162	15/18870	0.00026197	0.022490001	0.018246312	EDN1/MAPK3/SEMA3C	3
HS/REC	BP	GO:0061308	cardiac neural crest cell development involved in heart development	3/162	15/18870	0.00026197	0.022490001	0.018246312	EDN1/MAPK3/SEMA3C	3
HS/REC	BP	GO:0002822	regulation of adaptive immune response based on somatic recombination of immune receptors built from immunoglobulin superfamily domains	8/162	193/18870	0.000267318	0.022490001	0.018246312	TNFAIP3/IL1RL1/MR1/STAT6/CD274/TYK2/NCKAP1L/IL6	8
HS/REC	BP	GO:0070301	cellular response to hydrogen peroxide	5/162	67/18870	0.000275606	0.022490001	0.018246312	TNFAIP3/STAT6/EDN1/MDM2/IL6	5
HS/REC	BP	GO:0010038	response to metal ion	11/162	359/18870	0.000280599	0.022490001	0.018246312	MCOLN1/ANK3/VCAM1/EDN1/FOS/MDM2/MAPK9/SLC25A12/MAPK3/HNRNPD/BCL2	11
HS/CON	BP	GO:0006939	smooth muscle contraction	5/121	109/18870	0.000685111	0.023435061	0.01923955	MYLK/ITGA2/MYH11/ACTA2/NMU	5
HS/CON	BP	GO:0071241	cellular response to inorganic substance	7/121	229/18870	0.0006924	0.023435061	0.01923955	MAPK3/WNT5A/HNRNPD/SLC39A8/MT1A/MT2A/FABP4	7
HS/REC	MF	GO:0015245	fatty acid transmembrane transporter activity	3/167	10/18496	8.28064E-05	0.023630196	0.021852771	CD36/SLC43A3/MFSD2A	3
HS/REC	MF	GO:0140326	ATPase-coupled intramembrane lipid transporter activity	4/167	27/18496	9.56688E-05	0.023630196	0.021852771	ABCA1/ATP9B/ATP8B1/MFSD2A	4
HS/REC	BP	GO:0002687	positive regulation of leukocyte migration	7/162	149/18870	0.000305325	0.023874938	0.019369922	ICAM1/CCR7/EDN1/C3AR1/NCKAP1L/MAPK3/IL6	7
HS/CON	MF	GO:0008083	growth factor activity	6/118	162/18496	0.000604468	0.024303721	0.022524044	TGFB2/BMP7/LIF/GDF5/INHBA/IL6	6
HS/CON	MF	GO:0022804	active transmembrane transporter activity	10/118	454/18496	0.000660427	0.024303721	0.022524044	CYC1/SLC17A9/SLC6A6/SLC39A8/SLC36A4/SLC35E3/MFSD2A/MFSD12/SLC7A11/SLC25A21	10
HS/CON	BP	GO:0042634	regulation of hair cycle	3/121	28/18870	0.00074943	0.025044228	0.020560632	TGFB2/WNT5A/FST	3
HS/CON	BP	GO:0034250	positive regulation of amide metabolic process	6/121	169/18870	0.000776272	0.025616991	0.021030855	ITGA2/GSAP/HNRNPD/PRMT1/RBM3/IL6	6
HS/REC	BP	GO:0043434	response to peptide hormone	12/162	430/18870	0.000348389	0.025693893	0.020845654	OTOP1/STAT6/PLCB1/PTPRE/TYK2/EDN1/FOS/MDM2/EGR1/CYC1/MAPK3/INHBA	12
HS/REC	BP	GO:0071375	cellular response to peptide hormone stimulus	10/162	310/18870	0.000355233	0.025693893	0.020845654	OTOP1/STAT6/PLCB1/PTPRE/TYK2/EDN1/FOS/MDM2/MAPK3/INHBA	10
HS/REC	BP	GO:0097305	response to alcohol	9/162	254/18870	0.000355962	0.025693893	0.020845654	A2M/ABCA1/VCAM1/CCR7/FOS/MDM2/HNRNPD/PTGDR/INHBA	9
HS/REC	BP	GO:0001819	positive regulation of cytokine production	13/162	499/18870	0.00038228	0.025693893	0.020845654	IL1RL1/TRAF3/PLCB1/CD274/TYK2/CCR7/TMEM106A/EGR1/CD36/C3AR1/FERMT1/MAPK9/IL6	13
HS/REC	BP	GO:0002764	immune response-regulating signaling pathway	13/162	500/18870	0.000389605	0.025693893	0.020845654	TNFAIP3/TRAF3/NAGLU/MIR19A/PLD2/KHDRBS1/CCR7/CD36/C3AR1/NCKAP1L/MAPKAPK3/MAPK9/BCL2	13
HS/REC	BP	GO:0032611	interleukin-1 beta production	6/162	111/18870	0.000393131	0.025693893	0.020845654	TNFAIP3/CCR7/TMEM106A/EGR1/CD36/IL6	6
HS/REC	BP	GO:0032651	regulation of interleukin-1 beta production	6/162	111/18870	0.000393131	0.025693893	0.020845654	TNFAIP3/CCR7/TMEM106A/EGR1/CD36/IL6	6
HS/REC	BP	GO:0071347	cellular response to interleukin-1	6/162	111/18870	0.000393131	0.025693893	0.020845654	PLCB1/EDN1/EGR1/ANKRD1/MAPK3/IL6	6
HS/REC	BP	GO:0050777	negative regulation of immune response	8/162	205/18870	0.000400716	0.025693893	0.020845654	A2M/TNFAIP3/OTOP1/IL1RL1/STAT6/MIR19A/NCKAP1L/PLK2	8
HS/CON	BP	GO:0030166	proteoglycan biosynthetic process	4/121	65/18870	0.000805319	0.025927339	0.021285643	FOXL1/CHSY3/GLCE/HS3ST3A1	4
HS/CON	BP	GO:1905330	regulation of morphogenesis of an epithelium	4/121	65/18870	0.000805319	0.025927339	0.021285643	BMP7/WNT5A/GREM1/LIF	4
HS/CON	BP	GO:0072012	glomerulus vasculature development	3/121	29/18870	0.000832008	0.02637852	0.02165605	BMP7/ACTA2/ANGPT2	3
HS/CON	BP	GO:0018958	phenol-containing compound metabolic process	5/121	114/18870	0.000839317	0.02637852	0.02165605	TGFB2/WNT5A/BCL2/MFSD12/SLC7A11	5
HS/REC	BP	GO:2000060	positive regulation of ubiquitin-dependent protein catabolic process	6/162	113/18870	0.000432555	0.027191577	0.022060737	MDM2/PLK1/USP5/MAPK9/SH3RF2/PRICKLE1	6
HS/REC	BP	GO:0002819	regulation of adaptive immune response	8/162	208/18870	0.000441375	0.027212437	0.022077661	TNFAIP3/IL1RL1/MR1/STAT6/CD274/TYK2/NCKAP1L/IL6	8
HS/REC	BP	GO:0045732	positive regulation of protein catabolic process	8/162	210/18870	0.000470297	0.028448522	0.023080506	TNFAIP3/MDM2/PLK1/USP5/PLK2/MAPK9/SH3RF2/PRICKLE1	8
HS/CON	BP	GO:0001654	eye development	9/121	391/18870	0.000942883	0.028478101	0.023379748	TGFB2/BMP7/WNT5A/PRICKLE1/FJX1/MFSD2A/BCL2/INHBA/SLC7A11	9
HS/CON	BP	GO:0042303	molting cycle	5/121	117/18870	0.000943459	0.028478101	0.023379748	TGFB2/WNT5A/BCL2/INHBA/FST	5
HS/CON	BP	GO:0042633	hair cycle	5/121	117/18870	0.000943459	0.028478101	0.023379748	TGFB2/WNT5A/BCL2/INHBA/FST	5
HS/CON	BP	GO:0019226	transmission of nerve impulse	4/121	69/18870	0.001008259	0.028478101	0.023379748	ITGA2/CACNG4/GRIK2/S1PR1	4
HS/CON	BP	GO:0032835	glomerulus development	4/121	69/18870	0.001008259	0.028478101	0.023379748	BMP7/BCL2/ACTA2/ANGPT2	4
HS/CON	BP	GO:0050918	positive chemotaxis	4/121	69/18870	0.001008259	0.028478101	0.023379748	ITGA2/WNT5A/ANGPT2/S1PR1	4
HS/CON	BP	GO:0150063	visual system development	9/121	395/18870	0.001012528	0.028478101	0.023379748	TGFB2/BMP7/WNT5A/PRICKLE1/FJX1/MFSD2A/BCL2/INHBA/SLC7A11	9
HS/CON	BP	GO:0033688	regulation of osteoblast proliferation	3/121	31/18870	0.001013993	0.028478101	0.023379748	GREM1/BCL2/MIR138-1	3
HS/CON	BP	GO:0061437	renal system vasculature development	3/121	31/18870	0.001013993	0.028478101	0.023379748	BMP7/ACTA2/ANGPT2	3
HS/CON	BP	GO:0061440	kidney vasculature development	3/121	31/18870	0.001013993	0.028478101	0.023379748	BMP7/ACTA2/ANGPT2	3
HS/CON	BP	GO:0050922	negative regulation of chemotaxis	4/121	70/18870	0.001064119	0.029571315	0.024277248	WNT5A/GREM1/ANGPT2/SEMA3C	4
HS/CON	BP	GO:0030510	regulation of BMP signaling pathway	5/121	121/18870	0.001096906	0.029994192	0.024624419	WNT5A/GREM1/GDF5/PRMT1/FST	5
HS/CON	BP	GO:0007435	salivary gland morphogenesis	3/121	32/18870	0.001113681	0.029994192	0.024624419	TGFB2/BMP7/SEMA3C	3
HS/CON	BP	GO:0061384	heart trabecula morphogenesis	3/121	32/18870	0.001113681	0.029994192	0.024624419	TGFB2/BMP7/S1PR1	3
HS/CON	BP	GO:0048880	sensory system development	9/121	401/18870	0.001124782	0.029994192	0.024624419	TGFB2/BMP7/WNT5A/PRICKLE1/FJX1/MFSD2A/BCL2/INHBA/SLC7A11	9
HS/REC	BP	GO:1901800	positive regulation of proteasomal protein catabolic process	6/162	117/18870	0.000520618	0.030909298	0.025076953	MDM2/PLK1/USP5/MAPK9/SH3RF2/PRICKLE1	6
HS/CON	BP	GO:0060411	cardiac septum morphogenesis	4/121	72/18870	0.001182298	0.031212674	0.025624759	TGFB2/BMP7/WNT5A/SEMA3C	4
HS/CON	BP	GO:0031069	hair follicle morphogenesis	3/121	33/18870	0.001219349	0.031872095	0.026166126	TGFB2/BCL2/FST	3
HS/CON	BP	GO:0001838	embryonic epithelial tube formation	5/121	125/18870	0.001268131	0.032722522	0.026864303	TGFB2/BMP7/WNT5A/GREM1/PRICKLE1	5
HS/CON	BP	GO:0060415	muscle tissue morphogenesis	4/121	74/18870	0.00130938	0.032722522	0.026864303	TGFB2/MYLK/WNT5A/S1PR1	4
HS/CON	BP	GO:0061138	morphogenesis of a branching epithelium	6/121	187/18870	0.001309496	0.032722522	0.026864303	BMP7/WNT5A/GREM1/BCL2/HS3ST3A1/SEMA3C	6
HS/CON	BP	GO:0007178	transmembrane receptor protein serine/threonine kinase signaling pathway	9/121	410/18870	0.001311897	0.032722522	0.026864303	TGFB2/BMP7/MAPK3/WNT5A/GREM1/GDF5/PRMT1/INHBA/FST	9
HS/CON	BP	GO:2000027	regulation of animal organ morphogenesis	5/121	126/18870	0.001313859	0.032722522	0.026864303	BMP7/WNT5A/GREM1/PRICKLE1/TNFRSF11B	5
HS/CON	BP	GO:0007431	salivary gland development	3/121	34/18870	0.00133113	0.032842826	0.02696307	TGFB2/BMP7/SEMA3C	3
HS/REC	BP	GO:0007519	skeletal muscle tissue development	7/162	166/18870	0.000584843	0.033482252	0.02716441	FOS/MAFF/GTF3C5/EGR1/NLN/ANKRD1/BCL2	7
HS/REC	BP	GO:0070665	positive regulation of leukocyte proliferation	7/162	166/18870	0.000584843	0.033482252	0.02716441	VCAM1/CD274/TYK2/NCKAP1L/MAPK3/BCL2/IL6	7
HS/CON	BP	GO:0001707	mesoderm formation	4/121	75/18870	0.001376369	0.033488125	0.027492843	BMP7/ITGA2/WNT5A/INHBA	4
HS/CON	BP	GO:0030308	negative regulation of cell growth	6/121	189/18870	0.001382654	0.033488125	0.027492843	TGFB2/WNT5A/GREM1/BCL2/INHBA/SEMA3C	6
HS/CON	BP	GO:0098773	skin epidermis development	5/121	128/18870	0.001408954	0.033814895	0.027761112	TGFB2/WNT5A/BCL2/INHBA/FST	5
HS/CON	BP	GO:0071695	anatomical structure maturation	7/121	261/18870	0.001479606	0.035190635	0.028890557	TGFB2/WNT5A/GREM1/RAC3/BCL2/S1PR1/DEFB1	7
HS/REC	BP	GO:0002437	inflammatory response to antigenic stimulus	5/162	80/18870	0.00062734	0.035285147	0.028627113	A2M/MIR19A/CCR7/IL31RA/PLK2	5
HS/REC	BP	GO:1902622	regulation of neutrophil migration	4/162	46/18870	0.000645034	0.035654808	0.028927022	CCR7/EDN1/C3AR1/NCKAP1L	4
HS/CON	BP	GO:0048332	mesoderm morphogenesis	4/121	77/18870	0.00151746	0.035768705	0.029365137	BMP7/ITGA2/WNT5A/INHBA	4
HS/CON	BP	GO:0006882	intracellular zinc ion homeostasis	3/121	36/18870	0.001573544	0.036439969	0.029916226	SLC39A8/MT1A/MT2A	3
HS/CON	BP	GO:0033687	osteoblast proliferation	3/121	36/18870	0.001573544	0.036439969	0.029916226	GREM1/BCL2/MIR138-1	3
HS/CON	BP	GO:0042476	odontogenesis	5/121	132/18870	0.001614251	0.0370576	0.030423284	TGFB2/BMP7/TNFRSF11B/INHBA/FST	5
HS/REC	BP	GO:0042113	B cell activation	9/162	278/18870	0.000682569	0.037090123	0.030091504	TNFAIP3/STAT6/VCAM1/MIR19A/HHEX/NCKAP1L/BCL2/INHBA/IL6	9
HS/REC	BP	GO:0001952	regulation of cell-matrix adhesion	6/162	125/18870	0.000738167	0.037822936	0.030686041	DLC1/CCR7/DAPK3/CD36/FERMT1/BCL2	6
HS/REC	BP	GO:0002688	regulation of leukocyte chemotaxis	6/162	126/18870	0.000769657	0.037822936	0.030686041	CCR7/EDN1/C3AR1/NCKAP1L/MAPK3/IL6	6
HS/REC	BP	GO:0007259	receptor signaling pathway via JAK-STAT	7/162	174/18870	0.000772656	0.037822936	0.030686041	STAT6/MIR19A/TYK2/IL31RA/DOT1L/LIF/IL6	7

Cluster	ONTOLOGY	ID	Description	GeneRatio	BgRatio	pvalue	p.adjust	qvalue	geneID	Count
HS/REC	BP	GO:0002757	immune response-activating signaling pathway	12/162	473/18870	0.000808702	0.037822936	0.030686041	TNFAIP3/TRAF3/NAGLU/MIR19A/PLD2/KHDRBS1/CCR7/CD36/C3AR1/NCKAP1L/MAPKAPK3/BCL2	12
HS/REC	BP	GO:0071900	regulation of protein serine/threonine kinase activity	9/162	285/18870	0.000814199	0.037822936	0.030686041	SPRY4/EDN1/PLK1/GTPBP4/HHEX/CEMIP/CCNB3/MAPRE3/DIRAS3	9
HS/REC	BP	GO:0140694	non-membrane-bounded organelle assembly	11/162	408/18870	0.000815873	0.037822936	0.030686041	CENPE/WDR62/CENPH/EDN1/PLK1/CIRBP/MAPRE3/PLK2/MAPK9/ANKRD1/PRICKLE1	11
HS/REC	BP	GO:0006911	phagocytosis, engulfment	4/162	49/18870	0.000820954	0.037822936	0.030686041	ABCA1/HAVCR1/CD36/NCKAP1L	4
HS/REC	BP	GO:0033628	regulation of cell adhesion mediated by integrin	4/162	49/18870	0.000820954	0.037822936	0.030686041	IFT74/NCKAP1L/FERMT1/LIF	4
HS/REC	BP	GO:0043410	positive regulation of MAPK cascade	12/162	474/18870	0.000823639	0.037822936	0.030686041	ICAM1/PLCB1/CCR7/EDN1/TMEM106A/CD36/SH3RF2/MAPK3/LIF/PRMT1/INHBA/IL6	12
HS/REC	BP	GO:0002768	immune response-regulating cell surface receptor signaling pathway	10/162	346/18870	0.000832453	0.037822936	0.030686041	TNFAIP3/NAGLU/MIR19A/PLD2/KHDRBS1/CCR7/C3AR1/NCKAP1L/MAPK9/BCL2	10
HS/REC	BP	GO:0002685	regulation of leukocyte migration	8/162	230/18870	0.000853319	0.037822936	0.030686041	ICAM1/PLCB1/CCR7/EDN1/C3AR1/NCKAP1L/MAPK3/IL6	8
HS/REC	BP	GO:0055013	cardiac muscle cell development	5/162	86/18870	0.000872121	0.037822936	0.030686041	NAGLU/MIR19A/SORBS2/EDN1/PRICKLE1	5
HS/REC	BP	GO:0060538	skeletal muscle organ development	7/162	178/18870	0.000882938	0.037822936	0.030686041	FOS/MAFF/GTF3C5/EGR1/NLN/ANKRD1/BCL2	7
HS/REC	BP	GO:2000058	regulation of ubiquitin-dependent protein catabolic process	7/162	178/18870	0.000882938	0.037822936	0.030686041	MDM2/ARAF/PLK1/USP5/MAPK9/SH3RF2/PRICKLE1	7
HS/REC	BP	GO:0032612	interleukin-1 production	6/162	130/18870	0.000906131	0.037822936	0.030686041	TNFAIP3/CCR7/TMEM106A/EGR1/CD36/IL6	6
HS/REC	BP	GO:0032652	regulation of interleukin-1 production	6/162	130/18870	0.000906131	0.037822936	0.030686041	TNFAIP3/CCR7/TMEM106A/EGR1/CD36/IL6	6
HS/REC	BP	GO:0050864	regulation of B cell activation	6/162	130/18870	0.000906131	0.037822936	0.030686041	TNFAIP3/STAT6/NCKAP1L/BCL2/INHBA/IL6	6
HS/REC	BP	GO:0070661	leukocyte proliferation	10/162	350/18870	0.000908411	0.037822936	0.030686041	TNFAIP3/STAT6/VCAM1/CD274/TYK2/HHEX/NCKAP1L/MAPK3/BCL2/IL6	10
HS/CON	BP	GO:0072088	nephron epithelium morphogenesis	4/121	79/18870	0.001668327	0.03796882	0.031171372	GREM1/LIF/BCL2/HS3ST3A1	4
HS/CON	BP	GO:0043010	camera-type eye development	8/121	344/18870	0.001698029	0.038132957	0.031306124	TGFB2/BMP7/WNT5A/PRICKLE1/FJX1/MFSD2A/INHBA/SLC7A11	8
HS/CON	BP	GO:0048730	epidermis morphogenesis	3/121	37/18870	0.001704428	0.038132957	0.031306124	TGFB2/BCL2/FST	3
HS/REC	BP	GO:1903131	mononuclear cell differentiation	12/162	481/18870	0.000934801	0.038422699	0.031172633	MR1/STAT6/VCAM1/CCR7/IL31RA/FOS/EGR1/HHEX/NCKAP1L/BCL2/INHBA/IL6	12
HS/REC	BP	GO:0033627	cell adhesion mediated by integrin	5/162	88/18870	0.000967664	0.038779141	0.031461817	ICAM1/IFT74/NCKAP1L/FERMT1/LIF	5
HS/REC	BP	GO:0048864	stem cell development	5/162	88/18870	0.000967664	0.038779141	0.031461817	EDN1/MSI2/MAPK3/BCL2/SEMA3C	5
HS/CON	BP	GO:0003253	cardiac neural crest cell migration involved in outflow tract morphogenesis	2/121	10/18870	0.00177437	0.039036143	0.032047615	BMP7/SEMA3C	2
HS/CON	BP	GO:0046085	adenosine metabolic process	2/121	10/18870	0.00177437	0.039036143	0.032047615	PTGDR/NT5C2	2
HS/REC	BP	GO:0007517	muscle organ development	10/162	354/18870	0.000989978	0.039183571	0.031789934	NAGLU/FOS/MAFF/GTF3C5/EGR1/NLN/ANKRD1/MYLK/LIF/BCL2	10
HS/CON	BP	GO:0032496	response to lipopolysaccharide	8/121	348/18870	0.001825371	0.039225427	0.032203012	MAPK3/GBP2/WNT5A/IL18BP/PCK2/IL36A/THBD/IL6	8
HS/CON	BP	GO:0042440	pigment metabolic process	4/121	81/18870	0.001829315	0.039225427	0.032203012	WNT5A/BCL2/MFSD12/SLC7A11	4
HS/CON	BP	GO:0032965	regulation of collagen biosynthetic process	3/121	38/18870	0.001841923	0.039225427	0.032203012	ITGA2/INHBA/IL6	3
HS/CON	BP	GO:0071248	cellular response to metal ion	6/121	200/18870	0.001842406	0.039225427	0.032203012	MAPK3/WNT5A/SLC39A8/MT1A/MT2A/FABP4	6
HS/REC	BP	GO:0061041	regulation of wound healing	6/162	133/18870	0.001020169	0.039718462	0.032223896	TNFAIP3/EDN1/CD36/FERMT1/MYLK/THBD	6
HS/REC	BP	GO:0045332	phospholipid translocation	4/162	52/18870	0.00102827	0.039718462	0.032223896	ABCA1/ATP9B/ATP8B1/MFSD2A	4
HS/CON	BP	GO:0072175	epithelial tube formation	5/121	137/18870	0.001900823	0.040145372	0.032958262	TGFB2/BMP7/WNT5A/GREM1/PRICKLE1	5
HS/REC	BP	GO:0007160	cell-matrix adhesion	8/162	239/18870	0.0010925	0.041187669	0.033415875	VCAM1/DLC1/CCR7/DAPK3/THSD1/CD36/FERMT1/BCL2	8
HS/REC	BP	GO:0071219	cellular response to molecule of bacterial origin	8/162	239/18870	0.0010925	0.041187669	0.033415875	ABCA1/TNFAIP3/MIR19A/CD274/CD36/ANKRD1/MAPK3/IL6	8
HS/REC	BP	GO:0045058	T cell selection	4/162	53/18870	0.001104847	0.041187669	0.033415875	STAT6/CCR7/BCL2/IL6	4
HS/CON	BP	GO:0001763	morphogenesis of a branching structure	6/121	203/18870	0.001985859	0.041608465	0.034159422	BMP7/WNT5A/GREM1/BCL2/HS3ST3A1/SEMA3C	6
HS/CON	BP	GO:0090596	sensory organ morphogenesis	7/121	277/18870	0.002073581	0.041762372	0.034285775	BMP7/MAPK3/WNT5A/FJX1/MFSD2A/BCL2/CLRN2	7
HS/CON	BP	GO:0060395	SMAD protein signal transduction	4/121	84/18870	0.002090529	0.041762372	0.034285775	TGFB2/BMP7/GDF5/INHBA	4
HS/CON	BP	GO:0033028	myeloid cell apoptotic process	3/121	40/18870	0.002137216	0.041762372	0.034285775	BCL2/SLC7A11/IL6	3
HS/CON	BP	GO:0045727	positive regulation of translation	5/121	141/18870	0.002155588	0.041762372	0.034285775	ITGA2/HNRNPD/PRMT1/RBM3/IL6	5
HS/CON	BP	GO:0003337	mesenchymal to epithelial transition involved in metanephros morphogenesis	2/121	11/18870	0.002159592	0.041762372	0.034285775	GREM1/LIF	2
HS/CON	BP	GO:0007494	midgut development	2/121	11/18870	0.002159592	0.041762372	0.034285775	WNT5A/FOXL1	2
HS/CON	BP	GO:0044848	biological phase	2/121	11/18870	0.002159592	0.041762372	0.034285775	TGFB2/WNT5A	2
HS/CON	BP	GO:0060601	lateral sprouting from an epithelium	2/121	11/18870	0.002159592	0.041762372	0.034285775	BMP7/WNT5A	2
HS/CON	BP	GO:0090009	primitive streak formation	2/121	11/18870	0.002159592	0.041762372	0.034285775	WNT5A/PRICKLE1	2
HS/CON	BP	GO:2001054	negative regulation of mesenchymal cell apoptotic process	2/121	11/18870	0.002159592	0.041762372	0.034285775	BMP7/GDF5	2
HS/CON	BP	GO:0034103	regulation of tissue remodeling	4/121	85/18870	0.002183033	0.041762372	0.034285775	GREM1/TNFRSF11B/S1PR1/IL6	4
HS/CON	BP	GO:1901264	carbohydrate derivative transport	4/121	85/18870	0.002183033	0.041762372	0.034285775	SLC17A9/SLC50A1/SLC35E3/MFSD2A	4
HS/REC	BP	GO:1903320	regulation of protein modification by small protein conjugation or removal	8/162	243/18870	0.001214696	0.044079623	0.035762139	TNFAIP3/COPS3/PLK1/GTPBP4/EGR1/MAPK9/SH3RF2/PRICKLE1	8
HS/REC	BP	GO:0001942	hair follicle development	5/162	93/18870	0.001240439	0.044079623	0.035762139	NAGLU/FERMT1/BCL2/INHBA/FST	5
HS/REC	BP	GO:0002363	alpha-beta T cell lineage commitment	3/162	25/18870	0.00124339	0.044079623	0.035762139	STAT6/BCL2/IL6	3
HS/REC	BP	GO:0035994	response to muscle stretch	3/162	25/18870	0.00124339	0.044079623	0.035762139	EDN1/FOS/ANKRD1	3
HS/REC	BP	GO:0042770	signal transduction in response to DNA damage	7/162	189/18870	0.001251168	0.044079623	0.035762139	COPS3/MDM2/PLK1/DOT1L/PLK2/ANKRD1/MAPK3	7
HS/REC	BP	GO:1902074	response to salt	10/162	366/18870	0.001271469	0.0441624	0.035829297	MCOLN1/PLCB1/EDN1/FOS/MDM2/EGR1/CD36/SLC25A12/HNRNPD/CACNG4	10
HS/REC	BP	GO:1903052	positive regulation of proteolysis involved in protein catabolic process	6/162	139/18870	0.001281068	0.0441624	0.035829297	MDM2/PLK1/USP5/MAPK9/SH3RF2/PRICKLE1	6
HS/REC	BP	GO:0051146	striated muscle cell differentiation	9/162	305/18870	0.001308014	0.044320731	0.035957752	NAGLU/MIR19A/SORBS2/EDN1/NLN/ANKRD1/PRICKLE1/BCL2/MYEF2	9
HS/REC	BP	GO:0048863	stem cell differentiation	8/162	246/18870	0.001313309	0.044320731	0.035957752	A2M/EDN1/MSI2/MAPK3/PRICKLE1/LIF/BCL2/SEMA3C	8
HS/REC	BP	GO:0070555	response to interleukin-1	6/162	140/18870	0.001329091	0.044353558	0.035984385	PLCB1/EDN1/EGR1/ANKRD1/MAPK3/IL6	6
HS/REC	BP	GO:0009410	response to xenobiotic stimulus	11/162	434/18870	0.001341951	0.044353558	0.035984385	ABCA1/EDN1/FOS/TFAP2B/MDM2/NCKAP1L/DUSP6/ANKRD1/PRICKLE1/BCL2/SEMA3C	11
HS/REC	BP	GO:0002438	acute inflammatory response to antigenic stimulus	3/162	26/18870	0.001396763	0.045232546	0.036697515	A2M/CCR7/IL31RA	3
HS/REC	BP	GO:0043369	CD4-positive or CD8-positive, alpha-beta T cell lineage commitment	3/162	26/18870	0.001396763	0.045232546	0.036697515	STAT6/BCL2/IL6	3
HS/REC	BP	GO:0022404	molting cycle process	5/162	96/18870	0.001429202	0.045366555	0.036806238	NAGLU/FERMT1/BCL2/INHBA/FST	5
HS/REC	BP	GO:0022405	hair cycle process	5/162	96/18870	0.001429202	0.045366555	0.036806238	NAGLU/FERMT1/BCL2/INHBA/FST	5
HS/REC	BP	GO:0034204	lipid translocation	4/162	57/18870	0.001451347	0.045617819	0.03701009	ABCA1/ATP9B/ATP8B1/MFSD2A	4
HS/CON	BP	GO:0061448	connective tissue development	7/121	285/18870	0.002432508	0.045737274	0.037549062	BMP7/MAPK3/WNT5A/GREM1/GDF5/ACTA2/MIR138-1	7
HS/CON	BP	GO:0050921	positive regulation of chemotaxis	5/121	145/18870	0.002434417	0.045737274	0.037549062	MAPK3/ITGA2/WNT5A/S1PR1/IL6	5
HS/CON	BP	GO:0045124	regulation of bone resorption	3/121	42/18870	0.002460324	0.045737274	0.037549062	TNFRSF11B/S1PR1/IL6	3
HS/CON	BP	GO:0033627	cell adhesion mediated by integrin	4/121	88/18870	0.002477436	0.045737274	0.037549062	TGFB2/ITGA2/RAC3/LIF	4
HS/CON	BP	GO:0048864	stem cell development	4/121	88/18870	0.002477436	0.045737274	0.037549062	BMP7/MAPK3/BCL2/SEMA3C	4
HS/REC	BP	GO:0046883	regulation of hormone secretion	8/162	251/18870	0.001491748	0.046160094	0.037450042	PLCB1/EDN1/TFAP2B/CDK16/LIF/PFKFB2/INHBA/IL6	8
HS/REC	BP	GO:0055001	muscle cell development	7/162	195/18870	0.001497395	0.046160094	0.037450042	NAGLU/MIR19A/SORBS2/EDN1/ANKRD1/PRICKLE1/BCL2	7
HS/REC	BP	GO:0061136	regulation of proteasomal protein catabolic process	7/162	196/18870	0.001541868	0.04634643	0.037601218	MDM2/ARAF/PLK1/USP5/MAPK9/SH3RF2/PRICKLE1	7
HS/REC	BP	GO:0015804	neutral amino acid transport	4/162	58/18870	0.001548548	0.04634643	0.037601218	SFXN2/SLC25A12/SFXN3/MFSD12	4
HS/REC	BP	GO:0099024	plasma membrane invagination	4/162	58/18870	0.001548548	0.04634643	0.037601218	ABCA1/HAVCR1/CD36/NCKAP1L	4
HS/REC	BP	GO:0015711	organic anion transport	11/162	443/18870	0.001578922	0.04634643	0.037601218	CRABP2/EDN1/SFXN2/CD36/SFXN4/SLC25A12/ATP8B1/SFXN3/SLC43A3/MFSD2A/MFSD12	11
HS/REC	BP	GO:0050671	positive regulation of lymphocyte proliferation	6/162	145/18870	0.001590177	0.04634643	0.037601218	VCAM1/CD274/TYK2/NCKAP1L/BCL2/IL6	6
HS/REC	BP	GO:0050921	positive regulation of chemotaxis	6/162	145/18870	0.001590177	0.04634643	0.037601218	CCR7/EDN1/C3AR1/NCKAP1L/MAPK3/IL6	6
HS/CON	BP	GO:0000101	sulfur amino acid transport	2/121	12/18870	0.002580662	0.046346581	0.038049286	MFSD12/SLC7A11	2
HS/CON	BP	GO:0060379	cardiac muscle cell myoblast differentiation	2/121	12/18870	0.002580662	0.046346581	0.038049286	GREM1/PRICKLE1	2
HS/CON	BP	GO:0061309	cardiac neural crest cell development involved in outflow tract morphogenesis	2/121	12/18870	0.002580662	0.046346581	0.038049286	BMP7/SEMA3C	2
HS/CON	BP	GO:1902337	regulation of apoptotic process involved in morphogenesis	2/121	12/18870	0.002580662	0.046346581	0.038049286	TGFB2/BMP7	2
HS/CON	BP	GO:0002237	response to molecule of bacterial origin	8/121	369/18870	0.002623115	0.046644327	0.038293728	MAPK3/GBP2/WNT5A/IL18BP/PCK2/IL36A/THBD/IL6	8
HS/CON	BP	GO:0010712	regulation of collagen metabolic process	3/121	43/18870	0.002632578	0.046644327	0.038293728	ITGA2/INHBA/IL6	3
HS/CON	BP	GO:0006029	proteoglycan metabolic process	4/121	90/18870	0.002688199	0.047312311	0.038842124	FOXL1/CHSY3/GLCE/HS3ST3A1	4
HS/REC	BP	GO:0002696	positive regulation of leukocyte activation	10/162	380/18870	0.001679176	0.048499431	0.039347964	IL1RL1/STAT6/VCAM1/HAVCR1/CD274/TYK2/CCR7/NCKAP1L/BCL2/IL6	10
HS/CON	BP	GO:0006865	amino acid transport	5/121	150/18870	0.00281876	0.048896848	0.040142987	SFXN3/SLC6A6/SLC36A4/MFSD12/SLC7A11	5
HS/CON	BP	GO:0072073	kidney epithelium development	5/121	150/18870	0.00281876	0.048896848	0.040142987	BMP7/GREM1/LIF/BCL2/HS3ST3A1	5
HS/CON	BP	GO:0042594	response to starvation	6/121	218/18870	0.002833795	0.048896848	0.040142987	MAPK3/DAPL1/TBC1D5/MFSD2A/BCL2/PCK2	6
HS/REC	BP	GO:0001573	ganglioside metabolic process	3/162	28/18870	0.001737947	0.04919205	0.039909891	ST3GAL5/NAGLU/CLN6	3
HS/REC	BP	GO:0002429	immune response-activating cell surface receptor signaling pathway	9/162	318/18870	0.001742055	0.04919205	0.039909891	TNFAIP3/NAGLU/MIR19A/PLD2/KHDRBS1/CCR7/C3AR1/NCKAP1L/BCL2	9
HS/REC	BP	GO:1990542	mitochondrial transmembrane transport	4/162	60/18870	0.001756377	0.04919205	0.039909891	SFXN2/SFXN4/SLC25A12/SFXN3	4
HS/REC	BP	GO:0032946	positive regulation of mononuclear cell proliferation	6/162	148/18870	0.001764531	0.04919205	0.039909891	VCAM1/CD274/TYK2/NCKAP1L/BCL2/IL6	6
HS/CON	BP	GO:0035148	tube formation	5/121	151/18870	0.002900605	0.049724651	0.040822591	TGFB2/BMP7/WNT5A/GREM1/PRICKLE1	5
