# Supplementary material for: Establishment and characterization of Hanwoo cumulus cell line for heat stress studies
Source: Anim Biosci. 2026 Jun 15;39(7):250896. doi: 10.5713/ab.250896 (PMC13353149; doi:10.5713/ab.250896)
Supplement: Supplementary file 11 [file ab-250896-Supplementary-11.pdf]

Supplement 11. Cumulus upregulated DEP - GO enrichment (BP, MF)

Cluster	ONTOLOGY	ID	Description	GeneRatio	BgRatio	pvalue	p.adjust	qvalue	geneID	Count
HS/CON	BP	GO:0042026	protein refolding	8/35	27/18870	1.28108E-16	1.20166E-13	8.8732E-14	CRYAB/DNAJA1/DNAJA4/DNAJB2/HSP90AA1/HSPA1L/HSPA6/HSPB1	8
HS/CON	BP	GO:0006986	response to unfolded protein	10/35	137/18870	4.58788E-14	2.15171E-11	1.58885E-11	BAG3/DNAJA1/DNAJB1/DNAJB2/HSP90AA1/HSPA1L/HSPA6/HSPB1/HSPB8/HSPH1	10
HS/CON	BP	GO:0006457	protein folding	11/35	223/18870	1.5949E-13	3.89636E-11	2.87712E-11	BAG3/CRYAB/DNAJA1/DNAJA4/DNAJB1/DNAJB2/HSP90AA1/HSPA1L/HSPA6/HSPB1/HSPH1	11
HS/CON	BP	GO:0009408	response to heat	9/35	103/18870	1.88954E-13	3.89636E-11	2.87712E-11	BAG3/CRYAB/DNAJA1/DNAJA4/DNAJB1/HSP90AA1/HSPA6/HSPB1/IRAK1	9
HS/CON	BP	GO:0035966	response to topologically incorrect protein	10/35	159/18870	2.07695E-13	3.89636E-11	2.87712E-11	BAG3/DNAJA1/DNAJB1/DNAJB2/HSP90AA1/HSPA1L/HSPA6/HSPB1/HSPB8/HSPH1	10
HS/CON	MF	GO:0051082	unfolded protein binding	9/36	122/18496	1.4199E-12	1.68968E-10	1.10603E-10	CRYAB/DNAJA1/DNAJA4/DNAJB1/DNAJB2/HSP90AA1/HSPA1L/HSPA6/HSPB1	9
HS/CON	BP	GO:0009266	response to temperature stimulus	9/35	171/18870	1.92363E-11	3.00727E-09	2.22061E-09	BAG3/CRYAB/DNAJA1/DNAJA4/DNAJB1/HSP90AA1/HSPA6/HSPB1/IRAK1	9
HS/CON	MF	GO:0044183	protein folding chaperone	6/36	66/18496	2.92984E-09	1.74325E-07	1.1411E-07	DNAJB1/HSP90AA1/HSPA1L/HSPA6/HSPB1/HSPH1	6
HS/CON	MF	GO:0031072	heat shock protein binding	7/36	128/18496	4.5521E-09	1.80567E-07	1.18195E-07	DNAJA1/DNAJA4/DNAJB1/DNAJB2/HSPA1L/HSPA6/IRAK1	7
HS/CON	BP	GO:0061077	chaperone-mediated protein folding	6/35	75/18870	4.76258E-09	6.38185E-07	4.71245E-07	DNAJB1/DNAJB2/HSPA1L/HSPA6/HSPB1/HSPH1	6
HS/CON	MF	GO:0060590	ATPase regulator activity	5/36	49/18496	3.74942E-08	1.11545E-06	7.30151E-07	BAG3/DNAJA1/DNAJB1/DNAJB2/HSPH1	5
REC/CON	BP	GO:0006986	response to unfolded protein	7/38	137/18870	9.53174E-09	5.84749E-06	4.844E-06	BAG3/DNAJB1/HSPA1L/HSPA6/HSPB1/HSPB8/HSPH1	7
REC/CON	BP	GO:0006457	protein folding	8/38	223/18870	1.20941E-08	5.84749E-06	4.844E-06	BAG3/CRYAB/DNAJB1/BAG4/HSPA1L/HSPA6/HSPB1/HSPH1	8
REC/CON	BP	GO:0035966	response to topologically incorrect protein	7/38	159/18870	2.67673E-08	8.62799E-06	7.14733E-06	BAG3/DNAJB1/HSPA1L/HSPA6/HSPB1/HSPB8/HSPH1	7
HS/CON	BP	GO:0070841	inclusion body assembly	4/35	24/18870	1.02614E-07	1.20315E-05	8.88422E-06	BAG3/DNAJA4/DNAJB1/DNAJB2	4
REC/CON	BP	GO:0009408	response to heat	6/38	103/18870	5.47024E-08	1.32243E-05	1.09549E-05	BAG3/CRYAB/DNAJB1/HSPA6/HSPB1/IRAK1	6
HS/CON	BP	GO:0034605	cellular response to heat	5/35	66/18870	1.34274E-07	1.39944E-05	1.03336E-05	BAG3/DNAJB1/HSP90AA1/HSPA6/IRAK1	5
HS/CON	MF	GO:0140662	ATP-dependent protein folding chaperone	4/36	40/18496	1.0506E-06	2.50043E-05	1.63672E-05	HSP90AA1/HSPA1L/HSPA6/HSPH1	4
REC/CON	MF	GO:0044183	protein folding chaperone	5/38	66/18496	2.27215E-07	2.90835E-05	2.10473E-05	DNAJB1/HSPA1L/HSPA6/HSPB1/HSPH1	5
HS/CON	BP	GO:0051085	chaperone cofactor-dependent protein refolding	4/35	32/18870	3.43627E-07	3.22323E-05	2.38007E-05	DNAJB1/HSPA1L/HSPA6/HSPH1	4
REC/CON	BP	GO:0042026	protein refolding	4/38	27/18870	2.37287E-07	4.58914E-05	3.80159E-05	CRYAB/HSPA1L/HSPA6/HSPB1	4
HS/REC	BP	GO:0006953	acute-phase response	5/39	48/18870	4.63769E-08	5.24523E-05	4.32526E-05	A2M/SERPINA1/ORM1/SERPINF2/IL6	5
HS/CON	BP	GO:0051084	'de novo' post-translational protein folding	4/35	37/18870	6.26979E-07	5.34642E-05	3.94787E-05	DNAJB1/HSPA1L/HSPA6/HSPH1	4
HS/CON	MF	GO:0030544	Hsp70 protein binding	4/36	51/18496	2.82937E-06	5.61159E-05	3.67322E-05	DNAJA1/DNAJA4/DNAJB1/DNAJB2	4
REC/CON	BP	GO:0061077	chaperone-mediated protein folding	5/38	75/18870	3.92534E-07	6.32635E-05	5.24068E-05	DNAJB1/HSPA1L/HSPA6/HSPB1/HSPH1	5
REC/CON	BP	GO:0051085	chaperone cofactor-dependent protein refolding	4/38	32/18870	4.82709E-07	6.66828E-05	5.52393E-05	DNAJB1/HSPA1L/HSPA6/HSPH1	4
HS/CON	BP	GO:0006458	'de novo' protein folding	4/35	41/18870	9.56333E-07	7.47534E-05	5.51989E-05	DNAJB1/HSPA1L/HSPA6/HSPH1	4
HS/CON	BP	GO:0090084	negative regulation of inclusion body assembly	3/35	12/18870	1.27134E-06	9.17323E-05	6.77363E-05	DNAJA4/DNAJB1/DNAJB2	3
HS/CON	MF	GO:0051087	protein-folding chaperone binding	5/36	133/18496	5.61904E-06	9.55238E-05	6.25277E-05	BAG3/DNAJA1/DNAJA4/DNAJB1/DNAJB2	5
REC/CON	BP	GO:0051084	'de novo' post-translational protein folding	4/38	37/18870	8.80185E-07	0.000106392	8.81343E-05	DNAJB1/HSPA1L/HSPA6/HSPH1	4
REC/CON	BP	GO:0009266	response to temperature stimulus	6/38	171/18870	1.10102E-06	0.000118298	9.79972E-05	BAG3/CRYAB/DNAJB1/HSPA6/HSPB1/IRAK1	6
REC/CON	BP	GO:0006458	'de novo' protein folding	4/38	41/18870	1.34187E-06	0.000129759	0.000107491	DNAJB1/HSPA1L/HSPA6/HSPH1	4
REC/CON	MF	GO:0060590	ATPase regulator activity	4/38	49/18496	3.00269E-06	0.000152695	0.000110503	BAG3/DNAJB1/BAG4/HSPH1	4
REC/CON	MF	GO:0000774	adenyl-nucleotide exchange factor activity	3/38	15/18496	3.57878E-06	0.000152695	0.000110503	BAG3/BAG4/HSPH1	3
REC/CON	MF	GO:0051082	unfolded protein binding	5/38	122/18496	4.8486E-06	0.000155155	0.000112283	CRYAB/DNAJB1/HSPA1L/HSPA6/HSPB1	5
HS/CON	BP	GO:0090083	regulation of inclusion body assembly	3/35	17/18870	3.90469E-06	0.000261614	0.000193179	DNAJA4/DNAJB1/DNAJB2	3
HS/CON	MF	GO:0001671	ATPase activator activity	3/36	27/18496	1.91805E-05	0.000285311	0.000186758	DNAJA1/DNAJB1/DNAJB2	3
HS/CON	MF	GO:0044389	ubiquitin-like protein ligase binding	6/36	327/18496	3.63191E-05	0.000480219	0.000314341	AMBRA1/CEBPB/DNAJA1/HSP90AA1/HSPA1L/HSPA6	6
REC/CON	BP	GO:0034605	cellular response to heat	4/38	66/18870	9.21178E-06	0.0008098	0.000670829	BAG3/DNAJB1/HSPA6/IRAK1	4
HS/CON	MF	GO:0042826	histone deacetylase binding	4/36	127/18496	0.000105329	0.001253414	0.000820457	AKAP8L/CEBPB/GMNN/HSP90AA1	4
HS/REC	BP	GO:0002526	acute inflammatory response	5/39	107/18870	2.63495E-06	0.001490063	0.001228718	A2M/SERPINA1/ORM1/SERPINF2/IL6	5
REC/CON	MF	GO:0000146	microfilament motor activity	3/38	37/18496	5.92365E-05	0.001516454	0.001097434	MYH1/MYH7/MYO5C	3
REC/CON	MF	GO:0140662	ATP-dependent protein folding chaperone	3/38	40/18496	7.50029E-05	0.001600061	0.001157939	HSPA1L/HSPA6/HSPH1	3
HS/REC	MF	GO:0004866	endopeptidase inhibitor activity	5/39	168/18496	2.61205E-05	0.001682452	0.001317626	A2M/SERPINA1/SERPINF2/FETUB/SERPINA7	5
HS/REC	MF	GO:0030414	peptidase inhibitor activity	5/39	175/18496	3.17698E-05	0.001682452	0.001317626	A2M/SERPINA1/SERPINF2/FETUB/SERPINA7	5
HS/REC	MF	GO:0061135	endopeptidase regulator activity	5/39	184/18496	4.03788E-05	0.001682452	0.001317626	A2M/SERPINA1/SERPINF2/FETUB/SERPINA7	5
HS/REC	MF	GO:0004867	serine-type endopeptidase inhibitor activity	4/39	101/18496	5.94808E-05	0.001858776	0.001455715	A2M/SERPINA1/SERPINF2/SERPINA7	4
HS/CON	BP	GO:0032386	regulation of intracellular transport	6/35	336/18870	3.19954E-05	0.002000778	0.001477401	AKAP8L/BAG3/CRYAB/FEZ1/HSP90AA1/HSPA1L	6
REC/CON	MF	GO:0031072	heat shock protein binding	4/38	128/18496	0.000134612	0.002461476	0.001781332	DNAJB1/HSPA1L/HSPA6/IRAK1	4
HS/CON	BP	GO:0010506	regulation of autophagy	6/35	355/18870	4.35037E-05	0.002550407	0.001883254	AMBRA1/BAG3/CCNY/FEZ1/HSPB1/HSPB8	6
HS/REC	MF	GO:0061134	peptidase regulator activity	5/39	228/18496	0.000111444	0.002786102	0.002181958	A2M/SERPINA1/SERPINF2/FETUB/SERPINA7	5
HS/REC	MF	GO:0004857	enzyme inhibitor activity	6/39	386/18496	0.000144779	0.003016225	0.002362181	A2M/SERPINA1/SERPINF2/CDKN2A/FETUB/SERPINA7	6
HS/CON	MF	GO:0031625	ubiquitin protein ligase binding	5/36	308/18496	0.000305958	0.003309905	0.00216659	AMBRA1/DNAJA1/HSP90AA1/HSPA1L/HSPA6	5
HS/CON	MF	GO:0000774	adenyl-nucleotide exchange factor activity	2/36	15/18496	0.000380635	0.003774628	0.002470787	BAG3/HSPH1	2
HS/CON	BP	GO:0010658	striated muscle cell apoptotic process	3/35	56/18870	0.000151475	0.008357874	0.006171565	AMBRA1/BAG3/HSP90AA1	3
HS/CON	BP	GO:0010508	positive regulation of autophagy	4/35	150/18870	0.000165796	0.008639792	0.006379736	AMBRA1/BAG3/CCNY/HSPB8	4
HS/CON	BP	GO:0016241	regulation of macroautophagy	4/35	159/18870	0.00020733	0.010235576	0.007558084	AMBRA1/BAG3/FEZ1/HSPB8	4
HS/CON	MF	GO:0023026	MHC class II protein complex binding	2/36	27/18496	0.001253858	0.011477625	0.007512997	HSP90AA1/PKM	2
HS/CON	BP	GO:0098840	protein transport along microtubule	2/35	14/18870	0.00029991	0.01339598	0.009891769	BAG3/HSPB1	2
HS/CON	BP	GO:0099118	microtubule-based protein transport	2/35	14/18870	0.00029991	0.01339598	0.009891769	BAG3/HSPB1	2
HS/CON	BP	GO:0061684	chaperone-mediated autophagy	2/35	15/18870	0.000345647	0.014406953	0.010638284	BAG3/HSP90AA1	2
HS/CON	BP	GO:0016239	positive regulation of macroautophagy	3/35	75/18870	0.000360196	0.014406953	0.010638284	AMBRA1/BAG3/HSPB8	3
HS/CON	BP	GO:0033674	positive regulation of kinase activity	5/35	337/18870	0.000368621	0.014406953	0.010638284	AMBRA1/CCNY/EREG/HSP90AA1/IRAK1	5
REC/CON	MF	GO:0015377	chloride:monoatomic cation symporter activity	2/38	22/18496	0.000925122	0.014801955	0.010711941	SLC12A6/SLC12A9	2
REC/CON	MF	GO:0015296	monoatomic anion:monoatomic cation symporter activity	2/38	25/18496	0.001196798	0.017021128	0.012317921	SLC12A6/SLC12A9	2
HS/CON	BP	GO:0031396	regulation of protein ubiquitination	4/35	200/18870	0.000495712	0.018222969	0.013456081	DNAJA1/DNAJB2/HSP90AA1/TRIM44	4
HS/CON	BP	GO:0032388	positive regulation of intracellular transport	4/35	201/18870	0.000505114	0.018222969	0.013456081	BAG3/FEZ1/HSP90AA1/HSPA1L	4
HS/CON	BP	GO:0007076	mitotic chromosome condensation	2/35	19/18870	0.000560294	0.019465032	0.014373237	AKAP8L/CDCA5	2
HS/CON	MF	GO:0023023	MHC protein complex binding	2/36	37/18496	0.002350214	0.019976817	0.013076377	HSP90AA1/PKM	2
HS/CON	BP	GO:0010657	muscle cell apoptotic process	3/35	90/18870	0.000614858	0.01998628	0.014758133	AMBRA1/BAG3/HSP90AA1	3
HS/CON	BP	GO:0032273	positive regulation of protein polymerization	3/35	91/18870	0.000635013	0.01998628	0.014758133	AMBRA1/HSP90AA1/OCLN	3
HS/CON	BP	GO:0099111	microtubule-based transport	4/35	214/18870	0.00063922	0.01998628	0.014758133	BAG3/FEZ1/HSPB1/KATNIP	4
REC/CON	MF	GO:0003774	cytoskeletal motor activity	3/38	112/18496	0.001562638	0.02000177	0.014474965	MYH1/MYH7/MYO5C	3
HS/CON	BP	GO:0050821	protein stabilization	4/35	217/18870	0.000673435	0.020376826	0.015046517	BAG3/CRYAB/HSP90AA1/TRIM44	4
HS/CON	BP	GO:0061912	selective autophagy	3/35	96/18870	0.000742128	0.021753614	0.016063156	AMBRA1/BAG3/HSPB8	3
HS/CON	BP	GO:0009651	response to salt stress	2/35	24/18870	0.000899085	0.025555811	0.018870748	HSP90AA1/SLC12A6	2
HS/CON	BP	GO:0051347	positive regulation of transferase activity	5/35	414/18870	0.00093633	0.025831699	0.019074468	AMBRA1/CCNY/EREG/HSP90AA1/IRAK1	5
REC/CON	BP	GO:0098840	protein transport along microtubule	2/38	14/18870	0.000353897	0.026324502	0.02180694	BAG3/HSPB1	2
REC/CON	BP	GO:0099118	microtubule-based protein transport	2/38	14/18870	0.000353897	0.026324502	0.02180694	BAG3/HSPB1	2
HS/CON	BP	GO:0007018	microtubule-based movement	5/35	420/18870	0.000998535	0.026746813	0.0197502	BAG3/DNAJA1/FEZ1/HSPB1/KATNIP	5
HS/CON	BP	GO:1903320	regulation of protein modification by small protein conjugation or removal	4/35	243/18870	0.00102653	0.026746813	0.0197502	DNAJA1/DNAJB2/HSP90AA1/TRIM44	4
HS/CON	BP	GO:0046822	regulation of nucleocytoplasmic transport	3/35	109/18870	0.001072593	0.027191692	0.020078704	AKAP8L/BAG3/HSP90AA1	3
HS/CON	BP	GO:0000045	autophagosome assembly	3/35	114/18870	0.00122082	0.02947814	0.021767048	AMBRA1/BAG3/FEZ1	3
HS/CON	BP	GO:0010592	positive regulation of lamellipodium assembly	2/35	28/18870	0.001225637	0.02947814	0.021767048	HSP90AA1/OCLN	2
REC/CON	MF	GO:0051087	protein-folding chaperone binding	3/38	133/18496	0.002551235	0.029687103	0.021484088	BAG3/DNAJB1/BAG4	3
HS/CON	BP	GO:1905037	autophagosome organization	3/35	121/18870	0.001449153	0.033982627	0.02509322	AMBRA1/BAG3/FEZ1	3
HS/CON	MF	GO:0005080	protein kinase C binding	2/36	51/18496	0.004423053	0.035089555	0.022968838	FEZ1/HSPB1	2
HS/CON	BP	GO:0072655	establishment of protein localization to mitochondrion	3/35	124/18870	0.001554668	0.035567766	0.026263707	DNAJA1/HSP90AA1/HSPA1L	3
HS/CON	BP	GO:0002230	positive regulation of defense response to virus by host	2/35	32/18870	0.001600777	0.035750697	0.026398787	HSP90AA1/TRIM44	2
HS/CON	BP	GO:0010664	negative regulation of striated muscle cell apoptotic process	2/35	33/18870	0.001702073	0.0362851	0.026793397	AMBRA1/BAG3	2
HS/CON	BP	GO:0043618	regulation of transcription from RNA polymerase II promoter in response to stress	2/35	33/18870	0.001702073	0.0362851	0.026793397	BAG3/CEBPB	2
HS/CON	BP	GO:0045860	positive regulation of protein kinase activity	4/35	282/18870	0.001775169	0.036353753	0.026844091	CCNY/EREG/HSP90AA1/IRAK1	4
HS/CON	BP	GO:0070585	protein localization to mitochondrion	3/35	131/18870	0.001819312	0.036353753	0.026844091	DNAJA1/HSP90AA1/HSPA1L	3
HS/CON	BP	GO:1901873	regulation of post-translational protein modification	4/35	284/18870	0.001821563	0.036353753	0.026844091	DNAJA1/DNAJB2/HSP90AA1/TRIM44	4
REC/CON	MF	GO:0031625	ubiquitin protein ligase binding	4/38	308/18496	0.003563822	0.0380141	0.027510204	BAG4/HSPA1L/HSPA6/BID	4
HS/CON	BP	GO:1902745	positive regulation of lamellipodium organization	2/35	36/18870	0.002023811	0.039548641	0.029203238	HSP90AA1/OCLN	2
HS/CON	BP	GO:0001819	positive regulation of cytokine production	5/35	499/18870	0.002136442	0.040897597	0.030199325	CEBPB/EREG/HSP90AA1/HSPB1/IRAK1	5
REC/CON	BP	GO:0031334	positive regulation of protein-containing complex assembly	4/38	198/18870	0.000656549	0.04152528	0.034399105	LGALS3/BAG4/BID/OCLN	4
REC/CON	BP	GO:0055064	chloride ion homeostasis	2/38	19/18870	0.000660804	0.04152528	0.034399105	SLC12A6/SLC12A9	2
REC/CON	BP	GO:0008625	extrinsic apoptotic signaling pathway via death domain receptors	3/38	86/18870	0.000687078	0.04152528	0.034399105	BAG3/LGALS3/BID	3
HS/CON	BP	GO:1900180	regulation of protein localization to nucleus	3/35	143/18870	0.002335354	0.042789063	0.031596009	BAG3/DCLK1/HSP90AA1	3
HS/CON	BP	GO:0021955	central nervous system neuron axonogenesis	2/35	39/18870	0.002372102	0.042789063	0.031596009	DCLK1/HSP90AA1	2
HS/CON	BP	GO:0043620	regulation of DNA-templated transcription in response to stress	2/35	39/18870	0.002372102	0.042789063	0.031596009	BAG3/CEBPB	2
REC/CON	MF	GO:0044389	ubiquitin-like protein ligase binding	4/38	327/18496	0.004408934	0.043411039	0.031415883	BAG4/HSPA1L/HSPA6/BID	4
HS/CON	BP	GO:0042307	positive regulation of protein import into nucleus	2/35	40/18870	0.002494049	0.044139964	0.032593532	BAG3/HSP90AA1	2
HS/CON	BP	GO:0010591	regulation of lamellipodium assembly	2/35	41/18870	0.002618904	0.044664224	0.032980652	HSP90AA1/OCLN	2
HS/CON	BP	GO:0032781	positive regulation of ATP-dependent activity	2/35	41/18870	0.002618904	0.044664224	0.032980652	DNAJB1/DNAJB2	2
REC/CON	BP	GO:0055081	monoatomic anion homeostasis	2/38	21/18870	0.000809455	0.046043683	0.038142103	SLC12A6/SLC12A9	2
HS/REC	MF	GO:0000146	microfilament motor activity	2/39	37/18496	0.002753893	0.049176654	0.038513085	MYH1/MYH7	2
HS/CON	BP	GO:0031647	regulation of protein stability	4/35	325/18870	0.00296859	0.049546319	0.036585656	BAG3/CRYAB/HSP90AA1/TRIM44	4
HS/CON	BP	GO:0050691	regulation of defense response to virus by host	2/35	44/18870	0.00301081	0.049546319	0.036585656	HSP90AA1/TRIM44	2
