# Supplementary material for: Establishment and characterization of Hanwoo cumulus cell line for heat stress studies
Source: Anim Biosci. 2026 Jun 15;39(7):250896. doi: 10.5713/ab.250896 (PMC13353149; doi:10.5713/ab.250896)
Supplement: Supplementary file 12 [file ab-250896-Supplementary-12.pdf]

Supplement 12. Cumulus downregulated DEP - GO enrichment (BP, MF)

Cluster	ONTOLOGY	ID	Description	GeneRatio	BgRatio	pvalue	p.adjust	qvalue	geneID	Count
REC/CON	MF	GO:0005201	extracellular matrix structural constituent	12/143	166/18496	6.30204E-09	2.20571E-06	2.00339E-06	COL1A1/COL12A1/COL1A2/COL6A2/COL5A1/CCN1/LUM/IGFBP7/COL11A1/BGN/FGB/VTN	12
HS/REC	BP	GO:0030199	collagen fibril organization	6/49	65/18870	1.64258E-08	2.05979E-05	1.60973E-05	COL1A1/COL1A2/COL5A1/LUM/COL11A1/SFRP2	6
REC/CON	BP	GO:0042060	wound healing	17/136	423/18870	1.06342E-08	2.77659E-05	2.36527E-05	COL5A1/APOE/F2/BLOC1S4/CCN1/PLAUR/FGB/ERBB2/GGCX/JMJD1C/MACF1/CPB2/SERPINE1/TNFRSF12A/TPM1/VTN/WNT5A	17
REC/CON	BP	GO:0042730	fibrinolysis	6/136	27/18870	3.27872E-08	3.7677E-05	3.20956E-05	F2/PLAUR/FGB/CPB2/SERPINE1/VTN	6
REC/CON	BP	GO:0030195	negative regulation of blood coagulation	7/136	48/18870	4.97719E-08	3.7677E-05	3.20956E-05	APOE/F2/PLAUR/FGB/CPB2/SERPINE1/VTN	7
REC/CON	BP	GO:1900047	negative regulation of hemostasis	7/136	49/18870	5.77204E-08	3.7677E-05	3.20956E-05	APOE/F2/PLAUR/FGB/CPB2/SERPINE1/VTN	7
REC/CON	BP	GO:0050819	negative regulation of coagulation	7/136	52/18870	8.82951E-08	4.61077E-05	3.92774E-05	APOE/F2/PLAUR/FGB/CPB2/SERPINE1/VTN	7
REC/CON	BP	GO:0051917	regulation of fibrinolysis	5/136	18/18870	1.4352E-07	6.2455E-05	5.3203E-05	F2/PLAUR/CPB2/SERPINE1/VTN	5
REC/CON	MF	GO:0005539	glycosaminoglycan binding	12/143	240/18496	3.67209E-07	6.42616E-05	5.83669E-05	COL5A1/APOE/F2/CCDC80/FSTL1/CCN1/CCN2/COL11A1/BGN/FGFR1/HYAL2/VTN	12
REC/CON	MF	GO:0008201	heparin binding	10/143	174/18496	1.01885E-06	8.55092E-05	7.76655E-05	COL5A1/APOE/F2/CCDC80/FSTL1/CCN1/CCN2/COL11A1/FGFR1/VTN	10
REC/CON	MF	GO:0019838	growth factor binding	9/143	135/18496	1.04916E-06	8.55092E-05	7.76655E-05	COL1A1/COL1A2/COL5A1/CCN1/CCN2/IGFBP7/ERBB2/FGFR1/HYAL2	9
REC/CON	MF	GO:0030020	extracellular matrix structural constituent conferring tensile strength	6/143	45/18496	1.22156E-06	8.55092E-05	7.76655E-05	COL1A1/COL12A1/COL1A2/COL6A2/COL5A1/COL11A1	6
REC/CON	MF	GO:1901681	sulfur compound binding	12/143	275/18496	1.54928E-06	9.03746E-05	8.20846E-05	COL5A1/APOE/RRM2/F2/FST/CCDC80/FSTL1/CCN1/CCN2/COL11A1/FGFR1/VTN	12
REC/CON	BP	GO:0030199	collagen fibril organization	7/136	65/18870	4.25072E-07	0.000158552	0.000135064	COL1A1/COL12A1/COL1A2/COL5A1/LUM/COL11A1/SFRP2	7
REC/CON	BP	GO:0030193	regulation of blood coagulation	7/136	68/18870	5.81382E-07	0.000189748	0.000161639	APOE/F2/PLAUR/FGB/CPB2/SERPINE1/VTN	7
REC/CON	BP	GO:1900046	regulation of hemostasis	7/136	70/18870	7.10362E-07	0.000206084	0.000175555	APOE/F2/PLAUR/FGB/CPB2/SERPINE1/VTN	7
HS/CON	BP	GO:0042060	wound healing	14/110	423/18870	1.69475E-07	0.000238386	0.000215236	APOE/BLOC1S4/CCN1/FGA/FGB/GGCX/JMJD1C/MACF1/PLAU/SERPIND1/SERPINE1/TPM1/VTN/WNT5A	14
HS/CON	BP	GO:0030195	negative regulation of blood coagulation	6/110	48/18870	3.43898E-07	0.000238386	0.000215236	APOE/FGA/FGB/PLAU/SERPINE1/VTN	6
HS/CON	BP	GO:1900047	negative regulation of hemostasis	6/110	49/18870	3.90036E-07	0.000238386	0.000215236	APOE/FGA/FGB/PLAU/SERPINE1/VTN	6
HS/CON	BP	GO:0042730	fibrinolysis	5/110	27/18870	4.47673E-07	0.000238386	0.000215236	FGA/FGB/PLAU/SERPINE1/VTN	5
HS/CON	BP	GO:0050819	negative regulation of coagulation	6/110	52/18870	5.59846E-07	0.000238495	0.000215335	APOE/FGA/FGB/PLAU/SERPINE1/VTN	6
REC/CON	BP	GO:0050818	regulation of coagulation	7/136	73/18870	9.48337E-07	0.000247611	0.00021093	APOE/F2/PLAUR/FGB/CPB2/SERPINE1/VTN	7
HS/CON	BP	GO:0007596	blood coagulation	10/110	224/18870	7.57004E-07	0.000266713	0.000240813	APOE/BLOC1S4/FGA/FGB/GGCX/JMJD1C/PLAU/SERPIND1/SERPINE1/VTN	10
HS/CON	BP	GO:0050817	coagulation	10/110	229/18870	9.25616E-07	0.000266713	0.000240813	APOE/BLOC1S4/FGA/FGB/GGCX/JMJD1C/PLAU/SERPIND1/SERPINE1/VTN	10
HS/CON	BP	GO:0007599	hemostasis	10/110	231/18870	1.00174E-06	0.000266713	0.000240813	APOE/BLOC1S4/FGA/FGB/GGCX/JMJD1C/PLAU/SERPIND1/SERPINE1/VTN	10
REC/CON	BP	GO:0061045	negative regulation of wound healing	7/136	75/18870	1.1415E-06	0.000270951	0.000230813	APOE/F2/PLAUR/FGB/CPB2/SERPINE1/VTN	7
REC/CON	BP	GO:0051918	negative regulation of fibrinolysis	4/136	13/18870	1.75494E-06	0.000381846	0.00032528	F2/CPB2/SERPINE1/VTN	4
HS/REC	MF	GO:0048407	platelet-derived growth factor binding	3/55	11/18496	4.03665E-06	0.000477704	0.000421851	COL1A1/COL1A2/COL5A1	3
HS/REC	MF	GO:0030020	extracellular matrix structural constituent conferring tensile strength	4/55	45/18496	9.52236E-06	0.000477704	0.000421851	COL1A1/COL1A2/COL5A1/COL11A1	4
HS/REC	MF	GO:0005201	extracellular matrix structural constituent	6/55	166/18496	9.6182E-06	0.000477704	0.000421851	COL1A1/COL1A2/COL5A1/SPARC/LUM/COL11A1	6
HS/CON	BP	GO:0030193	regulation of blood coagulation	6/110	68/18870	2.79084E-06	0.000660499	0.000596359	APOE/FGA/FGB/PLAU/SERPINE1/VTN	6
HS/CON	BP	GO:1900046	regulation of hemostasis	6/110	70/18870	3.31178E-06	0.00067541	0.000609822	APOE/FGA/FGB/PLAU/SERPINE1/VTN	6
HS/CON	BP	GO:0045807	positive regulation of endocytosis	8/110	155/18870	3.48803E-06	0.00067541	0.000609822	AHSG/APOA2/APOE/CCL2/CD47/SERPINE1/VTN/WNT5A	8
REC/CON	BP	GO:0030198	extracellular matrix organization	12/136	321/18870	3.68916E-06	0.000684352	0.000582973	COL1A1/COL12A1/COL1A2/COL5A1/CCDC80/CCN1/LUM/COL11A1/SFRP2/SLC39A8/CPB2/VTN	12
REC/CON	BP	GO:0043062	extracellular structure organization	12/136	322/18870	3.80866E-06	0.000684352	0.000582973	COL1A1/COL12A1/COL1A2/COL5A1/CCDC80/CCN1/LUM/COL11A1/SFRP2/SLC39A8/CPB2/VTN	12
REC/CON	BP	GO:0045229	external encapsulating structure organization	12/136	323/18870	3.93155E-06	0.000684352	0.000582973	COL1A1/COL12A1/COL1A2/COL5A1/CCDC80/CCN1/LUM/COL11A1/SFRP2/SLC39A8/CPB2/VTN	12
HS/CON	BP	GO:0050818	regulation of coagulation	6/110	73/18870	4.2393E-06	0.000752477	0.000679404	APOE/FGA/FGB/PLAU/SERPINE1/VTN	6
HS/CON	BP	GO:0061045	negative regulation of wound healing	6/110	75/18870	4.96742E-06	0.000813893	0.000734856	APOE/FGA/FGB/PLAU/SERPINE1/VTN	6
REC/CON	BP	GO:0007596	blood coagulation	10/136	224/18870	5.24743E-06	0.000836229	0.000712351	APOE/F2/BLOC1S4/PLAUR/FGB/GGCX/JMJD1C/CPB2/SERPINE1/VTN	10
REC/CON	BP	GO:0061041	regulation of wound healing	8/136	133/18870	5.44461E-06	0.000836229	0.000712351	APOE/F2/PLAUR/FGB/CPB2/SERPINE1/TNFRSF12A/VTN	8
HS/CON	BP	GO:0010810	regulation of cell-substrate adhesion	9/110	220/18870	5.66232E-06	0.000861482	0.000777824	CCDC80/CCN1/CDKN2A/FGA/FGB/MACF1/PLAU/SERPINE1/VTN	9
REC/CON	BP	GO:0050817	coagulation	10/136	229/18870	6.37642E-06	0.000890181	0.000758311	APOE/F2/BLOC1S4/PLAUR/FGB/GGCX/JMJD1C/CPB2/SERPINE1/VTN	10
REC/CON	BP	GO:1903035	negative regulation of response to wounding	7/136	97/18870	6.47776E-06	0.000890181	0.000758311	APOE/F2/PLAUR/FGB/CPB2/SERPINE1/VTN	7
REC/CON	BP	GO:0007599	hemostasis	10/136	231/18870	6.88367E-06	0.000898663	0.000765537	APOE/F2/BLOC1S4/PLAUR/FGB/GGCX/JMJD1C/CPB2/SERPINE1/VTN	10
HS/CON	BP	GO:0031589	cell-substrate adhesion	11/110	356/18870	7.43882E-06	0.001056313	0.000953735	CCDC80/CCN1/CCN2/CDKN2A/FGA/FGB/MACF1/MSLN/PLAU/SERPINE1/VTN	11
REC/CON	BP	GO:0045785	positive regulation of cell adhesion	14/136	482/18870	1.03056E-05	0.001281326	0.001091512	CCDC80/CCL2/CCN1/PLAUR/CD47/FGB/ERBB2/ADAM19/SOX4/SFRP2/NR4A3/TPM1/VTN/WNT5A	14
HS/CON	BP	GO:0007160	cell-matrix adhesion	9/110	239/18870	1.10345E-05	0.001468967	0.001326317	CCN2/CDKN2A/FGA/FGB/MACF1/MSLN/PLAU/SERPINE1/VTN	9
REC/CON	BP	GO:0050878	regulation of body fluid levels	12/136	365/18870	1.35144E-05	0.001603915	0.001366313	APOE/F2/BLOC1S4/PLAUR/FGB/GGCX/CCND1/JMJD1C/CPB2/SERPINE1/HYAL2/VTN	12
HS/REC	MF	GO:0019838	growth factor binding	5/55	135/18496	4.99121E-05	0.001859227	0.001641847	COL1A1/COL1A2/COL5A1/ERBB2/IGFBP4	5
REC/CON	BP	GO:0045807	positive regulation of endocytosis	8/136	155/18870	1.67386E-05	0.001900196	0.001618704	APOA2/APOE/CCL2/CD47/AHI1/SERPINE1/VTN/WNT5A	8
REC/CON	BP	GO:2001236	regulation of extrinsic apoptotic signaling pathway	8/136	158/18870	1.92318E-05	0.002031369	0.001730446	FGB/INHBA/SFRP2/FGFR1/SERPINE1/TNFRSF12A/TIMP3/HYAL2	8
REC/CON	BP	GO:0051216	cartilage development	9/136	207/18870	1.94501E-05	0.002031369	0.001730446	COL1A1/CCN1/CCN2/COL11A1/BGN/ZEB1/SFRP2/HYAL2/WNT5A	9
HS/CON	BP	GO:0031639	plasminogen activation	4/110	27/18870	1.72992E-05	0.002167494	0.001957011	FGA/FGB/PLAU/SERPINE1	4
HS/CON	BP	GO:1903035	negative regulation of response to wounding	6/110	97/18870	2.19755E-05	0.002600438	0.002347912	APOE/FGA/FGB/PLAU/SERPINE1/VTN	6
REC/CON	BP	GO:0035987	endodermal cell differentiation	5/136	49/18870	2.6699E-05	0.002681198	0.002284009	COL12A1/COL5A1/COL11A1/INHBA/VTN	5
REC/CON	BP	GO:1903034	regulation of response to wounding	8/136	173/18870	3.68825E-05	0.003447203	0.002936539	APOE/F2/PLAUR/FGB/CPB2/SERPINE1/TNFRSF12A/VTN	8
REC/CON	BP	GO:0050678	regulation of epithelial cell proliferation	12/136	407/18870	3.93908E-05	0.003447203	0.002936539	APOE/CCL2/ERBB2/ZEB1/CCND1/CYP7B1/SFRP2/FGFR1/CPB2/NR4A3/UHRF1/WNT5A	12
REC/CON	BP	GO:0001704	formation of primary germ layer	7/136	128/18870	3.96153E-05	0.003447203	0.002936539	COL12A1/COL5A1/COL11A1/INHBA/SFRP2/VTN/WNT5A	7
REC/CON	BP	GO:0031639	plasminogen activation	4/136	27/18870	3.98298E-05	0.003447203	0.002936539	PLAUR/FGB/CPB2/SERPINE1	4
REC/CON	BP	GO:0061448	connective tissue development	10/136	285/18870	4.20783E-05	0.003447203	0.002936539	COL1A1/COL5A1/CCN1/CCN2/COL11A1/BGN/ZEB1/SFRP2/HYAL2/WNT5A	10
REC/CON	BP	GO:0097191	extrinsic apoptotic signaling pathway	9/136	229/18870	4.29871E-05	0.003447203	0.002936539	CASP8AP2/FGB/INHBA/SFRP2/FGFR1/SERPINE1/TNFRSF12A/TIMP3/HYAL2	9
REC/CON	BP	GO:0050673	epithelial cell proliferation	13/136	480/18870	4.46971E-05	0.003447203	0.002936539	APOE/FST/CCL2/ERBB2/ZEB1/CCND1/CYP7B1/SFRP2/FGFR1/CPB2/NR4A3/UHRF1/WNT5A	13
REC/CON	BP	GO:0030194	positive regulation of blood coagulation	4/136	28/18870	4.62092E-05	0.003447203	0.002936539	F2/CPB2/SERPINE1/VTN	4
REC/CON	BP	GO:1900048	positive regulation of hemostasis	4/136	28/18870	4.62092E-05	0.003447203	0.002936539	F2/CPB2/SERPINE1/VTN	4
REC/CON	MF	GO:0048407	platelet-derived growth factor binding	3/143	11/18496	7.13518E-05	0.00356759	0.003240338	COL1A1/COL1A2/COL5A1	3
REC/CON	BP	GO:0001706	endoderm formation	5/136	58/18870	6.09104E-05	0.004316054	0.00367668	COL12A1/COL5A1/COL11A1/INHBA/VTN	5
REC/CON	BP	GO:0050820	positive regulation of coagulation	4/136	30/18870	6.1162E-05	0.004316054	0.00367668	F2/CPB2/SERPINE1/VTN	4
HS/REC	BP	GO:1901991	negative regulation of mitotic cell cycle phase transition	6/49	196/18870	1.1217E-05	0.004689688	0.003664987	CCL2/ZFP36L1/SPDL1/WEE1/CLSPN/FBXO5	6
HS/REC	BP	GO:0030198	extracellular matrix organization	7/49	321/18870	1.79662E-05	0.004689688	0.003664987	COL1A1/COL1A2/COL5A1/MMP2/LUM/COL11A1/SFRP2	7
HS/REC	BP	GO:0043062	extracellular structure organization	7/49	322/18870	1.83295E-05	0.004689688	0.003664987	COL1A1/COL1A2/COL5A1/MMP2/LUM/COL11A1/SFRP2	7
HS/REC	BP	GO:0045229	external encapsulating structure organization	7/49	323/18870	1.86989E-05	0.004689688	0.003664987	COL1A1/COL1A2/COL5A1/MMP2/LUM/COL11A1/SFRP2	7
HS/REC	BP	GO:0044772	mitotic cell cycle phase transition	8/49	470/18870	2.56671E-05	0.005364426	0.004192295	CCL2/CCNY/PHF10/ZFP36L1/SPDL1/WEE1/CLSPN/FBXO5	8
HS/CON	BP	GO:0030100	regulation of endocytosis	9/110	291/18870	5.19093E-05	0.005547257	0.005008569	ABCA2/AHSG/APOA2/APOE/CCL2/CD47/SERPINE1/VTN/WNT5A	9
HS/CON	BP	GO:0051918	negative regulation of fibrinolysis	3/110	13/18870	5.28288E-05	0.005547257	0.005008569	PLAU/SERPINE1/VTN	3
HS/CON	BP	GO:0050878	regulation of body fluid levels	10/110	365/18870	5.46913E-05	0.005547257	0.005008569	APOE/BLOC1S4/FGA/FGB/GGCX/JMJD1C/PLAU/SERPIND1/SERPINE1/VTN	10
REC/CON	MF	GO:0003714	transcription corepressor activity	8/143	194/18496	0.00013473	0.005894458	0.005353763	ANKRD1/CASP8AP2/TCF25/PFDN5/GMNN/CCND1/BCOR/RIOX2	8
HS/REC	BP	GO:1901990	regulation of mitotic cell cycle phase transition	7/49	355/18870	3.42256E-05	0.006131263	0.004791577	CCL2/PHF10/ZFP36L1/SPDL1/WEE1/CLSPN/FBXO5	7
REC/CON	BP	GO:0030177	positive regulation of Wnt signaling pathway	7/136	146/18870	9.12772E-05	0.006166962	0.005253398	COL1A1/DKK2/SOX4/SFRP2/MACF1/GID8/WNT5A	7
REC/CON	BP	GO:0007369	gastrulation	8/136	197/18870	9.21147E-05	0.006166962	0.005253398	COL12A1/COL5A1/COL11A1/INHBA/SFRP2/NR4A3/VTN/WNT5A	8
REC/CON	BP	GO:0031100	animal organ regeneration	5/136	64/18870	9.79088E-05	0.006390995	0.005444243	APOA2/CCND1/CPB2/NR4A3/VTN	5
HS/REC	BP	GO:0045930	negative regulation of mitotic cell cycle	6/49	248/18870	4.22612E-05	0.006624443	0.005176997	CCL2/ZFP36L1/SPDL1/WEE1/CLSPN/FBXO5	6
REC/CON	BP	GO:0060828	regulation of canonical Wnt signaling pathway	9/136	262/18870	0.000120691	0.007685979	0.00654739	COL1A1/APOE/DKK2/PFDN5/LZTS2/SOX4/SFRP2/GID8/WNT5A	9
REC/CON	BP	GO:0003007	heart morphogenesis	9/136	264/18870	0.000127824	0.007946403	0.006769235	COL5A1/ANKRD1/CCN1/COL11A1/SOX4/SFRP2/AHI1/TPM1/WNT5A	9
HS/REC	BP	GO:0007492	endoderm development	4/49	83/18870	6.34106E-05	0.00883521	0.00690471	COL5A1/MMP2/COL11A1/ZFP36L1	4
HS/REC	BP	GO:1901988	negative regulation of cell cycle phase transition	6/49	277/18870	7.80279E-05	0.009244377	0.007224474	CCL2/ZFP36L1/SPDL1/WEE1/CLSPN/FBXO5	6
HS/REC	BP	GO:0045786	negative regulation of cell cycle	7/49	407/18870	8.1091E-05	0.009244377	0.007224474	CCL2/ZFP36L1/GMNN/SPDL1/WEE1/CLSPN/FBXO5	7
HS/CON	BP	GO:0034375	high-density lipoprotein particle remodeling	3/110	16/18870	0.00010213	0.009888057	0.008927838	APOA2/APOE/PLTP	3
REC/CON	BP	GO:0030111	regulation of Wnt signaling pathway	10/136	338/18870	0.000172191	0.010359447	0.008824814	COL1A1/APOE/DKK2/PFDN5/LZTS2/SOX4/SFRP2/MACF1/GID8/WNT5A	10
REC/CON	BP	GO:0032438	melanosome organization	4/136	39/18870	0.000174575	0.010359447	0.008824814	ABCB6/APOE/BLOC1S4/SNAPIN	4
REC/CON	BP	GO:0044344	cellular response to fibroblast growth factor stimulus	6/136	115/18870	0.00018556	0.010766631	0.009171679	COL1A1/CCL2/CCN2/FGFR1/HYAL2/WNT5A	6
REC/CON	BP	GO:0048753	pigment granule organization	4/136	40/18870	0.000192894	0.010948836	0.009326892	ABCB6/APOE/BLOC1S4/SNAPIN	4
HS/CON	BP	GO:0061041	regulation of wound healing	6/110	133/18870	0.000128715	0.011920122	0.010762571	APOE/FGA/FGB/PLAU/SERPINE1/VTN	6
HS/CON	BP	GO:0008625	extrinsic apoptotic signaling pathway via death domain receptors	5/110	86/18870	0.000146984	0.012572037	0.011351179	CASP8AP2/FGA/FGB/SERPINE1/TIMP3	5
HS/CON	BP	GO:0051917	regulation of fibrinolysis	3/110	18/18870	0.000147559	0.012572037	0.011351179	PLAU/SERPINE1/VTN	3
HS/REC	BP	GO:0001503	ossification	7/49	444/18870	0.000139275	0.013786545	0.010774174	COL1A1/COL1A2/MMP2/COL11A1/SFRP2/BCOR/FBXO5	7
HS/REC	BP	GO:0032963	collagen metabolic process	4/49	106/18870	0.000164077	0.013786545	0.010774174	COL1A1/COL1A2/COL5A1/MMP2	4
HS/REC	BP	GO:1901987	regulation of cell cycle phase transition	7/49	456/18870	0.000164161	0.013786545	0.010774174	CCL2/PHF10/ZFP36L1/SPDL1/WEE1/CLSPN/FBXO5	7
HS/REC	BP	GO:0010948	negative regulation of cell cycle process	6/49	320/18870	0.00017176	0.013786545	0.010774174	CCL2/ZFP36L1/SPDL1/WEE1/CLSPN/FBXO5	6
HS/REC	BP	GO:0071364	cellular response to epidermal growth factor stimulus	3/49	42/18870	0.000175905	0.013786545	0.010774174	COL1A1/ZFP36L1/ERBB2	3
HS/CON	BP	GO:0014910	regulation of smooth muscle cell migration	5/110	89/18870	0.000172775	0.014154259	0.012779753	NR4A3/PLAU/SERPINE1/TPM1/VTN	5
REC/CON	BP	GO:0030100	regulation of endocytosis	9/136	291/18870	0.00026424	0.014248208	0.0121375	ABCA2/APOA2/APOE/CCL2/CD47/AHI1/SERPINE1/VTN/WNT5A	9
REC/CON	BP	GO:0048705	skeletal system morphogenesis	8/136	230/18870	0.000266354	0.014248208	0.0121375	COL1A1/CCN2/COL11A1/FLVCR1/ZEB1/FGFR1/HYAL2/ZFAND5	8
REC/CON	BP	GO:0071774	response to fibroblast growth factor	6/136	123/18870	0.000267393	0.014248208	0.0121375	COL1A1/CCL2/CCN2/FGFR1/HYAL2/WNT5A	6
REC/CON	BP	GO:0010755	regulation of plasminogen activation	3/136	18/18870	0.000276035	0.014414542	0.012279194	PLAUR/CPB2/SERPINE1	3
HS/REC	BP	GO:0044786	cell cycle DNA replication	3/49	44/18870	0.000202195	0.014914859	0.01165595	RTF2/GMNN/FBXO5	3
REC/CON	BP	GO:0003206	cardiac chamber morphogenesis	6/136	127/18870	0.000317742	0.015954328	0.013590879	CCN1/COL11A1/SOX4/SFRP2/TPM1/WNT5A	6
REC/CON	BP	GO:0003231	cardiac ventricle development	6/136	127/18870	0.000317742	0.015954328	0.013590879	CCN1/COL11A1/SOX4/SFRP2/TPM1/WNT5A	6
HS/REC	BP	GO:0070849	response to epidermal growth factor	3/49	46/18870	0.000230908	0.016086582	0.012571651	COL1A1/ZFP36L1/ERBB2	3
HS/CON	BP	GO:1902041	regulation of extrinsic apoptotic signaling pathway via death domain receptors	4/110	50/18870	0.000204772	0.016154266	0.014585542	FGA/FGB/SERPINE1/TIMP3	4
REC/CON	BP	GO:0007492	endoderm development	5/136	83/18870	0.000334237	0.016465907	0.014026673	COL12A1/COL5A1/COL11A1/INHBA/VTN	5
REC/CON	BP	GO:0042476	odontogenesis	6/136	132/18870	0.000390815	0.018373195	0.015651419	COL1A1/COL1A2/FST/INHBA/BCOR/SERPINE1	6
REC/CON	BP	GO:0032964	collagen biosynthetic process	4/136	48/18870	0.000392797	0.018373195	0.015651419	COL1A1/COL5A1/F2/INHBA	4
REC/CON	BP	GO:0008625	extrinsic apoptotic signaling pathway via death domain receptors	5/136	86/18870	0.000394063	0.018373195	0.015651419	CASP8AP2/FGB/SFRP2/SERPINE1/TIMP3	5
HS/REC	BP	GO:0035987	endodermal cell differentiation	3/49	49/18870	0.000278724	0.018391852	0.014373218	COL5A1/MMP2/COL11A1	3
HS/REC	BP	GO:0060346	bone trabecula formation	2/49	10/18870	0.000293331	0.018391852	0.014373218	COL1A1/MMP2	2
HS/CON	BP	GO:0014909	smooth muscle cell migration	5/110	96/18870	0.000246348	0.018740007	0.016920184	NR4A3/PLAU/SERPINE1/TPM1/VTN	5
HS/REC	BP	GO:0001704	formation of primary germ layer	4/49	128/18870	0.000337913	0.020178206	0.015769252	COL5A1/MMP2/COL11A1/SFRP2	4
REC/CON	BP	GO:0044321	response to leptin	3/136	21/18870	0.000442847	0.020285504	0.017280441	FGB/CCND1/NR4A3	3
REC/CON	BP	GO:1902041	regulation of extrinsic apoptotic signaling pathway via death domain receptors	4/136	50/18870	0.000459761	0.020697162	0.017631117	FGB/SFRP2/SERPINE1/TIMP3	4
REC/CON	BP	GO:0060070	canonical Wnt signaling pathway	9/136	316/18870	0.000481904	0.021326302	0.018167057	COL1A1/APOE/DKK2/PFDN5/LZTS2/SOX4/SFRP2/GID8/WNT5A	9
REC/CON	BP	GO:0016055	Wnt signaling pathway	11/136	459/18870	0.000497973	0.021670132	0.018459952	COL1A1/APOE/DKK2/PFDN5/LZTS2/CCND1/SOX4/SFRP2/MACF1/GID8/WNT5A	11
REC/CON	BP	GO:0198738	cell-cell signaling by wnt	11/136	461/18870	0.000516326	0.022100459	0.018826531	COL1A1/APOE/DKK2/PFDN5/LZTS2/CCND1/SOX4/SFRP2/MACF1/GID8/WNT5A	11
REC/CON	BP	GO:0090090	negative regulation of canonical Wnt signaling pathway	6/136	140/18870	0.000534376	0.022504119	0.019170394	APOE/DKK2/PFDN5/LZTS2/SFRP2/WNT5A	6
REC/CON	BP	GO:2001238	positive regulation of extrinsic apoptotic signaling pathway	4/136	53/18870	0.000574912	0.023805111	0.020278659	INHBA/TNFRSF12A/TIMP3/HYAL2	4
REC/CON	BP	GO:0003283	atrial septum development	3/136	23/18870	0.000583503	0.023805111	0.020278659	CCN1/SOX4/WNT5A	3
HS/REC	BP	GO:0043589	skin morphogenesis	2/49	12/18870	0.000428793	0.024441207	0.019100784	COL1A1/COL1A2	2
HS/REC	BP	GO:0001706	endoderm formation	3/49	58/18870	0.000459204	0.024799019	0.019380414	COL5A1/MMP2/COL11A1	3
HS/REC	BP	GO:0000086	G2/M transition of mitotic cell cycle	4/49	140/18870	0.000474622	0.024799019	0.019380414	CCNY/WEE1/CLSPN/FBXO5	4
REC/CON	BP	GO:2001233	regulation of apoptotic signaling pathway	10/136	398/18870	0.000625976	0.025144966	0.02142003	PLAUR/FGB/INHBA/SFRP2/FGFR1/SERPINE1/TNFRSF12A/TIMP3/HYAL2/WNT5A	10
HS/REC	BP	GO:0061430	bone trabecula morphogenesis	2/49	13/18870	0.000505915	0.025376715	0.019831882	COL1A1/MMP2	2
HS/REC	BP	GO:0060828	regulation of canonical Wnt signaling pathway	5/49	262/18870	0.000575098	0.027737437	0.021676785	COL1A1/CCNY/SOX4/SFRP2/IGFBP4	5
REC/CON	MF	GO:0008171	O-methyltransferase activity	3/143	23/18496	0.000715488	0.027824522	0.025272197	MRM1/PCMTD1/TRMT13	3
HS/CON	BP	GO:0031638	zymogen activation	4/110	59/18870	0.000388716	0.02855053	0.025778018	FGA/FGB/PLAU/SERPINE1	4
REC/CON	BP	GO:0032102	negative regulation of response to external stimulus	11/136	481/18870	0.000733149	0.029003811	0.024707232	APOE/F2/CCL2/PLAUR/FGB/ASH1L/SLC39A8/CPB2/SERPINE1/VTN/WNT5A	11
REC/CON	MF	GO:0050840	extracellular matrix binding	4/143	55/18496	0.000860492	0.030117237	0.027354604	CCN1/COL11A1/BGN/VTN	4
HS/CON	BP	GO:2000351	regulation of endothelial cell apoptotic process	4/110	61/18870	0.000441744	0.03136381	0.028318103	CCL2/FGA/FGB/SERPINE1	4
HS/CON	BP	GO:0014812	muscle cell migration	5/110	110/18870	0.00046247	0.031776198	0.028690444	NR4A3/PLAU/SERPINE1/TPM1/VTN	5
HS/CON	BP	GO:0043086	negative regulation of catalytic activity	10/110	480/18870	0.000500141	0.032297237	0.029160886	AHSG/APOA2/APOE/CDK5RAP1/CDKN2A/PNKP/SERPIND1/SERPINE1/TIMP3/VTN	10
HS/CON	BP	GO:0045785	positive regulation of cell adhesion	10/110	482/18870	0.000516567	0.032297237	0.029160886	CCDC80/CCL2/CCN1/CD47/FGA/FGB/NR4A3/TPM1/VTN/WNT5A	10
HS/CON	BP	GO:0031100	animal organ regeneration	4/110	64/18870	0.000530681	0.032297237	0.029160886	APOA2/CDK1/NR4A3/VTN	4
HS/CON	BP	GO:1903034	regulation of response to wounding	6/110	173/18870	0.000530706	0.032297237	0.029160886	APOE/FGA/FGB/PLAU/SERPINE1/VTN	6
HS/REC	BP	GO:0044839	cell cycle G2/M phase transition	4/49	155/18870	0.000696017	0.032326119	0.025262837	CCNY/WEE1/CLSPN/FBXO5	4
HS/CON	BP	GO:0030194	positive regulation of blood coagulation	3/110	28/18870	0.000567802	0.032686961	0.029512764	PLAU/SERPINE1/VTN	3
HS/CON	BP	GO:1900048	positive regulation of hemostasis	3/110	28/18870	0.000567802	0.032686961	0.029512764	PLAU/SERPINE1/VTN	3
REC/CON	BP	GO:0031638	zymogen activation	4/136	59/18870	0.000864268	0.033680665	0.028691264	PLAUR/FGB/CPB2/SERPINE1	4
HS/CON	BP	GO:0072577	endothelial cell apoptotic process	4/110	67/18870	0.000631667	0.035406573	0.031968277	CCL2/FGA/FGB/SERPINE1	4
HS/CON	MF	GO:0120020	cholesterol transfer activity	3/115	22/18496	0.000330797	0.035898029	0.031309559	APOA2/APOE/PLTP	3
HS/CON	MF	GO:0008171	O-methyltransferase activity	3/115	23/18496	0.000378697	0.035898029	0.031309559	MRM1/PCMTD1/TRMT13	3
HS/CON	MF	GO:0120015	sterol transfer activity	3/115	23/18496	0.000378697	0.035898029	0.031309559	APOA2/APOE/PLTP	3
HS/CON	MF	GO:0061134	peptidase regulator activity	7/115	228/18496	0.000559868	0.035898029	0.031309559	ABCA2/AHSG/CASP8AP2/ITIH4/SERPIND1/SERPINE1/TIMP3	7
HS/CON	MF	GO:0005539	glycosaminoglycan binding	7/115	240/18496	0.000757919	0.035898029	0.031309559	APOE/CCDC80/CCN1/CCN2/HABP2/SERPIND1/VTN	7
HS/CON	MF	GO:0008201	heparin binding	6/115	174/18496	0.000767448	0.035898029	0.031309559	APOE/CCDC80/CCN1/CCN2/SERPIND1/VTN	6
HS/CON	MF	GO:0071813	lipoprotein particle binding	3/115	31/18496	0.000927011	0.035898029	0.031309559	APOA2/APOE/PLTP	3
HS/CON	MF	GO:0071814	protein-lipid complex binding	3/115	31/18496	0.000927011	0.035898029	0.031309559	APOA2/APOE/PLTP	3

Cluster	ONTOLOGY	ID	Description	GeneRatio	BgRatio	pvalue	p.adjust	qvalue	geneID	Count
HS/CON	MF	GO:0061135	endopeptidase regulator activity	6/115	184/18496	0.001025658	0.035898029	0.031309559	ABCA2/AHSG/ITIH4/SERPIND1/SERPINE1/TIMP3	6
REC/CON	BP	GO:0033081	regulation of T cell differentiation in thymus	3/136	27/18870	0.000943636	0.036232865	0.030865385	ERBB2/ZEB1/ZC3H8	3
REC/CON	BP	GO:0055008	cardiac muscle tissue morphogenesis	4/136	61/18870	0.000980044	0.037085425	0.031591648	ANKRD1/COL11A1/TPM1/WNT5A	4
HS/REC	BP	GO:0003208	cardiac ventricle morphogenesis	3/49	71/18870	0.00083069	0.037203028	0.029074137	COL11A1/SOX4/SFRP2	3
HS/CON	BP	GO:0050820	positive regulation of coagulation	3/110	30/18870	0.00069775	0.038107875	0.034407258	PLAU/SERPINE1/VTN	3
REC/CON	BP	GO:0032963	collagen metabolic process	5/136	106/18870	0.001022436	0.038136868	0.032487332	COL1A1/COL1A2/COL5A1/F2/INHBA	5
REC/CON	BP	GO:0090303	positive regulation of wound healing	4/136	62/18870	0.001041836	0.038313146	0.032637496	F2/CPB2/SERPINE1/VTN	4
REC/CON	BP	GO:0010810	regulation of cell-substrate adhesion	7/136	220/18870	0.001090284	0.038786278	0.033040539	COL1A1/CCDC80/CCN1/FGB/MACF1/SERPINE1/VTN	7
REC/CON	BP	GO:0033059	cellular pigmentation	4/136	63/18870	0.001106314	0.038786278	0.033040539	ABCB6/APOE/BLOC1S4/SNAPIN	4
REC/CON	BP	GO:0032757	positive regulation of interleukin-8 production	4/136	63/18870	0.001106314	0.038786278	0.033040539	APOA2/SERPINE1/HYAL2/WNT5A	4
REC/CON	BP	GO:0031589	cell-substrate adhesion	9/136	356/18870	0.001121476	0.038786278	0.033040539	COL1A1/CCDC80/CCN1/CCN2/FGB/MACF1/MSLN/SERPINE1/VTN	9
REC/CON	BP	GO:0090287	regulation of cellular response to growth factor stimulus	9/136	357/18870	0.001143573	0.038786278	0.033040539	FST/FSTL1/CCN1/EPN2/ZEB1/SFRP2/FGFR1/VTN/WNT5A	9
REC/CON	BP	GO:0003209	cardiac atrium morphogenesis	3/136	29/18870	0.001166484	0.038786278	0.033040539	CCN1/SOX4/WNT5A	3
REC/CON	BP	GO:0097421	liver regeneration	3/136	29/18870	0.001166484	0.038786278	0.033040539	CCND1/CPB2/VTN	3
REC/CON	BP	GO:0043462	regulation of ATP-dependent activity	4/136	64/18870	0.001173541	0.038786278	0.033040539	TPM2/PXK/TPM1/UHRF1	4
HS/REC	BP	GO:0045661	regulation of myoblast differentiation	3/49	73/18870	0.000900671	0.03894626	0.030436471	PHF10/ZFP36L1/SOX4	3
REC/CON	BP	GO:0090263	positive regulation of canonical Wnt signaling pathway	5/136	110/18870	0.001206866	0.039389078	0.033554041	COL1A1/DKK2/SOX4/SFRP2/GID8	5
HS/CON	BP	GO:0010811	positive regulation of cell-substrate adhesion	5/110	123/18870	0.000769067	0.039963205	0.036082419	CCDC80/CCN1/FGA/FGB/VTN	5
HS/CON	BP	GO:1902042	negative regulation of extrinsic apoptotic signaling pathway via death domain receptors	3/110	31/18870	0.000769245	0.039963205	0.036082419	FGA/FGB/SERPINE1	3
HS/CON	BP	GO:0070371	ERK1 and ERK2 cascade	8/110	336/18870	0.000788883	0.040007637	0.036122537	APOE/CCL2/CCN1/CCN2/CDK1/FGA/FGB/TIMP3	8
REC/CON	MF	GO:0005178	integrin binding	6/143	154/18496	0.001265024	0.040250772	0.036558596	COL5A1/CCN1/CCN2/ESM1/SFRP2/VTN	6
REC/CON	BP	GO:0071347	cellular response to interleukin-1	5/136	111/18870	0.001256582	0.040505392	0.034504986	ANKRD1/CCL2/CD47/FGB/HYAL2	5
HS/CON	BP	GO:0050766	positive regulation of phagocytosis	4/110	72/18870	0.00082914	0.041071336	0.037082941	AHSG/APOA2/CCL2/CD47	4
HS/CON	BP	GO:0033344	cholesterol efflux	4/110	73/18870	0.000873303	0.041957992	0.037883495	ABCA2/APOA2/APOE/PLTP	4
HS/CON	BP	GO:0010951	negative regulation of endopeptidase activity	5/110	128/18870	0.000920289	0.041957992	0.037883495	AHSG/SERPIND1/SERPINE1/TIMP3/VTN	5
HS/CON	BP	GO:0034369	plasma lipoprotein particle remodeling	3/110	33/18870	0.000925834	0.041957992	0.037883495	APOA2/APOE/PLTP	3
HS/CON	BP	GO:0034368	protein-lipid complex remodeling	3/110	33/18870	0.000925834	0.041957992	0.037883495	APOA2/APOE/PLTP	3
REC/CON	BP	GO:0003205	cardiac chamber development	6/136	168/18870	0.001379745	0.043933115	0.037424931	CCN1/COL11A1/SOX4/SFRP2/TPM1/WNT5A	6
HS/REC	BP	GO:0031397	negative regulation of protein ubiquitination	3/49	78/18870	0.001091693	0.044243014	0.03457588	DNAJA1/SOX4/FBXO5	3
HS/REC	BP	GO:0031214	biomineral tissue development	4/49	175/18870	0.001093727	0.044243014	0.03457588	COL1A1/COL1A2/BCOR/FBXO5	4
REC/CON	BP	GO:1902042	negative regulation of extrinsic apoptotic signaling pathway via death domain receptors	3/136	31/18870	0.001419955	0.044668705	0.038051552	FGB/SFRP2/SERPINE1	3
HS/CON	MF	GO:0015248	sterol transporter activity	3/115	36/18496	0.001439616	0.045347915	0.039551565	APOA2/APOE/PLTP	3
HS/CON	BP	GO:0034470	ncRNA processing	9/110	439/18870	0.001074865	0.047697152	0.04306533	CDK5RAP1/IMP3/MRM1/PDCD11/PUS7/THUMPD2/TRMT13/TSEN34/ZC3H7B	9
HS/REC	BP	GO:0055093	response to hyperoxia	2/49	20/18870	0.001218137	0.047735742	0.037305444	COL1A1/MMP2	2
HS/REC	BP	GO:0060070	canonical Wnt signaling pathway	5/49	316/18870	0.001330759	0.04784711	0.037392478	COL1A1/CCNY/SOX4/SFRP2/IGFBP4	5
HS/REC	BP	GO:0071230	cellular response to amino acid stimulus	3/49	85/18870	0.001399425	0.04784711	0.037392478	COL1A1/COL1A2/MMP2	3
HS/REC	BP	GO:0051591	response to cAMP	3/49	85/18870	0.001399425	0.04784711	0.037392478	COL1A1/VGF/ZFP36L1	3
HS/REC	BP	GO:2000781	positive regulation of double-strand break repair	3/49	86/18870	0.001447374	0.04784711	0.037392478	MORF4L2/PHF10/RAD51AP1	3
HS/REC	BP	GO:0050673	epithelial cell proliferation	6/49	480/18870	0.001453323	0.04784711	0.037392478	SPARC/CCL2/ZFP36L1/ERBB2/SFRP2/IGFBP4	6
HS/REC	BP	GO:0035313	wound healing, spreading of epidermal cells	2/49	22/18870	0.001476099	0.04784711	0.037392478	COL5A1/AJUBA	2
HS/REC	BP	GO:1903321	negative regulation of protein modification by small protein conjugation or removal	3/49	90/18870	0.001649461	0.04784711	0.037392478	DNAJA1/SOX4/FBXO5	3
HS/REC	BP	GO:0030071	regulation of mitotic metaphase/anaphase transition	3/49	90/18870	0.001649461	0.04784711	0.037392478	PHF10/SPDL1/FBXO5	3
HS/REC	BP	GO:0007369	gastrulation	4/49	197/18870	0.001691175	0.04784711	0.037392478	COL5A1/MMP2/COL11A1/SFRP2	4
HS/REC	BP	GO:0071214	cellular response to abiotic stimulus	5/49	334/18870	0.001698284	0.04784711	0.037392478	COL1A1/MMP2/ZFP36L1/SFRP2/RAD51AP1	5
HS/REC	BP	GO:0104004	cellular response to environmental stimulus	5/49	334/18870	0.001698284	0.04784711	0.037392478	COL1A1/MMP2/ZFP36L1/SFRP2/RAD51AP1	5
HS/REC	BP	GO:0031396	regulation of protein ubiquitination	4/49	200/18870	0.001787209	0.04784711	0.037392478	DNAJA1/SOX4/RCHY1/FBXO5	4
HS/REC	BP	GO:0030111	regulation of Wnt signaling pathway	5/49	338/18870	0.001789182	0.04784711	0.037392478	COL1A1/CCNY/SOX4/SFRP2/IGFBP4	5
HS/REC	BP	GO:1902099	regulation of metaphase/anaphase transition of cell cycle	3/49	93/18870	0.001812056	0.04784711	0.037392478	PHF10/SPDL1/FBXO5	3
HS/REC	BP	GO:0071229	cellular response to acid chemical	3/49	94/18870	0.001868397	0.04784711	0.037392478	COL1A1/COL1A2/MMP2	3
HS/REC	BP	GO:0007091	metaphase/anaphase transition of mitotic cell cycle	3/49	94/18870	0.001868397	0.04784711	0.037392478	PHF10/SPDL1/FBXO5	3
HS/REC	BP	GO:0008156	negative regulation of DNA replication	2/49	25/18870	0.00190751	0.04784711	0.037392478	GMNN/FBXO5	2
HS/REC	BP	GO:0070482	response to oxygen levels	5/49	343/18870	0.00190778	0.04784711	0.037392478	COL1A1/MMP2/ZFP36L1/AJUBA/SOX4	5
HS/CON	BP	GO:0034367	protein-containing complex remodeling	3/110	35/18870	0.00110127	0.047871535	0.04322278	APOA2/APOE/PLTP	3
HS/CON	BP	GO:0008033	tRNA processing	5/110	134/18870	0.001129764	0.048127939	0.043454284	CDK5RAP1/PUS7/THUMPD2/TRMT13/TSEN34	5
HS/REC	BP	GO:0044784	metaphase/anaphase transition of cell cycle	3/49	97/18870	0.002043953	0.048812572	0.038146986	PHF10/SPDL1/FBXO5	3
HS/REC	BP	GO:1901874	negative regulation of post-translational protein modification	3/49	97/18870	0.002043953	0.048812572	0.038146986	DNAJA1/SOX4/FBXO5	3
HS/REC	BP	GO:0060343	trabecula formation	2/49	26/18870	0.002063051	0.048812572	0.038146986	COL1A1/MMP2	2
HS/CON	BP	GO:2000352	negative regulation of endothelial cell apoptotic process	3/110	36/18870	0.001196316	0.049181114	0.044405187	FGA/FGB/SERPINE1	3
HS/CON	BP	GO:0032374	regulation of cholesterol transport	4/110	80/18870	0.001230016	0.049181114	0.044405187	ABCA2/APOA2/APOE/PLTP	4
HS/CON	BP	GO:0032371	regulation of sterol transport	4/110	80/18870	0.001230016	0.049181114	0.044405187	ABCA2/APOA2/APOE/PLTP	4
HS/CON	BP	GO:0010466	negative regulation of peptidase activity	5/110	137/18870	0.001246845	0.049181114	0.044405187	AHSG/SERPIND1/SERPINE1/TIMP3/VTN	5
REC/CON	BP	GO:0050680	negative regulation of epithelial cell proliferation	6/136	173/18870	0.001602118	0.049213288	0.041922909	APOE/CCL2/ZEB1/SFRP2/CPB2/WNT5A	6
REC/CON	BP	GO:0030178	negative regulation of Wnt signaling pathway	6/136	173/18870	0.001602118	0.049213288	0.041922909	APOE/DKK2/PFDN5/LZTS2/SFRP2/WNT5A	6
HS/REC	BP	GO:0045619	regulation of lymphocyte differentiation	4/49	212/18870	0.002209386	0.049810903	0.038927181	PHF10/ZFP36L1/ERBB2/SOX4	4
HS/REC	BP	GO:0042026	protein refolding	2/49	27/18870	0.00222441	0.049810903	0.038927181	DNAJA1/HSPA6	2
HS/REC	BP	GO:0036296	response to increased oxygen levels	2/49	27/18870	0.00222441	0.049810903	0.038927181	COL1A1/MMP2	2
