# Supplementary material for: Establishment and characterization of Hanwoo cumulus cell line for heat stress studies
Source: Anim Biosci. 2026 Jun 15;39(7):250896. doi: 10.5713/ab.250896 (PMC13353149; doi:10.5713/ab.250896)
Supplement: Supplementary file 13 [file ab-250896-Supplementary-13.pdf]

Supplement 13. Cumulus upregulated DEG pathway enrichment (KEGG and Reactome)

<table>
<tr><th>_LogP_HS_CON</th><th>_LogP_HS_REC</th><th>_LogP_REC_CON</th><th>GO</th><th>Category</th><th>Description</th><th>Enrichment</th><th>#GeneInGOAndHitList</th><th>Hits</th></tr>
<tr><td>-8.212337332</td><td>-10.68056349</td><td>0</td><td>R-HSA-3371571</td><td>Reactome Gene Sets</td><td>HSF1-dependent transactivation</td><td>103.5547945</td><td>6</td><td>FKBP4|HSBP1|HSPA1A|HSP90AA1|DNAJB1|HSPB8</td></tr>
<tr><td>-7.128189394</td><td>-10.36846143</td><td>0</td><td>R-HSA-3371556</td><td>Reactome Gene Sets</td><td>Cellular response to heat stress</td><td>37.65628892</td><td>8</td><td>FKBP4|HSBP1|HSPA1A|HSP90AA1|DNAJB1|BAG3|HSPA4L|HSPB8</td></tr>
<tr><td>-5.878687032</td><td>-9.852629008</td><td>0</td><td>R-HSA-3371568</td><td>Reactome Gene Sets</td><td>Attenuation phase</td><td>147.9354207</td><td>5</td><td>FKBP4|HSBP1|HSPA1A|HSP90AA1|DNAJB1</td></tr>
<tr><td>-4.569293747</td><td>-6.567187373</td><td>0</td><td>R-HSA-3371497</td><td>Reactome Gene Sets</td><td>HSP90 chaperone cycle for steroid hormone receptors (SHR) in the presence of ligand</td><td>36.33501562</td><td>5</td><td>FKBP4|HSPA1A|HSP90AA1|DNAJB1|DNAJA4</td></tr>
<tr><td>-4.47899267</td><td>-4.705837709</td><td>0</td><td>R-HSA-2262752</td><td>Reactome Gene Sets</td><td>Cellular responses to stress</td><td>3.088936265</td><td>23</td><td>AREG|CRYAB|FKBP4|GPX3|HSBP1|DNAJA1|HSPA1A|HSP90AA1|HSPE1|DNAJB1|RPL7A|TRIM21|H2BC21|H2BC11|BAG3|TATDN2|TUBB4A|ANAPC10|HSPA4L|HSPB8|DNAJA4|OMA1|CREBRF</td></tr>
<tr><td>-2.067399957</td><td>-4.644998407</td><td>0</td><td>R-HSA-3371453</td><td>Reactome Gene Sets</td><td>Regulation of HSF1-mediated heat shock response</td><td>24.365834</td><td>4</td><td>HSPA1A|DNAJB1|BAG3|HSPA4L</td></tr>
<tr><td>-3.15475797</td><td>0</td><td>0</td><td>R-HSA-446219</td><td>Reactome Gene Sets</td><td>Synthesis of substrates in N-glycan biosythesis</td><td>9.793684211</td><td>6</td><td>DPM2|ST3GAL5|ST3GAL6|SLC35A1|SLC35C1|GNPNAT1</td></tr>
<tr><td>0</td><td>0</td><td>-4.27342306</td><td>hsa04668</td><td>KEGG Pathway</td><td>TNF signaling pathway</td><td>12.45592355</td><td>5</td><td>EDN1|ICAM1|SELE|TNFAIP3|TNFRSF1B</td></tr>
<tr><td>-2.817338374</td><td>0</td><td>0</td><td>R-HSA-446193</td><td>Reactome Gene Sets</td><td>Biosynthesis of the N-glycan precursor (dolichol lipid-linked oligosaccharide, LLO) and transfer to a nascent protein</td><td>7.957368421</td><td>6</td><td>DPM2|ST3GAL5|ST3GAL6|SLC35A1|SLC35C1|GNPNAT1</td></tr>
<tr><td>-3.221257233</td><td>0</td><td>0</td><td>R-HSA-3295583</td><td>Reactome Gene Sets</td><td>TRP channels</td><td>15.71825861</td><td>4</td><td>TRPC3|TRPA1|MCOLN3|MCOLN2</td></tr>
<tr><td>-3.758004546</td><td>0</td><td>0</td><td>R-HSA-9841251</td><td>Reactome Gene Sets</td><td>Mitochondrial unfolded protein response (UPRmt)</td><td>27.09498208</td><td>3</td><td>DNAJA1|HSPA1A|HSPE1</td></tr>
<tr><td>-2.301271679</td><td>-3.466817416</td><td>0</td><td>hsa05134</td><td>KEGG Pathway</td><td>Legionellosis</td><td>22.19031311</td><td>3</td><td>CLK1|HSPA1A|CLK4</td></tr>
<tr><td>0</td><td>-3.438951597</td><td>0</td><td>hsa04915</td><td>KEGG Pathway</td><td>Estrogen signaling pathway</td><td>11.91997635</td><td>4</td><td>FKBP4|HSPA1A|HSP90AA1|KRT31</td></tr>
<tr><td>0</td><td>0</td><td>-3.41154135</td><td>hsa04933</td><td>KEGG Pathway</td><td>AGE-RAGE signaling pathway in diabetic complications</td><td>11.74063289</td><td>4</td><td>EDN1|ICAM1|SELE|STAT5A</td></tr>
<tr><td>0</td><td>0</td><td>-3.347536929</td><td>hsa04064</td><td>KEGG Pathway</td><td>NF-kappa B signaling pathway</td><td>11.29337068</td><td>4</td><td>ICAM1|GADD45B|TNFAIP3|RIGI</td></tr>
<tr><td>0</td><td>0</td><td>-3.289976894</td><td>R-HSA-9006931</td><td>Reactome Gene Sets</td><td>Signaling by Nuclear Receptors</td><td>6.009141494</td><td>6</td><td>ABCA1|AREG|CRABP2|EREG|H2BC21|H2BC11</td></tr>
<tr><td>-3.163356316</td><td>-3.10066287</td><td>0</td><td>hsa04141</td><td>KEGG Pathway</td><td>Protein processing in endoplasmic reticulum</td><td>5.704206753</td><td>6</td><td>CRYAB|DNAJB2|DNAJA1|HSPA1A|HSP90AA1|HSPA4L</td></tr>
<tr><td>-2.899938231</td><td>0</td><td>0</td><td>R-HSA-9833482</td><td>Reactome Gene Sets</td><td>PKR-mediated signaling</td><td>8.55631013</td><td>4</td><td>FANCB|HSPA1A|EIF2AK2|TUBB4A</td></tr>
<tr><td>0</td><td>0</td><td>-2.898846839</td><td>R-HSA-1280215</td><td>Reactome Gene Sets</td><td>Cytokine Signaling in Immune system</td><td>3.438220133</td><td>9</td><td>ICAM1|TNFRSF9|TRIM21|STAT5A|TNFRSF1B|IL1RL1|TUBB4A|RIGI|ALPK1</td></tr>
<tr><td>0</td><td>0</td><td>-2.163689305</td><td>R-HSA-8939211</td><td>Reactome Gene Sets</td><td>ESR-mediated signaling</td><td>3.823360202</td><td>8</td><td>AREG|EREG|FKBP4|HSPB1|HSP90AA1|PPID|H2BC21|H2BC11</td></tr>
<tr><td>0</td><td>0</td><td>-2.709318676</td><td>R-HSA-2299718</td><td>Reactome Gene Sets</td><td>Condensation of Prophase Chromosomes</td><td>12.182917</td><td>3</td><td>H2BC21|H2BC11|PHF8</td></tr>
<tr><td>0</td><td>0</td><td>-2.585924265</td><td>R-HSA-5688426</td><td>Reactome Gene Sets</td><td>Deubiquitination</td><td>5.312741584</td><td>5</td><td>TNFAIP3|H2BC21|H2BC11|RIGI|USP30</td></tr>
<tr><td>-2.570639575</td><td>0</td><td>0</td><td>R-HSA-3899300</td><td>Reactome Gene Sets</td><td>SUMOylation of transcription cofactors</td><td>10.83799283</td><td>3</td><td>CASP8AP2|DDX17|UHRF2</td></tr>
<tr><td>0</td><td>0</td><td>-2.506767763</td><td>hsa04012</td><td>KEGG Pathway</td><td>ErbB signaling pathway</td><td>10.34131327</td><td>3</td><td>AREG|EREG|STAT5A</td></tr>
<tr><td>0</td><td>0</td><td>-2.492583013</td><td>hsa05210</td><td>KEGG Pathway</td><td>Colorectal cancer</td><td>10.2224476</td><td>3</td><td>AREG|EREG|GADD45B</td></tr>
<tr><td>0</td><td>0</td><td>-2.492583013</td><td>R-HSA-912446</td><td>Reactome Gene Sets</td><td>Meiotic recombination</td><td>10.2224476</td><td>3</td><td>BRCA2|H2BC21|H2BC11</td></tr>
<tr><td>0</td><td>0</td><td>-2.398003121</td><td>R-HSA-8936459</td><td>Reactome Gene Sets</td><td>RUNX1 regulates genes involved in megakaryocyte differentiation and platelet function</td><td>9.461201502</td><td>3</td><td>NR4A3|H2BC21|H2BC11</td></tr>
<tr><td>0</td><td>0</td><td>-2.299198676</td><td>R-HSA-5689880</td><td>Reactome Gene Sets</td><td>Ub-specific processing proteases</td><td>5.841398628</td><td>4</td><td>H2BC21|H2BC11|RIGI|USP30</td></tr>
<tr><td>0</td><td>0</td><td>-2.13426087</td><td>R-HSA-1989781</td><td>Reactome Gene Sets</td><td>PPARA activates gene expression</td><td>4.53411306</td><td>5</td><td>ABCA1|CYP1A1|ANKRD1|MED29|MED11</td></tr>
<tr><td>0</td><td>0</td><td>-2.291706288</td><td>hsa05169</td><td>KEGG Pathway</td><td>Epstein-Barr virus infection</td><td>5.812764321</td><td>4</td><td>ICAM1|GADD45B|TNFAIP3|RIGI</td></tr>
<tr><td>0</td><td>0</td><td>-2.114088155</td><td>R-HSA-400206</td><td>Reactome Gene Sets</td><td>Regulation of lipid metabolism by PPARalpha</td><td>4.45790948</td><td>5</td><td>ABCA1|CYP1A1|ANKRD1|MED29|MED11</td></tr>
<tr><td>0</td><td>0</td><td>-2.165249404</td><td>R-HSA-6811558</td><td>Reactome Gene Sets</td><td>PI5P, PP2A and IER3 Regulate PI3K/AKT Signaling</td><td>7.801341589</td><td>3</td><td>AREG|EREG|IL1RL1</td></tr>
<tr><td>0</td><td>0</td><td>-2.114088155</td><td>R-HSA-1500620</td><td>Reactome Gene Sets</td><td>Meiosis</td><td>7.473554128</td><td>3</td><td>BRCA2|H2BC21|H2BC11</td></tr>
<tr><td>-2.110701083</td><td>0</td><td>0</td><td>R-HSA-5173105</td><td>Reactome Gene Sets</td><td>O-linked glycosylation</td><td>5.160948967</td><td>4</td><td>SLC35A1|B4GAT1|GCNT4|ADAMTSL4</td></tr>
<tr><td>0</td><td>0</td><td>-2.094289187</td><td>R-HSA-199418</td><td>Reactome Gene Sets</td><td>Negative regulation of the PI3K/AKT network</td><td>7.350024307</td><td>3</td><td>AREG|EREG|IL1RL1</td></tr>
<tr><td>0</td><td>0</td><td>-2.068200465</td><td>hsa05014</td><td>KEGG Pathway</td><td>Amyotrophic lateral sclerosis</td><td>3.995296232</td><td>5</td><td>GPX3|TNFRSF1B|DNALI1|TUBB4A|SMCR8</td></tr>
</table>
