# Supplementary material for: Establishment and characterization of Hanwoo cumulus cell line for heat stress studies
Source: Anim Biosci. 2026 Jun 15;39(7):250896. doi: 10.5713/ab.250896 (PMC13353149; doi:10.5713/ab.250896)
Supplement: Supplementary file 14 [file ab-250896-Supplementary-14.pdf]

Supplement 14. Cumulus downregulated DEG pathway enrichment (KEGG and Reactome)

_LogP_HS_CON	_LogP_HS_REC	_LogP_REC_CON	GO	Category	Description	Enrichment	#GeneInGOAndHitList	Hits
-5.089532173	-9.075036342	0	hsa04933	KEGG Pathway	AGE-RAGE signaling pathway in diabetic complications	11.74063289	12	BCL2|EDN1|EGR1|ICAM1|IL6|MAPK3|MAPK9|MAPK10|TGFB2|THBD|VCAM1|PLCB1
-2.624062182	-8.373822537	0	hsa04668	KEGG Pathway	TNF signaling pathway	13.44448891	10	EDN1|FOS|ICAM1|IL6|LIF|MAPK3|MAPK9|TNFAIP3|TRAF3|VCAM1
-2.455861898	-6.889003859	0	hsa05417	KEGG Pathway	Lipid and atherosclerosis	8.147609249	11	ABCA1|BCL2|CD36|FOS|ICAM1|IL6|MAPK3|MAPK9|TRAF3|VCAM1|PLCB1
-3.035908002	-4.470345479	0	hsa05200	KEGG Pathway	Pathways in cancer	3.707954727	20	ARAF|BCL2|DAPK3|EDN1|FOS|GSTA3|IL6|ITGA2|MDM2|MMP1|PLD2|MAPK3|MAPK9|MAPK10|RAC3|STAT6|TGFB2|TRAF3|WNT5A|PLCB1
-2.988798644	-6.026641805	0	hsa05161	KEGG Pathway	Hepatitis B	6.668631461	11	ARAF|BCL2|FOS|IL6|MAPK3|MAPK9|MAPK10|STAT6|TGFB2|TRAF3|TYK2
-2.349151727	-4.48723968	-2.876929219	hsa05418	KEGG Pathway	Fluid shear stress and atherosclerosis	6.958943202	10	BCL2|EDN1|FOS|GSTA3|ICAM1|MAPK9|MAPK10|RAC3|THBD|VCAM1
0	-4.554020491	0	R-HSA-450341	Reactome Gene Sets	Activation of the AP-1 family of transcription factors	39.52679739	4	FOS|MAPK3|MAPK9|MAPK10
-4.253352425	-3.656041198	0	hsa05210	KEGG Pathway	Colorectal cancer	9.086620089	8	ARAF|BCL2|FOS|MAPK3|MAPK9|MAPK10|RAC3|TGFB2
-2.983669999	-4.520393703	0	hsa04657	KEGG Pathway	IL-17 signaling pathway	8.321431029	8	FOS|IL6|MMP1|MAPK3|MAPK9|MAPK10|TNFAIP3|TRAF3
0	-5.082506718	0	hsa04928	KEGG Pathway	Parathyroid hormone synthesis, secretion and action	9.738486312	7	ARAF|BCL2|EGR1|FOS|PLD2|MAPK3|PLCB1
-3.32710597	-2.853311353	0	hsa05212	KEGG Pathway	Pancreatic cancer	8.983363042	7	ARAF|PLD2|MAPK3|MAPK9|MAPK10|RAC3|TGFB2
0	-4.389730804	0	R-HSA-450282	Reactome Gene Sets	MAPK targets/ Nuclear events mediated by MAP kinases	15.93822475	5	DUSP6|FOS|MAPK3|MAPK9|MAPK10
0	-4.42913599	0	R-HSA-166016	Reactome Gene Sets	Toll Like Receptor 4 (TLR4) Cascade	7.723627075	7	CD36|DUSP6|FOS|MAPK3|MAPK9|TRAF3|MAPKAPK3
0	-4.324227705	0	R-HSA-450294	Reactome Gene Sets	MAP kinase activation	9.411142235	6	DUSP6|FOS|MAPK3|MAPK9|MAPK10|MAPKAPK3
0	-4.275229208	0	R-HSA-168164	Reactome Gene Sets	Toll Like Receptor 3 (TLR3) Cascade	9.142252457	6	DUSP6|FOS|MAPK3|MAPK9|TRAF3|MAPKAPK3
-2.485661195	-2.860632856	0	hsa04926	KEGG Pathway	Relaxin signaling pathway	6.081045752	8	ACTA2|EDN1|FOS|MMP1|MAPK3|MAPK9|MAPK10|PLCB1
0	-4.184406568	0	hsa04620	KEGG Pathway	Toll-like receptor signaling pathway	8.806756954	6	FOS|IL6|MAPK3|MAPK9|TRAF3|TYK2
0	-4.184406568	0	hsa04659	KEGG Pathway	Th17 cell differentiation	8.806756954	6	FOS|IL6|MAPK3|MAPK9|STAT6|TYK2
0	-4.162286319	0	R-HSA-166166	Reactome Gene Sets	MyD88-independent TLR4 cascade	8.726695527	6	DUSP6|FOS|MAPK3|MAPK9|TRAF3|MAPKAPK3
0	-4.162286319	0	R-HSA-937061	Reactome Gene Sets	TRIF (TICAM1)-mediated TLR4 signaling	8.726695527	6	DUSP6|FOS|MAPK3|MAPK9|TRAF3|MAPKAPK3
0	-4.140391527	0	R-HSA-166058	Reactome Gene Sets	MyD88:MAL(TIRAP) cascade initiated on plasma membrane	8.648076648	6	CD36|DUSP6|FOS|MAPK3|MAPK9|MAPKAPK3
0	-4.140391527	0	R-HSA-168188	Reactome Gene Sets	Toll Like Receptor TLR6:TLR2 Cascade	8.648076648	6	CD36|DUSP6|FOS|MAPK3|MAPK9|MAPKAPK3
0	-4.076019006	0	R-HSA-168179	Reactome Gene Sets	Toll Like Receptor TLR1:TLR2 Cascade	8.420495684	6	CD36|DUSP6|FOS|MAPK3|MAPK9|MAPKAPK3
0	-4.076019006	0	R-HSA-181438	Reactome Gene Sets	Toll Like Receptor 2 (TLR2) Cascade	8.420495684	6	CD36|DUSP6|FOS|MAPK3|MAPK9|MAPKAPK3
0	-4.074036192	0	R-HSA-448424	Reactome Gene Sets	Interleukin-17 signaling	8.350731842	6	DUSP6|FOS|MAPK3|MAPK9|MAPK10|MAPKAPK3
-2.853495045	-3.316376531	0	hsa05142	KEGG Pathway	Chagas disease	6.715718002	7	FOS|IL6|MAPK3|MAPK9|MAPK10|TGFB2|PLCB1
0	-3.992869493	0	R-HSA-168898	Reactome Gene Sets	Toll-like Receptor Cascades	6.587799564	7	CD36|DUSP6|FOS|MAPK3|MAPK9|TRAF3|MAPKAPK3
0	-3.958693259	0	R-HSA-9006931	Reactome Gene Sets	Signaling by Nuclear Receptors	4.864543115	9	ABCA1|BCL2|CRABP2|FOS|H2BC5|PRMT1|MAPK3|H2BC21|H2BC11
0	-3.611908176	0	hsa05167	KEGG Pathway	Kaposi sarcoma-associated herpesvirus infection	4.537515006	9	ANGPT2|FOS|ICAM1|IL6|MAPK3|MAPK9|MAPK10|TRAF3|TYK2
-2.536527683	-2.536157891	0	hsa04010	KEGG Pathway	MAPK signaling pathway	3.62328976	11	ANGPT2|ARAF|DUSP6|FOS|MAPK3|MAPK9|MAPK10|RAC3|TGFB2|MAPKAPK3|CACNG4
0	-2.981683457	0	R-HSA-2871796	Reactome Gene Sets	FCERI mediated MAPK activation	12.35212418	4	FOS|MAPK3|MAPK9|MAPK10
0	-3.521000078	0	hsa04658	KEGG Pathway	Th1 and Th2 cell differentiation	8.601581612	5	FOS|MAPK3|MAPK9|STAT6|TYK2
0	-3.478148643	0	R-HSA-168142	Reactome Gene Sets	Toll Like Receptor 10 (TLR10) Cascade	8.420495684	5	DUSP6|FOS|MAPK3|MAPK9|MAPKAPK3
0	-3.478148643	0	R-HSA-168176	Reactome Gene Sets	Toll Like Receptor 5 (TLR5) Cascade	8.420495684	5	DUSP6|FOS|MAPK3|MAPK9|MAPKAPK3
0	-3.478148643	0	R-HSA-975871	Reactome Gene Sets	MyD88 cascade initiated on plasma membrane	8.420495684	5	DUSP6|FOS|MAPK3|MAPK9|MAPKAPK3
0	-3.233457636	0	R-HSA-198725	Reactome Gene Sets	Nuclear Events (kinase and transcription factor activation)	8.099753563	5	DUSP6|EGR1|FOS|NAB2|MAPK3
0	-3.355453339	0	R-HSA-975138	Reactome Gene Sets	TRAF6 mediated induction of NFkB and MAP kinases upon TLR7/8 or 9 activation	7.920268217	5	DUSP6|FOS|MAPK3|MAPK9|MAPKAPK3
0	-3.355000518	0	R-HSA-9634638	Reactome Gene Sets	Estrogen-dependent nuclear events downstream of ESR-membrane signaling	19.99867725	3	BCL2|FOS|MAPK3
0	-3.335806439	0	R-HSA-975155	Reactome Gene Sets	MyD88 dependent cascade initiated on endosome	7.842618529	5	DUSP6|FOS|MAPK3|MAPK9|MAPKAPK3
-2.388959092	-3.263413856	0	hsa05166	KEGG Pathway	Human T-cell leukemia virus 1 infection	3.97032563	9	EGR1|FOS|ICAM1|IL6|MAPK3|MAPK9|MAPK10|TGFB2|PTTG1
0	-2.455153092	0	hsa05231	KEGG Pathway	Choline metabolism in cancer	5.988908695	6	FOS|PLD2|MAPK3|MAPK9|MAPK10|RAC3
0	-3.259346531	0	R-HSA-168181	Reactome Gene Sets	Toll Like Receptor 7/8 (TLR7/8) Cascade	7.54667066	5	DUSP6|FOS|MAPK3|MAPK9|MAPKAPK3
0	-3.204122481	0	R-HSA-168138	Reactome Gene Sets	Toll Like Receptor 9 (TLR9) Cascade	7.338964128	5	DUSP6|FOS|MAPK3|MAPK9|MAPKAPK3
-2.338378525	0	0	hsa04380	KEGG Pathway	Osteoclast differentiation	4.837195484	7	FOS|TNFRSF11B|MAPK3|MAPK9|MAPK10|TGFB2|TYK2
0	-3.108313867	0	hsa05171	KEGG Pathway	Coronavirus disease - COVID-19	3.736777064	9	C3AR1|FOS|IL6|MMP1|MAPK3|MAPK9|MAPK10|TRAF3|TYK2
0	-3.098699918	0	R-HSA-187037	Reactome Gene Sets	Signaling by NTRK1 (TRKA)	6.956061652	5	DUSP6|EGR1|FOS|MAPK3|MAPKAPK3
-2.719896226	-2.264869456	0	hsa05145	KEGG Pathway	Toxoplasmosis	5.293767507	6	BCL2|MAPK3|MAPK9|MAPK10|TGFB2|TYK2
0	-2.973967864	0	hsa05132	KEGG Pathway	Salmonella infection	4.461856279	7	BCL2|FOS|NCKAP1L|IL6|MAPK3|MAPK9|ACTR1B
-2.232838923	-2.832550565	0	hsa05133	KEGG Pathway	Pertussis	6.334422658	5	FOS|IL6|MAPK3|MAPK9|MAPK10
-2.248406226	-2.853311353	0	R-HSA-9009391	Reactome Gene Sets	Extra-nuclear estrogen signaling	8.311138597	4	BCL2|FOS|PRMT1|MAPK3
0	-2.816837552	0	R-HSA-166520	Reactome Gene Sets	Signaling by NTRKs	6.014639774	5	DUSP6|EGR1|FOS|MAPK3|MAPKAPK3
0	-2.135207835	0	hsa04935	KEGG Pathway	Growth hormone synthesis, secretion and action	4.859852138	6	FOS|IGFALS|MAPK3|MAPK9|MAPK10|PLCB1
-2.393135279	0	0	hsa05135	KEGG Pathway	Yersinia infection	4.29639102	6	FOS|IL6|MAPK3|MAPK9|MAPK10|RAC3
0	-2.527410845	0	hsa04921	KEGG Pathway	Oxytocin signaling pathway	5.160948967	5	FOS|MYLK|MAPK3|PLCB1|CACNG4
0	-2.03839554	0	hsa05130	KEGG Pathway	Pathogenic Escherichia coli infection	3.407482533	7	FOS|NCKAP1L|IL6|MYH11|MAPK3|MAPK9|MAPK10
0	-2.211436431	0	hsa04725	KEGG Pathway	Cholinergic synapse	5.516876482	4	BCL2|FOS|MAPK3|PLCB1
-5.502109681	-6.774438407	0	hsa04068	KEGG Pathway	FoxO signaling pathway	9.658803872	13	ARAF|S1PR1|PRMT1|IL6|MDM2|PCK2|PLK1|MAPK3|MAPK9|MAPK10|TGFB2|PLK2|CCNB3
0	-5.00423995	0	R-HSA-9768919	Reactome Gene Sets	NPAS4 regulates expression of target genes	29.08898509	4	FOS|MDM2|MAPK3|PLK2
-3.56100321	-2.983401641	0	hsa04071	KEGG Pathway	Sphingolipid signaling pathway	6.47980285	8	BCL2|S1PR1|PLD2|MAPK3|MAPK9|MAPK10|RAC3|PLCB1
0	-4.419010939	0	hsa01522	KEGG Pathway	Endocrine resistance	9.696328363	6	ARAF|BCL2|FOS|MDM2|MAPK3|MAPK9
0	-3.90326736	0	hsa05219	KEGG Pathway	Bladder cancer	12.05085286	5	ARAF|DAPK3|MDM2|MMP1|MAPK3
0	-4.227626289	0	R-HSA-9634815	Reactome Gene Sets	Transcriptional Regulation by NPAS4	18.82228447	4	FOS|MDM2|MAPK3|PLK2
-2.061689664	-3.587258408	0	R-HSA-9700206	Reactome Gene Sets	Signaling by ALK in cancer	8.888300999	5	ATIC|MDM2|MAPK3|MAPK9|TYK2
-2.061689664	-3.587258408	0	R-HSA-9725370	Reactome Gene Sets	Signaling by ALK fusions and activated point mutants	8.888300999	5	ATIC|MDM2|MAPK3|MAPK9|TYK2
0	-3.521000078	0	hsa04912	KEGG Pathway	GnRH signaling pathway	8.601581612	5	EGR1|PLD2|MAPK3|MAPK9|PLCB1
0	-3.259346531	0	hsa05215	KEGG Pathway	Prostate cancer	7.54667066	5	ARAF|BCL2|ERG|MDM2|MAPK3
0	-3.168259979	0	hsa04914	KEGG Pathway	Progesterone-mediated oocyte maturation	7.20673054	5	ARAF|PLK1|MAPK3|MAPK9|CCNB3
0	-2.607690045	0	R-HSA-5675221	Reactome Gene Sets	Negative regulation of MAPK pathway	11.16205242	3	ARAF|DUSP6|MAPK3
0	-2.363947457	0	hsa04625	KEGG Pathway	C-type lectin receptor signaling pathway	6.094834971	4	IL6|MDM2|MAPK3|MAPK9
0	-2.211436431	0	hsa04724	KEGG Pathway	Glutamatergic synapse	5.516876482	4	GRIK2|PLD2|MAPK3|PLCB1
0	-2.198425186	0	hsa04730	KEGG Pathway	Long-term depression	7.999470899	3	ARAF|MAPK3|PLCB1
0	-2.135207835	0	hsa04919	KEGG Pathway	Thyroid hormone signaling pathway	5.245554688	4	MDM2|PFKFB2|MAPK3|PLCB1
0	-2.106998532	0	R-HSA-5663202	Reactome Gene Sets	Diseases of signal transduction by growth factor receptors and second messengers	2.831671115	8	ARAF|ATIC|DUSP6|MDM2|MAPK3|MAPK9|TYK2|DKK2
0	-2.066135339	0	hsa04720	KEGG Pathway	Long-term potentiation	7.163705283	3	ARAF|MAPK3|PLCB1
0	-2.04851033	0	hsa05221	KEGG Pathway	Acute myeloid leukemia	7.058356676	3	ARAF|DUSP6|MAPK3
0	-2.012669575	0	R-HSA-112314	Reactome Gene Sets	Neurotransmitter receptors and postsynaptic signal transmission	3.883238301	5	GRIK2|MDM2|MAPK3|PLCB1|CACNG4
-4.239279532	-6.691092927	0	R-HSA-449147	Reactome Gene Sets	Signaling by Interleukins	4.560784314	21	BCL2|CD36|DUSP6|S1PR1|FOS|ICAM1|IL6|LIF|MMP1|SERPINB2|MAPK3|MAPK9|MAPK10|STAT6|TYK2|VCAM1|MAPKAPK3|IL1RL1|IL18BP|IL36A|IL31RA
-3.845934964	-7.629993999	0	R-HSA-6785807	Reactome Gene Sets	Interleukin-4 and Interleukin-13 signaling	10.25459366	11	BCL2|CD36|S1PR1|FOS|ICAM1|IL6|LIF|MMP1|STAT6|TYK2|VCAM1
-4.133194318	-5.074207281	0	R-HSA-1280215	Reactome Gene Sets	Cytokine Signaling in Immune system	3.438220133	27	BCL2|CD36|DUSP6|S1PR1|EGR1|FOS|GBP2|ICAM1|IL6|ISG20|LIF|MMP1|MT2A|TNFRSF11B|SERPINB2|MAPK3|MAPK9|MAPK10|STAT6|TRAF3|TYK2|VCAM1|MAPKAPK3|IL1RL1|IL18BP|IL36A|IL31RA
0	-4.846332589	0	R-HSA-6783589	Reactome Gene Sets	Interleukin-6 family signaling	26.664903	4	IL6|LIF|TYK2|IL31RA
0	-3.814711636	0	R-HSA-6788467	Reactome Gene Sets	IL-6-type cytokine receptor ligand interactions	28.2334267	3	LIF|TYK2|IL31RA
0	-3.744041213	0	R-HSA-6783783	Reactome Gene Sets	Interleukin-10 signaling	14.2212816	4	ICAM1|IL6|LIF|TYK2
0	-3.167257052	0	hsa04630	KEGG Pathway	JAK-STAT signaling pathway	5.713907785	6	BCL2|IL6|LIF|STAT6|TYK2|IL31RA
-2.559426779	-5.924009581	0	R-HSA-2559580	Reactome Gene Sets	Oxidative Stress Induced Senescence	8.766023614	11	FOS|H2BC5|H2BC3|MDM2|MAPK3|MAPK9|MAPK10|MAPKAPK3|H2AC6|H2BC21|H2BC11
-2.695494409	-5.476165748	0	R-HSA-2559583	Reactome Gene Sets	Cellular Senescence	6.241073271	12	FOS|H2BC5|H2BC3|IL6|MDM2|MAPK3|MAPK9|MAPK10|MAPKAPK3|H2AC6|H2BC21|H2BC11
-2.734135527	-4.140391527	0	R-HSA-2559582	Reactome Gene Sets	Senescence-Associated Secretory Phenotype (SASP)	7.121945475	8	FOS|H2BC5|H2BC3|IL6|MAPK3|H2AC6|H2BC21|H2BC11
-2.664334949	-2.211436431	0	R-HSA-9610379	Reactome Gene Sets	HCMV Late Events	6.814965066	8	H2BC5|H2BC3|H2AC6|H2AC17|H2BC21|H2BC11|VPS36|H2AC12
-2.512869728	-3.206602649	0	R-HSA-73728	Reactome Gene Sets	RNA Polymerase I Promoter Opening	9.562934851	6	H2BC5|H2BC3|MAPK3|H2AC6|H2BC21|H2BC11
-2.010251407	-2.536147019	0	R-HSA-3214815	Reactome Gene Sets	HDACs deacetylate histones	7.358712279	7	H2BC5|H2BC3|H2AC6|H2AC17|H2BC21|H2BC11|H2AC12
0	-2.63711282	0	R-HSA-9821993	Reactome Gene Sets	Replacement of protamines by nucleosomes in the male pronucleus	11.76392779	5	H2BC5|H2BC3|PRM1|H2BC21|H2BC11
-2.438607915	0	0	R-HSA-9818564	Reactome Gene Sets	Epigenetic regulation of gene expression by MLL3 and MLL4 complexes	5.899521998	8	CD36|FABP4|H2BC5|H2BC3|H2AC6|H2BC21|H2BC11|MGLL
-2.438607915	0	0	R-HSA-9841922	Reactome Gene Sets	MLL4 and MLL3 complexes regulate expression of PPARG target genes in adipogenesis and hepatic steatosis	5.899521998	8	CD36|FABP4|H2BC5|H2BC3|H2AC6|H2BC21|H2BC11|MGLL
-2.438607915	0	0	R-HSA-9851695	Reactome Gene Sets	Epigenetic regulation of adipogenesis genes by MLL3 and MLL4 complexes	5.899521998	8	CD36|FABP4|H2BC5|H2BC3|H2AC6|H2BC21|H2BC11|MGLL
0	-2.939484359	0	R-HSA-2299718	Reactome Gene Sets	Condensation of Prophase Chromosomes	8.121944668	6	H2BC5|H2BC3|PLK1|H2AC6|H2BC21|H2BC11
0	-2.939484359	0	R-HSA-606279	Reactome Gene Sets	Deposition of new CENPA-containing nucleosomes at the centromere	8.121944668	6	H2BC5|H2BC3|H2AC6|H2BC21|H2BC11|CENPH
0	-2.939484359	0	R-HSA-774815	Reactome Gene Sets	Nucleosome assembly	8.121944668	6	H2BC5|H2BC3|H2AC6|H2BC21|H2BC11|CENPH
0	-3.645723734	0	R-HSA-195258	Reactome Gene Sets	RHO GTPase Effectors	3.637435342	12	CENPE|H2BC5|H2BC3|NCKAP1L|MYH11|MYLK|PLK1|MAPK3|H2AC6|H2BC21|H2BC11|CENPH
0	-3.406563317	0	R-HSA-5688426	Reactome Gene Sets	Deubiquitination	3.896010495	11	H2BC5|H2BC3|MDM2|TNFAIP3|TRAF3|USP5|H2AC6|H2AC17|H2BC21|H2BC11|H2AC12
0	-3.838902933	0	R-HSA-194315	Reactome Gene Sets	Signaling by Rho GTPases	3.190672154	14	ALDH3A2|CENPE|NIPSNAP2|H2BC5|NCKAP1L|MYLK|PLD2|PLK1|MAPK3|H2BC21|H2BC11|DLC1|CENPH|MSI2
-2.27551531	-3.442717389	0	R-HSA-9018519	Reactome Gene Sets	Estrogen-dependent gene expression	5.305610387	8	BCL2|FOS|H2BC5|H2BC3|PRMT1|H2AC6|H2BC21|H2BC11
0	-2.879288637	0	hsa05034	KEGG Pathway	Alcoholism	4.656298121	9	ARAF|H2BC5|H2BC3|MAPK3|H2AC6|H2AC17|H2BC21|H2BC11|H2AC12
0	-2.396319353	0	R-HSA-171306	Reactome Gene Sets	Packaging Of Telomere Ends	9.687940536	5	H2BC5|H2BC3|H2AC6|H2BC21|H2BC11
0	-3.73824636	0	R-HSA-9716542	Reactome Gene Sets	Signaling by Rho GTPases, Miro GTPases and RHOBTB3	3.119570824	14	ALDH3A2|CENPE|NIPSNAP2|H2BC5|NCKAP1L|MYLK|PLD2|PLK1|MAPK3|H2BC21|H2BC11|DLC1|CENPH|MSI2
0	-2.822167288	0	hsa04613	KEGG Pathway	Neutrophil extracellular trap formation	4.537515006	9	H2BC5|H2BC3|MAPK3|H2AC6|H2AC17|H2BC21|H2BC11|PLCB1|H2AC12

_LogP_HS_CON	_LogP_HS_REC	_LogP_REC_CON	GO	Category	Description	Enrichment	#GeneInGOAndHitList	Hits
0	-2.766780852	0	R-HSA-9764265	Reactome Gene Sets	Regulation of CDH1 Expression and Function	4.424641498	9	ACTA2|ANK3|H2BC5|H2BC3|MDM2|MAPK3|H2AC6|H2BC21|H2BC11
0	-2.766780852	0	R-HSA-9764274	Reactome Gene Sets	Regulation of Expression and Function of Type I Classical Cadherins	4.424641498	9	ACTA2|ANK3|H2BC5|H2BC3|MDM2|MAPK3|H2AC6|H2BC21|H2BC11
0	-2.303940833	0	R-HSA-110330	Reactome Gene Sets	Recognition and association of DNA glycosylase with site containing an affected purine	8.983363042	5	H2BC5|H2BC3|H2AC6|H2BC21|H2BC11
0	-2.303940833	0	R-HSA-110331	Reactome Gene Sets	Cleavage of the damaged purine	8.983363042	5	H2BC5|H2BC3|H2AC6|H2BC21|H2BC11
0	-2.303940833	0	R-HSA-73927	Reactome Gene Sets	Depurination	8.983363042	5	H2BC5|H2BC3|H2AC6|H2BC21|H2BC11
0	-2.745092417	0	R-HSA-5689880	Reactome Gene Sets	Ub-specific processing proteases	4.381048971	9	H2BC5|H2BC3|MDM2|USP5|H2AC6|H2AC17|H2BC21|H2BC11|H2AC12
0	-3.593012425	0	R-HSA-68886	Reactome Gene Sets	M Phase	3.940626059	10	CENPE|H2BC5|PLK1|MAPK3|H2BC21|H2BC11|PTTG1|NCAPD2|CENPH|CC2D1B
-2.148861266	0	0	R-HSA-9609646	Reactome Gene Sets	HCMV Infection	4.879851529	8	H2BC5|H2BC3|H2AC6|H2AC17|H2BC21|H2BC11|VPS36|H2AC12
-2.121318488	0	0	R-HSA-9917777	Reactome Gene Sets	Epigenetic regulation by WDR5-containing histone modifying complexes	4.791126956	8	CD36|FABP4|H2BC5|H2BC3|H2AC6|H2BC21|H2BC11|MGLL
0	-2.198425186	0	R-HSA-110328	Reactome Gene Sets	Recognition and association of DNA glycosylase with site containing an affected pyrimidine	8.234749455	5	H2BC5|H2BC3|H2AC6|H2BC21|H2BC11
0	-2.198425186	0	R-HSA-110329	Reactome Gene Sets	Cleavage of the damaged pyrimidine	8.234749455	5	H2BC5|H2BC3|H2AC6|H2BC21|H2BC11
0	-2.198425186	0	R-HSA-73928	Reactome Gene Sets	Depyrimidination	8.234749455	5	H2BC5|H2BC3|H2AC6|H2BC21|H2BC11
-2.022877066	0	0	R-HSA-5625740	Reactome Gene Sets	RHO GTPases activate PKNs	6.375289901	6	H2BC5|H2BC3|MYH11|H2AC6|H2BC21|H2BC11
-2.022877066	-2.552937023	0	R-HSA-9764725	Reactome Gene Sets	Negative Regulation of CDH1 Gene Transcription	6.375289901	6	H2BC5|H2BC3|MAPK3|H2AC6|H2BC21|H2BC11
0	-2.600232698	0	R-HSA-9759476	Reactome Gene Sets	Regulation of Homotypic Cell-Cell Adhesion	4.098400651	9	ACTA2|ANK3|H2BC5|H2BC3|MDM2|MAPK3|H2AC6|H2BC21|H2BC11
-2.010251407	-2.536147019	0	R-HSA-8936459	Reactome Gene Sets	RUNX1 regulates genes involved in megakaryocyte differentiation and platelet function	6.307467668	6	H2BC5|H2BC3|PRMT1|H2AC6|H2BC21|H2BC11
0	-2.158932722	0	R-HSA-73929	Reactome Gene Sets	Base-Excision Repair, AP Site Formation	7.969112376	5	H2BC5|H2BC3|H2AC6|H2BC21|H2BC11
-2.40541322	-3.286529935	0	R-HSA-8939211	Reactome Gene Sets	ESR-mediated signaling	4.00609433	9	BCL2|FOS|H2BC5|H2BC3|PRMT1|MAPK3|H2AC6|H2BC21|H2BC11
0	-2.120839896	0	R-HSA-5334118	Reactome Gene Sets	DNA methylation	7.720077614	5	H2BC5|H2BC3|H2AC6|H2BC21|H2BC11
0	-2.084059006	0	R-HSA-5625886	Reactome Gene Sets	Activated PKN1 stimulates transcription of AR (androgen receptor) regulated genes KLK2 and KLK3	7.486135868	5	H2BC5|H2BC3|H2AC6|H2BC21|H2BC11
0	-2.718982312	0	R-HSA-73886	Reactome Gene Sets	Chromosome Maintenance	4.940849673	7	H2BC5|H2BC3|H2AC6|H2BC21|H2BC11|ANKRD28|CENPH
0	-2.066135339	0	R-HSA-427359	Reactome Gene Sets	SIRT1 negatively regulates rRNA expression	7.374402497	5	H2BC5|H2BC3|H2AC6|H2BC21|H2BC11
0	-2.066135339	0	R-HSA-9670095	Reactome Gene Sets	Inhibition of DNA recombination at telomere	7.374402497	5	H2BC5|H2BC3|H2AC6|H2BC21|H2BC11
0	-2.705471749	0	R-HSA-68875	Reactome Gene Sets	Mitotic Prophase	4.905808186	7	H2BC5|H2BC3|PLK1|MAPK3|H2AC6|H2BC21|H2BC11
0	-2.04851033	0	R-HSA-68616	Reactome Gene Sets	Assembly of the ORC complex at the origin of replication	7.265955402	5	H2BC5|H2BC3|H2AC6|H2BC21|H2BC11
0	-2.39366706	0	R-HSA-9764560	Reactome Gene Sets	Regulation of CDH1 Gene Transcription	5.756329716	6	H2BC5|H2BC3|MAPK3|H2AC6|H2BC21|H2BC11
0	-2.014121186	0	R-HSA-9821002	Reactome Gene Sets	Chromatin modifications during the maternal to zygotic transition (MZT)	7.058356676	5	H2BC5|H2BC3|H2AC6|H2BC21|H2BC11
0	-2.014121186	0	R-HSA-9843970	Reactome Gene Sets	Regulation of endogenous retroelements by the Human Silencing Hub (HUSH) complex	7.058356676	5	H2BC5|H2BC3|H2AC6|H2BC21|H2BC11
0	-2.385672616	0	R-HSA-418990	Reactome Gene Sets	Adherens junctions interactions	3.705637255	9	ACTA2|ANK3|H2BC5|H2BC3|MDM2|MAPK3|H2AC6|H2BC21|H2BC11
0	-2.306425719	0	R-HSA-73854	Reactome Gene Sets	RNA Polymerase I Promoter Clearance	5.43946753	6	H2BC5|H2BC3|MAPK3|H2AC6|H2BC21|H2BC11
0	-2.278576151	0	R-HSA-73864	Reactome Gene Sets	RNA Polymerase I Transcription	5.341459106	6	H2BC5|H2BC3|MAPK3|H2AC6|H2BC21|H2BC11
0	-2.264869456	0	R-HSA-157579	Reactome Gene Sets	Telomere Maintenance	5.293767507	6	H2BC5|H2BC3|H2AC6|H2BC21|H2BC11|ANKRD28
0	-2.88216592	0	R-HSA-1640170	Reactome Gene Sets	Cell Cycle	2.806831895	12	CENPE|H2BC5|MDM2|PLK1|MAPK3|H2BC21|H2BC11|PTTG1|NCAPD2|ANKRD28|CENPH|CC2D1B
0	-2.134330139	0	R-HSA-3247509	Reactome Gene Sets	Chromatin modifying enzymes	3.28174517	9	H2BC5|H2BC3|PRMT1|H2AC6|H2AC17|H2BC21|H2BC11|DOT1L|H2AC12
0	-2.723662371	0	hsa05203	KEGG Pathway	Viral carcinogenesis	4.682617112	6	H2BC5|MDM2|MAPK3|TRAF3|H2BC21|H2BC11
0	-2.126843762	0	R-HSA-421270	Reactome Gene Sets	Cell-cell junction organization	3.269679931	9	ACTA2|ANK3|H2BC5|H2BC3|MDM2|MAPK3|H2AC6|H2BC21|H2BC11
0	-2.659983406	0	R-HSA-69620	Reactome Gene Sets	Cell Cycle Checkpoints	3.929564652	7	CENPE|H2BC5|MDM2|PLK1|H2BC21|H2BC11|CENPH
0	-2.572858498	0	R-HSA-69278	Reactome Gene Sets	Cell Cycle, Mitotic	2.887895632	10	CENPE|H2BC5|PLK1|MAPK3|H2BC21|H2BC11|PTTG1|NCAPD2|CENPH|CC2D1B
0	-2.025657625	0	R-HSA-4839726	Reactome Gene Sets	Chromatin organization	3.109625668	9	H2BC5|H2BC3|PRMT1|H2AC6|H2AC17|H2BC21|H2BC11|DOT1L|H2AC12
0	-2.04851033	0	R-HSA-5693571	Reactome Gene Sets	Nonhomologous End-Joining (NHEJ)	5.812764321	4	H2BC5|H2BC3|H2BC21|H2BC11
-6.05553813	0	0	hsa04350	KEGG Pathway	TGF-beta signaling pathway	13.64706641	7	BMP7|INHBA|MAPK3|TGFB2|GDF5|FST|GREM1
-4.19156672	-3.814711636	0	R-HSA-1502540	Reactome Gene Sets	Signaling by Activin	37.84480601	3	INHBA|MAPK3|FST
-2.327691876	0	0	hsa04550	KEGG Pathway	Signaling pathways regulating pluripotency of stem cells	5.957052797	4	INHBA|LIF|MAPK3|WNT5A
-2.186530137	0	0	R-HSA-9006936	Reactome Gene Sets	Signaling by TGFB family members	5.429212676	4	INHBA|MAPK3|TGFB2|FST
0	0	-5.499868654	hsa04723	KEGG Pathway	Retrograde endocannabinoid signaling	22.05865188	5	CNR1|NDUFB4|MAPK10|NDUFA11|SLC32A1
0	-2.503626047	-3.404331471	hsa05208	KEGG Pathway	Chemical carcinogenesis - reactive oxygen species	4.353171518	10	ARAF|CYC1|FOS|GSTA3|NDUFB4|PLD2|MAPK3|MAPK9|MAPK10|NDUFA11
-2.11398997	-3.606676664	0	hsa05022	KEGG Pathway	Pathways of neurodegeneration - multiple diseases	3.068850729	15	ARAF|BCL2|CYC1|FUS|IL6|NDUFB4|MAPK3|MAPK9|MAPK10|PSMD4|WNT5A|ACTR1B|PLCB1|DKK2|NDUFA11
-2.608572302	-2.47667206	-2.533555544	hsa05010	KEGG Pathway	Alzheimer disease	3.285475486	13	ARAF|CYC1|IL6|NDUFB4|MAPK3|MAPK9|MAPK10|PSMD4|WNT5A|PLCB1|DKK2|SLC39A8|NDUFA11
0	-2.021192361	-2.424651361	hsa05415	KEGG Pathway	Diabetic cardiomyopathy	4.33830703	9	CD36|CYC1|GYS1|NDUFB4|MAPK9|MAPK10|TGFB2|PLCB1|NDUFA11
0	0	-3.11581619	hsa05012	KEGG Pathway	Parkinson disease	9.702550939	4	NDUFB4|MAPK10|SLC39A8|NDUFA11
0	0	-2.752113759	hsa04932	KEGG Pathway	Non-alcoholic fatty liver disease	4.405853212	7	CYC1|FOS|IL6|NDUFB4|MAPK9|MAPK10|NDUFA11
-2.002256882	-2.082664307	-2.059547486	hsa05020	KEGG Pathway	Prion disease	3.199111299	9	CYC1|EGR1|IL6|NDUFB4|MAPK3|MAPK9|MAPK10|PSMD4|NDUFA11
0	0	-2.25965341	hsa04714	KEGG Pathway	Thermogenesis	8.391674376	3	CNR1|NDUFB4|NDUFA11
0	0	-2.148036549	R-HSA-1428517	Reactome Gene Sets	Aerobic respiration and respiratory electron transport	7.643579373	3	NDUFB4|DMAC2L|NDUFA11
0	-5.224952229	0	hsa05169	KEGG Pathway	Epstein-Barr virus infection	7.058356676	9	BCL2|ICAM1|IL6|MDM2|MAPK9|PSMD4|TNFAIP3|TRAF3|TYK2
-2.705471749	-4.574486121	0	hsa04621	KEGG Pathway	NOD-like receptor signaling pathway	5.228412353	10	BCL2|GBP2|IL6|MAPK3|MAPK9|MAPK10|TNFAIP3|TRAF3|TYK2|PLCB1
0	-4.546759291	0	hsa05162	KEGG Pathway	Measles	8.05702105	7	BCL2|FOS|IL6|MAPK9|TNFAIP3|TRAF3|TYK2
-2.177009081	-3.292929645	0	hsa04217	KEGG Pathway	Necroptosis	5.593414724	9	BCL2|MAPK9|MAPK10|STAT6|TNFAIP3|TYK2|H2AC6|H2AC17|H2AC12
0	-3.278150676	0	hsa04064	KEGG Pathway	NF-kappa B signaling pathway	7.618543714	5	BCL2|ICAM1|TNFAIP3|TRAF3|VCAM1
-5.135888781	0	0	R-HSA-109582	Reactome Gene Sets	Hemostasis	4.489373123	13	ANGPT2|ITGA2|MMP1|SERPINB2|SERPINB8|MAPK3|TGFB2|THBD|PHACTR2|MGLL|SLC7A11|SCCPDH|MICAL1
-2.427095287	0	0	R-HSA-202733	Reactome Gene Sets	Cell surface interactions at the vascular wall	6.354189651	4	ANGPT2|MMP1|THBD|SLC7A11
0	-4.923389969	0	R-HSA-112409	Reactome Gene Sets	RAF-independent MAPK1/3 activation	27.82424661	4	DUSP6|IL6|MAPK3|TYK2
0	-4.554020491	0	R-HSA-110056	Reactome Gene Sets	MAPK3 (ERK1) activation	47.9968254	3	IL6|MAPK3|TYK2
-2.202357259	-2.791911277	0	hsa01521	KEGG Pathway	EGFR tyrosine kinase inhibitor resistance	7.999470899	4	ARAF|BCL2|IL6|MAPK3
-2.777085989	0	0	hsa05152	KEGG Pathway	Tuberculosis	5.891590679	5	BCL2|IL6|MAPK3|MAPK10|TGFB2
-2.748519541	-2.292427343	0	hsa04066	KEGG Pathway	HIF-1 signaling pathway	7.798323662	4	ANGPT2|BCL2|IL6|MAPK3
0	-2.434039817	0	R-HSA-9658195	Reactome Gene Sets	Leishmania infection	4.907650859	5	C3AR1|NCKAP1L|IL6|MAPK3|PLK2
0	-2.434039817	0	R-HSA-9824443	Reactome Gene Sets	Parasitic Infection Pathways	4.907650859	5	C3AR1|NCKAP1L|IL6|MAPK3|PLK2
0	-2.324787795	0	hsa05164	KEGG Pathway	Influenza A	4.623971618	5	ICAM1|IL6|MAPK3|TRAF3|TYK2
-2.265323346	0	0	hsa05226	KEGG Pathway	Gastric cancer	5.718770686	4	BCL2|MAPK3|TGFB2|WNT5A
-2.14438283	0	0	hsa04151	KEGG Pathway	PI3K-Akt signaling pathway	3.554484542	6	ANGPT2|BCL2|IL6|ITGA2|PCK2|MAPK3
-4.082884923	0	0	hsa04060	KEGG Pathway	Cytokine-cytokine receptor interaction	4.31080844	13	ACVRL1|BMP7|CCR7|IL6|INHBA|LIF|TNFRSF11B|TGFB2|GDF5|IL1RL1|GDF15|IL36A|IL31RA
-3.163295296	0	0	R-HSA-2129379	Reactome Gene Sets	Molecules associated with elastic fibres	17.38815411	3	BMP7|TGFB2|GDF5
-3.061621101	0	0	hsa04390	KEGG Pathway	Hippo signaling pathway	6.829742061	5	BMP7|CCN2|TGFB2|WNT5A|GDF5
-2.942042001	0	0	R-HSA-1566948	Reactome Gene Sets	Elastic fibre formation	14.62185687	3	BMP7|TGFB2|GDF5
-2.412861901	0	0	R-HSA-1474244	Reactome Gene Sets	Extracellular matrix organization	4.046300013	6	ACTA2|BMP7|ITGA2|MMP1|TGFB2|GDF5
0	-4.08043712	0	hsa05143	KEGG Pathway	African trypanosomiasis	17.2961533	4	ICAM1|IL6|VCAM1|PLCB1
0	-3.565490883	0	hsa05144	KEGG Pathway	Malaria	9.881699346	5	CD36|ICAM1|IL6|TGFB2|VCAM1
-2.917120524	0	0	hsa05410	KEGG Pathway	Hypertrophic cardiomyopathy	8.664804069	4	IL6|ITGA2|TGFB2|CACNG4
-2.543666361	-2.544163986	0	R-HSA-71387	Reactome Gene Sets	Metabolism of carbohydrates and carbohydrate derivatives	3.635407786	11	GYS1|HAS3|MAN2C1|NAGLU|PCK2|PFKFB2|HS3ST3A1|GLCE|CEMIP|SLC37A2|CHSY3
0	-3.545474172	0	hsa05202	KEGG Pathway	Transcriptional misregulation in cancer	5.571770776	7	DUSP6|ERG|FUS|HHEX|IL6|MDM2|DOT1L
-3.40564653	0	0	hsa04510	KEGG Pathway	Focal adhesion	6.338538937	6	BCL2|ITGA2|MYLK|MAPK3|MAPK10|RAC3
0	0	-2.383906734	hsa04015	KEGG Pathway	Rap1 signaling pathway	9.302091879	3	ANGPT2|CNR1|RAC3
-2.381599058	0	0	hsa04664	KEGG Pathway	Fc epsilon RI signaling pathway	9.32408264	3	MAPK3|MAPK10|RAC3
-2.324867693	0	0	hsa04810	KEGG Pathway	Regulation of actin cytoskeleton	4.621851308	5	ITGA2|MYH11|MYLK|MAPK3|RAC3
0	0	-2.244430751	hsa04014	KEGG Pathway	Ras signaling pathway	8.285896967	3	ANGPT2|MAPK10|RAC3
-2.438607915	-2.802494287	0	hsa04270	KEGG Pathway	Vascular smooth muscle contraction	5.162081748	7	ACTA2|ARAF|EDN1|MYH11|MYLK|MAPK3|PLCB1
-2.370962778	-2.718982312	0	hsa04371	KEGG Pathway	Apelin signaling pathway	4.940849673	7	ACTA2|CCN2|EGR1|MYL4|MYLK|MAPK3|PLCB1
-2.913555359	0	0	R-HSA-445355	Reactome Gene Sets	Smooth Muscle Contraction	14.29692671	3	ACTA2|MYH11|MYLK
-3.31092373	0	0	R-HSA-352230	Reactome Gene Sets	Amino acid transport across the plasma membrane	19.49580916	3	SLC6A6|SLC7A11|SLC36A4
-2.218902657	0	-2.204842688	R-HSA-425407	Reactome Gene Sets	SLC-mediated transmembrane transport	4.35881912	5	SLC6A6|SLC7A11|SLC50A1|SLC39A8|SLC36A4
0	-2.306425719	0	hsa04931	KEGG Pathway	Insulin resistance	5.43946753	6	CD36|GYS1|IL6|PCK2|MAPK9|MAPK10
0	-2.665606371	0	hsa04936	KEGG Pathway	Alcoholic liver disease	5.555188125	5	ALDH3A2|C3AR1|IL6|MAPK9|TRAF3
-2.858573021	-2.497060227	0	hsa00620	KEGG Pathway	Pyruvate metabolism	8.409956891	4	ALDH3A2|HAGH|PCK2|GRHPR
-2.756301246	0	0	hsa04360	KEGG Pathway	Axon guidance	5.82755165	5	BMP7|MAPK3|RAC3|WNT5A|SEMA3C
-2.042105312	0	0	hsa04310	KEGG Pathway	Wnt signaling pathway	3.975396289	7	MAPK9|MAPK10|RAC3|WNT5A|PLCB1|DKK2|PRICKLE1
-2.217491135	0	0	R-HSA-4086400	Reactome Gene Sets	PCP/CE pathway	8.143819014	3	RAC3|WNT5A|PRICKLE1
0	-2.051677127	0	R-HSA-114608	Reactome Gene Sets	Platelet degranulation	4.596139231	6	A2M|CD36|SELENOP|TGFB2|PHACTR2|SCCPDH
-2.106482097	0	0	R-HSA-76002	Reactome Gene Sets	Platelet activation, signaling and aggregation	3.017312778	8	A2M|CD36|MAPK3|SELENOP|TGFB2|PHACTR2|MGLL|SCCPDH
0	-2.519839111	0	R-HSA-556833	Reactome Gene Sets	Metabolism of lipids	2.536159862	12	ABCA1|ALDH3A2|CD36|PLD2|ST3GAL5|THRAP3|LDLRAP1|ANKRD1|LPCAT2|MFSD2A|OSBPL1A|GDPD1
0	-2.396319353	0	hsa04979	KEGG Pathway	Cholesterol metabolism	9.411142235	3	ABCA1|CD36|LDLRAP1
0	-2.198414395	0	R-HSA-1989781	Reactome Gene Sets	PPARA activates gene expression	5.469723692	4	ABCA1|CD36|THRAP3|ANKRD1
0	-2.172757563	0	R-HSA-400206	Reactome Gene Sets	Regulation of lipid metabolism by PPARalpha	5.377795563	4	ABCA1|CD36|THRAP3|ANKRD1
0	-2.420667398	0	hsa00565	KEGG Pathway	Ether lipid metabolism	9.599365079	3	PLD2|LPCAT2|GDPD1
-2.264198871	0	0	hsa03320	KEGG Pathway	PPAR signaling pathway	8.465285554	3	FABP4|MMP1|PCK2

_LogP_HS_CON	_LogP_HS_REC	_LogP_REC_CON	GO	Category	Description	Enrichment	#GeneInGOAndHitList	Hits
-2.232838923	0	0	R-HSA-3214858	Reactome Gene Sets	RMTs methylate histone arginines	8.24822695	3	PRMT1|H2AC6|H2AC12
