# Supplementary material for: Establishment and characterization of Hanwoo cumulus cell line for heat stress studies
Source: Anim Biosci. 2026 Jun 15;39(7):250896. doi: 10.5713/ab.250896 (PMC13353149; doi:10.5713/ab.250896)
Supplement: Supplementary file 15 [file ab-250896-Supplementary-15.pdf]

Supplement 15. Cumulus upregulated DEP pathway enrichment (KEGG and Reactome)

<table>
<tr><th>_LogP_HS_CON</th><th>_LogP_HS_REC</th><th>_LogP_REC_CON</th><th>GO</th><th>Category</th><th>Description</th><th>Enrichment</th><th>#GeneInGOAndHitList</th><th>Hits</th></tr>
<tr><td>-12.67311116</td><td>0</td><td>-10.54129963</td><td>R-HSA-3371556</td><td>Reactome Gene Sets</td><td>Cellular response to heat stress</td><td>70.48484848</td><td>8</td><td>CRYAB|HSPA1L|HSPA6|HSP90AA1|DNAJB1|BAG3|HSPH1|HSPB8</td></tr>
<tr><td>-10.32783839</td><td>0</td><td>-5.460121568</td><td>hsa04141</td><td>KEGG Pathway</td><td>Protein processing in endoplasmic reticulum</td><td>36.27290448</td><td>8</td><td>CRYAB|DNAJB2|DNAJA1|HSPA1L|HSPA6|HSP90AA1|DNAJB1|HSPH1</td></tr>
<tr><td>-9.942508249</td><td>0</td><td>-7.518571043</td><td>R-HSA-3371571</td><td>Reactome Gene Sets</td><td>HSF1-dependent transactivation</td><td>161.5277778</td><td>5</td><td>CRYAB|HSPA1L|HSP90AA1|DNAJB1|HSPB8</td></tr>
<tr><td>-7.962462253</td><td>0</td><td>0</td><td>R-HSA-3371497</td><td>Reactome Gene Sets</td><td>HSP90 chaperone cycle for steroid hormone receptors (SHR) in the presence of ligand</td><td>68.01169591</td><td>5</td><td>DNAJA1|HSPA1L|HSP90AA1|DNAJB1|DNAJA4</td></tr>
<tr><td>-7.570815979</td><td>0</td><td>-7.457851169</td><td>R-HSA-3371453</td><td>Reactome Gene Sets</td><td>Regulation of HSF1-mediated heat shock response</td><td>57.00980392</td><td>5</td><td>HSPA1L|HSPA6|DNAJB1|BAG3|HSPH1</td></tr>
<tr><td>-6.145732083</td><td>0</td><td>0</td><td>R-HSA-3371568</td><td>Reactome Gene Sets</td><td>Attenuation phase</td><td>166.1428571</td><td>3</td><td>HSPA1L|HSP90AA1|DNAJB1</td></tr>
<tr><td>-3.766499065</td><td>0</td><td>-4.96685687</td><td>hsa05417</td><td>KEGG Pathway</td><td>Lipid and atherosclerosis</td><td>10.53693748</td><td>7</td><td>BID|HSPA1L|HSPA6|HSP90AA1|IL6|IRAK1|OLR1</td></tr>
<tr><td>-3.113750014</td><td>0</td><td>-4.420867006</td><td>hsa05162</td><td>KEGG Pathway</td><td>Measles</td><td>21.22337252</td><td>4</td><td>BID|HSPA1L|HSPA6|IRAK1</td></tr>
<tr><td>-4.391462881</td><td>0</td><td>-4.284028871</td><td>hsa04010</td><td>KEGG Pathway</td><td>MAPK signaling pathway</td><td>12.92222222</td><td>5</td><td>EREG|HSPA1L|HSPA6|HSPB1|IRAK1</td></tr>
<tr><td>-4.147714428</td><td>0</td><td>-4.082124696</td><td>hsa04213</td><td>KEGG Pathway</td><td>Longevity regulating pathway - multiple species</td><td>37.51612903</td><td>3</td><td>CRYAB|HSPA1L|HSPA6</td></tr>
<tr><td>-3.801740185</td><td>0</td><td>0</td><td>hsa04612</td><td>KEGG Pathway</td><td>Antigen processing and presentation</td><td>28.71604938</td><td>3</td><td>HSPA1L|HSPA6|HSP90AA1</td></tr>
<tr><td>-3.38699857</td><td>0</td><td>-3.322478267</td><td>hsa05145</td><td>KEGG Pathway</td><td>Toxoplasmosis</td><td>20.76785714</td><td>3</td><td>HSPA1L|HSPA6|IRAK1</td></tr>
<tr><td>-3.113750014</td><td>0</td><td>0</td><td>hsa04915</td><td>KEGG Pathway</td><td>Estrogen signaling pathway</td><td>16.73381295</td><td>3</td><td>HSPA1L|HSPA6|HSP90AA1</td></tr>
<tr><td>-9.719885945</td><td>-2.430634878</td><td>-6.047024729</td><td>R-HSA-2262752</td><td>Reactome Gene Sets</td><td>Cellular responses to stress</td><td>7.408248265</td><td>18</td><td>ALB|CDKN2A|CEBPB|CRYAB|DNAJA1|HSPA1L|HSPA6|HSP90AA1|DNAJB1|IL6|COX5A|BAG3|HSPH1|UBE2C|TXN2|HSPB8|DNAJA4|CRTC3</td></tr>
<tr><td>0</td><td>-7.73819506</td><td>0</td><td>R-HSA-114608</td><td>Reactome Gene Sets</td><td>Platelet degranulation</td><td>35.16046512</td><td>6</td><td>A2M|ALB|ORM1|SERPINA1|SERPINF2|TF</td></tr>
<tr><td>0</td><td>-7.63926246</td><td>0</td><td>R-HSA-76005</td><td>Reactome Gene Sets</td><td>Response to elevated platelet cytosolic Ca2+</td><td>33.84850746</td><td>6</td><td>A2M|ALB|ORM1|SERPINA1|SERPINF2|TF</td></tr>
<tr><td>0</td><td>-6.501230983</td><td>0</td><td>R-HSA-8957275</td><td>Reactome Gene Sets</td><td>Post-translational protein phosphorylation</td><td>34.99768519</td><td>5</td><td>AFP|ALB|IL6|SERPINA1|TF</td></tr>
<tr><td>0</td><td>-6.185311305</td><td>0</td><td>R-HSA-381426</td><td>Reactome Gene Sets</td><td>Regulation of Insulin-like Growth Factor (IGF) transport and uptake by Insulin-like Growth Factor Binding Proteins (IGFBPs)</td><td>30.238</td><td>5</td><td>AFP|ALB|IL6|SERPINA1|TF</td></tr>
<tr><td>0</td><td>-5.92152787</td><td>0</td><td>R-HSA-76002</td><td>Reactome Gene Sets</td><td>Platelet activation, signaling and aggregation</td><td>17.31183206</td><td>6</td><td>A2M|ALB|ORM1|SERPINA1|SERPINF2|TF</td></tr>
<tr><td>0</td><td>-3.809133134</td><td>0</td><td>R-HSA-109582</td><td>Reactome Gene Sets</td><td>Hemostasis</td><td>7.303864734</td><td>6</td><td>A2M|ALB|ORM1|SERPINA1|SERPINF2|TF</td></tr>
<tr><td>0</td><td>-3.662205028</td><td>0</td><td>hsa04610</td><td>KEGG Pathway</td><td>Complement and coagulation cascades</td><td>25.77102273</td><td>3</td><td>A2M|SERPINA1|SERPINF2</td></tr>
<tr><td>0</td><td>-2.608575479</td><td>0</td><td>R-HSA-382551</td><td>Reactome Gene Sets</td><td>Transport of small molecules</td><td>5.264275766</td><td>5</td><td>A2M|ALB|APOD|TF|SLC43A2</td></tr>
<tr><td>0</td><td>-4.722156618</td><td>0</td><td>R-HSA-8853884</td><td>Reactome Gene Sets</td><td>Transcriptional Regulation by VENTX</td><td>33.34767025</td><td>4</td><td>CDKN2A|CEBPB|IL6|UBE2C</td></tr>
<tr><td>0</td><td>-3.365703794</td><td>0</td><td>R-HSA-2559582</td><td>Reactome Gene Sets</td><td>Senescence-Associated Secretory Phenotype (SASP)</td><td>11.71674901</td><td>4</td><td>CDKN2A|CEBPB|IL6|UBE2C</td></tr>
<tr><td>0</td><td>-2.928747677</td><td>0</td><td>hsa04932</td><td>KEGG Pathway</td><td>Non-alcoholic fatty liver disease</td><td>14.44490446</td><td>3</td><td>BID|IL6|COX5A</td></tr>
<tr><td>0</td><td>-2.691808669</td><td>0</td><td>R-HSA-2559583</td><td>Reactome Gene Sets</td><td>Cellular Senescence</td><td>11.93605263</td><td>3</td><td>CDKN2A|IL6|UBE2C</td></tr>
<tr><td>0</td><td>-2.478891137</td><td>0</td><td>hsa05163</td><td>KEGG Pathway</td><td>Human cytomegalovirus infection</td><td>10.03473451</td><td>3</td><td>BID|CDKN2A|IL6</td></tr>
<tr><td>0</td><td>0</td><td>-2.43133955</td><td>R-HSA-6798695</td><td>Reactome Gene Sets</td><td>Neutrophil degranulation</td><td>5.534294212</td><td>8</td><td>HSPA6|HSP90AA1|LGALS3|OLR1|ORM1|SERPINA1|PKM|TTR</td></tr>
<tr><td>0</td><td>-3.867919756</td><td>0</td><td>hsa04918</td><td>KEGG Pathway</td><td>Thyroid hormone synthesis</td><td>30.238</td><td>3</td><td>ALB|SERPINA7|TTR</td></tr>
<tr><td>-3.867028664</td><td>0</td><td>0</td><td>R-HSA-9009391</td><td>Reactome Gene Sets</td><td>Extra-nuclear estrogen signaling</td><td>30.20779221</td><td>3</td><td>EREG|HSPB1|HSP90AA1</td></tr>
<tr><td>-2.532202169</td><td>0</td><td>0</td><td>R-HSA-8939211</td><td>Reactome Gene Sets</td><td>ESR-mediated signaling</td><td>10.47747748</td><td>3</td><td>EREG|HSPB1|HSP90AA1</td></tr>
<tr><td>-2.184360425</td><td>0</td><td>0</td><td>R-HSA-9006931</td><td>Reactome Gene Sets</td><td>Signaling by Nuclear Receptors</td><td>7.858108108</td><td>3</td><td>EREG|HSPB1|HSP90AA1</td></tr>
<tr><td>-2.125498186</td><td>0</td><td>-2.065178372</td><td>R-HSA-5683057</td><td>Reactome Gene Sets</td><td>MAPK family signaling cascades</td><td>7.479099678</td><td>3</td><td>EREG|HSPB1|DNAJB1</td></tr>
<tr><td>0</td><td>0</td><td>-2.881611635</td><td>hsa05160</td><td>KEGG Pathway</td><td>Hepatitis C</td><td>13.91532444</td><td>3</td><td>BID|IFIT1|OCLN</td></tr>
<tr><td>0</td><td>-2.647197063</td><td>0</td><td>R-HSA-9711123</td><td>Reactome Gene Sets</td><td>Cellular response to chemical stress</td><td>11.51192893</td><td>3</td><td>ALB|CDKN2A|COX5A</td></tr>
<tr><td>0</td><td>0</td><td>-2.616268267</td><td>hsa04814</td><td>KEGG Pathway</td><td>Motor proteins</td><td>11.23115018</td><td>3</td><td>MYH1|MYH7|MYO5C</td></tr>
<tr><td>0</td><td>-2.446975052</td><td>-2.416415419</td><td>hsa04820</td><td>KEGG Pathway</td><td>Cytoskeleton in muscle cells</td><td>9.775215517</td><td>3</td><td>CKM|MYH1|MYH7</td></tr>
<tr><td>0</td><td>0</td><td>-2.57942054</td><td>hsa05130</td><td>KEGG Pathway</td><td>Pathogenic Escherichia coli infection</td><td>10.899195</td><td>3</td><td>IRAK1|MYO5C|OCLN</td></tr>
</table>
