# Supplementary material for: Establishment and characterization of Hanwoo cumulus cell line for heat stress studies
Source: Anim Biosci. 2026 Jun 15;39(7):250896. doi: 10.5713/ab.250896 (PMC13353149; doi:10.5713/ab.250896)
Supplement: Supplementary file 16 [file ab-250896-Supplementary-16.pdf]

Supplement 16. Cumulus downregulated DEP pathway enrichment (KEGG and Reactome)

| _LogP_HS_CON | _LogP_HS_REC | _LogP_REC_CON | GO | Category | Description | Enrichment | #GeneInGOAndHitList | Hits |
|---|---|---|---|---|---|---|---|---|
| -8.75997265 | -3.973772539 | -9.16734444 | R-HSA-381426 | Reactome Gene Sets | Regulation of Insulin-like Growth Factor (IGF) transport and uptake by Insulin-like Growth Factor Binding Proteins (IGFBPs) | 16.90158952 | 16 | AHSG\|APOA2\|APOE\|F2\|FGA\|SERPIND1\|IGFBP4\|IGFBP7\|CCN1\|MMP2\|SPP1\|VGF\|MSLN\|PRSS23\|FSTL1\|MXRA8 |
| -9.32784782 | -2.904665066 | -8.459905835 | R-HSA-8957275 | Reactome Gene Sets | Post-translational protein phosphorylation | 17.11677179 | 14 | AHSG\|APOA2\|APOE\|FGA\|SERPIND1\|IGFBP4\|IGFBP7\|CCN1\|SPP1\|VGF\|MSLN\|PRSS23\|FSTL1\|MXRA8 |
| -3.462063405 | -6.185427116 | -9.442743685 | R-HSA-216083 | Reactome Gene Sets | Integrin cell surface interactions | 21.74352828 | 9 | CD47\|COL1A1\|COL1A2\|COL5A1\|COL6A2\|FGB\|LUM\|SPP1\|VTN |
| -2.065805841 | -6.719662103 | -9.160457775 | R-HSA-1474244 | Reactome Gene Sets | Extracellular matrix organization | 7.058938233 | 17 | BGN\|CD47\|COL1A1\|COL1A2\|COL5A1\|COL6A2\|COL11A1\|COL12A1\|FGA\|FGB\|LUM\|MMP2\|SERPINE1\|SPARC\|SPP1\|VTN\|ADAM19 |
| 0 | -6.403241945 | -8.38870087 | R-HSA-3000178 | Reactome Gene Sets | ECM proteoglycans | 21.36206288 | 8 | BGN\|COL1A1\|COL1A2\|COL5A1\|COL6A2\|LUM\|SERPINE1\|VTN |
| 0 | -5.776323435 | -7.106483175 | R-HSA-8948216 | Reactome Gene Sets | Collagen chain trimerization | 27.67358145 | 6 | COL1A1\|COL1A2\|COL5A1\|COL6A2\|COL11A1\|COL12A1 |
| 0 | -6.779855164 | -6.115507165 | R-HSA-1442490 | Reactome Gene Sets | Collagen degradation | 40.03972458 | 5 | COL1A1\|COL1A2\|COL5A1\|COL11A1\|MMP2 |
| 0 | -2.956964074 | -6.600953037 | hsa04820 | KEGG Pathway | Cytoskeleton in muscle cells | 8.747396436 | 10 | BGN\|COL1A1\|COL1A2\|COL5A1\|COL6A2\|COL11A1\|MYBPC1\|TPM1\|TPM2\|ANKRD1 |
| 0 | -6.524098587 | -5.237535973 | R-HSA-1474228 | Reactome Gene Sets | Degradation of the extracellular matrix | 22.28297716 | 6 | COL1A1\|COL1A2\|COL5A1\|COL11A1\|MMP2\|SPP1 |
| 0 | -6.4623914 | -4.852511625 | R-HSA-8874081 | Reactome Gene Sets | MET activates PTK2 signaling | 68.33446328 | 4 | COL1A1\|COL1A2\|COL5A1\|COL11A1 |
| 0 | -5.231075281 | -6.283860938 | R-HSA-2022090 | Reactome Gene Sets | Assembly of collagen fibrils and other multimeric structures | 20.29395973 | 6 | COL1A1\|COL1A2\|COL5A1\|COL6A2\|COL11A1\|COL12A1 |
| 0 | -4.268107657 | -6.031162431 | hsa04974 | KEGG Pathway | Protein digestion and absorption | 13.52930649 | 7 | COL1A1\|COL1A2\|COL5A1\|COL6A2\|COL11A1\|COL12A1\|CPB2 |
| 0 | -5.039087923 | -5.996583081 | R-HSA-1650814 | Reactome Gene Sets | Collagen biosynthesis and modifying enzymes | 18.17369528 | 6 | COL1A1\|COL1A2\|COL5A1\|COL6A2\|COL11A1\|COL12A1 |
| 0 | -5.901690604 | -4.303147735 | R-HSA-8875878 | Reactome Gene Sets | MET promotes cell motility | 50.00082679 | 4 | COL1A1\|COL1A2\|COL5A1\|COL11A1 |
| 0 | 0 | -5.68364286 | R-HSA-9671793 | Reactome Gene Sets | Diseases of hemostasis | 42.72412575 | 4 | COL1A1\|COL1A2\|F2\|GGCX |
| 0 | -5.622494715 | -5.406689147 | R-HSA-9925563 | Reactome Gene Sets | Developmental Lineage of Pancreatic Ductal Cells | 42.70903955 | 4 | COL1A1\|COL1A2\|COL5A1\|COL11A1 |
| 0 | -4.549789582 | -5.270091823 | R-HSA-1474290 | Reactome Gene Sets | Collagen formation | 13.68132117 | 6 | COL1A1\|COL1A2\|COL5A1\|COL6A2\|COL11A1\|COL12A1 |
| 0 | -3.147990447 | -5.270091823 | hsa04512 | KEGG Pathway | ECM-receptor interaction | 13.68132117 | 6 | CD47\|COL1A1\|COL1A2\|COL6A2\|SPP1\|VTN |
| 0 | -5.136854809 | -5.258364402 | R-HSA-9734767 | Reactome Gene Sets | Developmental Cell Lineages | 10.36917651 | 7 | COL1A1\|COL1A2\|COL5A1\|COL11A1\|ERBB2\|VTN\|KRT24 |
| 0 | -5.104813925 | -3.900686395 | R-HSA-1566977 | Reactome Gene Sets | Fibronectin matrix formation | 76.87627119 | 3 | COL1A1\|COL1A2\|COL5A1 |
| 0 | -5.056247949 | -4.154061235 | R-HSA-9006934 | Reactome Gene Sets | Signaling by Receptor Tyrosine Kinases | 7.706894355 | 8 | COL1A1\|COL1A2\|COL5A1\|COL11A1\|ERBB2\|SPARC\|SPP1\|VGF |
| 0 | -4.699813209 | -5.041072096 | R-HSA-3000170 | Reactome Gene Sets | Syndecan interactions | 30.06512553 | 4 | COL1A1\|COL1A2\|COL5A1\|VTN |
| 0 | -4.754346208 | -3.194769496 | R-HSA-6806834 | Reactome Gene Sets | Signaling by MET | 25.94979618 | 4 | COL1A1\|COL1A2\|COL5A1\|COL11A1 |
| 0 | -4.732700688 | -4.314318928 | R-HSA-3000171 | Reactome Gene Sets | Non-integrin membrane-ECM interactions | 25.62542373 | 4 | COL1A1\|COL1A2\|COL5A1\|COL11A1 |
| 0 | -4.648870121 | -4.212550114 | R-HSA-9820448 | Reactome Gene Sets | Developmental Cell Lineages of the Exocrine Pancreas | 24.40516546 | 4 | COL1A1\|COL1A2\|COL5A1\|COL11A1 |
| 0 | -4.334063657 | -3.832473817 | hsa04933 | KEGG Pathway | AGE-RAGE signaling pathway in diabetic complications | 20.29736533 | 4 | COL1A1\|COL1A2\|MMP2\|CCL2 |
| 0 | -3.172352285 | -4.157966126 | hsa04510 | KEGG Pathway | Focal adhesion | 6.997917149 | 7 | CCND1\|COL1A1\|COL1A2\|COL6A2\|ERBB2\|SPP1\|VTN |
| 0 | -4.115033989 | -2.932105754 | R-HSA-2173782 | Reactome Gene Sets | Binding and Uptake of Ligands by Scavenger Receptors | 36.60774818 | 3 | COL1A1\|COL1A2\|SPARC |
| 0 | 0 | -3.969697255 | R-HSA-3000480 | Reactome Gene Sets | Scavenging by Class A Receptors | 32.04309431 | 3 | APOE\|COL1A1\|COL1A2 |
| 0 | -2.261344653 | -3.346850343 | hsa04151 | KEGG Pathway | PI3K-Akt signaling pathway | 4.484852979 | 8 | CCND1\|COL1A1\|COL1A2\|COL6A2\|ERBB2\|FGFR1\|SPP1\|VTN |
| 0 | 0 | -2.865333344 | hsa05165 | KEGG Pathway | Human papillomavirus infection | 4.265997541 | 7 | CCND1\|COL1A1\|COL1A2\|COL6A2\|SPP1\|VTN\|WNT5A |
| 0 | -2.674211698 | 0 | hsa04926 | KEGG Pathway | Relaxin signaling pathway | 11.82711864 | 3 | COL1A1\|COL1A2\|MMP2 |
| 0 | 0 | -2.34206745 | R-HSA-202733 | Reactome Gene Sets | Cell surface interactions at the vascular wall | 6.013025106 | 4 | CD47\|COL1A1\|COL1A2\|F2 |
| 0 | -2.121835443 | 0 | hsa05415 | KEGG Pathway | Diabetic cardiomyopathy | 7.500124018 | 3 | COL1A1\|COL1A2\|MMP2 |
| -5.86557804 | 0 | -5.298647926 | hsa04610 | KEGG Pathway | Complement and coagulation cascades | 13.50446606 | 9 | CPB2\|F2\|FGA\|FGB\|SERPIND1\|SERPINE1\|PLAU\|PLAUR\|VTN |
| -4.447391227 | 0 | -2.423522028 | R-HSA-109582 | Reactome Gene Sets | Hemostasis | 3.614721994 | 17 | AHSG\|CD47\|COL1A1\|COL1A2\|F2\|FGA\|FGB\|SERPIND1\|ITIH4\|SERPINE1\|PLAU\|PLAUR\|SPARC\|TIMP3\|WEE1\|HMG20B\|JMJD1C |
| 0 | 0 | -4.390164504 | R-HSA-76009 | Reactome Gene Sets | Platelet Aggregation (Plug Formation) | 16.9286754 | 5 | COL1A1\|COL1A2\|F2\|FGA\|FGB |
| -4.901450808 | 0 | 0 | R-HSA-114608 | Reactome Gene Sets | Platelet degranulation | 11.81864374 | 6 | AHSG\|FGA\|FGB\|ITIH4\|SERPINE1\|TIMP3 |
| -4.807353962 | 0 | 0 | R-HSA-76005 | Reactome Gene Sets | Response to elevated platelet cytosolic Ca2+ | 11.37764957 | 6 | AHSG\|FGA\|FGB\|ITIH4\|SERPINE1\|TIMP3 |
| -4.062222665 | 0 | 0 | R-HSA-140875 | Reactome Gene Sets | Common Pathway of Fibrin Clot Formation | 24.00793966 | 4 | F2\|FGA\|FGB\|SERPIND1 |
| -3.212831367 | 0 | -2.708882842 | R-HSA-76002 | Reactome Gene Sets | Platelet activation, signaling and aggregation | 5.039834661 | 10 | AHSG\|COL1A1\|COL1A2\|F2\|FGA\|FGB\|ITIH4\|SERPINE1\|SPARC\|TIMP3 |
| -3.310023034 | 0 | 0 | R-HSA-140877 | Reactome Gene Sets | Formation of Fibrin Clot (Clotting Cascade) | 13.54294032 | 4 | F2\|FGA\|FGB\|SERPIND1 |
| 0 | 0 | -2.448569764 | hsa04611 | KEGG Pathway | Platelet activation | 5.2398281 | 5 | COL1A1\|COL1A2\|F2\|FGA\|FGB |
| -2.058493371 | -4.304930793 | -7.120579244 | hsa05205 | KEGG Pathway | Proteoglycans in cancer | 7.767274595 | 12 | CCND1\|COL1A1\|COL1A2\|ERBB2\|FGFR1\|LUM\|MMP2\|PLAU\|PLAUR\|TIMP3\|VTN\|WNT5A |
| 0 | 0 | -2.719950138 | hsa05215 | KEGG Pathway | Prostate cancer | 6.228474911 | 5 | CCND1\|ERBB2\|FGFR1\|PLAU\|ZEB1 |
| 0 | 0 | -2.296603332 | hsa05206 | KEGG Pathway | MicroRNAs in cancer | 3.321853286 | 8 | CCND1\|CDKN2A\|ERBB2\|PLAU\|SOX4\|ZEB1\|TIMP3\|TPM1 |
| 0 | 0 | -2.201906876 | hsa05224 | KEGG Pathway | Breast cancer | 5.484853981 | 4 | CCND1\|ERBB2\|FGFR1\|WNT5A |
| -3.639907812 | -2.591724571 | -4.168735644 | R-HSA-453279 | Reactome Gene Sets | Mitotic G1 phase and G1/S transition | 9.499544469 | 10 | CCND1\|CDK1\|CDKN2A\|CKS1B\|RRM2\|WEE1\|CDC45\|FBXO5\|GMNN\|LIN54 |
| -2.866792376 | -2.763053368 | -4.505768013 | R-HSA-69206 | Reactome Gene Sets | G1/S Transition | 9.82142986 | 9 | CCND1\|CDK1\|CKS1B\|RRM2\|WEE1\|CDC45\|FBXO5\|GMNN\|LIN54 |
| 0 | -2.346844384 | -3.998642098 | R-HSA-69278 | Reactome Gene Sets | Cell Cycle, Mitotic | 4.051881513 | 17 | CCND1\|CDK1\|CDKN2A\|CKS1B\|RRM2\|UBE2E1\|WEE1\|CDC45\|UBE2C\|FBXO5\|GMNN\|SPDL1\|CENPH\|MZT2B\|CEP290\|AJUBA\|LIN54 |
| 0 | -2.670548275 | -3.811107143 | R-HSA-1640170 | Reactome Gene Sets | Cell Cycle | 3.66787967 | 19 | CCND1\|CDK1\|CDKN2A\|CKS1B\|RRM2\|UBE2E1\|WEE1\|CDC45\|UBE2C\|FBXO5\|GMNN\|PHF20\|SPDL1\|CLSPN\|CENPH\|MZT2B\|CEP290\|AJUBA\|LIN54 |
| -3.741881216 | 0 | -3.454796557 | R-HSA-69205 | Reactome Gene Sets | G1/S-Specific Transcription | 23.57922645 | 5 | CDK1\|RRM2\|CDC45\|FBXO5\|LIN54 |
| -2.527178909 | 0 | -3.985993764 | R-HSA-69242 | Reactome Gene Sets | S Phase | 7.042328967 | 8 | CCND1\|CKS1B\|UBE2E1\|WEE1\|CDC45\|UBE2C\|GMNN\|LIN54 |
| -2.261179301 | 0 | 0 | R-HSA-69620 | Reactome Gene Sets | Cell Cycle Checkpoints | 4.633111162 | 10 | CDK1\|CDKN2A\|UBE2E1\|WEE1\|CDC45\|UBE2C\|PHF20\|SPDL1\|CLSPN\|CENPH |
| 0 | 0 | -2.246545348 | R-HSA-69202 | Reactome Gene Sets | Cyclin E associated events during G1/S transition | 7.235269486 | 4 | CCND1\|CKS1B\|WEE1\|LIN54 |
| 0 | 0 | -2.21394482 | R-HSA-69656 | Reactome Gene Sets | Cyclin A:Cdk2-associated events at S phase entry | 7.042328967 | 4 | CCND1\|CKS1B\|WEE1\|LIN54 |
| -2.486099616 | 0 | -3.280236439 | hsa04115 | KEGG Pathway | p53 signaling pathway | 10.56349345 | 6 | CCND1\|CDK1\|CDKN2A\|SERPINE1\|RRM2\|RCHY1 |
| -3.450034467 | 0 | 0 | R-HSA-6804757 | Reactome Gene Sets | Regulation of TP53 Degradation | 21.78007203 | 3 | CDK1\|CDKN2A\|PHF20 |
| -3.413490625 | 0 | 0 | R-HSA-6806003 | Reactome Gene Sets | Regulation of TP53 Expression and Degradation | 21.17507003 | 3 | CDK1\|CDKN2A\|PHF20 |
| -2.456275412 | 0 | 0 | hsa04218 | KEGG Pathway | Cellular senescence | 5.046254833 | 6 | CCND1\|ZFP36L1\|CDK1\|CDKN2A\|SERPINE1\|LIN54 |
| 0 | 0 | -4.291725323 | R-HSA-3772470 | Reactome Gene Sets | Negative regulation of TCF-dependent signaling by WNT ligand antagonists | 40.58791946 | 3 | SFRP2\|WNT5A\|DKK2 |
| 0 | 0 | -3.635051481 | hsa04310 | KEGG Pathway | Wnt signaling pathway | 6.997917149 | 6 | CCND1\|SFRP2\|WNT5A\|FOSL1\|DKK2\|LZTS2 |
| 0 | -4.252032254 | -2.719950138 | R-HSA-6785807 | Reactome Gene Sets | Interleukin-4 and Interleukin-13 signaling | 19.33994244 | 4 | CEBPD\|COL1A2\|MMP2\|CCL2 |
| 0 | 0 | -3.86233234 | R-HSA-9006936 | Reactome Gene Sets | Signaling by TGFB family members | 7.706566987 | 6 | COL1A2\|INHBA\|SERPINE1\|TCF4\|FST\|FSTL1 |
| 0 | 0 | -3.059658918 | R-HSA-8864260 | Reactome Gene Sets | Transcriptional regulation by the AP-2 (TFAP2) family of transcription factors | 13.89933349 | 4 | APOE\|ERBB2\|ATAD2\|WWOX |
| -3.4877076 | 0 | 0 | R-HSA-8963899 | Reactome Gene Sets | Plasma lipoprotein remodeling | 22.42066238 | 3 | APOA2\|APOE\|PLTP |
| -2.967248845 | 0 | 0 | hsa04979 | KEGG Pathway | Cholesterol metabolism | 14.94710826 | 3 | APOA2\|APOE\|PLTP |
| -2.486099616 | 0 | 0 | R-HSA-174824 | Reactome Gene Sets | Plasma lipoprotein assembly, remodeling, and clearance | 10.16403361 | 3 | APOA2\|APOE\|PLTP |
| -2.119095177 | 0 | 0 | R-HSA-382551 | Reactome Gene Sets | Transport of small molecules | 2.831207135 | 8 | ABCA2\|APOA2\|APOE\|ATP8B1\|PLTP\|SLC20A1\|ABCB6\|FLVCR1 |
| 0 | 0 | -2.51996685 | R-HSA-4839726 | Reactome Gene Sets | Chromatin organization | 4.155220326 | 9 | CCND1\|MORF4L2\|HMG20B\|PHF20\|PHF10\|ATF7IP\|ASH1L\|KAT8\|RIOX2 |
| 0 | 0 | -2.635642165 | R-HSA-3247509 | Reactome Gene Sets | Chromatin modifying enzymes | 3.897968063 | 8 | CCND1\|MORF4L2\|HMG20B\|PHF20\|ATF7IP\|ASH1L\|KAT8\|RIOX2 |
| 0 | 0 | -3.128969082 | R-HSA-390522 | Reactome Gene Sets | Striated Muscle Contraction | 16.91163311 | 3 | MYBPC1\|TPM1\|TPM2 |
| 0 | 0 | -2.789937903 | R-HSA-977606 | Reactome Gene Sets | Regulation of Complement cascade | 12.95359132 | 3 | CPB2\|F2\|VTN |
| 0 | 0 | -2.527659692 | R-HSA-166658 | Reactome Gene Sets | Complement cascade | 10.49687572 | 3 | CPB2\|F2\|VTN |
| 0 | 0 | -2.46548569 | R-HSA-163841 | Reactome Gene Sets | Gamma carboxylation, hypusinylation, hydroxylation, and arylsulfatase activation | 9.980635934 | 3 | F2\|GGCX\|RIOX2 |
| 0 | 0 | -2.527659692 | R-HSA-186797 | Reactome Gene Sets | Signaling by PDGF | 10.49687572 | 3 | COL5A1\|COL6A2\|SPP1 |
| 0 | 0 | -2.425867054 | R-HSA-375165 | Reactome Gene Sets | NCAM signaling for neurite out-growth | 9.663790348 | 3 | COL5A1\|COL6A2\|FGFR1 |
| 0 | 0 | -2.050396585 | R-HSA-8878159 | Reactome Gene Sets | Transcriptional regulation by RUNX3 | 6.141565959 | 4 | CCND1\|CDKN2A\|CCN2\|SPP1 |
| 0 | 0 | -2.286377042 | hsa04371 | KEGG Pathway | Apelin signaling pathway | 5.798274209 | 4 | CCND1\|CCN2\|SERPINE1\|SPP1 |

| _LogP_HS_CON | _LogP_HS_REC | _LogP_REC_CON | GO | Category | Description | Enrichment | #GeneInGOAndHitList | Hits |
|---|---|---|---|---|---|---|---|---|
| 0 | 0 | -2.113033324 | hsa04390 | KEGG Pathway | Hippo signaling pathway | 4.205212361 | 5 | CCND1\|CCN2\|SERPINE1\|WNT5A\|AJUBA |
| -2.305096395 | 0 | 0 | R-HSA-9759194 | Reactome Gene Sets | Nuclear events mediated by NFE2L2 | 8.762097943 | 3 | BACH1\|CDKN2A\|CCL2 |
| 0 | 0 | -2.263218815 | R-HSA-199992 | Reactome Gene Sets | trans-Golgi Network Vesicle Budding | 8.455816555 | 3 | AP4M1\|SNAPIN\|BLOC1S4 |
| 0 | 0 | -2.178837707 | hsa05171 | KEGG Pathway | Coronavirus disease - COVID-19 | 4.263436918 | 5 | F2\|FGB\|CCL2\|RSL24D1\|RPL22L1 |
| 0 | 0 | -2.064270371 | hsa01232 | KEGG Pathway | Nucleotide metabolism | 7.162574023 | 3 | IMPDH1\|RRM2\|NUDT16 |
| -2.05445102 | 0 | 0 | R-HSA-8953854 | Reactome Gene Sets | Metabolism of RNA | 2.758218077 | 8 | SRSF5\|RNGTT\|PDCD11\|TRMT13\|PUS7\|IMP3\|TSEN34\|MRM1 |
| -2.013929151 | 0 | 0 | R-HSA-72306 | Reactome Gene Sets | tRNA processing | 6.867590279 | 3 | TRMT13\|PUS7\|TSEN34 |
